# Electrochemical synthesis of peptide aldehydes via C‒N bond cleavage of cyclic amines

**DOI:** 10.1038/s41467-024-49223-y

**Published:** 2024-06-18

**Authors:** Xinyue Fang, Yong Zeng, Yawen Huang, Zile Zhu, Shengsheng Lin, Wenyan Xu, Chengwei Zheng, Xinwei Hu, Youai Qiu, Zhixiong Ruan

**Affiliations:** 1 Guangzhou Municipal and Guangdong Provincial Key Laboratory of Molecular Target & Clinical Pharmacology and the State Key Laboratory of Respiratory Disease, School of Pharmaceutical Sciences & the Fifth Affiliated Hospital, Guangzhou Medical University, Guangzhou, 511436 PR China; 2 State Key Laboratory and Institute of Elemento-Organic Chemistry, Frontiers Science Center for New Organic Matter, College of Chemistry, Nankai University, 94 Weijin Road, Tianjin, 300071 PR China

**Keywords:** Synthetic chemistry methodology, Electrocatalysis, Synthetic chemistry methodology, Sustainability

## Abstract

Peptide aldehydes are crucial biomolecules essential to various biological systems, driving a continuous demand for efficient synthesis methods. Herein, we develop a metal-free, facile, and biocompatible strategy for direct electrochemical synthesis of unnatural peptide aldehydes. This electro-oxidative approach enabled a step- and atom-economical ring-opening via C‒N bond cleavage, allowing for homoproline-specific peptide diversification and expansion of substrate scope to include amides, esters, and cyclic amines of various sizes. The remarkable efficacy of the electro-synthetic protocol set the stage for the efficient modification and assembly of linear and macrocyclic peptides using a concise synthetic sequence with racemization-free conditions. Moreover, the combination of experiments and density functional theory (DFT) calculations indicates that different *N*-acyl groups play a decisive role in the reaction activity.

## Abstract (web summary)

Peptide aldehydes are an important class of biomolecules, playing essential roles in various living systems, and there is an ongoing demand for the efficient synthesis of peptide aldehydes. Herein, the authors report the electrooxidative ring-opening reaction via C‒N bond cleavage for the synthesis of unnatural peptide aldehydes.

## Introduction

**Fig. 1:**
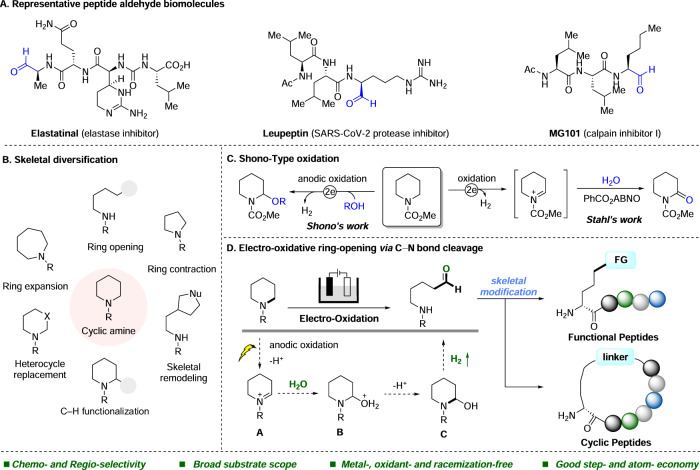
Cyclic amine modification for peptide aldehyde synthesis. **A** Representative peptide aldehyde biomolecules. **B** Skeletal diversification of cyclic amines (**C**) Shono-type oxidation. **D** This work: electro-oxidative ring-opening via C‒N bond cleavage. ABNO = 9-Azabicyclo[3.3.1]nonane *N*-oxyl.

Peptide aldehydes are a significantly important class of biomolecules, playing a diverse range of essential roles in various living systems^1^. They serve as effective enzyme inhibitors, particularly protease inhibitors, such as Leupeptin^2,3^, Elastatinal^4^, and MG101^5,6^ (Fig. 1A), thus regulating important biological processes such as digestion, blood clotting, inflammation as well as exhibiting anti-coronavirus activity^7^. Furthermore, aldehydes present in peptides provide a convenient handle for peptide backbone modification or site-specific ligation reactions^8–10^. However, due to the pronounced reactivity of aldehydes, late-stage incorporation in chemical synthesis is required through post-assembly chemical modification, either by employing a specific chemical reaction or by deprotection after peptide elongation to reveal the aldehyde^1,11–16^. Therefore, there is an ongoing and strong demand for innovative methods that enable highly efficient synthesis of peptide aldehydes.

Organic electrochemistry, utilizing protons and electrons as redox reagents, is emerging as a powerful approach for small-molecule synthesis^17–30^. However, it remains underexplored as a tool for achieving mild and chemoselective modification of existing peptides^31–33^. As a mild and customizable reaction platform^34^, electrochemistry offers great promise in overcoming the chemo- and regioselectivity issues encountered in conventional bioconjugation strategies^35–39^. Recent contributions in electrochemical modification of peptides were mainly achieved by tyrosine-^40–45^ and tryptophan-^46,47^ specific functionalization as well as side chain diversification via direct or indirect electrochemical methods. Recognizing the necessity for additional research methodologies to facilitate the coupling of specific functional groups, various structural functionalization reactions of cyclic amines^48^ were initiated, in order to access distinctive chemical and functional spaces. These reactions encompassed ring-opening reactions^49–51^, ring-expansion reactions^52^, ring contraction^53^, heterocycle replacement^54^, and C2-direct functionalization reactions^55,56^. These innovative approaches involved the breaking of conventional saturated C‒C or C‒N bonds for chemical bond reorganization (Fig. 1B). In addition, with regard to piperidine derivatives, prevalent in pesticides and peptide-based pharmaceuticals^57,58^, there is a wealth of examples employing the Shono-type oxidation strategy to achieve C2 functionalization of piperidines (Fig. 1C)^59–61^. However, there are few reports on electrochemical C‒N bond ring-opening reactions. Within our program on sustainable electrochemical modification of peptides^62,63^ and cyclic amines^64,65^, herein we present an electro-oxidative ring-opening approach to achieve unnatural peptide aldehydes (Fig. 1D). Notable features of our general strategy include (i) (homo)proline-specific diversification of peptide backbones using exceedingly mild and biocompatible conditions in a metal-, chemical oxidant-, and racemization-free fashion, (ii) a broad substrate scope with various peptides and complex bioactive molecules, (iii) step- and atom- economical ring-opening procedure for smoothly yielding peptide aldehydes and remote amino aldehydes via selective C‒N bond cleavage, and (iv) setting the stage for versatile syntheses of macrocyclic peptides. Detailed mechanistic insights of this process provided strong support for the presence and pivotal role of an *N*-Piv protecting group.

## Results

### Optimization of reaction conditions

We commenced our investigations by probing a variety of different electrolysis conditions, employing an undivided cell equipped with a graphite felt anode and a platinum cathode, for the envisioned electro-oxidative ring-opening of *D*-homoproline-containing dipeptide (Piv-Homopro-Leu-OMe) **1a**. The optimal results were obtained when dipeptide **1a** was directly electrolyzed at a constant current of 8.0 mA in a mixed electrolyte solution of *n*Bu_4_NHSO_4_ in MeCN/H_2_O (9:1) at room temperature under atmospheric conditions without additional oxidants and transition metal catalysts. Under these conditions, the desired dipeptide aldehyde **1b** was isolated in 73% yield without racemization (Table 1, entry 1) (For details, please see the Supplementary Table 1). The electrolyte and solvent were both found to be critical factors for the reaction to achieve the optimal yield (entries 2‒6). Among a variety of electrolytes, *n*Bu_4_NPF_6_ and *n*Bu_4_NOAc and *n*Bu_4_NOH showed poor efficacy (entries 2‒4), while LiClO_4_, *n*Bu_4_NI, or no electrolyte displayed inactive performance, highlighting the critical importance of HSO_4_¯ (entries 5‒6), which also confirmed by the further DFT calculations. In addition, solvent screening experiments verified the essential role of H_2_O as the oxygen source, since performing the electrolysis in the solvent with traceless H_2_O produced the remote amino aldehyde **1b** in extremely low yields (entries 7‒9). The choice of electrode material proved critical because a dramatically reduced product yield was obtained when using other types of carbon electrodes (entries 10‒12). A further control experiment indicated that electricity was indispensable to promote the desired reaction (entry 13).

**Table 1:** Optimization of reaction conditions^[a]^


Entry	Deviation from standard conditions	Yield [%]^[b]^
**1**	**None**	**73**
2	*n*Bu_4_NPF_6_ instead of *n*Bu_4_NHSO_4_	43
3	*n*Bu_4_NOAc instead of *n*Bu_4_NHSO_4_	22
4	*n*Bu_4_NOH instead of *n*Bu_4_NHSO_4_	29
5	LiClO_4_ instead of *n*Bu_4_NHSO_4_	0
6	*n*Bu_4_NI instead of *n*Bu_4_NHSO_4_	0
7	no *n*Bu_4_NHSO_4_	0
8	MeCN/HFIP (9:1) instead of MeCN/H_2_O (9:1)	10
9	MeCN instead of MeCN/H_2_O (9:1)	22
10	C as an anode	58
11	RVC as an anode	25
12	GF as a cathode	11
13	No electricity	0

Bold formatting shows that entry 1 is the optimal reaction conditions.

^[a]^ Reaction conditions: Undivided cell, graphite felt (GF) anode, platinum plate (Pt) cathode, **1a** (0.3 mmol), *n*Bu_4_NHSO_4_ (0.3 mmol), MeCN/H_2_O (9:1, 10 mL), constant current = 8.0 mA, 6 h (6.0 *F*), RT, under air. ^[b]^ Yields of isolated products. C = graphite plate. RVC = reticulated vitreous carbon. HFIP = 1,1,1,3,3,3-Hexafluoro-2-propanol.

### Substrate scope

**Fig. 2:**
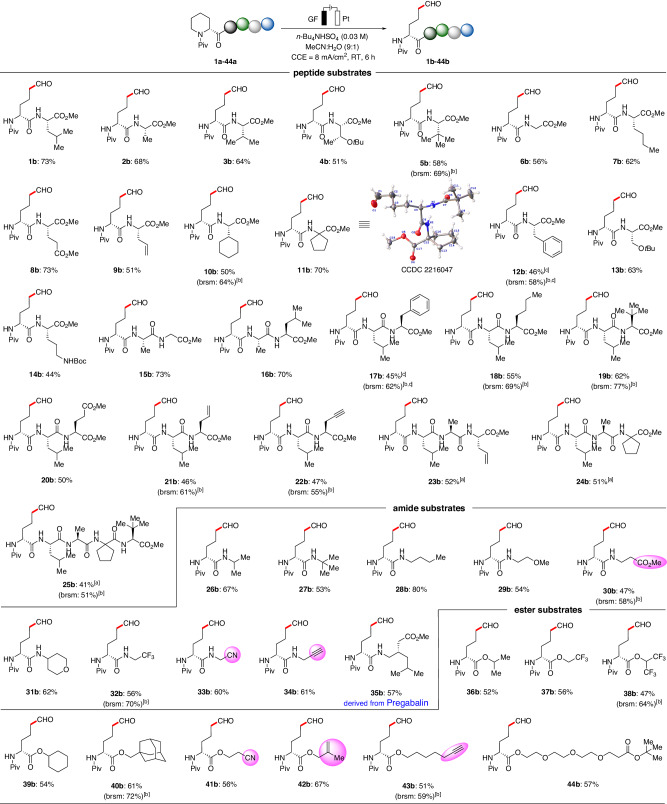
Scope of electrochemical ring-opening with peptides, amides, and esters. Reaction conditions: Undivided cell, graphite felt (GF) anode, platinum plate (Pt) cathode, **1a-44a** (0.3 mmol), *n*Bu_4_NHSO_4_ (0.3 mmol), MeCN/H_2_O (9:1, 10 mL), constant current = 8.0 mA, 6 h (6.0 *F*), RT, under air. Isolated yields are reported. ^[a]^ 9.0 h. ^[b]^ Yields based on recovered starting materials. ^[c]^ ketoABNO (50 mol %).

With the optimal conditions in hand, we explored the scope of the electro-oxidative C‒N bond cleavage protocol (Fig. 2). The electrochemical ring-opening reaction exhibited excellent compatibility with a variety of natural aliphatic amino acids such as leucine (Leu), alanine (Ala), valine (Val), threonine (Thr), glycine (Gly), and glutamic acid (Glu) as well as unnatural medicinally-useful bulky amino acids, e.g., L-*tert*-leucine, L-allylglycine, L-cyclohexylglycine, and cycloleucine, affording the dipeptide aldehydes in moderate to good yields (**1b**–**14b**). The structure of **11b** was unambiguously confirmed by single-crystal X-ray diffraction studies. Thereafter, we investigated the scope of tripeptides conjugated with a diversity of amino acid residues (**15b**–**22b**). The robust nature of the electro-oxidative ring-opening transformation was reflected by fully tolerating a wealth of valuable transformation groups, including alkenes and alkynes, which could serve as a handle for future late-stage bioconjugation. More importantly, the peptides containing electron-rich phenylalanine residues were compatible under slightly modified electrolytic conditions, which normally readily decomposed under direct electrochemical conditions. To our delight, the strategy for the direct electrochemical synthesis of peptide aldehyde smoothly provided L-allylglycine-containing tetrapeptides and bulky alkyl group-containing pentapeptides, further emphasizing the strong biocompatible conditions (**23b**–**25b**). Furthermore, a wide range of common amide and ester substrates with bulky (*iso*-propyl, *tert*-butyl, cyclohexyl, adamantyl) or linear alkyl groups (*n*-butyl), including electron-donating (-OMe) and electron-withdrawing substituents (-CO_2_Me, -CF_3_, -CN), as well as synthetic useful handles (alkenyl, alkynyl, linker), were found to be fully tolerated by the optimized electrooxidation (**26b**–**44b**). Gratifyingly, the practical utility of our approach was further illustrated by successfully performing the desired ring-opening with substrates derived from marketed drug pregabalin, which effectively delivered *ε*-amino aldehyde derivative **35b** in moderate yield. It is noteworthy that the antioxidant activity of aldehyde products was evaluated by DPPH (2,2-Diphenyl-1-picrylhydrazyl) radical-scavenging activity assays, some peptides, such as **21b,** **22b,** and **24b**, exhibited mild antioxidant activity at a concentration-dependent manner (For details, please see the Supplementary Figs. 14 and 15).

**Fig. 3:**
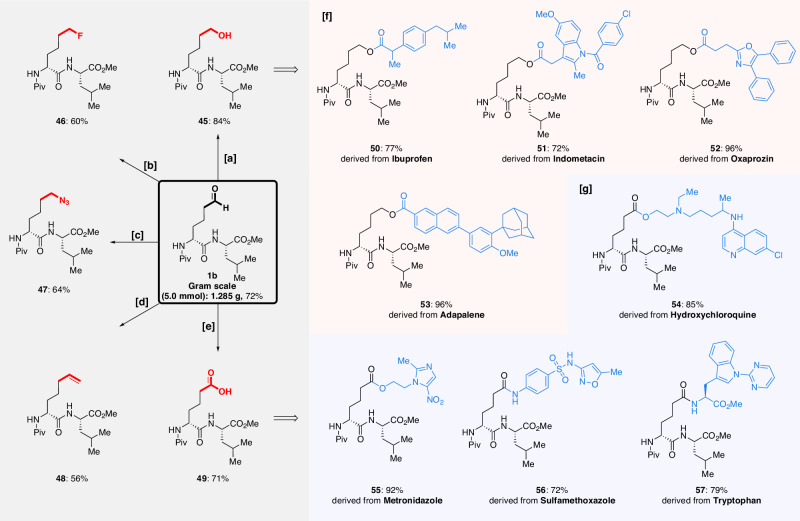
Diversifications of desired peptide aldehyde. Isolated yields are reported. [a] NaBH_4_, MeOH, 0 °C, 1 h. [b] (i) NaBH_4_, MeOH, 0 °C, 1 h; (ii) DAST, DCM, −78 °C to RT, 14 h. [c] (i) NaBH_4_, MeOH, 0 °C, 1 h; (ii) MsCl, Et_3_N, DCM, 0 °C to RT, 4 h; (iii) NaN_3_, DMF, RT, 12 h. [d] PPh_3_MeBr, *t*-BuONa, THF, 0 °C to RT, 12 h. [e] O_2_, NHPI, MeCN, RT, 12 h. [f] Transformations of hydroxyl peptides. [g] Applications of acid products. For details, please see the Supplementary Information on pages 57−69.

Furthermore, encouraged by these exciting results, we sought to investigate the synthetic applications of the peptide aldehyde product by functional group transformation (Fig. 3). Thus, the outstanding potential of our ring-opening approach was demonstrated by its easy scalability and versatile transformations of the generated peptide aldehyde. For instance, we electrolyzed 5.0 mmol of dipeptide **1a** to deliver the corresponding peptide aldehyde **1b** in 72% yield, without appreciable loss in efficacy. In addition, five novel peptide sequences (**45**–**49**) were each rapidly constructed in simple steps from **1b**, highlighting the potential for such reactions to enable efficient diversification of native residues in preassembled peptide building blocks, including the synthesis of unnatural amino acids. Strikingly, a series of peptide-conjugated drugs (**50**–**56**) were further assembled through ligation with marketed drugs via amination or esterification.

**Fig. 4:**
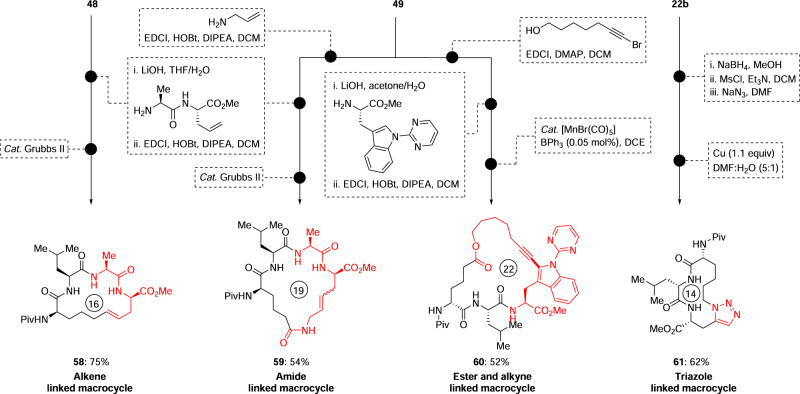
The synthesis of macrocyclic peptides 58–61.

In the realm of non-ribosomal peptide synthetase natural products, macrocyclic peptides stand out as highly prized therapeutic contenders compared to their linear counterparts. This preference is rooted in their heightened resistance to chemical and enzymatic degradation, improved receptor specificity, and advantageous pharmacokinetic profiles^49,66–69^. We sought to investigate the feasibility of efficiently constructing and expanding macrocycles by rapidly introducing new functional groups into peptides starting from a simple homoproline residue. Gratifyingly, olefin- and carboxylic acid-derived dipeptides (**48** and **49**) as well as tripeptide aldehyde (**22b**) could be rapidly transformed into four macrocycles containing (*E*)-alkene (**58**), amide (**59**), ester and alkyne (**60**)^70^, as well as triazole (**61**) linkers, respectively, through concise synthetic sequences by key olefin metathesis, manganese-catalyzed C–H alkynylation, and click reaction steps (Fig. 4) (For details, please see the Supplementary Information on pages 69−80). The incorporation of functional groups into the linker region of stapled peptide-like structures has been demonstrated to influence the biological properties of the resulting products. Our post-assembly electro-oxidative strategy enables the rapid synthesis of a diverse array of functionally and structurally enriched molecules, as exemplified by the small library of compounds synthesized from dipeptide aldehyde **1b**.

**Fig. 5:**
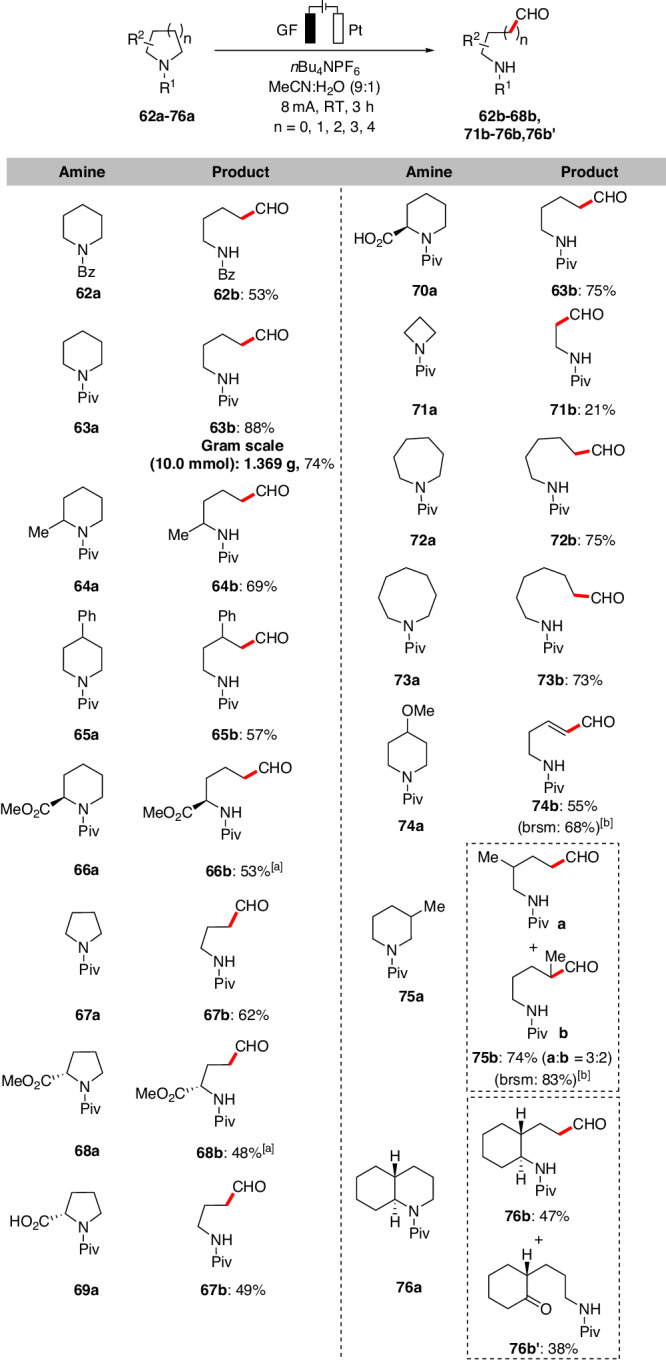
Scope of simple cyclic amine derivatives. Reaction conditions: Undivided cell, graphite felt (GF) anode, platinum plate (Pt) cathode, **62a-76a** (0.3 mmol), *n*Bu_4_NPF_6_ (0.3 mmol), MeCN/H_2_O (9:1, 10 mL), constant current = 8.0 mA, 3 h (3.0 *F*), RT, under air. Isolated yields are reported. ^[a]^ 6.0 h. ^[b]^ Yield based on recovered starting materials.

Remote amino aldehydes, especially unnatural derivatives, serving as direct precursors of variant amino acids, are indispensable building blocks in the synthesis of peptide aldehydes^1,71,72^. Thus, the scope of cyclic amines^73–75^ was also investigated using a slightly modified set of conditions (Fig. 5 and Supplementary Table 2 in Supplementary Information). Diverse substitution patterns on the piperidine ring exhibited excellent tolerance, enabling the synthesis of the corresponding acyclic amino aldehydes with moderate to good yields (up to 88%). Notably, benzoyl-protected piperidine afforded the corresponding aldehyde in moderate yield (**62b**), while that with other protected groups Boc and Cbz resulted in *ortho*-hydroxylation products instead of the desired aldehydes (For details, please see the Supplementary Table 3). Piperidines with ester functional groups on the saturated azacyclic backbone (**66a** and **68a**) exhibited effective performance. However, cyclic amines with free carboxylic acid groups at the α-positions (**69a** and **70a**) produced the respective decarboxylated amino aldehydes. Piperidines containing benzylic sites prone to oxidation yielded the corresponding aldehydes with a 57% yield (**65b**). Remarkably, complete positional selectivity was observed with 2-substituted azacycles (**64a,** **66a**, and **68a**) in the oxidative ring-opening protocol. The scalability of our ring-opening approach was demonstrated by a successful gram-scale reaction (**63b**). The method also tolerated saturated azacycles of various ring sizes (four to eight-membered rings), resulting in *β*-, *γ*-, or remote amino aldehydes (**67b,** **72b**, and **73b**), although the isolated yield for *β*-amino aldehyde (**71b**) was low. Moreover, the *α,β*-unsaturated aldehyde **74b** was obtained when the piperidine’s para-position was substituted with a methoxy group. Finally, both substituted cyclic amines **75a** and **76a** could be converted into corresponding ring-opening products, although the chemo-selectivity was unromantic.

### Mechanistic studies

**Fig. 6:**
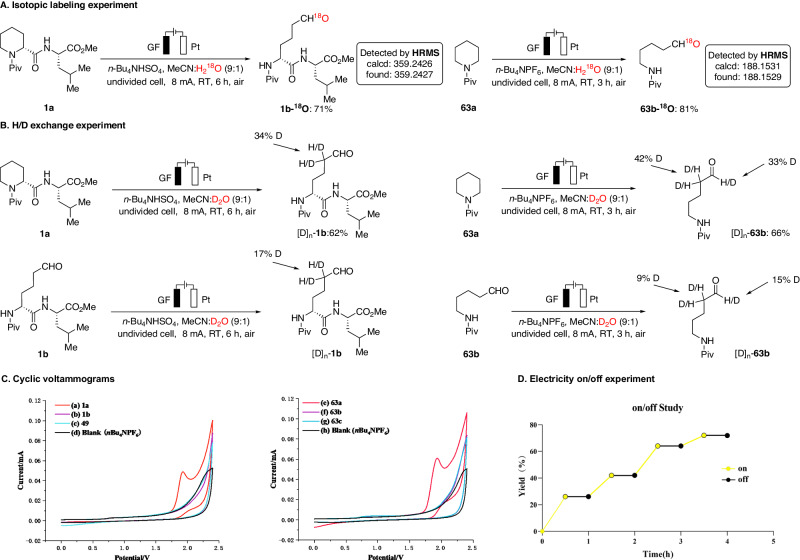
Control experiments. **A** Isotopic labeling experiment. **B** H/D exchange experiment. **C** Cyclic voltammograms. **D** Electricity on/off experiment.

In light of the outstanding versatility of the electrochemical ring-opening methodology, we were intrigued to delineate its mode of action. To this end, an isotopic labeling experiment was conducted in the presence of H_2_^18^O under the standard conditions. The results indicated that the oxygen atom in the newly formed aldehyde derived from the isotopically labeled water (Fig. 6A). Experiments with isotopically labeled co-solvents unraveled a facile C–H activation step, as evidenced by H/D scrambling at the *α*-position of the aldehyde (Fig. 6B). Furthermore, analysis of the voltammogram results disclosed that the dipeptide **1a** underwent anodic oxidation at ~1.928 V (vs Ag/AgCl), while no distinct oxidation peak was observed for **1b**, indicating the difficulty of further oxidizing aldehyde **1b** to acid **49** (Fig. 6C). This observation provided a plausible explanation for the absence of carboxylic acid products in the electrolytic reaction, even when using higher currents. Additionally, an electricity on/off study revealed that any chain processes were transient and short-lived (Fig. 6D). This finding suggested that electrolysis is required for sustained product formation, indicating that the reaction was less likely to proceed through a radical chain mechanism.

**Fig. 7:**
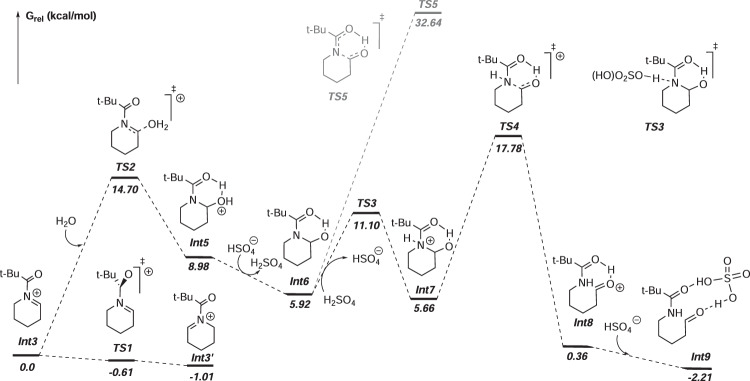
DFT-calculated potential energy surface of Int3’s conversion process. All energy units are kcal/mol.

Furthermore, DFT calculations were performed to further elucidate the reaction mechanism using **63a** as a model substrate (see Fig. 7 and Supplementary Table 7 in Supplementary Information). As is shown in Fig. 7, **63a** may first undergo two single electron anodic oxidation and the iminium intermediate **Int3** emerges. According to the potential energy surface, across **TS2**, water capture of **Int3** is endothermic (Δ*G* = 8.98, ΔG* = 14.70). In the yielding **Int5**, an O$-$H···O intramolecular hydrogen bond is found to stabilize the intermediate, while the analogous intermediate from water capture of **Int3′** cannot be located. Taking HSO_4_^–^ in _*n*_Bu_4_NHSO_4_ as a Bronsted base, the resulting **Int5** may deprotonate barrierlessly into **Int6**, and reprotonate into **Int7** via **TS3**, dissipating 3.32 kcal/mol Gibbs free energy. The proton transferring is a fast process due to low energy barriers. A C$-$N bond cleavage will take place in **Int7**, mounting an energy barrier of 12.12 kcal/mol via **TS4**, yielding protonated product **Int8**, which may associate with HSO_4_^–^ into **Int9**. Moreover, another reaction pathway was also considered, in which the C$-$N bond cleavage takes place in **Int6** via **TS5** yielding an enol-like product. However, the energy barrier (26.72 kcal/mol) is too high to cross.

We then altered the *N*-acyl group into Boc, Bz, and Cbz, and calculated the relative Gibbs free energy of some corresponding intermediates and transition states. Results are listed in Table 2. In each variation, **Int3** is selected as the energy zero-point.

**Table 2:** Relative calculated Gibbs free energy data of some corresponding intermediates and transition states, with the *N*-Piv group altered to Boc, Bz, and Cbz

*N*-acyl group & G_rel_ (kcal/mol)	Int3′	Int3	TS2	Int5	Int6	Int7	TS4
Boc	0.22	0.0	16.10	13.24	6.58	10.77	23.63
Bz	-0.82	0.0	14.31	9.47	5.10	8.08	20.67
Cbz	0.51	0.0	15.15	14.85	4.74	9.69	22.34
**Piv**	-1.01	0.0	14.70	8.98	5.92	5.66	**17.78**

Bold formatting shows that the TS4 Gibbs free energy data for the *N*-Piv group are the lowest.

With DFT results in hands, the insight into reactivity of substrates can be gained. According to the proposed mechanism, applying the steady-state approximation and Eyring equation, a rate equation in proportion to the concentration of iminium (**Int3**) could be derived (To check the process of deduction, please see the Supplementary Information on pages 91 − 93):

1 $${r}_{{Int}3\to {Product}}={k}_{{obs}}\left[{{{{{{\rm{H}}}}}}}_{2}{{{{{\rm{O}}}}}}\right]{\cdot }c\left({{{{{\rm{Int}}}}}}3\right)$$

2 $${k}_{{obs}}=\frac{{k}_{B}T}{h[1+\! \exp (-\frac{\Delta G({Int}3\to {Int}3^{\prime} )}{{RT}})]}\exp \left[-\frac{G({TS}4)-G({Int}3)}{{RT}}\right]$$

Where *r* is the reaction rate of **Int3**’s conversion to the product form, *k*_obs_ denotes the observed rate constant, *k*_B_ is the Boltzmann constant, *T* = 298.15 K, *h* is the Planck constant, *R* is the gas constant, [H_2_O] is the concentration of water, and *c*(Int3) is the analytic concentration of **Int3**, the iminium intermediate. According to the formula (Eq. 2), for each variation of the *N*-acyl group, the free energy difference between **TS4** and **Int3** plays a pivotal role in deciding the reactivity. Using the DFT-calculated data from Table 2, different *N*-acyl groups bear different *k*_obs_ calculated in Table 3. Among Boc, Bz, Cbz, and Piv-substituted cyclic amines, the Piv-substituted reacts the fastest (8.9 × 10^–2 ^L mol^–1^ s^–1^), but with an oxygen atom inserted (the Boc-substituted), *k*_obs_ drops prominently to 7.2 × 10^–7 ^L mol^–1^ s^–1^, which implies the scarce reactivity.

**Table 3:** Computed *k*_obs_ of different Int3’s conversion with varying *N*-acyl groups

Group	Boc	Bz	Cbz	Piv
*k*_obs_/(L·mol^−1^·s^−1^)	7.2 × 10^−7^	8.8 × 10^−4^	1.8 × 10^−4^	**8.9** **×** **10**^**−2**^

Bold formatting shows that the Piv-substituted reacts the fastest.

## Discussion

In summary, we have reported a pioneering electrochemical ring-opening protocol that enables direct synthesis of unnatural peptide aldehydes and remote amino aldehydes through mild and biocompatible reaction conditions involving C‒N bond cleavage. The strategy offers expedient access to a wide variety of peptide aldehydes with potential antioxidant activities, as well as expanded substrate scope including amides, esters, and cyclic amines with diverse synthetic functional groups. The unique power of the electro-oxidative ring-opening approach set the stage for efficient modification and assembly of acyclic and macrocyclic peptides by short synthetic sequence under racemization-free conditions. Notably, the experiments and DFT calculations demonstrate that different *N*-acyl groups play a decisive role for the reaction.

## Methods

### General procedure for electrochemical reactions

In an undivided cell (30 mL) equipped with a stirring bar, a mixture of substrates (0.3 mmol), *n*Bu_4_NHSO_4_ (0.3 mmol, 0.03 M), and MeCN/H_2_O (9:1, 10 mL) were added. The cell was equipped with graphite felt plate (GF, 1.5 cm × 1.0 cm × 0.2 cm) as the anode and platinum plate (Pt, 1.5 cm × 1.0 cm × 0.01 cm) as the cathode connected to an AXIOMET AX-3003P DC regulated power supply. The reaction mixture was stirred and electrolyzed at a constant current of 8 mA at room temperature for 3~6 h. Upon completion, the solvent was removed directly under reduced pressure to afford the crude product, which was further purified by flash column chromatography to afford the desired products.

### Supplementary information

Supplementary Information

Peer Review File

### Source data

Source data

## Data Availability

The authors declare that the data supporting the findings of this study are available within the paper and its Supplementary Information. Extra data are available from the corresponding author upon request. Source data are provided with this paper. Crystallographic data for the structures reported in this Article have been deposited at the Cambridge Crystallographic Data Centre, under deposition numbers CCDC 2216047 (11b). These data can be obtained free of charge from The Cambridge Crystallographic Data Centre via www.ccdc.cam.ac.uk/data_request/cif. Source data are provided with this paper.
