# Supplementary material for: Electrochemical synthesis of peptide aldehydes via C‒N bond cleavage of cyclic amines
Source: Nat Commun. 2024 Jun 18;15:5181. doi: 10.1038/s41467-024-49223-y (PMC11189564; doi:10.1038/s41467-024-49223-y)
Supplement: Supplementary file 1 — Supplementary Information [file 41467_2024_49223_MOESM1_ESM.pdf]

Supplementary Information

Electrochemical Synthesis of Peptide Aldehydes via C–N Bond Cleavage of Cyclic Amines

Xinyue Fang[1†], Yong Zeng[1†], Yawen Huang[1], Zile Zhu[2], Shengsheng Lin[1], Wenyan Xu[1], Chengwei Zheng[1], Xinwei Hu[1*], Youai Qiu[2*], Zhixiong Ruan[1*]

[1] Guangzhou Municipal and Guangdong Provincial Key Laboratory of Molecular Target & Clinical Pharmacology and the State Key Laboratory of Respiratory Disease, School of Pharmaceutical Sciences & the Fifth Affiliated Hospital, Guangzhou Medical University, Guangzhou 511436, P. R. China.

[2] State Key Laboratory and Institute of Elemento-Organic Chemistry, Frontiers Science Center for New Organic Matter, College of Chemistry, Nankai University, 94 Weijin Road, Tianjin, 300071, P. R. China.

*Corresponding authors: zruan@gzhmu.edu.cn; xinweihu@gzhmu.edu.cn; qiuyouai@nankai.edu.cn.

[†]These authors contributed equally to this work

Table of Contents

1 Supplementary Notes 3
1.1 General remarks 3
1.2 The Electrochemical Reaction Setup 4
2 Supplementary Methods 5
2.1 General Procedure for the Preparation of Starting Materials 5
2.2 Optimization Studies 7
2.3 General Procedure for Electrochemical Reactions 9
3 Supplementary Discussion 10
3.1 Characterization Data of Starting Materials 10
3.2 Characterization Data of Products 29
3.3 Gram-Scale Synthesis and Diversifications of Peptide Aldehyde 56
3.4 Removal of Piv- Protecting Group 80
3.5 Preliminary Mechanistic Study 81
3.6 Limitation 88
3.7 Theoretical Calculation of Reduction Potentials 88
3.8 Antioxidant Activity Assay 98
3.9 Studies on a Potential Racemization of Peptides 99
3.10 X-Ray Crystallographic Data of **11b** 104
3.11 NMR Spectra 107
4 Supplementary References 247

1 Supplementary Notes

1.1 General remarks

Electrochemical reactions were conducted using an AXIOMET AX-3003P potentiostat in constant current mode using undivided cell equipped with graphite felt plate (1.5 cm × 1.0 cm × 0.2 cm) as the anode and platinum plate (1.5 cm × 1.0 cm × 0.01 cm) as the cathode under atmosphere. Graphite felt plates are commercially available from Bei Jing Jinglong Special Carbon Technology Co. Ltd. Platinum electrodes are commercially available from Tian Jin Aida (China). Other chemicals were obtained from commercial sources and were used without further purification. Yields refer to isolated compounds, estimated to be >95% pure as determined by ^{1}H-NMR. TLC: Macherey-Nagel, TLC plates Alugram®Sil G/UV254. Detection under UV light at 254 nm. Chromatography separations were carried out on 300-400 mesh silica gel manufactured by Qingdao Haiyang Chemical Group Co. (China). High resolution mass spectrometry (HRMS) was measured on Thermo-DFS mass spectrometer (Q Exactive Focus). NMR spectra were recorded on *J*EOL 400 NMR (^{1}H 400 MHz; ^{13}C 100 MHz; ^{19}F 376 MHz) in $CDCl_3$. If not otherwise specified, chemical shifts (δ) are given in ppm.

1.2 The Electrochemical Reaction Setup

(a)

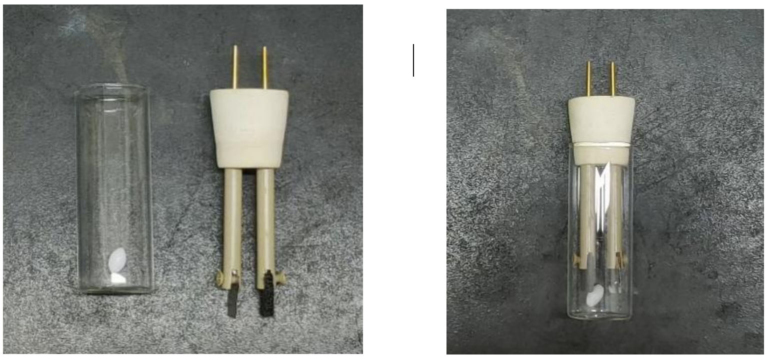

(b)

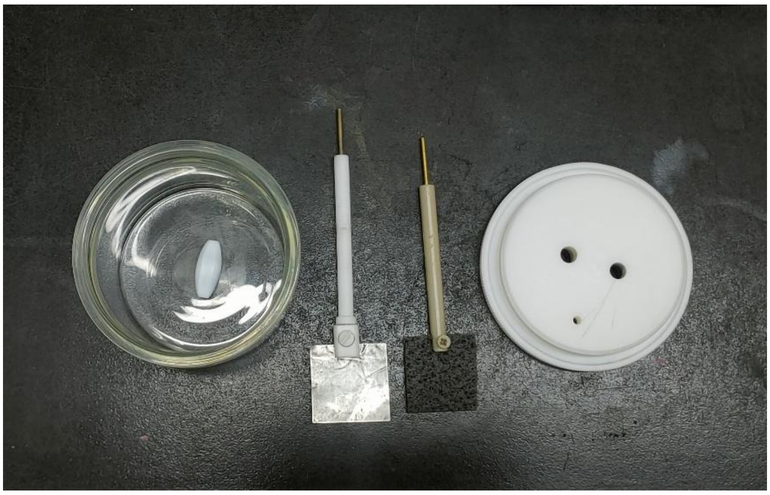

Supplementary Figure 1. The setup of electrochemical reaction. (a) Equipment of standard reaction; (b) Equipment of gram scale reaction.

2 Supplementary Methods

2.1 General Procedure for the Preparation of Starting Materials

2.1.1 Preparation of Peptides 1a-35a[1-4]

Representative Procedure for Methyl Ester Hydrolysis

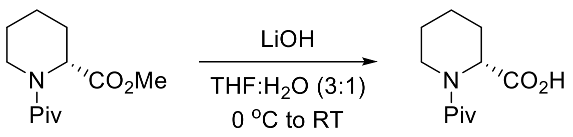

66a **70a**

A 100 mL round-bottom flask was charged with a solution of **66a** (20.0 mmol) in THF: H_2O (3:1, 40 mL) and cooled to 0 °C. LiOH (5.0 equiv., 100.0 mmol) was added and the resulting mixture was warmed to room temperature. After 12 h, the reaction mixture was cooled to 0 °C and acidified with 1M HCl aq. to pH < 2. The solution was then diluted with EtOAc (50 mL) and the aqueous layer was extracted with EtOAc (50 mL × 3). The combined organic layers were washed with brine (50 mL), dried over Na_2SO_4, filtered and concentrated under reduced pressure to afford **70a**, which was used in the next step without further purification.

Representative Procedure for Condensation Reaction

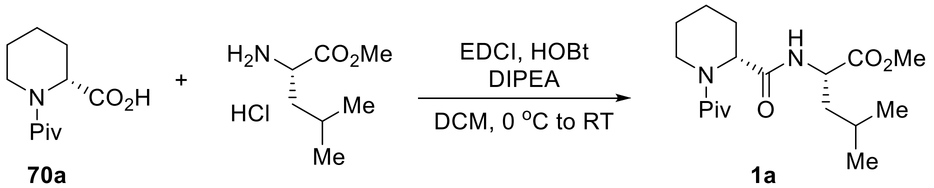

70a **1a**

A 100 mL round-bottomed flask was charged with a solution of **70a** (5.0 mmol), *L*-leucine methyl ester hydrochloride (1.2 equiv., 6.0 mmol) and 1-Hydroxybenzotriazole (HOBt: 1.2 equiv., 6.0 mmol) in DCM (30 mL) and cooled to 0 °C. *i*Pr_2NEt (3.0 equiv., 15.0 mmol) was added dropwise over 5 min and the resulting mixture was stirred at 0 °C for 10 min. To this solution was added *N*-(3-dimethylaminopropyl)-*N'*-ethylcarbodiimide hydrochloride (EDCI: 1.5 equiv., 7.5 mmol) and the resulting mixture was warmed to room temperature. After 12 h, the reaction mixture was cooled to 0 °C and quenched with 1M HCl aq. (50 mL). The phases were separated and the aqueous phase was extracted with DCM (50 mL × 3). The combined organic layers

were washed with brine (50 mL), dried over Na_2SO_4, filtered and concentrated under reduced pressure. The crude residue was purified by column chromatography to afford **1a** (80%) as a white solid.

2.1.2 Preparation of Esters 36a-44a[5-8]

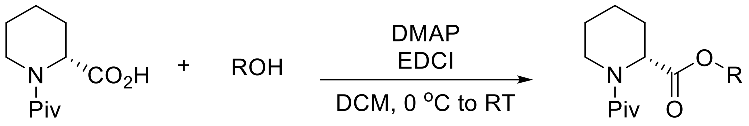

70a **36a-44a**

A 50 mL round-bottomed flask was charged with a solution of **70a** (2.0 mmol), alcohol (1.2 equiv., 2.4 mmol) and 4-Dimethylaminopyridine (DMAP: 20 mol %, 0.4 mmol) in DCM (20 mL) and cooled to 0 °C. To this solution was added EDCI (1.5 equiv., 3.0 mmol) and the resulting mixture was warmed to room temperature. After 12 h, the reaction mixture was cooled to 0 °C and quenched with 1M HCl aq. (20 mL). The phases were separated and the aqueous phase was extracted with DCM (20 mL × 3). The combined organic layers were washed with brine (20 mL), dried over Na_2SO_4, filtered and concentrated under reduced pressure. The crude residue was purified by column chromatography to afford **36a-44a**.

2.1.3 Preparation of Cyclic Amines 62a-76a

The substrates **62a**[2], **63a-64a**, **67a-68a**, **72a-73a, 75a-76a**[3], **65a**[9], **69a**[10], **71a**[11] and **74a**[12] were synthesized according to previously described methods as follows.

2.2 Optimization Studies

Supplementary Table 1. Optimization of Electrochemical Reaction Conditions[a]

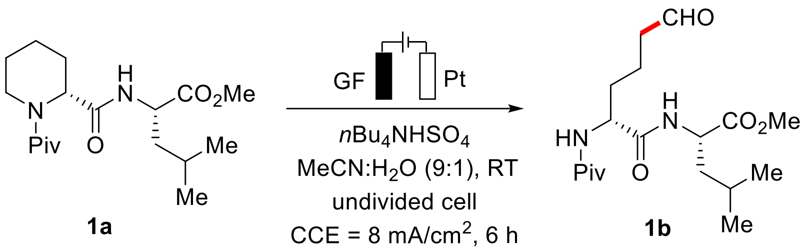

Entry	Deviation from standard conditions	Yield [%][b]
**1**	**none**	**73**
2	*n*Bu_4NPF_6 instead of *n*Bu_4NHSO_4	43
3	*n*Bu_4NOAc instead of *n*Bu_4NHSO_4	22
4	$LiClO_4$ instead of *n*Bu_4NHSO_4	0
5	*n*Bu_4NI instead of *n*Bu_4NHSO_4	0
6	$(NH_4)_2SO_4$ instead of *n*Bu_4NHSO_4	0
7	*n*Bu_4NOH instead of *n*Bu_4NHSO_4	29
8	no *n*Bu_4NHSO_4	0
9	MeCN/HFIP (9:1) instead of MeCN/H_2O (9:1)	10
10	MeCN instead of MeCN/H_2O (9:1)	22
11	C (graphite) as an anode	58
12	RVC (reticulated vitreous carbon) as an anode	25
13	GF (graphite felt) as an cathode	11
14	no electricity	0

[a] Reaction conditions: Undivided cell, graphite felt anode, Pt cathode, **1a** (0.3 mmol), *n*Bu_4NHSO_4 (0.3 mmol), MeCN/H_2O (9:1, 10 mL), constant current = 8.0 mA/cm^2, 6 h (6.0 Fmol^{-1}), 23 °C, under air. [b] Yields of isolated products.

Supplementary Table 2. Optimization of Electrochemical Reaction Conditions[a]

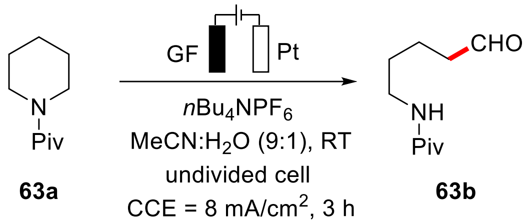

Entry	Deviation from standard conditions	Yield [%][b]
**1**	**none**	**88**
2	nBu$_4$NHSO$_4$ instead of nBu$_4$NPF$_6$	74
3	LiClO$_4$ instead of nBu$_4$NPF$_6$	10
4	nBu$_4$NOH instead of nBu$_4$NPF$_6$	72
5	no nBu$_4$NPF$_6$	0
6	MeCN/HFIP (9:1) instead of MeCN/H$_2$O (9:1)	42
7	MeCN instead of MeCN/H$_2$O (9:1)	13
8	C (graphite) as an anode	59
9	RVC (reticulated vitreous carbon) as an anode	17
10	no electricity	0

[a] Reaction conditions: Undivided cell, graphite felt anode, Pt cathode, **63a** (0.3 mmol), nBu$_4$NPF$_6$ (0.3 mmol), MeCN/H$_2$O (9:1, 10 mL), constant current = 8.0 mA/cm^2, 3 h (3.0 Fmol^{-1}), 23 °C, under air. [b] Yields of isolated products.

Supplementary Table 3. Optimization of Electrochemical Conditions for Different Protective Substrates

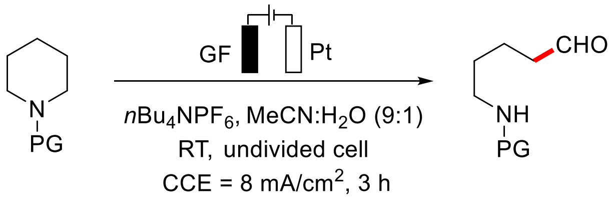

Amine	Product	Amine	Product	Amine	Product
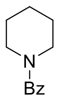	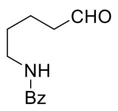 **62b**: 53%	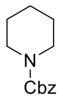	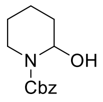 **78**: 45%	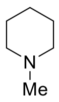	ND
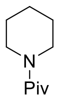	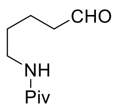 **63b**:88%	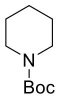	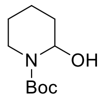 **79**: 30%	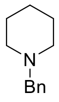	ND

2.3 General Procedure for Electrochemical Reactions

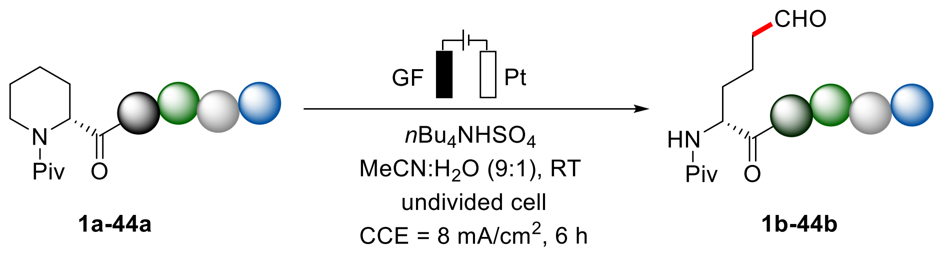

General Procedure A: In an undivided cell (30 mL) equipped with a stirring bar, a mixture of substrates **1a-44a** (0.3 mmol), *n*Bu_4NHSO_4 (0.3 mmol, 0.03M) and MeCN/H_2O (9:1, 10 mL) were added. The cell was equipped with graphite felt plate (1.5 cm × 1.0 cm × 0.2 cm) as the anode and platinum plate (1.5 cm × 1.0 cm × 0.01 cm) as the cathode connected to an AXIOMET AX-3003P DC regulated power supply. The reaction mixture was stirred and electrolyzed at a constant current of 8 mA at room temperature for 6 h. Upon completion, the solvent was removed directly under reduced pressure to afford the crude product, which was further purified by flash column chromatography to afford the desired products **1b-44b**.

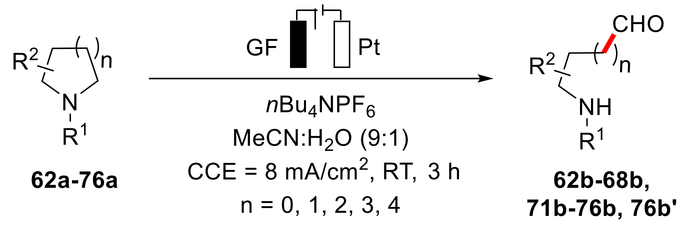

General Procedure B: In an undivided cell (30 mL) equipped with a stirring bar, a mixture of substrates **62a-76a** (0.3 mmol), *n*Bu_4NPF_6 (0.3 mmol, 0.03M) and MeCN/H_2O (9:1, 10 mL) were added. The cell was equipped with graphite felt plate (1.5 cm × 1.0 cm × 0.2 cm) as the anode and platinum plate (1.5 cm × 1.0 cm × 0.01 cm) as the cathode connected to an AXIOMET AX-3003P DC regulated power supply. The reaction mixture was stirred and electrolyzed at a constant current of 8 mA at room temperature for 3 h. Upon completion, the solvent was removed directly under reduced pressure to afford the crude product, which was further purified by flash column chromatography to afford the desired products **62b-68b, 71b-76b, 76b'**.

3 Supplementary Discussion

3.1 Characterization Data of Starting Materials

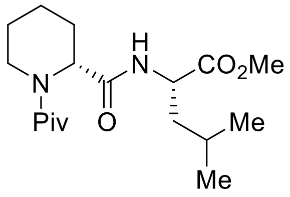

Methyl [(*R*)-6-oxo-2-pivalamidohexanoyl]-*L*-leucinate (**1a**) was prepared from **70a** and *L*-leucine methyl ester hydrochloride according to the representative procedure. Isolation by column chromatography yielded **1a** (80%) as a white solid.. ^{1}H NMR (400 MHz, $CDCl_3$) δ = 6.54 (d, J = 8.5 Hz, 1H), 5.17 (d, J = 5.3 Hz, 1H), 4.64 – 4.51 (m, 1H), 4.15 (d, J = 14.0 Hz, 1H), 3.68 (s, 3H), 3.01 (t, J = 12.6 Hz, 1H), 2.25 (d, J = 13.7 Hz, 1H), 1.79 – 1.52 (m, 5H), 1.55 – 1.37 (m, 3H), 1.31 (s, 9H), 0.90 (d, J = 6.1 Hz, 3H), 0.89 (d, J = 6.1 Hz, 3H). ^{13}C NMR (100 MHz, $CDCl_3$) δ = 178.8, 173.5, 171.3, 53.6, 52.3, 50.4, 44.7, 41.3, 39.0, 28.4, 25.4, 25.3, 24.9, 23.0, 21.8, 20.7. HR-MS(ESI) *m/z* calcd for: $C_{18}H_{32}N_2O_4Na^+$ $[M+Na]^+$ 363.2254, found 363.2252.

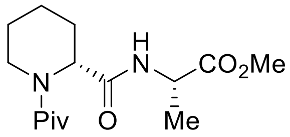

Methyl [(*R*)-1-pivaloylpiperidine-2-carbonyl]-*L*-alaninate (**2a**) was prepared from **70a** and *L*-alanine methyl ester hydrochloride according to the representative procedure. Isolation by column chromatography yielded **2a** (67%) as a colorless liquid. ^{1}H NMR (400 MHz, $CDCl_3$) δ = 6.64 (d, J = 6.7 Hz, 1H), 5.21 (d, J = 5.6 Hz, 1H), 4.63 – 4.51 (m, 1H), 4.17 (d, J = 13.9 Hz, 1H), 3.71 (s, 3H), 3.03 (t, J = 13.3 Hz, 1H), 2.28 (d, J = 13.6 Hz, 1H), 1.72 – 1.65 (m, 3H), 1.54 – 1.41 (m, 2H), 1.37 (d, J = 7.2 Hz, 3H), 1.33 (s, 9H). ^{13}C NMR (100 MHz, $CDCl_3$) δ = 178.7, 173.5, 170.9, 53.6, 52.5, 47.7, 44.8, 39.0, 28.3, 25.4, 25.2, 20.7, 18.2. HR-MS(ESI) *m/z* calcd for: $C_{15}H_{26}N_2O_4Na^+$ $[M+Na]^+$ 321.1785, found 321.1781.

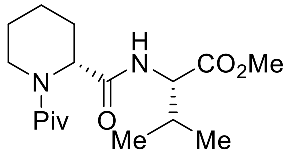

Methyl [(*R*)-1-pivaloylpiperidine-2-carbonyl]-*L*-valinate (**3a**) was prepared from **70a** and *L*-valine methyl ester hydrochloride according to the representative procedure. Isolation by column chromatography yielded **3a** (65%) as a white solid. ^{1}H NMR (400 MHz, $CDCl_3$) δ = 6.68 (d, J = 8.7 Hz, 1H), 5.22 (d, J = 5.5 Hz, 1H), 4.48 (dd, J = 8.8, 4.4 Hz, 1H), 4.17 (d, J = 13.9 Hz, 1H), 3.70 (s, 3H), 3.03 (t, J = 13.4 Hz, 1H), 2.28 (d, J = 13.7 Hz, 1H), 2.23 – 2.11 (m, 1H), 1.73 – 1.60 (m, 3H), 1.56 – 1.38 (m, 2H), 1.33 (s, 9H), 0.93 (d, J = 6.8 Hz, 3H), 0.84 (d, J = 6.8 Hz, 3H). ^{13}C NMR (100 MHz, $CDCl_3$) δ = 178.9, 172.5, 171.4, 57.0, 53.7, 52.1, 44.8, 39.1, 30.8, 28.4, 25.5, 25.3, 20.7, 19.2, 17.5. HR-MS(ESI) *m/z* calcd for: $C_{17}H_{30}N_2O_4Na^+$ $[M+Na]^+$ 349.2098, found 349.2099.

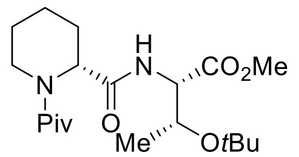

Methyl *O*-(*tert*-butyl)-*N*-[(*R*)-1-pivaloylpiperidine-2-carbonyl]-*L*-threoninate (**4a**) was prepared from **70a** and *O*-*tert*-butyl-*L*-threonine methyl ester hydrochloride according to the representative procedure. Isolation by column chromatography yielded **4a** (27%) as a colorless liquid. ^{1}H NMR (400 MHz, $CDCl_3$) δ = 6.77 (d, J = 9.0 Hz, 1H), 5.35 (s, 1H), 4.43 (d, J = 9.0 Hz, 1H), 4.28 – 4.15 (m, 2H), 3.70 (s, 3H), 3.26 (t, J = 13.4 Hz, 1H), 2.37 (d, J = 13.4 Hz, 1H), 1.71 – 1.63 (m, 3H), 1.57 – 1.39 (m, 2H), 1.34 (s, 9H), 1.16 (d, J = 6.1 Hz, 3H), 1.07 (s, 9H). ^{13}C NMR (100 MHz, $CDCl_3$) δ = 178.1, 171.8, 171.6, 74.1, 67.2, 58.0, 54.0, 52.2, 44.7, 39.1, 28.5, 28.4, 25.7, 25.5, 21.3, 21.0. HR-MS(ESI) *m/z* calcd for: $C_{20}H_{36}N_2O_5Na^+$ $[M+Na]^+$ 407.2516, found 407.2517.

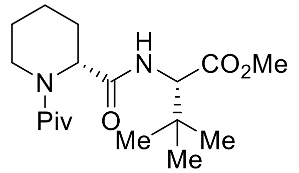

Methyl (*S*)-3,3-dimethyl-2-[(*R*)-1-pivaloylpiperidine-2-carboxamido]butanoate (**5a**) was prepared from **70a** and *L*-*tert*-leucine *(after methyl esterification)* according to the representative procedure. Isolation by column chromatography yielded **5a** (58%) as a colorless liquid. ^{1}H NMR (400 MHz, $CDCl_3$) δ = 6.77 (d, J = 8.6 Hz, 1H), 5.21 (d, J = 5.5 Hz, 1H), 4.28 (d, J = 8.8 Hz, 1H), 4.14 (d, J = 14.2 Hz, 1H), 3.68 (s, 3H), 2.94 (t, J = 13.2 Hz, 1H), 2.27 (d, J = 13.6 Hz, 1H), 1.73 – 1.58 (m, 3H), 1.54 – 1.39 (m,

2H), 1.32 (s, 9H), 0.93 (s, 9H). ^{13}C NMR (100 MHz, $CDCl_3$) δ = 178.9, 172.2, 171.1, 60.5, 53.6, 51.8, 44.9, 39.0, 34.3, 28.4, 26.7, 25.4, 25.2, 20.7. HR-MS(ESI) *m/z* calcd for: $C_{18}H_{32}N_2O_4Na^+$ $[M+Na]^+$ 363.2254, found 363.2250.

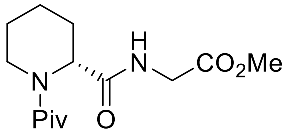

Methyl (*R*)-(1-pivaloylpiperidine-2-carbonyl)glycinate (**6a**) was prepared from **70a** and glycine methyl ester hydrochloride according to the representative procedure. Isolation by column chromatography yielded **6a** (92%) as a white solid. ^{1}H NMR (400 MHz, $CDCl_3$) δ = 6.67 (s, 1H), 5.19 (s, 1H), 4.29 – 4.10 (m, 2H), 3.79 (dd, J = 18.3, 4.3 Hz, 1H), 3.71 (s, 3H), 3.04 (t, J = 13.5 Hz, 1H), 2.28 (d, J = 13.5 Hz, 1H), 1.78 – 1.60 (m, 3H), 1.55 – 1.40 (m, 2H), 1.31 (s, 9H). ^{13}C NMR (100 MHz, $CDCl_3$) δ = 178.8, 171.7, 170.4, 53.7, 52.4, 44.8, 41.0, 39.0, 28.4, 25.4, 25.3, 20.7. HR-MS(ESI) *m/z* calcd for: $C_{14}H_{24}N_2O_4Na^+$ $[M+Na]^+$ 307.1628, found 307.1626.

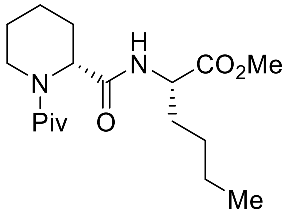

Methyl (*S*)-2-[(*R*)-1-pivaloylpiperidine-2-carboxamido]hexanoate (**7a**) was prepared from **70a** and *L*-norleucine methyl ester hydrochloride according to the representative procedure. Isolation by column chromatography yielded **7a** (60%) as a colorless liquid. ^{1}H NMR (400 MHz, $CDCl_3$) δ = 6.60 (d, J = 8.0 Hz, 1H), 5.17 (d, J = 5.3 Hz, 1H), 4.56 – 4.47 (m, 1H), 4.14 (d, J = 14.1 Hz, 1H), 3.67 (s, 3H), 3.01 (t, J = 13.4 Hz, 1H), 2.25 (d, J = 13.6 Hz, 1H), 1.83 – 1.54 (m, 5H), 1.53 – 1.36 (m, 2H), 1.30 (s, 9H), 1.28 – 1.12 (m, 4H), 0.84 (t, J = 6.7 Hz, 3H). ^{13}C NMR (100 MHz, $CDCl_3$) δ = 178.8, 173.0, 171.2, 53.7, 52.3, 51.9, 44.7, 39.0, 31.9, 28.3, 27.5, 25.4, 25.2, 22.3, 20.7, 13.9. HR-MS(ESI) *m/z* calcd for: $C_{18}H_{32}N_2O_4Na^+$ $[M+Na]^+$ 363.2254, found 363.2251.

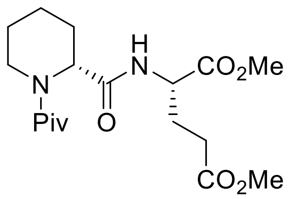

Dimethyl [(*R*)-1-pivaloylpiperidine-2-carbonyl]-*L*-glutamate (**8a**) was prepared from **70a** and *L*-glutamic acid dimethyl ester hydrochloride according to the representative procedure. Isolation by column chromatography yielded **8a** (65%) as a colorless liquid. ^{1}H NMR (400 MHz, $CDCl_3$) δ = 6.78 (d, J = 8.3 Hz, 1H), 5.21 (d, J = 4.3 Hz, 1H), 4.65 – 4.54 (m, 1H), 4.16 (d, J = 14.0 Hz, 1H), 3.71 (s, 3H), 3.66 (s, 3H), 3.02 (t, J = 12.9 Hz, 1H), 2.43 – 2.16 (m, 4H), 2.00 – 1.88 (m, 1H), 1.73 – 1.60 (m, 3H), 1.53 – 1.40 (m, 2H), 1.33 (s, 9H). ^{13}C NMR (100 MHz, $CDCl_3$) δ = 178.7, 173.1, 172.3, 171.4, 53.7, 52.5, 51.9, 51.4, 44.7, 39.0, 30.1, 28.3, 27.1, 25.4, 25.2, 20.6. HR-MS(ESI) *m/z* calcd for: $C_{18}H_{30}N_2O_6Na^+$ $[M+Na]^+$ 393.1996, found 393.1994.

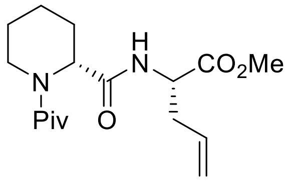

Methyl (*S*)-2-[(*R*)-1-pivaloylpiperidine-2-carboxamido]pent-4-enoate (**9a**) was prepared from **70a** and *L*-allylglycine *(after methyl esterification)* according to the representative procedure. Isolation by column chromatography yielded **9a** (52%) as a colorless liquid. ^{1}H NMR (400 MHz, $CDCl_3$) δ = 6.61 (d, J = 7.8 Hz, 1H), 5.70 – 5.53 (m, 1H), 5.20 (d, J = 5.5 Hz, 1H), 5.16 – 5.05 (m, 2H), 4.62 (dd, J = 8.1, 5.5 Hz, 1H), 4.15 (d, J = 13.8 Hz, 1H), 3.71 (s, 3H), 3.05 (t, J = 13.2 Hz, 1H), 2.50 (t, J = 6.4 Hz, 2H), 2.27 (d, J = 13.6 Hz, 1H), 1.73 – 1.58 (m, 3H), 1.54 – 1.36 (m, 2H), 1.30 (s, 9H). ^{13}C NMR (100 MHz, $CDCl_3$) δ = 178.6, 172.2, 171.1, 132.0, 119.7, 53.8, 52.4, 51.3, 44.7, 39.0, 36.2, 28.4, 25.5, 25.3, 20.7. HR-MS(ESI) *m/z* calcd for: $C_{17}H_{28}N_2O_4Na^+$ $[M+Na]^+$ 347.1941, found 347.1940.

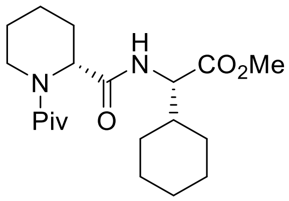

Methyl (*S*)-2-cyclohexyl-2-[(*R*)-1-pivaloylpiperidine-2-carboxamido]acetate (**10a**) was prepared from **70a** and *L*-cyclohexylglycine methyl ester hydrochloride according to the representative procedure. Isolation by column chromatography yielded **10a** (63%) as a colorless liquid. ^{1}H NMR (400 MHz, $CDCl_3$) δ = 6.65 (d, J = 8.6 Hz, 1H), 5.18 (d, J = 5.5 Hz, 1H), 4.44 (dd, J = 8.9, 4.6 Hz, 1H), 4.13 (d, J = 13.7 Hz, 1H), 3.66 (s, 3H), 2.98 (t, J = 13.2 Hz, 1H), 2.25 (d, J = 13.5 Hz, 1H), 1.82 – 1.54 (m, 8H), 1.51 – 1.43 (m, 2H), 1.30 (s, 9H), 1.26 – 0.90 (m, 6H). ^{13}C NMR (100 MHz, $CDCl_3$) δ = 178.8, 172.4, 171.2, 56.7, 53.7, 52.1, 44.7, 40.6, 39.0, 29.7, 28.4, 28.0, 26.0, 25.9, 25.4, 25.2, 20.7. HR-MS(ESI) *m/z* calcd for: $C_{20}H_{34}N_2O_4Na^+$ $[M+Na]^+$ 389.2411, found 389.2409.

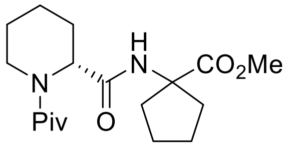

Methyl (*R*)-1-(1-pivaloylpiperidine-2-carboxamido)cyclopentane-1-carboxylate (**11a**) was prepared from **70a** and cycloeucine *(after methyl esterification)* according to the representative procedure. Isolation by column chromatography yielded **11a** (94%) as a white solid. ^{1}H NMR (400 MHz, $CDCl_3$) δ = 6.70 (s, 1H), 5.09 (d, J = 5.6 Hz, 1H), 4.15 (d, J = 13.7 Hz, 1H), 3.69 (s, 3H), 3.02 (t, J = 13.3 Hz, 1H), 2.29 – 2.08 (m, 3H), 2.00 – 1.90 (m, 1H), 1.87 – 1.61 (m, 8H), 1.52 – 1.39 (m, 2H), 1.31 (s, 9H). ^{13}C NMR (100 MHz, $CDCl_3$) δ = 178.8, 174.6, 171.2, 65.7, 53.7, 52.5, 44.7, 39.1, 37.9, 37.1, 28.4, 25.5, 25.3, 24.7, 24.6, 20.6. HR-MS(ESI) *m/z* calcd for: $C_{18}H_{30}N_2O_4Na^+$ $[M+Na]^+$ 361.2098, found 361.2094.

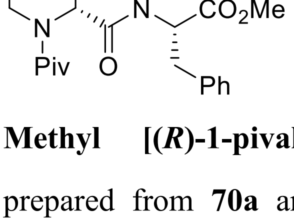

Methyl [(*R*)-1-pivaloylpiperidine-2-carbonyl]-*L*-phenylalaninate (**12a**) was prepared from **70a** and methyl *L*-phenylalaninate hydrochloride according to the representative procedure. Isolation by column chromatography yielded **12a** (87%) as a

white solid. ^{1}H NMR (400 MHz, $CDCl_3$) δ = 7.34 – 7.19 (m, 3H), 7.13 – 7.08 (m, 2H), 6.52 (d, J = 8.7 Hz, 1H), 5.23 (d, J = 6.5 Hz, 1H), 4.94 – 4.86 (m, 1H), 4.14 – 4.03 (m, 1H), 3.71 (s, 3H), 3.16 – 2.92 (m, 3H), 2.33 – 2.24 (m, 1H), 1.69 – 1.57 (m, 3H), 1.52 – 1.33 (m, 2H), 1.20 (s, 9H). ^{13}C NMR (100 MHz, $CDCl_3$) δ = 178.3, 172.0, 170.9, 135.6, 129.3, 128.8, 127.2, 52.6, 52.3, 44.7, 38.8, 37.9, 28.2, 25.4, 25.2, 20.7. HR-MS(ESI) *m/z* calcd for: $C_{21}H_{30}N_2O_4Na^+$ $[M+Na]^+$ 397.2098, found 397.2103.

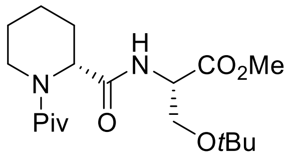

Methyl *O*-(tert-butyl)-*N*-[(*R*)-1-pivaloylpiperidine-2-carbonyl]-*L*-serinate (**13a**) was prepared from **70a** and methyl *O*-(tert-butyl)-*L*-serinate hydrochloride according to the representative procedure. Isolation by column chromatography yielded **13a** (76%) as a white solid. ^{1}H NMR (400 MHz, $CDCl_3$) δ = 6.81 (d, J = 8.7 Hz, 1H), 5.28 (t, J = 3.0 Hz, 1H), 4.65 (dt, J = 8.6, 2.7 Hz, 1H), 4.18 (d, J = 13.8 Hz, 1H), 3.81 (dd, J = 8.8, 2.5 Hz, 1H), 3.70 (s, 3H), 3.49 (dd, J = 9.1, 3.1 Hz, 1H), 3.22 (t, J = 13.3 Hz, 1H), 2.39 – 2.28 (m, 1H), 1.72 – 1.60 (m, 3H), 1.53 – 1.37 (m, 2H), 1.31 (s, 9H), 1.09 (s, 9H). ^{13}C NMR (100 MHz, $CDCl_3$) δ = 178.2, 171.2, 171.1, 73.5, 61.7, 53.8, 52.6, 52.3, 44.7, 39.0, 28.4, 27.3, 25.7, 25.3, 20.9. HR-MS(ESI) *m/z* calcd for: $C_{19}H_{34}N_2O_5Na^+$ $[M+Na]^+$ 393.2360, found 393.2357.

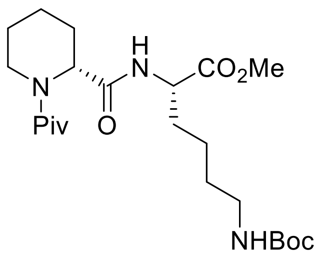

Methyl N^6-(tert-butoxycarbonyl)-N^2-[(*R*)-1-pivaloylpiperidine-2-carbonyl]-*L*-lysinate (**14a**) was prepared from **70a** and methyl N^6-(tert-butoxycarbonyl)-*L*-lysinate hydrochloride according to the representative procedure. Isolation by column chromatography yielded **14a** (56%) as a colorless liquid. ^{1}H NMR (400 MHz, $CDCl_3$) δ = 6.66 (d, J = 8.3 Hz, 1H), 5.20 (d, J = 5.5 Hz, 1H), 4.66 (t, J = 5.0 Hz, 1H), 4.60 – 4.52 (m, 1H), 4.16 (d, J = 13.9 Hz, 1H), 3.70 (s, 3H), 3.14 – 2.94 (m, 3H), 2.27 (d, J =

13.5 Hz, 1H), 1.83 – 1.77 (m, 1H), 1.76 – 1.57 (m, 4H), 1.54 – 1.44 (m, 4H), 1.42 (s, 9H), 1.32 (s, 9H), 1.30 – 1.23 (m, 2H). ^{13}C NMR (100 MHz, $CDCl_3$) δ = 178.9, 172.9, 171.3, 156.1, 79.1, 53.6, 52.4, 51.7, 44.8, 40.3, 39.1, 32.0, 29.4, 28.5, 28.4, 25.4, 25.2, 22.6, 20.7. HR-MS(ESI) *m/z* calcd for: $C_{23}H_{41}N_3O_6Na^+$ $[M+Na]^+$ 478.2888, found 478.2881.

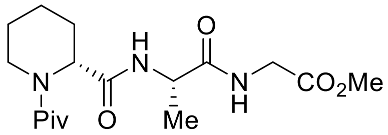

Methyl [(*R*)-1-pivaloylpiperidine-2-carbonyl]-*L*-alanylglycinate (15a) was prepared from **2a** and glycine methyl ester hydrochloride according to the representative procedure. Isolation by column chromatography yielded **15a** (85%) as a colorless liquid. ^{1}H NMR (400 MHz, $CDCl_3$) δ = 7.00 (t, J = 5.5 Hz, 1H), 6.68 (d, J = 7.7 Hz, 1H), 5.10 (d, J = 4.8 Hz, 1H), 4.53 (p, J = 7.2 Hz, 1H), 4.13 (d, J = 14.6 Hz, 1H), 4.08 – 3.90 (m, 2H), 3.71 (s, 3H), 3.11 (t, J = 13.1 Hz, 1H), 2.24 (d, J = 13.3 Hz, 1H), 1.71 – 1.59 (m, 3H), 1.60 – 1.39 (m, 2H), 1.36 (d, J = 6.9 Hz, 3H), 1.29 (s, 9H). ^{13}C NMR (100 MHz, $CDCl_3$) δ = 178.9, 172.7, 171.6, 170.2, 54.4, 52.4, 48.6, 44.7, 41.2, 39.0, 28.3, 25.5, 25.3, 20.5, 18.1. HR-MS(ESI) *m/z* calcd for: $C_{17}H_{29}N_3O_5Na^+$ $[M+Na]^+$ 378.1999, found 378.1995.

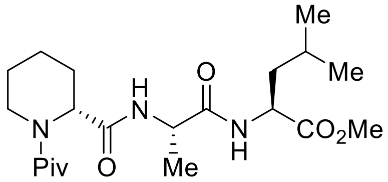

Methyl [(*R*)-1-pivaloylpiperidine-2-carbonyl]-*L*-alanyl-*L*-leucinate (16a) was prepared from **2a** and *L*-leucine methyl ester hydrochloride according to the representative procedure. Isolation by column chromatography yielded **16a** (85%) as a white solid. ^{1}H NMR (400 MHz, $CDCl_3$) δ = 6.61 (t, J = 8.5 Hz, 2H), 5.14 (d, J = 5.2 Hz, 1H), 4.60 – 4.53 (m, 1H), 4.47 (p, J = 7.2 Hz, 1H), 4.15 (d, J = 14.8 Hz, 1H), 3.70 (s, 3H), 3.00 (t, J = 13.0 Hz, 1H), 2.27 (d, J = 13.3 Hz, 1H), 1.69 – 1.57 (m, 5H), 1.58 – 1.41 (m, 3H), 1.33 (d, J = 6.9 Hz, 3H), 1.30 (s, 9H), 0.91 (d, J = 4.6 Hz, 3H), 0.89 (d, J = 4.6 Hz, 3H). ^{13}C NMR (100 MHz, $CDCl_3$) δ = 178.8, 173.3, 172.1, 171.4, 54.2, 52.4, 50.8, 48.7, 44.7, 41.4, 39.1, 28.4, 25.5, 25.4, 24.9, 22.9, 21.9, 20.6, 18.0. HR-

MS(ESI) *m/z* calcd for: $C_{21}H_{37}N_3O_5Na^+$ $[M+Na]^+$ 434.2626, found 434.2621.

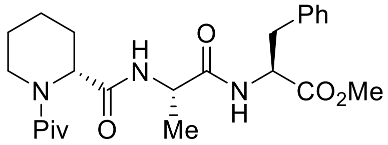

Methyl [(*R*)-1-pivaloylpiperidine-2-carbonyl]-*L*-alanyl-*L*-phenylalaninate (17a) was prepared from **2a** and methyl *L*-phenylalaninate hydrochloride according to the representative procedure. Isolation by column chromatography yielded **17a** (70%) as a white solid. ^{1}H NMR (400 MHz, $CDCl_3$) δ = 7.32 – 7.20 (m, 3H), 7.15 – 7.06 (m, 2H), 6.57 (d, J = 7.3 Hz, 1H), 6.50 (d, J = 7.8 Hz, 1H), 5.15 (d, J = 3.9 Hz, 1H), 4.87 – 4.79 (m, 1H), 4.49 – 4.39 (m, 1H), 4.12 (d, J = 13.9 Hz, 1H), 3.12 (qd, J = 13.9, 5.9 Hz, 2H), 2.96 (t, J = 13.2 Hz, 1H), 2.34 – 2.23 (m, 1H), 1.71 – 1.59 (m, 3H), 1.57 – 1.37 (m, 2H), 1.33 (d, J = 7.0 Hz, 3H), 1.29 (s, 9H). ^{13}C NMR (100 MHz, $CDCl_3$) δ = 178.8, 171.9, 171.8, 171.3, 135.8, 129.3, 128.7, 127.2, 54.1, 53.2, 52.5, 48.8, 44.8, 39.0, 37.8, 28.3, 25.4, 20.7, 18.5. HR-MS(ESI) *m/z* calcd for: $C_{24}H_{35}N_3O_5Na^+$ $[M+Na]^+$ 468.2469, found 468.2474.

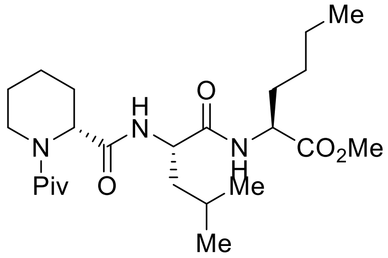

Methyl (*S*)-2-{(*S*)-4-methyl-2-[(*R*)-1-pivaloylpiperidine-2-carboxamido]pentanamido}hexanoate (18a) was prepared from **1a** and *L*-norleucine methyl ester hydrochloride according to the representative procedure. Isolation by column chromatography yielded **18a** (73%) as a colorless liquid. ^{1}H NMR (400 MHz, $CDCl_3$) δ = 6.48 (d, J = 8.5 Hz, 2H), 5.15 (d, J = 5.3 Hz, 1H), 4.58 – 4.49 (m, 1H), 4.46 – 4.37 (m, 1H), 4.16 (d, J = 13.7 Hz, 1H), 3.72 (s, 3H), 3.00 (t, J = 12.5 Hz, 1H), 2.27 (d, J = 13.4 Hz, 1H), 1.84 – 1.77 (m, 2H), 1.72 – 1.58 (m, 6H), 1.56 – 1.40 (m, 3H), 1.31 (s, 9H), 1.29 – 1.22 (m, 3H), 0.92 (d, J = 5.8 Hz, 3H), 0.91 (d, J = 5.8 Hz, 3H), 0.87 (t, J = 6.9 Hz, 3H). ^{13}C NMR (100 MHz, $CDCl_3$) δ = 178.9, 172.9, 171.8, 171.7, 54.1, 52.4, 52.2, 51.8, 44.8, 41.1, 39.1, 32.2, 28.4, 27.4, 25.5, 25.4, 24.9, 23.0, 22.4, 22.1, 20.7,

13.9. HR-MS(ESI) *m/z* calcd for: $C_{24}H_{43}N_3O_5Na^+$ $[M+Na]^+$ 476.3095, found 476.3093.

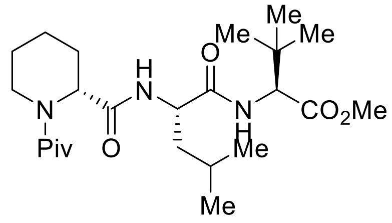

Methyl (*S*)-3,3-dimethyl-2-{(*S*)-4-methyl-2-[(*R*)-1-pivaloylpiperidine-2-carboxamido]pentanamido}butanoate (**19a**) was prepared from **1a** and *L*-*tert*-leucine *(after methyl esterification)* according to the representative procedure. Isolation by column chromatography yielded **19a** (50%) as a white solid. ^{1}H NMR (400 MHz, $CDCl_3$) δ = 6.62 (d, J = 9.1 Hz, 1H), 6.51 (d, J = 7.7 Hz, 1H), 5.14 (d, J = 5.3 Hz, 1H), 4.42 – 4.34 (m, 2H), 4.13 (d, J = 13.2 Hz, 1H), 3.69 (s, 3H), 2.97 (t, J = 12.5 Hz, 1H), 2.26 (d, J = 13.4 Hz, 1H), 1.70 – 1.49 (m, 5H), 1.54 – 1.36 (m, 3H), 1.28 (s, 9H), 0.93 (s, 9H), 0.90 (d, J = 5.8 Hz, 3H), 0.88 (d, J = 5.8 Hz, 3H). ^{13}C NMR (100 MHz, $CDCl_3$) δ = 178.8, 171.82, 171.76, 171.7, 60.0, 54.0, 52.0, 51.9, 44.7, 40.8, 39.1, 35.0, 28.4, 26.6, 25.5, 25.4, 24.8, 23.0, 22.0, 20.6. HR-MS(ESI) *m/z* calcd for: $C_{24}H_{43}N_3O_5Na^+$ $[M+Na]^+$ 476.3095, found 476.3091.

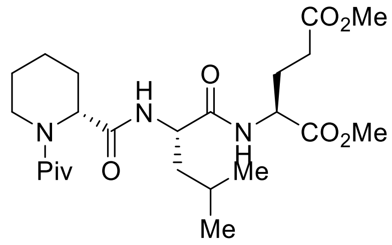

Dimethyl [(*R*)-1-pivaloylpiperidine-2-carbonyl]-*L*-leucyl-*L*-glutamate (**20a**) was prepared from **1a** and *L*-glutamic acid dimethyl ester hydrochloride according to the representative procedure. Isolation by column chromatography yielded **20a** (64%) as a colorless liquid. ^{1}H NMR (400 MHz, $CDCl_3$) δ = 6.68 (d, J = 7.9 Hz, 1H), 6.49 (d, J = 7.8 Hz, 1H), 5.13 (d, J = 5.4 Hz, 1H), 4.64 – 4.52 (m, 1H), 4.44 – 4.32 (m, 1H), 4.16 (d, J = 14.0 Hz, 1H), 3.73 (s, 3H), 3.66 (s, 3H), 3.03 (t, J = 13.3 Hz, 1H), 2.47 – 2.30 (m, 2H), 2.30 – 2.14 (m, 2H), 2.04 – 1.88 (m, 1H), 1.74 – 1.56 (m, 5H), 1.56 – 1.41 (m, 3H), 1.31 (s, 9H), 0.92 (d, J = 5.8Hz, 3H), 0.91 (d, J = 5.8 Hz, 3H). ^{13}C NMR (100 MHz, $CDCl_3$) δ = 178.9, 173.3, 172.1, 171.8, 54.1, 52.7, 52.0, 51.8, 51.6, 44.8, 41.1, 39.1, 30.0, 28.4, 27.4, 25.5, 25.4, 24.9, 23.0, 22.1, 20.6. HR-MS(ESI) *m/z* calcd for:

$C_{24}H_{41}N_3O_7Na^+$ $[M+Na]^+$ 506.2837, found 506.2840.

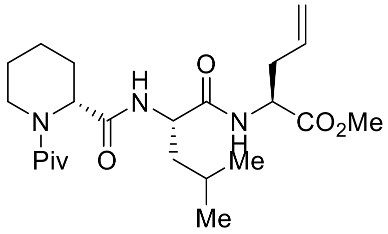

Methyl (*S*)-2-{(*S*)-4-methyl-2-[(*R*)-1-pivaloylpiperidine-2-carboxamido]pentanamido}pent-4-enoate (**21a**) was prepared from **1a** and *L*-allylglycine *(after methyl esterification)* according to the representative procedure. Isolation by column chromatography yielded **21a** (62%) as a white solid. ^{1}H NMR (400 MHz, $CDCl_3$) δ = 6.59 (d, J = 7.9 Hz, 1H), 6.48 (d, J = 8.0 Hz, 1H), 5.70 – 5.56 (m, 1H), 5.15 – 5.02 (m, 3H),4.62 – 4.53 (m, 1H), 4.45 – 4.35 (m, 1H), 4.13 (d, J = 13.8 Hz, 1H), 3.69 (s, 3H), 3.01 (t, J = 12.9 Hz, 1H), 2.59 – 2.40 (m, 2H), 2.24 (d, J = 13.4 Hz, 1H), 1.70 – 1.47 (m, 5H), 1.70 – 1.47 (m, 3H), 1.28 (s, 9H), 0.89 (d, J = 6.0 Hz, 3H), 0.87 (d, J = 6.0 Hz, 3H). ^{13}C NMR (100 MHz, $CDCl_3$) δ = 178.8, 171.9, 171.8, 171.6, 132.2, 119.2, 54.1, 52.4, 51.8, 51.7, 44.7, 41.1, 39.0, 36.3, 28.3, 25.5, 25.3, 24.8, 23.0, 22.0, 20.6. HR-MS(ESI) *m/z* calcd for: $C_{23}H_{39}N_3O_5Na^+$ $[M+Na]^+$ 460.2782, found 460.2777.

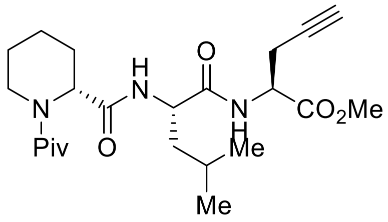

Methyl (*S*)-2-{(*S*)-4-methyl-2-[(*R*)-1-pivaloylpiperidine-2-carboxamido]pentanamido}pent-4-ynoate (**22a**) was prepared from **1a** and *L*-propargylglycine *(after methyl esterification)* according to the representative procedure. Isolation by column chromatography yielded **22a** (82%) as a white solid. ^{1}H NMR (400 MHz, $CDCl_3$) δ = 6.77 (d, J = 7.7 Hz, 1H), 6.52 (d, J = 7.9 Hz, 1H), 5.15 (d, J = 5.4 Hz, 1H), 4.71 – 4.62 (m, 1H), 4.52 – 4.41 (m, 1H), 4.16 (d, J = 14.0 Hz, 1H), 3.76 (s, 3H), 3.04 (t, J = 13.4 Hz, 1H), 2.80 – 2.67 (m, 2H), 2.27 (d, J = 13.7 Hz, 1H), 2.00 (s, 1H), 1.75 – 1.59 (m, 5H), 1.56 – 1.40 (m, 3H), 1.30 (s, 9H), 0.92 (d, J = 5.3 Hz, 3H), 0.91 (d, J = 5.3 Hz, 3H). ^{13}C NMR (100 MHz, $CDCl_3$) δ = 178.9, 171.9, 171.7, 170.6, 78.4, 71.8, 54.1, 52.9, 51.7, 50.8, 44.8, 41.1, 39.1, 28.4, 25.42, 25.39, 24.9, 23.1, 22.3, 22.0, 20.6. HR-MS(ESI)

m/z calcd for: $C_{23}H_{37}N_3O_5Na^+$ $[M+Na]^+$ 458.2626, found 458.2627.

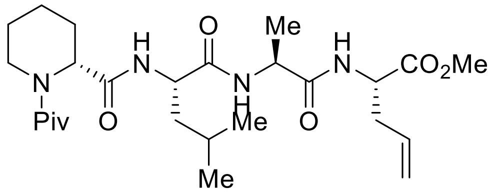

Methyl (*S*)-2-{(*S*)-2-{(*S*)-4-methyl-2-[(*R*)-1-pivaloylpiperidine-2-carboxamido] pentanamido}propanamido}pent-4-enoate (**23a**) was prepared from **1a** and methyl (*S*)-2-[(*S*)-2-aminopropanamido]pent-4-enoate hydrochloride according to the representative procedure. Isolation by column chromatography yielded **23a** (79%) as a white solid. ^{1}H NMR (400 MHz, $CDCl_3$) δ = 6.81 (d, *J* = 7.5 Hz, 1H), 6.72 (d, *J* = 7.8 Hz, 1H), 6.47 (d, *J* = 7.9 Hz, 1H), 5.72 – 5.58 (m, 1H), 5.15 – 5.05 (m, 2H), 4.99 (dd, *J* = 5.8, 3.1 Hz, 1H), 4.62 – 4.53 (m, 1H), 4.52 – 4.34 (m, 2H), 4.12 (d, *J* = 13.5 Hz, 1H), 3.72 (s, 3H), 3.11 (t, *J* = 13.0 Hz, 1H), 2.61 – 2.41 (m, 2H), 2.27 – 2.14 (m, 1H), 1.74 – 1.41 (m, 8H), 1.35 (d, *J* = 7.2 Hz, 3H), 1.30 (s, 9H), 0.91 (d, *J* = 5.8 Hz, 3H), 0.90 (d, *J* = 5.8 Hz, 3H). ^{13}C NMR (100 MHz, $CDCl_3$) δ = 179.2, 172.1, 172.1, 171.9, 171.7, 132.2, 119.4, 54.6, 52.5, 51.9, 51.7, 48.9, 44.7, 40.9, 39.1, 36.4, 28.4, 25.6, 25.1, 24.9, 23.1, 21.8, 20.4, 17.9. HR-MS(ESI) *m/z* calcd for: $C_{26}H_{44}N_4O_6Na^+$ $[M+Na]^+$ 531.3153, found 531.3155.

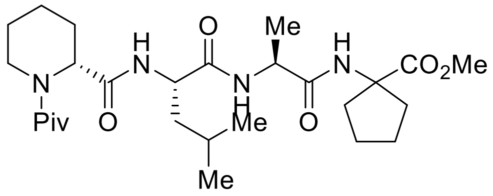

Methyl 1-{(*S*)-2-{(*S*)-4-methyl-2-[(*R*)-1-pivaloylpiperidine-2-carboxamido]pentanamido}propanamido}cyclopentane-1-carboxylate (**24a**) was prepared from **1a** and methyl (*S*)-1-(2-aminopropanamido)cyclopentane-1-carboxylate hydrochloride according to the representative procedure. Isolation by column chromatography yielded **24a** (61%) as a white solid. ^{1}H NMR (400 MHz, $CDCl_3$) δ = 6.95 – 6.84 (m, 2H), 6.45 (d, *J* = 7.2 Hz, 1H), 4.82 – 4.75 (m, 1H), 4.44 – 4.25 (m, 2H), 4.13 – 4.02 (m, 1H), 3.66 (s, 3H), 3.26 (t, *J* = 13.0 Hz, 1H), 2.29 – 2.07 (m, 3H), 1.99 – 1.87 (m, 2H), 1.78 – 1.57 (m, 10H), 1.56 – 1.44 (m, 2H), 1.35 (d, *J* = 7.2 Hz, 3H), 1.29 (s, 9H), 0.92 (d, *J* = 6.3 Hz, 3H), 0.90 (d, *J* = 6.3 Hz, 3H). ^{13}C NMR (100 MHz, $CDCl_3$) δ = 179.7, 174.6, 172.9,

172.0, 171.8, 65.9, 55.4, 52.4, 52.3, 49.0, 44.5, 40.7, 39.0, 37.4, 37.2, 28.3, 25.6, 25.1, 24.72, 24.65, 24.6, 23.2, 21.7, 20.0, 17.4. HR-MS(ESI) *m/z* calcd for: $C_{27}H_{46}N_4O_6Na^+$ $[M+Na]^+$ 545.3310, found 545.3308.

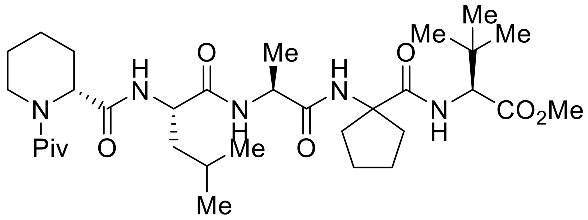

Methyl (*S*)-3,3-dimethyl-2-{1-{(*S*)-2-{(*S*)-4-methyl-2-[(*R*)-1-pivaloylpiperidine-2-carboxamido]pentanamido}propanamido}cyclopentane-1-carboxamido}butanoate (**25a**) was prepared from **24a** and *L-tert*-leucine *(after methyl esterification)* according to the representative procedure. Isolation by column chromatography yielded **25a** (57%) as a white solid. ^{1}H NMR (400 MHz, $CDCl_3$) δ = 7.71 (d, J = 8.9 Hz, 1H), 7.07 (d, J = 6.8 Hz, 1H), 6.82 (s, 1H), 6.39 (d, J = 6.8 Hz, 1H), 4.61 – 4.54 (m, 1H), 4.41 – 4.29 (m, 2H), 4.29 – 4.21 (m, 1H), 3.99 (d, J = 13.9 Hz, 1H), 3.66 (s, 3H), 3.39 (t, J = 12.5 Hz, 1H), 2.34 – 2.26 (m, 1H), 2.20 – 2.13 (m, 2H), 1.99 – 1.90 (m, 1H), 1.76 – 1.65 (m, 9H), 1.65 – 1.44 (m, 4H), 1.41 (d, J = 7.2 Hz, 3H), 1.25 (s, 9H), 0.96 (s, 9H), 0.93 (d, J = 6.3 Hz, 3H), 0.91 (d, J = 6.3 Hz, 3H). ^{13}C NMR (100 MHz, $CDCl_3$) δ = 180.1, 173.6, 173.4, 173.2, 172.2, 171.9, 68.0, 60.7, 56.2, 52.6, 51.6, 49.9, 44.7, 40.4, 39.0, 36.6, 36.3, 34.9, 28.3, 26.7, 25.6, 25.2, 24.5, 24.0, 23.7, 23.2, 21.6, 19.9, 17.4. HR-MS(ESI) *m/z* calcd for: $C_{33}H_{57}N_5O_7Na^+$ $[M+Na]^+$ 658.4150, found 658.4147.

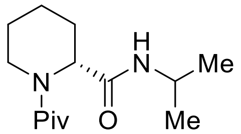

(*R*)-*N*-Isopropyl-1-pivaloylpiperidine-2-carboxamide (**26a**) was prepared from **70a** and isopropylamine according to the representative procedure. Isolation by column chromatography yielded **26a** (88%) as a white solid. ^{1}H NMR (400 MHz, $CDCl_3$) δ = 5.99 (d, J = 8.1 Hz, 1H), 5.06 (d, J = 5.5 Hz, 1H), 4.15 (d, J = 13.5 Hz, 1H), 4.09 – 3.98 (m, 1H), 2.90 (t, J = 12.2 Hz, 1H), 2.27 (d, J = 13.6 Hz, 1H), 1.87 – 1.74 (m, 1H), 1.69 – 1.61 (m, 2H), 1.53 – 1.39 (m, 2H), 1.29 (s, 9H), 1.10 (dd, J = 10.9, 6.7 Hz, 6H). ^{13}C NMR (100 MHz, $CDCl_3$) δ =178.5, 170.2, 54.1, 44.4, 41.2, 39.0, 28.3, 25.51, 25.46,

23.0, 22.7, 20.7. HR-MS(ESI) *m/z* calcd for: $C_{14}H_{26}N_2O_2Na^+$ $[M+Na]^+$ 277.1887, found 277.1886.

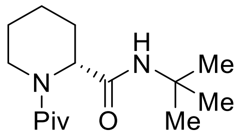

(*R*)-*N*-(*tert*-Butyl)-1-pivaloylpiperidine-2-carboxamide (**27a**) was prepared from **70a** and *tert*-butylamine according to the representative procedure. Isolation by column chromatography yielded **27a** (72%) as a white solid. ^{1}H NMR (400 MHz, $CDCl_3$) δ = 6.03 (br, 1H), 5.03 (d, J = 5.5 Hz, 1H), 4.13 (d, J = 13.6 Hz, 1H), 2.91 (t, J = 13.0 Hz, 1H), 2.25 (d, J = 13.4 Hz, 1H), 1.82 – 1.73 (m, 1H), 1.68 – 1.59 (m, 2H), 1.51 – 1.35 (m, 2H), 1.29 (d, J = 2.3 Hz, 18H). ^{13}C NMR (100 MHz, $CDCl_3$) δ = 178.3, 170.4, 54.4, 51.0, 44.6, 39.0, 28.9, 28.3, 25.6, 25.3, 20.7. HR-MS(ESI) *m/z* calcd for: $C_{15}H_{28}N_2O_2Na^+$ $[M+Na]^+$ 291.2043, found 291.2042.

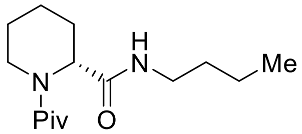

(*R*)-*N*-Butyl-1-pivaloylpiperidine-2-carboxamide (**28a**) was prepared from **70a** and butan-1-amine according to the representative procedure. Isolation by column chromatography yielded **28a** (50%) as a colorless liquid. ^{1}H NMR (400 MHz, $CDCl_3$) δ = 6.21 (br, 1H), 5.07 (br, 1H), 4.14 (d, J = 13.5 Hz, 1H), 3.37 – 3.23 (m, 1H), 3.19 – 3.05 (m, 1H), 2.92 (t, J = 13.5 Hz, 1H), 2.26 (d, J = 13.5 Hz, 1H), 1.84 – 1.71 (m, 1H), 1.70 – 1.60 (m, 2H), 1.52 – 1.36 (m, 4H), 1.32 – 1.24 (m, 11H), 0.88 (t, J = 7.3 Hz, 3H). ^{13}C NMR (100 MHz, $CDCl_3$) δ = 178.6, 171.2, 54.0, 44.5, 39.03, 38.99, 31.8, 28.4, 25.52, 25.48, 20.7, 20.1, 13.8. HR-MS(ESI) *m/z* calcd for: $C_{15}H_{28}N_2O_2Na^+$ $[M+Na]^+$ 291.2043, found 291.2038.

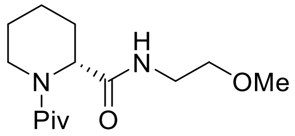

(*R*)-*N*-(2-Methoxyethyl)-1-pivaloylpiperidine-2-carboxamide (**29a**) was prepared from **70a** and 2-methoxyethan-1-amine according to the representative procedure. Isolation by column chromatography yielded **29a** (60%) as a yellow liquid. ^{1}H NMR

(400 MHz, $CDCl_3$) δ = 6.42 (br, 1H), 5.13 (br, 1H), 4.15 (d, J = 13.6 Hz, 1H), 3.55 – 3.42 (m, 1H), 3.43 – 3.28 (m, 3H), 3.29 (s, 3H), 2.92 (t, J = 13.3 Hz, 1H), 2.29 (d, J = 13.5 Hz, 1H), 2.02 – 1.96 (m, 1H), 1.69 – 1.63 (m, 2H), 1.54 – 1.35 (m, 2H), 1.28 (s, 9H). ^{13}C NMR (100 MHz, $CDCl_3$) δ = 178.3, 171.1, 71.2, 58.8, 54.2, 44.5, 39.0, 28.3, 25.53, 25.48, 20.8. HR-MS(ESI) *m/z* calcd for: $C_{14}H_{26}N_2O_3Na^+$ $[M+Na]^+$ 293.1836, found 293.1835.

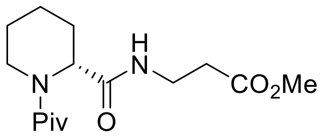

Methyl (*R*)-3-(1-pivaloylpiperidine-2-carboxamido)propanoate (**30a**) was prepared from **70a** and methyl 3-aminopropanoate according to the representative procedure. Isolation by column chromatography yielded **30a** (90%) as a colorless liquid. ^{1}H NMR (400 MHz, $CDCl_3$) δ = 6.62 (t, J = 5.3 Hz, 1H), 5.10 (d, J = 4.3 Hz, 1H), 4.13 (d, J = 13.6 Hz, 1H), 3.65 (s, 3H), 3.63 – 3.50 (m, 1H), 3.49 – 3.36 (m, 1H), 2.91 (t, J = 13.0 Hz, 1H), 2.50 (t, J = 6.1 Hz, 2H), 2.32 – 2.24 (m, 1H), 1.72 – 1.58 (m, 3H), 1.54 – 1.35 (m, 2H), 1.29 (s, 9H). ^{13}C NMR (100 MHz, $CDCl_3$) δ = 178.4, 172.8, 171.1, 54.1, 51.9, 44.6, 39.0, 34.9, 34.1, 28.3, 25.53, 25.49, 20.8. HR-MS(ESI) *m/z* calcd for: $C_{15}H_{26}N_2O_4Na^+$ $[M+Na]^+$ 321.1785, found 321.1783.

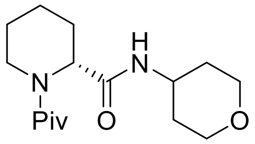

(*R*)-1-Pivaloyl-*N*-(tetrahydro-2H-pyran-4-yl)piperidine-2-carboxamide (**31a**) was prepared from **70a** and tetrahydro-2*H*-pyran-4-amine according to the representative procedure. Isolation by column chromatography yielded **31a** (83%) as a white solid. ^{1}H NMR (400 MHz, $CDCl_3$) δ = 6.22 (br, 1H), 5.05 (br, 1H), 4.11 (d, J = 13.7 Hz, 1H), 4.00 – 3.80 (m, 3H), 3.43 (t, J = 11.5 Hz, 2H), 2.90 (t, J = 13.2 Hz, 1H), 2.21 (d, J = 13.6 Hz, 1H), 1.87 – 1.68 (m, 3H), 1.69 – 1.58 (m, 2H), 1.52 – 1.33 (m, 4H), 1.27 (s, 9H). ^{13}C NMR (100 MHz, $CDCl_3$) δ = 178.7, 170.6, 66.7, 66.6, 53.8, 45.2, 44.6, 38.9, 33.2, 32.9, 28.3, 25.4, 25.3, 20.6. HR-MS(ESI) *m/z* calcd for: $C_{16}H_{28}N_2O_3Na^+$ $[M+Na]^+$ 319.1992, found 319.1993.

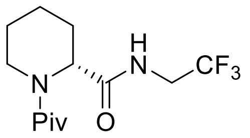

(*R*)-1-Pivaloyl-*N*-(2,2,2-trifluoroethyl)piperidine-2-carboxamide (**32a**) was prepared from **70a** and 2,2,2-trifluoroethan-1-amine according to the representative procedure. Isolation by column chromatography yielded **32a** (85%) as a white solid. ^{1}H NMR (400 MHz, $CDCl_3$) δ = 6.83 (br, 1H), 5.12 (d, J = 5.9 Hz, 1H), 4.29 – 4.16 (m, 1H), 4.12 (d, J = 13.8 Hz, 1H), 3.58 – 3.42 (m, 1H), 2.90 (t, J = 13.3 Hz, 1H), 2.21 (d, J = 13.9 Hz, 1H), 1.93 – 1.77 (m, 1H), 1.73 – 1.61 (m, 2H), 1.57 – 1.43 (m, 2H), 1.28 (s, 9H). ^{13}C NMR (100 MHz, $CDCl_3$) δ = 179.6, 172.1, 124.3(q, $^{1}J_{C\text{-}F}$ = 278.5 Hz), 53.4, 44.9, 40.3 (q, $^{2}J_{C\text{-}F}$ = 34.5 Hz), 39.0, 28.2, 25.3, 25.1, 20.4. ^{19}F NMR (376 MHz, $CDCl_3$) δ = -72.77 (t, J = 7.8 Hz). HR-MS(ESI) *m/z* calcd for: $C_{13}H_{21}F_3N_2O_2Na^+$ $[M+Na]^+$ 317.1447, found 317.1448.

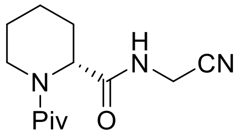

(*R*)-*N*-(Cyanomethyl)-1-pivaloylpiperidine-2-carboxamide (**33a**) was prepared from **70a** and 2-aminoacetonitrile according to the representative procedure. Isolation by column chromatography yielded **33a** (60%) as a colorless liquid. ^{1}H NMR (400 MHz, $CDCl_3$) δ = 7.01 (br, 1H), 5.07 (d, J = 5.6 Hz, 1H), 4.28 (dd, J = 17.5, 6.5 Hz, 1H), 4.14 (d, J = 13.9 Hz, 1H), 3.99 (dd, J = 17.4, 5.3 Hz, 1H), 2.94 (t, J = 13.3 Hz, 1H), 2.22 (d, J = 13.9 Hz, 1H), 1.60 – 1.41 (m, 1H), 1.60 – 1.41 (m, 2H), 1.60 – 1.41 (m, 2H), 1.31 (s, 9H). ^{13}C NMR (100 MHz, $CDCl_3$) δ = 179.4, 172.0, 116.3, 53.6, 44.8, 39.0, 28.3, 27.4, 25.5, 25.1, 20.3. HR-MS(ESI) *m/z* calcd for: $C_{13}H_{21}N_3O_2Na^+$ $[M+Na]^+$ 274.1526, found 274.1522.

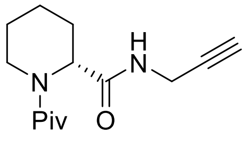

(*R*)-1-Pivaloyl-*N*-(prop-2-yn-1-yl)piperidine-2-carboxamide (**34a**) was prepared from **70a** and prop-2-yn-1-amine according to the representative procedure. Isolation by column chromatography yielded **34a** (90%) as a white solid. ^{1}H NMR (400 MHz,

$CDCl_3$) δ = 6.45 (br, 1H), 5.11 (d, J = 5.5 Hz, 1H), 4.20 – 4.05 (m, 2H), 3.97 – 3.85 (m, 1H), 2.95 (t, J = 13.4 Hz, 1H), 2.26 (d, J = 13.9 Hz, 1H), 2.17 (s, 1H), 1.88 – 1.61 (m, 3H), 1.54 – 1.42 (m, 2H), 1.30 (s, 9H). ^{13}C NMR (100 MHz, $CDCl_3$) δ = 178.8, 171.2, 79.7, 71.3, 53.8, 44.6, 39.0, 29.0, 28.3, 25.41, 25.36, 20.6. HR-MS(ESI) *m/z* calcd for: $C_{14}H_{22}N_2O_2Na^+$ $[M+Na]^+$ 273.1573, found 273.1570.

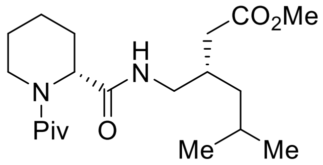

Methyl (*S*)-5-methyl-3-{[(*R*)-1-pivaloylpiperidine-2-carboxamido]methyl}hexanoate (**35a**) was prepared from **70a** and Pregabalin according to the representative procedure. Isolation by column chromatography yielded **35a** (80%) as a colorless liquid. ^{1}H NMR (400 MHz, $CDCl_3$) δ = 6.45 (br, 1H), 5.07 (br, 1H), 4.13 (d, J = 14.1 Hz, 1H), 3.64(s, 3H), 3.43 – 3.32 (m, 1H), 3.25 – 2.89 (m, 2H), 2.32 – 2.16 (m, 3H), 2.12 – 2.03 (m, 1H), 1.82 – 1.54 (m, 4H), 1.52 – 1.40 (m, 2H), 1.28 (s, 9H), 1.16 – 1.04 (m, 2H), 0.86 (d, J = 7.5 Hz, 3H), 0.84 (d, J = 7.5 Hz, 3H). ^{13}C NMR (100 MHz, $CDCl_3$) δ = 178.6, 173.3, 171.5, 54.0, 51.7, 44.7, 42.4, 41.6, 39.0, 37.2, 33.3, 28.4, 25.6, 25.5, 25.2, 22.8, 20.7. HR-MS(ESI) *m/z* calcd for: $C_{20}H_{36}N_2O_4Na^+$ $[M+Na]^+$ 391.2567, found 391.2566.

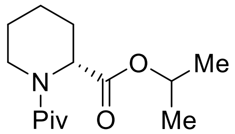

Isopropyl (*R*)-1-pivaloylpiperidine-2-carboxylate (**36a**) was prepared from **70a** and isopropyl alcohol according to the general procedure. Isolation by column chromatography yielded **36a** (78%) as a colorless liquid. ^{1}H NMR (400 MHz, $CDCl_3$) δ = 5.30 (br, 1H), 5.10 – 4.96 (m, 1H), 4.12 (br, 1H), 3.16 (br, 1H), 2.25 (d, J = 13.6 Hz, 1H), 1.73 – 1.54 (m, 3H), 1.51 – 1.28 (m, 2H), 1.28 (s, 9H), 1.22 (d, J = 5.4 Hz, 6H). ^{13}C NMR (100 MHz, $CDCl_3$) δ = 177.6, 171.2, 68.6, 53.5, 44.7, 38.8, 28.4, 26.9, 25.4, 21.95, 21.92, 21.3. HR-MS(ESI) *m/z* calcd for: $C_{14}H_{25}NO_3Na^+$ $[M+Na]^+$ 278.1727, found 278.1725.

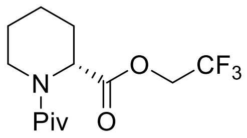

2,2,2-Trifluoroethyl (*R*)-1-pivaloylpiperidine-2-carboxylate (**37a**) was prepared from **70a** and 2,2,2-trifluoroethanol according to the general procedure. Isolation by column chromatography yielded **37a** (94%) as a colorless liquid. ^{1}H NMR (400 MHz, $CDCl_3$) δ = δ 5.44 (br, 1H), 4.57 – 4.44 (m, 2H), 4.15 (d, J = 13.6 Hz, 1H), 3.13 (t, J = 13.1 Hz, 1H), 2.26 (d, J = 13.6 Hz, 1H), 1.74 – 1.63 (m, 3H), 1.55 – 1.29 (m, 2H), 1.29 (s, 9H). ^{13}C NMR (100 MHz, $CDCl_3$) δ = 178.0, 170.5, 123.0 (q, $^1J_{C\text{-}F}$ = 277.3 Hz), 60.6 (q, $^2J_{C\text{-}F}$ = 36.6 Hz), 53.4, 44.8, 38.8, 28.2, 26.7, 25.2, 21.1. ^{19}F NMR (376 MHz, $CDCl_3$) δ = -73.62 (t, J = 9.3 Hz). HR-MS(ESI) *m/z* calcd for: $C_{13}H_{20}F_3NO_3Na^+$ $[M+Na]^+$ 318.1288, found 318.1287.

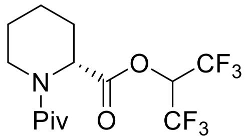

1,1,1,3,3,3-Hexafluoropropan-2-yl (*R*)-1-pivaloylpiperidine-2-carboxylate (**38a**) was prepared from **70a** and 1,1,1,3,3,3-hexafluoro-2-propanol according to the general procedure. Isolation by column chromatography yielded **38a** (64%) as a colorless liquid. ^{1}H NMR (400 MHz, $CDCl_3$) δ = 5.82 – 5.71 (m, 1H), 5.50 (br, 1H), 4.16 (d, J = 13.7 Hz, 1H), 3.13 (t, J = 13.2 Hz, 1H), 2.26 (d, J = 14.0 Hz, 1H), 1.81 – 1.64 (m, 3H), 1.56 – 1.41 (m, 1H), 1.40 – 1.31 (m, 1H), 1.28 (s, 9H). ^{13}C NMR (100 MHz, $CDCl_3$) δ = 178.2, 169.1, 120.4 (q, $^1J_{C\text{-}F}$ = 281.8 Hz), 66.9 (p, $^2J_{C\text{-}F}$ = 34.9 Hz), 53.4, 44.8, 38.8, 28.1, 26.6, 25.1, 21.0. ^{19}F NMR (376 MHz, $CDCl_3$) δ = -75.46 (s). HR-MS(ESI) *m/z* calcd for: $C_{14}H_{20}F_6NO_3$ $[M+H]^+$ 364.1342, found 364.1342.

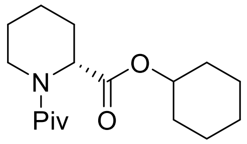

Cyclohexyl (*R*)-1-pivaloylpiperidine-2-carboxylate (**39a**) was prepared from **70a** and cyclohexanol according to the general procedure. Isolation by column chromatography yielded **39a** (62%) as a colorless liquid. ^{1}H NMR (400 MHz, $CDCl_3$) δ = 5.32 (br, 1H), 4.86 – 4.77 (m, 1H), 4.13 (br, 1H), 3.16 (br, 1H), 2.26 (d, J = 13.4 Hz, 1H), 1.86 – 1.77

(m, 2H), 1.75 – 1.56 (m, 5H), 1.54 – 1.31 (m, 8H), 1.28 (s, 9H). ^{13}C NMR (100 MHz, $CDCl_3$) δ = 177.6, 171.1, 73.4, 53.5, 44.8, 38.8, 31.7, 31.6, 28.4, 27.0, 25.4, 23.7, 23.6, 21.3. HR-MS(ESI) *m/z* calcd for: $C_{17}H_{30}NO_3$ $[M+H]^+$ 296.2220, found 296.2218.

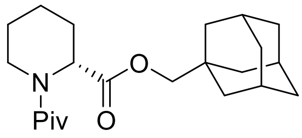

[(3*R*,5*R*,7*R*)-Adamantan-1-yl]methyl (*R*)-1-pivaloylpiperidine-2-carboxylate (**40a**) was prepared from **70a** and [(*3r,5r,7r*)-adamantan-1-yl]methanol according to the general procedure. Isolation by column chromatography yielded **40a** (58%) as a white solid. ^1H NMR (400 MHz, $CDCl_3$) δ = 5.38 (br, 1H), 4.15 (br, 1H), 3.79 – 3.62 (m, 2H), 3.17 (br, 1H), 2.28 (d, *J* = 13.5 Hz, 1H), 1.99 – 1.94 (m, 3H), 1.77 – 1.55 (m, 9H), 1.54 – 1.51 (m, 6H), 1.49 – 1.31 (m, 2H), 1.29 (s, 9H). ^{13}C NMR (100 MHz, $CDCl_3$) δ = 177.4, 171.9, 74.8, 53.5, 44.8, 39.4, 38.8, 37.0, 33.3, 28.5, 28.1, 27.0, 25.5, 21.4. HR-MS(ESI) *m/z* calcd for: $C_{22}H_{35}NO_3Na^+$ $[M+Na]^+$ 384.2509, found 384.2505.

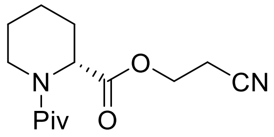

2-Cyanoethyl (*R*)-1-pivaloylpiperidine-2-carboxylate (**41a**) was prepared from **70a** and 3-hydroxypropanenitrile according to the general procedure. Isolation by column chromatography yielded **41a** (90%) as a white solid. ^1H NMR (400 MHz, $CDCl_3$) δ = 5.36 (br, 1H), 4.32 (t, *J* = 6.0 Hz, 2H), 4.15 (d, *J* = 14.0 Hz, 1H), 3.21 (t, *J* = 13.1 Hz, 1H), 2.72 (t, *J* = 5.8 Hz, 2H), 2.26 (d, *J* = 13.5 Hz, 1H), 1.76 – 1.61 (m, 3H), 1.54 – 1.32 (m, 2H), 1.29 (s, 9H). ^{13}C NMR (100 MHz, $CDCl_3$) δ = 178.0, 171.4, 116.8, 59.3, 53.5, 44.9, 38.8, 28.3, 26.7, 25.2, 21.2, 18.1. HR-MS(ESI) *m/z* calcd for: $C_{14}H_{22}N_2O_3Na^+$ $[M+Na]^+$ 289.1523, found 289.1521.

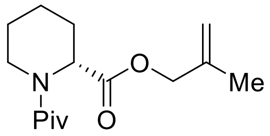

2-Methylallyl (*R*)-1-pivaloylpiperidine-2-carboxylate (**42a**) was prepared from **70a** and 2-methylprop-2-en-1-ol according to the general procedure. Isolation by column chromatography yielded **42a** (66%) as a colorless liquid. ^1H NMR (400 MHz, $CDCl_3$)

δ = 5.38 (br, 1H), 4.93 (d, J = 21.2 Hz, 2H), 4.52 (q, J = 13.1 Hz, 2H), 4.15 (br, 1H), 3.17 (br, 1H), 2.27 (d, J = 13.8 Hz, 1H), 1.73 (s, 3H), 1.71 – 1.59 (m, 3H), 2.34 – 2.21 (m, 2H), 1.28 (s, 9H). ^{13}C NMR (100 MHz, $CDCl_3$) δ = 177.7, 171.5, 139.7, 113.4, 68.4, 53.5, 44.8, 38.8, 28.4, 26.9, 25.4, 21.3, 19.6. HR-MS(ESI) *m/z* calcd for: $C_{15}H_{25}NO_3Na^+$ $[M+Na]^+$ 290.1727, found 290.1725.

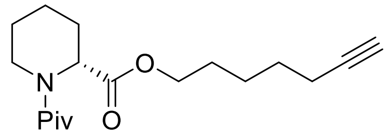

Hept-6-yn-1-yl (*R*)-1-pivaloylpiperidine-2-carboxylate (**43a**) was prepared from **70a** and hept-6-yn-1-ol according to the general procedure. Isolation by column chromatography yielded **43a** (63%) as a colorless liquid. ^{1}H NMR (400 MHz, $CDCl_3$) δ = 5.31 (br, 1H), 4.22 – 4.01 (m, 3H), 3.13 (br, 1H), 2.22 (d, J = 13.9 Hz, 1H), 2.15 (t, J = 5.3 Hz, 2H), 1.90 (s, 1H), 1.72 – 1.56 (m, 5H), 1.56 – 1.26 (m, 6H), 1.25 (s, 9H). ^{13}C NMR (100 MHz, $CDCl_3$) δ = 177.7, 171.8, 84.3, 68.6, 65.0, 53.4, 44.9, 38.8, 28.3, 28.2, 28.1, 26.9, 25.4, 25.1, 21.3, 18.4. HR-MS(ESI) *m/z* calcd for: $C_{18}H_{30}NO_3$ $[M+H]^+$ 308.2220, found 308.2214.

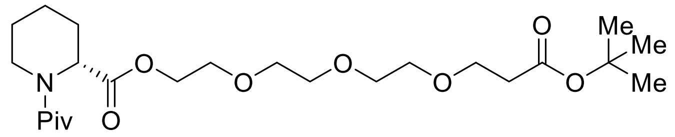

14,14-Dimethyl-12-oxo-3,6,9,13-tetraoxapentadecyl (*R*)-1-pivaloylpiperidine-2-carboxylate (**44a**) was prepared from **70a** and *tert*-butyl 3-{2-[2-(2-hydroxyethoxy) ethoxy]ethoxy}propanoate according to the general procedure. Isolation by column chromatography yielded **44a** (77%) as a colorless liquid. ^{1}H NMR (400 MHz, $CDCl_3$) δ = 5.35 (br, 1H), 4.36 – 4.17 (m, 2H), 4.12 (br, 1H), 3.73 – 3.62 (m, 4H), 3.63 – 3.55 (m, 8H), 3.18 (br, 1H), 2.48 (t, J = 6.6 Hz, 2H), 2.26 (d, J = 13.6 Hz, 1H), 1.73 – 1.55 (m, 3H), 1.48 – 1.35 (m, 11H), 1.28 (s, 9H). ^{13}C NMR (100 MHz, $CDCl_3$) δ = 177.7, 171.7, 171.0, 80.6, 70.7, 70.61, 70.56, 70.4, 69.1, 67.0, 64.0, 53.3, 44.8, 38.7, 36.3, 28.3, 28.2, 26.9, 25.3, 21.2. HR-MS(ESI) *m/z* calcd for: $C_{24}H_{43}NO_8Na^+$ $[M+Na]^+$ 496.2881, found 496.2879.

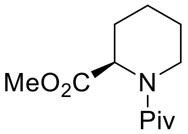

Methyl (*R*)-1-pivaloylpiperidine-2-carboxylate (66a) was prepared according to a reported procedure.[3] Isolation by column chromatography yielded **66a** (90%) as a colorless liquid. ^{1}H NMR (400 MHz, $CDCl_3$) δ = 5.32 (br, 1H), 4.12 (br, 1H), 3.69 (s, 3H), 3.14 (br, 1H), 2.22 (d, J = 14.1 Hz, 1H), 1.73 – 1.55 (m, 3H), 1.50 – 1.27 (m, 2H), 1.26 (s, 9H). ^{13}C NMR (100 MHz, $CDCl_3$) δ = 177.8, 172.2, 53.4, 52.2, 44.7, 38.7, 28.3, 26.8, 25.3, 21.2. HR-MS(ESI) *m/z* calcd for: $C_{12}H_{21}NO_3Na^+$ $[M+Na]^+$ 250.1414, found 250.1414.

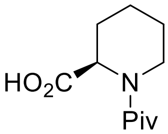

(*R*)-1-Pivaloylpiperidine-2-carboxylic acid (70a) was prepared according to a reported procedure.[2] Isolation by column chromatography yielded **70a** (83%) as a white solid. ^{1}H NMR (400 MHz, DMSO-d_6) δ = 5.04 (br, 1H), 4.08 (br, 1H), 3.07 (br, 1H), 2.11 (d, J = 13.3 Hz, 1H), 1.68 – 1.56 (m, 2H), 1.57 – 1.46 (m, 1H), 1.41 – 1.21 (m, 2H), 1.18 (s, 9H). ^{13}C NMR (100 MHz, DMSO-d_6) δ = 176.1, 172.8, 52.8, 44.0, 38.1, 28.1, 26.7, 25.0, 21.0. HR-MS(ESI) *m/z* calcd for: $C_{11}H_{19}NO_3Na^+$ $[M+Na]^+$ 236.1257, found 236.1255.

3.2 Characterization Data of Products

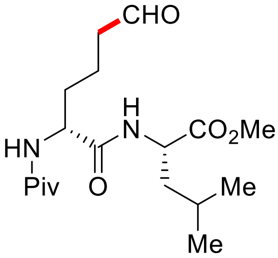

Methyl [(*R*)-6-oxo-2-pivalamidohexanoyl]-*L*-leucinate (1b): The general procedure **A** was followed using substrate **1a** (0.3 mmol, 102 mg). Isolation by column chromatography (PE/EA: 2/1) yielded **1b** (78 mg, 73%) as a white solid. M.p. = 110–111 °C. ^{1}H NMR (400 MHz, $CDCl_3$) δ = 9.75 (s, 1H), 7.01 (d, J = 8.1 Hz, 1H), 6.42 (d, J = 7.7 Hz, 1H), 4.59 – 4.51 (m, 1H), 4.51 – 4.43 (m, 1H), 3.70 (s, 3H), 2.57 – 2.46 (m,

2H), 1.93 – 1.82 (m, 1H), 1.73 – 1.52 (m, 6H), 1.21 (s, 9H), 0.93 (d, *J* = 4.9 Hz, 3H), 0.91 (d, *J* = 4.9 Hz, 3H). ^{13}C NMR (100 MHz, $CDCl_3$) *δ* = 202.2, 179.1, 173.2, 171.6, 52.42, 52.40, 50.9, 43.4, 41.3, 38.9, 31.5, 27.6, 25.0, 22.9, 21.8, 17.7. HR-MS(ESI) *m/z* calcd for $C_{18}H_{33}N_2O_5$ $[M+H]^+$ 357.2384, found 357.2379. $[\alpha]_D^{27}$ = 35.8° (*c* = 0.3, $CHCl_3$).

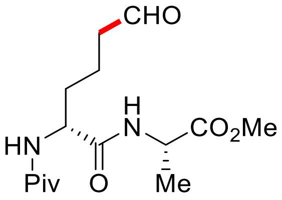

Methyl [(*R*)-6-oxo-2-pivalamidohexanoyl]-*L*-alaninate (2b): The general procedure **A** was followed using substrate **2a** (0.3 mmol, 89 mg). Isolation by column chromatography (PE/EA: 1/1) yielded **2b** (64 mg, 68%) as a colorless liquid. ^{1}H NMR (400 MHz, $CDCl_3$) *δ* = 9.75 (s, 1H), 7.04 (d, *J* = 7.4 Hz, 1H), 6.40 (d, *J* = 7.7 Hz, 1H), 4.57 – 4.49 (m, 1H), 4.49 – 4.40 (m, 1H), 3.72 (s, 3H), 2.58 – 2.47 (m, 2H), 1.94 – 1.79 (m, 1H), 1.73 – 1.55 (m, 3H), 1.40 (d, *J* = 7.3 Hz, 3H), 1.21 (s, 9H). ^{13}C NMR (100 MHz, $CDCl_3$) *δ* = 202.3, 179.1, 173.1, 171.2, 52.6, 52.4, 48.2, 43.4, 38.9, 31.5, 27.6, 18.2, 17.7. HR-MS(ESI) *m/z* calcd for $C_{15}H_{27}N_2O_5$ $[M+H]^+$ 315.1915, found 315.1908. $[\alpha]_D^{26}$ = 33.5° (*c* = 0.2, $CHCl_3$).

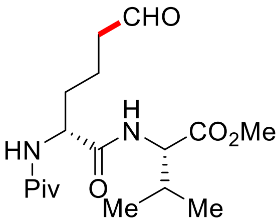

Methyl [(*R*)-6-oxo-2-pivalamidohexanoyl]-*L*-valinate (3b): The general procedure **A** was followed using substrate **3a** (0.3 mmol, 98 mg). Isolation by column chromatography (PE/EA: 2/1) yielded **3b** (66 mg, 64%) as a colorless liquid. ^{1}H NMR (400 MHz, $CDCl_3$) *δ* = 9.75 (s, 1H), 7.04 (d, *J* = 8.3 Hz, 1H), 6.43 (d, *J* = 7.7 Hz, 1H), 4.52 – 4.41 (m, 2H), 3.70 (s, 3H), 2.61 – 2.45 (m, 2H), 2.24 – 2.10 (m, 1H), 1.95 – 1.82 (m, 1H), 1.75 – 1.56 (m, 3H), 1.21 (s, 9H), 0.93 (d, *J* = 6.9 Hz, 3H), 0.89 (d, *J* = 6.9 Hz, 3H). ^{13}C NMR (100 MHz, $CDCl_3$) *δ* = 202.4, 179.1, 172.2, 171.8, 57.4, 52.5, 52.3, 43.4, 38.9, 31.6, 31.1, 27.5, 19.2, 17.8. HR-MS(ESI) *m/z* calcd for $C_{17}H_{31}N_2O_5$ $[M+H]^+$ 343.2228, found 343.2230. $[\alpha]_D^{26}$ = 35.0° (*c* = 0.2, $CHCl_3$).

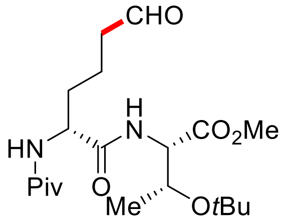

Methyl *O*-(*tert*-butyl)-*N*-[(*R*)-6-oxo-2-pivalamidohexanoyl]-*L*-threoninate (4b): The general procedure **A** was followed using substrate **4a** (0.3 mmol, 115 mg). Isolation by column chromatography (PE/EA: 2/1) yielded **4b** (61 mg, 51%) as a colorless liquid. ^{1}H NMR (400 MHz, $CDCl_3$) δ = 9.75 (s, 1H), 6.81 (d, J = 8.5 Hz, 1H), 6.44 (d, J = 5.6 Hz, 1H), 4.57 – 4.46 (m, 1H), 4.41 (d, J = 9.1 Hz, 1H), 4.26 – 4.15 (m, 1H), 3.68 (s, 3H), 2.62 – 2.43 (m, 2H), 1.98 – 1.84 (m, 1H), 1.76 – 1.58 (m, 3H), 1.23 (s, 9H), 1.14 (d, J = 6.1 Hz, 3H), 1.08 (s, 9H). ^{13}C NMR (100 MHz, $CDCl_3$) δ = 202.3, 178.8, 172.2, 171.2, 74.3, 67.3, 58.0, 52.8, 52.4, 43.4, 38.9, 31.7, 28.4, 27.6, 21.2, 17.9. HR-MS(ESI) *m/z* calcd for $C_{20}H_{37}N_2O_6$ $[M+H]^+$ 401.2646, found 401.2644. $[\alpha]_D^{27}$ = 32.5° (c = 0.2, $CHCl_3$).

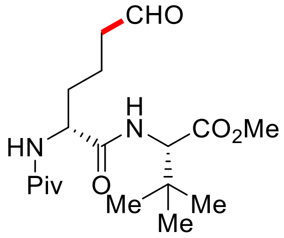

Methyl (*S*)-3,3-dimethyl-2-[(*R*)-6-oxo-2-pivalamidohexanamido]butanoate (5b): The general procedure **A** was followed using substrate **5a** (0.3 mmol, 102 mg). Isolation by column chromatography (PE/EA: 3/1) yielded **5b** (62 mg, 58%) as a white solid. M.p. = 127–128 °C. ^{1}H NMR (400 MHz, $CDCl_3$) δ = 9.76 (s, 1H), 7.05 (d, J = 7.7 Hz, 1H), 6.38 (d, J = 6.6 Hz, 1H), 4.51 – 4.40 (m, 1H), 4.34 (d, J = 9.0 Hz, 1H), 3.69 (s, 3H), 2.63 – 2.45 (m, 2H), 1.97 – 1.83 (m, 1H), 1.76 – 1.57 (m, 3H), 1.22 (s, 9H), 0.96 (s, 9H). ^{13}C NMR (100 MHz, $CDCl_3$) δ = 202.3, 179.2, 171.8, 171.6, 60.6, 52.5, 51.9, 43.4, 38.9, 34.6, 31.4, 27.6, 26.7, 17.9. HR-MS(ESI) *m/z* calcd for $C_{18}H_{33}N_2O_5$ $[M+H]^+$ 357.2384, found 357.2385. $[\alpha]_D^{27}$ = 62.7° (c = 0.05, $CHCl_3$).

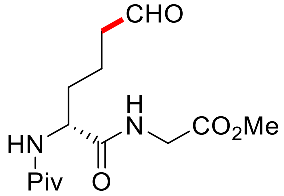

Methyl (*R*)-(6-oxo-2-pivalamidohexanoyl)glycinate (6b): The general procedure **A** was followed using substrate **6a** (0.3 mmol, 85 mg). Isolation by column

chromatography (PE/EA: 1/1) yielded **6b** (50 mg, 56%) as a white solid. M.p. = 75–76 °C. ^{1}H NMR (400 MHz, CDCl$_3$) δ = 9.72 (s, 1H), 7.51 (d, J = 6.2 Hz, 1H), 6.56 (d, J = 7.7 Hz, 1H), 4.60 – 4.49 (m, 1H), 3.98 (qd, J = 18.1, 5.5 Hz, 2H), 3.70 (s, 3H), 2.54 – 2.42 (m, 2H), 1.91 – 1.76 (m, 1H), 1.73 – 1.55 (m, 3H), 1.18 (s, 9H). ^{13}C NMR (100 MHz, CDCl$_3$) δ = 202.4, 179.0, 172.3, 170.1, 52.4, 52.3, 43.3, 41.1, 38.8, 31.9, 27.5, 17.7. HR-MS(ESI) *m/z* calcd for $C_{14}H_{25}N_2O_5$ [M+H]$^+$ 301.1758, found 301.1754.

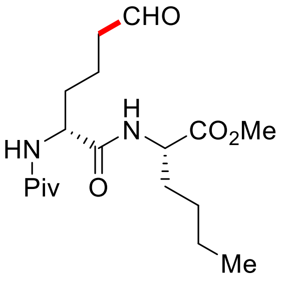

Methyl (*S*)-2-[(*R*)-6-oxo-2-pivalamidohexanamido]hexanoate (7b): The general procedure **A** was followed using substrate **7a** (0.3 mmol, 102 mg). Isolation by column chromatography (PE/EA: 2/1) yielded **7b** (66 mg, 62%) as a colorless liquid. ^{1}H NMR (400 MHz, CDCl$_3$) δ = 9.73 (s, 1H), 7.12 (d, J = 7.4 Hz, 1H), 6.45 (d, J = 6.4 Hz, 1H), 4.54 – 4.42 (m, 2H), 3.69 (s, 3H), 2.58 – 2.41 (m, 2H), 1.92 – 1.75 (m, 2H), 1.72 – 1.54 (m, 4H), 1.33 – 1.23 (m, 4H), 1.19 (s, 9H), 0.85 (t, J = 6.7 Hz, 3H). ^{13}C NMR (100 MHz, CDCl$_3$) δ = 202.2, 179.0, 172.8, 171.6, 52.4, 52.3, 43.4, 38.9, 31.9, 31.6, 27.5, 22.3, 17.8, 13.9. HR-MS(ESI) *m/z* calcd for $C_{18}H_{33}N_2O_5$ [M+H]$^+$ 357.2384, found 357.2383. $[\alpha]_D^{27}$ = 41.0° (c = 0.2, CHCl$_3$).

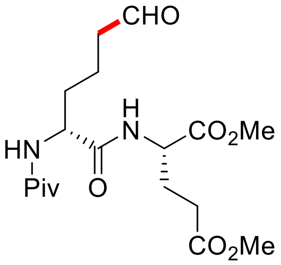

Dimethyl [(*R*)-6-oxo-2-pivalamidohexanoyl]-*L*-glutamate (8b): The general procedure **A** was followed using substrate **8a** (0.3 mmol, 116 mg). Isolation by column chromatography (PE/EA: 1/1) yielded **8b** (85 mg, 73%) as a colorless liquid. ^{1}H NMR (400 MHz, CDCl$_3$) δ = 9.75 (s, 1H), 7.25 (d, J = 8.7 Hz, 1H), 6.42 (d, J = 7.7 Hz, 1H), 4.61 – 4.50 (m, 1H), 4.50 – 4.41 (m, 1H), 3.71 (s, 3H), 3.66 (s, 3H), 2.60 – 2.47 (m, 2H), 2.46 – 2.31 (m, 2H), 2.26 – 2.14 (m, 1H), 2.05 – 1.94 (m, 1H), 1.93 – 1.82 (m, 1H), 1.72 – 1.55 (m, 3H), 1.21 (s, 9H). ^{13}C NMR (100 MHz, CDCl$_3$) δ = 202.3, 179.2, 173.2, 172.0, 171.8, 52.6, 52.5, 52.0, 51.7, 43.3, 38.9, 31.5, 30.0, 27.5, 27.1, 17.7. HR-

MS(ESI) *m/z* calcd for $C_{18}H_{31}N_2O_7$ $[M+H]^+$ 387.2126, found 387.2125. $[\alpha]_D^{27}$ = 34.0° (*c* = 0.2, $CHCl_3$).

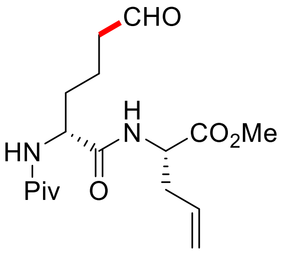

Methyl (*S*)-2-[(*R*)-6-oxo-2-pivalamidohexanamido]pent-4-enoate (9b): The general procedure **A** was followed using substrate **9a** (0.3 mmol, 97 mg). Isolation by column chromatography (PE/EA: 2/1) yielded **9b** (52 mg, 51%) as a colorless liquid. ^{1}H NMR (400 MHz, $CDCl_3$) δ = 9.75 (s, 1H), 6.90 (d, J = 9.0 Hz, 1H), 6.38 (d, J = 7.5 Hz, 1H), 5.75 – 5.58 (m, 1H), 5.12 (d, J = 12.9 Hz, 2H), 4.66 – 4.55 (m, 1H), 4.50 – 4.38 (m, 1H), 3.72 (s, 3H), 2.65 – 2.43 (m, 4H), 1.92 – 1.80 (m, 1H), 1.75 – 1.54 (m, 3H), 1.21 (s, 9H). ^{13}C NMR (100 MHz, $CDCl_3$) δ = 202.2, 179.1, 171.9, 171.4, 132.2, 119.5, 52.6, 52.5, 51.8, 43.4, 38.9, 36.4, 31.6, 27.6, 17.8. HR-MS(ESI) *m/z* calcd for $C_{17}H_{29}N_2O_5$ $[M+H]^+$ 341.2071, found 341.2072. $[\alpha]_D^{27}$ = 41.7° (*c* = 0.1, $CHCl_3$).

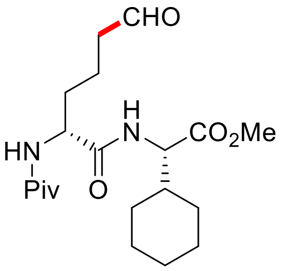

Methyl (*S*)-2-cyclohexyl-2-[(*R*)-6-oxo-2-pivalamidohexanamido]acetate (10b): The general procedure **A** was followed using substrate **10a** (0.3 mmol, 110 mg). Isolation by column chromatography (PE/EA: 2/1) yielded **10b** (57 mg, 50%) as a white solid. M.p. = 98–99 °C. ^{1}H NMR (400 MHz, $CDCl_3$) δ = 9.75 (s, 1H), 7.02 (d, J = 8.5 Hz, 1H), 6.43 (d, J = 5.2 Hz, 1H), 4.51 – 4.39 (m, 2H), 3.69 (s, 3H), 2.60 – 2.44 (m, 2H), 2.18 – 2.04 (m, 1H), 1.95 – 1.84 (m, 1H), 1.83 – 1.51 (m, 9H), 1.20 (s, 9H), 1.16 – 0.95 (m, 4H). ^{13}C NMR (100 MHz, $CDCl_3$) δ = 202.3, 179.1, 172.2, 171.7, 57.1, 52.5, 52.2, 43.4, 40.7, 38.9, 31.5, 29.7, 28.2, 27.5, 26.0, 17.8. HR-MS(ESI) *m/z* calcd for $C_{20}H_{35}N_2O_5$ $[M+H]^+$ 383.2541, found 383.2537. $[\alpha]_D^{27}$ = 47.0° (*c* = 0.17, $CHCl_3$).

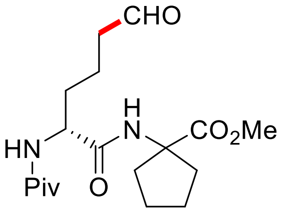

Methyl (*R*)-1-(6-oxo-2-pivalamidohexanamido)cyclopentane-1-carboxylate (11b): The general procedure **A** was followed using substrate **11a** (0.3 mmol, 101 mg). Isolation by column chromatography (PE/EA: 2/1) yielded **11b** (74 mg, 70%) as a white solid. M.p. = 129–130 °C. ^{1}H NMR (400 MHz, $CDCl_3$) δ = 9.74 (s, 1H), 7.32 (s, 1H), 6.50 (d, J = 7.4 Hz, 1H), 4.48 – 4.36 (m, 1H), 3.68 (s, 3H), 2.57 – 2.43 (m, 2H), 2.31 – 2.11 (m, 2H), 1.98 – 1.88 (m, 2H), 1.87 – 1.56 (m, 8H), 1.19 (s, 9H). ^{13}C NMR (100 MHz, $CDCl_3$) δ = 202.4, 179.0, 174.4, 171.2, 66.0, 52.5, 52.2, 43.5, 38.9, 37.5, 37.2, 31.8, 27.5, 24.6, 17.6. HR-MS(ESI) *m/z* calcd for $C_{18}H_{31}N_2O_5$ $[M+H]^+$ 355.2228, found 355.2224.

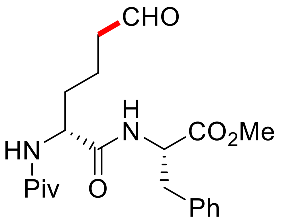

Methyl [(*R*)-6-oxo-2-pivalamidohexanoyl]-*L*-phenylalaninate (12b): The general procedure **A** was followed using substrate **12a** (0.3 mmol, 112 mg). Isolation by column chromatography (PE/EA: 2/1) yielded **12b** (54 mg, 46%) as a colorless liquid. ^{1}H NMR (400 MHz, $CDCl_3$) δ = 9.70 (s, 1H), 7.33 – 7.18 (m, 3H), 7.17 – 7.08 (m, 2H), 6.82 (d, J = 8.1 Hz, 1H), 6.31 (d, J = 7.7 Hz, 1H), 4.88 – 4.79 (m, 1H), 4.45 – 4.34 (m, 1H), 3.72 (s, 3H), 3.25 – 2.96 (m, 2H), 2.54 – 2.28 (m, 2H), 1.82 – 1.66 (m, 2H), 1.52 – 1.39 (m, 2H), 1.18 (s, 9H). ^{13}C NMR (100 MHz, $CDCl_3$) δ = 202.2, 178.9, 171.8, 171.3, 136.0, 129.3, 128.8, 127.3, 53.3, 52.6, 52.4, 43.4, 38.9, 38.0, 31.7, 27.5, 17.6. HR-MS(ESI) *m/z* calcd for $C_{21}H_{31}N_2O_5$ $[M+H]^+$ 391.2227, found 391.2221. $[\alpha]_D^{21}$ = 64.0° (c = 0.05, CH_2Cl_2).

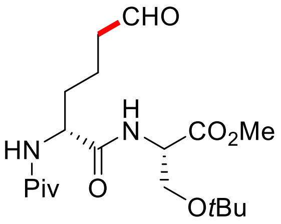

Methyl *O*-(tert-butyl)-*N*-[(*R*)-6-oxo-2-pivalamidohexanoyl]-*L*-serinate (13b): The general procedure **A** was followed using substrate **13a** (0.3 mmol, 111 mg). Isolation

by column chromatography (PE/EA: 2/1) yielded **13b** (73 mg, 63%) as a colorless liquid. ^{1}H NMR (400 MHz, $CDCl_3$) δ = 9.74 (s, 1H), 6.93 (d, J = 8.3 Hz, 1H), 6.40 (d, J = 7.7 Hz, 1H), 4.67 – 4.59 (m, 1H), 4.54 – 4.45 (m, 1H), 3.78 (dd, J = 9.0, 3.1 Hz, 1H), 3.71 (s, 3H), 3.54 (dd, J = 9.0, 3.3 Hz, 1H), 2.59 – 2.42 (m, 2H), 1.98 – 1.81 (m, 1H), 1.74 – 1.56 (m, 3H), 1.21 (s, 9H), 1.11 (s, 9H). ^{13}C NMR (100 MHz, $CDCl_3$) δ = 202.1, 178.8, 171.5, 170.7, 73.6, 61.7, 53.0, 52.5, 52.4, 43.4, 38.9, 31.7, 27.6, 27.3, 17.8. HR-MS(ESI) *m/z* calcd for $C_{19}H_{35}N_2O_6$ $[M+H]^+$ 387.2490, found 387.2485. $[\alpha]_D^{24}$ = 62.0° (c = 0.1, CH_2Cl_2).

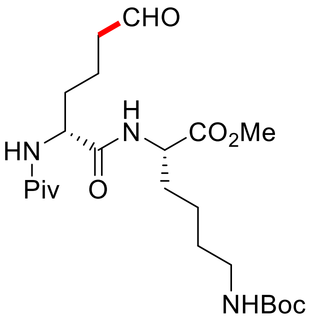

Methyl N^6-(tert-butoxycarbonyl)-N^2-[(*R*)-6-oxo-2-pivalamidohexanoyl]-*L*-lysinate (14b): The general procedure **A** was followed using substrate **14a** (0.3 mmol, 137 mg). Isolation by column chromatography (PE/EA: 2/1) yielded **14b** (62 mg, 44%) as a colorless liquid. ^{1}H NMR (400 MHz, $CDCl_3$) δ = 9.75 (s, 1H), 7.06 (d, J = 8.0 Hz, 1H), 6.43 (d, J = 7.5 Hz, 1H), 4.58 – 4.39 (m, 2H), 3.70 (s, 3H), 3.12 – 3.01 (m, 2H), 2.59 – 2.46 (m, 2H), 1.91 – 1.79 (m, 2H), 1.75 – 1.54 (m, 4H), 1.54 – 1.43 (m, 2H), 1.41 (s, 9H), 1.41 – 1.28 (m, 2H), 1.21 (s, 9H). ^{13}C NMR (100 MHz, $CDCl_3$) δ = 202.3, 179.1, 172.5, 171.6, 156.2, 79.2, 52.6, 52.5, 52.2, 43.4, 40.2, 38.9, 31.8, 31.5, 29.5, 28.5, 27.6, 22.6, 17.7. HR-MS(ESI) *m/z* calcd for $C_{23}H_{42}N_3O_7$ $[M+H]^+$ 472.3017, found 472.3014. $[\alpha]_D^{24}$ = 63.0° (c = 0.1, CH_2Cl_2).

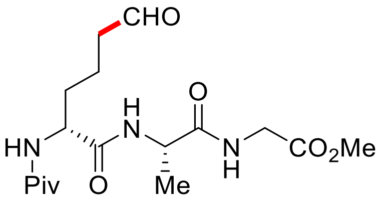

Methyl [(*R*)-6-oxo-2-pivalamidohexanoyl]-*L*-alanylglycinate (15b): The general procedure **A** was followed using substrate **15a** (0.3 mmol, 107 mg). Isolation by column chromatography (DCM/MeOH: 30/1) yielded **15b** (81 mg, 73%) as a colorless liquid. ^{1}H NMR (400 MHz, $CDCl_3$) δ = 9.74 (s, 1H), 7.47 (d, J = 7.5 Hz, 1H), 7.40 (t, J = 5.9 Hz, 1H), 6.64 (d, J = 7.4 Hz, 1H), 4.59 – 4.45 (m, 1H), 4.42 – 4.31 (m, 1H), 4.12 – 3.82

(m, 2H), 3.71 (s, 3H), 2.59 – 2.41 (m, 2H), 1.91 – 1.75 (m, 1H), 1.74 – 1.49 (m, 3H), 1.37 (d, J = 6.9 Hz, 3H), 1.18 (s, 9H). ^{13}C NMR (100 MHz, $CDCl_3$) δ = 202.4, 179.7, 172.8, 172.1, 170.6, 53.3, 52.5, 48.9, 43.3, 41.2, 38.8, 31.4, 27.4, 17.9, 17.9. HR-MS(ESI) m/z calcd for $C_{17}H_{30}N_3O_6$ $[M+H]^+$ 372.2129, found 372.2123.

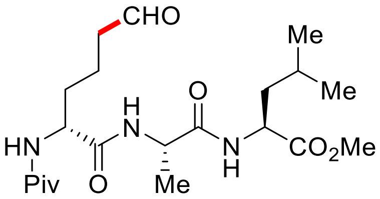

Methyl [(*R*)-6-oxo-2-pivalamidohexanoyl]-*L*-alanyl-*L*-leucinate (16b): The general procedure **A** was followed using substrate **16a** (0.3 mmol, 123 mg). Isolation by column chromatography (DCM/MeOH: 30/1) yielded **16b** (90 mg, 70%) as a colorless liquid. ^{1}H NMR (400 MHz, $CDCl_3$) δ = 9.74 (s, 1H), 7.16 (d, J = 7.6 Hz, 1H), 6.85 (d, J = 8.1 Hz, 1H), 6.52 (d, J = 7.3 Hz, 1H), 4.58 – 4.45 (m, 2H), 4.46 – 4.35 (m, 1H), 3.70 (s, 3H), 2.59 – 2.43 (m, 2H), 1.89 – 1.78 (m, 1H), 1.73 – 1.51 (m, 6H), 1.37 (d, J = 7.1 Hz, 3H), 1.20 (s, 9H), 0.90 (d, J = 5.9 Hz, 3H), 0.89 (d, J = 5.9 Hz, 3H). ^{13}C NMR (100 MHz, $CDCl_3$) δ = 202.2, 179.2, 173.3, 171.9, 171.7, 52.9, 52.4, 51.0, 49.0, 43.4, 41.1, 38.9, 31.8, 27.5, 24.9, 22.9, 21.9, 18.1, 17.8. HR-MS(ESI) m/z calcd for $C_{21}H_{38}N_3O_6$ $[M+H]^+$ 428.2755, found 428.2750. $[\alpha]_D^{27}$ = 19.0° (c = 0.2, $CHCl_3$).

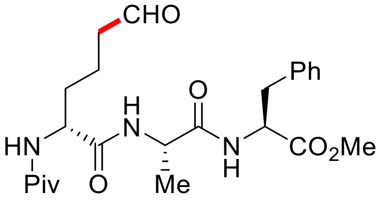

Methyl [(*R*)-6-oxo-2-pivalamidohexanoyl]-*L*-alanyl-*L*-phenylalaninate (17b): The general procedure **A** was followed using substrate **17a** (0.3 mmol, 134 mg). Isolation by column chromatography (DCM/MeOH: 30/1) yielded **17b** (62 mg, 45%) as a colorless liquid. ^{1}H NMR (400 MHz) δ = 9.75 (s, 1H), 7.31 – 7.16 (m, 3H), 7.14 – 7.06 (m, 2H), 6.95 (d, J = 7.4 Hz, 1H), 6.71 (d, J = 7.6 Hz, 1H), 6.45 (d, J = 7.0 Hz, 1H), 4.83 – 4.75 (m, 1H), 4.48 – 4.31 (m, 2H), 3.69 (s, 3H), 3.09 (qd, J = 13.8, 6.2 Hz, 2H),2.59 – 2.45 (m, 2H), 1.92 – 1.79 (m, 1H), 1.73 – 1.53 (m, 3H), 1.31 (d, J = 7.1 Hz, 3H), 1.20 (s, 9H). ^{13}C NMR (100 MHz) δ = 202.2, 179.3, 171.9, 171.7, 171.6, 136.0, 129.4, 128.7, 127.2, 53.5, 52.9, 52.5, 49.1, 43.4, 38.9, 37.8, 31.6, 27.5, 18.2, 17.7. HR-

MS(ESI) *m/z* calcd for: $C_{24}H_{36}N_3O_6$ $[M+H]^+$ 462.2599, found 462.2596. $[\alpha]_D^{21}$ = 54.0° (*c* = 0.05, CH_2Cl_2).

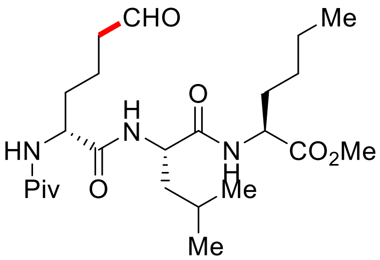

Methyl (*S*)-2-{(*S*)-4-methyl-2-[(*R*)-6-oxo-2-pivalamidohexanamido]pentanamido} hexanoate (18b): The general procedure **A** was followed using substrate **18a** (0.3 mmol, 136 mg). Isolation by column chromatography (DCM/MeOH: 50/1) yielded **18b** (77 mg, 55%) as a white solid. M.p. = 103–104 °C. ^{1}H NMR (400 MHz, $CDCl_3$) δ = 9.72 (s, 1H), 7.22 (d, *J* = 8.2 Hz, 1H), 6.86 (d, *J* = 7.9 Hz, 1H), 6.55 (d, *J* = 7.4 Hz, 1H), 4.51 – 4.38 (m, 3H), 3.69 (s, 3H), 2.57 – 2.42 (m, 2H), 1.88 – 1.72 (m, 2H), 1.70 – 1.51 (m, 7H), 1.31 – 1.24 (m, 4H), 1.19 (s, 9H), 0.95 – 0.80 (m, 9H). ^{13}C NMR (100 MHz, $CDCl_3$) δ = 202.1, 179.1, 172.9, 172.0, 171.7, 52.9, 52.4, 51.9, 43.3, 40.9, 38.9, 31.9, 31.8, 29.8, 27.5, 24.8, 23.0, 22.3, 21.9, 17.8, 13.9. HR-MS(ESI) *m/z* calcd for $C_{24}H_{44}N_3O_6$ $[M+H]^+$ 470.3225, found 470.3227. $[\alpha]_D^{27}$ = -5.5° (*c* = 0.2, $CHCl_3$).

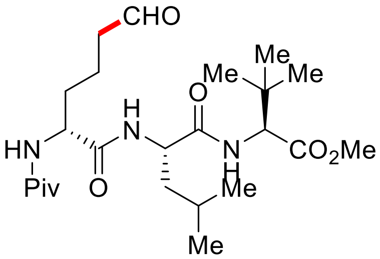

Methyl (*S*)-3,3-dimethyl-2-{(*S*)-4-methyl-2-[(*R*)-6-oxo-2-pivalamidohexanamido] pentanamido}butanoate (19b): The general procedure **A** was followed using substrate **19a** (0.3 mmol, 136 mg). Isolation by column chromatography (DCM/MeOH: 50/1) yielded **19b** (87 mg, 62%) as a white solid. M.p. = 114–115 °C. ^{1}H NMR (400 MHz, $CDCl_3$) δ = 9.71 (s, 1H), 7.69 (d, *J* = 8.5 Hz, 1H), 6.98 (d, *J* = 6.8 Hz, 1H), 6.53 (d, *J* = 7.7 Hz, 1H), 4.56 – 4.46 (m, 1H), 4.44 – 4.39 (m, 1H), 4.35 (d, *J* = 9.6 Hz, 1H), 3.68 (s, 3H), 2.55 – 2.42 (m, 2H), 1.87 – 1.75 (m, 1H), 1.70 – 1.53 (m, 6H), 1.17 (s, 9H), 0.90 (s, 12H), 0.85 (d, *J* = 5.6 Hz, 3H). ^{13}C NMR (100 MHz, $CDCl_3$) δ = 202.1, 178.7,

172.4, 171.7, 171.5, 60.2, 52.5, 52.0, 51.9, 43.3, 39.6, 38.8, 34.9, 32.4, 27.5, 26.6, 24.7, 23.1, 21.7, 17.8. HR-MS(ESI) *m/z* calcd for $C_{24}H_{44}N_3O_6$ $[M+H]^+$ 470.3225, found 470.3228. $[\alpha]_D^{27}$ = -19.5° (*c* = 0.2, $CHCl_3$).

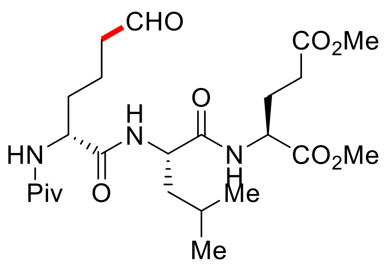

Dimethyl [(*R*)-6-oxo-2-pivalamidohexanoyl]-*L*-leucyl-*L*-glutamate (20b): The general procedure **A** was followed using substrate **20a** (0.3 mmol, 145 mg). Isolation by column chromatography (DCM/MeOH: 50/1) yielded **20b** (75 mg, 50%) as a colorless liquid. ^{1}H NMR (400 MHz, $CDCl_3$) δ = 9.73 (s, 1H), 7.20 – 6.99 (m, 2H), 6.56 (d, *J* = 6.8 Hz, 1H), 4.58 – 4.47 (m, 1H), 4.46 – 4.34 (m, 2H), 3.70 (s, 3H), 3.64 (s, 3H), 2.60 – 2.46 (m, 2H), 2.39 – 2.33 (m, 2H), 2.22 – 2.14 (m, 1H), 2.01 – 1.92 (m, 1H), 1.91 – 1.77 (m, 1H), 1.71 – 1.48 (m, 6H), 1.19 (s, 9H), 0.91 (d, *J* = 6.0 Hz, 3H), 0.88 (d, *J* = 6.0 Hz, 3H). ^{13}C NMR (100 MHz, $CDCl_3$) δ = 202.2, 179.4, 173.4, 172.0, 53.0, 52.6, 52.0, 51.7, 43.3, 40.8, 38.9, 31.5, 30.1, 29.8, 27.5, 27.0, 24.8, 23.0, 21.9, 17.9. HR-MS(ESI) *m/z* calcd for $C_{24}H_{42}N_3O_8$ $[M+H]^+$ 500.2967, found 500.2966. $[\alpha]_D^{27}$ = -5.3° (*c* = 0.2, $CHCl_3$).

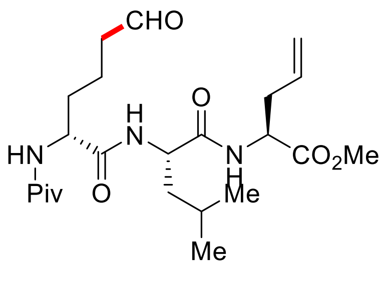

Methyl (*S*)-2-{(*S*)-4-methyl-2-[(*R*)-6-oxo-2-pivalamidohexanamido]pentanamido} pent-4-enoate (21b): The general procedure **A** was followed using substrate **21a** (0.3 mmol, 131 mg). Isolation by column chromatography (DCM/MeOH: 50/1) yielded **21b** (63 mg, 46%) as a white solid. M.p. = 122–123 °C. ^{1}H NMR (400 MHz, $CDCl_3$) δ = 9.75 (s, 1H), 7.31 (d, *J* = 7.9 Hz, 1H), 6.91 (d, *J* = 7.6 Hz, 1H), 6.57 (d, *J* = 7.4 Hz, 1H), 5.73 – 5.58 (m, 1H), 5.15 – 5.03 (m, 2H), 4.64 – 4.55 (m, 1H), 4.53 – 4.40 (m, 2H),

3.72 (s, 3H), 2.61 – 2.43 (m, 4H), 1.93 – 1.80 (m, 1H), 1.74 – 1.52 (m, 6H), 1.22 (s, 9H), 0.94 (d, *J* = 5.3 Hz, 3H), 0.90 (d, *J* = 5.3 Hz, 3H). ^{13}C NMR (100 MHz, $CDCl_3$) δ = 202.1, 179.0, 172.0, 171.9, 171.6, 132.3, 119.2, 52.8, 52.4, 51.9, 43.3, 40.7, 38.9, 36.3, 32.0, 29.8, 27.5, 24.8, 23.0, 21.8, 17.8. HR-MS(ESI) *m/z* calcd for $C_{23}H_{40}N_3O_6$ $[M+H]^+$ 454.2912, found 454.2915. $[\alpha]_D^{27}$ = 7.9° (*c* = 0.17, $CHCl_3$).

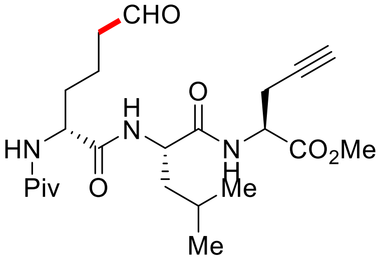

Methyl (*S*)-2-{(*S*)-4-methyl-2-[(*R*)-6-oxo-2-pivalamidohexanamido]pentanamido} pent-4-ynoate (22b): The general procedure **A** was followed using substrate **22a** (0.3 mmol, 131 mg). Isolation by column chromatography (DCM/MeOH: 50/1) yielded **22b** (64 mg, 47%) as a colorless liquid. ^1H NMR (400 MHz, $CDCl_3$) δ = 9.73 (s, 1H), 7.31 (d, *J* = 7.2 Hz, 1H), 7.12 (d, *J* = 6.7 Hz, 1H), 6.56 (d, *J* = 7.5 Hz, 1H), 4.70 – 4.62 (m, 1H), 4.54 – 4.40 (m, 2H), 3.74 (s, 3H), 2.77 – 2.65 (m, 2H), 2.57 – 2.46 (m, 2H), 2.01 (t, *J* = 2.5 Hz, 1H), 1.92 – 1.77 (m, 1H), 1.72 – 1.53 (m, 6H), 1.19 (s, 9H), 0.92 (d, *J* = 5.5 Hz, 3H), 0.89 (d, *J* = 5.5 Hz, 3H). ^{13}C NMR (100 MHz, $CDCl_3$) δ = 202.2, 179.2, 172.1, 171.9, 170.7, 78.6, 71.8, 52.84, 52.80, 51.8, 50.8, 43.3, 40.8, 38.9, 31.8, 27.5, 24.8, 23.1, 22.2, 21.8, 17.8. HR-MS(ESI) *m/z* calcd for $C_{23}H_{38}N_3O_6$ $[M+H]^+$ 452.2755, found 452.2752. $[\alpha]_D^{27}$ = 47.3° (*c* = 0.1, $CHCl_3$).

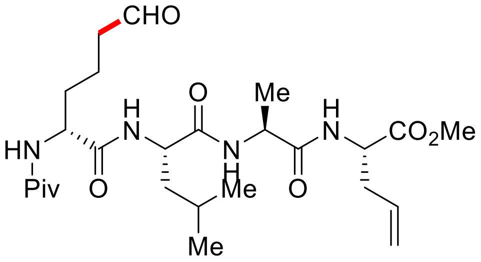

Methyl (2*S*,5*S*,8*S*,11*R*)-2-allyl-8-isobutyl-5,14,14-trimethyl-4,7,10,13-tetraoxo-11-(4-oxobutyl)-3,6,9,12-tetraazapentadecanoate (23b): The general procedure **A** was followed using substrate **23a** (0.3 mmol, 152 mg). Isolation by column chromatography (DCM/MeOH: 30/1) yielded **23b** (82 mg, 52%) as a white solid. M.p. = 158–159 °C.

^{1}H NMR (400 MHz, $CDCl_3$) δ = 9.71 (s, 1H), 7.70 (d, J = 7.7 Hz, 2H), 7.11 (d, J = 7.8 Hz, 1H), 6.69 (d, J = 6.8 Hz, 1H), 5.78 – 5.64 (m, 1H), 5.15 – 5.01 (m, 2H), 4.81 – 4.70 (m, 1H), 4.70 – 4.61 (m, 1H), 4.61 – 4.47 (m, 2H), 3.70 (s, 3H), 2.61 – 2.39 (m, 4H), 1.87 – 1.74 (m, 1H), 1.71 – 1.50 (m, 6H), 1.26 (d, J = 7.0 Hz, 3H), 1.19 (s, 9H), 0.92 (d, J = 6.0 Hz, 3H), 0.91 (d, J = 6.0 Hz, 3H). ^{13}C NMR (100 MHz, $CDCl_3$) δ = 201.9, 179.0, 172.3, 172.0, 171.8, 171.4, 132.6, 118.9, 52.7, 52.4, 52.2, 51.9, 48.6, 43.4, 41.9, 38.8, 36.3, 32.7, 27.5, 24.9, 23.1, 22.2, 18.9, 17.7. HR-MS(ESI) *m/z* calcd for $C_{26}H_{45}N_4O_7$ $[M+H]^+$ 525.3283, found 525.3283. $[\alpha]_D^{27}$ = -29.5° (c = 0.14, $CHCl_3$).

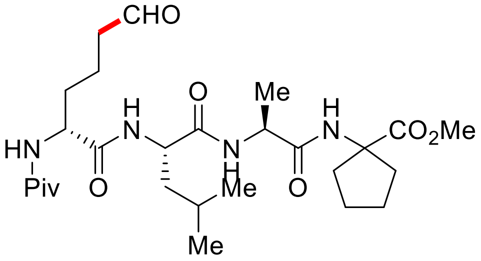

Methyl 1-{(*S*)-2-{(*S*)-4-methyl-2-[(*R*)-6-oxo-2-pivalamidohexanamido]pentanamido}propanamido}cyclopentane-1-carboxylate (24b): The general procedure **A** was followed using substrate **24a** (0.3 mmol, 157 mg). Isolation by column chromatography (DCM/MeOH: 30/1) yielded **24b** (82 mg, 51%) as a white solid. M.p. = 179–180 °C. ^{1}H NMR (400 MHz, $CDCl_3$) δ = 9.71 (s, 1H), 7.82 (d, J = 7.9 Hz, 1H), 7.75 (s, 1H), 7.10 (d, J = 8.4 Hz, 1H), 6.75 (d, J = 6.8 Hz, 1H), 4.74 – 4.59 (m, 2H), 4.54 – 4.42 (m, 1H), 3.65 (s, 3H), 2.55 – 2.38 (m, 2H),2.25 – 2.13 (m, 2H), 2.03 – 1.87 (m, 2H), 1.85 – 1.50 (m, 11H), 1.25 (d, J = 7.0 Hz, 3H), 1.19 (s, 9H), 0.93 (d, J = 5.4 Hz, 3H), 0.91 (d, J = 5.4 Hz, 3H). ^{13}C NMR (100 MHz, $CDCl_3$) δ = 201.9, 179.0, 174.5, 172.2, 171.9, 171.4, 65.8, 52.8, 52.4, 52.1, 48.5, 43.4, 41.9, 38.8, 37.2, 32.6, 27.5, 25.0, 24.6, 23.1, 22.1, 18.7, 17.6. HR-MS(ESI) *m/z* calcd for $C_{27}H_{47}N_4O_7$ $[M+H]^+$ 539.3439, found 539.3441. $[\alpha]_D^{27}$ = -43.3° (c = 0.05, $CHCl_3$).

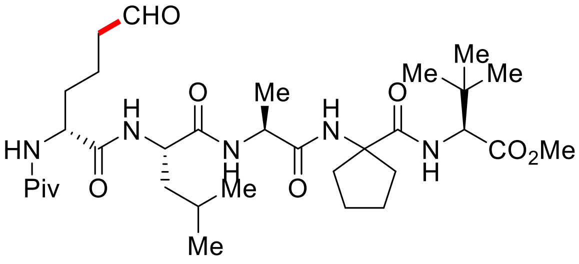

Methyl (*S*)-3,3-dimethyl-2-{1-{(*S*)-2-{(*S*)-4-methyl-2-[(*R*)-6-oxo-2-pivalamidohex-anamido]pentanamido}propanamido}cyclopentane-1-carboxamido}butanoate (25b): The general procedure **A** was followed using substrate **25a** (0.3 mmol, 191 mg). Isolation by column chromatography (DCM/MeOH: 50/1) yielded **25b** (80 mg, 41%) as a white solid. M.p. = 155–156 °C. ^{1}H NMR (400 MHz, $CDCl_3$) δ = 9.73 (s, 1H), 7.64 (d, J = 9.1 Hz, 1H), 7.49 (s, 1H), 7.46 (d, J = 7.5 Hz, 1H), 7.08 (d, J = 7.7 Hz, 1H), 6.70 (d, J = 6.3 Hz, 1H), 4.58 (q, J = 7.3 Hz, 1H), 4.50 – 4.30 (m, 3H), 3.65 (s, 3H), 2.58 – 2.42 (m, 2H), 2.34 – 2.24 (m, 1H), 2.22 – 2.12 (m, 2H), 1.99 – 1.90 (m, 1H), 1.86 – 1.53 (m, 11H), 1.34 (d, J = 7.0 Hz, 3H), 1.18 (s, 9H), 0.95 (s, 9H), 0.93 (d, J = 5.9 Hz, 3H), 0.91 (d, J = 5.9 Hz, 3H). ^{13}C NMR (100 MHz, $CDCl_3$) δ = 202.0, 179.5, 173.6, 173.3, 172.3, 172.0, 171.6, 67.9, 60.5, 53.4, 52.4, 51.6, 49.5, 43.3, 41.4, 38.8, 36.5, 36.3, 34.9, 31.9, 27.5, 26.7, 25.0, 24.0, 23.8, 23.2, 21.8, 18.3, 17.6. HR-MS(ESI) *m/z* calcd for $C_{33}H_{58}N_5O_8$ $[M+H]^+$ 652.4280, found 652.4279. $[\alpha]_D^{27}$ = -2.0° (c = 0.09, $CHCl_3$).

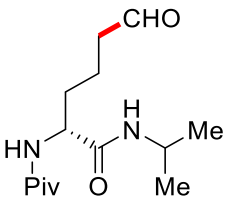

(*R*)-*N*-Isopropyl-6-oxo-2-pivalamidohexanamide (26b): The general procedure **A** was followed using substrate **26a** (0.3 mmol, 76 mg). Isolation by column chromatography (PE/EA: 5:1) yielded **26b** (55 mg, 67%) as a colorless liquid. ^{1}H NMR (400 MHz, $CDCl_3$) δ = 9.74 (s, 1H), 6.54 (d, J = 8.0 Hz, 1H), 6.48 (d, J = 8.0 Hz, 1H), 4.36 (q, J = 6.6 Hz, 1H), 4.08 – 3.98 (m, 1H), 2.54 – 2.44 (m, 2H), 1.87 – 1.76 (m, 1H), 1.68 – 1.55 (m, 3H), 1.20 (s, 9H), 1.14 (dd, J = 11.5, 6.7 Hz, 6H). ^{13}C NMR (100 MHz, $CDCl_3$) δ = 202.3, 178.8, 170.6, 52.5, 43.4, 41.6, 38.8, 32.0, 27.5, 22.7, 22.6, 17.7. HR-MS (ESI) *m/z* calcd for $C_{14}H_{27}N_2O_3$ $[M+H]^+$ 271.2016, found 271.2015. $[\alpha]_D^{28}$ = 32.0° (c = 0.1, CH_2Cl_2).

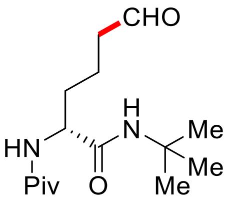

(*R*)-*N*-(*tert*-Butyl)-6-oxo-2-pivalamidohexanamide (27b): The general procedure **A**

was followed using substrate **27a** (0.3 mmol, 81 mg). Isolation by column chromatography (PE/EA: 5:1) yielded **27b** (46 mg, 53%) as a colorless liquid. ^{1}H NMR (400 MHz, CDCl$_3$) δ = 9.75 (s, 1H), 6.45 (d, J = 7.7 Hz, 1H), 6.30 (d, J = 5.0 Hz, 1H), 4.28 (q, J = 6.5, 1H), 2.53 – 2.49 (m, 2H), 1.82 – 1.73 (m, 1H), 1.69 – 1.55 (m, 3H), 1.33 (s, 9H), 1.20 (s, 9H). ^{13}C NMR (100 MHz, CDCl$_3$) δ = 202.3, 178.7, 170.7, 52.8, 51.5, 43.5, 38.8, 32.0, 28.8, 27.5, 17.7. HR-MS (ESI) *m/z* calcd for $C_{15}H_{29}N_2O_3$ $[M+H]^+$ 285.2173, found 285.2169.

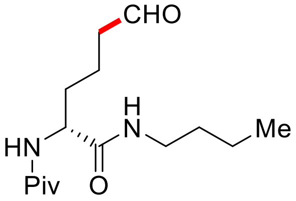

(*R*)-*N*-(*tert*-Butyl)-6-oxo-2-pivalamidohexanamide (28b): The general procedure **A** was followed using substrate **28a** (0.3 mmol, 81 mg). Isolation by column chromatography (PE/EA: 3:1) yielded **28b** (68 mg, 80%) as a colorless liquid. ^{1}H NMR (400 MHz, CDCl$_3$) δ = 9.70 (s, 1H), 7.14 (d, J = 5.3 Hz, 1H), 6.58 (d, J = 6.8 Hz, 1H), 4.46 (q, J = 6.9 Hz, 1H), 3.30 – 3.06 (m, 2H), 2.47 – 2.42 (m, , 2H), 1.84 – 1.71 (m, 1H), 1.64 – 1.52 (m, 3H), 1.48 – 1.38 (m, 2H), 1.29 (p, J = 7.4 Hz, 2H), 1.16 (s, 9H), 0.86 (t, J = 7.3 Hz, 3H). ^{13}C NMR (100 MHz, CDCl$_3$) δ = 202.2, 178.8, 171.6, 52.5, 43.4, 39.3, 38.8, 32.3, 31.6, 27.5, 20.1, 17.8, 13.8. HR-MS (ESI) *m/z* calcd for $C_{15}H_{29}N_2O_3$ $[M+H]^+$ 285.2173, found 285.2173. $[\alpha]_D^{28}$ = 41.0° (c = 0.1, CH_2Cl_2).

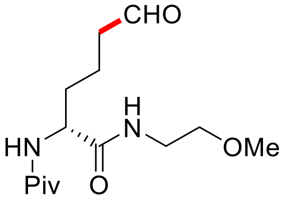

(*R*)-*N*-(2-Methoxyethyl)-6-oxo-2-pivalamidohexanamide (29b): The general procedure **A** was followed using substrate **29a** (0.3 mmol, 81 mg). Isolation by column chromatography (PE/EA: 1:1) yielded **29b** (46 mg, 54%) as a colorless liquid. ^{1}H NMR (400 MHz, CDCl$_3$) δ = 9.72 (s, 1H), 6.88 (d, J = 7.9 Hz, 1H), 6.48 (d, J = 7.8 Hz, 1H), 4.43 (q, J = 6.7 Hz, 1H), 3.46 – 3.34 (m, 4H), 3.30 (s, 3H), 2.52 – 2.44 (m, 2H), 1.87 – 1.74 (m, 1H), 1.69 – 1.55 (m, 3H), 1.18 (s, 9H). ^{13}C NMR (100 MHz, CDCl$_3$) δ = 202.2, 178.7, 171.7, 71.0, 58.8, 52.5, 43.4, 39.3, 38.8, 32.2, 27.5, 17.8. HR-MS (ESI) *m/z* calcd

for $C_{14}H_{27}N_2O_4$ [M+H]$^+$ 287.1965, found 287.1959.

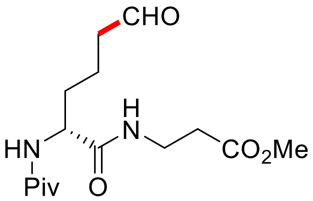

Methyl (*R*)-3-(6-oxo-2-pivalamidohexanamido)propanoate (30b): The general procedure **A** was followed using substrate **30a** (0.3 mmol, 90 mg). Isolation by column chromatography (PE/EA: 3:1) yielded **30b** (45 mg, 47%) as a colorless liquid. ^{1}H NMR (400 MHz, $CDCl_3$) δ = 9.71 (s, 1H), 7.13 (d, J = 12.8 Hz, 1H), 6.49 (d, J = 7.6 Hz, 1H), 4.39 (q, J = 6.4 Hz, 1H), 3.65 (s, 3H), 3.57 – 3.39 (m, 2H), 2.54 – 2.45 (m, 4H), 1.82 – 1.76 (m, 1H), 1.66 – 1.50 (m, 3H), 1.17 (s, 9H). ^{13}C NMR (100 MHz, $CDCl_3$) δ = 202.2, 178.8, 172.6, 171.7, 52.5, 51.9, 43.3, 38.8, 35.1, 33.8, 32.0, 27.5, 17.7. HR-MS (ESI) *m/z* calcd for $C_{15}H_{27}N_2O_5$ [M+H]$^+$ 315.1915, found 315.1906. $[\alpha]_D^{28}$ = 17.0° (c = 0.1, CH_2Cl_2).

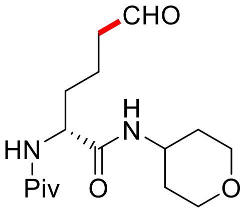

(*R*)-6-Oxo-2-pivalamido-*N*-(tetrahydro-2*H*-pyran-4-yl)hexanamide (31b): The general procedure **A** was followed using substrate **31a** (0.3 mmol, 89 mg). Isolation by column chromatography (PE/EA: 1:1) yielded **31b** (58 mg, 62%) as a colorless liquid. ^{1}H NMR (400 MHz, $CDCl_3$) δ = 9.72 (s, 1H), 7.05 (d, J = 7.9 Hz, 1H), 6.51 (d, J = 7.6 Hz, 1H), 4.42 (q, J = 6.7 Hz, 1H), 3.98 – 3.82 (m, 3H), 3.48 – 3.38 (m, 2H), 2.52 – 2.45 (m, 2H), 1.83 – 1.71 (m, 2H), 1.68 – 1.57 (m, 3H), 1.56 – 1.40 (m, 3H), 1.18 (s, 9H). ^{13}C NMR (100 MHz, $CDCl_3$) δ = 202.2, 178.9, 170.9, 66.7, 52.5, 45.8, 43.4, 38.8, 32.9, 32.8, 32.1, 27.6, 17.7. HR-MS (ESI) *m/z* calcd for $C_{16}H_{29}N_2O_4$ [M+H]$^+$ 313.2122, found 313.2121. $[\alpha]_D^{28}$ = 10.0° (c = 0.1, CH_2Cl_2).

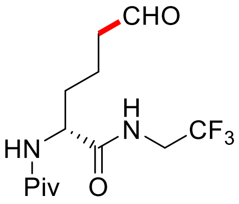

(*R*)-6-Oxo-2-pivalamido-*N*-(2,2,2-trifluoroethyl)hexanamide (32b): The general procedure **A** was followed using substrate **32a** (0.3 mmol, 88 mg). Isolation by column chromatography (PE/EA: 3:1) yielded **32b** (52 mg, 56%) as a white solid. M.p. = 117–118 °C. ^{1}H NMR (400 MHz, $CDCl_3$) δ = 9.69 (s, 1H), 8.08 (d, J = 6.4 Hz, 1H), 6.60 (d, J = 8.0 Hz, 1H), 4.66 (q, J = 6.8 Hz, 1H), 3.93 (ddd, J = 17.9, 9.1, 4.5 Hz, 1H), 3.77 (ddd, J = 12.6, 9.0, 6.2 Hz, 1H), 2.51 – 2.44 (m, 2H), 1.84 – 1.74 (m, 1H), 1.67 – 1.53 (m, 3H), 1.17 (s, 9H). ^{13}C NMR (100 MHz, $CDCl_3$) δ = 202.6, 178.9, 172.7, 124.1 (q, $^1J_{C-F}$ = 278.7 Hz), 52.3, 43.2, 40.6 (q, $^2J_{C-F}$ = 34.9 Hz), 38.8, 31.9, 27.4, 17.6. ^{19}F NMR (376 MHz, $CDCl_3$) δ = -72.35 (t, J = 9.0 Hz). HR-MS (ESI) *m/z* calcd for $C_{13}H_{22}F_3N_2O_3$ $[M+H]^+$ 311.1577, found 311.1571. $[\alpha]_D^{28}$ = -14.0° (c = 0.1, CH_2Cl_2).

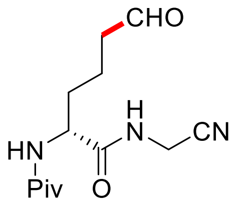

(*R*)-*N*-(Cyanomethyl)-6-oxo-2-pivalamidohexanamide (33b): The general procedure **A** was followed using substrate **33a** (0.3 mmol, 76 mg). Isolation by column chromatography (PE/EA: 3:1) yielded **33b** (48 mg, 60%) as a colorless liquid. ^{1}H NMR (400 MHz, $CDCl_3$) δ = 9.73 (s, 1H), 8.26 (d, J = 11.8 Hz, 1H), 6.64 (d, J = 7.9 Hz, 1H), 4.57 (q, J = 7.1 Hz, 1H), 4.14 (d, J = 5.5 Hz, 2H), 2.54 – 2.47 (m, 2H), 1.85 – 1.77 (m, 1H), 1.69 – 1.57 (m, 3H), 1.21 (s, 9H). ^{13}C NMR (100 MHz, $CDCl_3$) δ = 202.2, 179.7, 172.4, 116.0, 52.4, 43.2, 38.9, 31.7, 27.5, 17.8. HR-MS (ESI) *m/z* calcd for $C_{13}H_{22}N_3O_3$ $[M+H]^+$ 268.1656, found 268.1655.

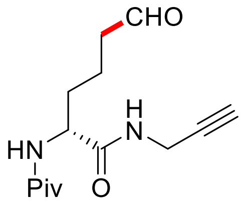

(*R*)-6-Oxo-2-pivalamido-*N*-(prop-2-yn-1-yl)hexanamide (34b): The general procedure **A** was followed using substrate **34a** (0.3 mmol, 75 mg). Isolation by column chromatography (PE/EA: 5:1) yielded **34b** (49 mg, 61%) as a colorless liquid. ^{1}H NMR (400 MHz, $CDCl_3$) δ = 9.74 (s, 1H), 7.45 (d, J = 5.5 Hz, 1H), 6.51 (d, J = 7.8 Hz, 1H), 4.54 (q, J = 6.8 Hz, 1H), 4.08 – 3.96 (m, 2H), 2.54 – 2.47 (m, 2H), 2.20 (s, 1H), 1.90 –

1.76 (m, 1H), 1.72 – 1.54 (m, 3H), 1.21 (s, 9H). ^{13}C NMR (100 MHz, $CDCl_3$) δ = 202.3, 179.1, 171.5, 79.3, 71.6, 52.3, 43.4, 38.9, 32.0, 29.1, 27.5, 17.8. HR-MS (ESI) *m/z* calcd for $C_{14}H_{23}N_2O_3$ $[M+H]^+$ 267.1703, found 267.1702.

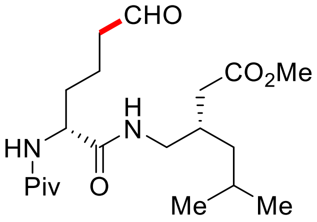

Methyl (*S*)-5-methyl-3-{[(*R*)-6-oxo-2-pivalamidohexanamido]methyl}hexanoate (35b): The general procedure **A** was followed using substrate **35a** (0.3 mmol, 111 mg). Isolation by column chromatography (PE/EA: 2:1) yielded **35b** (63 mg, 57%) as a colorless liquid. ^{1}H NMR (400 MHz, $CDCl_3$) δ = 9.74 (s, 1H), 6.91 (d, *J* = 6.1 Hz, 1H), 6.44 (d, *J* = 7.7 Hz, 1H), 4.37 (q, *J* = 6.9 Hz, 1H), 3.65 (s, 3H), 3.37 – 3.06 (m, 2H), 2.53 – 2.47 (m, 2H), 2.29 – 2.24 (m, 2H), 2.11 (p, *J* = 6.5 Hz, 1H), 1.88 – 1.75 (m, 1H), 1.68 – 1.58 (m, 4H), 1.19 (s, 9H), 1.17 – 1.02 (m, 2H), 0.87 (d, *J* = 6.5 Hz, 3H), 0.85 (d, *J* = 6.5 Hz, 3H). ^{13}C NMR (100 MHz, $CDCl_3$) δ = 202.2, 178.9, 173.6, 171.8, 52.7, 51.7, 43.4, 43.0, 41.5, 38.8, 37.3, 33.1, 31.9, 27.5, 25.2, 22.8, 22.7, 17.8. HR-MS (ESI) *m/z* calcd for $C_{20}H_{37}N_2O_5$ $[M+H]^+$ 385.2697, found 385.2696. $[\alpha]_D^{28}$ = 13.0° (*c* = 0.1, CH_2Cl_2).

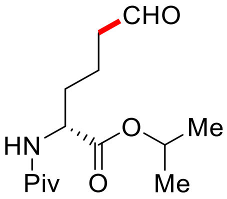

Isopropyl (*R*)-6-oxo-2-pivalamidohexanoate (36b): The general procedure **A** was followed using substrate **36a** (0.3 mmol, 77 mg). Isolation by column chromatography (PE/EA: 5:1) yielded **36b** (42 mg, 52%) as a colorless liquid. ^{1}H NMR (400 MHz, $CDCl_3$) δ = 9.74 (s, 1H), 6.26 (d, *J* = 7.6 Hz, 1H), 5.11 – 4.97 (m, 1H), 4.55 – 4.46 (m, 1H), 2.58 – 2.43 (m, 2H), 1.91 – 1.80 (m, 1H), 1.73 – 1.55 (m, 3H), 1.25 (dd, *J* = 6.4, 3.5 Hz, 6H), 1.21 (s, 9H). ^{13}C NMR (100 MHz, $CDCl_3$) δ = 201.9, 178.5, 172.1, 69.4, 51.9, 43.2, 38.9, 31.9, 27.6, 21.9, 21.8, 17.7. HR-MS(ESI) *m/z* calcd for $C_{14}H_{26}NO_4$ $[M+H]^+$ 272.1857, found 272.1855.

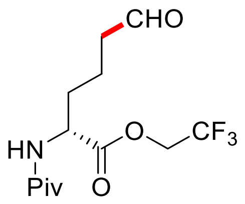

2,2,2-Trifluoroethyl (*R*)-6-oxo-2-pivalamidohexanoate (37b): The general procedure **A** was followed using substrate **37a** (0.3 mmol, 89 mg). Isolation by column chromatography (PE/EA: 3:1) yielded **37b** (52 mg, 56%) as a colorless liquid. ^{1}H NMR (400 MHz, $CDCl_3$) δ = 9.75 (s, 1H), 6.25 (d, J = 7.5 Hz, 1H), 4.69 – 4.54 (m, 2H), 4.48 – 4.32 (m, 1H), 2.62 – 2.44 (m, 2H), 1.95 – 1.81 (m, 1H), 1.78 – 1.59 (m, 3H), 1.22 (s, 9H). ^{13}C NMR (100 MHz, $CDCl_3$) δ = 201.7, 178.9, 171.1, 122.8 (q, $^1J_{C\text{-}F}$ = 277.4 Hz), 60.9 (q, $^2J_{C\text{-}F}$ = 36.8 Hz), 51.9, 43.0, 38.8, 31.2, 27.5, 17.6. ^{19}F NMR (376 MHz, $CDCl_3$) δ = -73.64 (s). HR-MS(ESI) *m/z* calcd for $C_{13}H_{21}F_3NO_4$ [M+H]$^+$ 312.1417, found 312.1417.

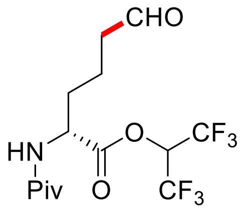

1,1,1,3,3,3-Hexafluoropropan-2-yl (*R*)-6-oxo-2-pivalamidohexanoate (38b): The general procedure **A** was followed using substrate **38a** (0.3 mmol, 109 mg). Isolation by column chromatography (PE/EA: 5:1) yielded **38b** (53 mg, 47%) as a colorless liquid. ^{1}H NMR (400 MHz, $CDCl_3$) δ = 9.78 (s, 1H), 6.29 (d, J = 7.0 Hz, 1H), 5.74 (p, J = 5.9 Hz, 1H), 4.71 – 4.60 (m, 1H), 2.65 – 2.49 (m, 2H), 1.83 – 1.60 (m, 4H), 1.24 (s, 9H). ^{13}C NMR (100 MHz, $CDCl_3$) δ = 201.6, 178.9, 169.4, 120.3 (q, $^1J_{C\text{-}F}$ = 283.5 Hz), 67.1 (p, $^2J_{C\text{-}F}$ = 34.7 Hz), 52.0, 43.0, 38.8, 30.7, 27.5, 17.4. ^{19}F NMR (376 MHz, $CDCl_3$) δ = -73.02 (d, J = 25.6 Hz), -75.47 (s). HR-MS(ESI) *m/z* calcd for $C_{14}H_{20}F_6NO_4$ [M+H]$^+$ 380.1291, found 380.1291. $[\alpha]_D^{29}$ = 21.0° (c = 0.1, CH_2Cl_2).

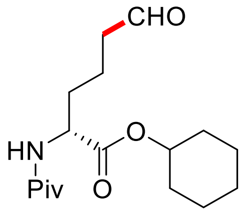

Cyclohexyl (*R*)-6-oxo-2-pivalamidohexanoate (39b): The general procedure **A** was followed using substrate **39a** (0.3 mmol, 89 mg). Isolation by column chromatography (PE/EA: 5:1) yielded **39b** (50 mg, 54%) as a colorless liquid. ^{1}H NMR (400 MHz, $CDCl_3$) δ = 9.74 (s, 1H), 6.28 (d, J = 7.6 Hz, 1H), 4.85 – 4.74 (m, 1H), 4.57 – 4.48 (m, 1H), 2.58 – 2.43 (m, 2H), 1.91 – 1.78 (m, 4H), 1.76 – 1.58 (m, 5H), 1.56 – 1.32 (m, 5H), 1.21 (s, 9H). ^{13}C NMR (100 MHz, $CDCl_3$) δ = 201.9, 178.5, 172.0, 74.2, 51.9, 43.3, 38.9, 32.0, 31.5, 27.6, 25.3, 23.7, 17.7. HR-MS(ESI) *m/z* calcd for $C_{17}H_{30}NO_4$ $[M+H]^+$ 312.2170, found 312.2168.

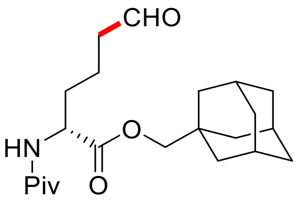

[(3*R*,5*R*,7*R*)-Adamantan-1-yl]methyl (*R*)-6-oxo-2-pivalamidohexanoate (40b): The general procedure **A** was followed using substrate **40a** (0.3 mmol, 108 mg). Isolation by column chromatography (PE/EA: 5:1) yielded **40b** (69 mg, 61%) as a colorless liquid. ^{1}H NMR (400 MHz, $CDCl_3$) δ = 9.75 (s, 1H), 6.28 (d, J = 7.6 Hz, 1H), 4.65 – 4.54 (m, 1H), 3.73 (q, J = 10.7 Hz, 2H), 2.56 – 2.45 (m, 2H), 1.98 (s, 3H), 1.96 – 1.83 (m, 1H), 1.77 – 1.58 (m, 9H), 1.51 (s, 6H), 1.22 (s, 9H). ^{13}C NMR (100 MHz, $CDCl_3$) δ = 201.8, 178.4, 172.7, 75.2, 51.8, 43.3, 39.3, 38.9, 37.0, 33.3, 32.1, 28.0, 27.6, 17.8. HR-MS(ESI) *m/z* calcd for $C_{22}H_{36}NO_4$ $[M+H]^+$ 378.2639, found 378.2640. $[\alpha]_D^{28}$ = -14.0° (c = 0.1, CH_2Cl_2).

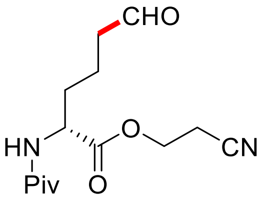

2-Cyanoethyl (*R*)-6-oxo-2-pivalamidohexanoate (41b): The general procedure **A** was followed using substrate **41a** (0.3 mmol, 80 mg). Isolation by column chromatography (PE/EA: 1:1) yielded **41b** (47 mg, 56%) as a colorless liquid. ^{1}H NMR (400 MHz, $CDCl_3$) δ = 9.75 (s, 1H), 6.26 (d, J = 7.2 Hz, 1H), 4.59 – 4.48 (m, 1H), 4.44 – 4.24 (m, 2H), 2.74 (t, J = 6.2 Hz, 2H), 2.60 – 2.48 (m, 2H), 1.96 – 1.83 (m, 1H), 1.78 – 1.59 (m, 3H), 1.22 (s, 9H). ^{13}C NMR (100 MHz, $CDCl_3$) δ = 201.9, 178.8, 172.0, 116.7, 59.5,

52.0, 43.1, 38.8, 31.3, 27.5, 18.1, 17.6. HR-MS(ESI) *m/z* calcd for $C_{14}H_{23}N_2O_4$ $[M+H]^+$ 283.1653, found 283.1652.

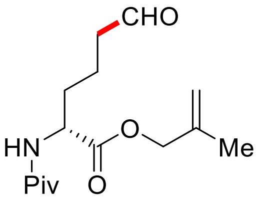

2-Methylallyl (*R*)-6-oxo-2-pivalamidohexanoate (42b): The general procedure **A** was followed using substrate **42a** (0.3 mmol, 80 mg). Isolation by column chromatography (PE/EA: 5:1) yielded **42b** (57 mg, 67%) as a colorless liquid. ^{1}H NMR (400 MHz, $CDCl_3$) δ = 9.74 (s, 1H), 6.26 (d, *J* = 7.6 Hz, 1H), 4.96 (d, *J* = 11.7 Hz, 2H), 4.65 – 4.49 (m, 3H), 2.58 – 2.43 (m, 2H), 1.97 – 1.79 (m, 2H), 1.74 (s, 3H), 1.69 – 1.60 (m, 2H), 1.21 (s, 9H). ^{13}C NMR (100 MHz, $CDCl_3$) δ = 201.8, 178.5, 172.3, 139.3, 113.9, 68.9, 51.8, 43.2, 38.9, 31.9, 27.6, 19.6, 17.7. HR-MS(ESI) *m/z* calcd for $C_{15}H_{26}NO_4$ $[M+H]^+$ 284.1857, found 284.1855. $[\alpha]_D^{28}$ = 38.0° (*c* = 0.1, CH_2Cl_2).

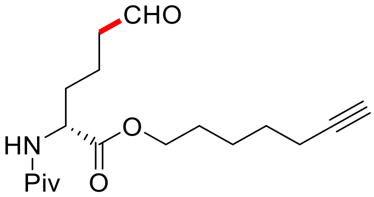

Hept-6-yn-1-yl (*R*)-6-oxo-2-pivalamidohexanoate (43b): The general procedure **A** was followed using substrate **43a** (0.3 mmol, 92 mg). Isolation by column chromatography (PE/EA: 3:1) yielded **43b** (49 mg, 51%) as a colorless liquid. ^{1}H NMR (400 MHz, $CDCl_3$) δ = 9.73 (s, 1H), 6.25 (d, *J* = 7.6 Hz, 1H), 4.59 – 4.50 (m, 1H), 4.12 (q, *J* = 4.5 Hz, 2H), 2.57 – 2.41 (m, 2H), 2.22 – 2.13 (m, 2H), 1.93 (s, 1H), 1.90 – 1.79 (m, 1H), 1.73 – 1.58 (m, 5H), 1.53 (t, J = 7.1 Hz, 2H), 1.45 (t, *J* = 7.7 Hz, 2H), 1.20 (s, 9H). ^{13}C NMR (100 MHz, $CDCl_3$) δ = 201.8, 178.5, 172.6, 84.2, 68.7, 65.5, 51.8, 43.2, 38.8, 31.9, 28.1, 28.0, 27.5, 25.0, 18.3, 17.7. HR-MS(ESI) *m/z* calcd for $C_{18}H_{30}NO_4$ $[M+H]^+$ 324.2170, found 324.2169. $[\alpha]_D^{28}$ = 11.0° (*c* = 0.1, CH_2Cl_2).

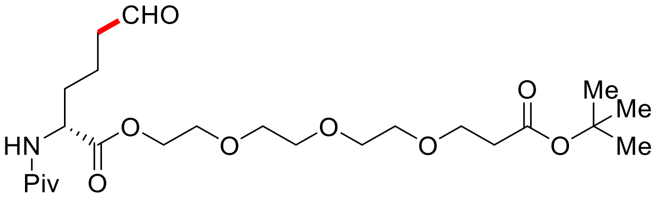

14,14-Dimethyl-12-oxo-3,6,9,13-tetraoxapentadecyl (*R*)-6-oxo-2-pivalamidohexa-

noate (44b): The general procedure **A** was followed using substrate **44a** (0.3 mmol, 142 mg). Isolation by column chromatography (PE/EA: 1:1) yielded **44b** (84 mg, 57%) as a colorless liquid. ^{1}H NMR (400 MHz, $CDCl_3$) δ = 9.74 (s, 1H), 6.33 (d, J = 7.6 Hz, 1H), 4.64 – 4.53 (m, 1H), 4.35 – 4.21 (m, 2H), 3.73 – 3.65 (m, 4H), 3.64 – 3.56 (m, 8H), 2.57 – 2.41 (m, 4H), 1.92 – 1.81 (m, 1H), 1.76 – 1.57 (m, 3H), 1.42 (s, 9H), 1.21 (s, 9H). ^{13}C NMR (100 MHz, $CDCl_3$) δ = 201.9, 178.6, 172.6, 171.0, 80.7, 70.7, 70.6, 70.4, 69.0, 67.0, 64.5, 51.8, 43.2, 38.8, 36.3, 31.8, 28.2, 27.6, 17.7. HR-MS(ESI) *m/z* calcd for $C_{24}H_{44}NO_9$ $[M+H]^+$ 490.3011, found 490.3010. $[\alpha]_D^{28}$ = -26.0° (c = 0.1, CH_2Cl_2).

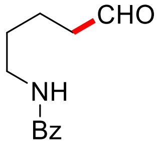

***N*-(5-Oxopentyl)benzamide (62b):** The general procedure **B** was followed using substrate **62a** (0.3 mmol, 57 mg). Isolation by column chromatography (PE/EA: 2:1) yielded **62b** (33 mg, 53%) as a colorless liquid. ^{1}H NMR (400 MHz, DMSO-d_6) δ = 9.67 (t, J = 1.5 Hz, 1H), 8.47 (t, J = 5.4 Hz, 1H), 7.88 – 7.79 (m, 2H), 7.53 – 7.48 (m, 1H), 7.47 – 7.39 (m, 2H), 3.41 – 3.38 (m, 1H), 3.26 (q, J = 6.3 Hz, 1H), 2.47 (td, J = 7.1, 1.6 Hz, 2H), 1.60 – 1.49 (m, 4H). ^{13}C NMR (100 MHz, DMSO-d_6) δ = 203.5, 166.2, 134.7, 131.1, 128.3, 127.2, 42.7, 38.9, 28.6, 19.1. HR-MS(ESI) *m/z* calcd for: $C_{12}H_{16}NO_2$ $[M+H]^+$ 206.1176, found 206.1173.

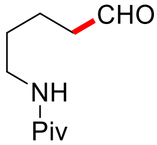

***N*-(5-Oxopentyl)pivalamide (63b):** The general procedure **B** was followed using substrate **63a** (0.3 mmol, 51 mg). Isolation by column chromatography (PE/EA: 4:1) yielded **63b** (49 mg, 88%) as a colorless liquid. ^{1}H NMR (400 MHz, $CDCl_3$) δ = 9.75 (s, 1H), 5.83 (s, 1H), 3.21 (q, J = 6.6 Hz, 2H), 2.48 (q, J = 4.9 Hz, 2H), 1.67 – 1.56 (m, 2H), 1.55 – 1.44 (m, 2H), 1.16 (s, 9H). ^{13}C NMR (100 MHz, $CDCl_3$) δ = 202.5, 178.7, 43.5, 39.0, 38.7, 29.1, 27.7, 19.0. HR-MS(ESI) *m/z* calcd for: $C_{10}H_{20}NO_2$ $[M+H]^+$ 186.1489, found 186.1492. All analytical data for compound **63b** were consistent with

the literature[6].

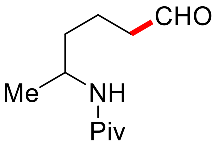

***N*-(6-Oxohexan-2-yl)pivalamide (64b):** The general procedure **B** was followed using substrate **64a** (0.3 mmol, 55 mg). Isolation by column chromatography (PE/EA: 3:1) yielded **64b** (41 mg, 69%) as a colorless liquid. ^{1}H NMR (400 MHz, $CDCl_3$) δ = 9.77 (s, 1H), 5.49 (s, 1H), 4.08 – 3.90 (m, 1H), 2.62 – 2.38 (m, 2H), 1.74 – 1.54 (m, 2H), 1.54 – 1.38 (m, 2H), 1.19 (s, 9H), 1.13 (d, J = 6.6 Hz, 3H). ^{13}C NMR (100 MHz, $CDCl_3$) δ = 202.4, 177.9, 44.5, 43.5, 38.6, 36.2, 27.6, 20.9, 18.4. HR-MS(ESI) *m/z* calcd for: $C_{11}H_{22}NO_2$ [M+H]$^+$ 200.1645, found 200.1648. All analytical data for compound **64b** were consistent with the literature[6].

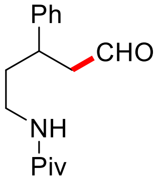

***N*-(5-Oxo-3-phenylpentyl)pivalamide (65b):** The general procedure **B** was followed using substrate **65a** (0.3 mmol, 74 mg). Isolation by column chromatography (PE/EA: 3:1) yielded **65b** (44 mg, 57%) as a colorless liquid. ^{1}H NMR (400 MHz, $CDCl_3$) δ = 9.67 (s, 1H), 7.36 – 7.29 (m, 2H), 7.26 – 7.17 (m, 3H), 5.66 (s, 1H), 3.30 – 3.16 (m, 2H), 3.12 – 3.00 (m, 1H), 2.78 (d, J = 7.1 Hz, 2H), 1.96 – 1.76 (m, 2H), 1.11 (s, 9H). ^{13}C NMR (100 MHz, $CDCl_3$) δ = 201.5, 178.5, 143.2, 129.1, 127.5, 127.1, 50.6, 38.6, 38.0, 37.9, 36.1, 27.6. HR-MS(ESI) *m/z* calcd for: $C_{16}H_{24}NO_2$ [M+H]$^+$ 262.1802, found 262.1803. All analytical data for compound **65b** were consistent with the literature[6].

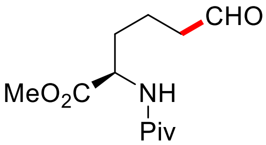

Methyl (*R*)-6-oxo-2-pivalamidohexanoate (66b): The general procedure **B** was followed using substrate **66a** (0.3 mmol, 68 mg). Isolation by column chromatography (PE/EA: 3:1) yielded **66b** (39 mg, 53%) as a colorless liquid. ^{1}H NMR (400 MHz, $CDCl_3$) δ = 9.73 (s, 1H), 6.26 (d, J = 7.6 Hz, 1H), 4.56 (q, J = 6.8 Hz, 1H), 3.73 (s, 3H),

2.49 (q, J = 6.4 Hz, 2H), 1.93 – 1.78 (m, 1H), 1.74 – 1.54 (m, 3H), 1.20 (s, 9H). ^{13}C NMR (100 MHz, $CDCl_3$) δ = 201.9, 178.6, 173.1, 52.6, 51.7, 43.2, 38.8, 31.8, 27.5, 17.7. HR-MS(ESI) *m/z* calcd for: $C_{12}H_{22}NO_4$ $[M+H]^+$ 244.1544, found 244.1544. $[\alpha]_D^{27}$ = -19.0° (c = 0.1, CH_2Cl_2).

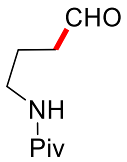

***N*-(4-Oxobutyl)pivalamide (67b):** The general procedure **B** was followed using substrate **67a** (0.3 mmol, 47 mg). Isolation by column chromatography (PE/EA: 3:1) yielded **67b** (32 mg, 62%) as a colorless liquid. ^{1}H NMR (400 MHz, $CDCl_3$) δ = 9.76 (s, 1H), 5.93 (s, 1H), 3.24 (q, J = 6.6 Hz, 2H), 2.51 (t, J = 7.0 Hz, 2H), 1.87 – 1.77 (m, 2H), 1.16 (s, 9H). ^{13}C NMR (100 MHz, $CDCl_3$) δ = 202.3, 178.9, 41.7, 39.1, 38.7, 27.6, 21.9. HR-MS(ESI) *m/z* calcd for: $C_9H_{18}NO_2$ $[M+H]^+$ 172.1332, found 172.1334. All analytical data for compound **67b** were consistent with the literature[6].

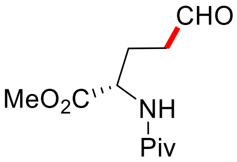

Methyl (*S*)-5-oxo-2-pivalamidopentanoate (68b): The general procedure **B** was followed using substrate **68a** (0.3 mmol, 64 mg). Isolation by column chromatography (PE/EA: 3:1) yielded **68b** (33 mg, 48%) as a colorless liquid. ^{1}H NMR (400 MHz, $CDCl_3$) δ = 9.74 (s, 1H), 6.37 (d, J = 7.5 Hz, 1H), 4.59 – 4.50 (m, 1H), 3.74 (s, 3H), 2.67 – 2.44 (m, 2H), 2.29 – 2.12 (m, 1H), 2.05 – 1.90 (m, 1H), 1.19 (s, 9H). ^{13}C NMR (100 MHz, $CDCl_3$) δ = 201.3, 178.8, 172.7, 52.7, 51.7, 40.2, 38.8, 27.5, 24.7. HR-MS(ESI) *m/z* calcd for: $C_{11}H_{20}NO_4$ $[M+H]^+$ 230.1387, found 230.1387. $[\alpha]_D^{27}$ = 14.0° (c = 0.1, CH_2Cl_2).

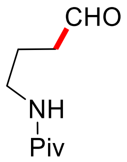

***N*-(4-Oxobutyl)pivalamide (67b):** The general procedure **B** was followed using substrate **69a** (0.3 mmol, 60 mg). Isolation by column chromatography (PE/EA: 3:1) yielded **67b** (25 mg, 49%) as a colorless liquid. ^{1}H NMR (400 MHz, $CDCl_3$) δ = 9.77

(s, 1H), 5.92 (s, 1H), 3.25 (q, J = 6.5 Hz, 2H), 2.52 (t, J = 6.9 Hz, 2H), 1.88 – 1.77 (m, 2H), 1.16 (s, 9H). ^{13}C NMR (100 MHz, $CDCl_3$) δ = 202.4, 178.9, 41.7, 39.2, 38.7, 27.6, 21.9. HR-MS(ESI) *m/z* calcd for: $C_9H_{18}NO_2$ $[M+H]^+$ 172.1332, found 172.1332. All analytical data for compound **67b** were consistent with the literature[6].

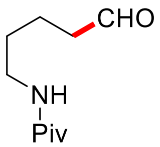

***N*-(5-Oxopentyl)pivalamide (63b):** The general procedure **B** was followed using substrate **70a** (0.3 mmol, 64 mg). Isolation by column chromatography (PE/EA: 4:1) yielded **63b** (42 mg, 75%) as a colorless liquid. ^{1}H NMR (400 MHz, $CDCl_3$) δ = 9.75 (s, 1H), 5.84 (s, 1H), 3.22 (q, J = 6.1 Hz, 2H), 2.48 (q, J = 4.6 Hz, 2H), 1.67 – 1.56 (m, 2H), 1.56 – 1.45 (m, 2H), 1.17 (s, 9H). ^{13}C NMR (100 MHz, $CDCl_3$) δ = 202.6, 178.8, 43.5, 39.0, 38.7, 29.1, 27.7, 19.0. HR-MS(ESI) *m/z* calcd for: $C_{10}H_{20}NO_2$ $[M+H]^+$ 200.1645, found 200.1646. All analytical data for compound **63b** were consistent with the literature[6].

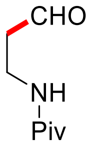

***N*-(3-Oxopropyl)pivalamide (71b):** The general procedure **B** was followed using substrate **71a** (0.3 mmol, 42 mg). Isolation by column chromatography (PE/EA: 3:1) yielded **71b** (10 mg, 21%) as a colorless liquid. ^{1}H NMR (400 MHz, $CDCl_3$) δ = 9.80 (s, 1H), 6.16 (s, 1H), 3.52 (q, J = 5.9 Hz, 2H), 2.73 (t, J = 5.7 Hz, 2H), 1.15 (s, 9H). ^{13}C NMR (100 MHz, $CDCl_3$) δ = 201.9, 178.7, 43.9, 38.7, 33.1, 27.6. HR-MS(ESI) *m/z* calcd for: $C_8H_{16}NO_2$ $[M+H]^+$ 158.1176, found 158.1175.

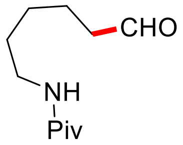

***N*-(6-Oxohexyl)pivalamide (72b):** The general procedure **B** was followed using substrate **72a** (0.3 mmol, 55 mg). Isolation by column chromatography (PE/EA: 3:1) yielded **72b** (45 mg, 75%) as a colorless liquid. ^{1}H NMR (400 MHz, $CDCl_3$) δ = 9.73 (s, 1H), 5.75 (s, 1H), 3.20 (q, J = 6.7 Hz, 2H), 2.42 (td, J = 7.2, 1.7 Hz, 2H), 1.67 – 1.57

(m, 2H), 1.54 – 1.43 (m, 2H), 1.38 – 1.26 (m, 2H), 1.15 (s, 9H). ^{13}C NMR (100 MHz, $CDCl_3$) δ = 202.7, 178.6, 43.8, 39.2, 38.7, 29.4, 27.7, 26.3, 21.6. HR-MS(ESI) *m/z* calcd for: $C_{11}H_{22}NO_2$ $[M+H]^+$ 200.1645, found 200.1646. All analytical data for compound **72b** were consistent with the literature[6].

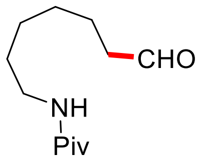

***N*-(7-Oxoheptyl)pivalamide (73b):** The general procedure **B** was followed using substrate **73a** (0.3 mmol, 59 mg). Isolation by column chromatography (PE/EA: 3:1) yielded **73b** (47 mg, 73%) as a colorless liquid. ^{1}H NMR (400 MHz, $CDCl_3$) δ = 9.74 (s, 1H), 5.67 (s, 1H), 3.20 (q, J = 6.8 Hz, 2H), 2.41 (t, J = 7.4 Hz, 2H), 1.66 – 1.55 (m, 2H), 1.53 – 1.42 (m, 2H), 1.38 – 1.27 (m, 4H), 1.16 (s, 9H). ^{13}C NMR (100 MHz, $CDCl_3$) δ = 202.9, 178.5, 43.9, 39.5, 38.7, 29.5, 28.8, 27.7, 26.7, 22.0. HR-MS(ESI) *m/z* calcd for: $C_{12}H_{24}NO_2$ $[M+H]^+$ 214.1802, found 214.1804. All analytical data for compound **73b** were consistent with the literature[6].

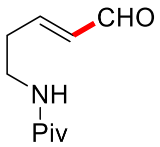

(*E*)-*N*-(5-Oxopent-3-en-1-yl)pivalamide (74b): The general procedure **B** was followed using substrate **74a** (0.3 mmol, 60 mg). Isolation by column chromatography (PE/EA: 3:1) yielded **74b** (30 mg, 55%) as a colorless liquid. ^{1}H NMR (400 MHz, $CDCl_3$) δ = 9.48 (d, J = 7.7 Hz, 1H), 6.79 (dt, J = 14.3, 6.8 Hz, 1H), 6.10 (dd, J = 15.7, 7.8 Hz, 1H), 5.91 (s, 1H), 3.42 (q, J = 6.3 Hz, 2H), 2.55 (q, J = 6.9 Hz, 2H), 1.15 (s, 9H). ^{13}C NMR (100 MHz, $CDCl_3$) δ = 193.9, 178.9, 155.0, 134.4, 38.8, 37.6, 33.3, 27.6. HR-MS(ESI) *m/z* calcd for: $C_{10}H_{18}NO_2$ $[M+H]^+$ 184.1332, found 184.1336.

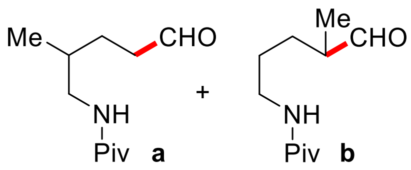

***N*-(2-methyl-5-oxopentyl)pivalamide/(*R*)-*N*-(4-methyl-5-oxopentyl)pivalamide (a:b = 3:2) (75b):** The general procedure **B** was followed using substrate **75a** (0.3 mmol,

55 mg). Isolation by column chromatography (PE/EA: 3:1) yielded **75b** (44 mg, 74%) as a colorless liquid. ^{1}H NMR (400 MHz, $CDCl_3$) δ = 9.76 (s, 1H), 9.59 (s, 0.6H), 5.92 (s, 1H), 5.81 (s, 0.6H), 3.28 – 3.19 (m, 1.3H), 3.18 – 3.00 (m, 2H), 2.51 (q, J = 7.2 Hz, 2H), 2.42 – 2.30 (m, 1H), 1.76 – 1.64 (m, 2H), 1.64 – 1.58 (m, 0.7H), 1.57 – 1.46 (m, 1.3H), 1.45 – 1.36 (m, 1.3H), 1.18 (s, 6H), 1.17 (s, 9H), 1.09 (d, J = 7.2 Hz, 2H), 0.88 (d, J = 6.1 Hz, 3H). ^{13}C NMR (100 MHz, $CDCl_3$) δ = 205.1, 202.7, 178.84, 178.75, 46.0, 44.7, 41.4, 39.4, 38.9, 38.8, 33.0, 27.71, 27.68, 27.4, 27.1, 25.8, 17.6, 13.5. HR-MS(ESI) *m/z* calcd for: $C_{11}H_{22}NO_2$ $[M+H]^+$ 200.1645, found 200.1645.

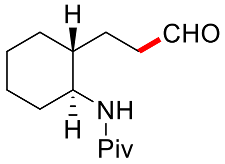

***N*-((1*S*,2*R*)-2-(3-oxopropyl)cyclohexyl)pivalamide** (**76b**)**:** The general procedure **B** was followed using substrate **76a** (0.3 mmol, 67 mg). Isolation by column chromatography (PE/EA: 5:1) yielded **76b** (34 mg, 47%) as a white solid. M.p. = 114–115 °C. ^{1}H NMR (400 MHz, $CDCl_3$) δ = 9.74 (s, 1H), 5.66 (d, J = 8.7 Hz, 1H), 3.55 (qd, J = 10.8, 3.8 Hz, 1H), 2.62 – 2.36 (m, 2H), 2.00 – 1.91 (m, 1H), 1.91 – 1.82 (m, 1H), 1.82 – 1.73 (m, 1H), 1.73 – 1.62 (m, 2H), 1.34 – 1.25 (m, 2H), 1.19 (s, 9H), 1.17 – 1.07 (m, 2H), 1.07 – 0.93 (m, 2H). ^{13}C NMR (100 MHz, $CDCl_3$) δ = 203.0, 178.1, 51.8, 42.5, 40.9, 38.8, 33.8, 31.1, 27.7, 25.7, 25.3, 24.3. HR-MS(ESI) *m/z* calcd for: $C_{14}H_{26}NO_2$ $[M+H]^+$ 240.1958, found 240.1959.

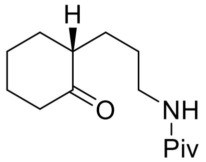

(*R*)-*N*-(3-(2-oxocyclohexyl)propyl)pivalamide (**76b'**)**:** The general procedure **B** was followed using substrate **76a** (0.3 mmol, 60 mg). Isolation by column chromatography (PE/EA: 3:1) yielded **76b'** (27 mg, 38%) as a colorless liquid. ^{1}H NMR (400 MHz, $CDCl_3$) δ = 5.97 (s, 1H), 3.29 – 3.06 (m, 2H), 2.42 – 2.20 (m, 3H), 2.11 – 1.99 (m, 2H), 1.89 – 1.58 (m, 4H), 1.55 – 1.30 (m, 3H), 1.24 – 1.21 (m, 1H), 1.18 (s, 9H). ^{13}C NMR (100 MHz, $CDCl_3$) δ = 213.7, 178.7, 50.5, 42.3, 39.5, 38.7, 34.3, 28.2, 27.7, 27.2, 26.5,

25.2. HR-MS(ESI) *m/z* calcd for: $C_{14}H_{26}NO_2$ $[M+H]^+$ 240.1958, found 240.1960.

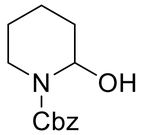

Benzyl 2-hydroxypiperidine-1-carboxylate (78): The general procedure **B** was followed using substrate benzyl piperidine-1-carboxylate (0.3 mmol, 66 mg). Isolation by column chromatography (PE/EA: 5:1) yielded **78** (32 mg, 45%) as a colorless liquid. ^{1}H NMR (500 MHz, DMSO-d_6) δ = 5.97 (s, 1H), 3.29 – 3.06 (m, 2H), 2.42 – 2.20 (m, 3H), 2.11 – 1.99 (m, 2H), 1.89 – 1.58 (m, 4H), 1.55 – 1.30 (m, 3H), 1.24 – 1.21 (m, 1H), 1.18 (s, 9H). ^{13}C NMR (125 MHz, DMSO-d_6) δ = 213.7, 178.7, 50.5, 42.3, 39.5, 38.7, 34.3, 28.2, 27.7, 27.2, 26.5, 25.2. HR-MS(ESI) *m/z* calcd for: $C_{14}H_{26}NO_2$ $[M+H]^+$ 240.1958, found 240.1960.

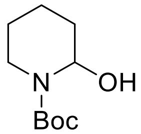

***Tert*-butyl 2-hydroxypiperidine-1-carboxylate (79):** The general procedure **B** was followed using substrate *tert*-butyl piperidine-1-carboxylate (0.3 mmol, 56 mg). Isolation by column chromatography (PE/EA: 10:1) yielded **79** (18 mg, 30%) as a colorless liquid. ^{1}H NMR (400 MHz, DMSO-d_6) δ = 5.53 (s, 1H), 5.44 (d, J = 3.2 Hz, 1H), 3.66 (d, J = 12.7 Hz, 1H), 2.94 (t, J = 12.0 Hz, 1H), 1.79 – 1.52 (m, 4H), 1.48 – 1.42 (m, 2H), 1.40 (s, 9H). ^{13}C NMR (100 MHz, DMSO-d_6) δ = 153.5, 78.4, 72.9, 37.9, 31.1, 27.9, 24.7, 17.4. HR-MS(ESI) *m/z* calcd for $C_{10}H_{19}NO_3Na^+$ $[M+Na]^+$ 224.1257, found 224.1254.

3.3 Gram-Scale Synthesis and Diversifications of Peptide Aldehyde

3.3.1 Gram-Scale Synthesis

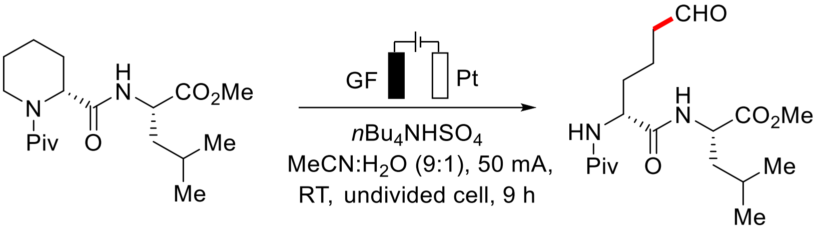

1a (5.0 mmol) **1b**: 72% (**1.285 g**)

In an undivided cell (250 mL) equipped with a stirring bar, a mixture of substrate **1a** (5.0 mmol, 1.700 g), *n*Bu_4NHSO_4 (5.0 mmol, 1.698 g) and MeCN/H_2O (9:1, 150 mL) were added. The cell was equipped with graphite felt plate (3 cm × 3 cm × 0.2 cm) as the anode and platinum plate as the cathode and connected to an AXIOMET AX-3003P DC regulated power supply. The reaction mixture was stirred and electrolyzed at a constant current of 50 mA at room temperature for 9 h. Upon completion, the solvent was further removed directly under reduced pressure to afford the crude product, which was purified by flash column chromatography (PE/EA: 2:1) to afford **1b** (1.285 g, 72%) as a white solid. M.p. = 110–111 °C. The gram scale reactor and the synthetic product **1b** were displayed in **Supplementary Figure 2**.

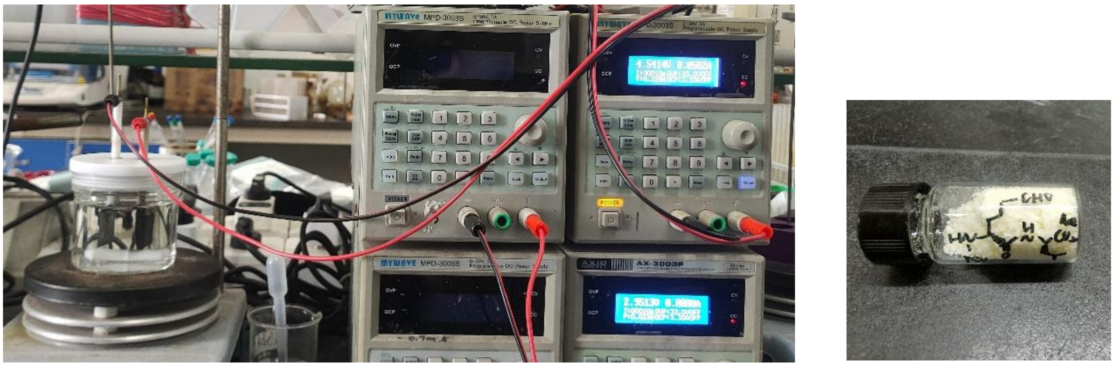

Supplementary Figure 2. Gram scale reactor and synthetic product **1b** of gram scale reaction.

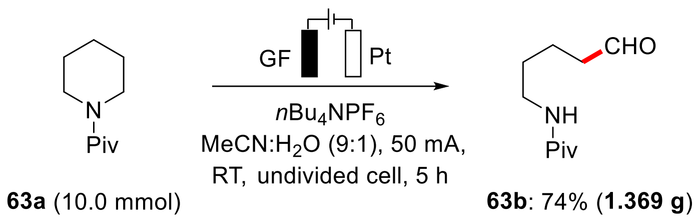

In an undivided cell (250 mL) equipped with a stirring bar, a mixture of substrate **63a** (10.0 mmol, 1.690 g), *n*Bu$_4$NPF$_6$ (10.0 mmol, 3.874 g) and MeCN/H_2O (9:1, 150 mL) were added. The cell was equipped with graphite felt plate (3 cm × 3 cm × 0.2 cm) as the anode and platinum plate as the cathode and connected to an AXIOMET AX-3003P DC regulated power supply. The reaction mixture was stirred and electrolyzed at a constant current of 50 mA at room temperature for 5 h. Upon completion, the solvent was further removed directly under reduced pressure to afford the crude product, which was purified by flash column chromatography (PE/EA: 2:1) to afford **63b** (1.369 g, 74%) as a colorless liquid.

3.3.2 Diversifications of Peptide Aldehyde

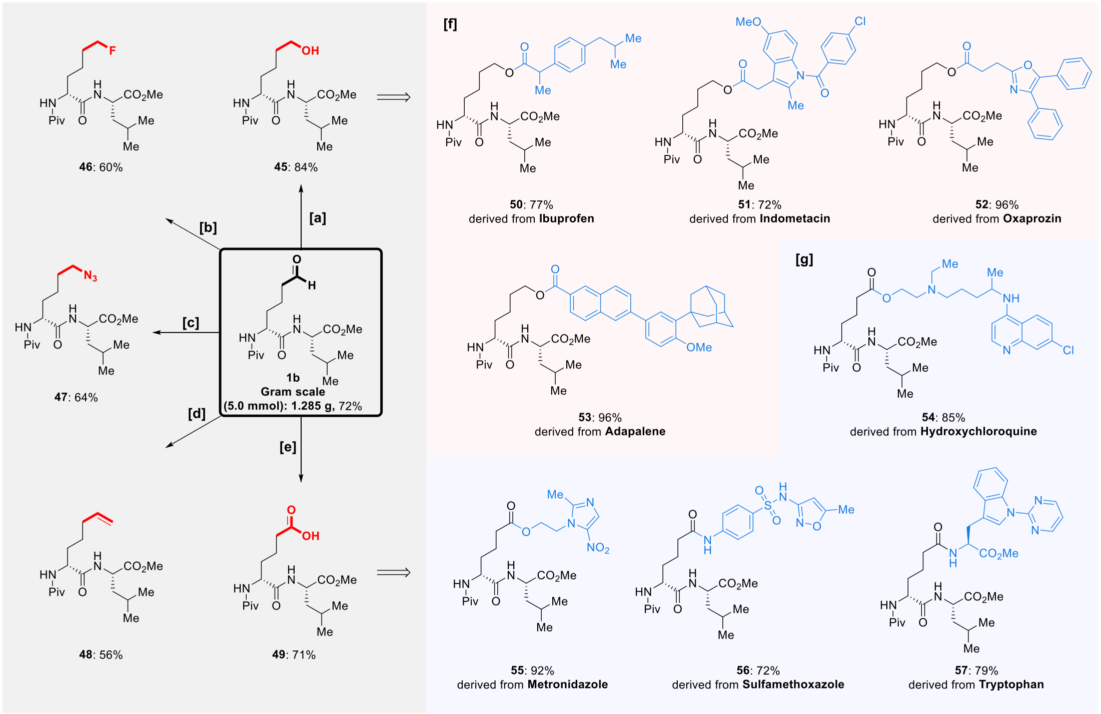

Supplementary Figure 3. Diversifications of peptide aldehyde.

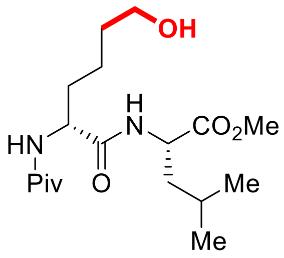

***Procedure for preparation of* 45**[13]: To a solution of compound **1b** (356 mg, 1.0 mmol) in MeOH (5 mL) was added $NaBH_4$ (38 mg, 1.0 mmol). The mixture was stirred at 0 °C for 1 h, MeOH was evaporated and the residue was dissolved in EtOAc (20 mL). The organic layer was washed with water (20 mL × 3) and brine (20 mL × 3), and dried over Na_2SO_4, filtered and concentrated under reduced pressure. The crude residue was purified by column chromatography (PE/EA: 1:1) to afford **45** (301 mg, 84%) as a colorless liquid.

Methyl [(*R*)-6-hydroxy-2-pivalamidohexanoyl]-*L*-leucinate (45): ^{1}H NMR (400 MHz, $CDCl_3$) δ = 7.19 (d, J = 8.3 Hz, 1H), 6.45 (d, J = 7.4 Hz, 1H), 4.63 – 4.46 (m, 2H), 3.69 (s, 3H), 3.61 (t, J = 6.3 Hz, 2H), 1.94 – 1.82 (m, 1H), 1.74 – 1.49 (m, 6H), 1.45 – 1.34 (m, 2H), 1.19 (s, 9H), 0.92 (d, J = 4.1 Hz, 3H), 0.91 (d, J = 4.1 Hz, 3H). ^{13}C NMR (100 MHz, $CDCl_3$) δ = 179.1, 173.3, 172.1, 62.3, 52.6, 52.4, 50.8, 41.2, 38.9, 32.2, 32.1, 27.6, 25.0, 23.0, 21.8. HR-MS(ESI) *m/z* calcd for $C_{18}H_{35}N_2O_5$ $[M+H]^+$ 359.2541, found 359.2541. $[\alpha]_D^{27}$ = 31.0° (c = 0.2, $CHCl_3$).

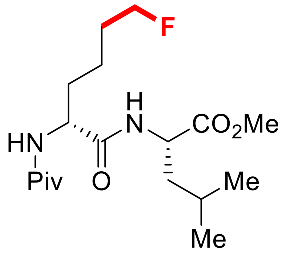

***Procedure for preparation of* 46**[1]: Compound **45** (72 mg, 0.2 mmol) was dissolved in DCM and cooled to -78 °C. DAST (53 μL, 0.4 mmol) was added dropwise to the stirring solution, which was allowed to warm to room temperature overnight. The crude residue was purified by column chromatography (PE/EA: 3:1) to afford **46** (43 mg, 60%) as a white solid.

Methyl [(*R*)-6-fluoro-2-pivalamidohexanoyl]-*L*-leucinate (46): M.p. = 116–117 °C.

^{1}H NMR (400 MHz, $CDCl_3$) δ = 6.99 (d, J = 8.3 Hz, 1H), 6.33 (d, J = 7.7 Hz, 1H), 4.55 (p, J = 7.5 Hz, 2H), 4.50 – 4.43 (m, 1H), 4.39 – 4.32 (m, 1H), 3.69 (s, 3H), 2.03 – 1.85 (m, 2H), 1.74 – 1.56 (m, 5H), 1.50 – 1.37 (m, 2H), 1.20 (s, 9H), 0.93 (d, J = 4.9 Hz, 3H), 0.91 (d, J = 4.9 Hz, 3H). ^{13}C NMR (100 MHz, $CDCl_3$) δ = 178.9, 173.2, 171.8, 83.8 (d, $^1J_{C\text{-}F}$ = 164.7 Hz), 52.5 (d, $^3J_{C\text{-}F}$ = 8.1 Hz), 50.8, 41.2, 38.9, 31.9, 30.1 (d, $^2J_{C\text{-}F}$ = 19.5 Hz), 27.6, 27.5, 25.0, 23.0, 21.7, 21.2 (d, $^4J_{C\text{-}F}$ = 4.8 Hz). ^{19}F NMR (376 MHz) δ = -218.54 (m). HR-MS(ESI) m/z calcd for $C_{18}H_{34}FN_2O_4$ $[M+H]^+$ 361.2497, found 361.2497. $[\alpha]_D^{27}$ = 42.3° (c = 0.2, $CHCl_3$).

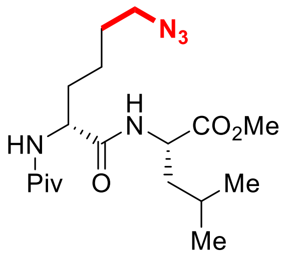

***Procedure for preparation of* 47**[13]: To a solution of compound **45** (179 mg, 0.5 mmol) and Et_3N (0.1 mL, 0.75 mmol) in anhydrous DCM (5 mL) was added dropwise methanesulfonyl chloride (46 μL, 0.6 mmol). The mixture was stirred at 0 °C for 4 h, DCM was evaporated under vacuum. The residue was dissolved in EtOAc (10 mL) and washed with 10% HCl (10 mL × 3), saturated $NaHCO_3$ (10 mL × 3) and brine (10 mL × 3), and then dried over Na_2SO_4, filtered and concentrated under reduced pressure. The crude residue was purified by column chromatography (PE/EA: 1:1) to afford **S-1** (151 mg, 70%) as a colorless liquid.

Methyl {(*R*)-6-[(methylsulfonyl)oxy]-2-pivalamidohexanoyl}-*L*-leucinate (S-1): ^{1}H NMR (400 MHz, $CDCl_3$) δ = 6.87 (d, J = 8.3 Hz, 1H), 6.31 (d, J = 7.8 Hz, 1H), 4.58 – 4.46 (m, 2H), 4.29 – 4.14 (m, 2H), 3.70 (s, 3H), 3.00 (s, 3H), 1.96 – 1.86 (m, 1H), 1.83 – 1.74 (m, 2H), 1.73 – 1.53 (m, 4H), 1.49 – 1.40 (m, 2H), 1.20 (s, 9H), 0.93 (d, J = 4.3 Hz, 3H), 0.92 (d, J = 4.3 Hz, 3H). ^{13}C NMR (100 MHz, $CDCl_3$) δ = 179.1, 173.2, 171.7, 69.7, 52.5, 52.4, 50.8, 41.2, 38.9, 37.5, 31.4, 28.8, 27.6, 25.0, 22.9, 21.8, 21.5. HR-MS(ESI) m/z calcd for $C_{19}H_{37}N_2O_7S$ $[M+H]^+$ 437.2316, found 437.2314.

Compound **S-1** (131 mg, 0.3 mmol) was dissolved in DMF (1 mL) followed by the addition of NaN_3 (29 mg, 0.45 mmol). The mixture was stirred at room temperature for

12 h and then was quenched by being poured into ice-cold water. The mixture was extracted with EtOAc (10 mL × 3). The organic layer was washed with water (10 mL × 3) and brine (10 mL × 3), and dried over Na_2SO_4, filtered and concentrated under reduced pressure. The crude residue was purified by column chromatography (PE/EA: 3:1) to afford **47** (73 mg, 64%) as a white solid.

Methyl *N*-diazo-*N*-pivaloyl-*D*-lysyl-*L*-leucinate (47): M.p. = 112–113 °C. ^{1}H NMR (400 MHz, $CDCl_3$) δ = 6.95 (d, J = 6.8 Hz, 1H), 6.32 (d, J = 7.6 Hz, 1H), 4.60 – 4.49 (m, 2H), 3.70 (s, 3H), 3.26 (td, J = 6.6, 2.8 Hz, 2H), 1.96 – 1.86 (m, 1H), 1.72 – 1.55 (m, 6H), 1.45 – 1.31 (m, 2H), 1.20 (s, 9H), 0.94 (d, J = 4.3 Hz, 3H), 0.92 (d, J = 4.3 Hz, 3H). ^{13}C NMR (100 MHz, $CDCl_3$) δ = 178.9, 173.2, 171.7, 52.5, 51.2, 50.8, 41.3, 38.9, 31.8, 28.7, 27.6, 25.0, 23.0, 22.6, 21.7. HR-MS(ESI) *m/z* calcd for $C_{18}H_{34}N_5O_4$ $[M+H]^+$ 384.2606, found 384.2602. $[\alpha]_D^{27}$ = 39.0° (c = 0.2, $CHCl_3$).

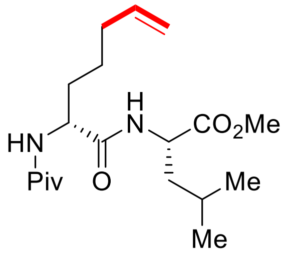

Wittig Reagent Preparation: A flame-dried flask under inert atmosphere was charged with methyltriphenylphosphonium bromide (214 mg, 0.6 mmol) in THF (3 mL) and cooled to 0 °C. Sodium *tert*-butoxide (52 mg, 0.54 mmol) was added in one portion and the reaction allowed to warm to room temperature, stirring for 12h.

***Procedure for preparation of* 48**[14]: Compound **1b** (68 mg, 0.2 mmol) was dissolved in THF (1 mL) and added dropwise to the previously prepared solution of Wittig reagent in THF at 0 °C, stirred overnight and allowed to warm to room temperature. The reaction mixture was purified by flash chromatography (PE/EA: 5/1) to afford **48** (40 mg, 56%) as a white solid.

Methyl [(*R*)-2-pivalamidohept-6-enoyl]-*L*-leucinate (48): M.p. = 119–120 °C. ^{1}H NMR (400 MHz, $CDCl_3$) δ = 6.62 (d, J = 8.0 Hz, 1H), 6.19 (d, J = 8.0 Hz, 1H), 5.83 – 5.69 (m, 1H), 5.05 – 4.91 (m, 2H), 4.60 – 4.51 (m, 1H), 4.44 (q, J = 7.2 Hz, 1H), 3.72 (s, 3H), 2.06 (q, J = 7.2 Hz, 2H), 1.91 – 1.82 (m, 1H), 1.70 – 1.51 (m, 4H), 1.48 – 1.36

(m, 2H), 1.19 (s, 9H), 0.91 (d, $J = 5.5$ Hz, 3H), 0.89 (d, $J = 5.5$ Hz, 3H). ^{13}C NMR (100 MHz, $CDCl_3$) $\delta = 178.7$, 173.2, 171.8, 138.2, 115.2, 52.7, 52.4, 50.9, 41.3, 38.8, 33.4, 31.6, 27.6, 24.9, 24.7, 22.9, 21.9. HR-MS(ESI) *m/z* calcd for $C_{19}H_{35}N_2O_4$ $[M+H]^+$ 355.2592, found 355.2587. $[\alpha]_D^{28} = 44.0°$ ($c = 0.1$, $CHCl_3$).

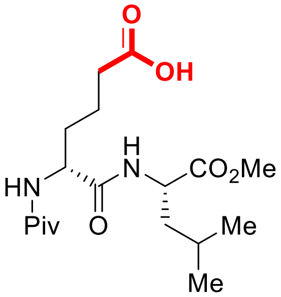

***Procedure for preparation of* 49**[15]: *N*-Hydroxyphthalimide (NHPI) (41 mg, 0.25 mmol) was weighed into a 50 mL Schlenk tube. After dried *in vacuo* for 15 min, 50 mL Schlenk tube was filled with oxygen. Then dry MeCN (20 mL) was added. Compound **1b** (1.780 g, 5.0 mmol) was last added and stirred at room temperature for 12 h. After **1b** completely transformed, the reaction mixture was purified by flash chromatography (PE/EA: 1/1) to afford **49** (1.315 g, 71%) as a white solid.

(*R*)-6-{[(*S*)-1-Methoxy-4-methyl-1-oxopentan-2-yl]amino}-6-oxo-5-pivalamidohexanoic acid (49): M.p. = 118–119 °C. ^{1}H NMR (400 MHz, $CDCl_3$) $\delta = 7.35$ (d, $J = 8.2$ Hz, 1H), 6.94 (d, $J = 8.1$ Hz, 1H), 4.61 – 4.49 (m, 2H), 3.68 (s, 3H), 2.46 – 2.29 (m, 2H), 1.91 – 1.63 (m, 3H), 1.68 – 1.51 (m, 4H), 1.19 (s, 9H), 0.92 (d, $J = 5.0$ Hz, 3H), 0.90 (d, $J = 5.0$ Hz, 3H). ^{13}C NMR (100 MHz, $CDCl_3$) $\delta = 179.6$, 176.7, 173.2, 172.6, 52.7, 52.5, 50.9, 41.1, 38.9, 33.3, 31.3, 27.5, 24.9, 22.9, 21.8, 20.9. HR-MS(ESI) *m/z* calcd for $C_{18}H_{33}N_2O_6$ $[M+H]^+$ 373.2333, found 373.2330. $[\alpha]_D^{28} = 50.3°$ ($c = 0.1$, $CHCl_3$).

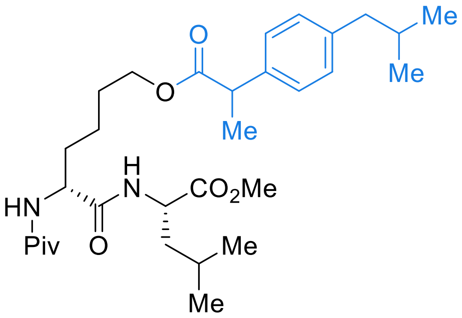

***Procedure for preparation of* 50**: A 25 mL round-bottomed flask was charged with a

solution of compound **45** (72 mg, 0.2 mmol), Ibuprofen (50 mg, 0.24 mmol) and DMAP (5 mg, 0.04 mmol) in DCM (1 mL) and cooled to 0 °C. To this solution was added EDCI (58 mg, 0.3 mmol) and the resulting mixture was warmed to room temperature. After 12 h, the reaction mixture was cooled to 0 °C and quenched with 1M HCl aq. (10 mL). The phases were separated and the aqueous phase was extracted with DCM (10 mL × 3). The combined organic layers were washed with brine (10 mL), dried over Na_2SO_4, filtered and concentrated under reduced pressure. The crude residue was purified by column chromatography (PE/EA: 5:1) to afford **50** (84 mg, 77%) as a colorless liquid.

Methyl {(2*R*)-6-{[2-(4-isobutylphenyl)propanoyl]oxy}-2-pivalamidohexanoyl}-*L*-leucinate (**50**)**:** ^{1}H NMR (400 MHz, $CDCl_3$) δ = 7.17 (dd, J = 8.0, 1.4 Hz, 2H), 7.08 (dd, J = 8.0 Hz, 1.4 Hz, 2H), 6.77 (d, J = 7.7 Hz, 1H), 6.22 (d, J = 7.8 Hz, 1H), 4.59 – 4.50 (m, 1H), 4.48 – 4.40 (m, 1H), 4.12 – 3.94 (m, 2H), 3.70 (s, 3H), 3.65 (q, J = 7.4 Hz, 1H), 2.43 (d, J = 7.1 Hz, 2H), 1.94 – 1.83 (m, 2H), 1.68 – 1.50 (m, 6H), 1.46 (d, J = 7.0 Hz, 3H), 1.37 – 1.26 (m, 2H), 1.20 (s, 9H), 0.93 (d, J = 4.0 Hz, 3H), 0.91 (d, J = 4.0 Hz, 3H), 0.88 (d, J = 6.7 Hz, 6H). ^{13}C NMR (100 MHz, $CDCl_3$) δ = 179.0, 174.9, 173.1, 171.7, 140.6, 137.8, 129.4, 127.2, 64.4, 52.5, 52.4, 50.8, 45.3, 45.1, 41.3, 38.9, 31.6, 30.3, 28.4, 27.6, 25.0, 22.9, 22.5, 22.0, 21.8, 18.7, 14.3. HR-MS(ESI) *m/z* calcd for $C_{31}H_{51}N_2O_6$ $[M+H]^+$ 547.3742, found 547.3741. $[\alpha]_D^{27}$ = 29.7° (*c* = 0.2, $CHCl_3$).

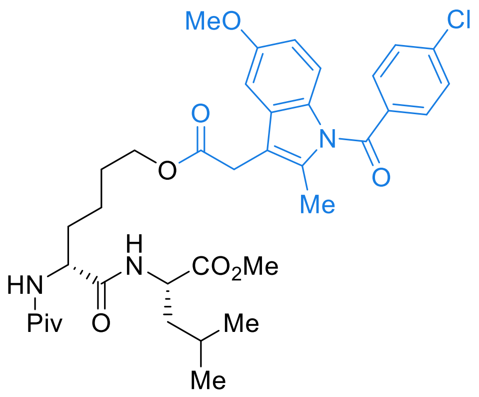

Procedure for preparation of **51**: A 25 mL round-bottomed flask was charged with a solution of compound **45** (72 mg, 0.2 mmol), Indometacin (86 mg, 0.24 mmol) and DMAP (5 mg, 0.04 mmol) in DCM (1 mL) and cooled to 0 °C. To this solution was added EDCI (58 mg, 0.3 mmol) and the resulting mixture was warmed to room

temperature. After 12 h, the reaction mixture was cooled to 0 °C and quenched with 1M HCl aq. (10 mL). The phases were separated and the aqueous phase was extracted with DCM (10 mL × 3). The combined organic layers were washed with brine (10 mL), dried over Na_2SO_4, filtered and concentrated under reduced pressure. The crude residue was purified by column chromatography (PE/EA: 3:1) to afford **51** (100 mg, 72%) as a yellow liquid.

Methyl {(*R*)-6-{2-[1-(4-chlorobenzoyl)-5-methoxy-2-methyl-1H-indol-3-yl]acetoxy}-2-pivalamidohexanoyl}-*L*-leucinate (**51**)**:** ^{1}H NMR (400 MHz, $CDCl_3$) δ = 7.66 (d, J = 8.6 Hz, 2H), 7.47 (d, J = 8.6 Hz, 2H), 6.95 (d, J = 2.6 Hz, 1H), 6.87 (d, J = 9.0 Hz, 1H), 6.76 (d, J = 8.1 Hz, 1H), 6.67 (dd, J = 9.1, 2.7 Hz, 1H), 6.22 (d, J = 7.9 Hz, 1H), 4.58 – 4.50 (m, 1H), 4.42 (q, J = 7.6 Hz, 1H), 4.07 (t, J = 6.5 Hz, 2H), 3.83 (s, 3H), 3.70 (s, 3H), 3.64 (s, 2H), 2.37 (s, 3H), 1.92 – 1.79 (m, 2H), 1.68 – 1.57 (m, 5H), 1.34 (p, J = 7.7 Hz, 2H), 1.20 (s, 9H), 0.91 (d, J = 5.7 Hz, 6H). ^{13}C NMR (100 MHz, $CDCl_3$) δ = 179.0, 173.1, 171.7, 171.0, 168.5, 156.0, 139.4, 136.1, 134.0, 131.3, 131.0, 130.8, 129.3, 115.1, 112.7, 111.5, 101.7, 64.8, 55.9, 52.6, 52.4, 50.8, 41.3, 38.9, 31.7, 30.5, 28.5, 27.6, 25.0, 22.9, 22.2, 21.9, 13.5. HR-MS(ESI) *m/z* calcd for $C_{37}H_{49}ClN_3O_8$ $[M+H]^+$ 698.3203, found 698.3199. $[\alpha]_D^{27}$ = 18.5° (c = 0.2, $CHCl_3$).

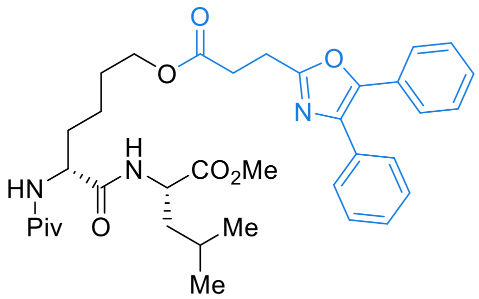

***Procedure for preparation of* 52**: A 25 mL round-bottomed flask was charged with a solution of compound **45** (72 mg, 0.2 mmol), Oxaprozin (70 mg, 0.24 mmol) and DMAP (5 mg, 0.04 mmol) in DCM (1 mL) and cooled to 0 °C. To this solution was added EDCI (58 mg, 0.3 mmol) and the resulting mixture was warmed to room temperature. After 12 h, the reaction mixture was cooled to 0 °C and quenched with 1M HCl aq. (10 mL). The phases were separated and the aqueous phase was extracted with DCM (10 mL × 3). The combined organic layers were washed with brine (10 mL), dried

over Na_2SO_4, filtered and concentrated under reduced pressure. The crude residue was purified by column chromatography (PE/EA: 2:1) to afford **52** (122 mg, 96%) as a colorless liquid.

Methyl {(*R*)-6-{[3-(4,5-diphenyloxazol-2-yl)propanoyl]oxy}-2-pivalamidohexanoyl}-*L*-leucinate (52): ^{1}H NMR (400 MHz, $CDCl_3$) δ = 7.61 (d, J = 7.2 Hz, 2H), 7.55 (d, J = 7.2 Hz, 2H), 7.38 – 7.27 (m, 6H), 7.05 (d, J = 8.3 Hz, 1H), 6.34 (d, J = 7.8 Hz, 1H), 4.61 – 4.47 (m, 2H), 4.09 (t, J = 6.6 Hz, 2H), 3.68 (s, 3H), 3.15 (t, J = 7.6 Hz, 2H), 2.88 (t, J = 7.6 Hz, 2H), 1.96 – 1.84 (m, 1H), 1.73 – 1.51 (m, 6H), 1.44 – 1.32 (m, 2H), 1.19 (s, 9H), 0.92 (d, J = 4.0 Hz, 3H), 0.91 (d, J = 4.0 Hz, 3H). ^{13}C NMR (100 MHz, $CDCl_3$) δ = 178.8, 173.1, 172.1, 171.7, 161.8, 145.5, 135.1, 132.5, 129.0, 128.7, 128.6, 128.5, 128.1, 127.9, 126.5, 64.5, 52.5, 52.3, 50.8, 41.2, 38.8, 31.9, 31.1, 28.4, 27.5, 24.9, 23.5, 22.9, 21.8, 21.7. HR-MS(ESI) m/z calcd for $C_{36}H_{48}N_3O_7$ $[M+H]^+$ 634.3487, found 634.3488. $[\alpha]_D^{28}$ = 24.0° (c = 0.1, $CHCl_3$).

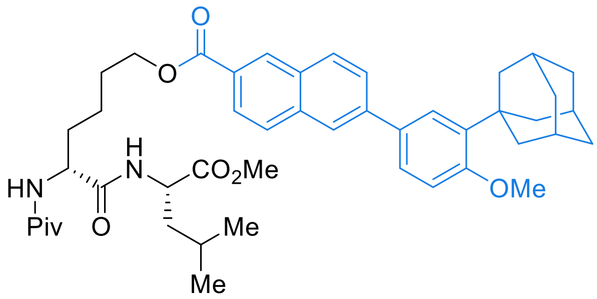

***Procedure for preparation of* 53**: A 25 mL round-bottomed flask was charged with a solution of compound **45** (72 mg, 0.2 mmol), Adapalene (99 mg, 0.24 mmol) and DMAP (5 mg, 0.04 mmol) in DCM (1 mL) and cooled to 0 °C. To this solution was added EDCI (58 mg, 0.3 mmol) and the resulting mixture was warmed to room temperature. After 12 h, the reaction mixture was cooled to 0 °C and quenched with 1M HCl aq. (10 mL). The phases were separated and the aqueous phase was extracted with DCM (10 mL × 3). The combined organic layers were washed with brine (10 mL), dried over Na_2SO_4, filtered and concentrated under reduced pressure. The crude residue was purified by column chromatography (PE/EA: 3:1) to afford **53** (144 mg, 96%) as a colorless liquid.

(*R*)-6-{[(*S*)-1-Methoxy-4-methyl-1-oxopentan-2-yl]amino}-6-oxo-5-pivalamidohe-

xyl 6-{3-[(*3r*,*5r*,*7r*)-adamantan-1-yl]-4-methoxyphenyl}-2-naphthoate (53): ^{1}H NMR (400 MHz, $CDCl_3$) δ = 8.57 (s, 1H), 8.03 (d, J = 8.6 Hz, 1H), 7.99 (s, 1H), 7.97 (d, J = 8.7 Hz, 1H), 7.88 (d, J = 8.6 Hz, 1H), 7.78 (d, J = 8.5 Hz, 1H), 7.60 (s, 1H), 7.52 (d, J = 8.4 Hz, 1H), 7.14 (d, J = 8.3 Hz, 1H), 6.97 (d, J = 8.5 Hz, 1H), 6.43 (d, J = 7.7 Hz, 1H), 4.68 – 4.53 (m, 2H), 4.44 – 4.31 (m, 2H), 3.89 (s, 3H), 3.70 (s, 3H), 2.18 (s, 6H), 2.10 (s, 3H), 2.06 – 1.97 (m, 1H), 1.92 – 1.75 (m, 9H), 1.72 – 1.48 (m, 5H), 1.21 (s, 9H), 0.93 (d, J = 3.2 Hz, 3H), 0.91 (d, J = 3.2 Hz, 3H). ^{13}C NMR (100 MHz, $CDCl_3$) δ = 178.9, 173.2, 171.8, 166.9, 159.0, 141.4, 139.0, 136.0, 132.6, 131.3, 130.8, 129.8, 128.3, 127.1, 126.5, 126.0, 125.8, 125.6, 124.8, 112.2, 64.8, 55.2, 52.6, 52.3, 50.8, 41.2, 40.7, 38.8, 37.3, 37.2, 32.1, 29.2, 28.7, 27.5, 25.0, 22.9, 22.0, 21.7. HR-MS(ESI) *m/z* calcd for $C_{46}H_{61}N_2O_7$ $[M+H]^+$ 753.4474, found 753.4474. $[\alpha]_D^{28}$ = 20.3° (*c* = 0.2, $CHCl_3$).

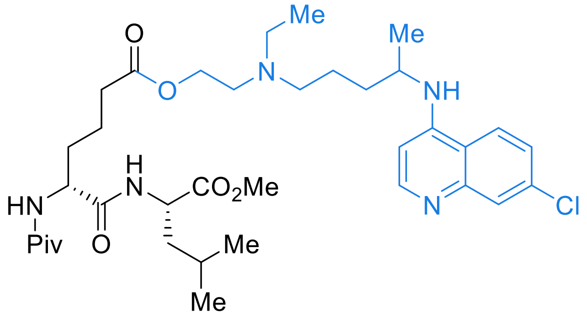

***Procedure for preparation of* 54**: A 25 mL round-bottomed flask was charged with a solution of **49** (74 mg, 0.2 mmol), Hydroxychloroquine (81 mg, 0.24 mmol) and DMAP (5 mg, 0.04 mmol) in DCM (1 mL) and cooled to 0 °C. To this solution was added EDCI (58 mg, 0.3 mmol) and the resulting mixture was warmed to room temperature. After 12 h, the reaction mixture was cooled to 0 °C and quenched with 1M HCl aq. (10 mL). The phases were separated and the aqueous phase was extracted with DCM (10 mL × 3). The combined organic layers were washed with brine (10 mL), dried over Na_2SO_4, filtered and concentrated under reduced pressure. The crude residue was purified by column chromatography (DCM/MeOH: 30:1) to afford **54** (123 mg, 85%) as a yellow liquid.

2-{{4-[(7-Chloroquinolin-4-yl)amino]pentyl}(ethyl)amino}ethyl (5*R*)-6-{[(*S*)-1-methoxy-4-methyl-1-oxopentan-2-yl]amino}-6-oxo-5-pivalamidohexanoate (54):

^{1}H NMR (400 MHz, $CDCl_3$) δ = 8.47 (dd, J = 7.8, 5.6 Hz, 1H), 7.93 (dd, J = 4.3, 2.1 Hz, 1H), 7.77 (dd, J = 9.0, 6.0 Hz, 1H), 7.32 (dd, J = 8.9, 2.2 Hz, 1H), 7.14 (d, J = 7.9, 1H), 6.47 – 6.35 (m, 2H), 5.54 (s, 1H), 4.60 – 4.49 (m, 1H), 4.49 – 4.38 (m, 1H), 4.20 – 4.04 (m, 2H), 3.69 (s, 3H), 2.68 (t, J = 6.0 Hz, 2H), 2.56 (q, J = 7.2 Hz, 2H), 2.50 (t, J = 6.7 Hz, 2H), 2.40 – 2.17 (m, 2H), 1.91 – 1.81 (m, 1H), 1.78 – 1.70 (m, 1H), 1.69 – 1.52 (m, 10H), 1.31 (d, J = 6.1 Hz, 3H), 1.20 (s, 9H), 0.99 (t, J = 7.1 Hz, 3H), 0.92 (d, J = 2.2 Hz, 3H), 0.90 (d, J = 2.2 Hz, 3H). ^{13}C NMR (100 MHz, $CDCl_3$) δ = 179.1, 173.5, 173.2, 171.7, 150.4, 150.2, 147.6, 135.6, 127.1, 125.5, 122.1, 117.1, 99.0, 62.4, 53.6, 52.4, 52.3, 51.6, 50.9, 48.7, 48.1, 41.2, 38.9, 34.1, 33.5, 31.5, 27.6, 25.0, 23.9, 22.9, 21.8, 20.6, 20.3, 11.4. HR-MS(ESI) m/z calcd for $C_{36}H_{57}ClN_5O_6$ $[M+H]^+$ 690.3992, found 690.3987. $[\alpha]_D^{28}$ = 25.0° (c = 0.1, $CHCl_3$).

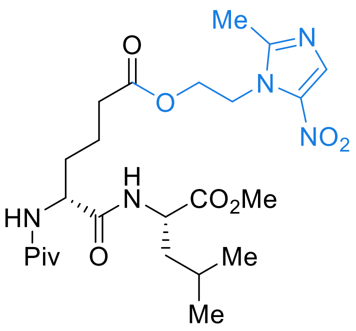

***Procedure for preparation of* 55**: A 25 mL round-bottomed flask was charged with a solution of compound **49** (74 mg, 0.2 mmol), Metronidazole (41 mg, 0.24 mmol) and DMAP (5 mg, 0.04 mmol) in DCM (1 mL) and cooled to 0 °C. To this solution was added EDCI (58 mg, 0.3 mmol) and the resulting mixture was warmed to room temperature. After 12 h, the reaction mixture was cooled to 0 °C and quenched with 1M HCl aq. (10 mL). The phases were separated and the aqueous phase was extracted with DCM (10 mL × 3). The combined organic layers were washed with brine (10 mL), dried over Na_2SO_4, filtered and concentrated under reduced pressure. The crude residue was purified by column chromatography (DCM/MeOH: 50:1) to afford **55** (102 mg, 92%) as a yellow liquid.

2-(2-Methyl-5-nitro-1*H*-imidazol-1-yl)ethyl (*R*)-6-{[(*S*)-1-methoxy-4-methyl-1-oxopentan-2-yl]amino}-6-oxo-5-pivalamidohexanoate (55): ^{1}H NMR (400 MHz, $CDCl_3$) δ = 7.94 (s, 1H), 6.98 (d, J = 8.4 Hz, 1H), 6.33 (d, J = 7.6 Hz, 1H), 4.69 – 4.29

(m, 6H), 3.70 (s, 3H), 2.52 (s, 3H), 2.33 (t, J = 6.3 Hz, 2H), 1.88 – 1.74 (m, 1H), 1.72 – 1.49 (m, 6H), 1.21 (s, 9H), 0.93 (d, J = 4.7 Hz, 3H), 0.91 (d, J = 4.7 Hz, 3H). ^{13}C NMR (100 MHz, $CDCl_3$) δ = 179.2, 173.1, 172.8, 171.5, 150.9, 133.1, 62.5, 52.4, 52.0, 50.9, 45.2, 41.4, 38.9, 33.0, 31.2, 27.6, 25.0, 22.9, 21.9, 20.3, 14.5. (*One ^{13}C signal is overlapping with others*). HR-MS(ESI) *m/z* calcd for $C_{24}H_{40}N_5O_8$ $[M+H]^+$ 526.2872, found 526.2873. $[\alpha]_D^{28}$ = 31.0° (c = 0.2, $CHCl_3$).

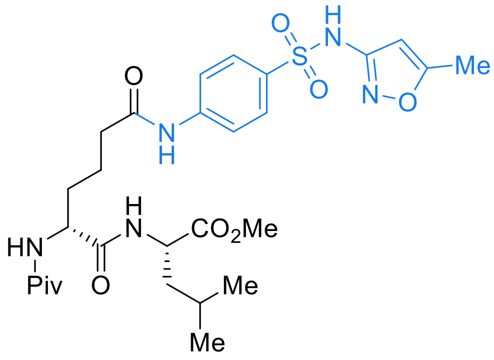

***Procedure for preparation of* 56**: A 25 mL round-bottomed flask was charged with a solution of compound **49** (74 mg, 0.2 mmol), Sulfamethoxazole (101 mg, 0.24 mmol) and HOBt (32 mg, 0.24 mmol) in DCM (2 mL) and cooled to 0 °C. $i$$Pr_2NEt$ (0.1 mL, 0.6 mmol) was added dropwise over 5 min and the resulting mixture was stirred at 0 °C for 10 min. To this solution was added EDCI (58 mg, 0.3 mmol) and the resulting mixture was warmed to room temperature. After 12 h, the reaction mixture was cooled to 0 °C and quenched with 1M HCl aq. (10 mL). The phases were separated and the aqueous phase was extracted with DCM (10 mL × 3). The combined organic layers were washed with brine (10 mL), dried over Na_2SO_4, filtered and concentrated under reduced pressure. The crude residue was purified by column chromatography (DCM/MeOH: 40:1) to afford **56** (92 mg, 72%) as a white solid.

Methyl {(*R*)-6-{{4-[*N*-(5-methylisoxazol-3-yl)sulfamoyl]phenyl}amino}-6-oxo-2-pivalamidohexanoyl}-*L*-leucinate (56): M.p. = 73–74 °C. ^{1}H NMR (400 MHz, $CDCl_3$) δ = 9.99 (s, 1H), 9.40 (s, 1H), 7.69 – 7.56 (m, 5H), 6.84 (d, J = 7.3 Hz, 1H), 6.24 (s, 1H), 4.63 – 4.49 (m, 2H), 3.69 (s, 3H), 2.62 – 2.49 (m, 1H), 2.44 – 2.36 (m, 1H), 2.34 (s, 3H), 1.94 – 1.80 (m, 2H), 1.77 – 1.53 (m, 5H), 1.17 (s, 9H), 0.90 (d, J = 4.6 Hz, 6H). ^{13}C NMR (100 MHz, $CDCl_3$) δ = 179.7, 173.3, 172.8, 172.0, 171.0, 158.1, 143.2, 133.3, 128.3, 119.5, 96.0, 52.5, 51.8, 51.0, 41.1, 38.9, 36.5, 29.8, 27.5, 25.0, 22.9, 21.7, 21.0,

12.8. HR-MS(ESI) *m/z* calcd for $C_{28}H_{42}N_5O_8S$ [M+H]$^+$ 608.2749, found 608.2754. $[\alpha]_D^{28}$ = 31.8° (*c* = 0.2, $CHCl_3$).

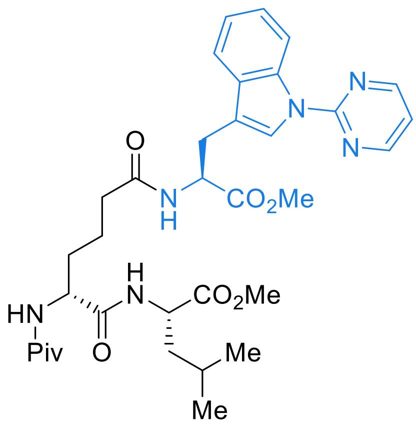

***Procedure for preparation of* 57**: A 25 mL round-bottomed flask was charged with a solution of compound **49** (74 mg, 0.2 mmol), methyl 1-(pyrimidin-2-yl)-*L*-tryptophanate (36 mg, 0.12 mmol) and HOBt (32 mg, 0.24 mmol) in DCM (2 mL) and cooled to 0 °C. *i*Pr_2NEt (0.1 mL, 0.6 mmol) was added dropwise over 5 min and the resulting mixture was stirred at 0 °C for 10 min. To this solution was added EDCI (58 mg, 0.3 mmol) and the resulting mixture was warmed to room temperature. After 12 h, the reaction mixture was cooled to 0 °C and quenched with 1M HCl aq. (10 mL). The phases were separated and the aqueous phase was extracted with DCM (10 mL × 3). The combined organic layers were washed with brine (10 mL), dried over Na_2SO_4, filtered and concentrated under reduced pressure. The crude residue was purified by column chromatography (DCM/MeOH: 50:1) to afford **57** (51 mg, 79%) as a white solid.

Methyl {(*R*)-6-{{(*R*)-1-methoxy-1-oxo-3-[1-(pyrimidin-2-yl)-1*H*-indol-3-yl]propan-2-yl}amino}-6-oxo-2-pivalamidohexanoyl}-*L*-leucinate (57): M.p. = 89–90 °C. ^{1}H NMR (400 MHz, $CDCl_3$) δ = 8.78 (d, J = 8.4 Hz, 1H), 8.67 (d, J = 4.8 Hz, 2H), 8.12 (s, 1H), 7.54 (d, J = 7.7 Hz, 1H), 7.37 – 7.31 (m, 1H), 7.26 – 7.20 (m, 2H), 7.03 (dd, J = 4.8 Hz, 3.9 Hz, 1H), 6.59 (d, J = 7.2 Hz, 1H), 6.56 (d, J = 7.7 Hz, 1H), 4.99 – 4.88 (m, 1H), 4.62 – 4.51 (m, 2H), 3.72 (s, 3H), 3.68 (s, 3H), 3.41 – 3.22 (m, 2H), 2.39 – 2.19 (m, 2H), 1.92 – 1.79 (m, 1H), 1.81 – 1.72 (m, 1H), 1.69 – 1.55 (m, 5H), 1.20 (s, 9H), 0.91 (d, J = 3.7 Hz, 3H), 0.90 (d, J = 3.7 Hz, 3H). ^{13}C NMR (100 MHz, $CDCl_3$) δ =

179.2, 173.2, 173.0, 172.9, 172.0, 158.3, 157.6, 135.6, 131.0, 124.3, 124.2, 122.2, 118.7, 116.5, 116.2, 114.5, 52.8, 52.7, 52.3, 51.7, 50.8, 41.2, 38.9, 34.7, 31.5, 27.5, 27.4, 24.9, 23.0, 21.7, 20.7. HR-MS(ESI) *m/z* calcd for $C_{34}H_{47}N_6O_7$ $[M+H]^+$ 651.3501, found 651.3506. $[\alpha]_D^{28}$ = 53.0° (*c* = 0.2, $CHCl_3$).

3.3.3 Procedure for Preparation of Macrocyclic Peptides 58-61

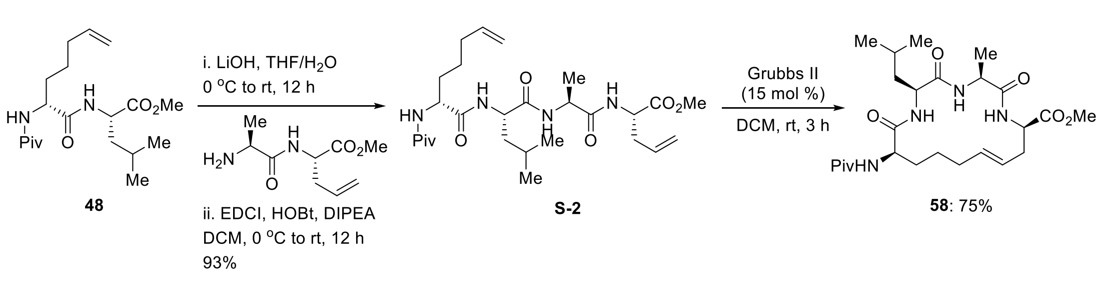

Supplementary Figure 4. Synthesis of cyclic peptide **58**.

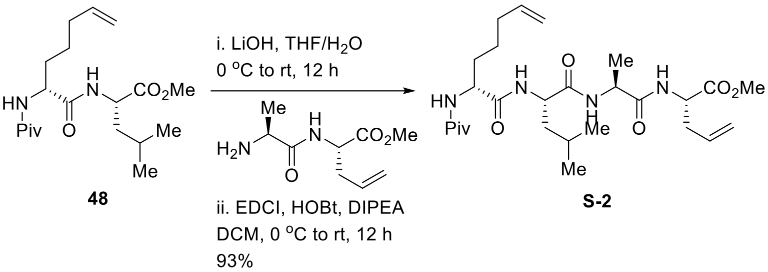

A 25 mL round-bottom flask was charged with a solution of **48** (106 mg, 0.3 mmol) in THF/H_2O (3:1, 4 mL) and cooled to 0 °C. LiOH (36 mg, 1.5 mmol) was added and the resulting mixture was warmed to room temperature. After 12 h, the reaction mixture was cooled to 0 °C and acidified with 1M HCl aq. to pH < 2. The solution was then diluted with EtOAc (10 mL) and the aqueous layer was extracted with EtOAc (10 mL × 3). The combined organic layers were washed with brine (10 mL), dried over Na_2SO_4, filtered and concentrated under reduced pressure to afford crude acid, which was used in the next step without further purification.

A 25 mL round-bottomed flask was charged with a solution of the crude acid (95 mg, 0.28 mmol), methyl (*S*)-2-[(*S*)-2-aminopropanamido]pent-4-enoate (67 mg, 0.336 mmol) and HOBt (45 mg, 0.336 mmol) in DCM (2 mL) and cooled to 0 °C. *i*Pr_2NEt (0.15 mL, 0.84 mmol) was added dropwise over 5 min and the resulting mixture was stirred at 0 °C for 10 min. To this solution was added EDCI (81 mg, 0.42 mmol) and the resulting mixture was warmed to room temperature. After 12 h, the reaction mixture

was cooled to 0 °C and quenched with 1M HCl aq. (10 mL). The phases were separated and the aqueous phase was extracted with DCM (10 mL × 3). The combined organic layers were washed with brine (10 mL), dried over Na_2SO_4, filtered and concentrated under reduced pressure. The crude residue was purified by column chromatography (DCM/MeOH: 50:1) to afford **S-2** (135 mg, 93%) as a white solid.

Methyl (2*S*,5*S*,8*S*,11*R*)-2-allyl-8-isobutyl-5,14,14-trimethyl-4,7,10,13-tetraoxo-11-(pent-4-en-1-yl)-3,6,9,12-tetraazapentadecanoate (**S-2**): ^{1}H NMR (400 MHz, $CDCl_3$) δ = 7.84 (d, J = 7.1 Hz, 1H), 7.70 (d, J = 6.8 Hz, 1H), 7.05 (d, J = 6.9 Hz, 1H), 6.59 (d, J = 7.5 Hz, 1H), 5.77 – 5.62 (m, 2H), 5.14 – 5.02 (m, 2H), 4.98 – 4.84 (m, 2H), 4.83 (q, J = 7.2 Hz, 1H), 4.69 (q, J = 7.0 Hz, 1H), 4.63 – 4.52 (m, 2H), 3.71 (s, 3H), 2.63 – 2.41 (m, 2H), 2.16 – 2.08 (m, 1H), 2.05 – 1.91 (m, 2H), 1.80 – 1.67 (m, 1H), 1.68 – 1.51 (m, 4H), 1.36 – 1.31 (m, 4H), 1.19 (s, 9H), 0.91 (d, J = 5.5 Hz, 3H), 0.89 (d, J = 5.5 Hz, 3H). ^{13}C NMR (100 MHz, $CDCl_3$) δ = 178.6, 172.3, 172.0, 171.8, 171.5, 138.1, 132.5, 119.0, 115.1, 52.8, 52.4, 52.1, 51.8, 48.7, 42.3, 38.8, 36.3, 33.5, 32.5, 27.6, 24.8, 24.5, 23.0, 22.6, 19.1. HR-MS(ESI) *m/z* calcd for $C_{27}H_{47}N_4O_6$ $[M+H]^+$ 523.3490, found 523.3488.

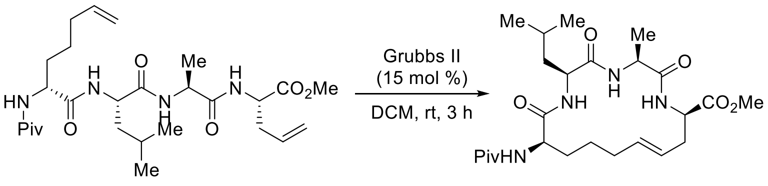

S-2 **58**: 75%

To a solution of **S-2** (52 mg, 0.1 mmol) in DCM (20 mL) was added Grubbs catalyst 2nd Generation[16] (13 mg, 0.015 mmol). The reaction mixture was stirred for 3 h at room temperature and then concentrated onto silica gel for purification by flash chromatography (DCM/MeOH: 50:1) to afford **58** (37 mg, 75%) as a white solid.

Methyl (2*S*,5*S*,8*R*,15*R*,*E*)-2-isobutyl-5-methyl-3,6,16-trioxo-15-pivalamido-1,4,7-triazacyclohexadec-10-ene-8-carboxylate (**58**): M.p. = 296–297 °C. ^{1}H NMR (400 MHz, DMSO-d_6) δ = 8.34 (d, J = 7.7 Hz, 1H), 8.27 (d, J = 8.7 Hz, 1H), 8.11 (d, J = 8.8 Hz, 1H), 6.96 (d, J = 7.4 Hz, 1H), 5.49 (dt, J = 15.5, 5.7 Hz, 1H), 5.30 (dt, J = 15.0, 7.0 Hz, 1H), 4.53 – 4.44 (m, 1H), 4.38 – 4.23 (m, 3H), 3.63 (s, 3H), 2.48 – 2.39 (m, 1H), 2.23 – 2.12 (m, 1H), 1.95 – 1.86 (m, 1H), 1.85 – 1.63 (m, 3H), 1.56 – 1.36 (m, 5H),

1.15 (d, J = 6.9 Hz, 3H), 1.07 (s, 9H), 0.88 (d, J = 6.5 Hz, 3H), 0.84 (d, J = 6.5 Hz, 3H). ^{13}C NMR (100 MHz, DMSO-d_6) δ = 176.6, 171.9, 171.7, 171.3, 171.1, 132.2, 125.0, 52.2, 52.0, 51.7, 50.2, 47.9, 41.0, 37.9, 34.1, 32.1, 31.2, 27.2, 24.2, 24.0, 22.8, 22.0, 17.6. HR-MS(ESI) *m/z* calcd for $C_{25}H_{43}N_4O_6$ $[M+H]^+$ 495.3177, found 495.3174. $[\alpha]_D^{28}$ = 22.3° (c = 0.09, $CHCl_3$).

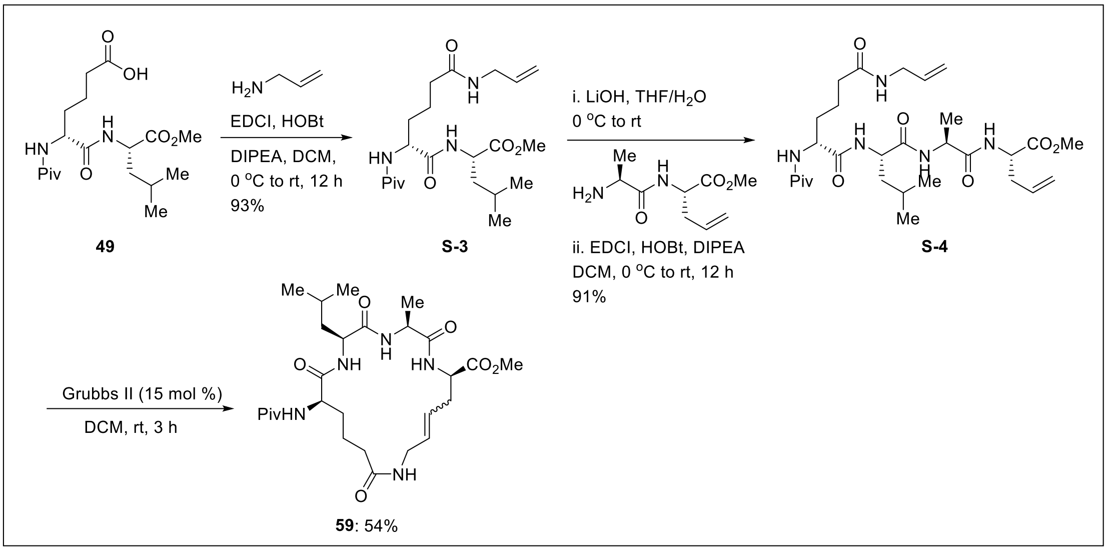

Supplementary Figure 5. Synthesis of cyclic peptide **59.**

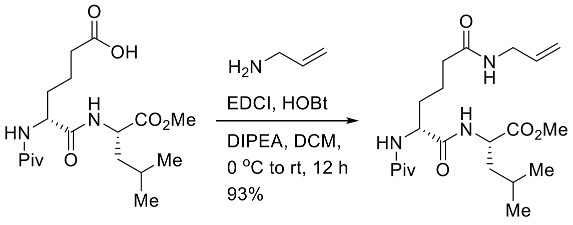

A 25 mL round-bottomed flask was charged with a solution of the **49** (186 mg, 0.5 mmol), prop-2-en-1-amine (56 mg, 0.6 mmol) and HOBt (81 mg, 0.6 mmol) in DCM (5 mL) and cooled to 0 °C. *i*Pr$_2$NEt (0.26 mL, 1.5 mmol) was added dropwise over 5 min and the resulting mixture was stirred at 0 °C for 10 min. To this solution was added EDCI (144 mg, 0.75 mmol) and the resulting mixture was warmed to room temperature for 12 h. The crude residue was purified by column chromatography (DCM/MeOH: 30:1) to afford **S-3** (192 mg, 93%) as a colorless liquid.

Methyl [(*R*)-6-(allylamino)-6-oxo-2-pivalamidohexanoyl]-*L*-leucinate (S-3): ^{1}H NMR (400 MHz, $CDCl_3$) δ = 7.31 (d, J = 8.0 Hz, 1H), 6.64 (d, J = 7.3 Hz, 1H), 6.20 (d, J = 7.6 Hz, 1H), 5.87 – 5.74 (m, 1H), 5.21 – 5.05 (m, 2H), 4.58 – 4.41 (m, 2H), 3.91

– 3.80 (m, 2H), 3.69 (s, 3H), 2.41 – 2.18 (m, 2H), 1.88 – 1.54 (m, 7H), 1.20 (s, 9H), 0.92 (d, *J* = 5.2 Hz, 3H), 0.90 (d, *J* = 5.2 Hz, 3H). ^{13}C NMR (100 MHz, $CDCl_3$) δ = 179.1, 173.2, 173.0, 171.9, 134.3, 116.5, 52.4, 52.0, 50.9, 42.1, 41.2, 38.9, 35.6, 32.2, 27.6, 25.0, 23.0, 21.8, 21.4. HR-MS(ESI) *m/z* calcd for $C_{21}H_{38}N_3O_5$ $[M+H]^+$ 412.2806, found 412.2804.

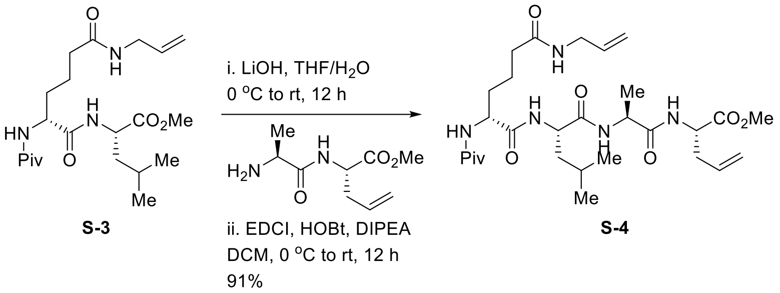

A 25 mL round-bottom flask was charged with a solution of **S-3** (164 mg, 0.4 mmol) in THF/H_2O (3:1, 8 mL) and cooled to 0 °C. LiOH (48 mg, 2.0 mmol) was added and the resulting mixture was warmed to room temperature. After 12 h, the reaction mixture was cooled to 0 °C and acidified with 1M HCl aq. to pH < 2. The solution was then diluted with EtOAc (10 mL) and the aqueous layer was extracted with EtOAc (10 mL × 3). The combined organic layers were washed with brine (10 mL), dried over Na_2SO_4, filtered and concentrated under reduced pressure to afford crude acid, which was used in the next step without further purification.

A 25 mL round-bottomed flask was charged with a solution of the crude acid (159 mg, 0.4 mmol), methyl (*S*)-2-[(*S*)-2-aminopropanamido]pent-4-enoate (96 mg, 0.48 mmol) and HOBt (65 mg, 0.48 mmol) in DCM (5 mL) and cooled to 0 °C. *i*Pr_2NEt (0.21 mL, 1.2 mmol) was added dropwise over 5 min and the resulting mixture was stirred at 0 °C for 10 min. To this solution was added EDCI (115 mg, 0.6 mmol) and the resulting mixture was warmed to room temperature for 12 h. The crude residue was purified by column chromatography (DCM/MeOH: 30:1) to afford **S-4** (211 mg, 91%) as a white solid.

Methyl (2*S*,5*S*,8*S*,11*R*)-2-allyl-8-isobutyl-5-methyl-4,7,10,15-tetraoxo-11-pivalami-do-3,6,9,16-tetraazanonadec-18-enoate (S-4): M.p. = 170–171 °C. ^1H NMR (400 MHz, $CDCl_3$) δ = 7.50 (d, *J* = 8.2 Hz, 1H), 7.42 (d, *J* = 7.7 Hz, 1H), 7.24 (d, *J* = 7.9 Hz,

1H), 6.82 (d, J = 6.9 Hz, 1H), 6.23 (t, J = 5.8 Hz, 1H), 5.87 – 5.62 (m, 2H), 5.21 – 5.02 (m, 4H), 4.67 – 4.38 (m, 4H), 3.91 – 3.79 (m, 2H), 3.70 (s, 3H), 2.60 – 2.42 (m, 2H), 2.36 – 2.19 (m, 2H), 1.87 – 1.54 (m, 7H), 1.31 (d, J = 7.1 Hz, 3H), 1.18 (s, 9H), 0.92 (d, J = 5.6 Hz, 3H), 0.89 (d, J = 5.6 Hz, 3H). ^{13}C NMR (100 MHz, $CDCl_3$) δ = 179.5, 172.8, 172.5, 172.2, 172.0, 171.9, 134.3, 132.5, 119.0, 116.5, 53.0, 52.4, 52.2, 51.9, 48.7, 42.0, 41.0, 38.8, 36.3, 35.6, 32.3, 24.9, 23.2, 21.8, 21.5, 18.1. HR-MS(ESI) *m/z* calcd for $C_{29}H_{50}N_5O_7$ [M+H]$^+$ 580.3705, found 580.3707.

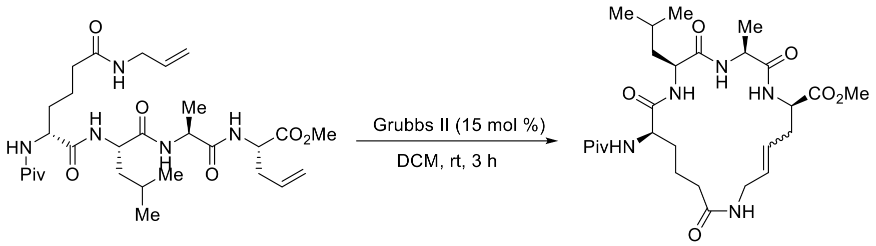

S-4 **59**: 54%

To a solution of **S-4** (58 mg, 0.1 mmol) in DCM (20 mL) was added Grubbs catalyst 2nd Generation (13 mg, 0.015 mmol). The reaction mixture was stirred for 3 h at room temperature and then concentrated onto silica gel for purification by flash chromatography (DCM/MeOH: 30:1) to afford **59** (30 mg, 54%) as a white solid.

Methyl (2*S*,5*S*,8*R*,18*R*)-2-isobutyl-5-methyl-3,6,14,19-tetraoxo-18-pivalamido-1,4,7,13-tetraazacyclononadec-10-ene-8-carboxylate (59): M.p. = 250–251 °C. ^{1}H NMR (400 MHz, DMSO-d_6) δ = 8.23 (d, J = 7.7 Hz, 1H), 8.03 (d, J = 7.5 Hz, 1H), 7.83 (t, J = 5.3 Hz, 1H), 7.79 (d, J = 7.2 Hz, 1H), 7.50 (d, J = 6.0 Hz, 1H), 5.51 – 5.44 (m, 2H), 4.30 – 4.07 (m, 4H), 3.76 – 3.50 (m, 5H), 2.43 – 2.30 (m, 2H), 2.11 – 2.05 (m, 2H), 1.65 – 1.43 (m, 7H), 1.26 (d, J = 7.3 Hz, 3H), 1.09 (s, 9H), 0.86 (d, J = 6.3 Hz, 3H), 0.80 (d, J = 6.3 Hz, 3H). ^{13}C NMR (100 MHz, DMSO-d_6) δ = 178.1, 172.8, 172.1, 171.8, 171.7, 171.5, 130.3, 126.1, 53.9, 52.1, 51.8, 51.1, 48.0, 40.2, 37.9, 34.8, 33.6, 30.7, 27.2, 24.1, 23.2, 21.7, 20.9, 17.1. HR-MS(ESI) *m/z* calcd for $C_{27}H_{46}N_5O_7$ [M+H]$^+$ 552.3392, found 552.3391. $[\alpha]_D^{28}$ = -14.9° (c = 0.15, $CHCl_3$).

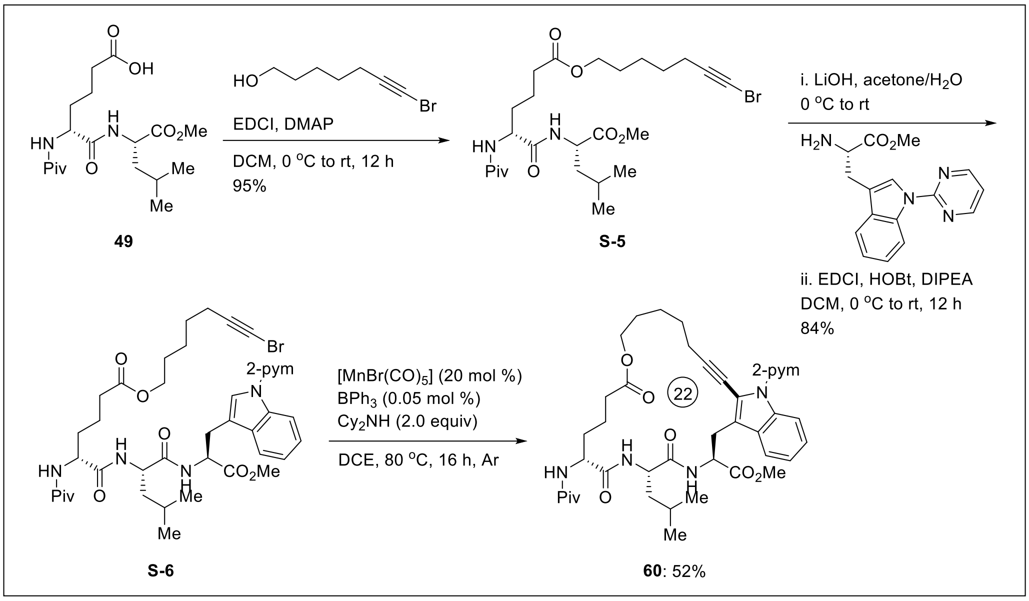

Supplementary Figure 6. Synthesis of cyclic peptide **60.**

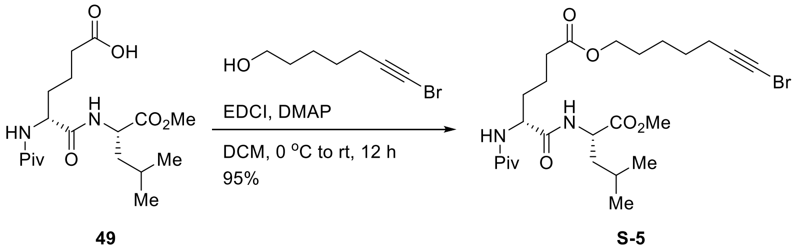

A 25 mL round-bottomed flask was charged with a solution of the compound **49** (372 mg, 1.0 mmol), 7-bromohept-6-yn-1-ol[17] (228 mg, 1.2 mmol) and DMAP (25 mg, 0.2 mmol) in DCM (5 mL) and cooled to 0 °C. To this solution was added EDCI (288 mg, 1.5 mmol) and the resulting mixture was warmed to room temperature. After 12 h, the reaction mixture was cooled to 0 °C and quenched with 1M HCl aq. (20 mL). The phases were separated and the aqueous phase was extracted with DCM (20 mL × 3). The combined organic layers were washed with brine (20 mL), dried over Na_2SO_4, filtered and concentrated under reduced pressure. The crude residue was purified by column chromatography (PE/EA: 3:1) to afford **S-5** (519 mg, 95%) as a colorless liquid.

7-Bromohept-6-yn-1-yl (*R*)-6-{[(*S*)-1-methoxy-4-methyl-1-oxopentan-2-yl]amino} -6-oxo-5-pivalamidohexanoate (S-5): ^{1}H NMR (400 MHz, $CDCl_3$) δ = 6.92 (d, J = 8.2 Hz, 1H), 6.39 (d, J = 7.6 Hz, 1H), 4.60 – 4.41 (m, 2H), 4.05 (t, J = 6.7 Hz, 2H), 3.70 (s, 3H), 2.35 (td, J = 6.9, 3.7 Hz, 2H), 2.21 (t, J = 6.9 Hz, 2H), 1.95 – 1.83 (m, 1H), 1.70 – 1.47 (m, 10H), 1.50 – 1.37 (m, 2H), 1.21 (s, 9H), 0.93 (d, J = 3.2 Hz, 3H), 0.91

(d, J = 3.2 Hz, 3H). ^{13}C NMR (100 MHz, $CDCl_3$) δ = 179.2, 173.6, 173.2, 171.7, 80.0, 64.5, 52.43, 52.40, 50.8, 41.3, 38.9, 38.1, 33.6, 31.3, 28.2, 27.9, 27.5, 25.2, 25.0, 22.9, 21.8, 20.7, 19.7. HR-MS(ESI) m/z calcd for $C_{25}H_{41}BrN_2O_6Na^+$ $[M+Na]^+$ 567.2040, found 567.2041.

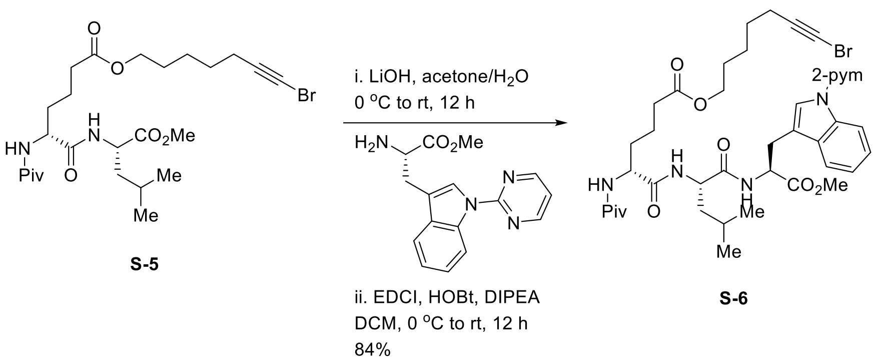

A 50 mL round-bottom flask was charged with a solution of **S-5** (544 mg, 1.0 mmol) in Acetone/H_2O (4:1, 20 mL) and cooled to 0 °C. LiOH (36 mg, 1.5 mmol) was added and the resulting mixture was warmed to room temperature. After 1 h, the reaction mixture was cooled to 0 °C and acidified with 1M HCl aq. to pH < 2. The solution was then diluted with EtOAc (20 mL) and the aqueous layer was extracted with EtOAc (20 mL × 3). The combined organic layers were washed with brine (20 mL), dried over Na_2SO_4, filtered and concentrated under reduced pressure to afford crude acid, which was used in the next step without further purification.

A 25 mL round-bottomed flask was charged with a solution of the crude acid (53 mg, 0.1 mmol), methyl 1-(pyrimidin-2-yl)-*L*-tryptophanate[18] (36 mg, 0.12 mmol) and HOBt (16 mg, 0.12 mmol) in DCM (2 mL) and cooled to 0 °C. *i*Pr_2NEt (52 μL, 0.3 mmol) was added dropwise over 5 min and the resulting mixture was stirred at 0 °C for 10 min. To this solution was added EDCI (29 mg, 0.15 mmol) and the resulting mixture was warmed to room temperature for 12 h. The crude residue was purified by column chromatography (PE/EA: 1:1) to afford **S-6** (68 mg, 84%) as a colorless liquid.

7-Bromohept-6-yn-1-yl (*R*)-6-{(*S*)-1-{{(*S*)-1-methoxy-1-oxo-3-[1-(pyrimidin-2-yl)-1*H*-indol-3-yl]propan-2-yl}amino}-4-methyl-1-oxopentan-2-yl}amino-6-oxo-5-pivalamidohexanoate (S-6): ^{1}H NMR (400 MHz, $CDCl_3$) δ = 8.76 (d, J = 8.3 Hz, 1H), 8.69 (d, J = 4.9 Hz, 2H), 8.10 (s, 1H), 7.56 (d, J = 7.7 Hz, 1H), 7.36 – 7.30 (m, 1H), 7.26 – 7.20 (m, 1H), 7.05 (dd, J = 4.8, 4.1 Hz, 1H), 6.94 (d, J = 7.8 Hz, 1H), 6.78 (d, J

= 8.1 Hz, 1H), 6.40 (d, J = 6.9 Hz, 1H), 4.94 – 4.84 (m, 1H), 4.47 – 4.36 (m, 1H), 4.28 (q, J = 6.6 Hz, 1H), 4.04 (t, J = 6.6 Hz, 2H), 3.67 (s, 3H), 3.37 – 3.28 (m, 2H), 2.40 – 2.29 (m, 2H), 2.21 (t, J = 6.9 Hz, 2H), 1.90 – 1.79 (m, 1H), 1.70 – 1.47 (m, 9H), 1.46 – 1.37 (m, 3H), 1.13 (s, 9H), 0.85 (d, J = 6.4 Hz, 6H). ^{13}C NMR (100 MHz, $CDCl_3$) δ = 179.5, 173.5, 172.0, 171.9, 171.7, 158.3, 157.6, 135.6, 131.1, 124.5, 124.1, 122.2, 118.8, 116.5, 116.2, 114.8, 80.0, 64.5, 53.3, 52.8, 52.6, 51.9, 40.8, 38.8, 38.1, 33.5, 31.0, 28.2, 27.9, 27.4, 25.1, 24.9, 23.1, 21.7, 20.8, 19.7. HR-MS(ESI) *m/z* calcd for $C_{40}H_{53}BrN_6O_7Na^+$ $[M+Na]^+$ 831.3051, found 831.3056.

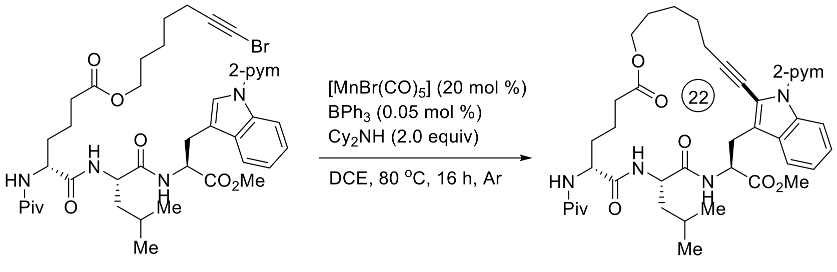

S-6 **60**: 52%

To a solution of **S-6** (81 mg, 0.1 mmol), $MnBr(CO)_5$ (5 mg, 0.02 mmol), Cy_2NH (36 mg, 0.2 mmol) and BPh_3 (10 μL, 0.05 mol %, 0.005M stock solution in DCE) in DCE (10 mL)[19]. The mixture was stirred at 80 °C for 16 h. After completion of the reaction, DCM (5 mL) was added at ambient temperature and the volatiles were removed in vacuo. Purification by flash chromatography (DCM/MeOH: 60:1) to afford **60** (38 mg, 52%) as a brown liquid.

Cyclic peptide 60: ^{1}H NMR (400 MHz, $CDCl_3$) δ = 8.83 (d, J = 4.8 Hz, 2H), 8.19 (d, J = 8.4 Hz, 1H), 7.57 (d, J = 7.8 Hz, 1H), 7.33 – 7.27 (m, 1H), 7.27 – 7.17 (m, 2H), 6.91 (d, J = 7.7 Hz, 1H), 6.84 (d, J = 8.2 Hz, 1H), 6.30 (d, J = 7.1 Hz, 1H), 4.92 (q, J = 6.5 Hz, 1H), 4.50 – 4.42 (m, 1H), 4.38 – 4.24 (m, 2H), 4.13 – 4.04 (m, 1H), 3.58 (s, 3H), 3.49 – 3.30 (m, 2H), 2.66 – 2.52 (m, 2H), 2.50 – 2.32 (m, 2H), 1.95 – 1.86 (m, 2H), 1.74 – 1.53 (m, 11H), 1.09 (s, 9H), 0.89 (d, J = 2.4 Hz, 3H), 0.87 (d, J = 2.4 Hz, 3H). ^{13}C NMR (100 MHz, $CDCl_3$) δ = 179.0, 174.1, 172.1, 171.7, 171.6, 158.3, 157.4, 135.9, 128.4, 125.0, 122.5, 120.8, 120.5, 119.0, 117.9, 114.0, 99.8, 72.7, 64.5, 52.9, 52.6, 52.4, 51.7, 41.1, 38.7, 34.0, 31.3, 29.8, 28.4, 28.1, 27.4, 25.9, 24.8, 23.1, 21.9,

21.4, 20.2. HR-MS(ESI) *m/z* calcd for $C_{40}H_{52}N_6O_7Na^+$ $[M+Na]^+$ 751.3790, found 751.3795. $[\alpha]_D^{28}$ = 10.4° (*c* = 0.19, $CHCl_3$).

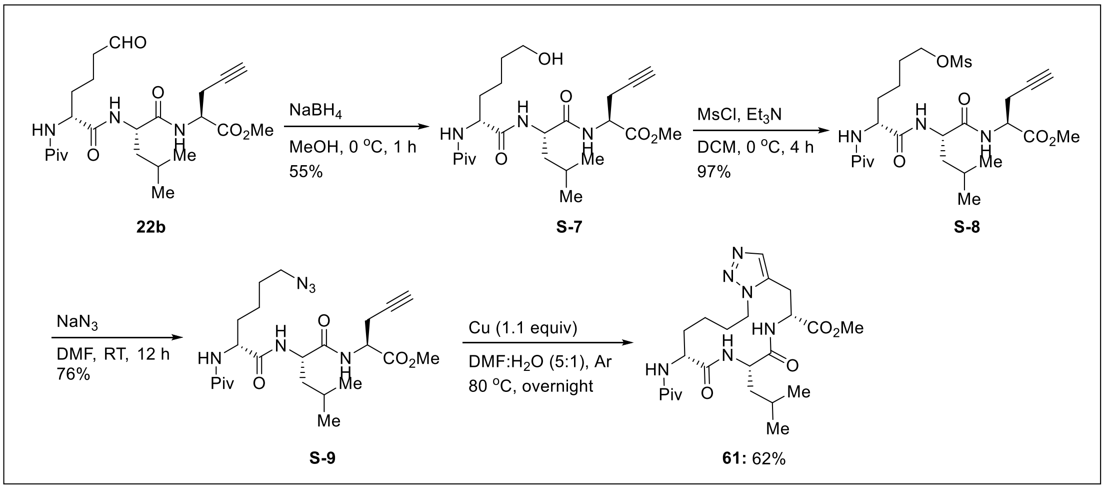

Supplementary Figure 7. Synthesis of cyclic peptide **61.**

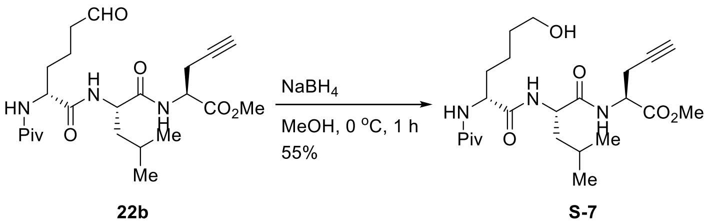

To a solution of compound **22b** (812 mg, 1.8 mmol) in MeOH (6 mL) was added $NaBH_4$ (68 mg, 1.8 mmol). The reaction mixture was stirred at 0 °C for 1 h, MeOH was evaporated and the residue was dissolved in EtOAc (20 mL). The organic layer was washed with water (20 mL × 3) and brine (20 mL × 3), and dried over Na_2SO_4, filtered and concentrated under reduced pressure. The crude residue was purified by column chromatography (DCM/MeOH: 30:1) to afford **S-7** (456 mg, 55%) as a colorless liquid.

Methyl (*S*)-2-{(*S*)-2-[(*R*)-6-hydroxy-2-pivalamidohexanamido]-4-methylpentanamido}pent-4-ynoate (S-7): ^{1}H NMR (400 MHz, $CDCl_3$) δ = 7.47 (d, *J* = 8.1 Hz, 1H), 7.18 (d, *J* = 8.0 Hz, 1H), 6.55 (d, *J* = 7.7 Hz, 1H), 4.70 – 4.61 (m, 1H), 4.54 – 4.42 (m, 2H), 3.74 (s, 3H), 3.59 (td, *J* = 6.4, 1.7 Hz, 2H), 2.71 (dd, *J* = 5.5, 2.7 Hz, 2H), 2.02 (d, *J* = 3.0 Hz, 1H), 1.90 – 1.78 (m 1H), 1.72 – 1.49 (m, 6H), 1.45 – 1.34 (m, 2H), 1.18 (s, 9H), 0.92 (d, *J* = 5.7 Hz, 3H), 0.89 (d, *J* = 5.7 Hz, 3H). ^{13}C NMR (100 MHz, $CDCl_3$) δ = 179.1, 172.5, 172.0, 170.7, 78.6, 71.8, 62.2, 53.1, 52.8, 51.9, 50.8, 40.6, 38.8, 32.5, 32.1, 27.5, 24.8, 23.1, 22.2, 21.84, 21.79. HR-MS(ESI) *m/z* calcd for $C_{23}H_{40}N_3O_6$ $[M+H]^+$ 454.2912, found 454.2910.

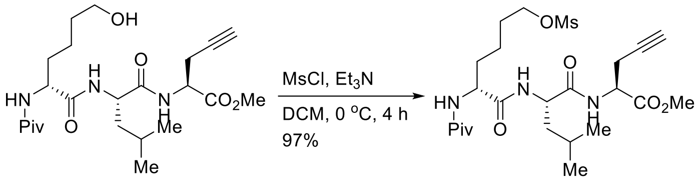

To a solution of compound **S-7** (453 mg, 1.0 mmol) and Et_3N (0.2 mL, 1.5 mmol) in anhydrous DCM (10 mL) was added dropwise methanesulfonyl chloride (93 μL, 1.2 mmol). The reaction mixture was stirred at 0 °C for 4 h, DCM was evaporated under vacuum. The residue was dissolved in EtOAc (10 mL) and washed with 10% HCl (10 mL × 3), saturated $NaHCO_3$ (10 mL × 3) and brine (10 mL × 3), and then dried over Na_2SO_4, filtered and concentrated under reduced pressure. The crude residue was purified by column chromatography (DCM/MeOH: 30:1) to afford **S-8** (515 mg, 55%) as a colorless liquid.

Methyl (*S*)-2-{(*S*)-4-methyl-2-{(*R*)-6-[(methylsulfonyl)oxy]-2-pivalamidohexanamido}pentanamido}pent-4-ynoate (S-8): ^{1}H NMR (400 MHz, $CDCl_3$) δ = 7.07 (d, J = 7.6 Hz, 1H), 7.01 (d, J = 7.8 Hz, 1H), 6.40 (d, J = 7.0 Hz, 1H), 4.70 – 4.63 (m, 1H), 4.52 – 4.41 (m, 2H), 4.27 – 4.13 (m, 2H), 3.75 (s, 3H), 3.00 (s, 3H), 2.77 – 2.69 (m, 2H), 2.02 (t, J = 2.6 Hz, 1H), 1.97 – 1.82 (m, 1H), 1.85 – 1.54 (m, 6H), 1.51 – 1.39 (m, 2H), 1.20 (s, 9H), 0.94 (d, J = 5.9 Hz, 3H), 0.91 (d, J = 5.9 Hz, 3H). ^{13}C NMR (100 MHz, $CDCl_3$) δ = 179.2, 172.1, 171.8, 170.7, 78.5, 71.8, 69.7, 52.89, 52.86, 51.9, 50.8, 40.8, 38.9, 37.5, 31.7, 28.8, 27.5, 24.9, 23.1, 22.2, 21.9, 21.6. HR-MS(ESI) *m/z* calcd for $C_{24}H_{42}N_3O_8S$ $[M+H]^+$ 532.2687, found 532.2685.

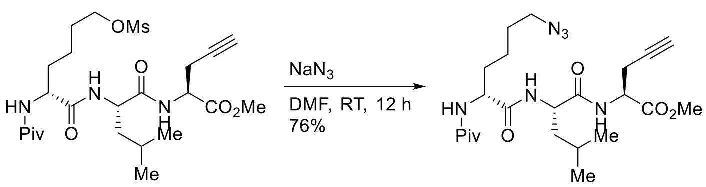

Compound **S-8** (494 mg, 0.93 mmol) was dissolved in DMF (1 mL) followed by the addition of NaN_3 (91 mg, 1.395 mmol). The mixture was stirred at room temperature for 12 h and then was quenched by being poured into ice-cold water. The mixture was extracted with EtOAc (10 mL × 3). The organic layer was washed with water (10 mL × 3) and brine (10 mL × 3), and dried over Na_2SO_4, filtered and concentrated under

reduced pressure. The crude residue was purified by column chromatography (PE/EA: 1:1) to afford **S-9** (339 mg, 76%) as a white solid.

Methyl (*S*)-2-{(*S*)-2-[(*R*)-6-azido-2-pivalamidohexanamido]-4-methylpentanamido}pent-4-ynoate (**S-9**)**:** M.p. = 89–90 °C. ^{1}H NMR (400 MHz, $CDCl_3$) δ = 7.16 (d, J = 8.1 Hz, 1H), 7.02 (d, J = 8.0 Hz, 1H), 6.40 (d, J = 7.6 Hz, 1H), 4.71 – 4.63 (m, 1H), 4.53 – 4.44 (m, 2H), 3.75 (s, 3H), 3.25 (td, J = 6.6, 2.0 Hz, 2H), 2.77 – 2.68 (m, 2H), 2.00 (t, J = 2.6 Hz, 1H), 1.94 – 1.81 (m, 1H), 1.73 – 1.52 (m, 6H), 1.44 – 1.33 (m, 2H), 1.19 (s, 9H), 0.94 (d, J = 5.9 Hz, 3H), 0.91 (d, J = 5.9 Hz, 3H). ^{13}C NMR (100 MHz, $CDCl_3$) δ = 178.9, 172.1, 171.8, 170.7, 78.5, 71.8, 52.90, 52.88, 51.8, 51.2, 50.8, 40.8, 38.9, 32.1, 28.7, 27.6, 24.9, 23.1, 22.7, 22.3, 21.8. HR-MS(ESI) *m/z* calcd for $C_{23}H_{39}N_6O_5$ $[M+H]^+$ 479.2977, found 479.2976.

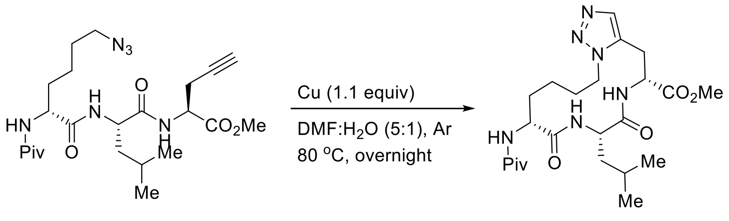

To a solution of **S-9** (48 mg, 0.1 mmol), Cu (7 mg, 0.11 mmol) in DMF/H_2O (5:1, 18 mL). The mixture was stirred at 80 °C overnight. After completion of the reaction, EtOAc (20 mL) was added and the mixture was washed with water (50 mL × 3), and brine (50 mL × 3), and then dried over Na_2SO_4, filtered and concentrated under reduced pressure. The crude residue was purified by column chromatography (DCM/MeOH: 30:1) to afford **61** (30 mg, 62%) as a yellow solid.

Methyl (5*R*,8*S*,11*R*)-8-isobutyl-7,10-dioxo-11-pivalamido-4,5,6,7,8,9,10,11,12,13,14,15-dodecahydro-[1,2,3]triazolo[5,1-g][1,4,8]triazacyclotetradecine-5-carboxylate (**61**)**:** M.p. = 276–277 °C. ^{1}H NMR (400 MHz, $CDCl_3$) δ = 62 (s, 1H), 7.14 (d, J = 8.5 Hz, 1H), 6.80 (d, J = 7.9 Hz, 1H), 6.37 (d, J = 7.4 Hz, 1H), 4.86 – 4.75 (m, 1H), 4.49 – 4.33 (m, 3H), 4.25 (q, J = 8.0 Hz, 1H), 3.77 (s, 3H), 3.46 (dd, J = 14.9, 3.3 Hz, 1H), 2.99 (dd, J = 14.9, 10.5 Hz, 1H), 1.98 – 1.76 (m, 3H), 1.69 – 1.58 (m, 2H), 1.59 – 1.45 (m, 2H), 1.19 (s, 9H), 1.13 – 1.04 (m, 1H), 0.89 (d, J = 6.1 Hz, 3H), 4.49 – 4.33 (m, 4H). ^{13}C NMR (100 MHz, $CDCl_3$) δ = 178.9, 172.1, 172.0, 171.6, 142.2, 124.2,

53.8, 52.8, 52.34, 52.29, 48.6, 39.8, 38.9, 29.2, 28.64, 28.57, 27.5, 24.7, 22.8, 22.2, 20.3. HR-MS(ESI) *m/z* calcd for $C_{23}H_{39}N_6O_5$ [M+H]$^+$ 479.2976, found 479.2974. $[\alpha]_D^{28}$ = 10.5° (c = 0.09, $CHCl_3$).

3.4 Removal of Piv- Protecting Group

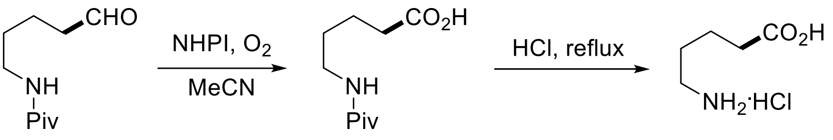

63b **63c**: 80% **77**: 82%

N-Hydroxyphthalimide (NHPI) (41 mg, 0.25 mmol) was weighed into a 50 mL Schlenk tube. After dried *in vacuo* for 15 min, 50 mL Schlenk tube was filled with oxygen. Then dry MeCN (20 mL) was added. Compound **63b** (0.925 g, 5.0 mmol) was last added and stirred at room temperature for 12 h. After **63b** completely transformed, the reaction mixture was purified by flash chromatography (PE/EA: 1/1) to afford **63c** (0.804 g, 80%) as a white solid.

5-Pivalamidopentanoic acid (63c): M.p. = 72–73 °C. ^{1}H NMR (400 MHz, $CDCl_3$) δ = 5.95 (t, J = 6.0 Hz, 1H), 3.23 (q, J = 6.6 Hz, 2H), 2.36 (t, J = 7.0 Hz, 2H), 1.70 – 1.47 (m, 4H), 1.16 (s, 9H). ^{13}C NMR (100 MHz, $CDCl_3$) δ = 179.2, 178.2, 39.2, 38.8, 33.6, 28.9, 27.6, 21.8. HR-MS(ESI) *m/z* calcd for $C_{10}H_{19}NO_3Na^+$ [M+Na]$^+$ 224.1257, found 224.1252.

63c (0.1 mmol, 1.0 equiv) was dissolved in 6*N* hydrochloric acid (0.5 M) and the mixture was refluxing at 110 °C overnight. After the completion of the reaction was confirmed by TLC, the filtrate was concentrated *in vacuo* to afford the product **77** (821 mg, 82%) as a brown liquid.

5-Aminopentanoic acid (77): ^{1}H NMR (400 MHz, DMSO-d_6) δ = 8.15 (s, 3H), 2.73 (t, J = 6.2 Hz, 2H), 2.23 (t, J = 6.6 Hz, 2H), 1.66-1.44 (m, 2H). ^{13}C NMR (100 MHz, DMSO-d_6) δ = 174.1, 38.4, 33.2, 26.4, 21.5. HR-MS(ESI) *m/z* calcd for $C_5H_{12}NO_2$ [M+H]$^+$ 118.0863, found 118.0863.

3.5 Preliminary Mechanistic Study

3.5.1 Isotopic Labeling Experiment

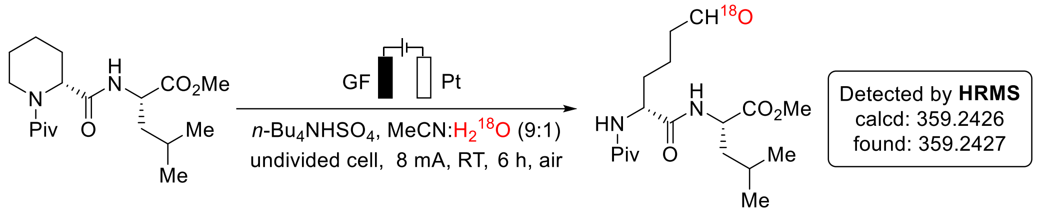

1a **1b-^{18}O**: 71%

In an undivided cell (30 mL) equipped with a stirring bar, a mixture of substrate **1a** (0.3 mmol), *n*Bu_4NHSO_4 (0.3 mmol) and MeCN/$H_2{}^{18}O$ (9:1, 10 mL) were added. The reaction mixture was stirred and electrolyzed at a constant current of 8 mA at room temperature for 6 h. The cell was equipped with graphite felt plate (1.5 cm × 1.0 cm × 0.2 cm) as the anode and platinum plate (1.5 cm × 1.0 cm × 0.01 cm) as the cathode connected to an AXIOMET AX-3003P DC regulated power supply. Upon completion, the solvent was removed directly under reduced pressure, the crude residue was purified by flash column chromatography (PE/EA: 2:1) to afford product **1b-^{18}O** (72 mg, 71%) as a white solid. HR-MS(ESI) *m/z* calcd for $C_{18}H_{33}N_2O_4{}^{18}O$ $[M+H]^+$ 359.2426, found 359.2427.

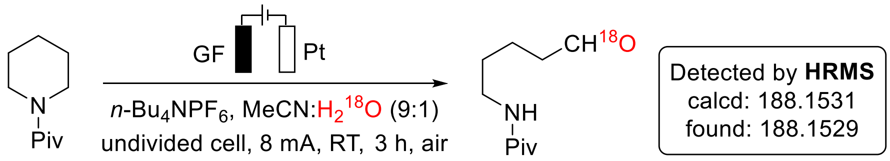

63a **63b-^{18}O**: 81%

In an undivided cell (30 mL) equipped with a stirring bar, a mixture of substrate **63a** (0.3 mmol), *n*Bu_4NPF_6 (0.3 mmol) and MeCN/$H_2{}^{18}O$ (9:1, 10 mL) were added. The reaction mixture was stirred and electrolyzed at a constant current of 8 mA at room temperature for 3 h. The cell was equipped with graphite felt plate (1.5 cm × 1.0 cm × 0.2 cm) as the anode and platinum plate (1.5 cm × 1.0 cm × 0.01 cm) as the cathode connected to an AXIOMET AX-3003P DC regulated power supply. Upon completion, the solvent was removed directly under reduced pressure, the crude residue was purified by flash column chromatography (PE/EA: 2:1) to afford product **63b-^{18}O** (45 mg, 81%) as a colorless liquid. HR-MS(ESI) *m/z* calcd for $C_{10}H_{20}NO^{18}O$ $[M+H]^+$ 188.1531, found 188.1529.

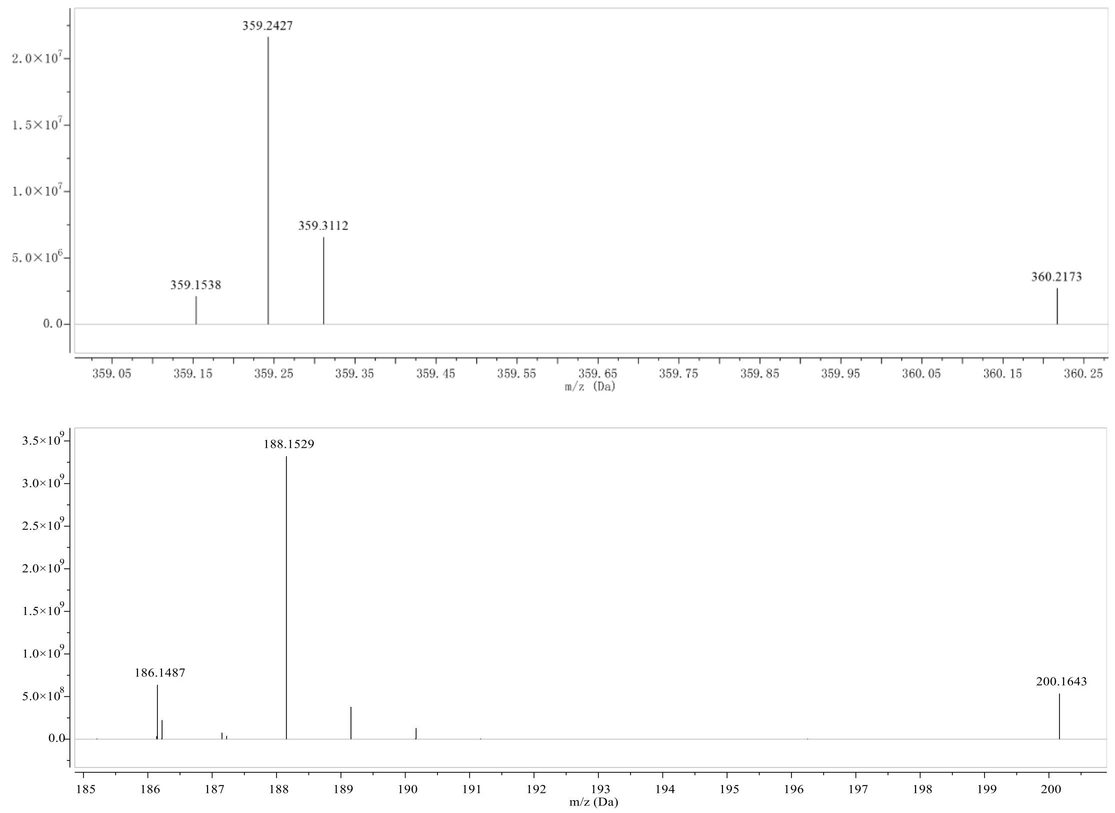

Supplementary Figure 8. HMSR spectra of **1b-^{18}O** and **63b-^{18}O**

3.5.2 H/D Exchange Experiment

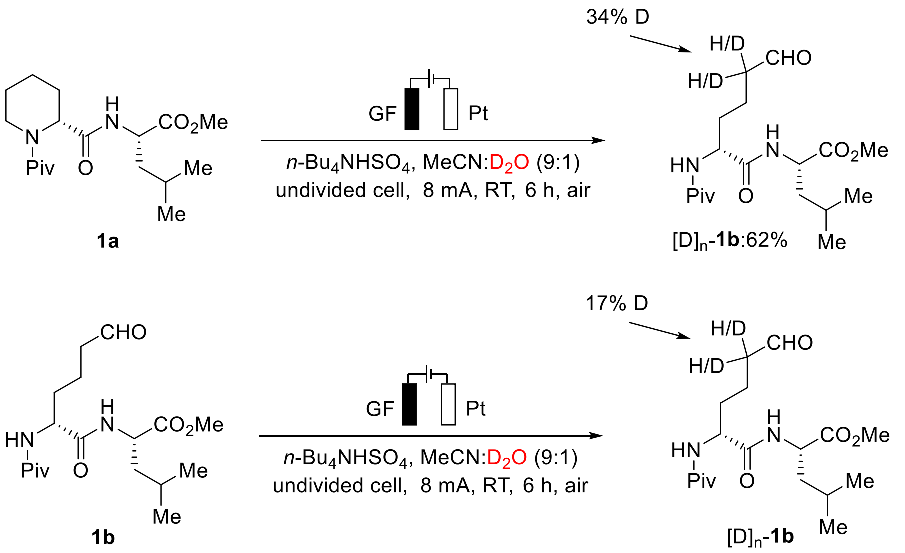

In an undivided cell (30 mL) equipped with a stirring bar, a mixture of substrate **1a** or **1b** (0.3 mmol), *n*Bu_4NHSO_4 (0.3 mmol) and MeCN/D_2O (9:1, 10 mL) were added. The reaction mixture was stirred and electrolyzed at a constant current of 8 mA at room temperature for 6 h. The cell was equipped with graphite felt plate (1.5 cm × 1.0 cm

× 0.2 cm) as the anode and platinum plate (1.5 cm × 1.0 cm × 0.01 cm) as the cathode connected to an AXIOMET AX-3003P DC regulated power supply. Upon completion, the solvent was removed directly under reduced pressure, the crude residue was purified by flash column chromatography (PE/EA: 2:1) to afford product [D]n-**1b** (66 mg, 62%) as a colorless liquid, the product [D]n-**1b** was examined by ^{1}H-NMR.

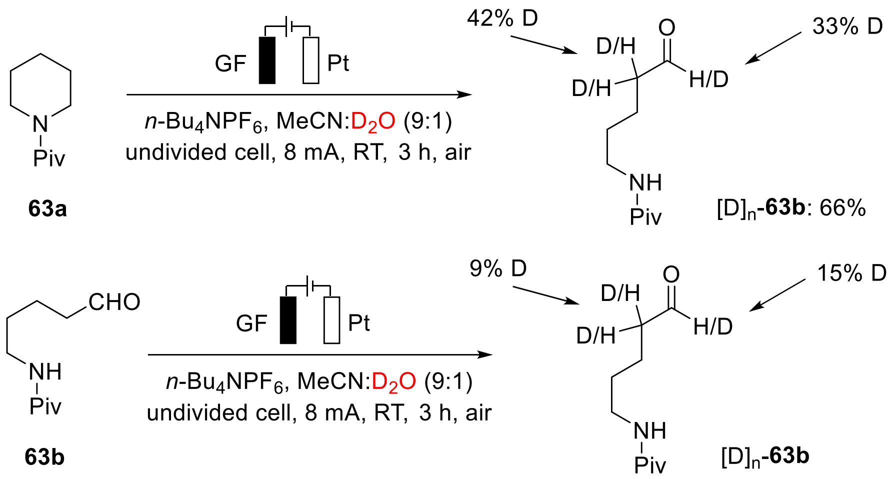

In an undivided cell (30 mL) equipped with a stirring bar, a mixture of substrate **63a** or **63b** (0.3 mmol), *n*Bu_4NPF_6 (0.3 mmol) and MeCN/D_2O (9:1, 10 mL) were added. The reaction mixture was stirred and electrolyzed at a constant current of 8 mA at room temperature for 3 h. The cell was equipped with graphite felt plate (1.5 cm × 1.0 cm × 0.2 cm) as the anode and platinum plate (1.5 cm × 1.0 cm × 0.01 cm) as the cathode connected to an AXIOMET AX-3003P DC regulated power supply. Upon completion, the solvent was removed directly under reduced pressure, the crude residue was purified by flash column chromatography (PE/EA: 2:1) to afford product [D]n-**63b** (37 mg, 66%) as a colorless liquid, the product [D]n-**63b** was examined by ^{1}H-NMR.

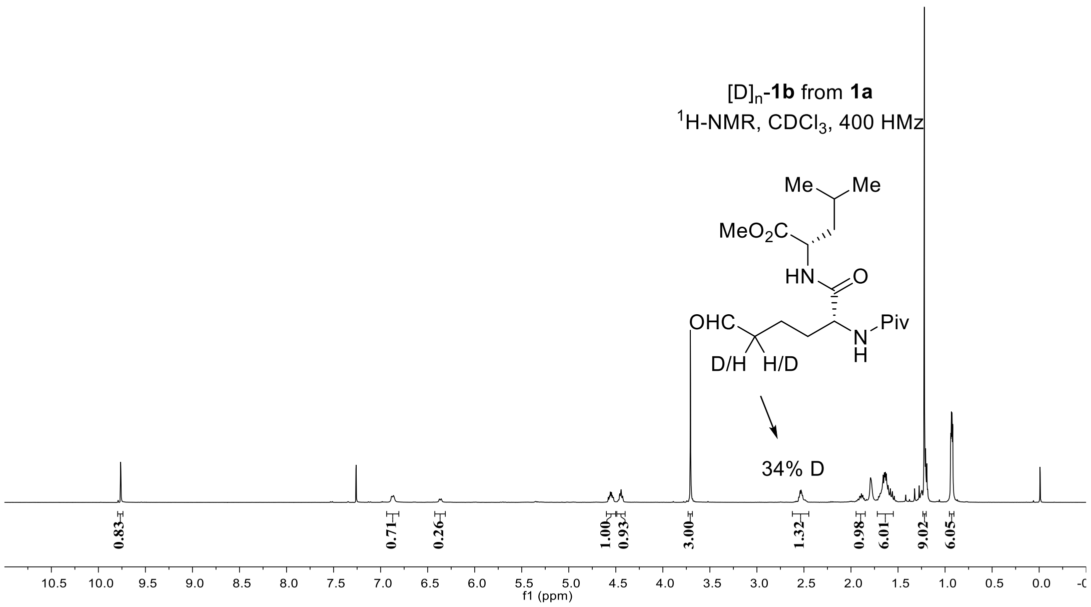
[D]n-1b from 1a
1H-NMR, CDCl3, 400 HMz
Me
Me
MeO2C
HN
O
OHC
N
Piv
H
D/H
H/D
34% D
0.83
0.71
0.26
1.00
0.93
3.00
1.32
0.98
6.01
9.02
6.05
10.5
10.0
9.5
9.0
8.5
8.0
7.5
7.0
6.5
6.0
5.5
5.0
4.5
4.0
3.5
3.0
2.5
2.0
1.5
1.0
0.5
0.0
f1 (ppm)

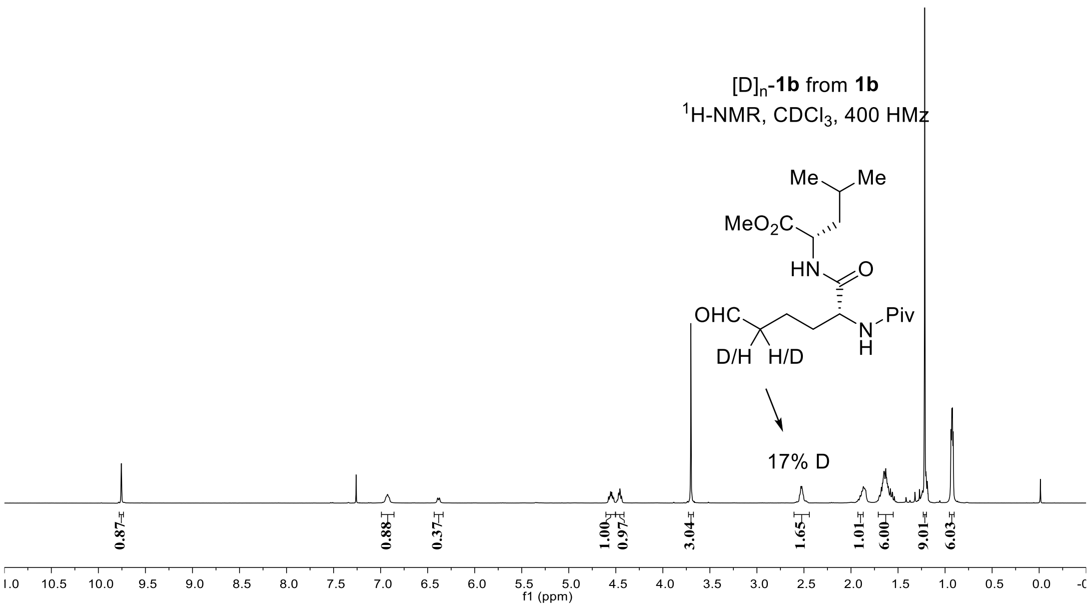
[D]n-1b from 1b
1H-NMR, CDCl3, 400 HMz
Me
Me
MeO2C
HN
O
OHC
N
Piv
H
D/H
H/D
17% D
0.87
0.88
0.37
1.00
0.97
3.04
1.65
1.01
6.00
9.01
6.03
10.5
10.0
9.5
9.0
8.5
8.0
7.5
7.0
6.5
6.0
5.5
5.0
4.5
4.0
3.5
3.0
2.5
2.0
1.5
1.0
0.5
0.0
f1 (ppm)

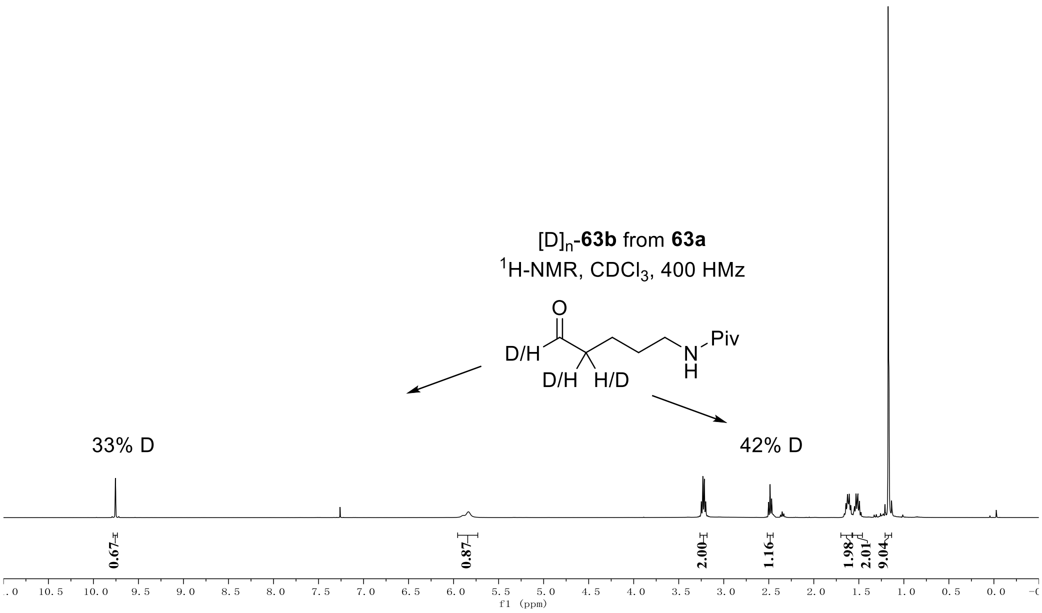

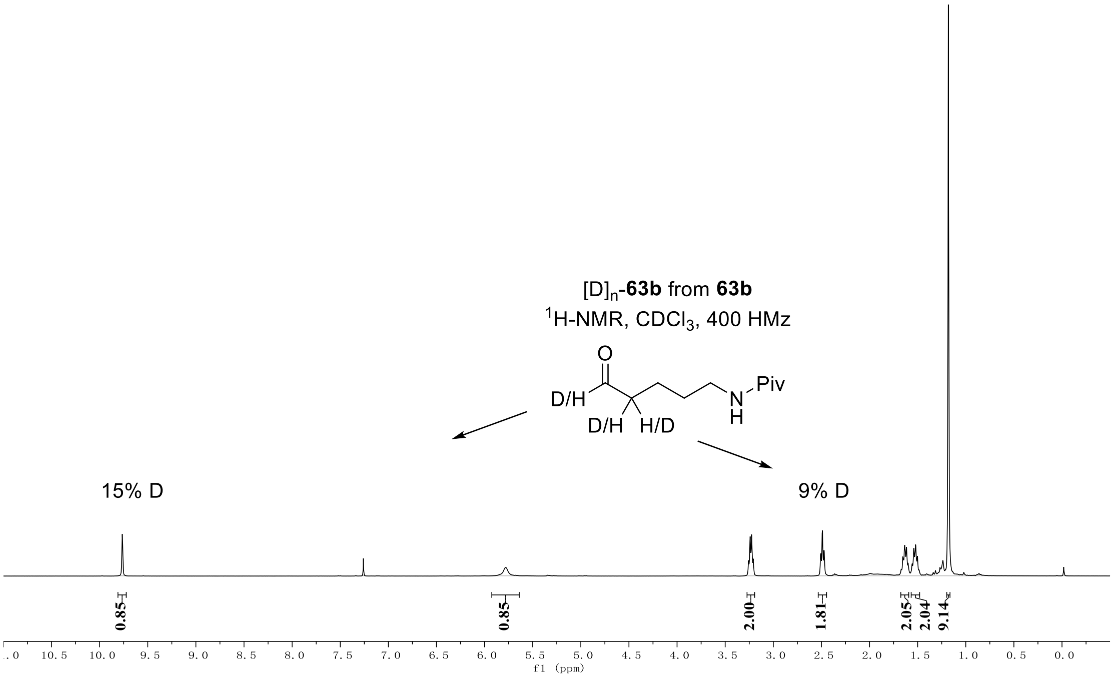

Supplementary Figure 9. ^{1}H-NMR spectra of [D]n-**1b** and [D]n-**63b**

3.5.3 Cyclic Voltammetry Studies

Cyclic voltammetry experiments were carried out on an IGS 1230 electrochemical work station (Ingsens instruments, Guangzhou). 0.1 M *n*Bu_4NPF_6 was dissolved in acetonitrile. Working electrode: glassy carbon, counter electrode: Graphite, reference electrode: Ag-AgCl (3 M KCl). Scan rate: 100 mV/s.

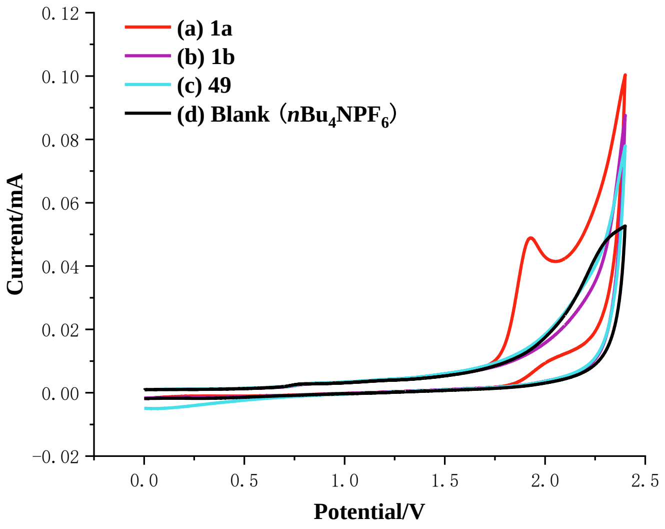

(a) Red line: **1a** (1 mM); (b) Purple line: **1b** (1 mM); (c) Blue line: **49** (1 mM); (d) Black line: background.

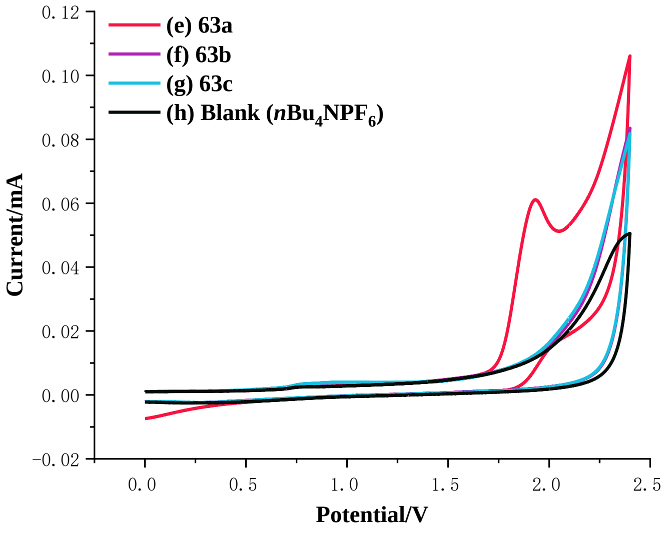

(e) Red line: **63a** (1 mM); (f) Purple line: **63b** (1 mM); (g) Blue line: **63c** (1 mM); (h) Black line: background.

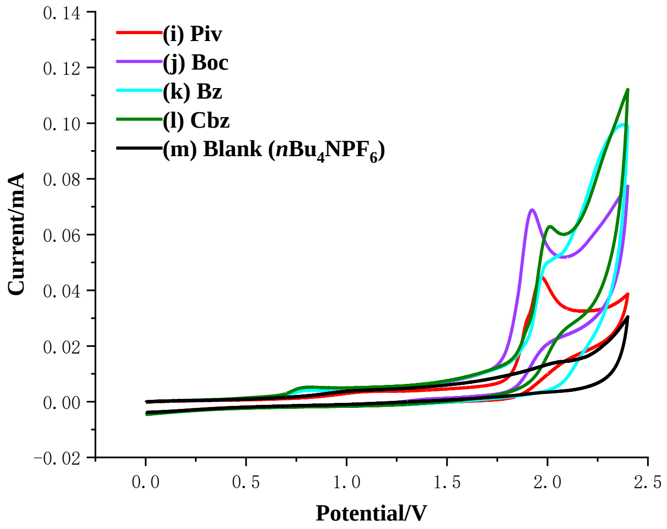

(i) Red line: **63a** (1 mM); (j) Purple line: ***tert*-butyl piperidine-1-carboxylate** (1 mM); (k) Blue line: **62a** (1 mM); (l) Green line: **benzyl piperidine-1-carboxylate** (1 mM); (m) Black line: background.

Supplementary Figure 10. Cyclic voltammetry studies. Conditions: a 0.1 M nBu$_4$NPF$_6$ solution in MeCN at room temperature; a glassy carbon working electrode, Ag/AgCl (3 M KCl) reference electrode, and a graphite counter electrode, respectively. Scan rate: 100m V/s.

3.5.4 Electricity on/off Experiment

Electricity on/off experiment were carried out by following the general procedure. In an undivided cell (30 mL) equipped with a stirring bar, a mixture of substrate **63a** (0.3 mmol), nBu$_4$NPF$_6$ (0.3 mmol) and MeCN/H_2O (9:1, 10 mL) were added. The reaction mixture was stirred and electrolyzed at a constant current of 8 mA at room temperature for 30 min. Then the reaction mixture was stirred for 30 min with electricity-off, during a total of 3 hours. The cell was equipped with graphite felt plate (1.5 cm × 1.0 cm × 0.2 cm) as the anode and platinum plate (1.5 cm × 1.0 cm × 0.01 cm) as the cathode connected to an AXIOMET AX-3003P DC regulated power supply. The GC yield of the product was monitored with dodecane as internal standard after each interval.

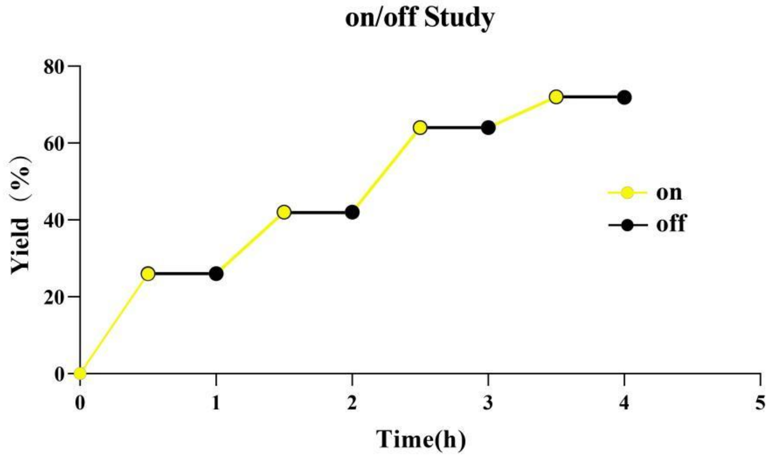

Supplementary Figure 11. Time profile of the transformation with the electricity on/off over time.

3.6 Limitation

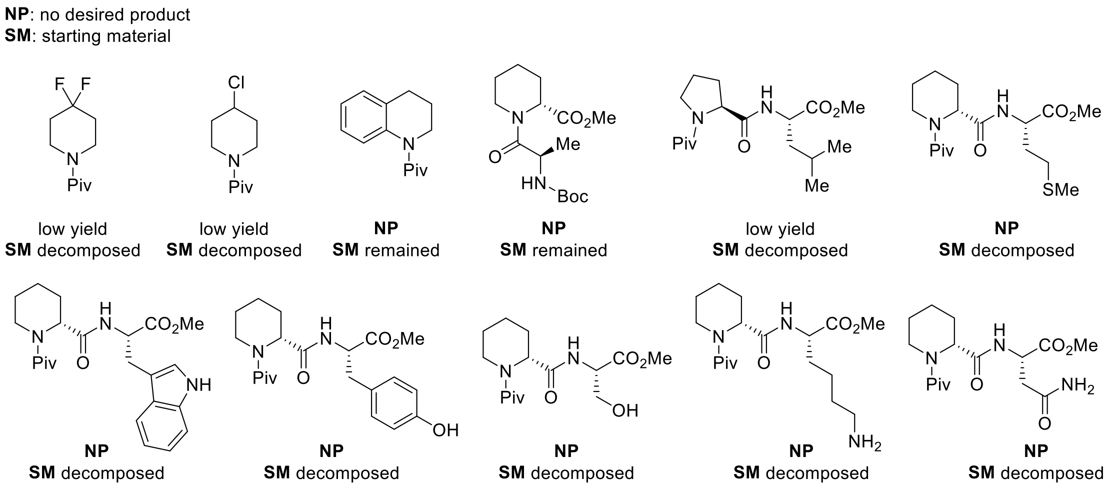

Supplementary Figure 12. Limitation of electrochemical reaction.

3.7 Theoretical Calculation of Reduction Potentials

3.7.1 General Information of DFT Computational Studies

For single-point energy (SPE) calculation of optimized geometries:

Calculation software: ORCA 5.0.3[20-21]

DFT functional: PWPB95-D4 (Double-hybrid functional PWPB95[22] with dispersion-correction DFT-D4[23-24])

Basis sets: def2-QZVPP[25]

Solvation model: SMD[26], an implicit solvation model. Parameters of the SMD model were modified to find a closer fit to the actual solution environment (MeCN:H_2O = 9:1). The input parameters are included in the "CPCM" input block, which is shown below:

```
%cpcm
    smd True
    epsilon 39.955  # Static dielectric constant
    SMDsolvent "MeCN"
    soln    1.343   # Refractive index at 293.15K
    soln25  1.341   # Refractive index at 298.15K
    sola    0.11    # Abraham's hydrogen bond acidity
    solb    0.38    # Abraham's hydrogen bond basicity
    solg    47.61   # Relative macroscopic surface tension
    solc    0.0     # Aromaticity index
    solh    0.0     # Electronegative halogenicity index
end
```

Other information: The keyword "Autoaux" was implemented to generate auxiliary basis sets[27]. Other settings were kept default.

For geometry optimization and frequency analysis:

Calculation software: Gaussian 16, Rev. A 03

DFT functional: M06-2X-D3 (Hybrid functional M06-2X[28] with dispersion-correction DFT-D3[29])

Basis sets: def2-SVP[25]

Solvation model: IEFPCM[30], an implicit solvation model. Parameters of the IEFPCM model were modified to find a closer fit to the actual solution environment (MeCN:H_2O = 9:1). Keyword "scrf=(solvent=generic)" was used. The input parameters are listed below:

```
eps=39.955 # Static dielectric constant
epsinf=1.804   # Square of the refractive index
```

Other information: The keyword "Opt=(TS, Calcfc, NoEigen)" was implemented

while searching transition states. All reactants and intermediates do not have any imaginary frequency, and each transition state only bears a sole imaginary frequency. Most other settings, like the accuracy of integration grids, and criteria of convergence, were kept default.

For the calculation of thermodynamic properties:

Calculation software: Shermo 2.3[31], the calculated harmonic frequencies from frequency analysis are required for a certain geometry. Thermal corrections, including the thermal correction to Gibbs free energy (TCG), are the output.

Environment parameters: T=298.15K and p=1atm

Treatment for low frequencies: Grimme's interpolation for entropy[32]

Harmonic vibrational frequency scale factors for zero-point energy (ZPE), thermal energy (U), and entropy (S) were set to 0.977, 0.948, and 0.952, respectively. To see how these scale factors are obtained, please check the supporting information of Feng's work[33].

In this work, we adopted the same method[33] to calculate the harmonic vibrational frequency scale factors. The procedure and test sets remained the same, however, while performing geometry optimization to molecules in test sets, M06-2X/def2-SVP was applied instead. To check test sets and source codes, please access:

https://github.com/TMSCN/Computational_Chemistry_Utils/tree/main/Scale_Factor_Generator

To convert the calculated Gibbs free energies (G_0) under the implicit solvation model applying the standard state of p^o=1atm, into those (G_{sol}) applying the standard state of c^o=1 mol/L, a correction of 1.89 kcal/mol was added[34]. Here is the relationship of the energies for a certain geometry B (T = 298.15K):

$$G_0(B) = SPE(B) + TCG(\mathrm{B}) \quad (Supplementary\ equation\ 1)$$

$$G_{sol}(B) - G_0(B) = RTln\frac{p_{sol}}{p^o} = RTln\frac{c^oRT}{p^o}$$

$$= 1.89\ \mathrm{kcal/mol} \quad (Supplementary\ equation\ 2)$$

Therefore:

$$G_{sol}(B) = SPE(B) + TCG(\mathrm{B}) + 1.89\ \mathrm{kcal/mol} \quad (Supplementary\ equation\ 3)$$

Unless especially emphasized, G_{sol}, rather than G_0, was adopted to calculate thermodynamic data (e.g. ΔG and $\Delta G^{\ddagger}$), under the standard state of T=298.15K and c^o=1 mol/L.

The level of DFT computation can be noted as SMD(MeCN/H_2O) / PWPB95-D4 / def2-QZVPP // IEFPCM(MeCN/H_2O) / M06-2X-D3 / def2-SVP.

3.7.2 Potential Calculation

All theoretical standard oxidative potentials E^{Theor} (vs. SCE, under 298.15K) were calculated via the formula below[35]:

$$E^{Theor} = \frac{G_{sol}(Ox) - G_{sol}(Red)}{n_{\mathrm{e}}\mathcal{F}} - E_{SCE}^{\mathrm{abs}} \quad (Supplementary\ equation\ 4)$$

Where n_{e} is the number of electrons transferred, $\mathcal{F}$ is Faraday's constant (96485 C/m), and E_{SCE}^{abs} is the absolute potential of a standard calomel electrode (SCE), which equals 4.429V[36].

3.7.3 Deduction of Rate Equations

For the conversion of **Int3**→Product, according to the potential energy surface in manuscript Fig. 7, we can decompose the conversion into several steps below. The formation of **Int6** was omitted:

$$\textbf{Int3} + H_2O \underset{k_{-1}}{\overset{k_1}{\rightleftharpoons}} \textbf{Int5} \quad (1)$$

$$\textbf{Int5} \underset{k_{-2}}{\overset{k_2}{\rightleftharpoons}} \textbf{Int7} \quad (2)$$

$$\textbf{Int7} \xrightarrow{k_3} \textbf{Product} \quad (3)$$

Applying the steady-state approximation to **Int5** and **Int7**:

$$\frac{d[Int5]}{dt} = 0 = k_1[Int3][H_2O] + k_{-2}[Int7] - (k_{-1} + k_2)[Int5] \quad (Supplementary\ equation\ 5)$$

$$\frac{d[Int7]}{dt} = 0 = k_2[Int5] - (k_{-2} + k_3)[Int7] \quad (Supplementary\ equation\ 6)$$

The overall rate r of conversion **Int3**→Product is:

$$r = \frac{d[Product]}{dt} = k_3[Int7] \quad (Supplementary\ equation\ 7)$$

Combine formulae (Supplementary equation 5)~(Supplementary equation 7), we can derive:

$$r = \frac{k_1k_2k_3}{k_{-1}(k_{-2} + k_3) + k_2k_3}[Int3][H_2O] \quad (Supplementary\ equation\ 8)$$

The Eyring equation reveals the relationship between the free energy barrier $\Delta G^{\ddagger}$ and rate constant k in an elementary reaction:

$$k = \kappa\frac{k_BT}{h(c^o)^{1-n}}\exp\left[-\frac{\Delta G^{\ddagger}}{RT}\right] \quad (Supplementary\ equation\ 9)$$

Where k_B is the Boltzmann constant (1.38065×10^{-23} J/K), $T = 298.15$K, h is the Planck constant (6.626×10^{-34} J·s), R is the gas constant (8.3145 $J{\cdot}mol^{-1}{\cdot}K^{-1}$), $c^o = 1$ mol/L, n is the number of reaction molecularity, $\Delta G^{\ddagger}$ is the free energy barrier of the elementary reaction, κ is the transmission coefficient, which equals 1 under most circumstances. In the case of constant temperature and $\kappa = 1$, $\Delta G^{\ddagger}$ is the only valuable decisive to the rate constant k.

To simplify the formula (Supplementary equation 7) and for the sake of convenience, we need some additional approximations. Inferred from the free energy barrier differences of the potential energy surface, using the Eyring equation, compared with k_{-2} ($\Delta G^{\ddagger} \approx 6$ kcal/mol), k_3 ($\Delta G^{\ddagger} \approx 12$ kcal/mol) is down 4~5 orders of magnitude ($\exp\left(-\frac{6\ kcal/mol}{RT}\right) = 4 \times 10^{-5}$). Therefore, $k_{-2} \gg k_3$. Similarly, using the Eyring equation, we can derive $k_{-1}k_{-2} \gg k_2k_3$. Therefore:

$$r = \frac{k_1k_2k_3}{k_{-1}k_{-2}}[Int3][H_2O] = K_1K_2k_3[Int3][H_2O] \quad (Supplementary\ equation\ 10)$$

Where K_1 and K_2 are the equilibrium constants of the first two steps under the standard state T=298.15K and c^o=1 mol/L. Using $\Delta G = -RT\ln K$ and the Eyring equation, we can derive:

$$r = \frac{k_BT}{h}\exp\left[-\frac{\Delta G_1 + \Delta G_2 + \Delta G_3^{\ddagger}}{RT}\right][Int3][H_2O]$$

$$= \frac{k_BT}{h}\exp\left[-\frac{\Delta G(\text{Int3} \rightarrow \text{TS4})}{RT}\right][Int3][H_2O] \quad (Supplementary\ equation\ 11)$$

Considering the quick isomerization of **Int3** into **Int3′**:

$$[Int3] = \frac{[Int3] + [Int3']}{1 + \exp\left[-\frac{\Delta G(\text{Int3} \rightarrow \text{Int3}')}{RT}\right]}$$

$$= \frac{1}{1 + \exp\left[-\frac{\Delta G(\text{Int3} \rightarrow \text{Int3}')}{RT}\right]} c(Int3) \quad (\textit{Supplementary equation } 12)$$

Where c(Int3) is the analytic concentration of **Int3**. Apply Supplementary equation 12 to Supplementary equation 11:

$$r = \frac{k_B T \exp\left[-\frac{\Delta G(\text{Int3} \rightarrow \text{TS4})}{RT}\right]}{h\left[1 + \exp\left(-\frac{\Delta G(\text{Int3} \rightarrow \text{Int3}')}{RT}\right)\right]} c(Int3)[H_2O] \quad (\textit{Supplementary equation } 13)$$

Therefore,

$$r = k_{obs}[\text{H}_2\text{O}] \cdot c(\text{Int3}) \quad (\textit{Supplementary equation } 14)$$

$$k_{obs} = \frac{k_B T}{h[1 + \exp(-\frac{\Delta G(Int3 \rightarrow Int3')}{RT})]} \exp\left[-\frac{\Delta G(\text{Int3} \rightarrow \text{TS4})}{RT}\right] \quad (\textit{Supplementary equation } 15)$$

3.7.4 The origin of different calculated reactivity among *N*-substituted piperidine substrates (R = Piv, Ac, Boc, Bz, Cbz)

The calculated k_{obs} values indicate that substrates with the Piv-protecting group exhibit the quickest reactions among other calculated substrates. However, this does not provide a clear explanation for why the other protecting groups, such as Cbz and Boc, are predicted ineffective. Therefore, we tried to seek for the origin of the different calculated reactivity among *N*-substituted piperidine substrates.

The computed k_{obs}, which predicts the reaction rate constant of the overall reaction of iminium (**Int3**) decyclization: **Int3** + H_2O → Product, was related to the relative free energy difference between **TS4** and **Int3** + H_2O, which can be decomposed into G_{rel}(**TS4**) − G_{rel}(**Int7**) and G_{rel}(**Int7**) − G_{rel}(**Int3**). Although the activation energies from **Int7** to **TS4** (G_{rel}(**TS4**) − G_{rel}(**Int7**)) of different substrates are close (12~13 kcal/mol, Supplementary Table 5), there lie significant differences in the relative free energy of the **Int7** intermediate (G_{rel}(**Int7**) − G_{rel}(**Int3**), 5~11 kcal/mol, Supplementary Table 4), strongly contributing to the differences in the k_{obs}.

Because the G_{rel} of **Int7** and the protonation energie changes (G_{rel}(**Int7**) − G_{rel}(**Int6**), -0.5~5 kcal/mol, Supplementary Table 5) differ prominently, we assumed that the G_{rel} of **Int7** was greatly influenced by the basicity of N atom in **Int6**, determined by electronic effect and steric effect.

To consider the electronic effect, we calculated the energies of acetyl-substituted intermediates (Ac, Supplementary Table 4), where the electronic effect of Ac group is similar with the Piv group. According to results, G_{rel}(**Ac_Int7**) and G_{rel}(**Ac_TS4**) were prominently higher than G_{rel}(**Piv_Int7**) and G_{rel}(**Piv_TS4**). Therefore, when the electronic effect of Acyl groups are similar, the steric effect may make a great impact. Furthermore, G_{rel}(**Ac_Int7**) was observed lower than G_{rel}(**Cbz_Int7**) and G_{rel}(**Boc_Int7**), indicating that a less electron-withdrawing group might raise the G_{rel}(**Int7**).

The steric effect may arise from the repulsion force between the acyl group and the piperidine ring, which may contribute to the torsion of the acyl group in **Int6** intermediates. The torsion of acyl renders the nitrogen atom in **Int6** donating less electron density to the carbonyl through conjugation, thus leading to the improvement of the basicity of the N atom. In Supplementary Figure 13, e defined a dihedral *d*(C1-N-C2-O) for **Int6** to measure the extent of such torsion. Shown in Table S6, when the *N*-acyl is formyl (CHO), the steric hindrance is too negligible to render a torsion, and thus *d*(C1-N-C2-O) is close to 180°. As for **Bz_Int6** and **Piv_Int6**, *d*(C1-N-C2-O) is around 140°, indicating an obvious acyl torsion, preventing the conjugation between the nitrogen atom and the carbonyl.

To characterize the extent of the conjugation between N atom and the carbonyl, the calculated Mayer bond order[37-38] (MBO) of C–N bond may be an appropriate descriptor. Obviously, the MBO of C–N bond in **Piv_Int6** intermediate is lower than others significantly (1.211, Supplementary Table 6), indicating lower conjugation electron donation and stronger basicity. MBO values were obtained directly from the ORCA output file, where the calculated SPE was adopted.

To summarize, through analyses on calculated energies, interpretation of electron and steric effects, and MBO analysis, we believed that the steric hindrance between Piv

group and the piperidine ring, and the ensuing torsion of acyl group, is the main factor in increasing the basicity of the nitrogen atom in **Int6**, and distinguishing its reactivity from the Ac-, Boc-, Cbz- substituted intermediates, whose electronic effect is similar. It hence lower the G_{rel}(**Piv_Int7**), which can provide a better explanation on the better reactivity of the Piv-substituted reactants.

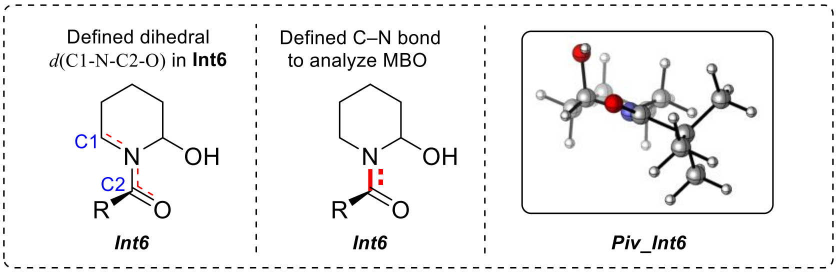

Supplementary Figure 13. Left: the definition of dihedral *d*(C1-N-C2-O) in **Int6**, labelled in red dashed lines; Medium: the defined C–N bond to analyze Mayer bond order (MBO), in red dashed lines; Right: The 3D-structure of the **Piv_Int6** intermediate, generated by CYLView20[39].

Supplementary Table 4. Relative Gibbs free energies (G_{rel}) of selected intermediates, with different *N*-acyl groups. Energy unit: kcal/mol.

***N*-acyl Group & G_{rel}**	**Int3**	**TS2**	**Int5**	**Int6**	**Int7**	**TS4**
Ac	0.0	15.19	8.11	5.25	8.42	21.58
Boc	0.0	16.10	13.24	6.58	10.77	23.63
Bz	0.0	14.31	9.47	5.10	8.08	20.67
Cbz	0.0	15.15	14.85	4.74	9.69	22.34
Piv	0.0	14.70	8.98	5.92	5.66	17.78

Supplementary Table 5. Gibbs free energies differences between selected intermediates, with different *N*-acyl groups. Energy unit: kcal/mol.

***N*-acyl Group**	**G_{rel}(TS3) − G_{rel}(Int7)**	**G_{rel}(Int7) − G_{rel}(Int6)**
Ac	13.16	3.17
Boc	12.86	4.19
Bz	12.55	2.98
Cbz	12.65	4.95
Piv	12.12	-0.26

Supplementary Table 6. Dihedral *d*(C1-N-C2-O) and Mayer bond order (MBO) of C–N bond, of different **Int6** intermediates.

***N*-acyl Group**	**Dihedral *d*(C1-N-C2-O) (°)**	**MBO of C–N bond**
Ac	153.9	1.262
Boc	155.5	1.283
Bz	138.5	1.223
Cbz	169.9	1.276
CHO	179.3	1.278
Piv	142.1	**1.211**

3.7.5 Computed Energies of Stationary Points

Supplementary Table 7. Single-point energies (SPE) and thermal corrections to Gibbs free energies (TCG). Names of the Piv-substituted structures (**Piv_IntX**) are abbreviated to **IntX**. 1 Hartree = 627.51 kcal/mol = 2625.5 kJ/mol.

Structures	SPE (Hartree)	TCG (Hartree)
**62a**	-522.3866159	0.238644
**Ac_Int3**	-403.7105097	0.148891
**Ac_Int3'**	-403.7115146	0.148958
**Ac_Int5**	-480.1540285	0.176746
**Ac_Int6**	-479.7231369	0.163827
**Ac_Int7**	-480.1533288	0.176546
**Ac_TS2**	-480.1391313	0.17312
**Ac_TS4**	-480.1281377	0.17232
**Boc_Int3**	-596.851299	0.230742
**Boc_Int3'**	-596.8507232	0.230514
**Boc_Int5**	-673.2858021	0.25775
**Boc_Int6**	-672.8612726	0.245136
**Boc_Int7**	-673.294199	0.259201
**Boc_TS2**	-673.2781743	0.254679
**Boc_TS4**	-673.2660956	0.254598
**Bz_Int3**	-595.3996736	0.197324
**Bz_Int3'**	-595.4010267	0.197378
**Bz_Int5**	-671.8426176	0.225462

**Bz_Int6**	-671.4143297	0.212733
**Bz_Int7**	-671.845504	0.226133
**Bz_TS2**	-671.8298613	0.221729
**Bz_TS4**	-671.8212307	0.221921
**Cbz_Int3**	-709.933692	0.227401
**Cbz_Int3'**	-709.9328745	0.228429
**Cbz_Int5**	-786.368289	0.254057
**Cbz_Int6**	-785.9463	0.241497
**Cbz_Int7**	-786.3754789	0.256034
**Cbz_TS2**	-786.3621242	0.25139
**Cbz_TS4**	-786.3499576	0.250672
**H_2O**	-76.43494564	0.00334
**H_2SO_4**	-700.2924321	0.010536
**HSO_4^-**	-699.8579531	-0.001414
**Int1**	-522.1613067	0.236201
**Int2**	-521.7276844	0.224334
**Int3**	-521.6057518	0.227079
**Int3'**	-521.6069175	0.226637
**Int4**	-521.166218	0.215289
**Int5**	-598.0485846	0.255625
**Int6**	-597.6179901	0.242686
**Int7**	-598.0541344	0.25589
**Int8**	-598.0570389	0.250351
**Int9**	-1297.937641	0.270501
**TS1**	-521.6066837	0.227031
**TS2**	-598.0351743	0.251335
**TS3**	-1297.919547	0.27362
**TS4**	-598.0309999	0.252066
**TS5**	-597.571691	0.238971

3.8 Antioxidant Activity Assay

3.8.1 DPPH Radical-Scavenging Activity

DPPH (2,2-Diphenyl-1-picrylhydrazyl) radical scavenging activity was detected as previously described with minor modification[40]. Briefly, compound solutions at different concentrations (50 μL) were added to an equal amount of DPPH solution (0.2 mM in ethanol). The mixtures were incubated in darkness at room temperature for 30 min. The absorbance was measured at 517 nm by a microplate reader. DPPH radical-scavenging rate were calculated using the formula: DPPH radical-scavenging activity (%) = (Acontrol - Asample)/Acontrol × 100%. Glutathione (GSH) was set as positive control.

3.8.2 Results

The Antioxidant activity of the compound were evaluated by DPPH radical-scavenging activity assays. As showed at **Supplementary Figure 14** and **15**, **21b**, **22b** and **24b** exhibited mild antioxidant activity at a concentration-dependent manner.

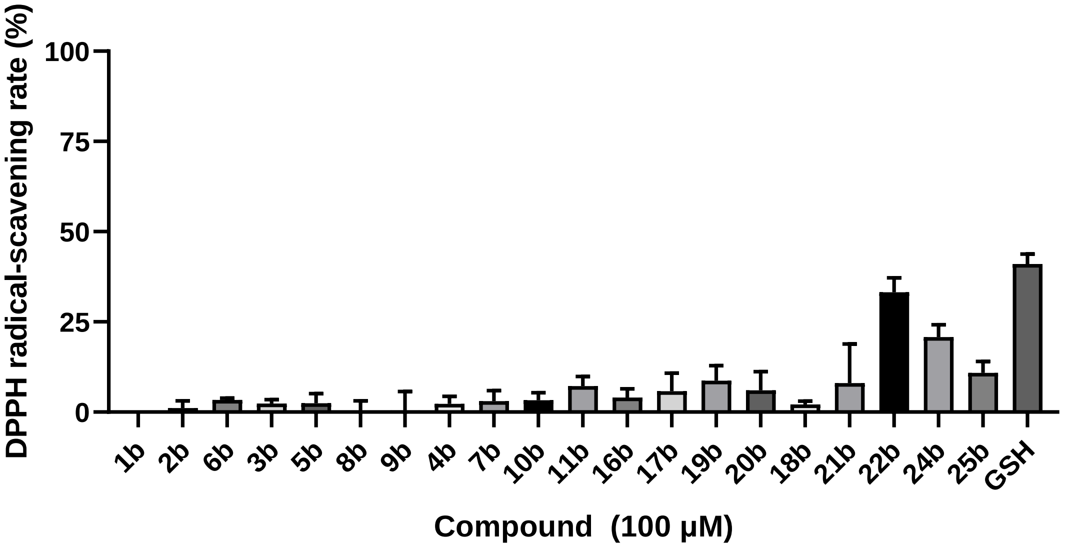

Supplementary Figure 14. DPPH radical-scavenging activity of compounds at 100 μM.

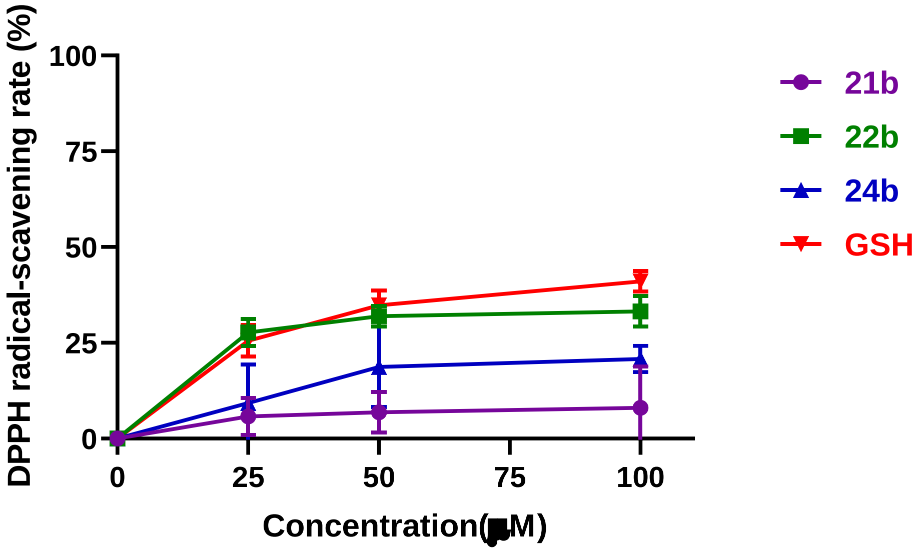

Supplementary Figure 15 DPPH radical-scavenging activity of compound **21b**, **22b** and **24b** at different concentrations.

3.9 Studies on a Potential Racemization of Peptides

In order to confirm that no racemization occurred within the peptides during the electro-oxidative ring-opening reaction, we prepared the diastereomers substrate methyl (1-pivaloylpiperidine-2-carbonyl)-*L*-leucinate (*dia*-**1a**) and racemic substrate methyl 1-pivaloylpiperidine-2-carboxylate (*rac*-**66a**). Ring-opening reactions were carried out under the optimized reaction conditions. The electro-oxidative ring-opening reaction *dia*-**1a** and *rac*-**66a** yielded diastereomers product *dia*-**1b** and racemic product *rac*-**66b**, respectively. NMR spectra and Chiral HPLC analysis showed that no racemization after the electro-oxidative ring-opening reaction process.

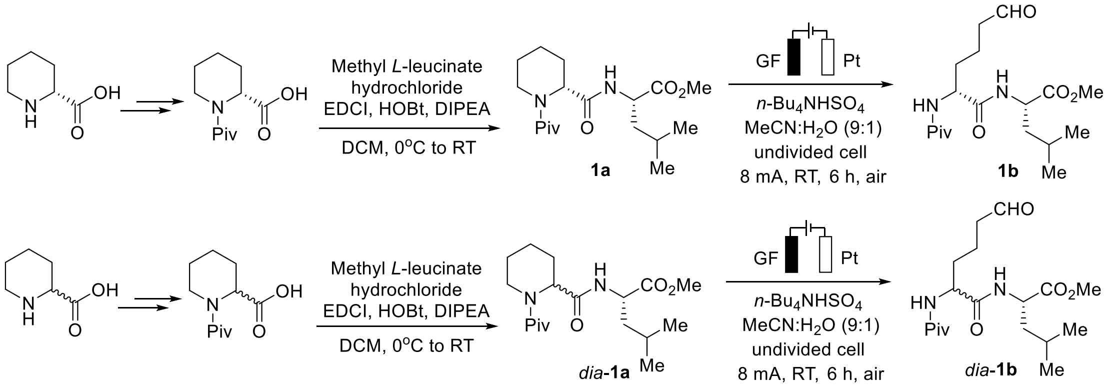

Methyl [(*R*)-6-oxo-2-pivalamidohexanoyl]-*L*-leucinate (1b): ^{1}H NMR (400 MHz, $CDCl_3$) δ = 9.75 (s, 1H), 7.01 (d, *J* = 8.1 Hz, 1H), 6.42 (d, *J* = 7.7 Hz, 1H), 4.59 – 4.51 (m, 1H), 4.51 – 4.43 (m, 1H), 3.70 (s, 3H), 2.57 – 2.46 (m, 2H), 1.93 – 1.82 (m, 1H), 1.73 – 1.52 (m, 6H), 1.21 (s, 9H), 0.93 (d, *J* = 4.9 Hz, 3H), 0.91 (d, *J* = 4.9 Hz, 3H). ^{13}C NMR (100 MHz, $CDCl_3$) δ = 202.2, 179.1, 173.2, 171.6, 52.42, 52.40, 50.9, 43.4, 41.3, 38.9, 31.5, 27.6, 25.0, 22.9, 21.8, 17.7.

Methyl (6-oxo-2-pivalamidohexanoyl)-*L*-leucinate (*diastereomers*-**1b**, 3.6:1)**:** ^{1}H NMR (400 MHz, $CDCl_3$) δ = 9.72 (s, 1H), 7.30 (d, *J* = 8.1 Hz, 1H), 6.52 (d, *J* = 7.7 Hz, 1H), 4.59 – 4.43 (m, 2H), 3.67 (s, 3H), 2.56 – 2.41 (m, 2H), 1.92 – 1.75 (m, 1H), 1.74 – 1.48 (m, 6H), 1.19 (s, 9H), 0.91 (d, *J* = 6.2 Hz, 3H), 0.88 (d, *J* = 6.2 Hz, 3H). ^{13}C NMR (100 MHz, $CDCl_3$) δ = 202.3, 179.0, 173.2, 171.7, 52.4, 52.3, 50.7, 43.4, 41.1, 38.8, 31.8, 27.5, 24.9, 22.9, 21.6, 17.6.

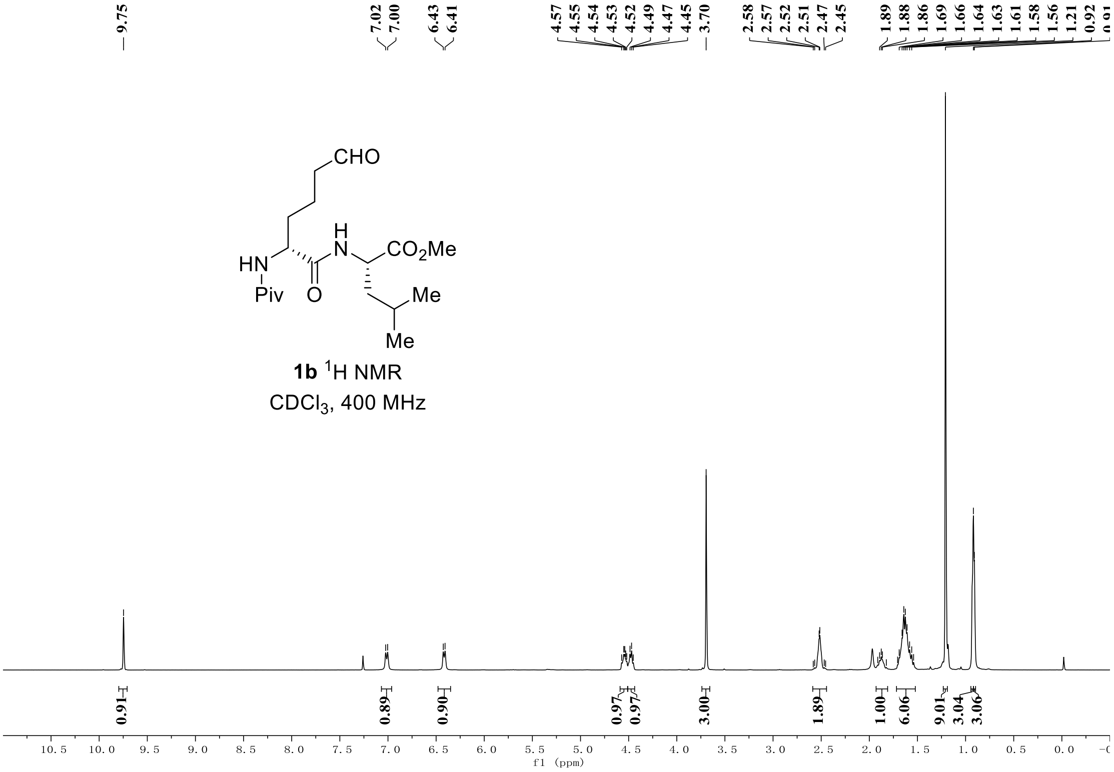

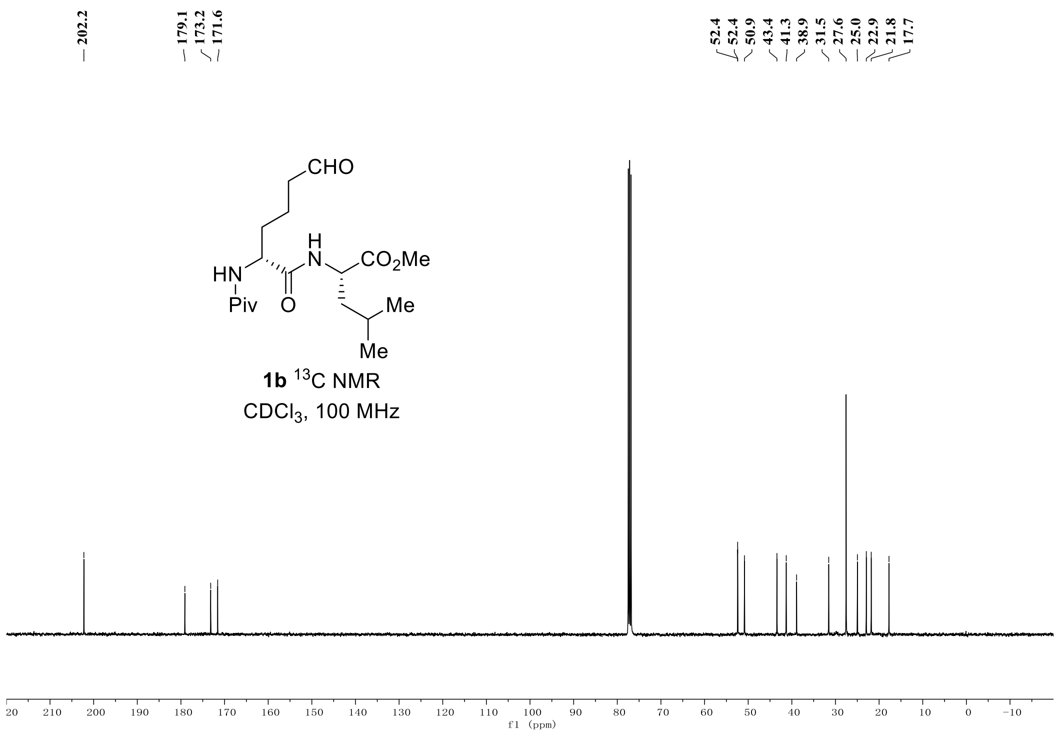
202.2
179.1
173.2
171.6
52.4
52.4
50.9
43.4
41.3
38.9
31.5
27.6
25.0
22.9
21.8
17.7
CHO
H
HN
N
CO2Me
Piv
O
Me
Me
1b 13C NMR
CDCl3, 100 MHz
f1 (ppm)

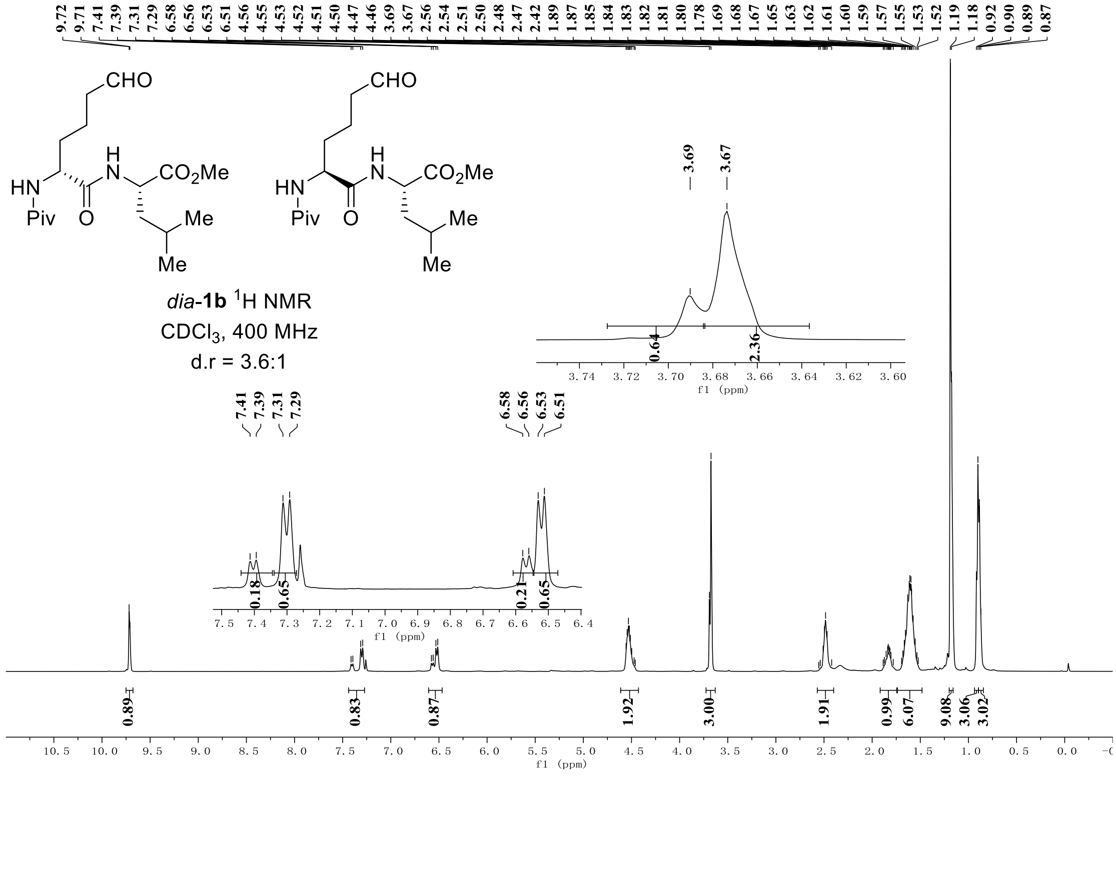
9.72
9.71
7.41
7.39
7.31
7.29
6.58
6.56
6.53
6.51
4.56
4.55
4.53
4.52
4.51
4.50
4.47
4.46
3.69
3.67
2.56
2.54
2.51
2.50
2.48
2.47
2.42
1.89
1.87
1.85
1.84
1.83
1.82
1.81
1.80
1.78
1.69
1.68
1.67
1.65
1.63
1.62
1.61
1.60
1.59
1.57
1.55
1.53
1.52
1.19
1.18
0.92
0.90
0.89
0.87
CHO
H
HN
N
CO2Me
Piv
O
Me
Me
dia-1b 1H NMR
CDCl3, 400 MHz
d.r = 3.6:1
0.64
2.36
0.18
0.65
0.21
0.65
0.89
0.83
0.87
1.92
3.00
1.91
0.99
6.07
9.08
3.06
3.02
f1 (ppm)

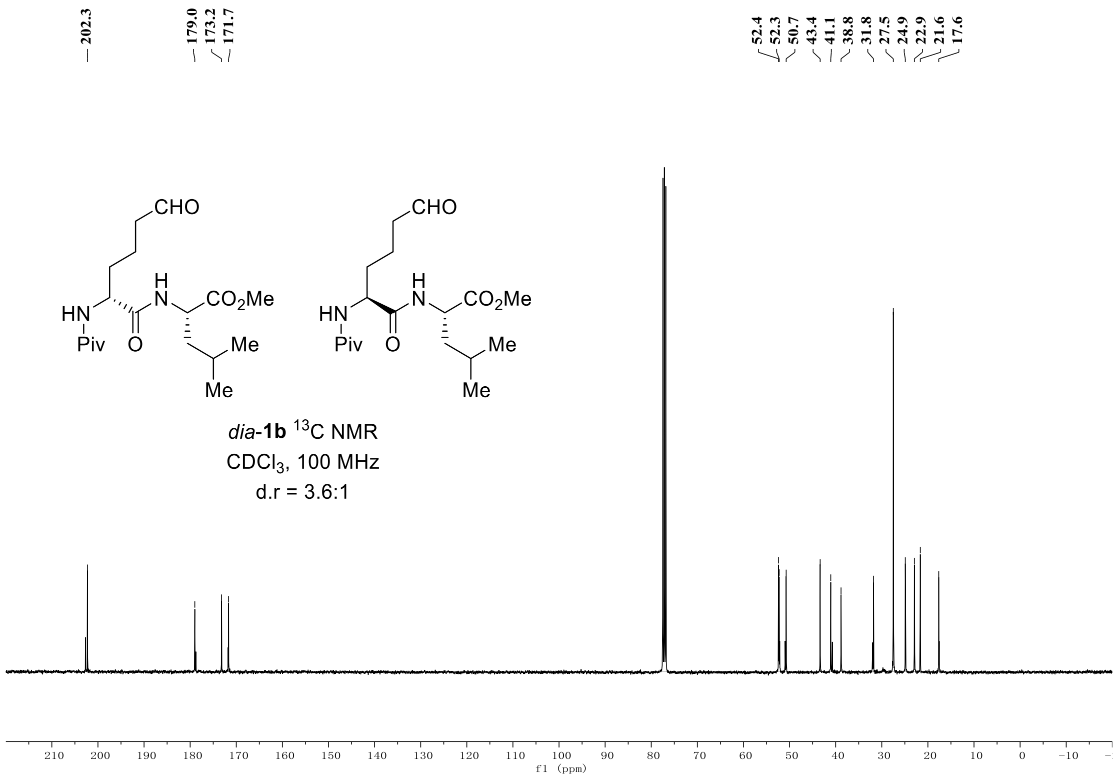

Supplementary Figure 16. NMR spectra of the compound **1b** and *dia*-**1b**.

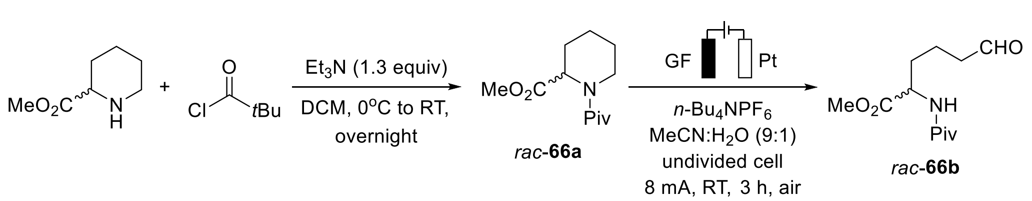

HPLC chromatograms were recorded on an UltiMate3000 Infinity using the column CHIRALPAK® ID and H_2O/MeOH (50:50, 0.5 mL/min, detection at 199nm/UV).

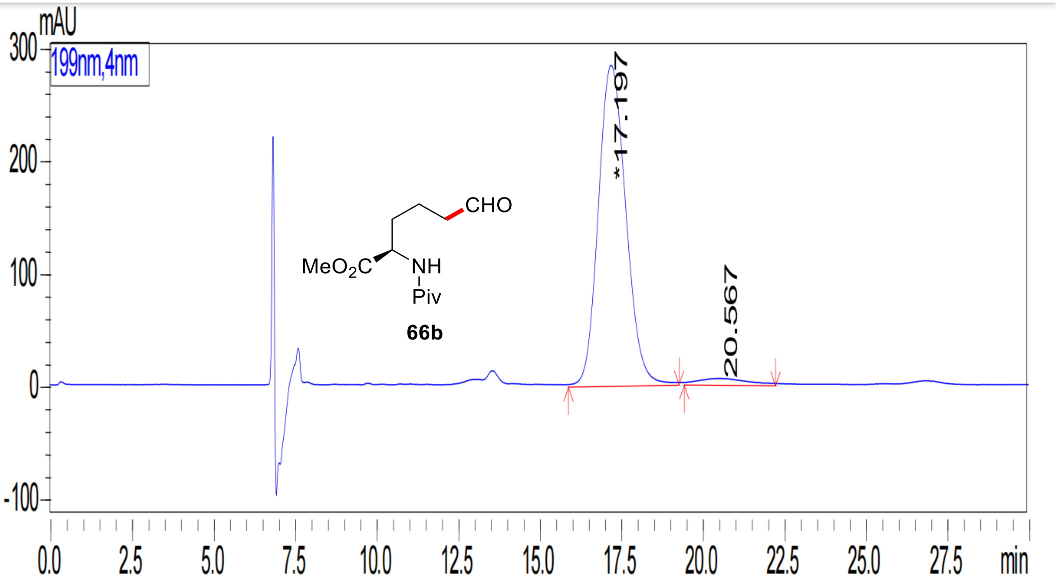

<Column Performance Report>

Peak No.	Time	Area	Area %	Plate number	Tailing	Resolution
1	17.197	16346064	98.066	2031	1.147	--
2	20.567	322321	1.934	1243	1.208	1.747

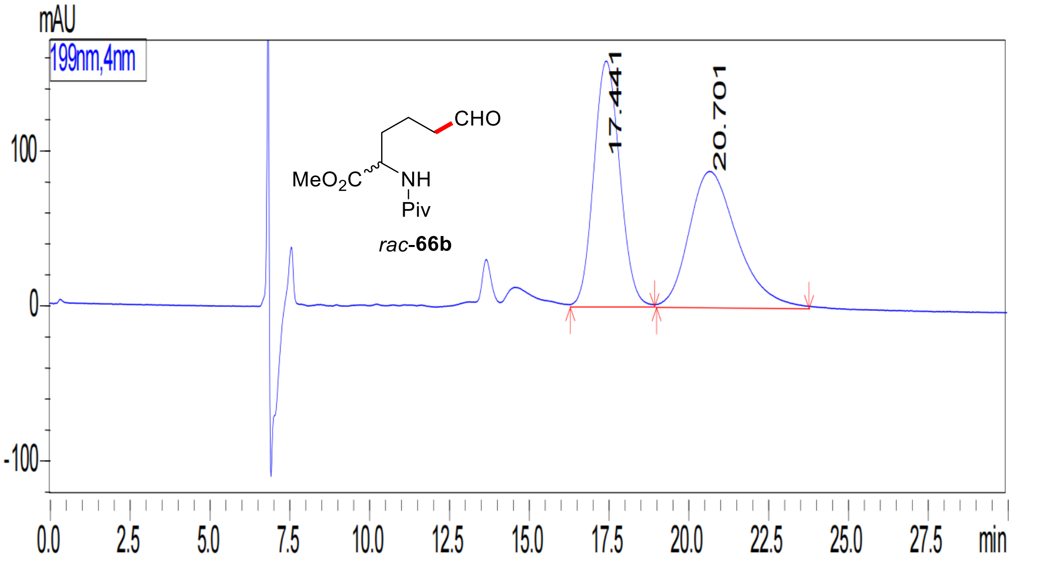

<Column Performance Report>

Peak No.	Time	Area	Area %	Plate number	Tailing	Resolution
1	17.441	8790134	49.874	2184	1.101	--
2	20.701	8834701	50.126	965	1.301	1.568

Supplementary Figure 17. HPLC-Chromatograms of the compound **66b** and *rac*-**66b**.

3.10 X-Ray Crystallographic Data of 11b

3.10.1 Crystal data and structure refinement for 11b.

Identification code	2216047
Empirical formula	$C_{18}H_{30}N_2O_5$
Formula weight	354.44
Temperature/K	150.00(10)
Crystal system	monoclinic
Space group	$P2_1/c$
a/Å	9.7120(5)
b/Å	16.2399(15)
c/Å	12.7420(8)
α/°	90
β/°	102.950(6)
γ/°	90
Volume/Å^3	1958.6(2)
Z	4
ρ_{calc}g/cm^3	1.202
μ/mm^{-1}	0.716
F(000)	768.0
Crystal size/mm^3	0.13 × 0.11 × 0.09
Radiation	Cu Kα (λ = 1.54184)
2Θ range for data collection/°	8.964 to 133.196
Index ranges	$-11 \le h \le 11$, $-13 \le k \le 19$, $-15 \le l \le 12$
Reflections collected	8898
Independent reflections	3432 [R_{int} = 0.0592, R_{sigma} = 0.0773]
Data/restraints/parameters	3432/45/270
Goodness-of-fit on F^2	1.059

Final R indexes [I>=2σ (I)] R_1 = 0.0838, wR_2 = 0.2363

Final R indexes [all data] R_1 = 0.1075, wR_2 = 0.2680

Largest diff. peak/hole / e Å^{-3} 0.53/-0.33

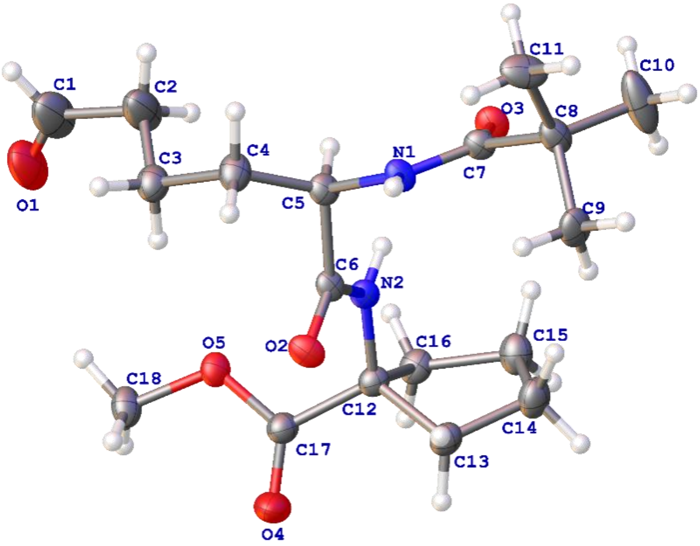

Supplementary Figure 18. X-ray structure of **11b**. Ellipsoids show 50% probability levels.

3.10.2 Bound length [Å] and Angles [°] of 11b

O1-C1	1.187(12)	C12-C13	1.530(4)	C7-C8-C11	108.3(3)
N1-C5	1.465(3)	C12-C17	1.526(4)	C7-C8-C10B	109.8(6)
N1-C7	1.331(4)	C16-C15	1.525(5)	C7-C8-C11B	115.3(5)
O3-C7	1.239(3)	C15-C14	1.515(5)	C9-C8-C11	107.6(4)
O2-C6	1.231(3)	C14-C13	1.514(5)	C10-C8-C7	108.9(4)
O4-C17	1.189(4)			C10-C8-C9	109.5(6)
O5-C17	1.328(4)	C7-N1-C5	122.5(2)	C10-C8-C11	112.5(6)
O5-C18	1.436(4)	C17-O5-C18	116.5(3)	C10B-C8-C11B	108.1(9)
O8-C1	0.95(2)	C6-N2-C12	122.0(2)	C9B-C8-C7	105.5(6)
N2-C6	1.332(4)	N1-C5-C4	111.8(2)	C9B-c8-C10B	108.6(10)
N2-C12	1.457(4)	N1-C5-C6	107.9(2)	C9B-C8-C11B	109.3(9)
C5-C4	1.509(5)	C4-C5-C6	111.7(3)	N2-C12-C16	108.3(2)
C5-C6	1.533(4)	C5-C4-C3	114.5(3)	N2-C12-C13	111.4(2)
C4-C3	1.526(5)	C2-C3-C4	113.3(3)	N2-C12-C17	111.0(3)
C3-C2	1.451(6)	C3-C2-C1	111.8(4)	C13-C12-C16	101.5(3)
C2-C1	1.496(7)	O1-C1-C2	130.5(9)	C17-C12-C16	110.4(2)
C7-C8	1.526(4)	O8-C1-C2	127.1(15)	C17-C12-C13	113.8(3)
C8-C9	1.534(6)	O2-C6-N2	122.4(3)	C15-C16-C12	104.1(3)
C8-C10	1.523(7)	O2-C6-C5	121.7(3)	C14-C15-C16	106.6(3)
C8-C11	1.536(8)	N2-C6-C5	115.9(2)	C13-C14-C15	106.4(3)
C8-C10B	1.529(11)	N1-C7-C8	118.1(2)	C14-C13-C12	104.3(3)
C8-C9B	1.512(13)	O3-C7-N1	121.2(3)	O4-C17-O5	123.2(3)
C8-C11B	1.538(12)	O3-C7-C8	120.7(3)	O4-C17-C12	124.5(3)
C12-C16	1.542(4)	C7-C8-C9	110.1(3)	O5-C17-C12	112.2(3)

3.11 NMR Spectra

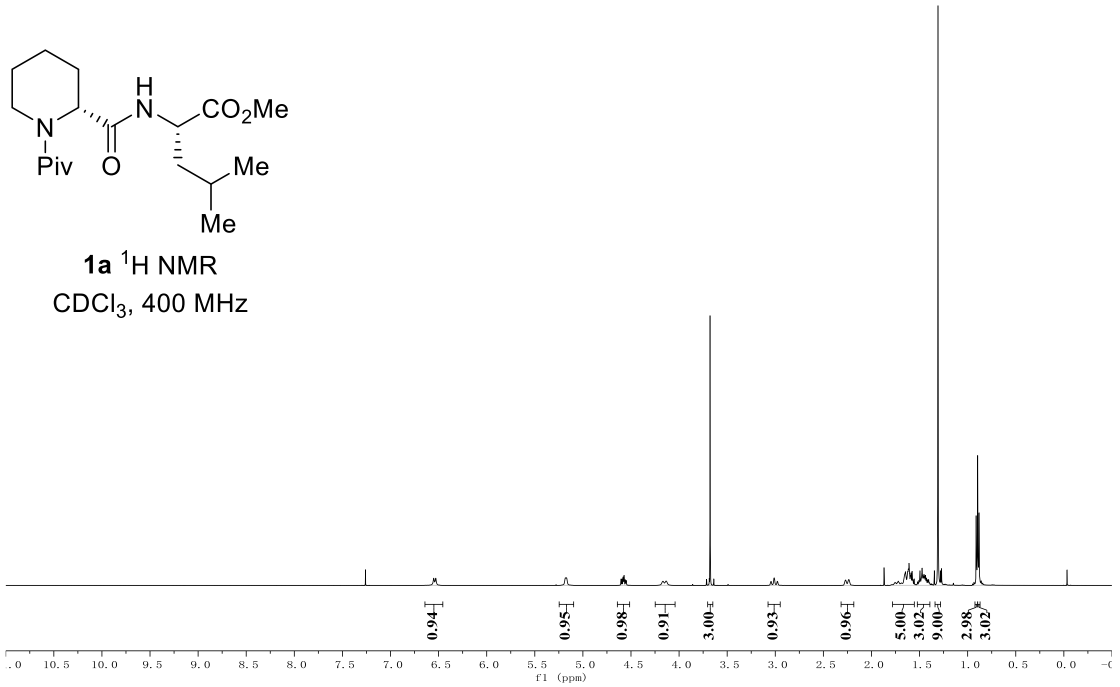

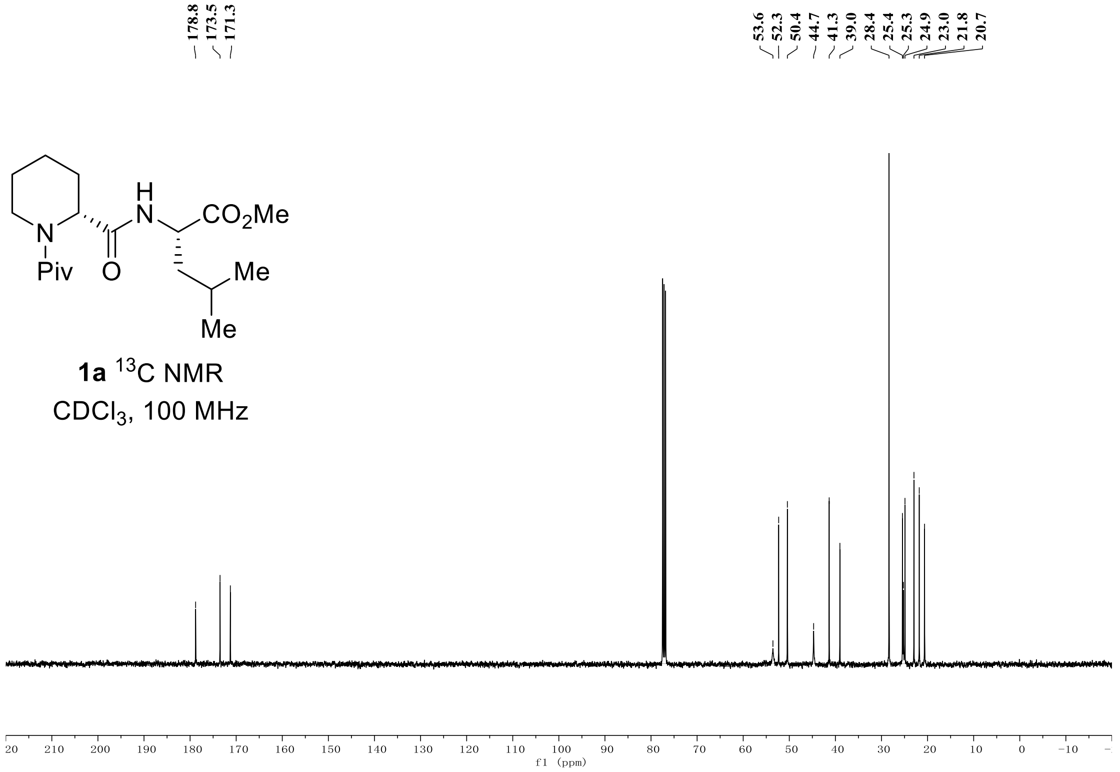

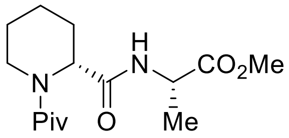

2a ^{1}H NMR

$CDCl_3$, 400 MHz

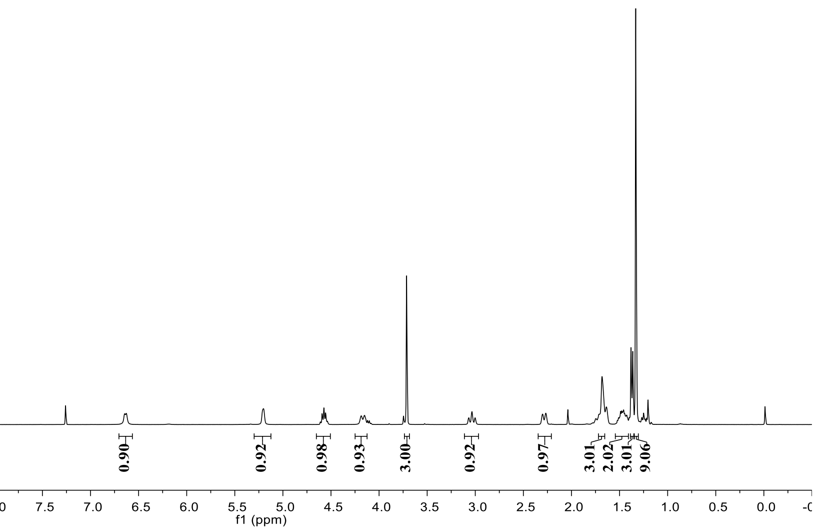

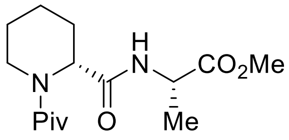

2a ^{13}C NMR

$CDCl_3$, 100 MHz

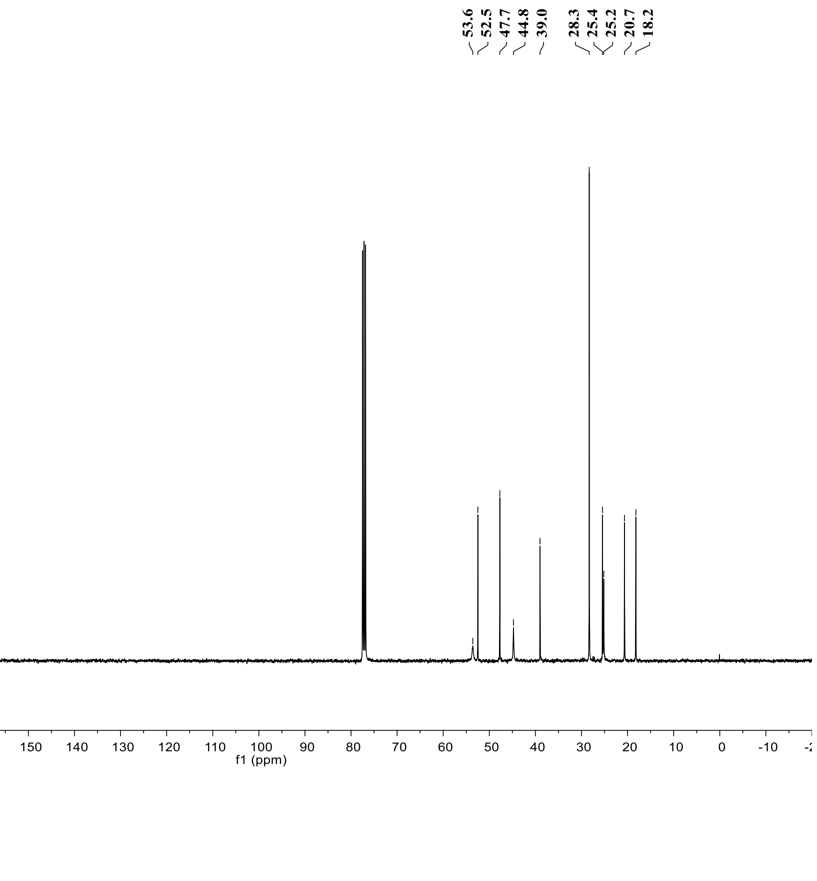

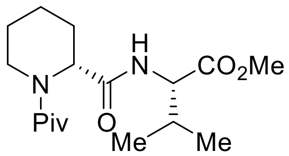

3a ^{1}H NMR
$CDCl_3$, 400 MHz

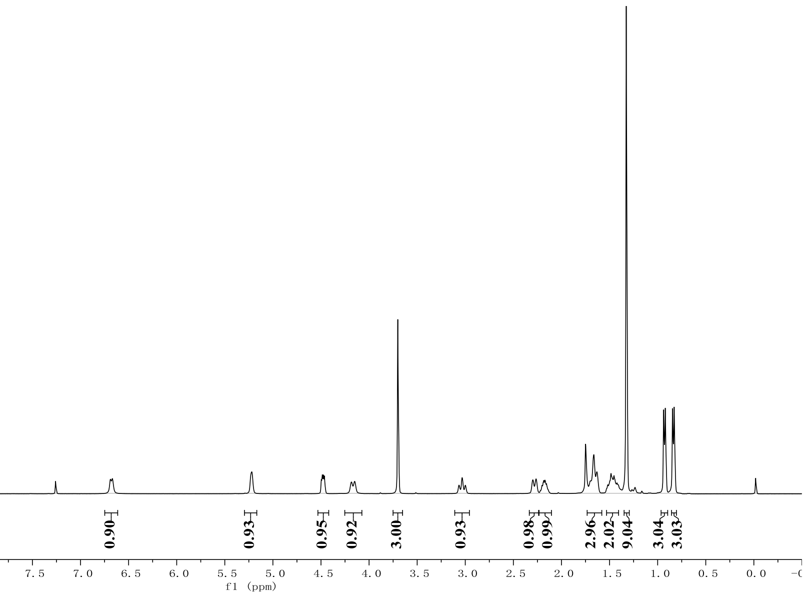

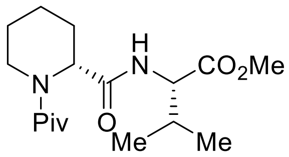

3a ^{13}C NMR
$CDCl_3$, 100 MHz

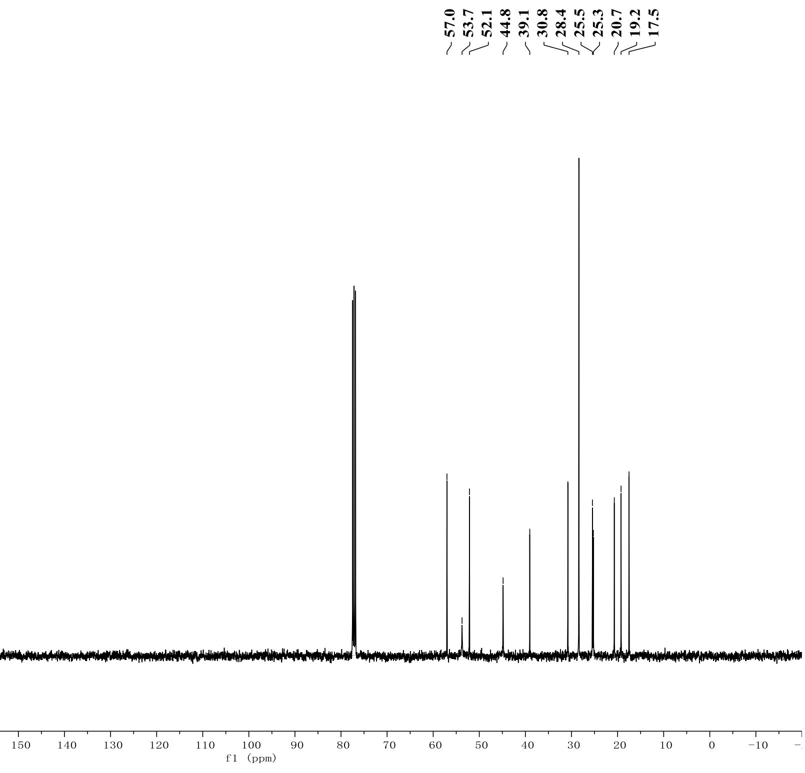

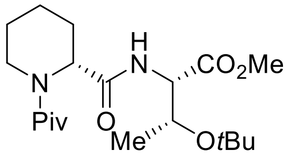

4a ^{1}H NMR
$CDCl_3$, 400 MHz

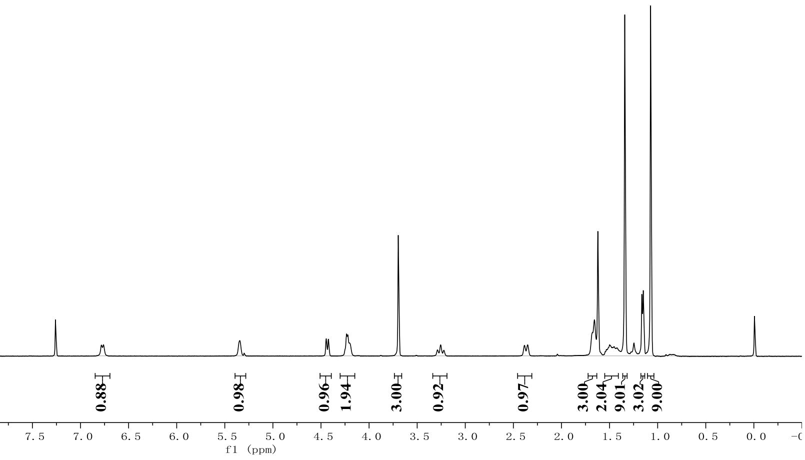

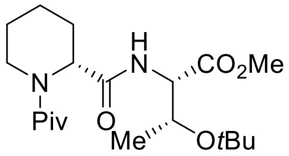

4a ^{13}C NMR
$CDCl_3$, 100 MHz

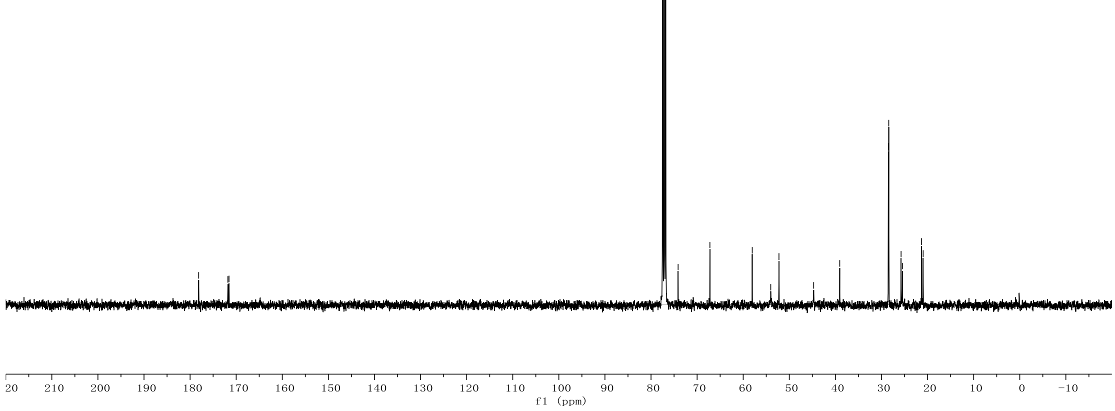

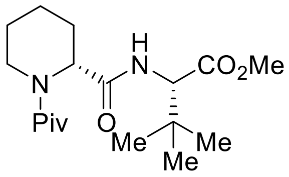

5a ^{1}H NMR
$CDCl_3$, 400 MHz

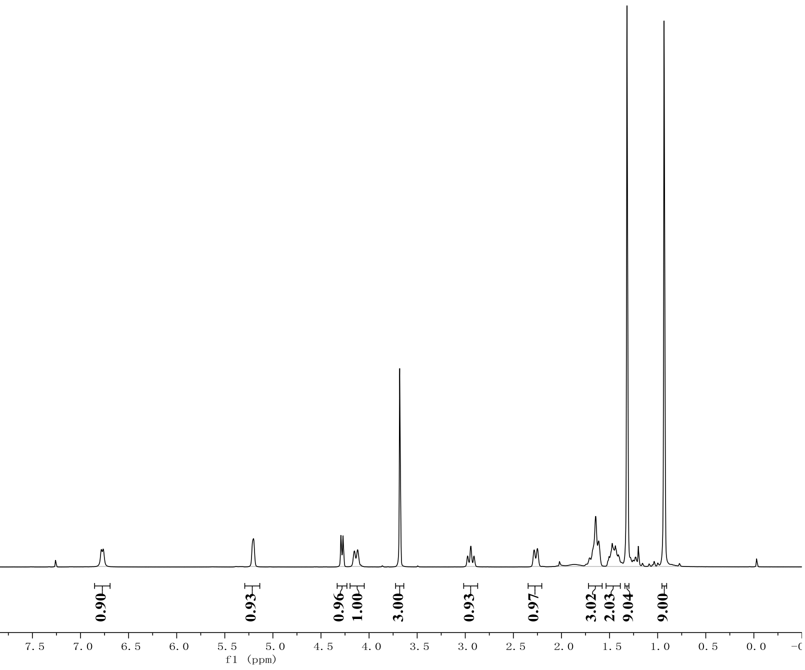

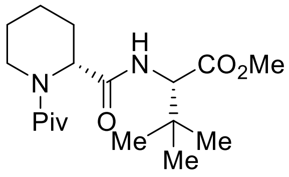

5a ^{13}C NMR
$CDCl_3$, 100 MHz

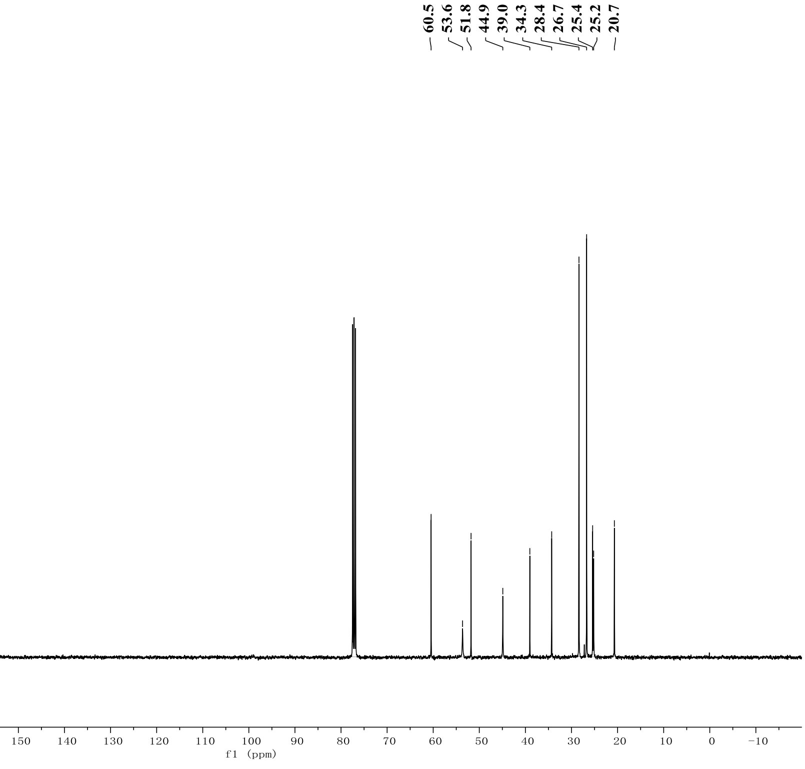

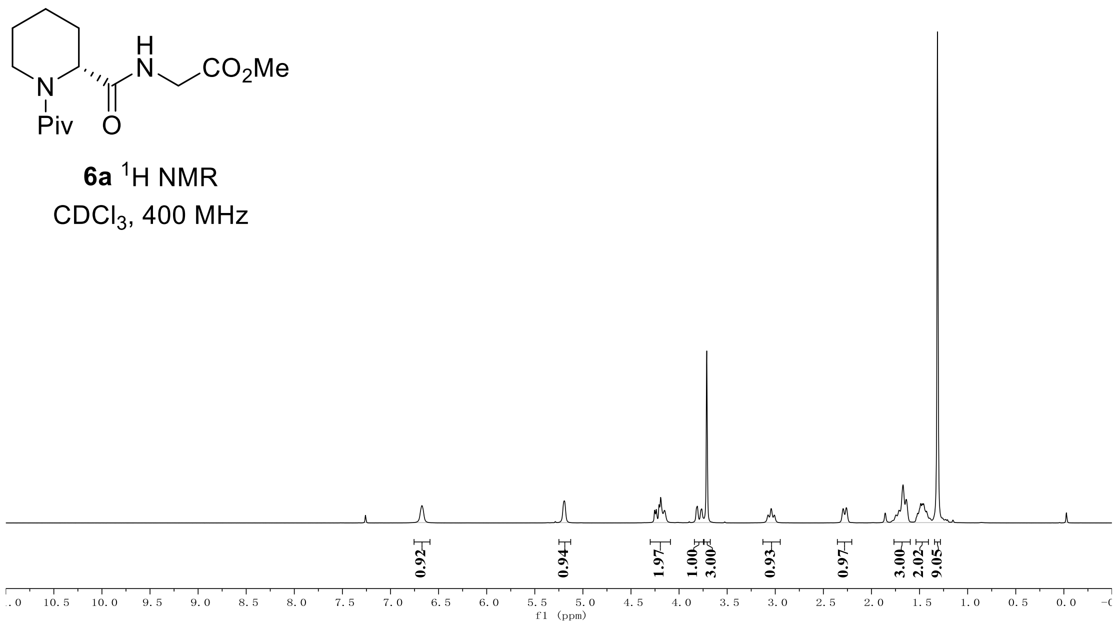
N
H
CO_2Me
Piv
O
6a ^{1}H NMR
$CDCl_3$, 400 MHz
0.92
0.94
1.97
1.00
3.00
0.93
0.97
3.00
2.02
9.05
f1 (ppm)

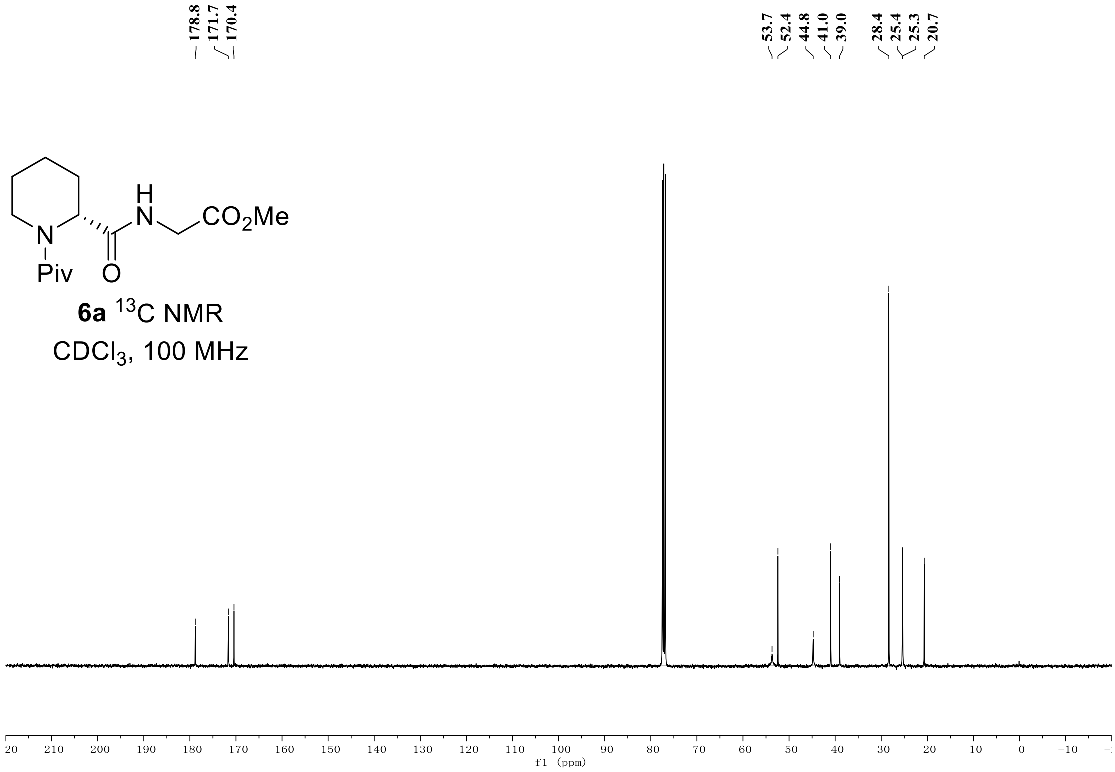
178.8
171.7
170.4
53.7
52.4
44.8
41.0
39.0
28.4
25.4
25.3
20.7
N
H
CO_2Me
Piv
O
6a ^{13}C NMR
$CDCl_3$, 100 MHz
f1 (ppm)

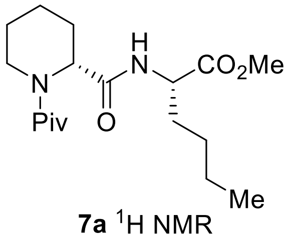

7a ^{1}H NMR
$CDCl_3$, 400 MHz

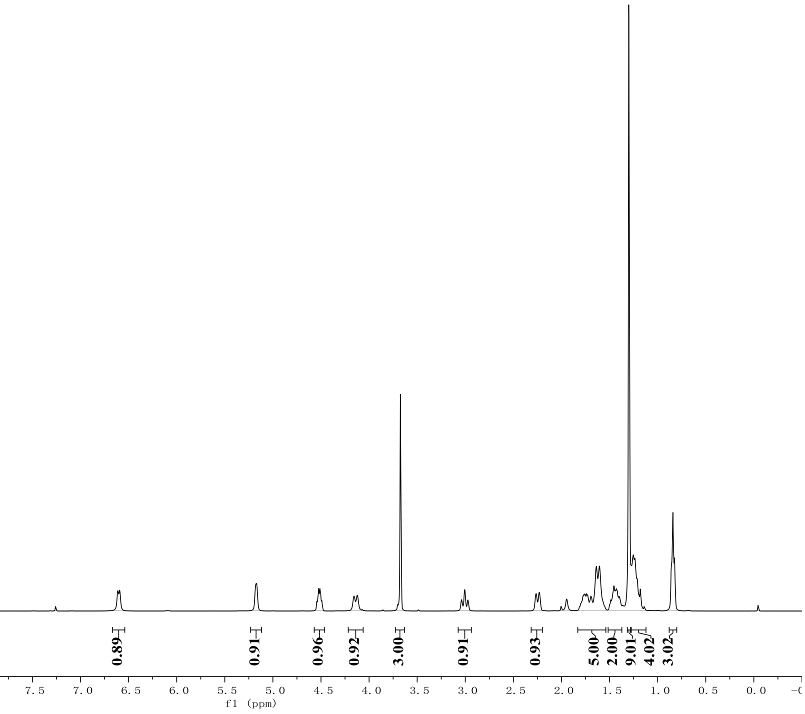

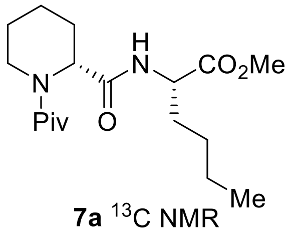

7a ^{13}C NMR
$CDCl_3$, 100 MHz

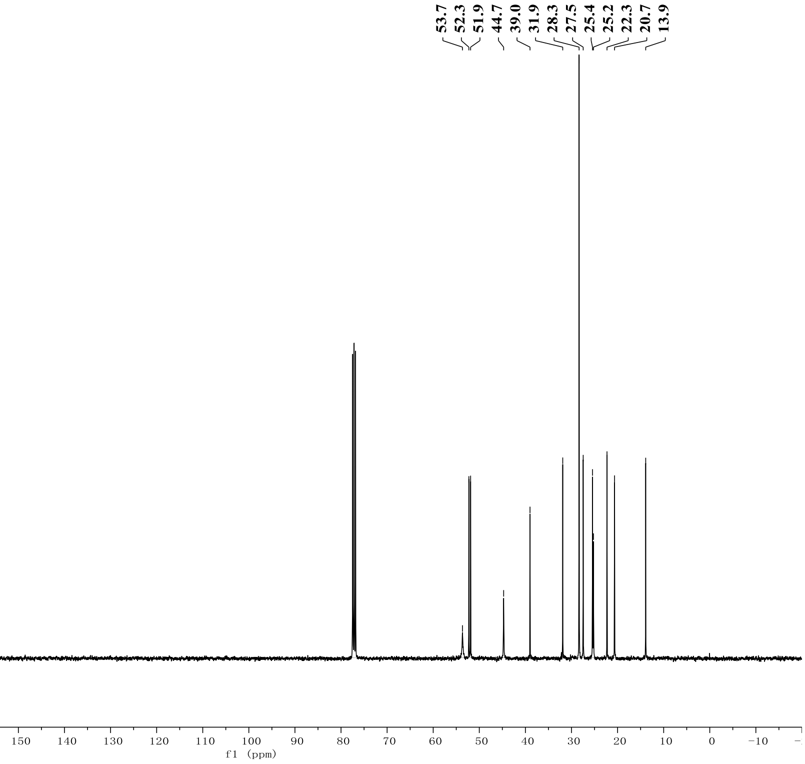

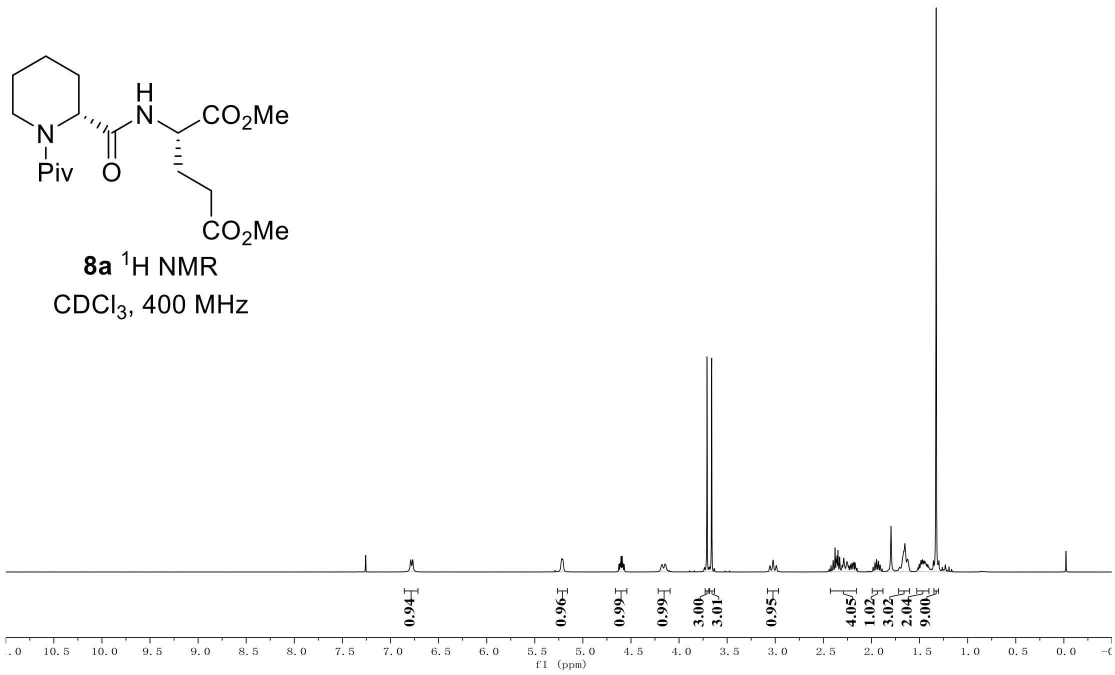
H
N
CO2Me
N
Piv
O
CO2Me
8a 1H NMR
CDCl3, 400 MHz
0.94
0.96
0.99
0.99
3.00
3.01
0.95
4.05
1.02
3.02
2.04
9.00
f1 (ppm)

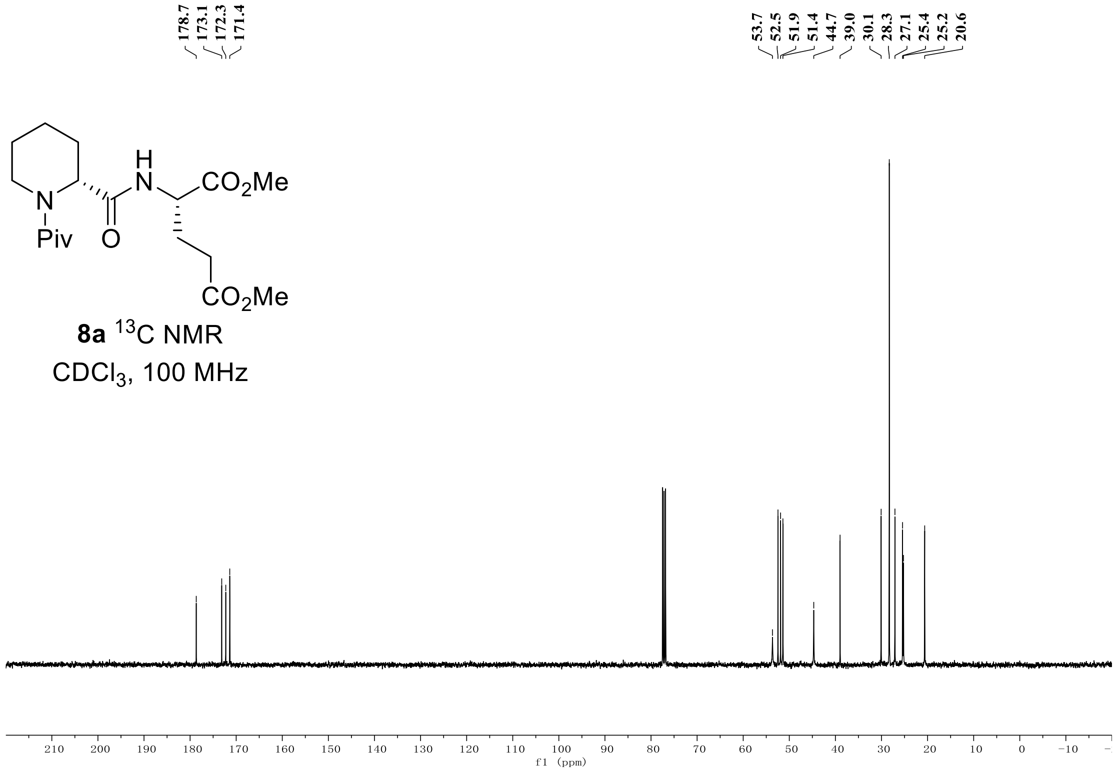
178.7
173.1
172.3
171.4
53.7
52.5
51.9
51.4
44.7
39.0
30.1
28.3
27.1
25.4
25.2
20.6
H
N
CO2Me
N
Piv
O
CO2Me
8a 13C NMR
CDCl3, 100 MHz
f1 (ppm)

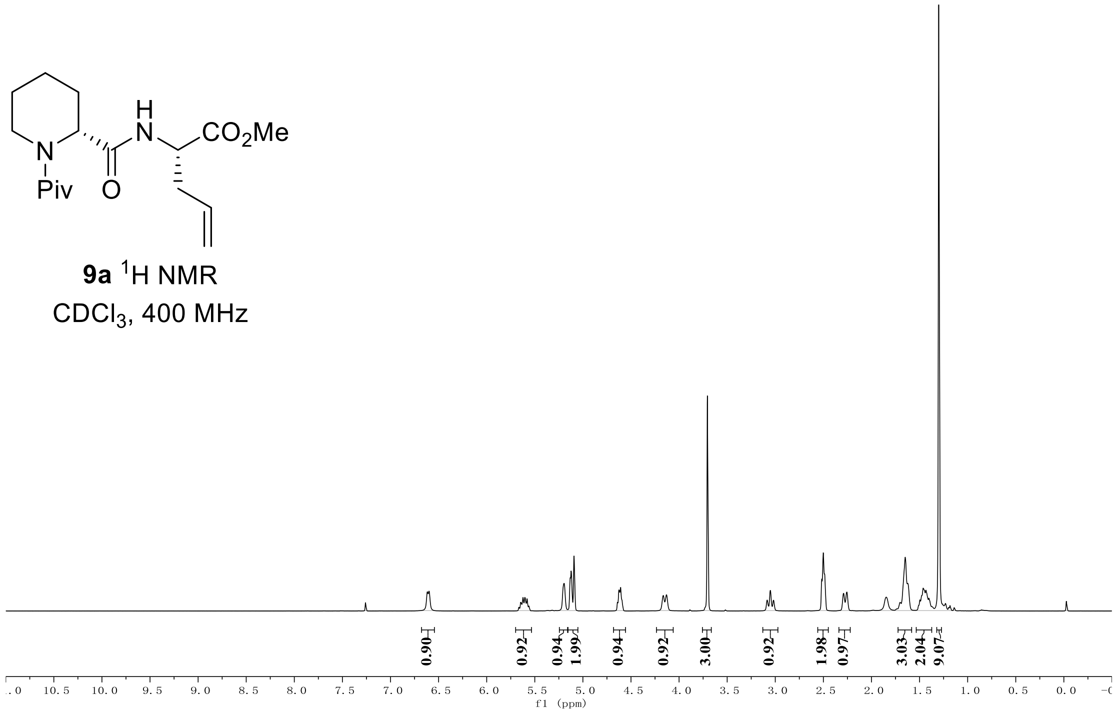

9a ^{1}H NMR
$CDCl_3$, 400 MHz

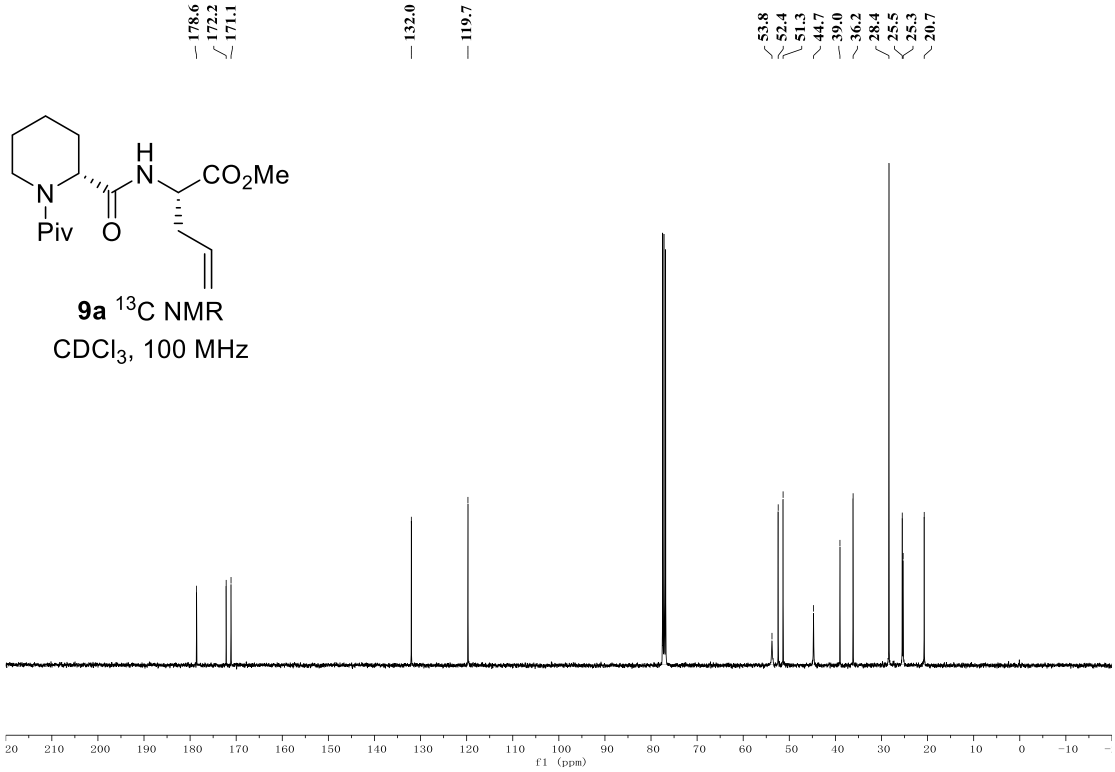

9a ^{13}C NMR
$CDCl_3$, 100 MHz

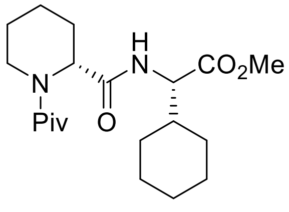

10a ^{1}H NMR
$CDCl_3$, 400 MHz

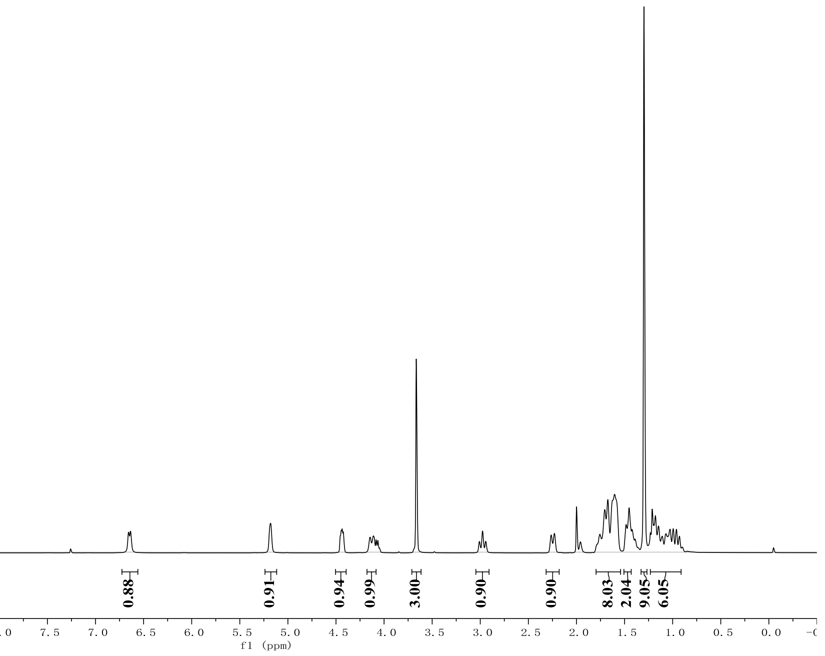

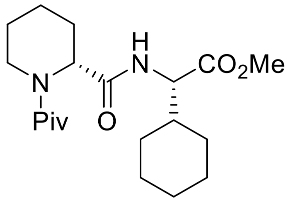

10a ^{13}C NMR
$CDCl_3$, 100 MHz

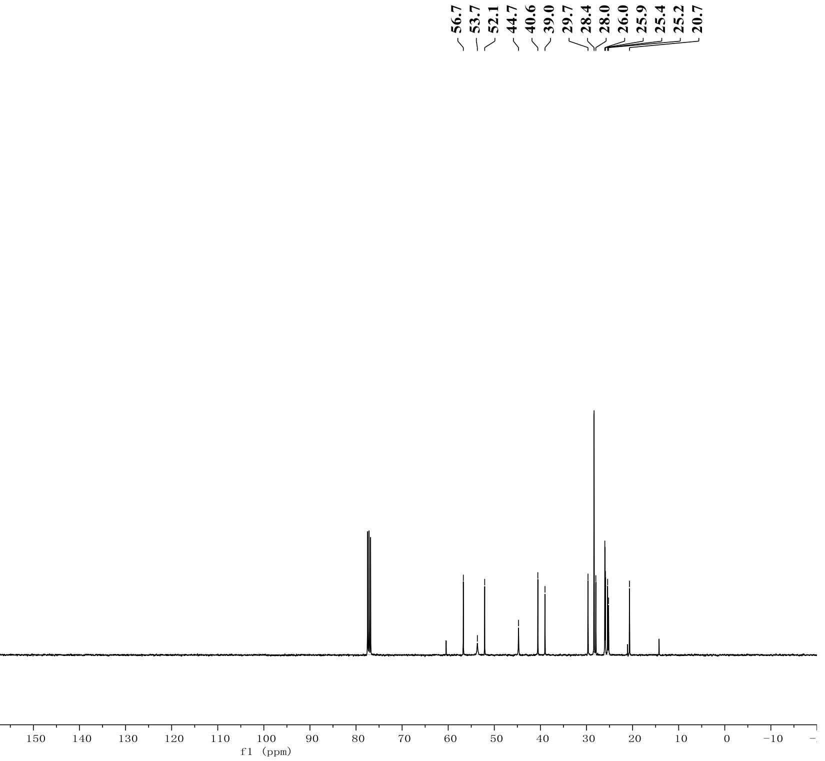

11a ^{1}H NMR
$CDCl_3$, 400 MHz

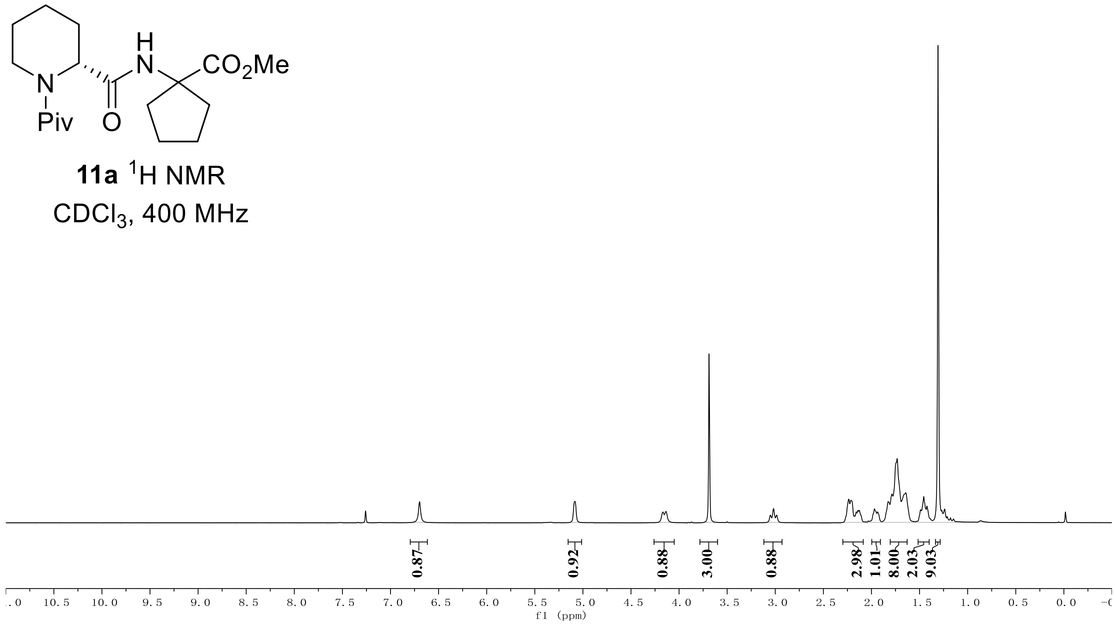

11a ^{13}C NMR
$CDCl_3$, 100 MHz

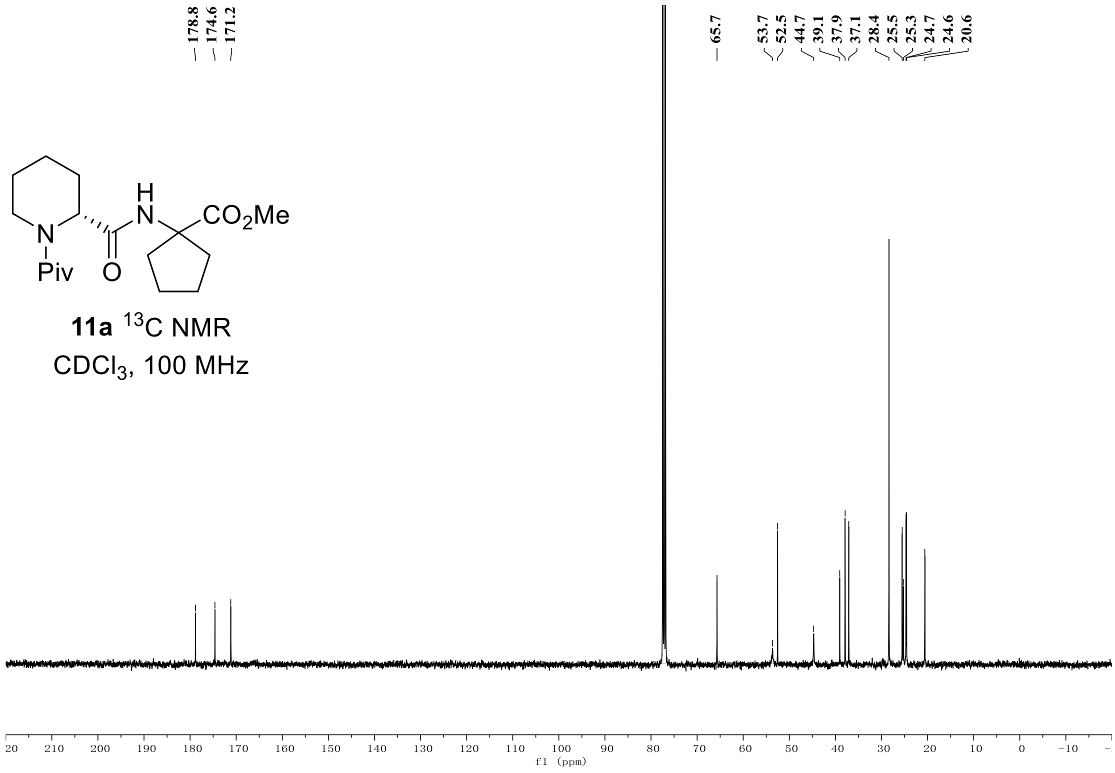

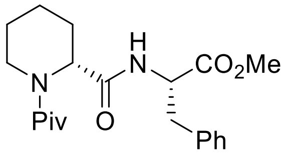

12a ^{1}H NMR
$CDCl_3$, 400 MHz

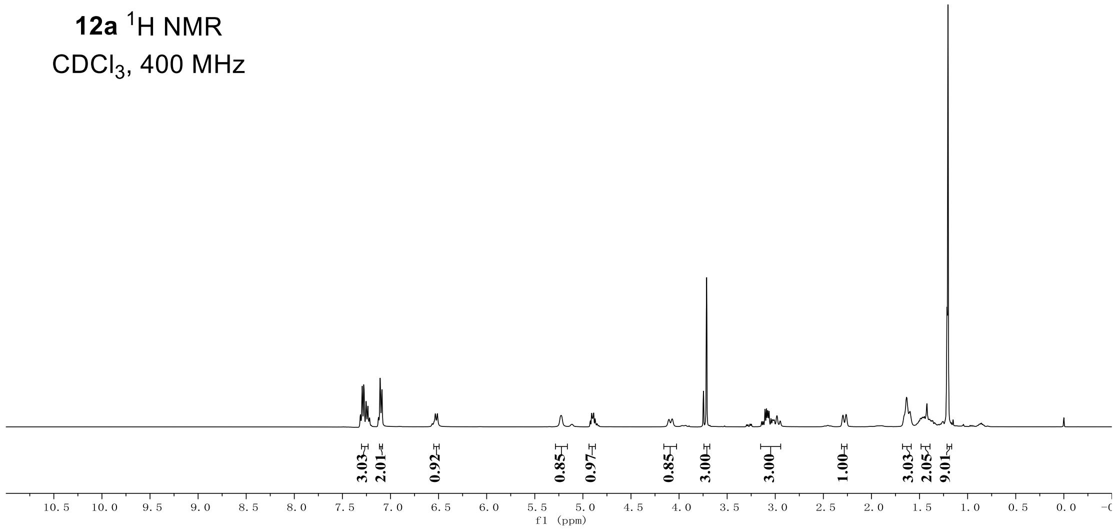

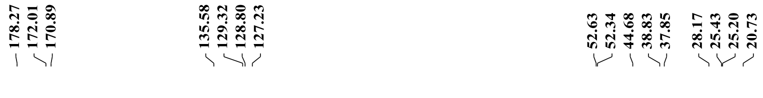

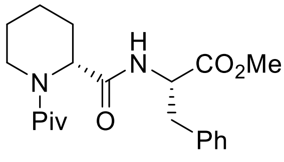

12a ^{13}C NMR
$CDCl_3$, 100 MHz

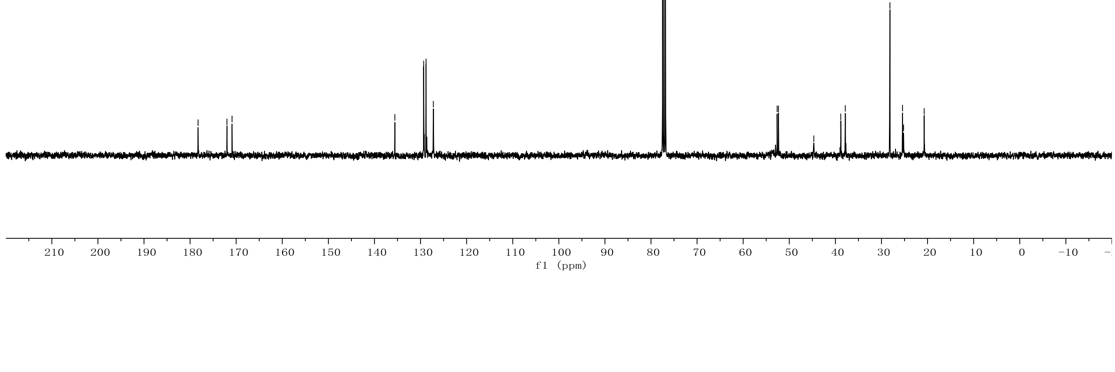

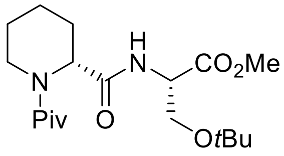

13a ^{1}H NMR
$CDCl_3$, 400 MHz

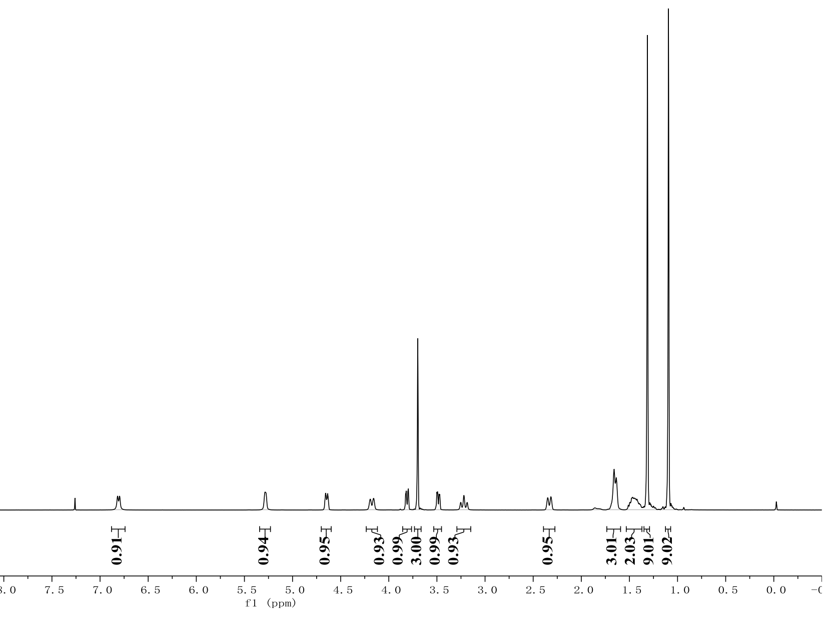

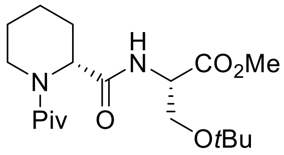

13a ^{13}C NMR
$CDCl_3$, 100 MHz

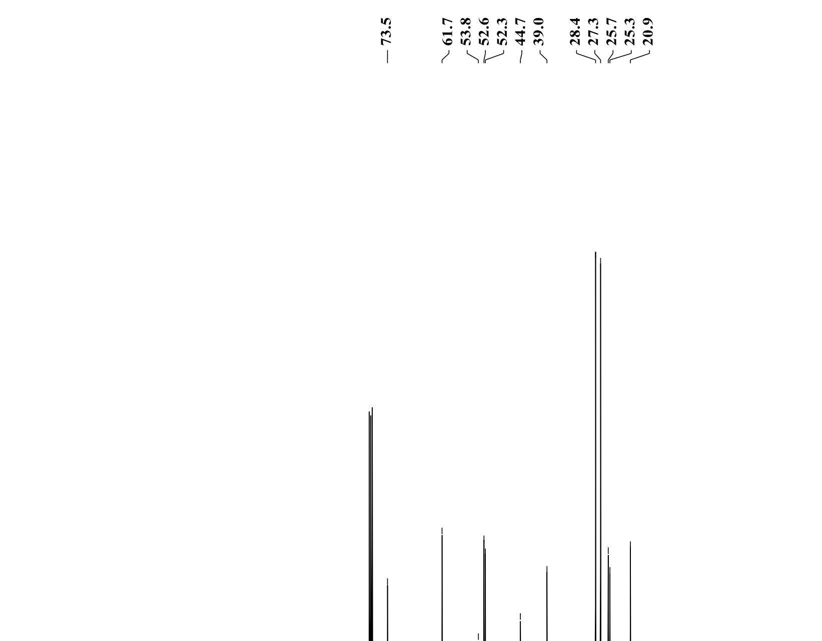

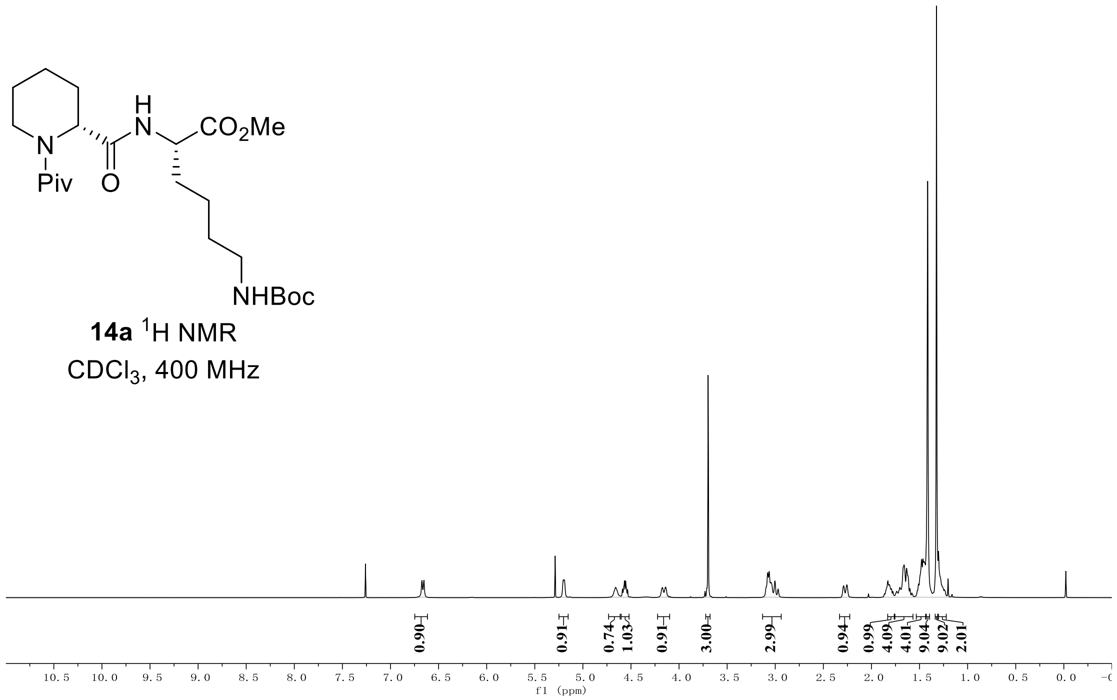
CO2Me
N
H
N
Piv
O
NHBoc
14a 1H NMR
CDCl3, 400 MHz
0.90
0.91
0.74
1.03
0.91
3.00
2.99
0.94
0.99
4.09
4.01
9.04
9.02
2.01
f1 (ppm)

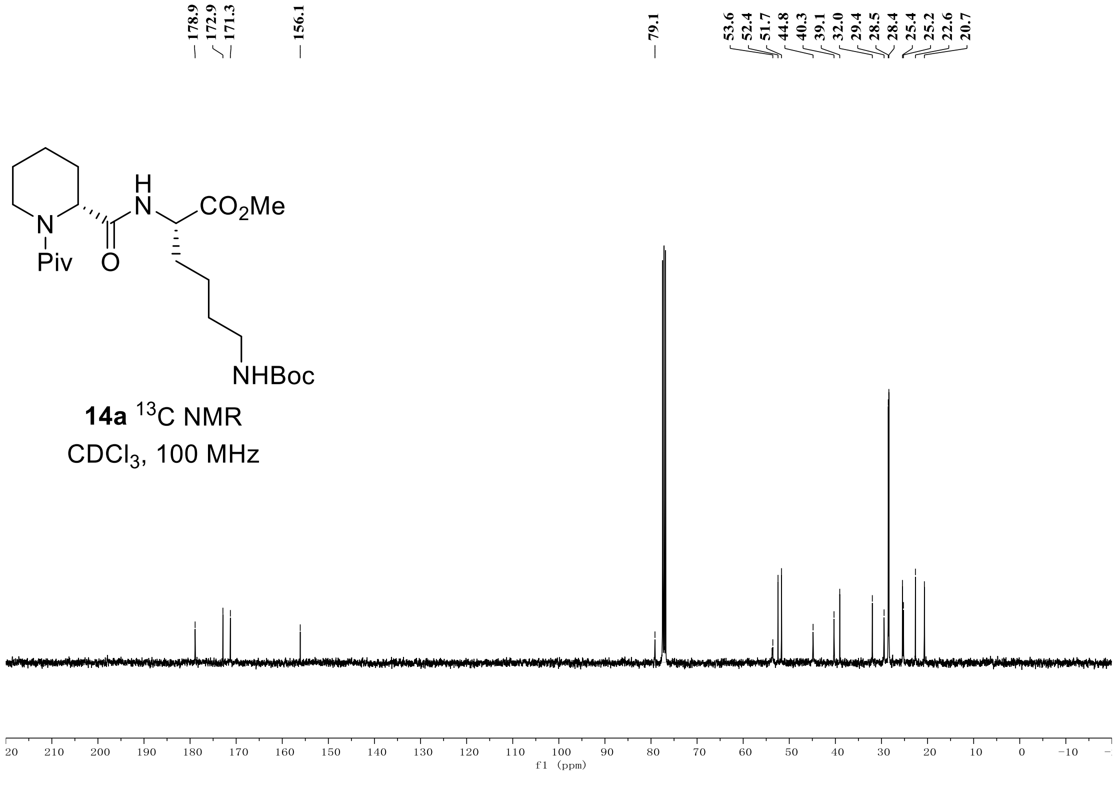
178.9
172.9
171.3
156.1
79.1
53.6
52.4
51.7
44.8
40.3
39.1
32.0
29.4
28.5
28.4
25.4
25.2
22.6
20.7
CO2Me
N
H
N
Piv
O
NHBoc
14a 13C NMR
CDCl3, 100 MHz
f1 (ppm)

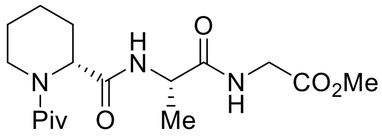

15a ^{1}H NMR
$CDCl_3$, 400 MHz

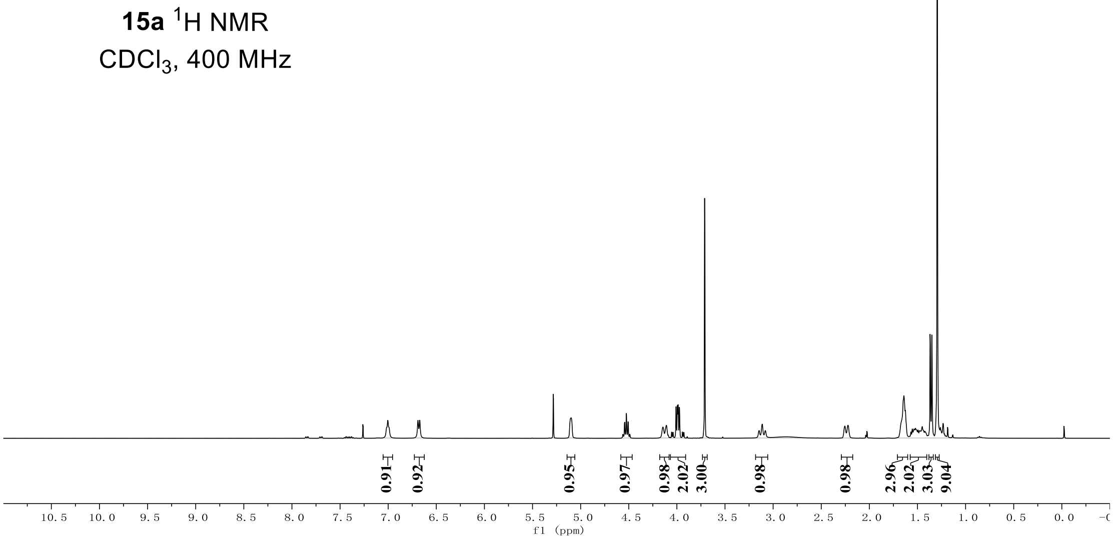

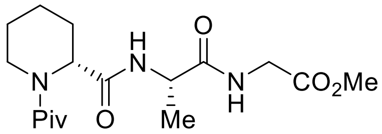

15a ^{13}C NMR
$CDCl_3$, 100 MHz

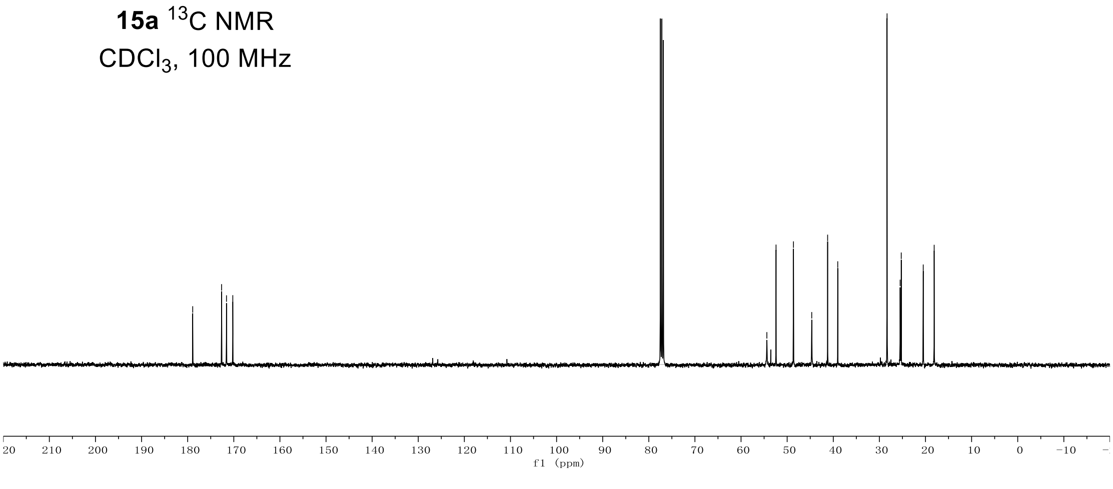

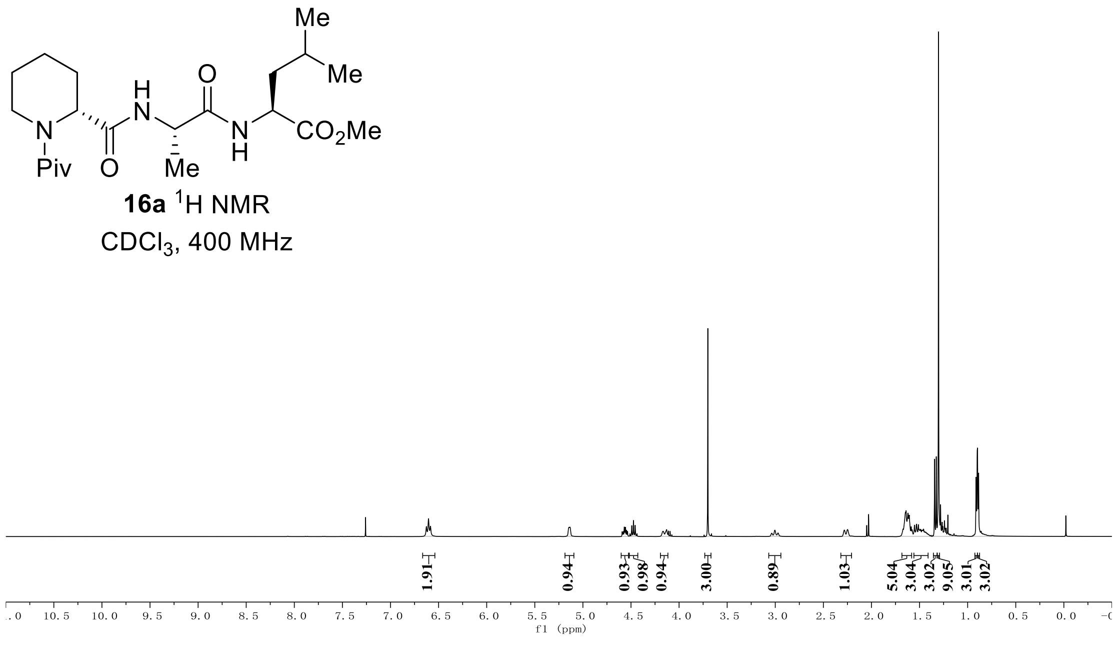
Me
Me
H
N
O
N
H
CO2Me
N
Piv
O
Me
16a 1H NMR
CDCl3, 400 MHz
1.91
0.94
0.93
0.98
0.94
3.00
0.89
1.03
5.04
3.04
3.02
9.05
3.01
3.02
f1 (ppm)

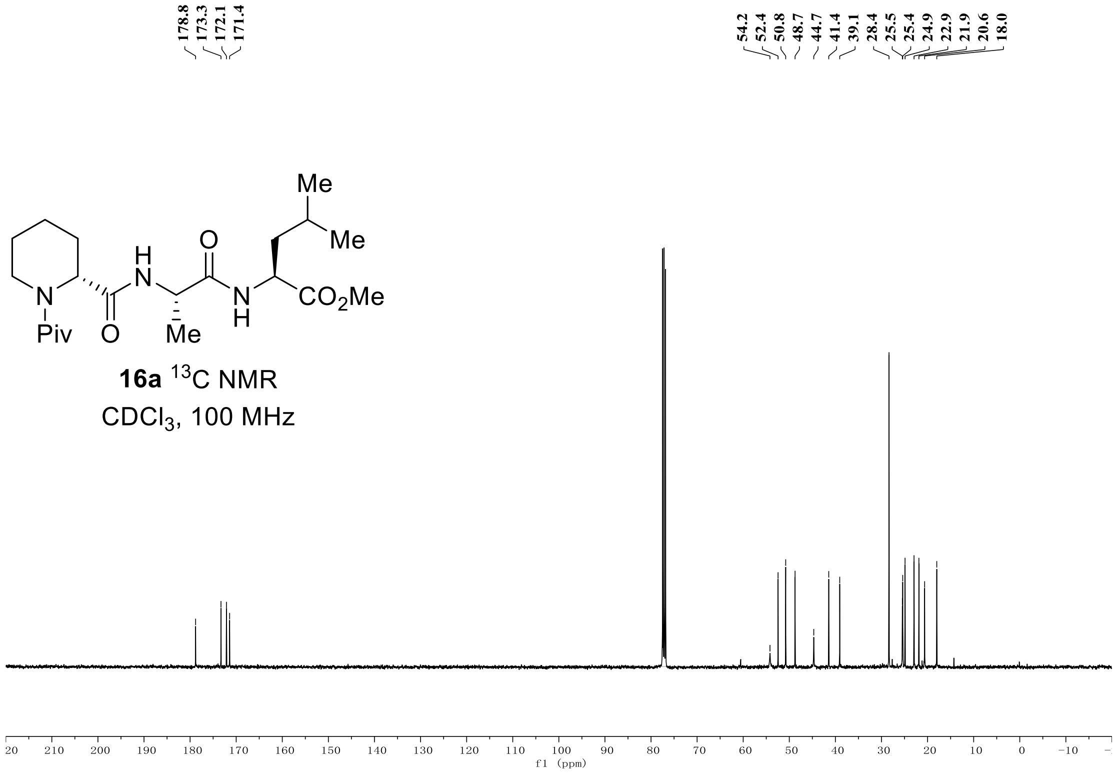
178.8
173.3
172.1
171.4
54.2
52.4
50.8
48.7
44.7
41.4
39.1
28.4
25.5
25.4
24.9
22.9
21.9
20.6
18.0
Me
Me
H
N
O
N
H
CO2Me
N
Piv
O
Me
16a 13C NMR
CDCl3, 100 MHz
f1 (ppm)

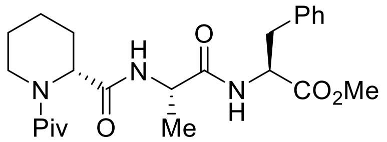

17a ^{1}H NMR
$CDCl_3$, 400 MHz

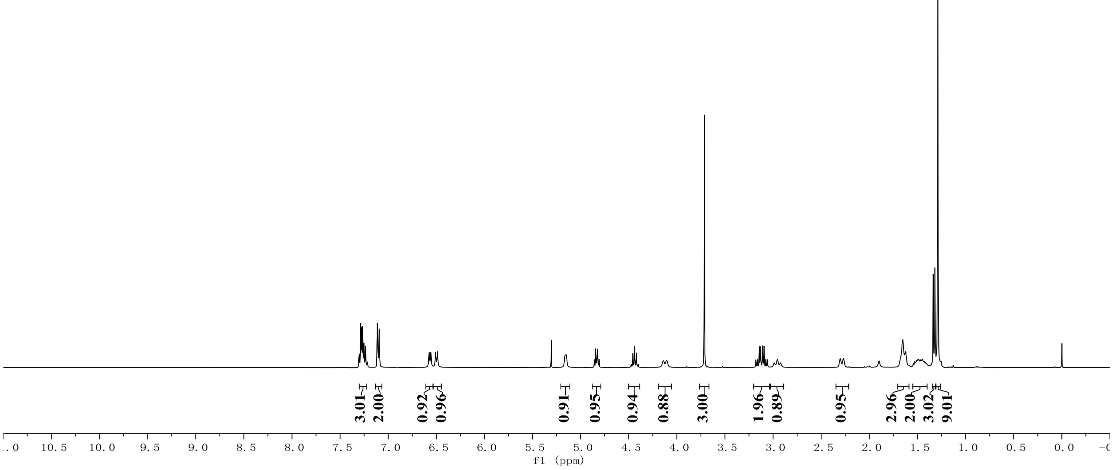

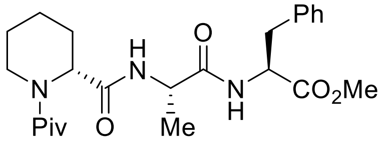

17a ^{13}C NMR
$CDCl_3$, 100 MHz

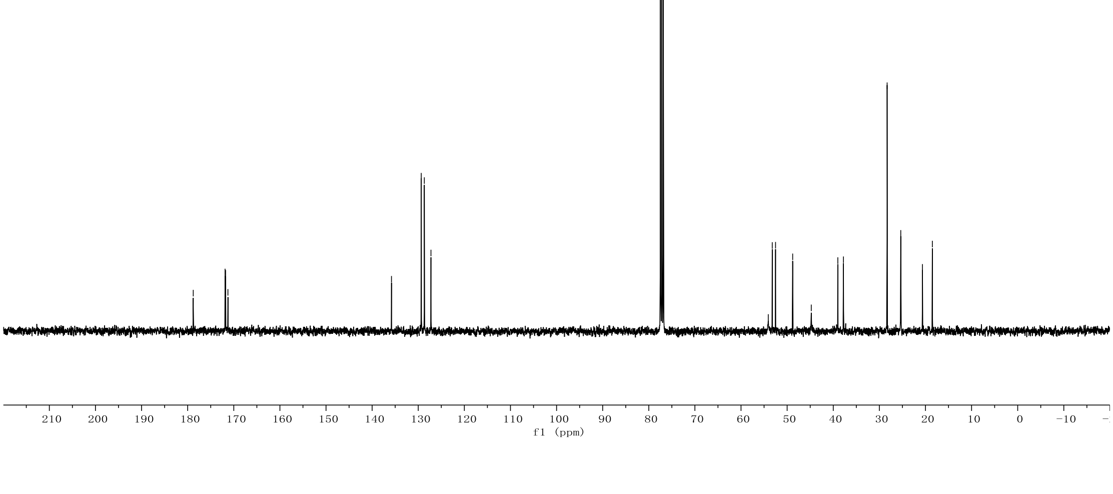

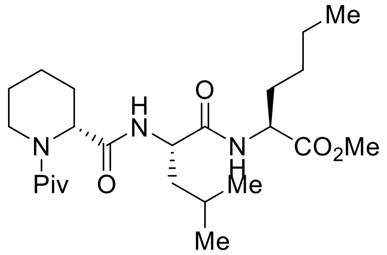

18a ^{1}H NMR
$CDCl_3$, 400 MHz

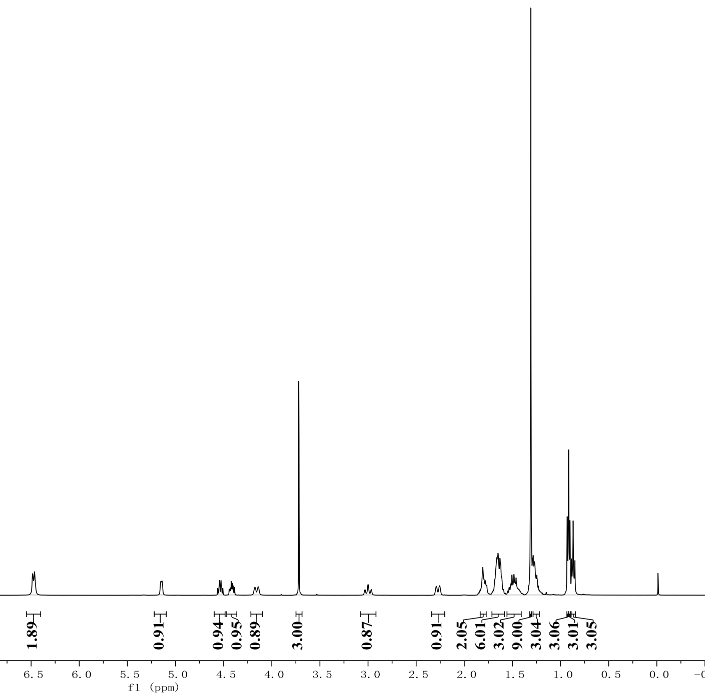

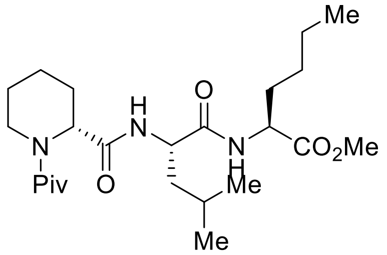

18a ^{13}C NMR
$CDCl_3$, 100 MHz

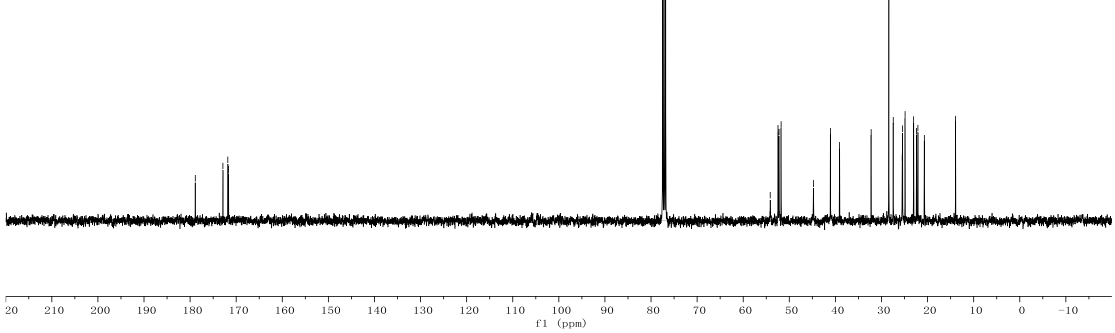

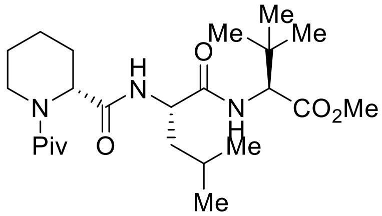

19a ^{1}H NMR

$CDCl_3$, 400 MHz

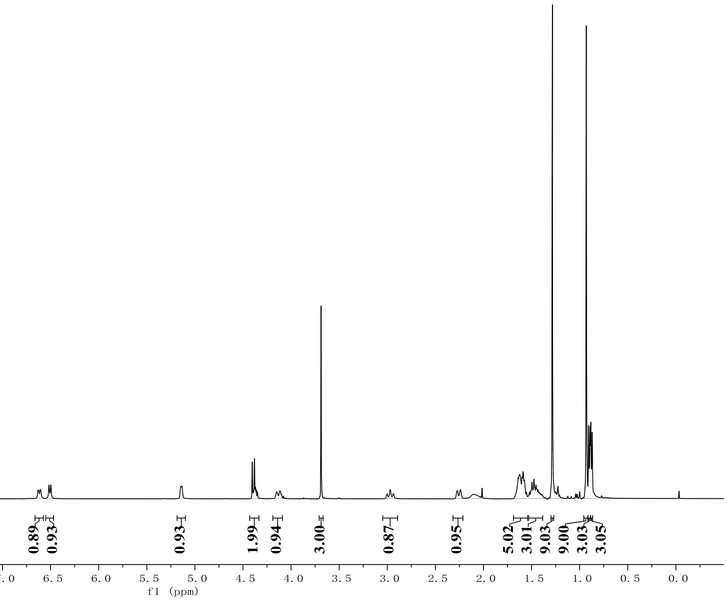

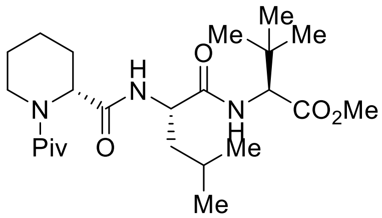

19a ^{13}C NMR

$CDCl_3$, 100 MHz

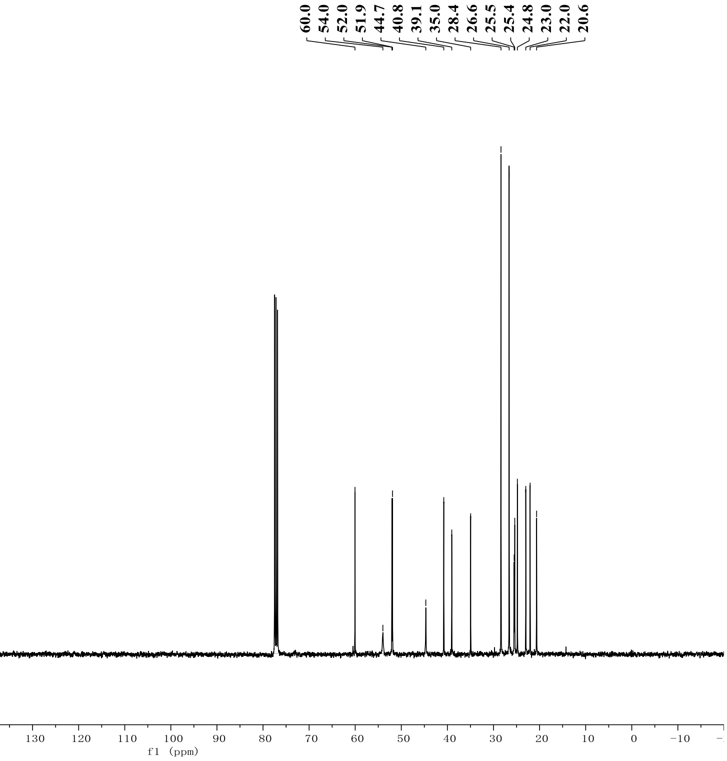

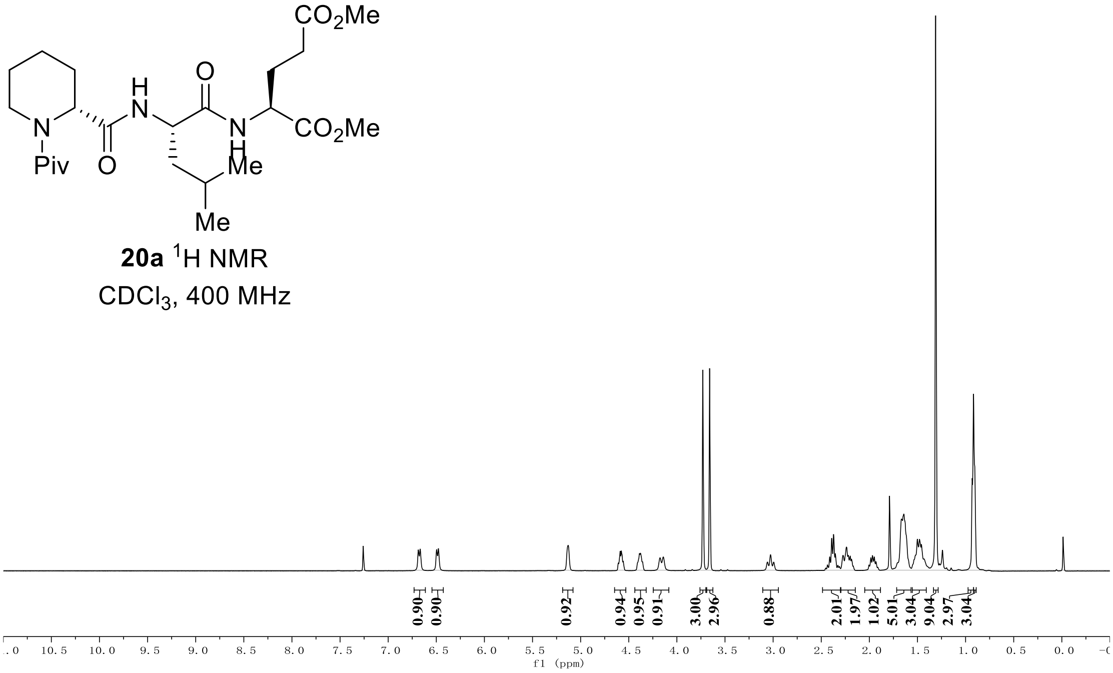
CO2Me
CO2Me
Piv
Me
Me
20a 1H NMR
CDCl3, 400 MHz
0.90
0.90
0.92
0.94
0.95
0.91
3.00
2.96
0.88
2.01
1.97
1.02
5.01
3.04
9.04
2.97
3.04
f1 (ppm)

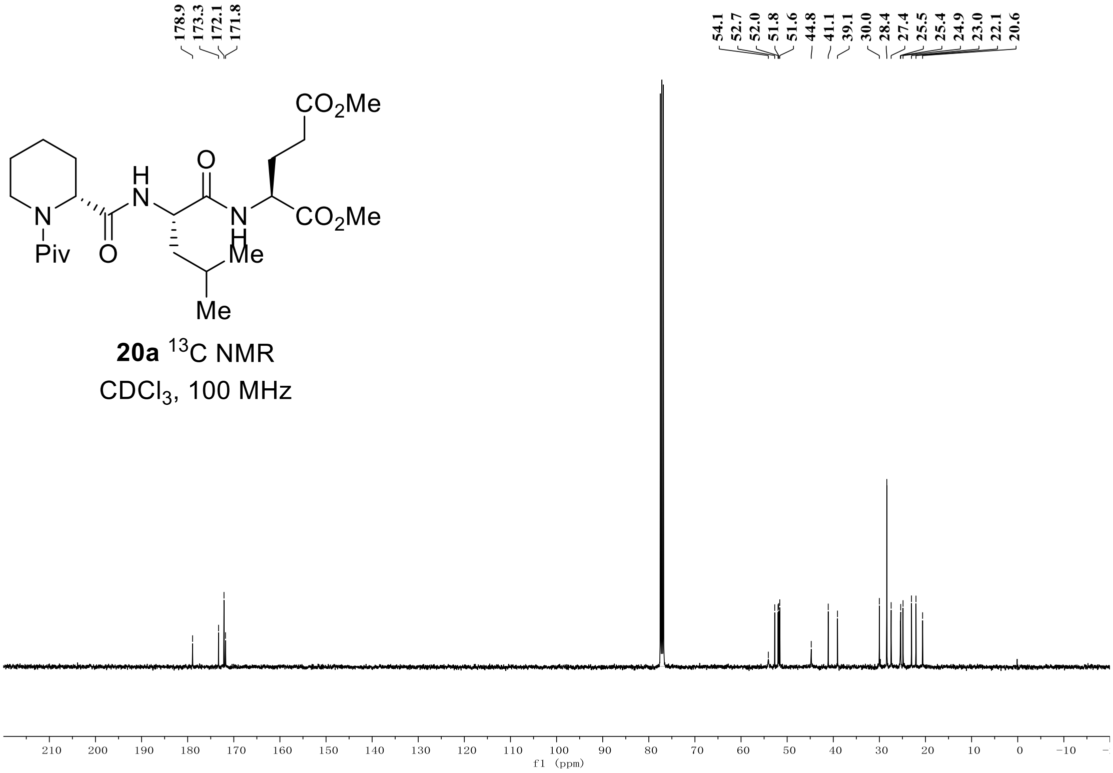
178.9
173.3
172.1
171.8
54.1
52.7
52.0
51.8
51.6
44.8
41.1
39.1
30.0
28.4
27.4
25.5
25.4
24.9
23.0
22.1
20.6
CO2Me
CO2Me
Piv
Me
Me
20a 13C NMR
CDCl3, 100 MHz
f1 (ppm)

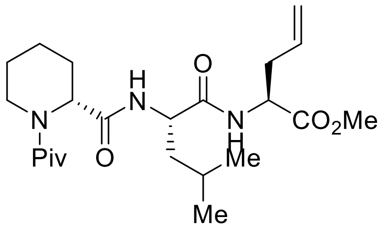

21a ^{1}H NMR
$CDCl_3$, 400 MHz

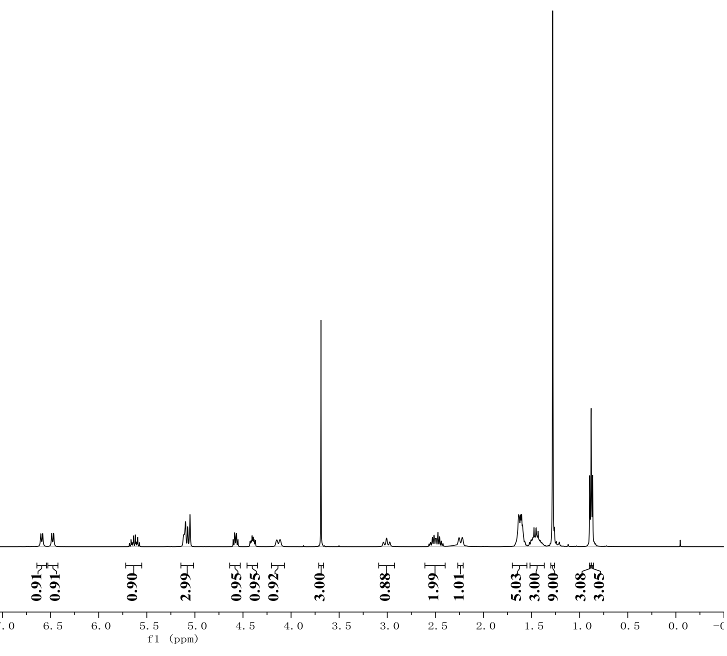

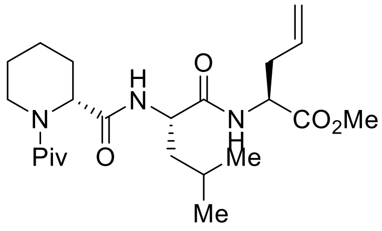

21a ^{13}C NMR
$CDCl_3$, 100 MHz

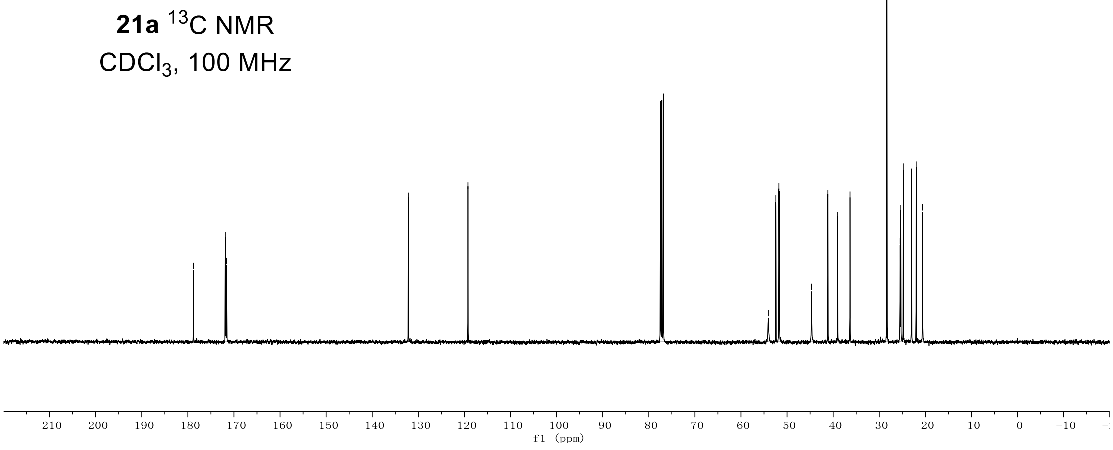

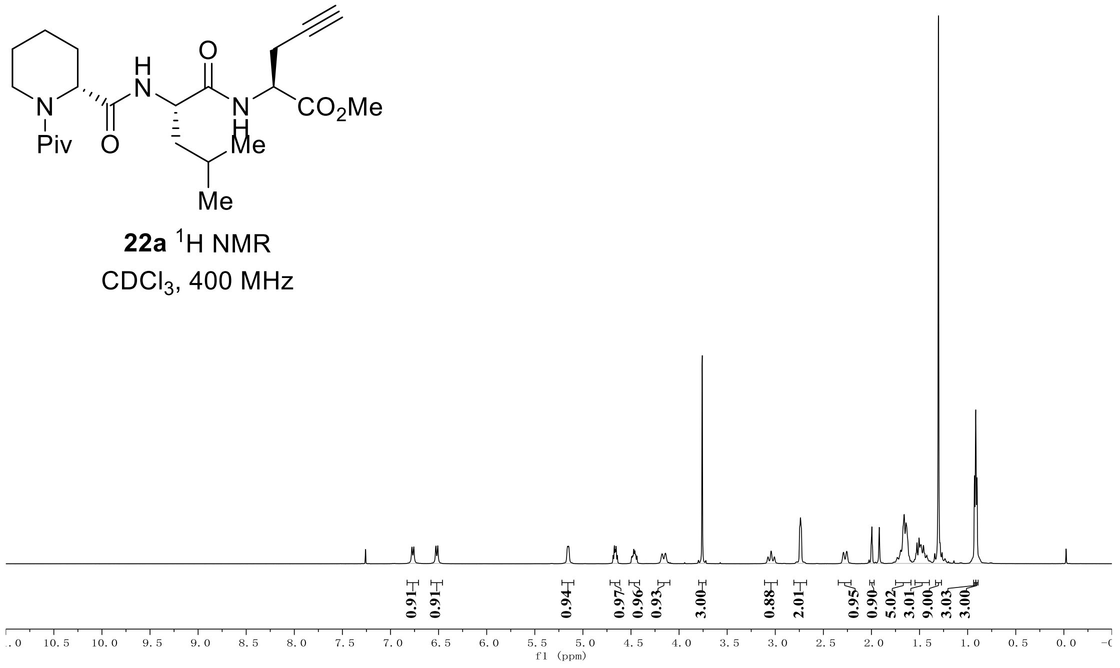
22a 1H NMR
CDCl3, 400 MHz
0.91
0.91
0.94
0.97
0.96
0.93
3.00
0.88
2.01
0.95
0.90
5.02
3.01
9.00
3.03
3.00
f1 (ppm)

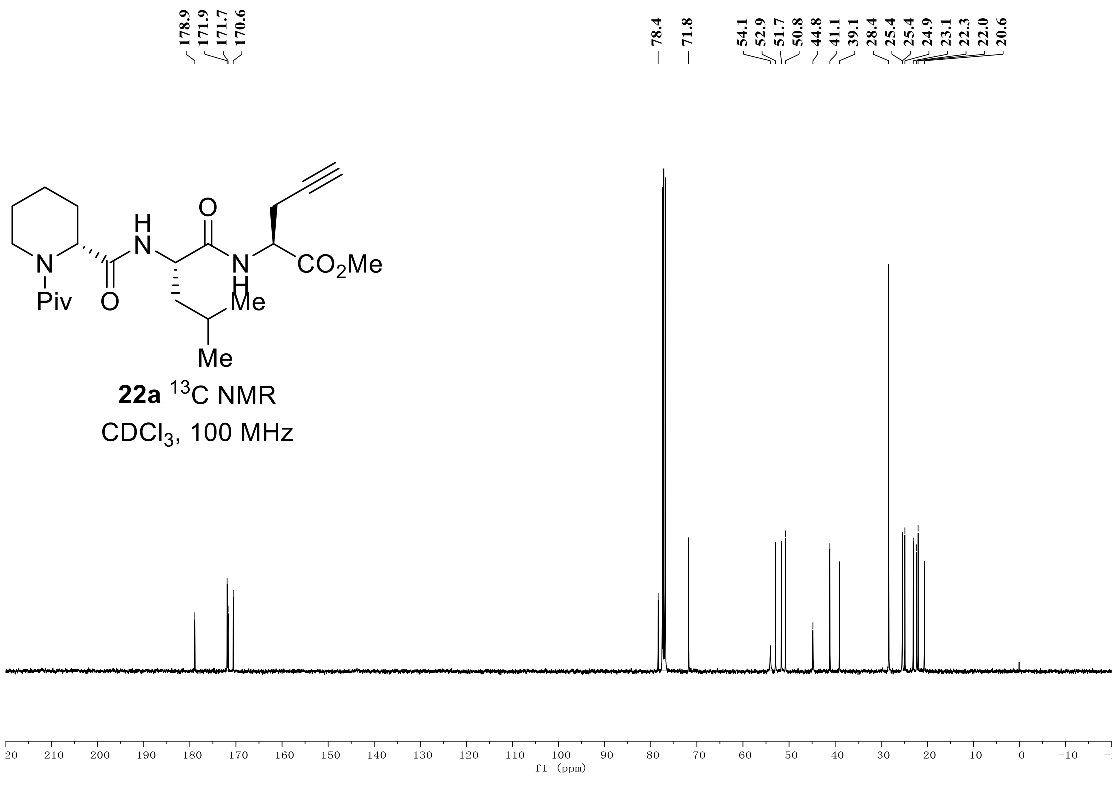
178.9
171.9
171.7
170.6
78.4
71.8
54.1
52.9
51.7
50.8
44.8
41.1
39.1
28.4
25.4
25.4
24.9
23.1
22.3
22.0
20.6
22a 13C NMR
CDCl3, 100 MHz
f1 (ppm)

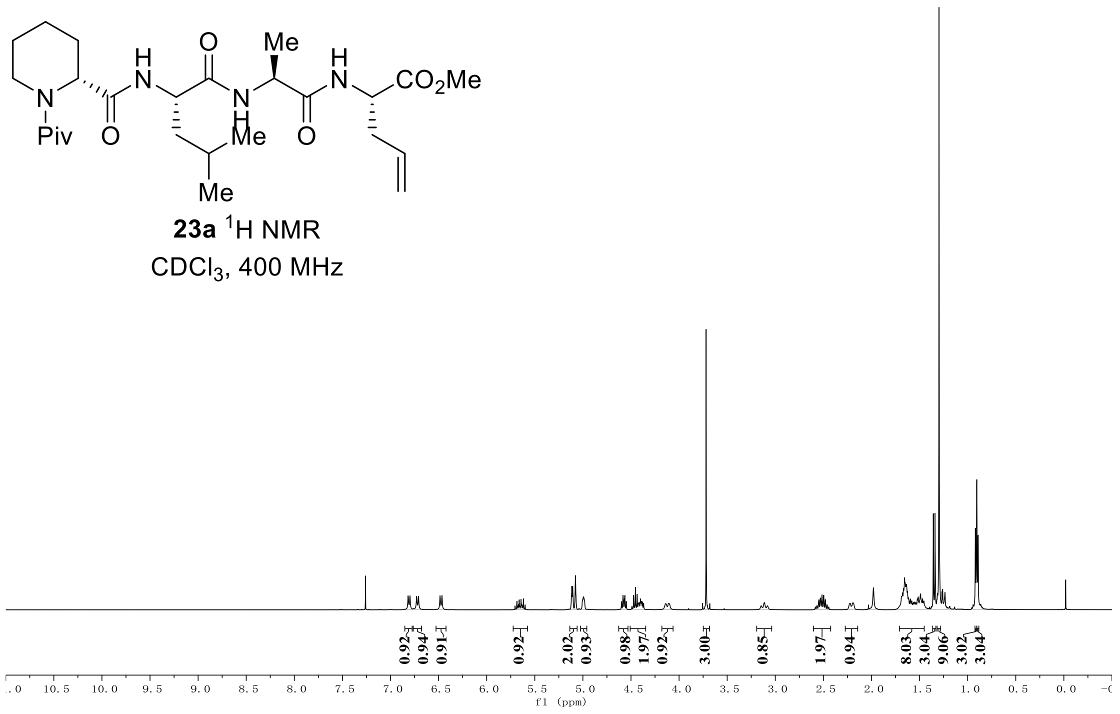

Piv
Me
CO2Me
23a 1H NMR
CDCl3, 400 MHz
0.92 0.94 0.91 0.92 2.02 0.93 0.98 1.97 0.92 3.00 0.85 1.97 0.94 8.03 3.04 9.06 3.02 3.04
f1 (ppm)

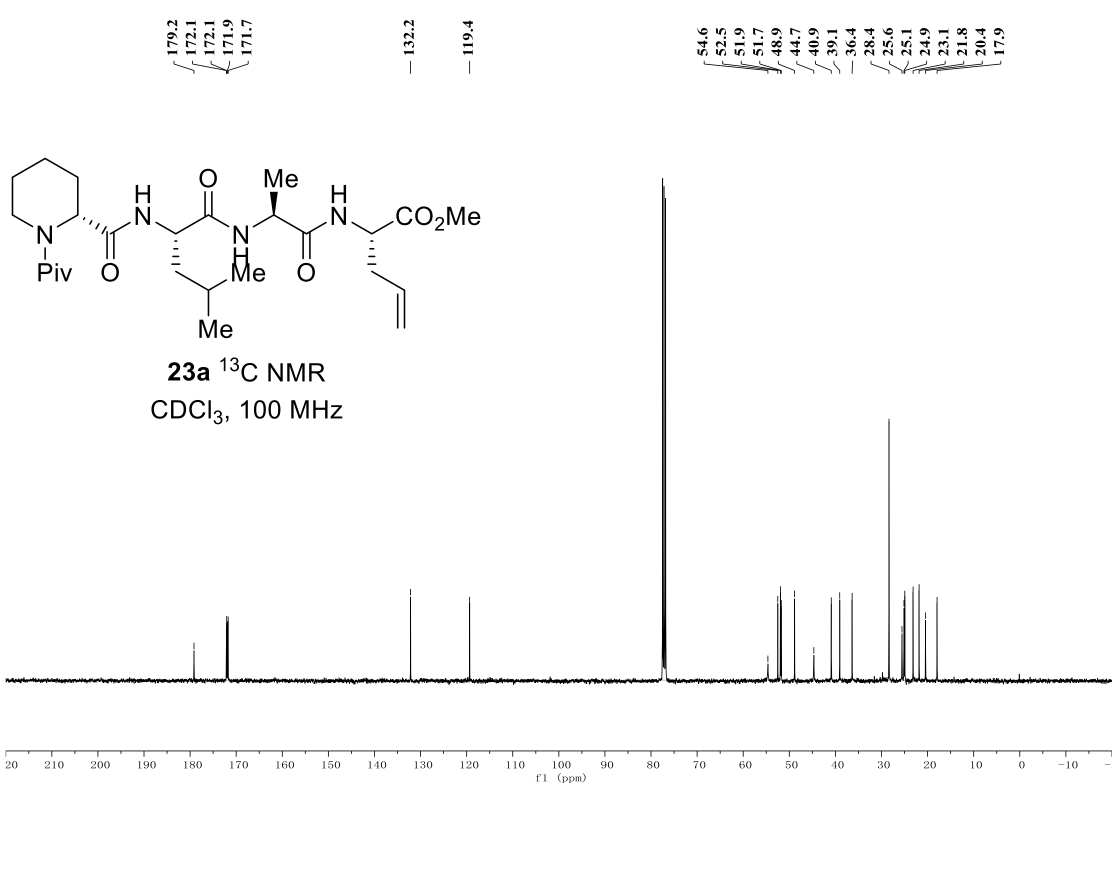

179.2
172.1
172.1
171.9
171.7
132.2
119.4
54.6
52.5
51.9
51.7
48.9
44.7
40.9
39.1
36.4
28.4
25.6
25.1
24.9
23.1
21.8
20.4
17.9
Piv
Me
CO2Me
23a 13C NMR
CDCl3, 100 MHz
f1 (ppm)

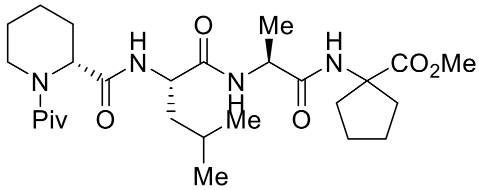

24a ^{1}H NMR
$CDCl_3$, 400 MHz

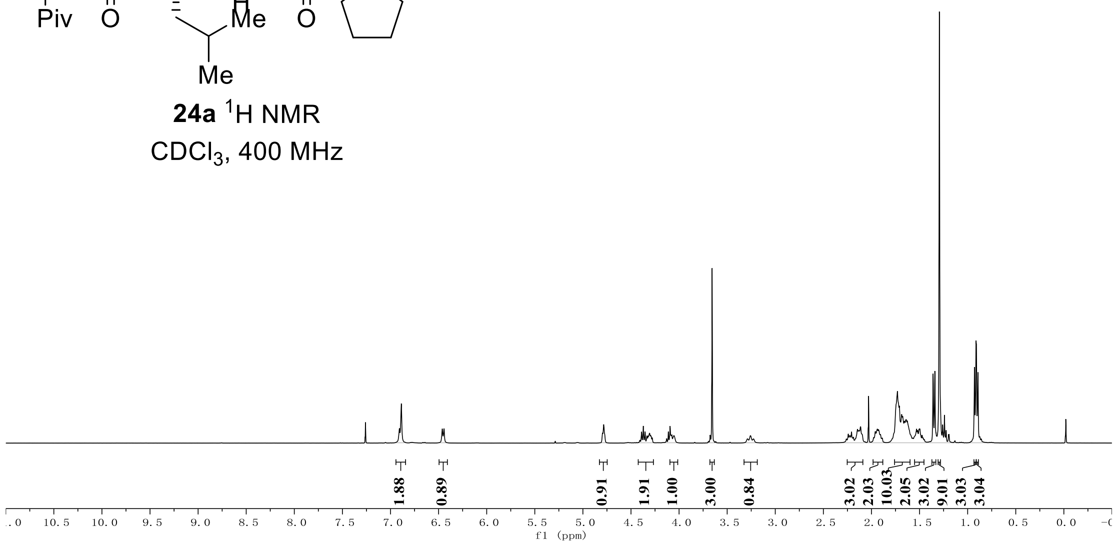

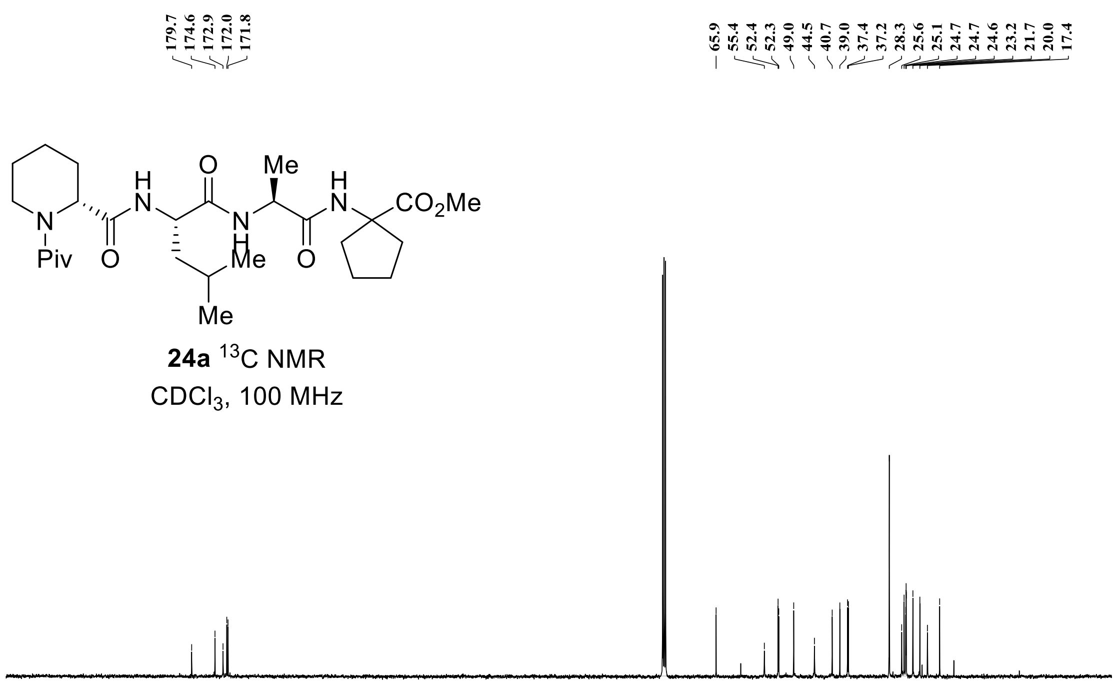

24a ^{13}C NMR
$CDCl_3$, 100 MHz

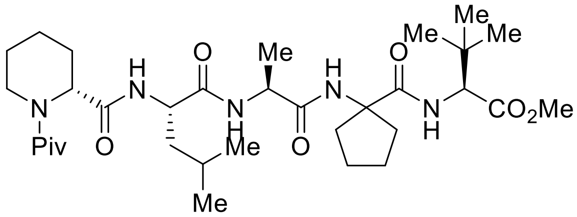

25a ^{1}H NMR
$CDCl_3$, 400 MHz

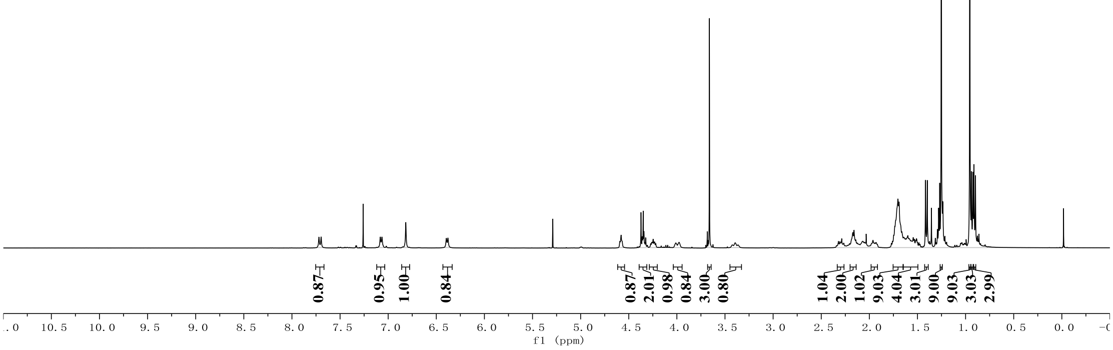

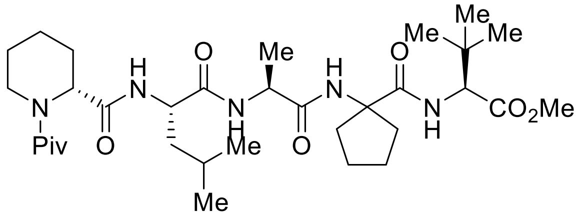

25a ^{13}C NMR
$CDCl_3$, 100 MHz

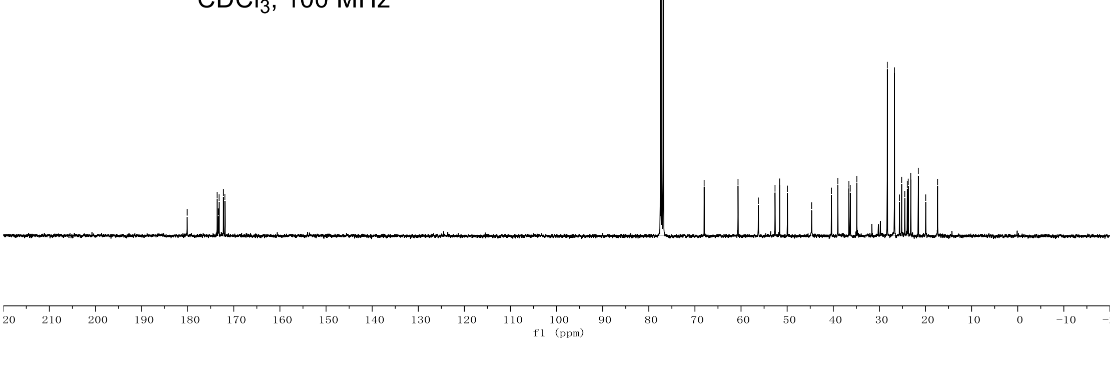

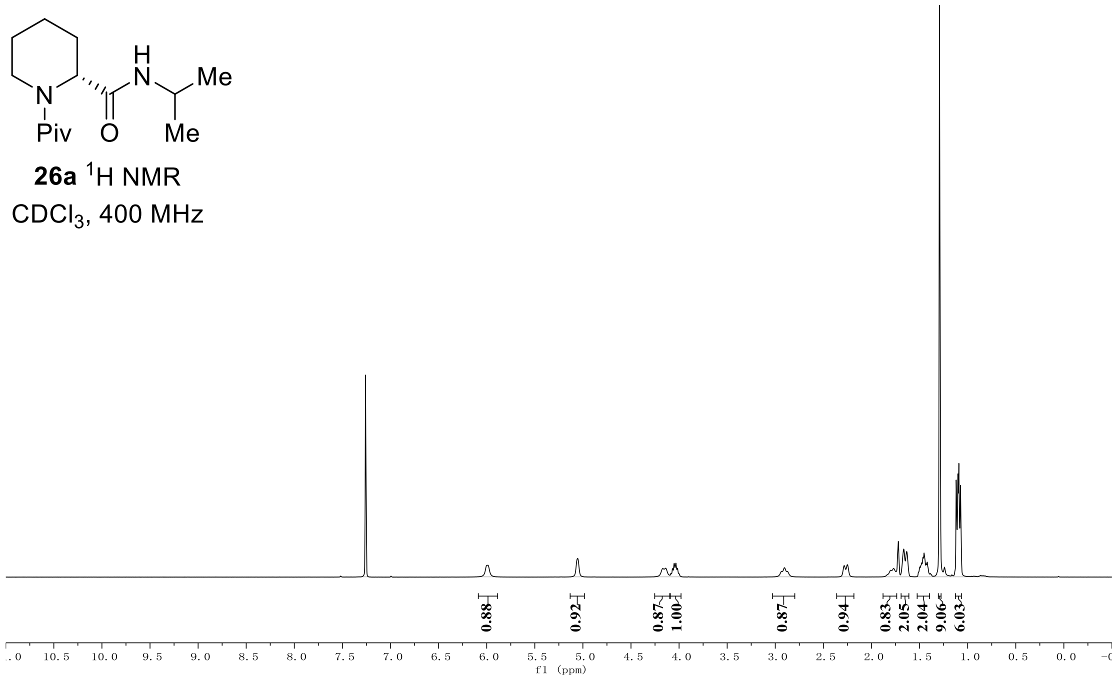
H
N
Me
N
Piv
O
Me
26a ^{1}H NMR
$CDCl_3$, 400 MHz
0.88
0.92
0.87
1.00
0.87
0.94
0.83
2.05
2.04
9.06
6.03
f1 (ppm)

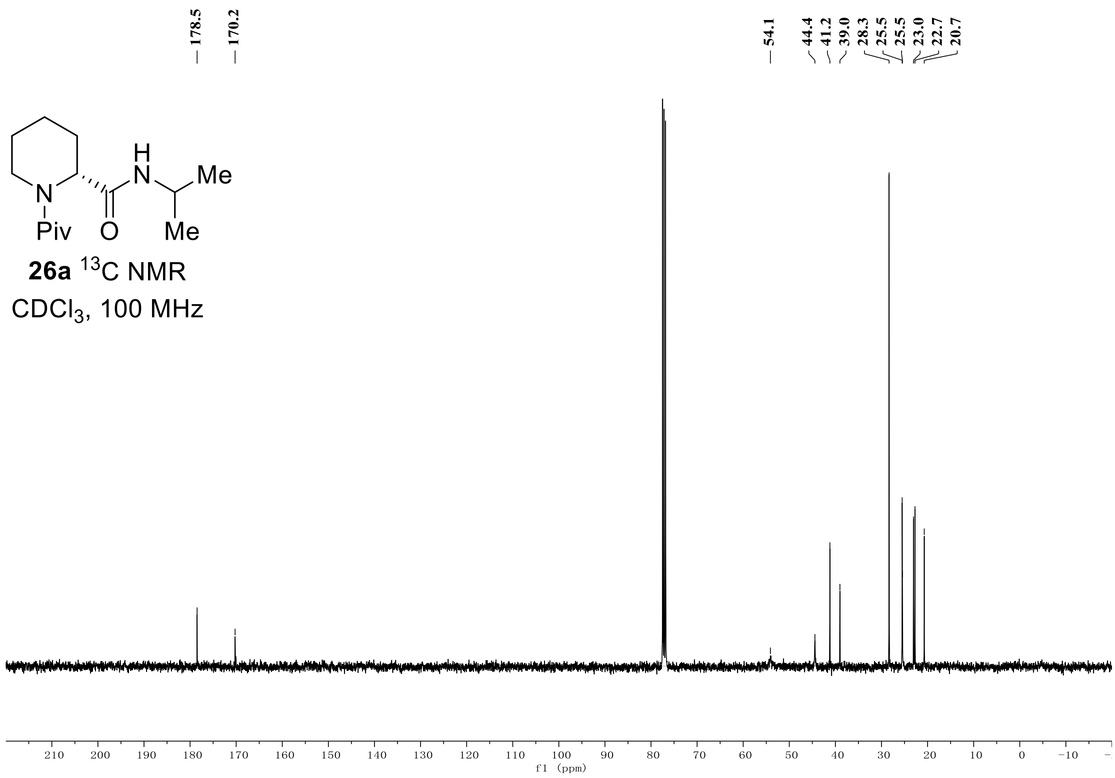
178.5
170.2
54.1
44.4
41.2
39.0
28.3
25.5
25.5
23.0
22.7
20.7
H
N
Me
N
Piv
O
Me
26a ^{13}C NMR
$CDCl_3$, 100 MHz
f1 (ppm)

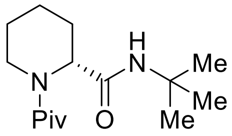

27a ^{1}H NMR
$CDCl_3$, 400 MHz

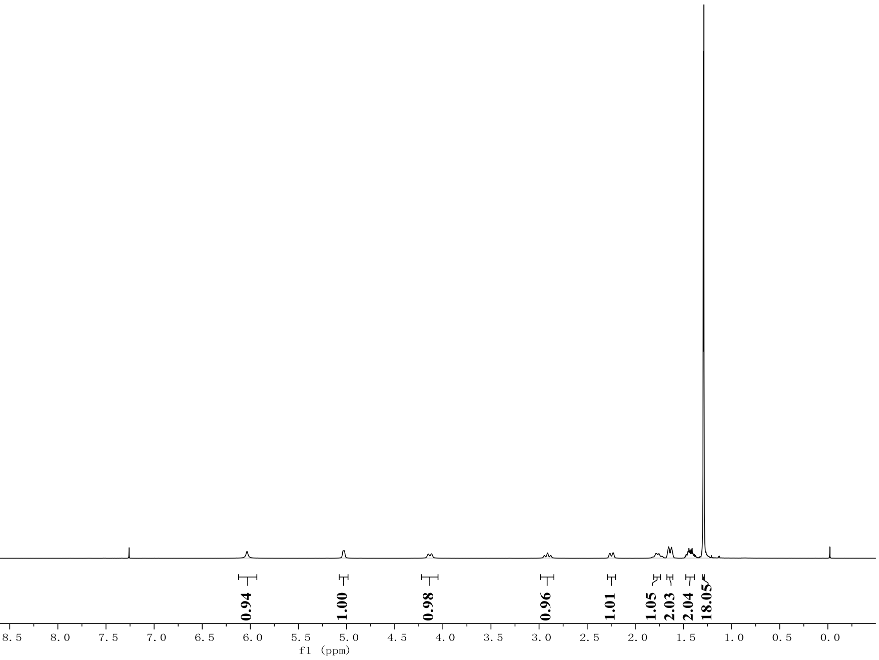

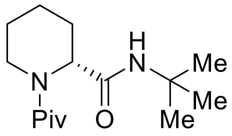

27a ^{13}C NMR
$CDCl_3$, 100 MHz

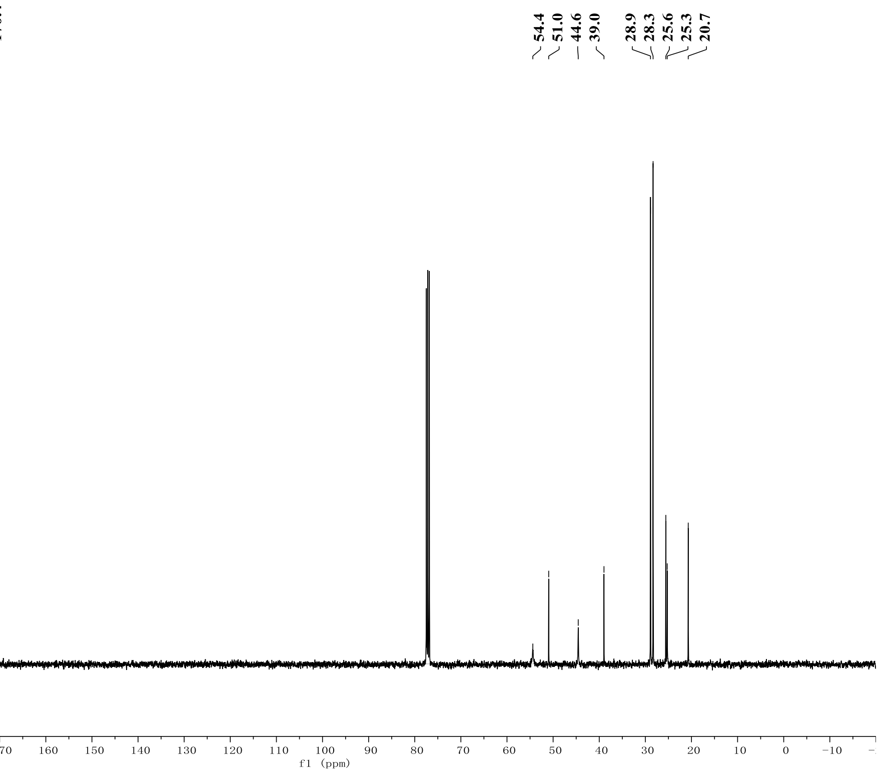

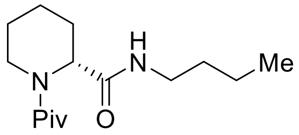

28a ^{1}H NMR

CDCl$_3$, 400 MHz

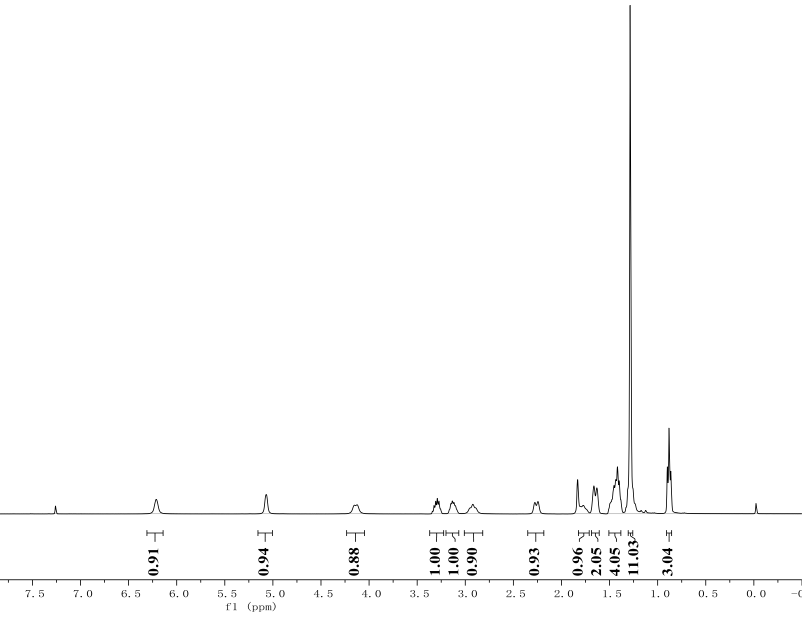

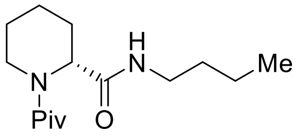

28a ^{13}C NMR

CDCl$_3$, 100 MHz

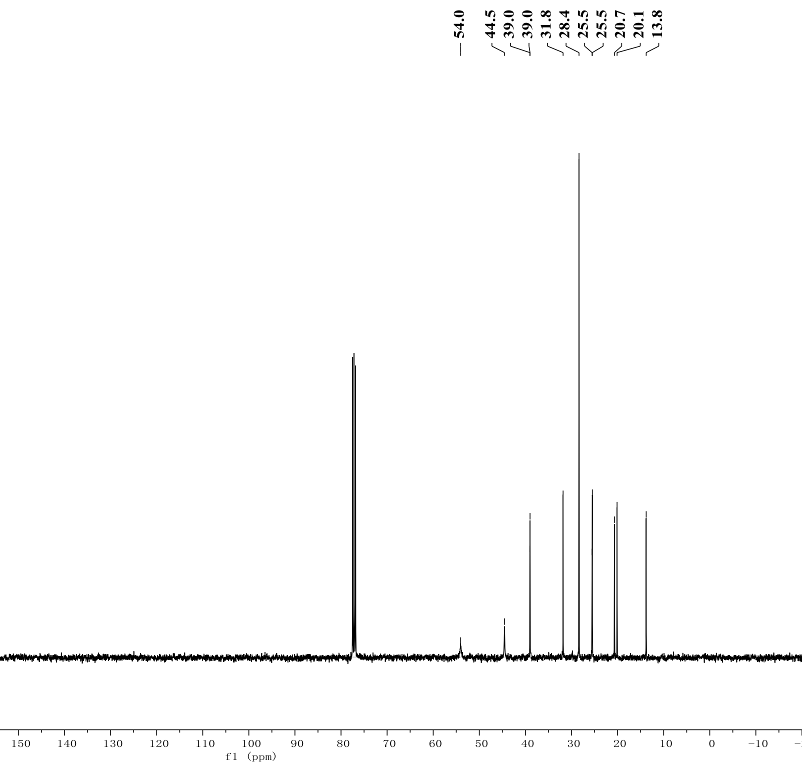

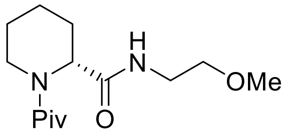

29a ^{1}H NMR
$CDCl_3$, 400 MHz

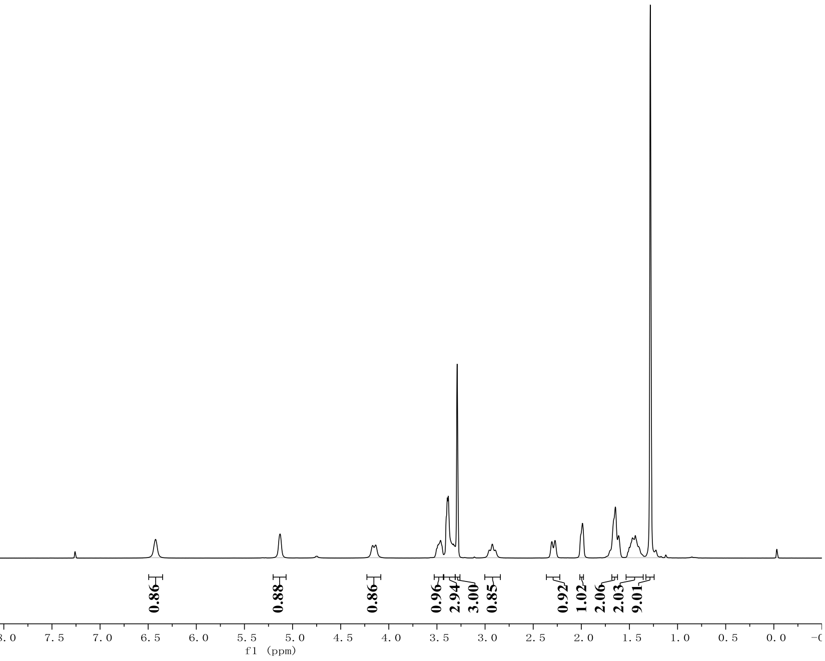

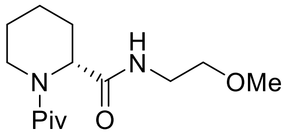

29a ^{13}C NMR
$CDCl_3$, 100 MHz

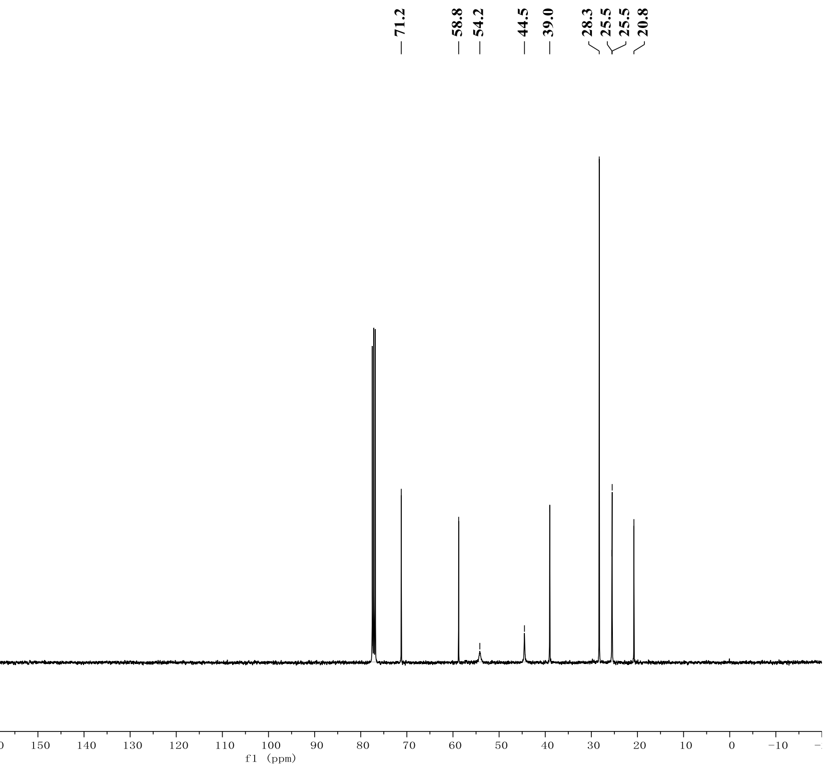

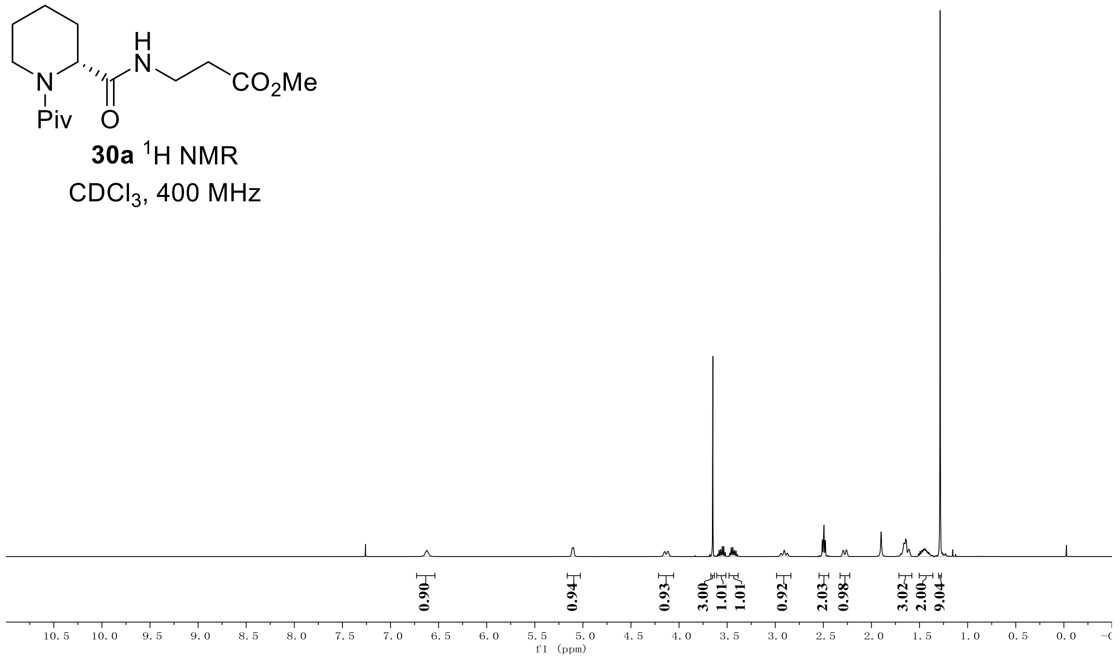

30a ^{1}H NMR
CDCl$_3$, 400 MHz
0.90
0.94
0.93
3.00
1.01
1.01
0.92
2.03
0.98
3.02
2.00
9.04
f1 (ppm)

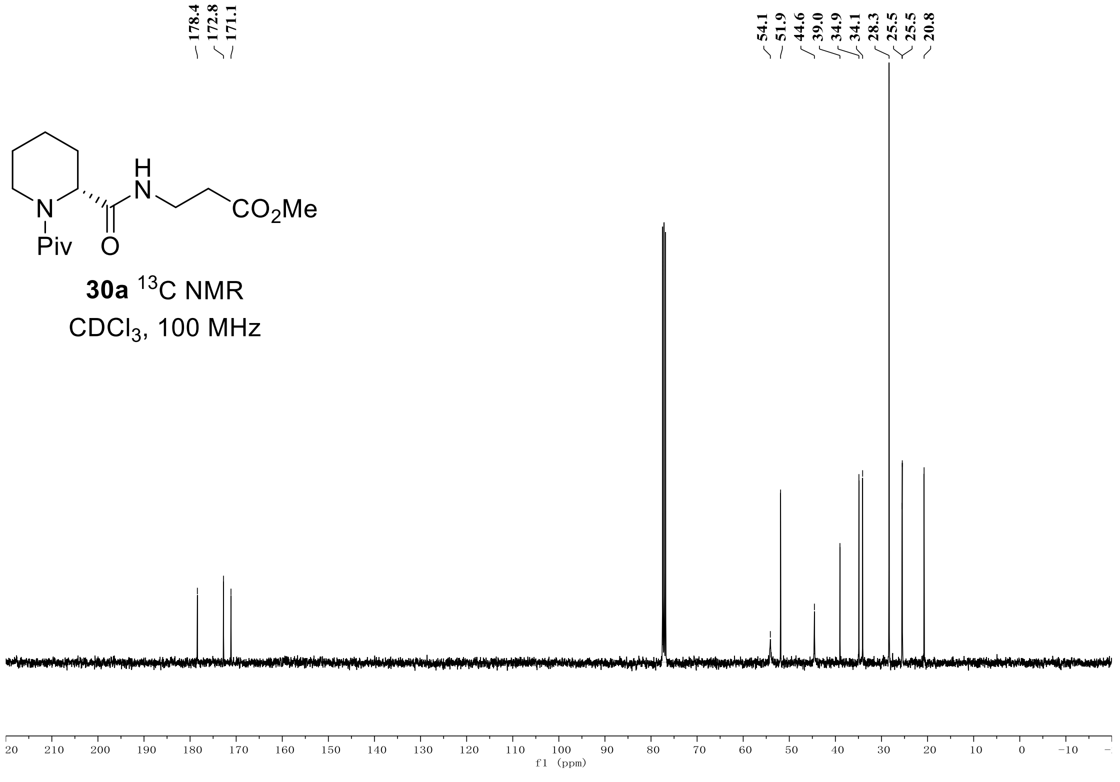

178.4
172.8
171.1
54.1
51.9
44.6
39.0
34.9
34.1
28.3
25.5
25.5
20.8
30a ^{13}C NMR
CDCl$_3$, 100 MHz
f1 (ppm)

31a ^{1}H NMR
$CDCl_3$, 400 MHz

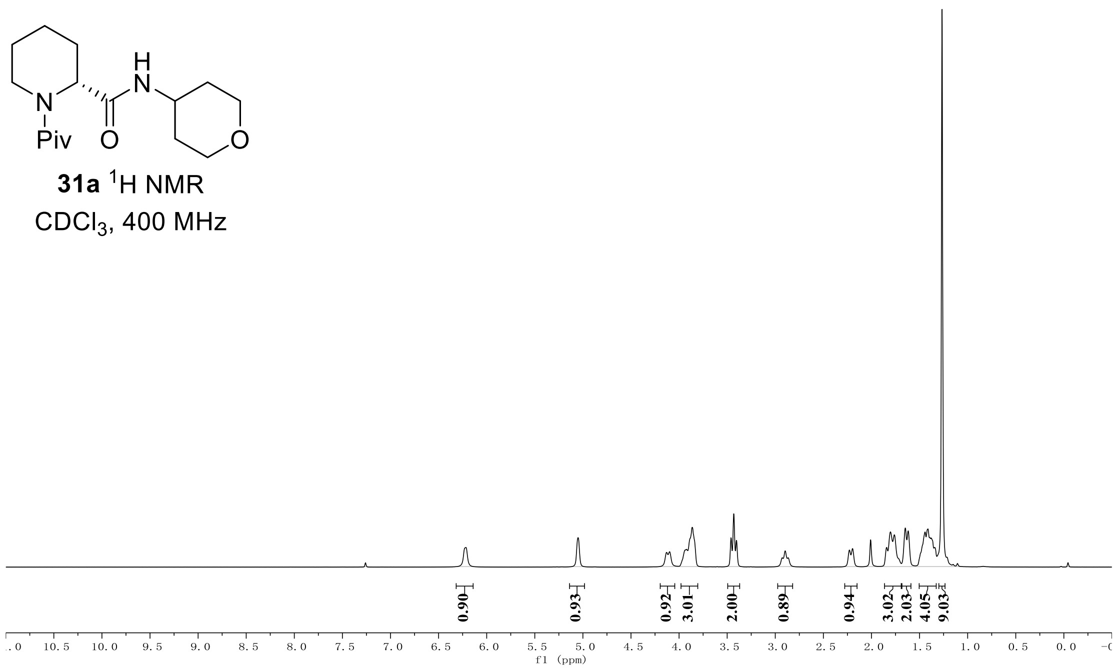

31a ^{13}C NMR
$CDCl_3$, 100 MHz

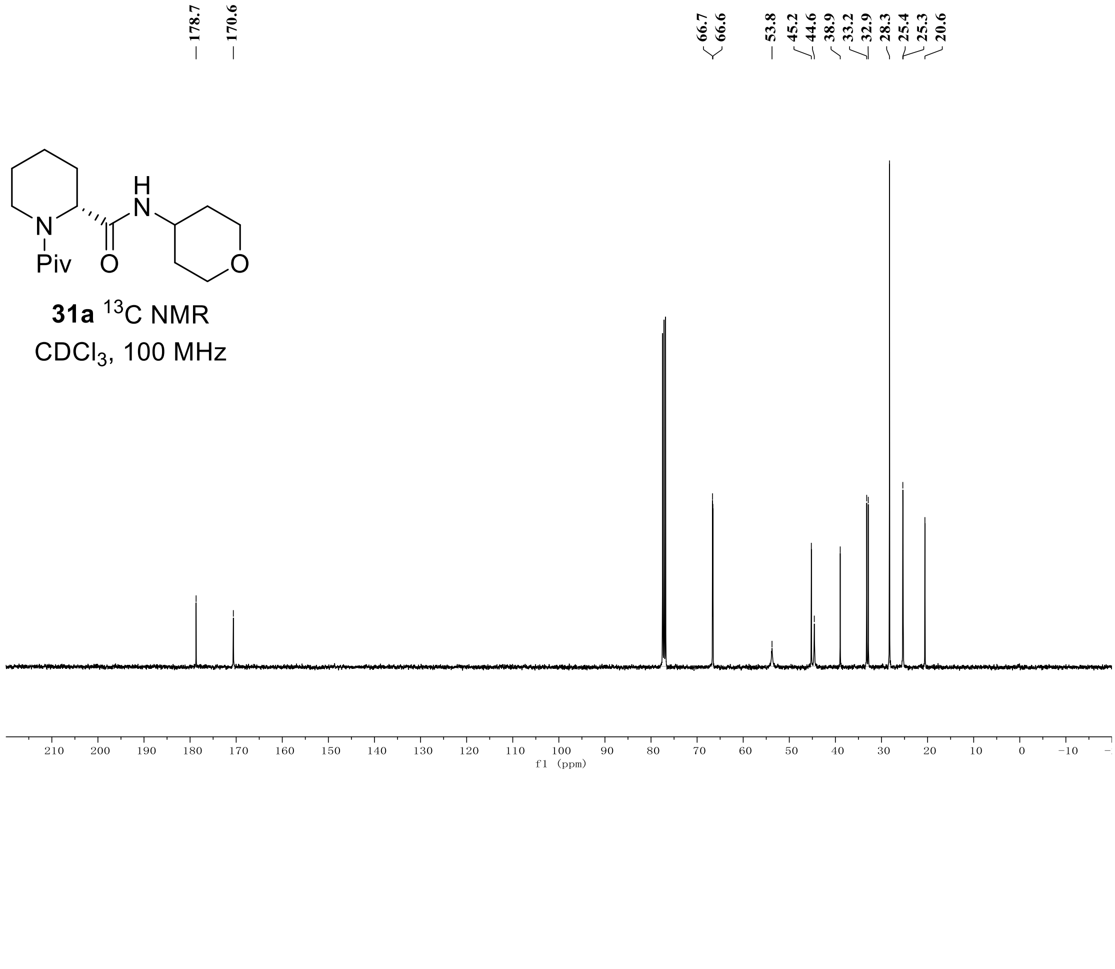

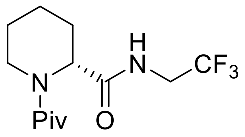

32a ^{1}H NMR
$CDCl_3$, 400 MHz

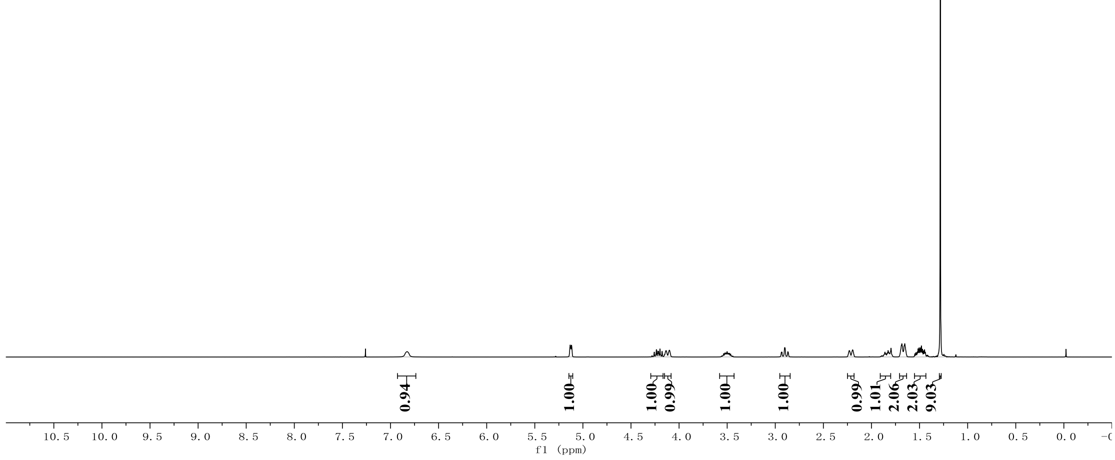

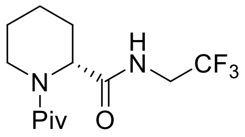

32a ^{13}C NMR
$CDCl_3$, 100 MHz

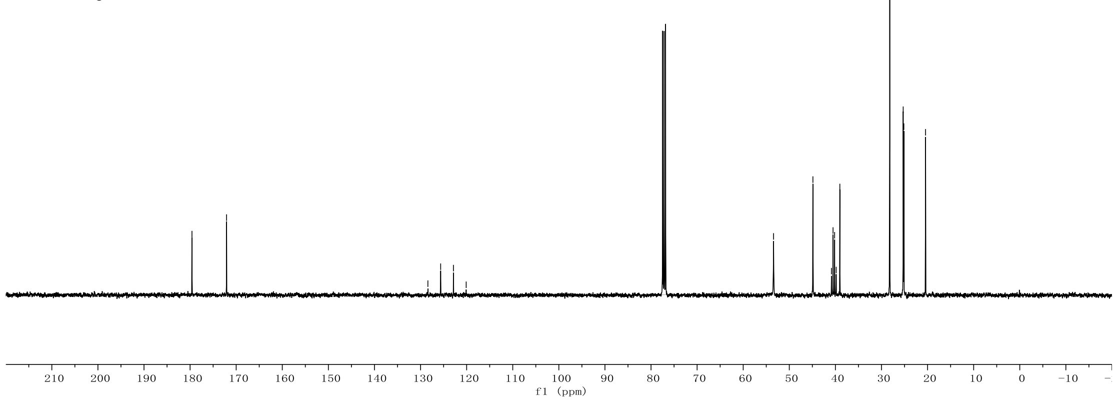

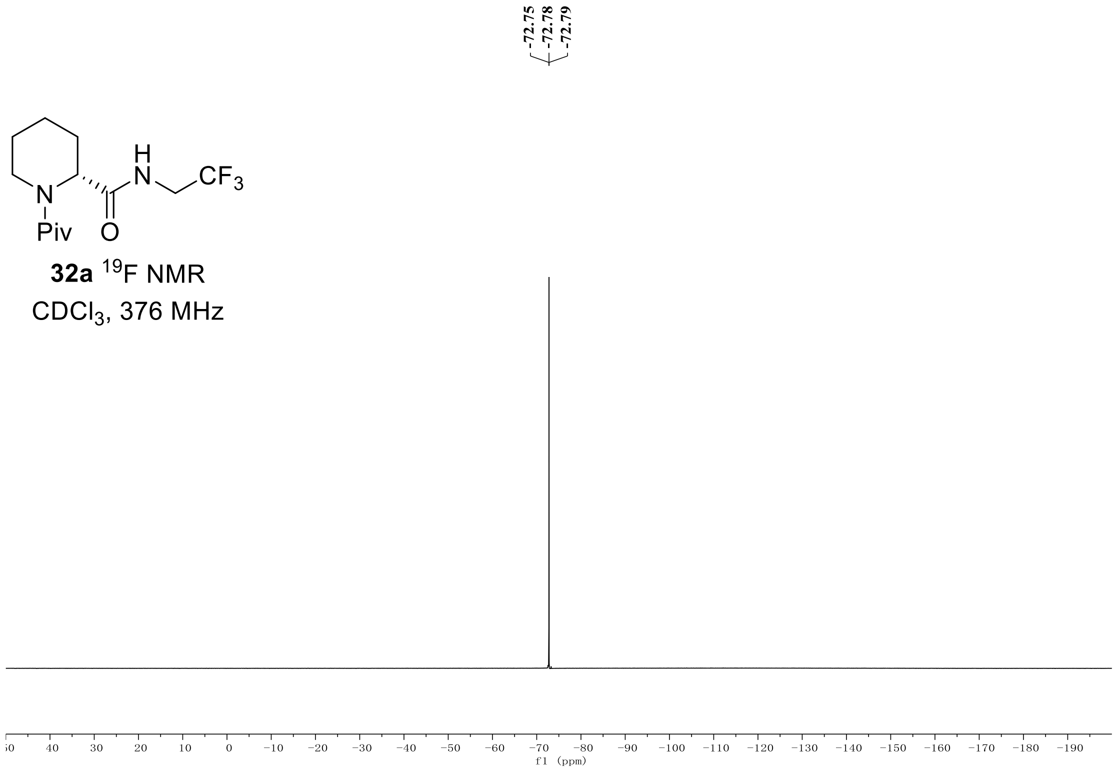
-72.75
-72.78
-72.79
32a ^{19}F NMR
$CDCl_3$, 376 MHz
f1 (ppm)

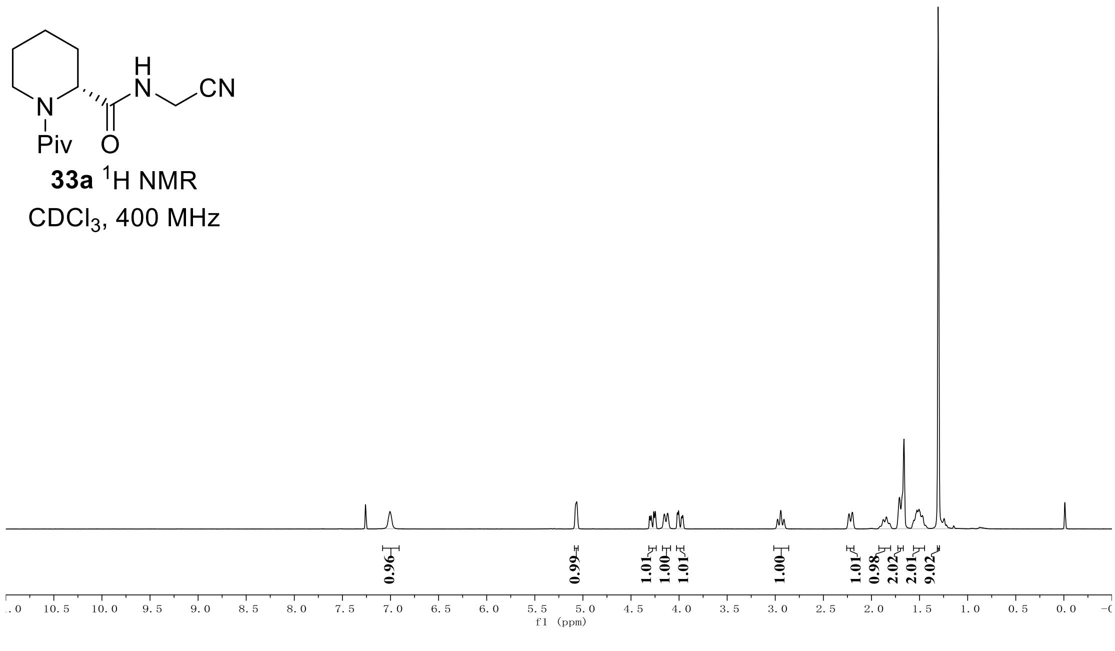
33a ^{1}H NMR
$CDCl_3$, 400 MHz
0.96
0.99
1.01
1.00
1.01
1.00
1.01
0.98
2.02
2.01
9.02
f1 (ppm)

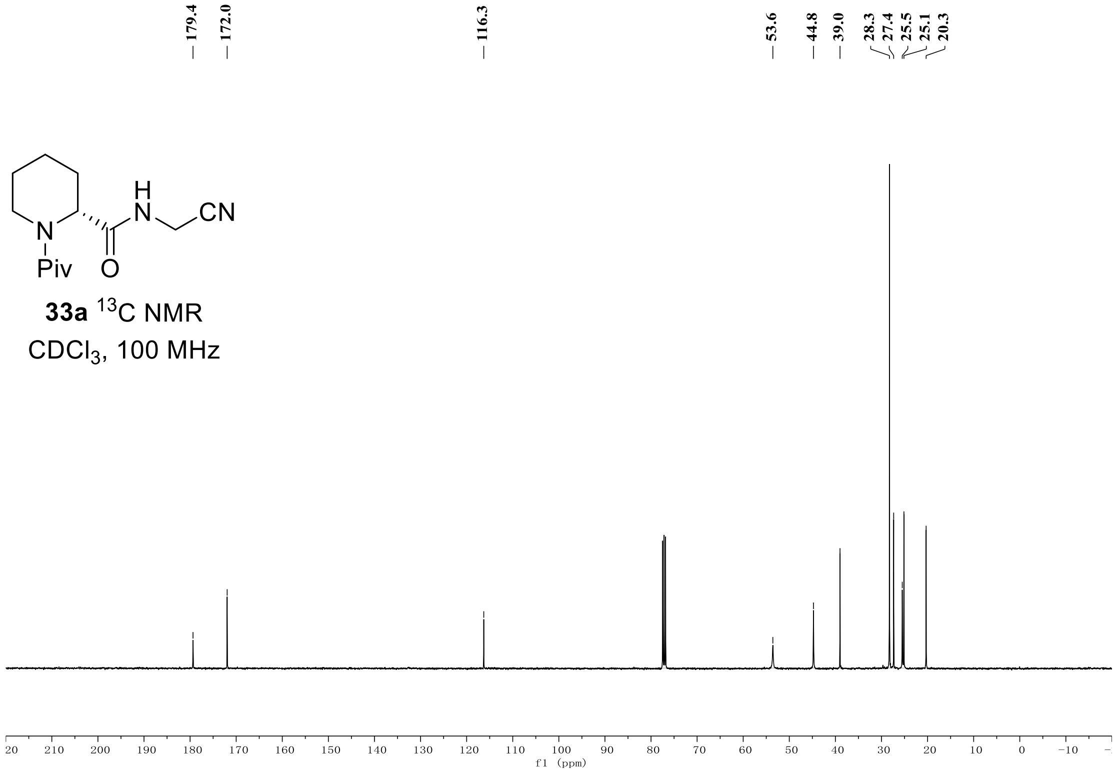
179.4
172.0
116.3
53.6
44.8
39.0
28.3
27.4
25.5
25.1
20.3
H
N
CN
N
Piv
O
33a ^{13}C NMR
$CDCl_3$, 100 MHz
f1 (ppm)

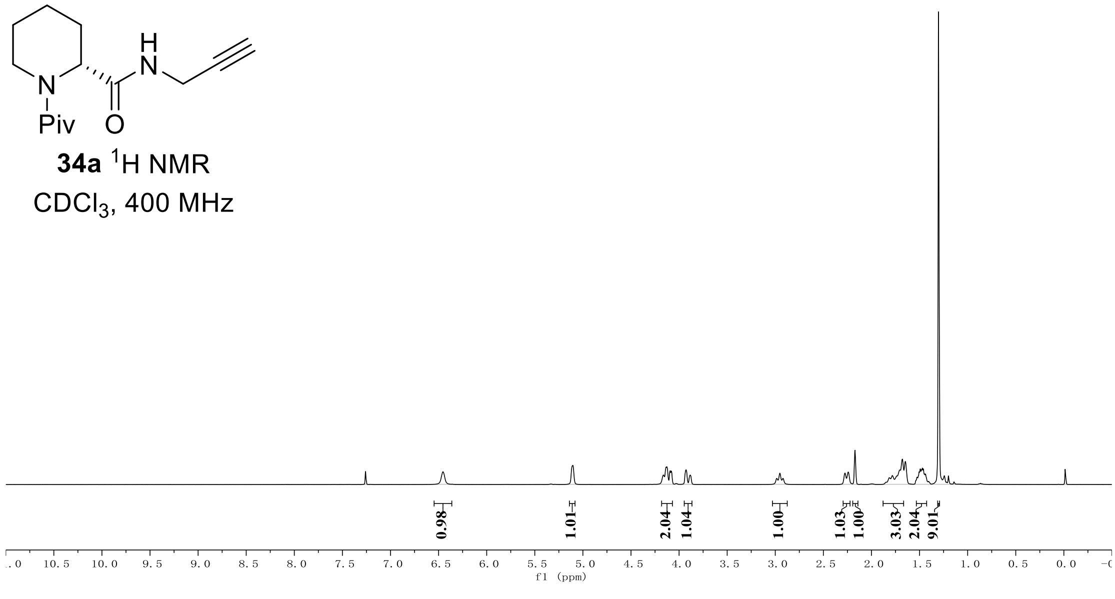
H
N
N
Piv
O
34a ^{1}H NMR
$CDCl_3$, 400 MHz
0.98
1.01
2.04
1.04
1.00
1.03
1.00
3.03
2.04
9.01
f1 (ppm)

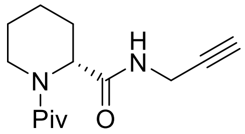

34a ^{13}C NMR
$CDCl_3$, 100 MHz

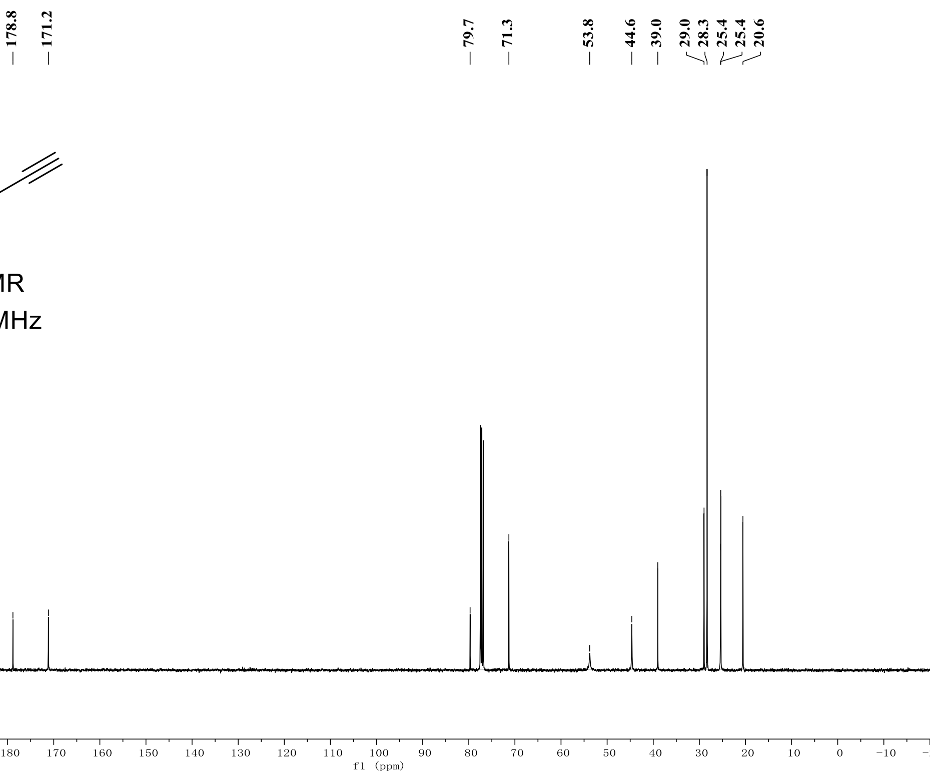

35a ^{1}H NMR
$CDCl_3$, 400 MHz

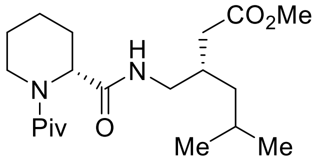

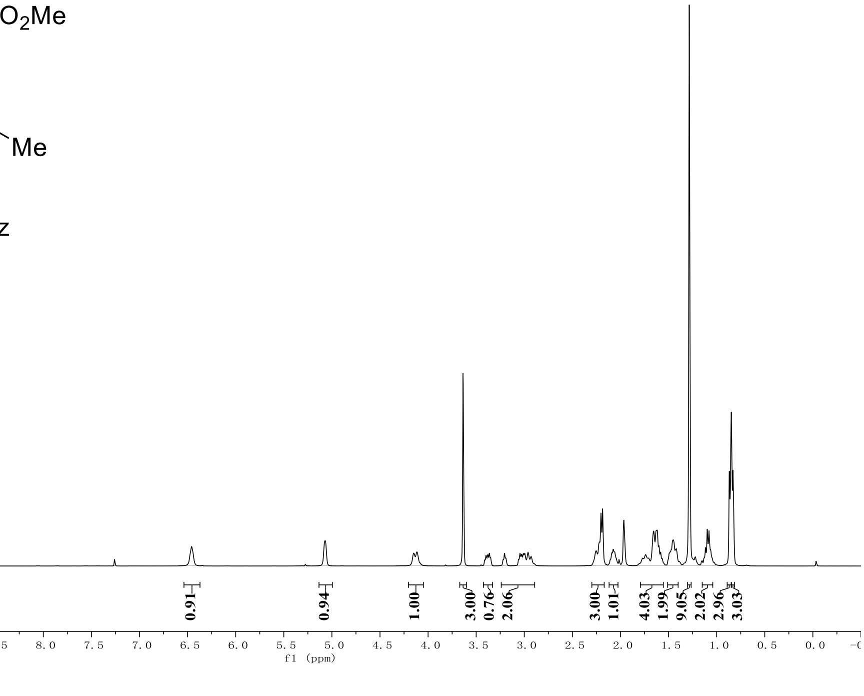

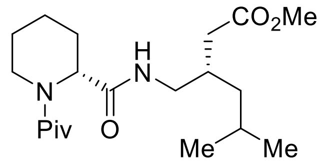

35a ^{13}C NMR

$CDCl_3$, 100 MHz

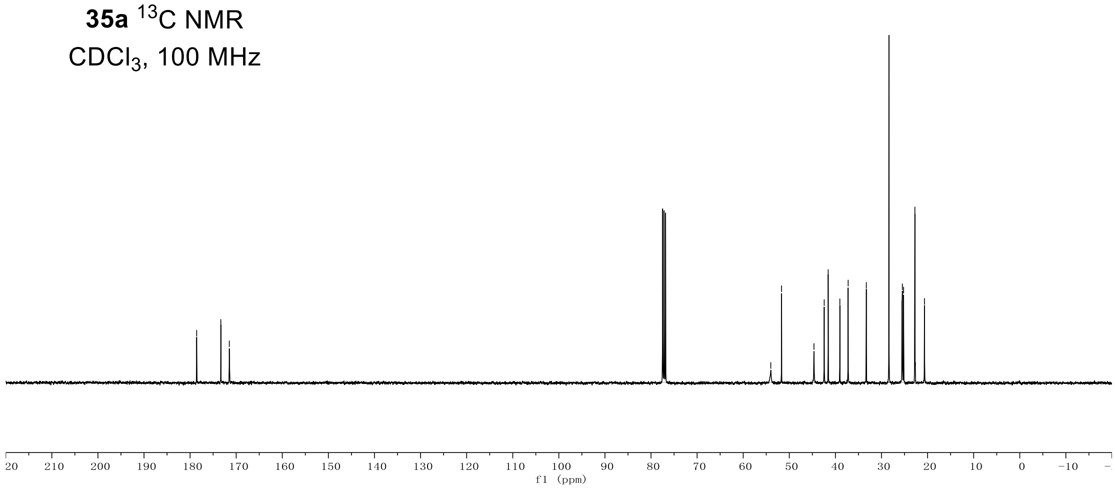

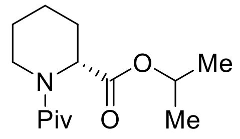

36a ^{1}H NMR

$CDCl_3$, 400 MHz

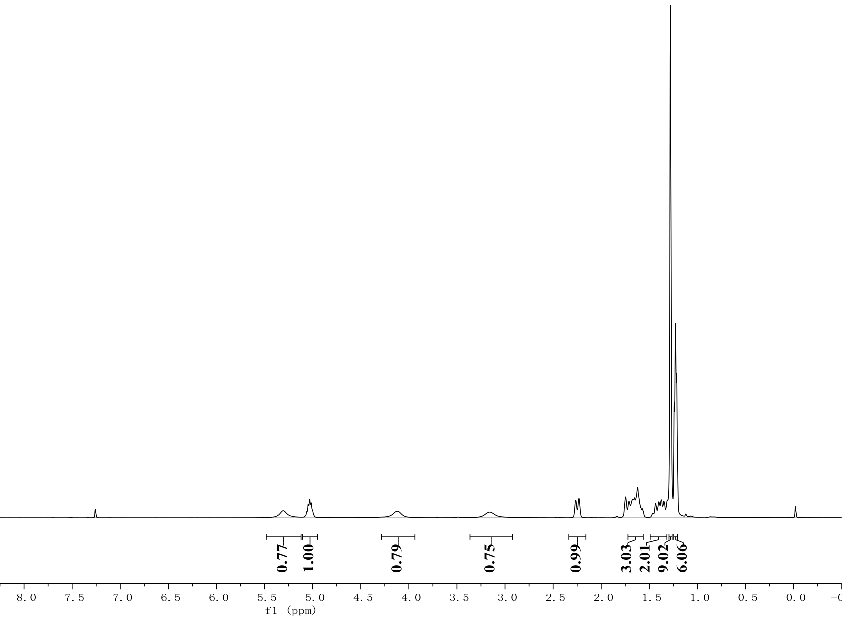

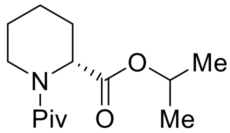

36a ^{13}C NMR
$CDCl_3$, 100 MHz

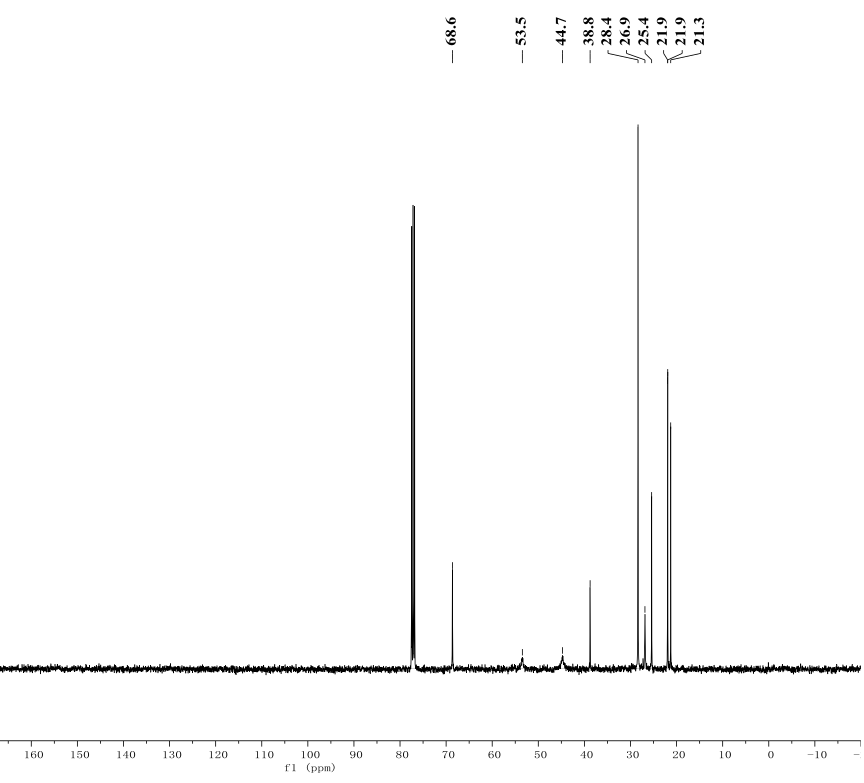

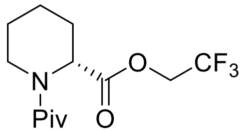

37a ^{1}H NMR
$CDCl_3$, 400 MHz

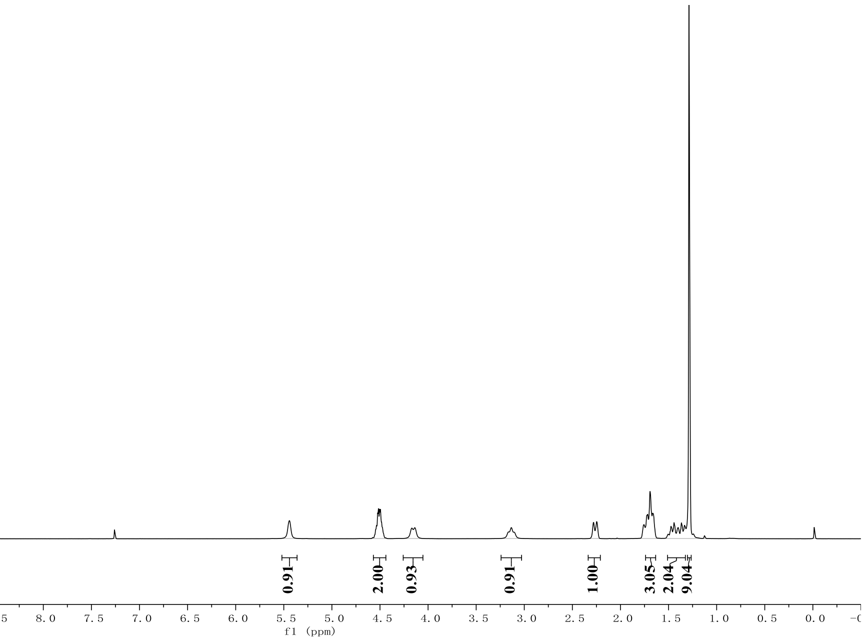

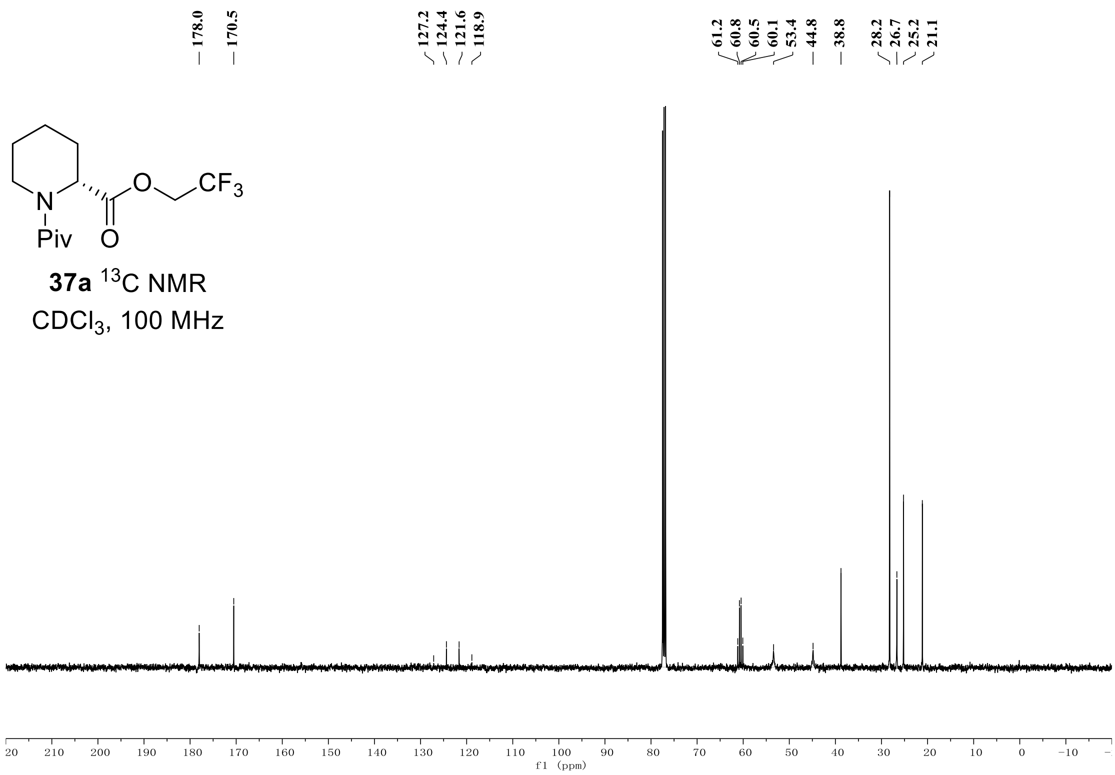
178.0
170.5
127.2
124.4
121.6
118.9
61.2
60.8
60.5
60.1
53.4
44.8
38.8
28.2
26.7
25.2
21.1
N
Piv
O
O
CF3
37a 13C NMR
CDCl3, 100 MHz
f1 (ppm)

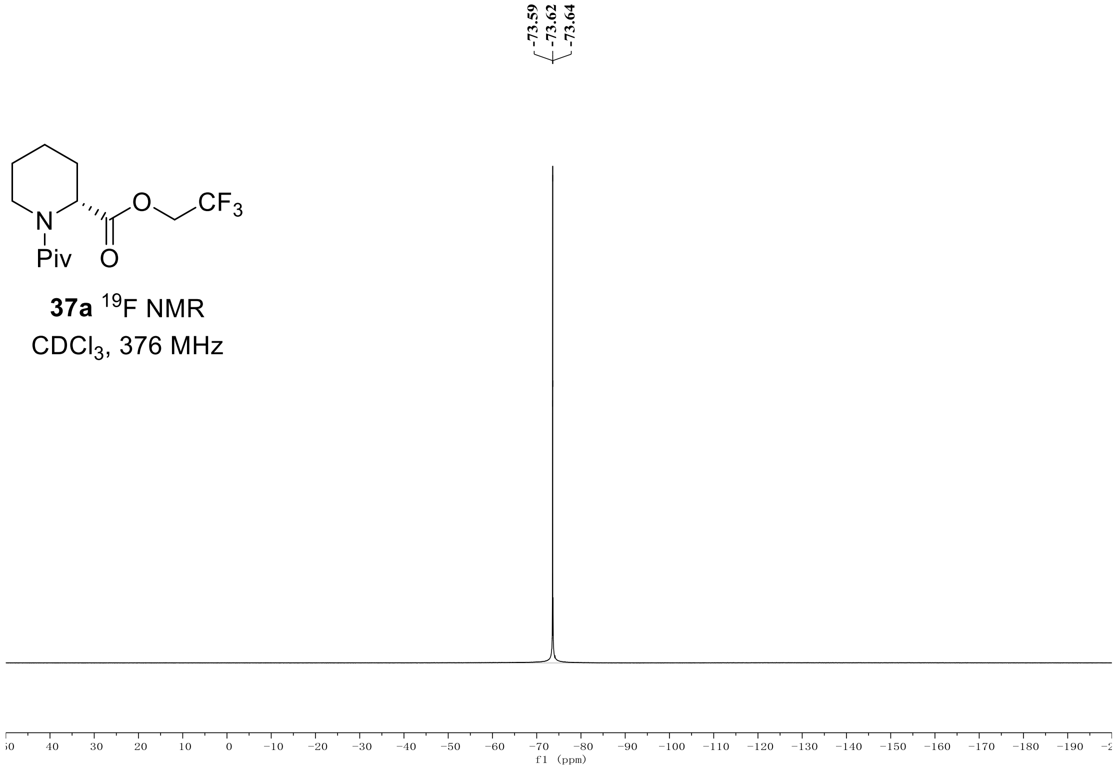
-73.59
-73.62
-73.64
N
Piv
O
O
CF3
37a 19F NMR
CDCl3, 376 MHz
f1 (ppm)

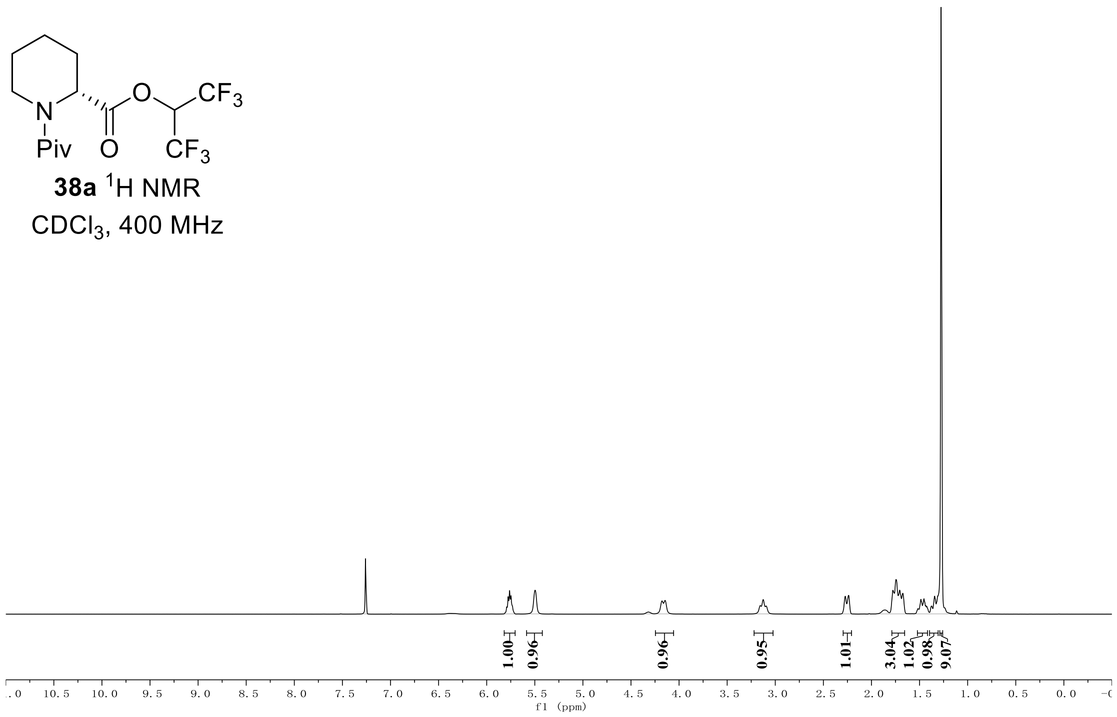

38a 1H NMR
CDCl3, 400 MHz
1.00
0.96
0.96
0.95
1.01
3.04
1.02
0.98
9.07
f1 (ppm)

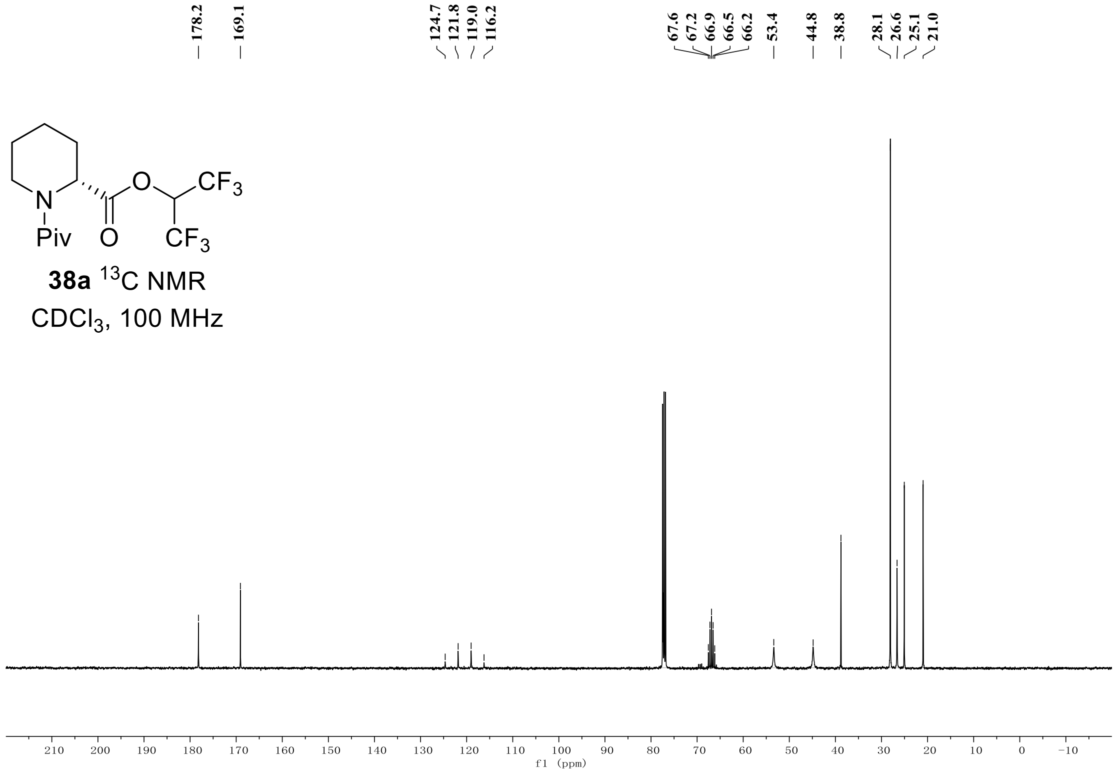

178.2
169.1
124.7
121.8
119.0
116.2
67.6
67.2
66.9
66.5
66.2
53.4
44.8
38.8
28.1
26.6
25.1
21.0
38a 13C NMR
CDCl3, 100 MHz
f1 (ppm)

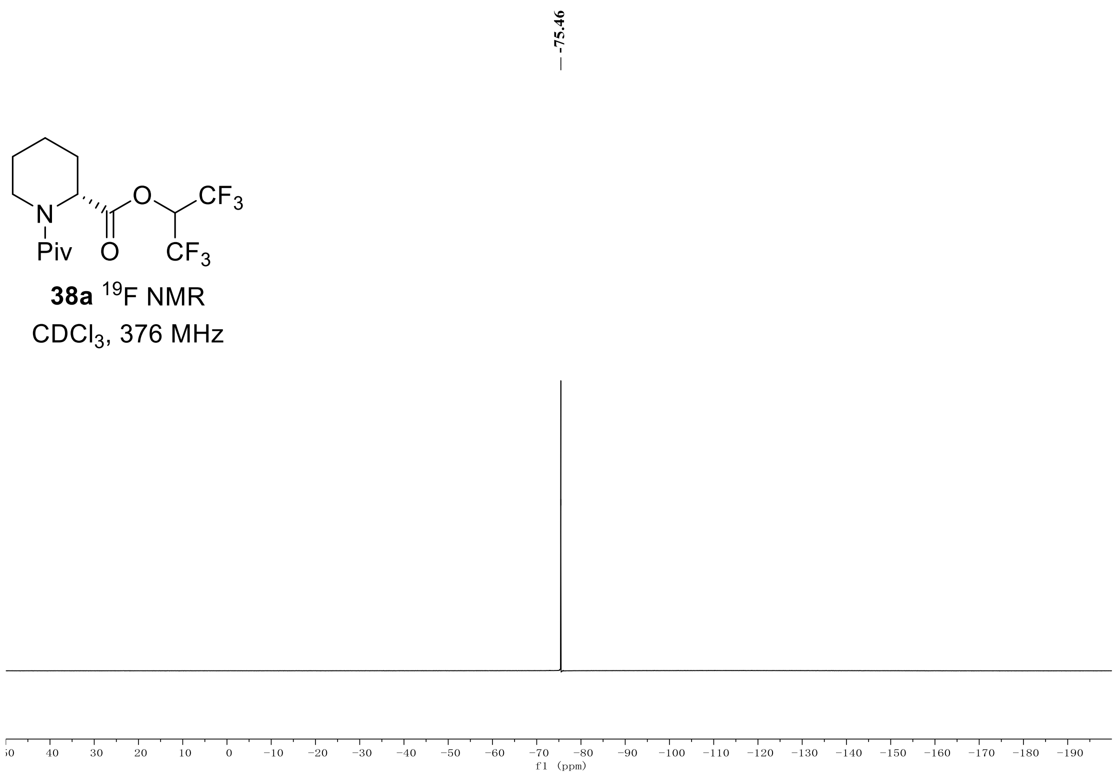
-75.46
N
Piv
O
O
CF_3
CF_3
38a ^{19}F NMR
$CDCl_3$, 376 MHz
f1 (ppm)

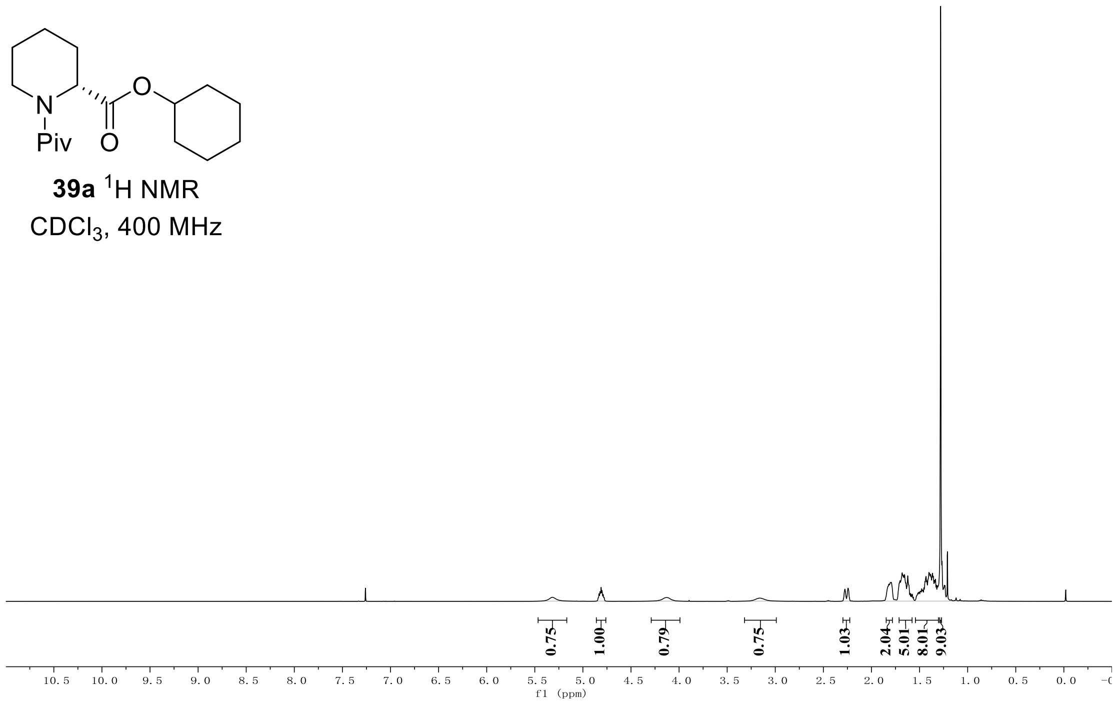
N
Piv
O
O
39a ^{1}H NMR
$CDCl_3$, 400 MHz
0.75
1.00
0.79
0.75
1.03
2.04
5.01
8.01
9.03
f1 (ppm)

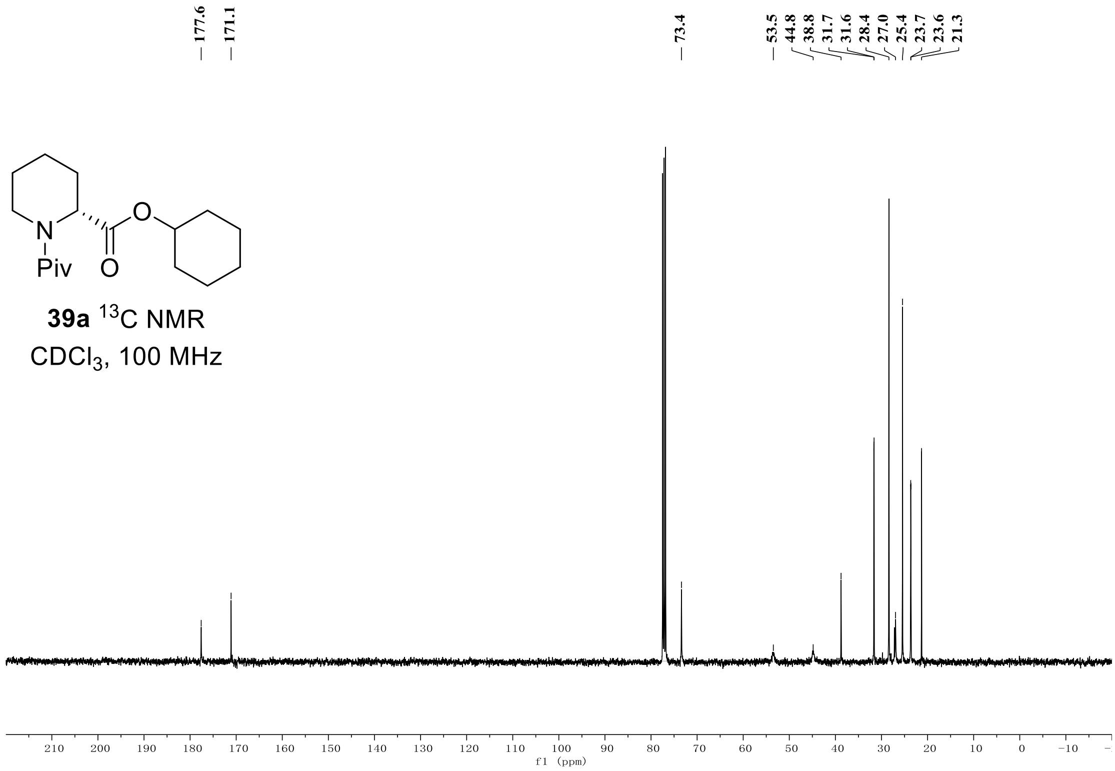
177.6
171.1
73.4
53.5
44.8
38.8
31.7
31.6
28.4
27.0
25.4
23.7
23.6
21.3
Piv
39a ^{13}C NMR
$CDCl_3$, 100 MHz
f1 (ppm)

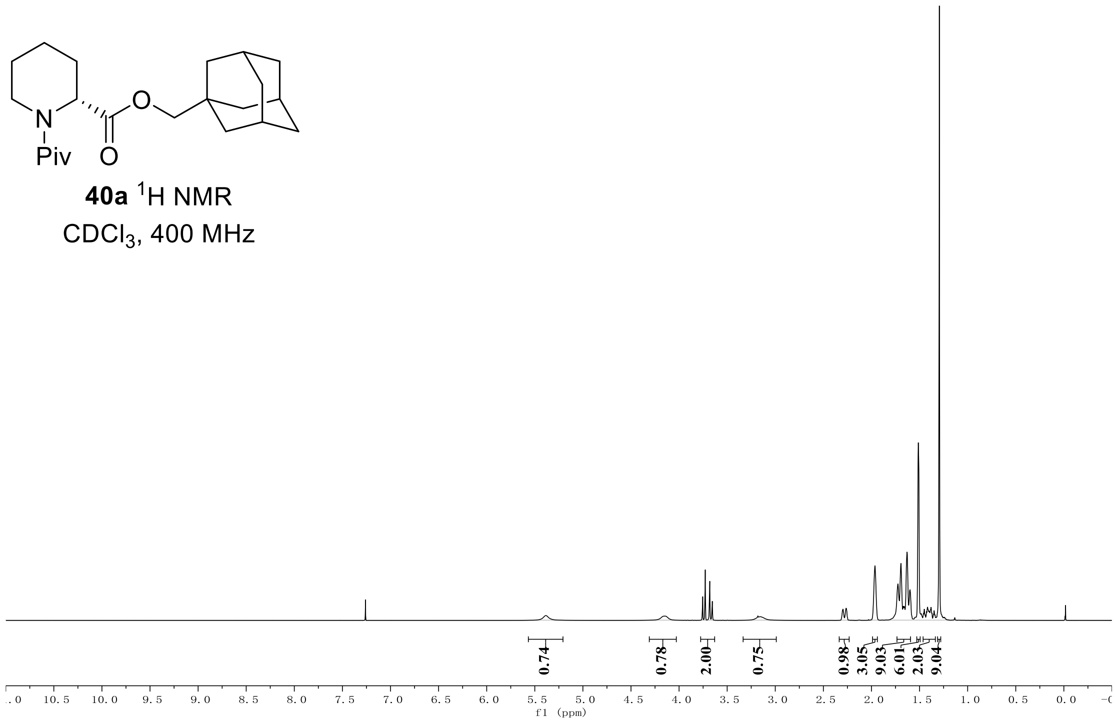
Piv
40a ^{1}H NMR
$CDCl_3$, 400 MHz
0.74
0.78
2.00
0.75
0.98
3.05
9.03
6.01
2.03
9.04
f1 (ppm)

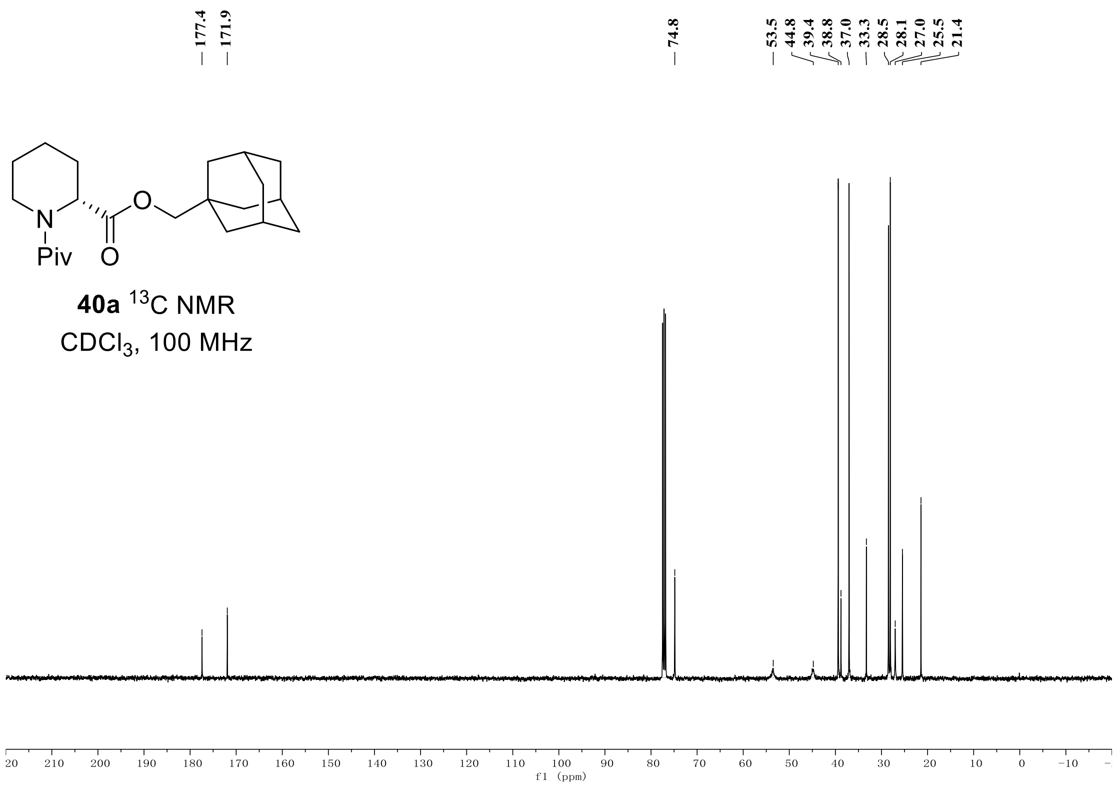

40a ^{13}C NMR
$CDCl_3$, 100 MHz

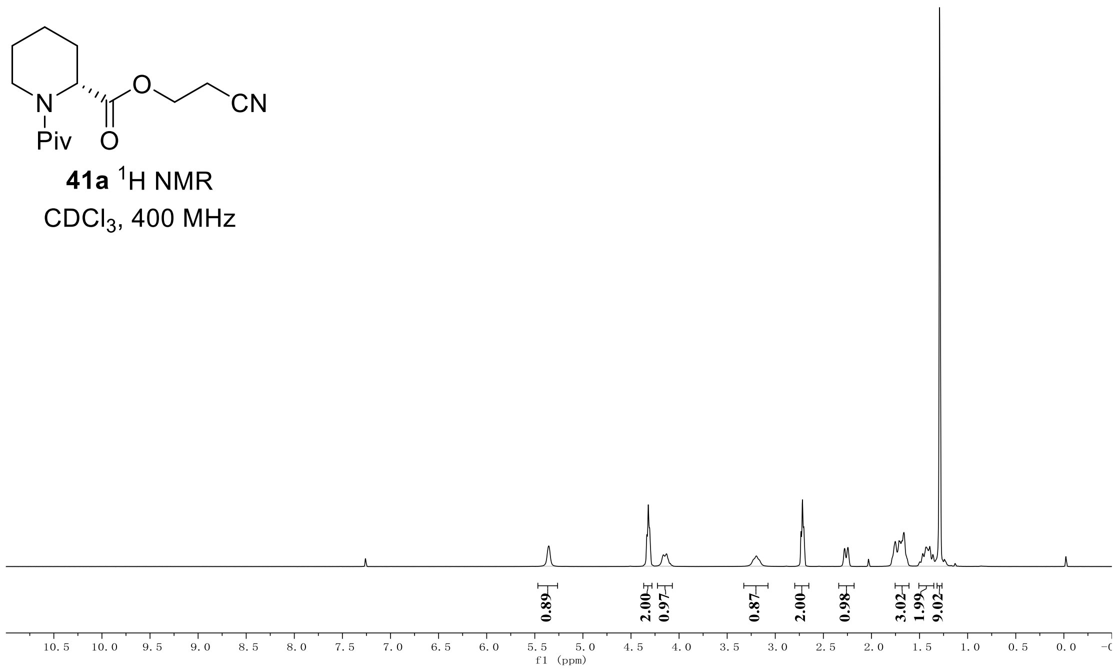

41a ^{1}H NMR
$CDCl_3$, 400 MHz

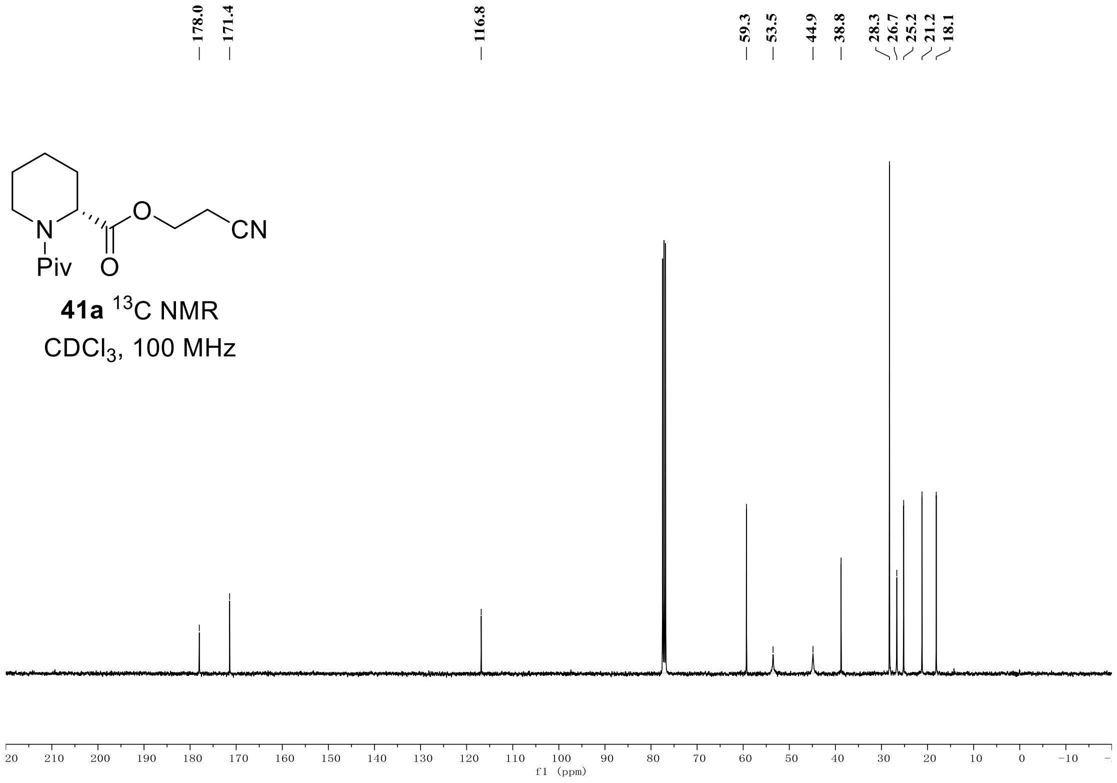
178.0
171.4
116.8
59.3
53.5
44.9
38.8
28.3
26.7
25.2
21.2
18.1
CN
Piv
41a ^{13}C NMR
$CDCl_3$, 100 MHz
f1 (ppm)

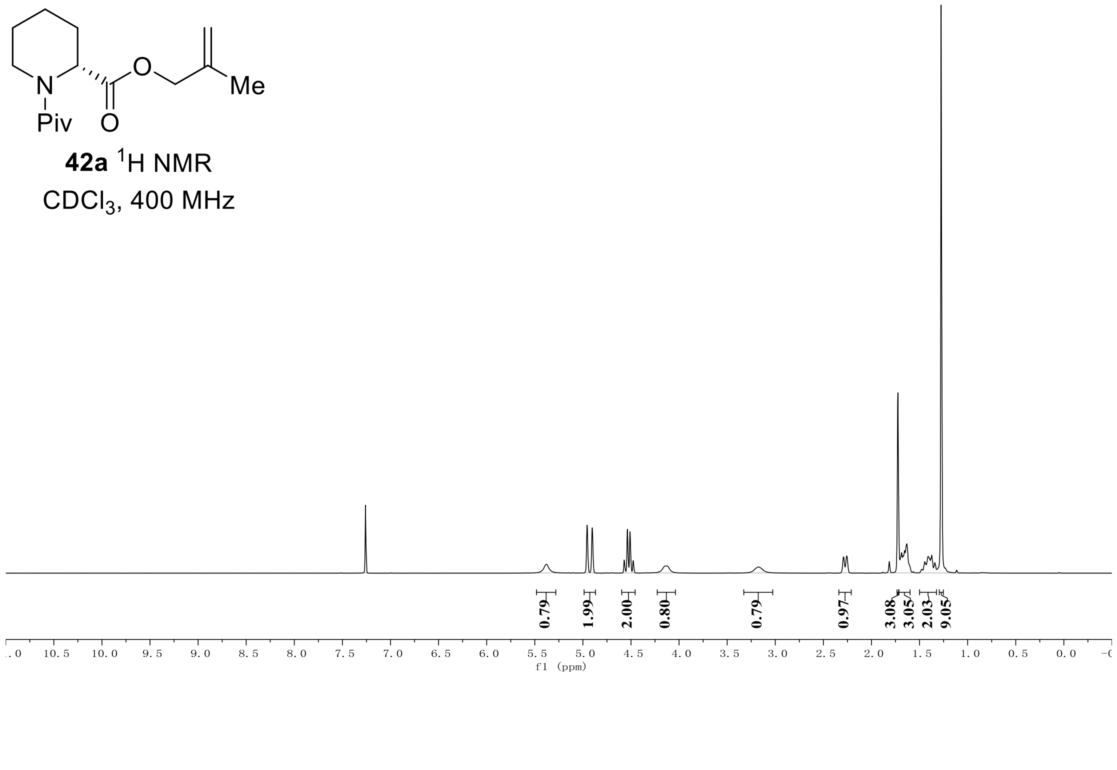
Me
Piv
42a ^{1}H NMR
$CDCl_3$, 400 MHz
0.79
1.99
2.00
0.80
0.79
0.97
3.08
3.05
2.03
9.05
f1 (ppm)

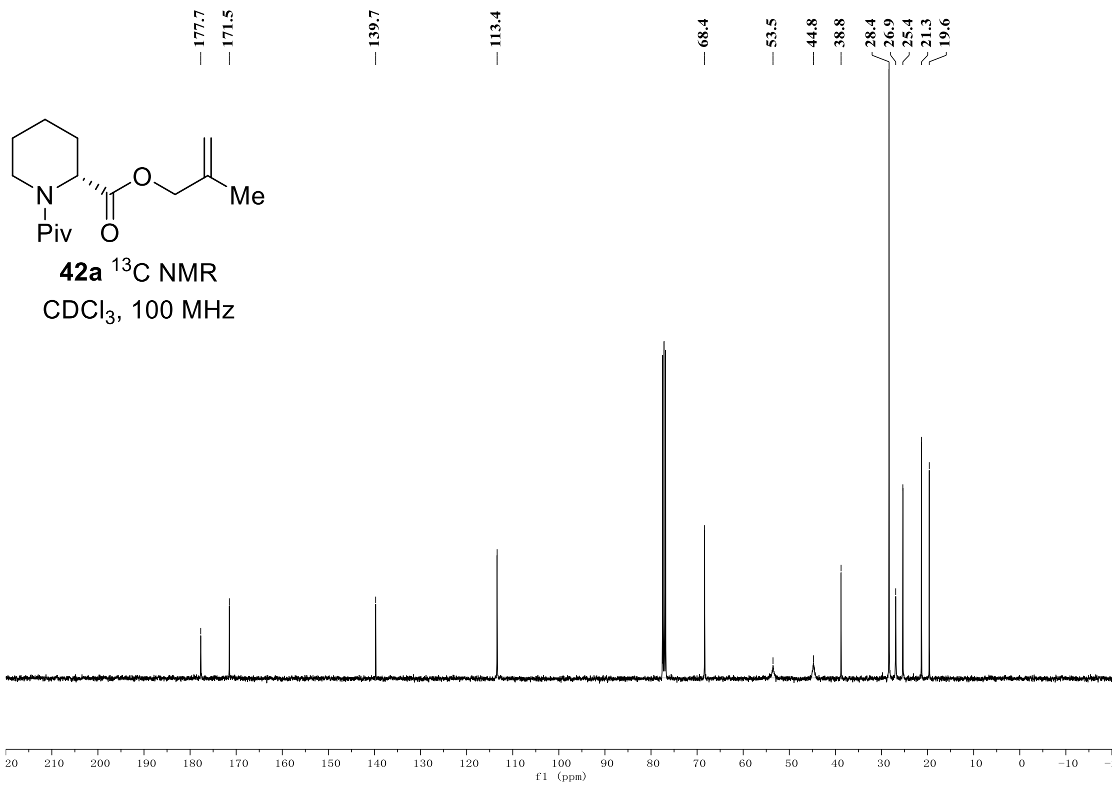

42a ^{13}C NMR
$CDCl_3$, 100 MHz

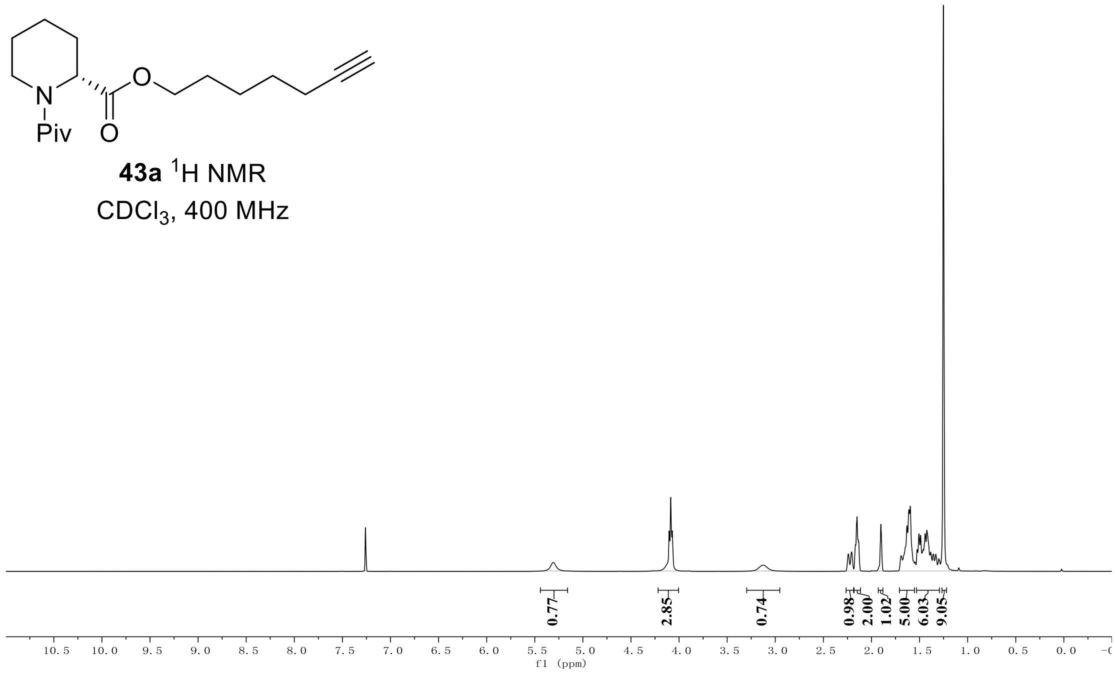

43a ^{1}H NMR
$CDCl_3$, 400 MHz

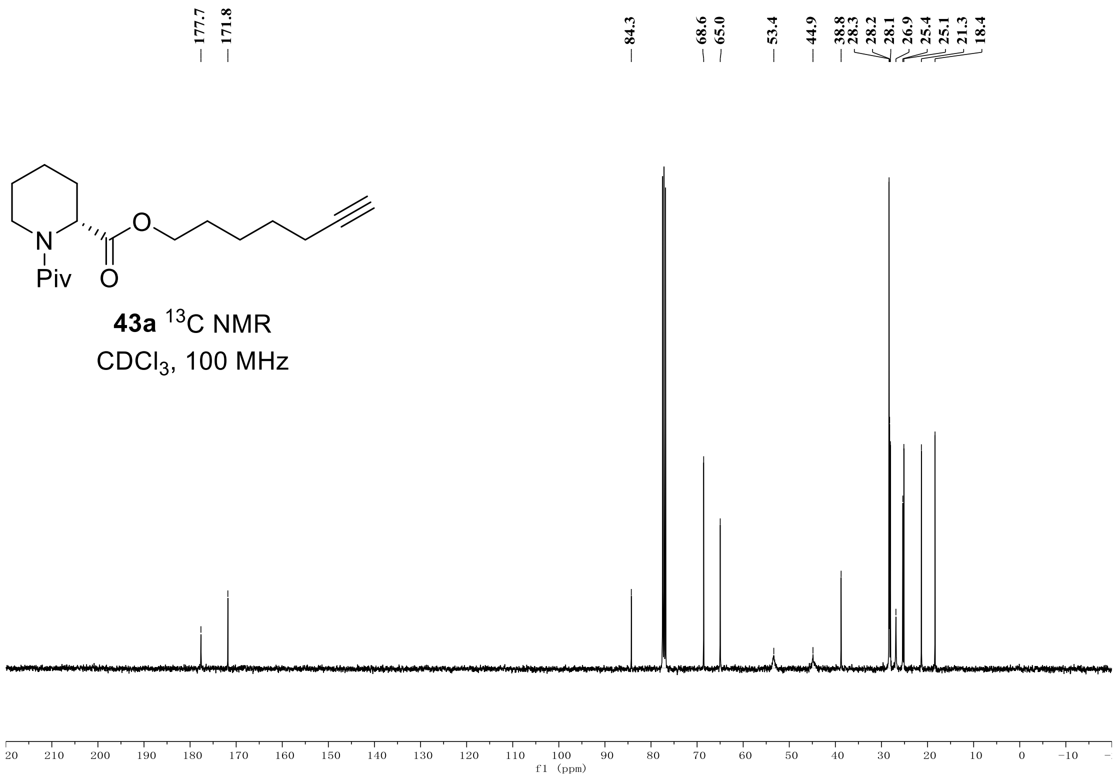

177.7
171.8
84.3
68.6
65.0
53.4
44.9
38.8
28.3
28.2
28.1
26.9
25.4
25.1
21.3
18.4
N
Piv
O
O
43a ^{13}C NMR
$CDCl_3$, 100 MHz
f1 (ppm)

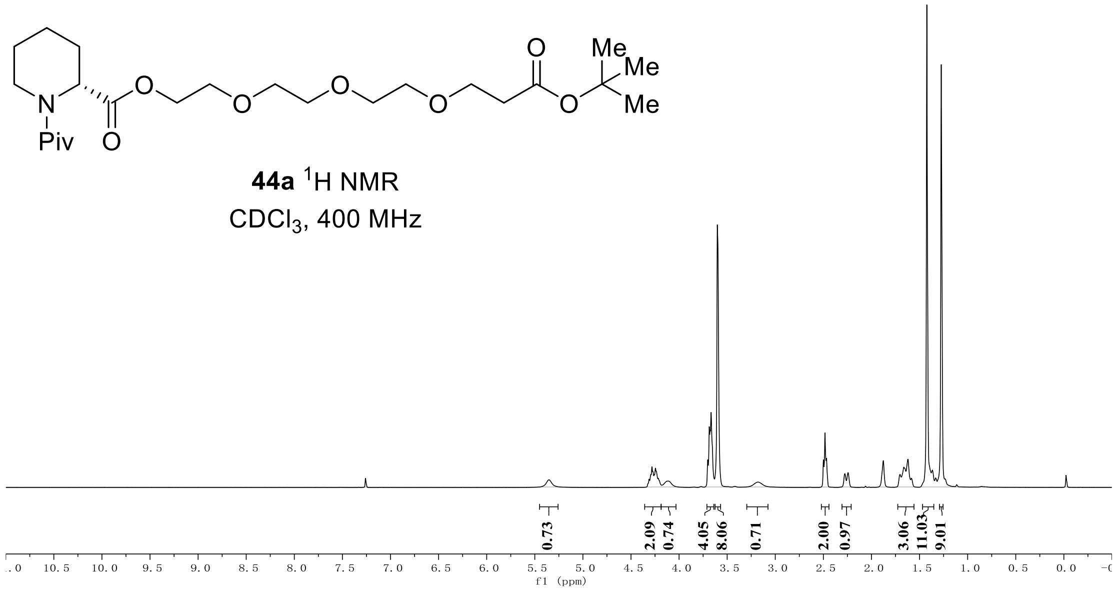

N
Piv
O
O
O
O
O
O
Me
Me
Me
44a ^{1}H NMR
$CDCl_3$, 400 MHz
0.73
2.09
0.74
4.05
8.06
0.71
2.00
0.97
3.06
11.03
9.01
f1 (ppm)

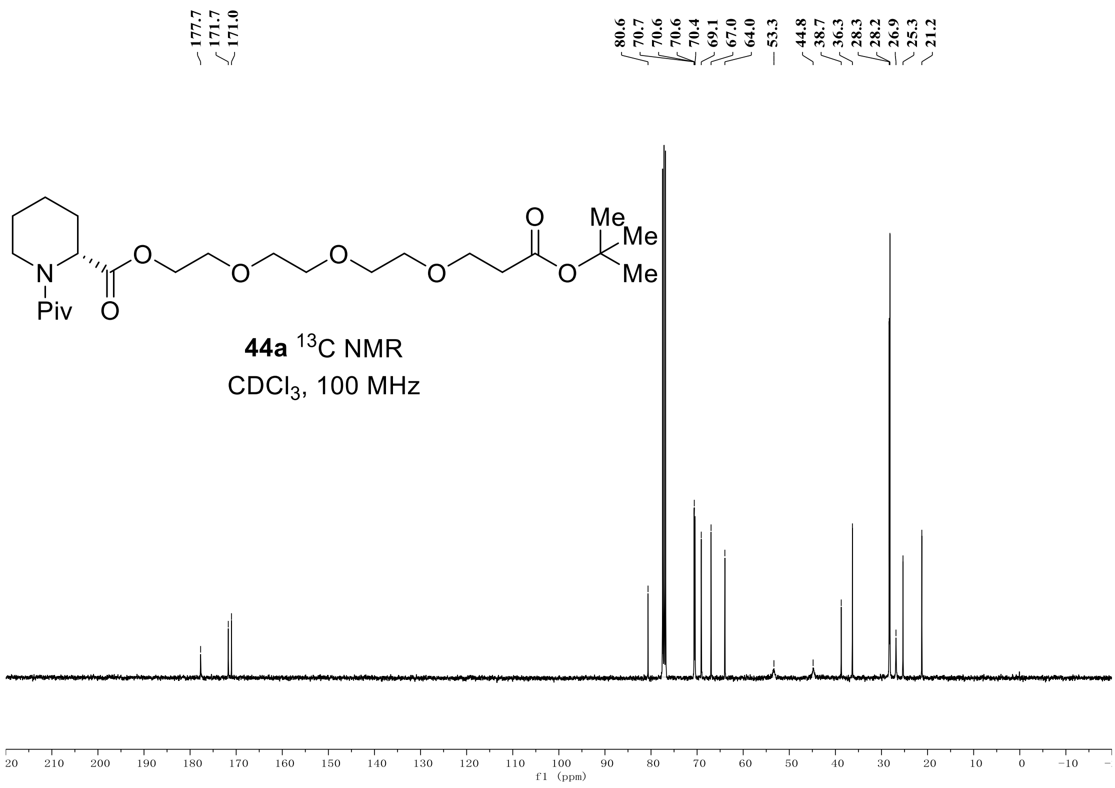
177.7
171.7
171.0
80.6
70.7
70.6
70.6
70.4
69.1
67.0
64.0
53.3
44.8
38.7
36.3
28.3
28.2
26.9
25.3
21.2
N
Piv
O
O
O
O
O
O
O
Me
Me
Me
44a ^{13}C NMR
$CDCl_3$, 100 MHz
f1 (ppm)

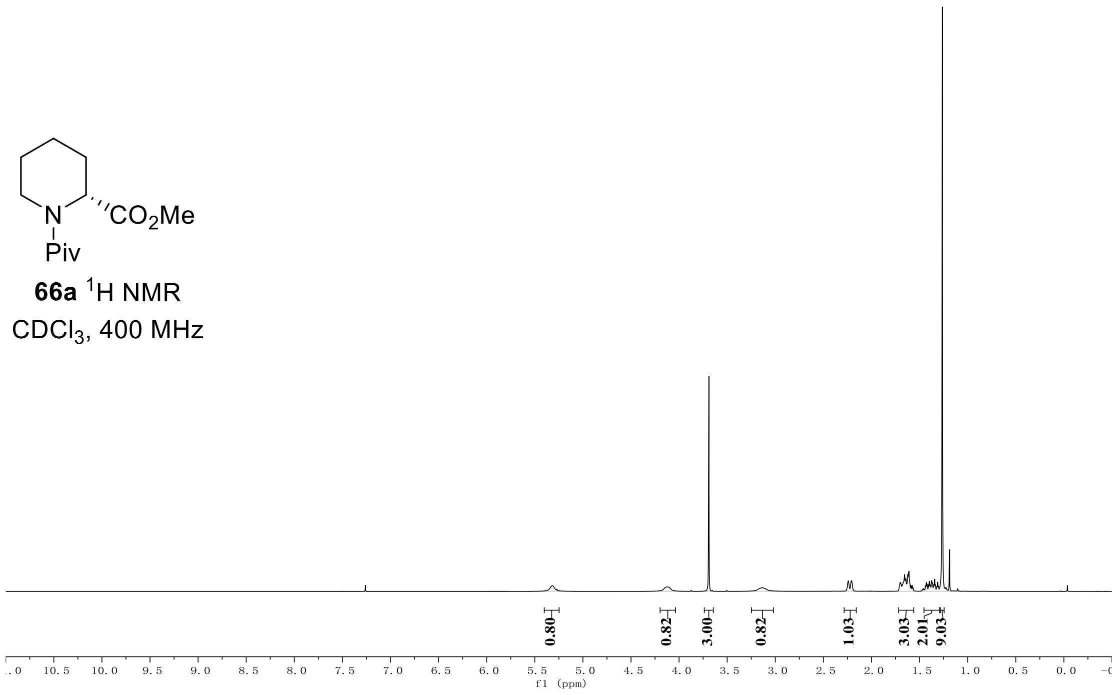
N
Piv
CO_2Me
66a ^{1}H NMR
$CDCl_3$, 400 MHz
0.80
0.82
3.00
0.82
1.03
3.03
2.01
9.03
f1 (ppm)

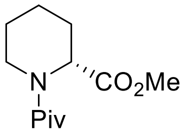

66a ^{13}C NMR
$CDCl_3$, 100 MHz

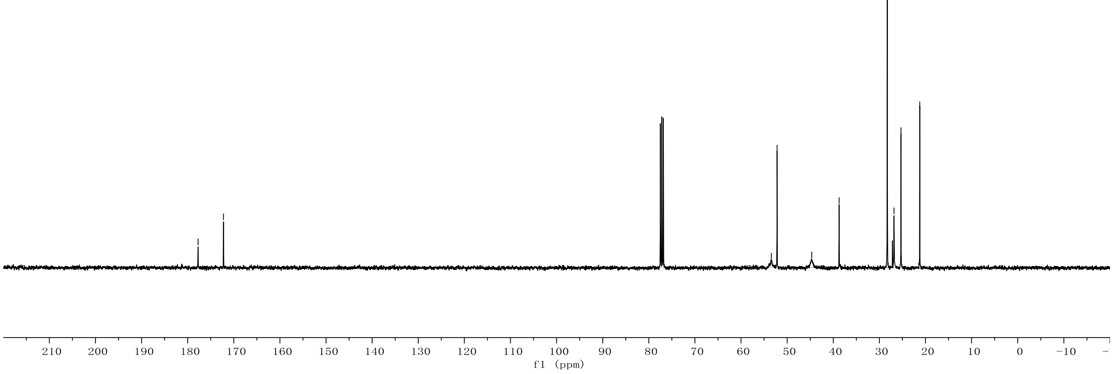

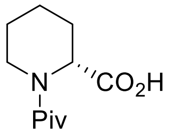

70a ^{1}H NMR
DMSO-d_6, 400 MHz

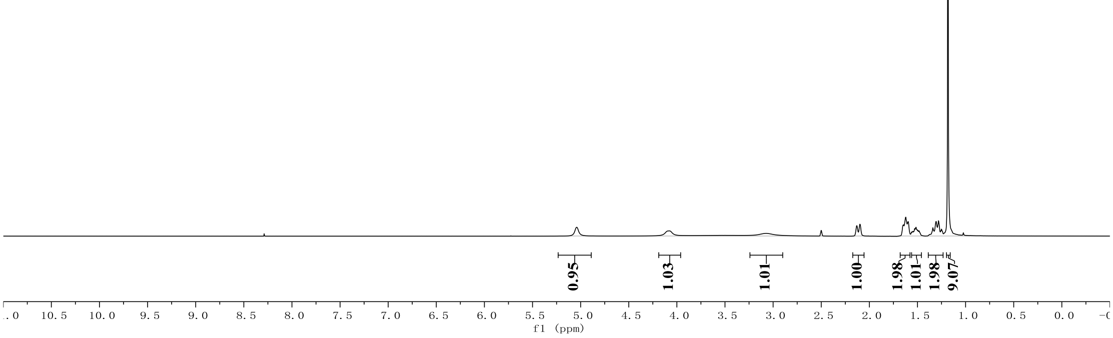

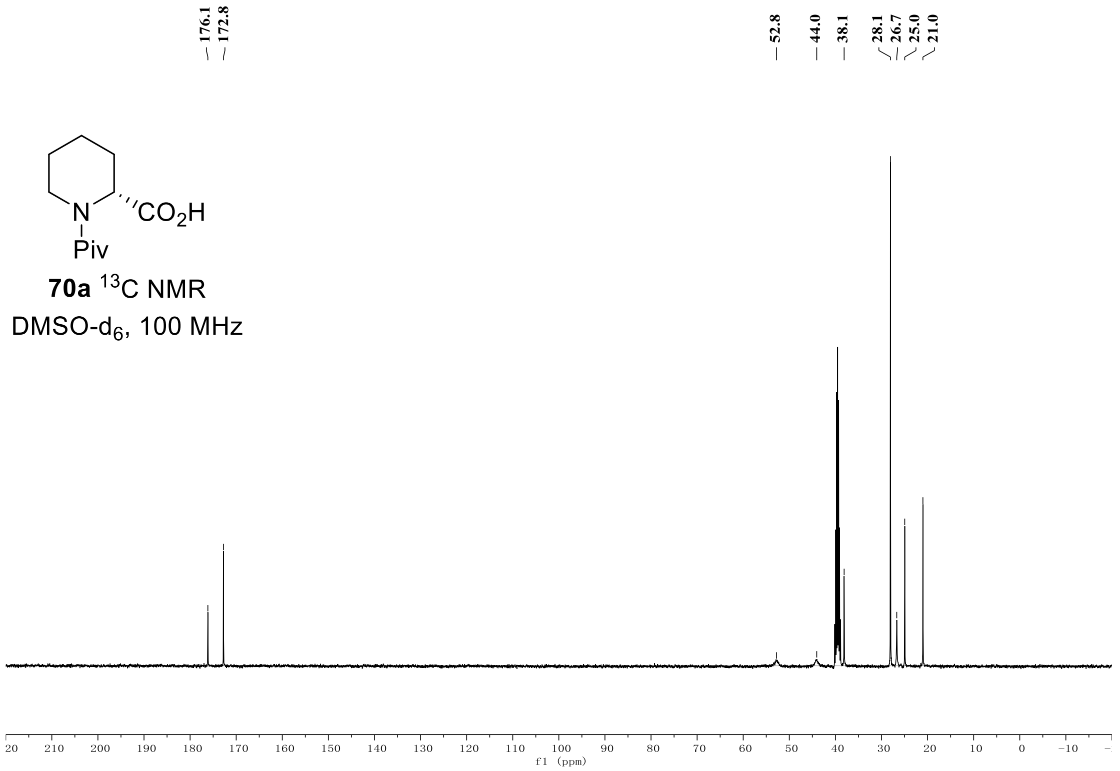

176.1
172.8
52.8
44.0
38.1
28.1
26.7
25.0
21.0
CO2H
N
Piv
70a 13C NMR
DMSO-d6, 100 MHz
f1 (ppm)

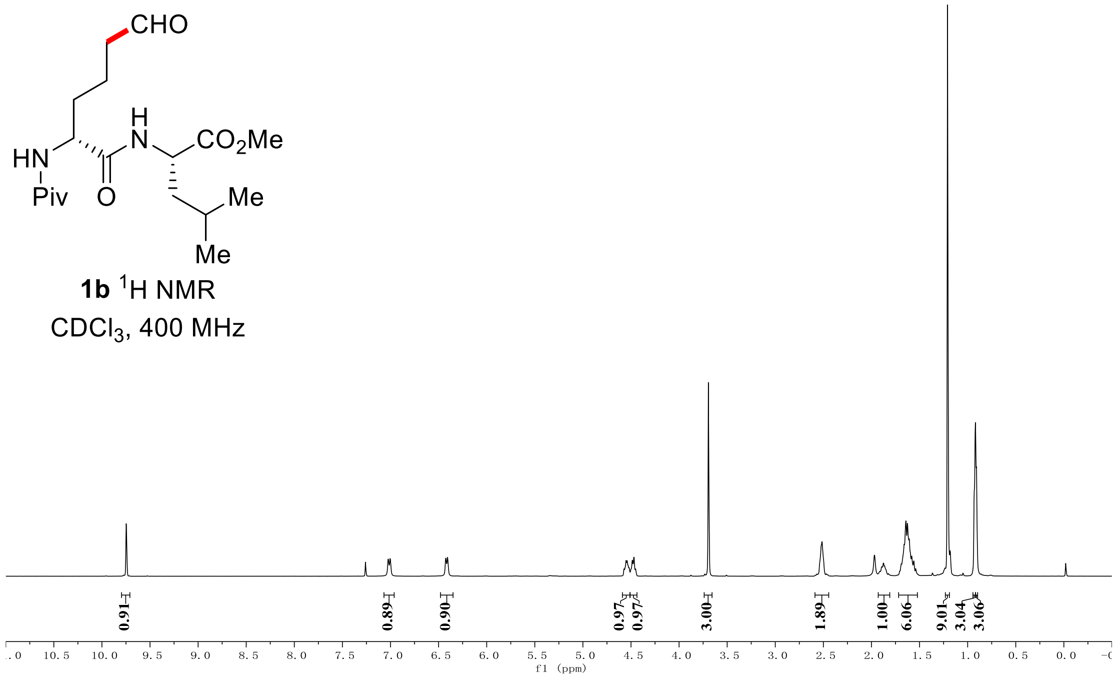
CHO
HN
Piv
O
H
N
CO_2Me
Me
Me
1b ^{1}H NMR
$CDCl_3$, 400 MHz
0.91
0.89
0.90
0.97
0.97
3.00
1.89
1.00
6.06
9.01
3.04
3.06
f1 (ppm)

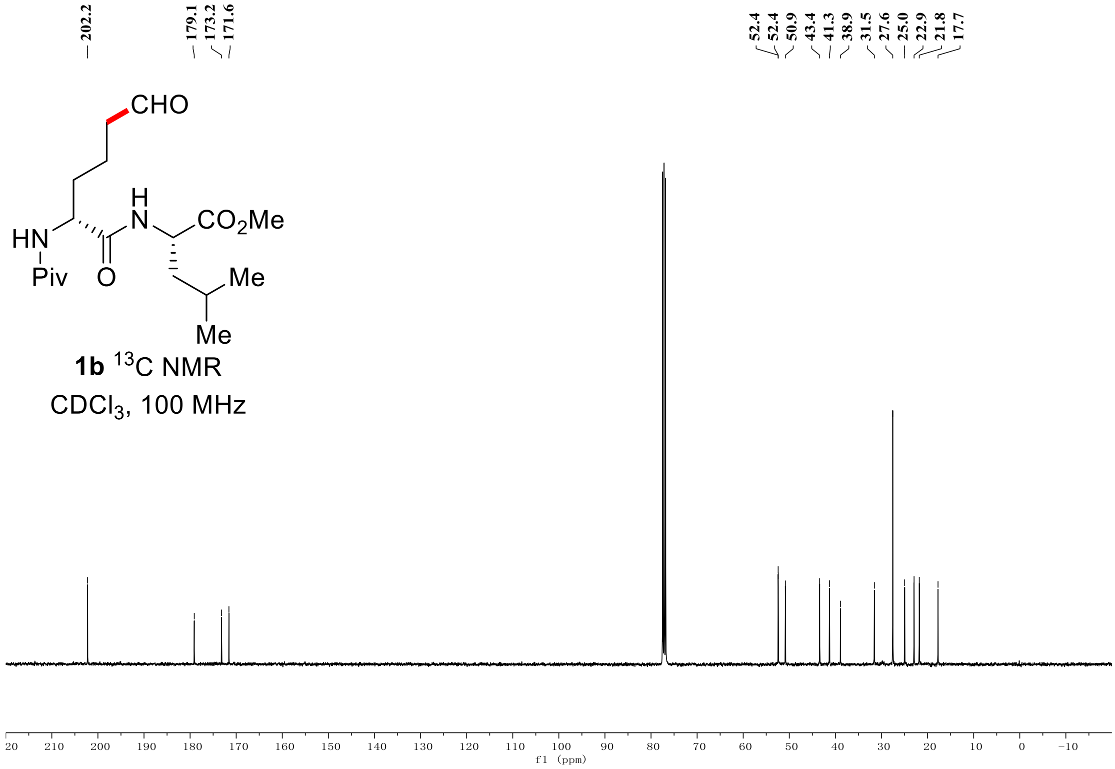
202.2
179.1
173.2
171.6
52.4
52.4
50.9
43.4
41.3
38.9
31.5
27.6
25.0
22.9
21.8
17.7
CHO
HN
Piv
O
H
N
CO_2Me
Me
Me
1b ^{13}C NMR
$CDCl_3$, 100 MHz
f1 (ppm)

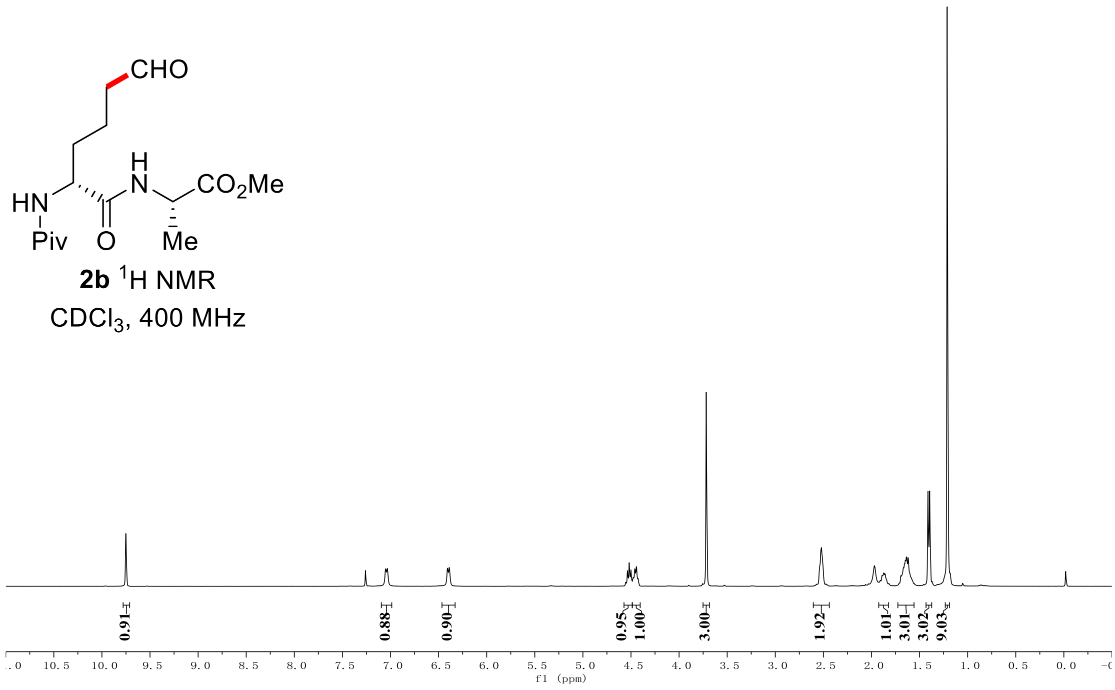
CHO
HN
Piv
H
N
O
CO2Me
Me
2b 1H NMR
CDCl3, 400 MHz
0.91
0.88
0.90
0.95
1.00
3.00
1.92
1.01
3.01
3.02
9.03
f1 (ppm)

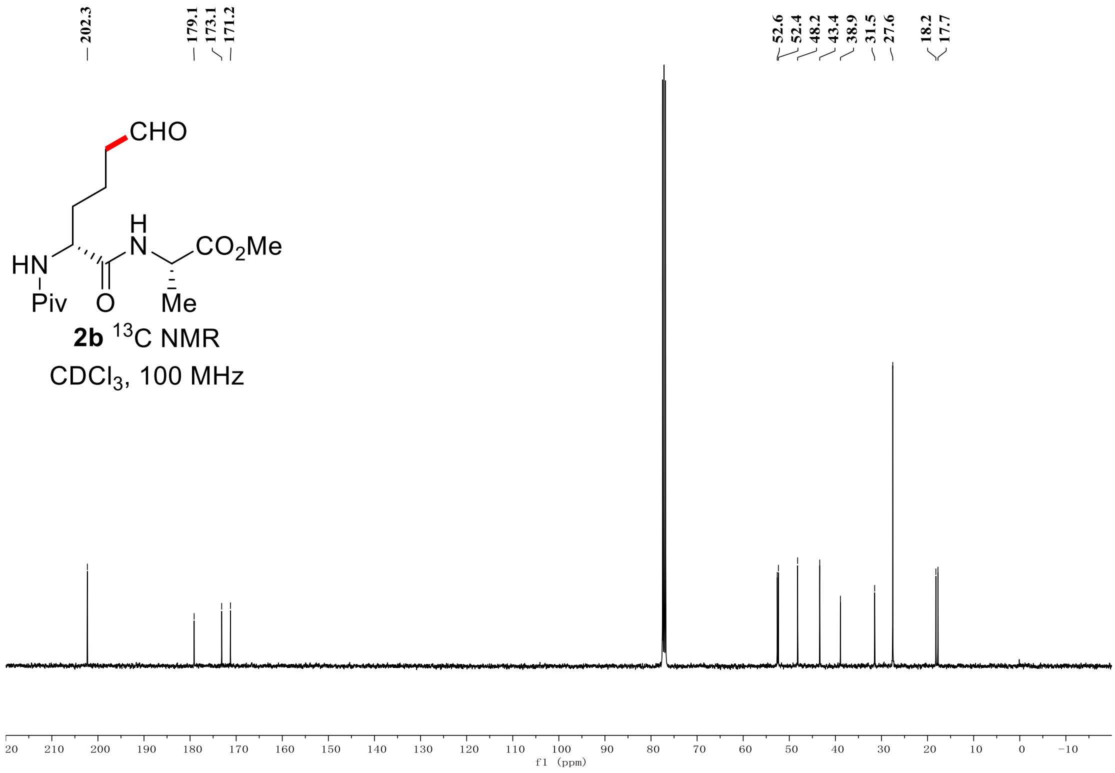
202.3
179.1
173.1
171.2
52.6
52.4
48.2
43.4
38.9
31.5
27.6
18.2
17.7
CHO
HN
Piv
H
N
O
CO2Me
Me
2b 13C NMR
CDCl3, 100 MHz
f1 (ppm)

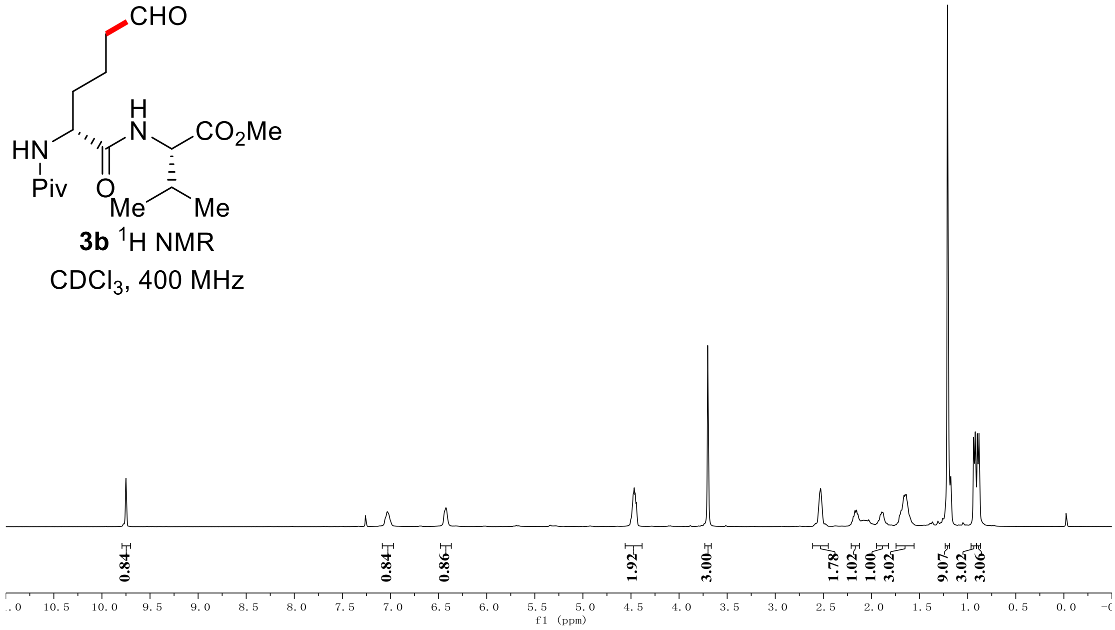
CHO
H
N
CO2Me
HN
Piv
O
Me
Me
3b 1H NMR
CDCl3, 400 MHz
0.84
0.84
0.86
1.92
3.00
1.78
1.02
1.00
3.02
9.07
3.02
3.06
f1 (ppm)

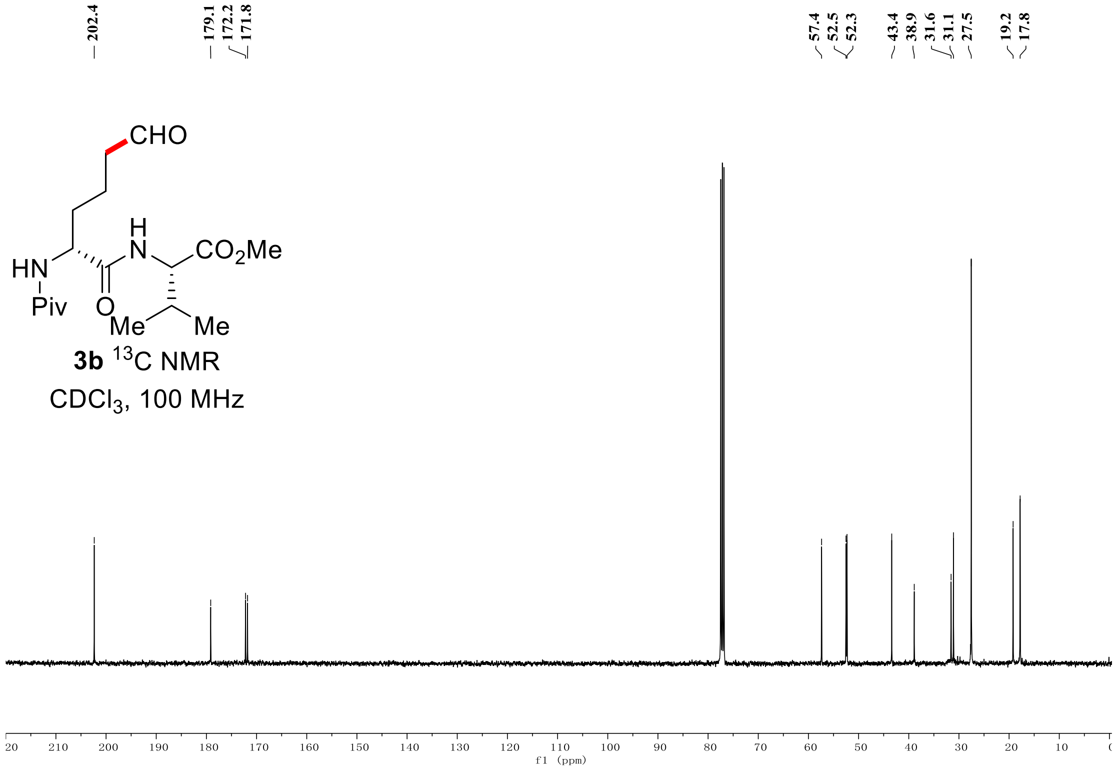
202.4
179.1
172.2
171.8
57.4
52.5
52.3
43.4
38.9
31.6
31.1
27.5
19.2
17.8
CHO
H
N
CO2Me
HN
Piv
O
Me
Me
3b 13C NMR
CDCl3, 100 MHz
f1 (ppm)

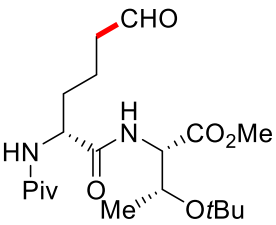

4b ^{1}H NMR

$CDCl_3$, 400 MHz

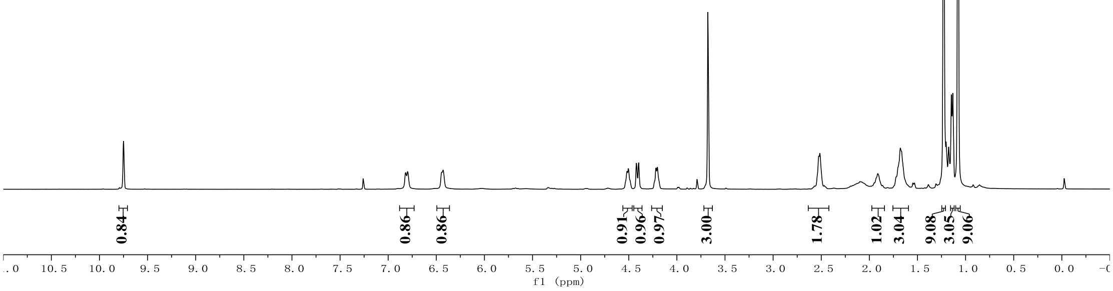

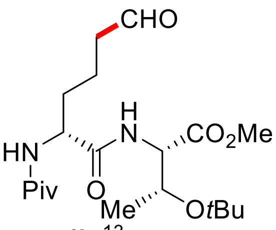

4b ^{13}C NMR

$CDCl_3$, 100 MHz

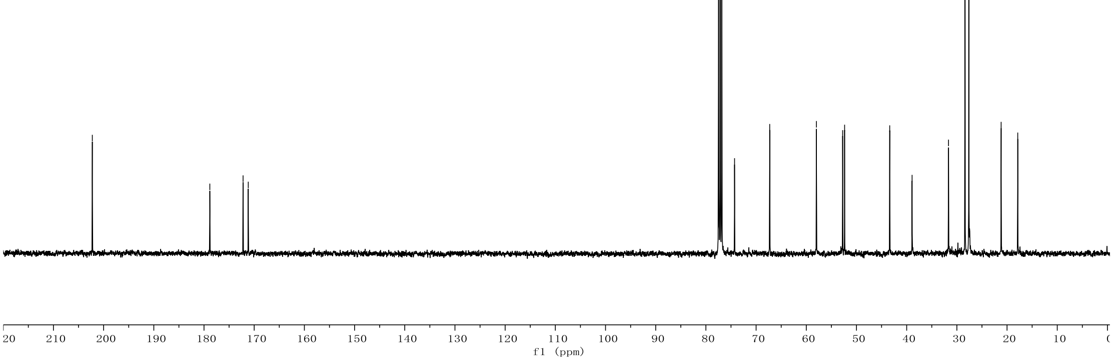

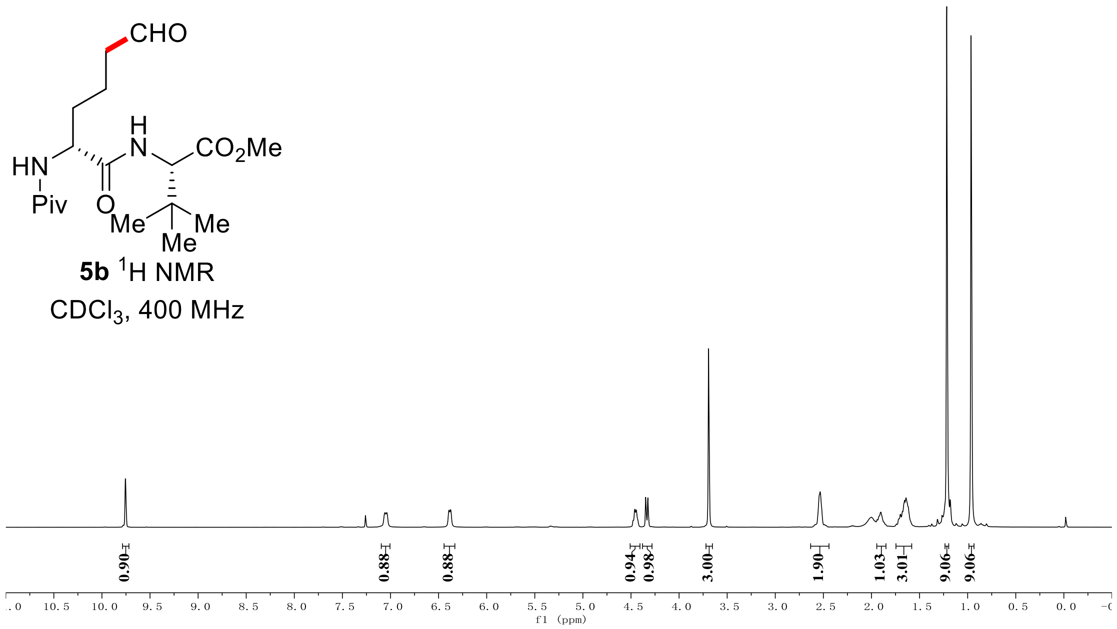
CHO
H
N
CO2Me
HN
Piv
O
Me
Me
Me
5b 1H NMR
CDCl3, 400 MHz
0.90
0.88
0.88
0.94
0.98
3.00
1.90
1.03
3.01
9.06
9.06
f1 (ppm)

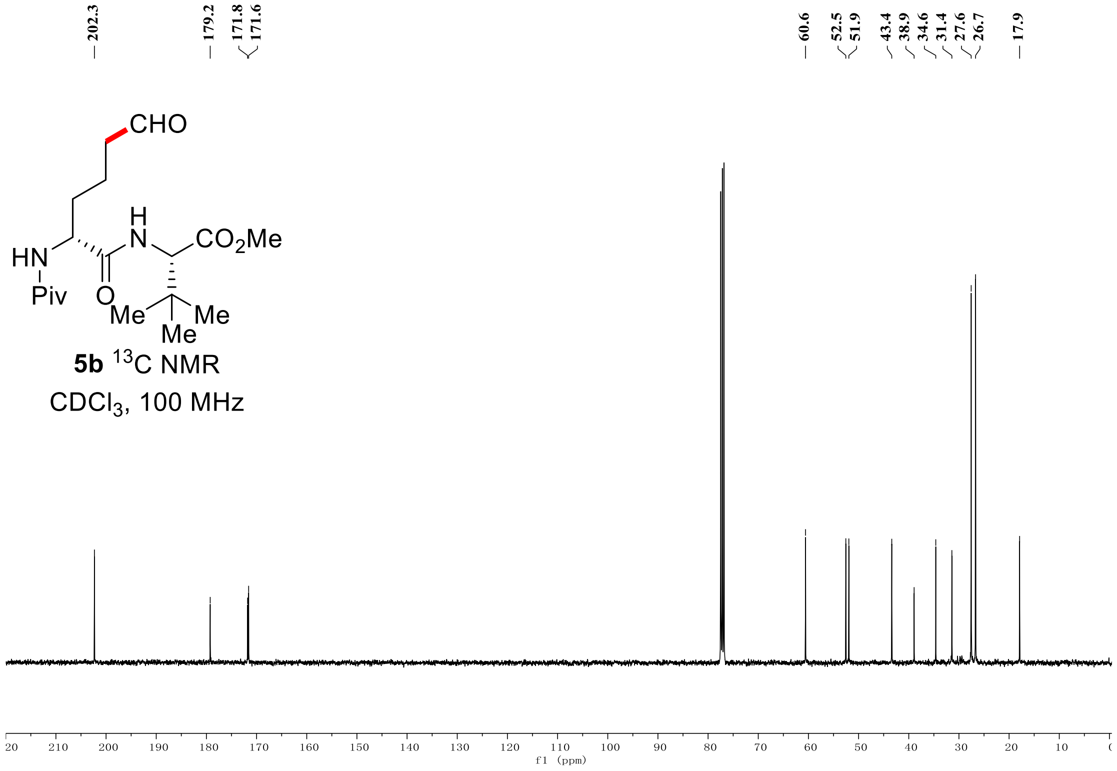
202.3
179.2
171.8
171.6
60.6
52.5
51.9
43.4
38.9
34.6
31.4
27.6
26.7
17.9
CHO
H
N
CO2Me
HN
Piv
O
Me
Me
Me
5b 13C NMR
CDCl3, 100 MHz
f1 (ppm)

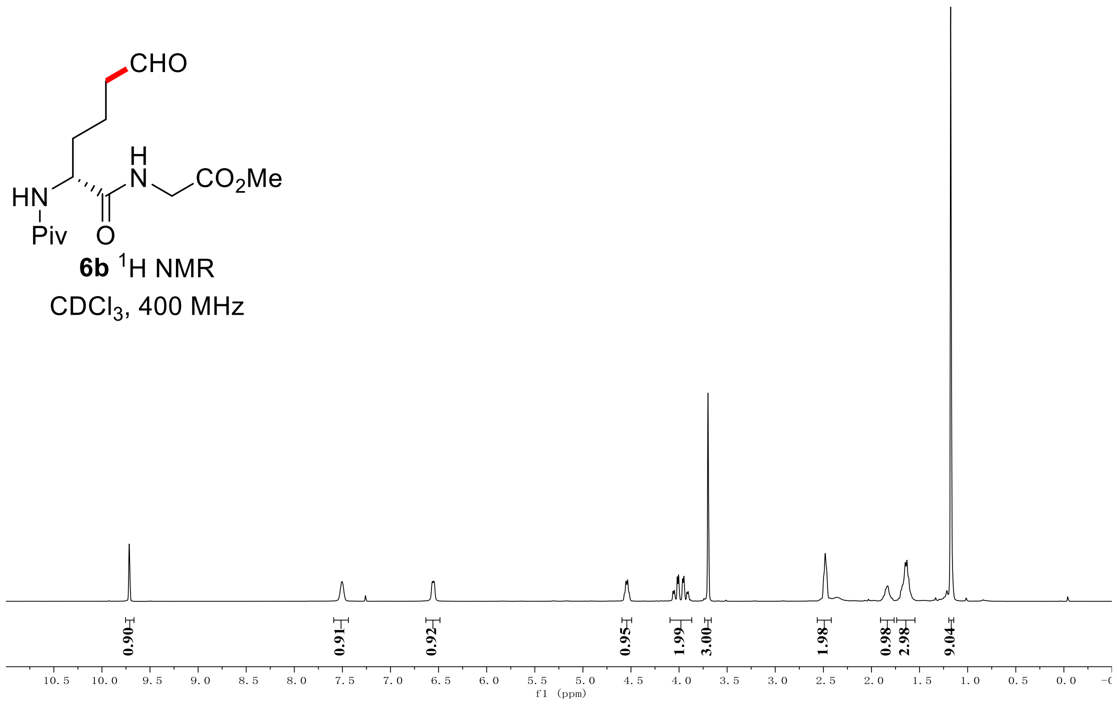
CHO
H
N
CO2Me
HN
Piv
O
6b 1H NMR
CDCl3, 400 MHz
0.90
0.91
0.92
0.95
1.99
3.00
1.98
0.98
2.98
9.04
f1 (ppm)

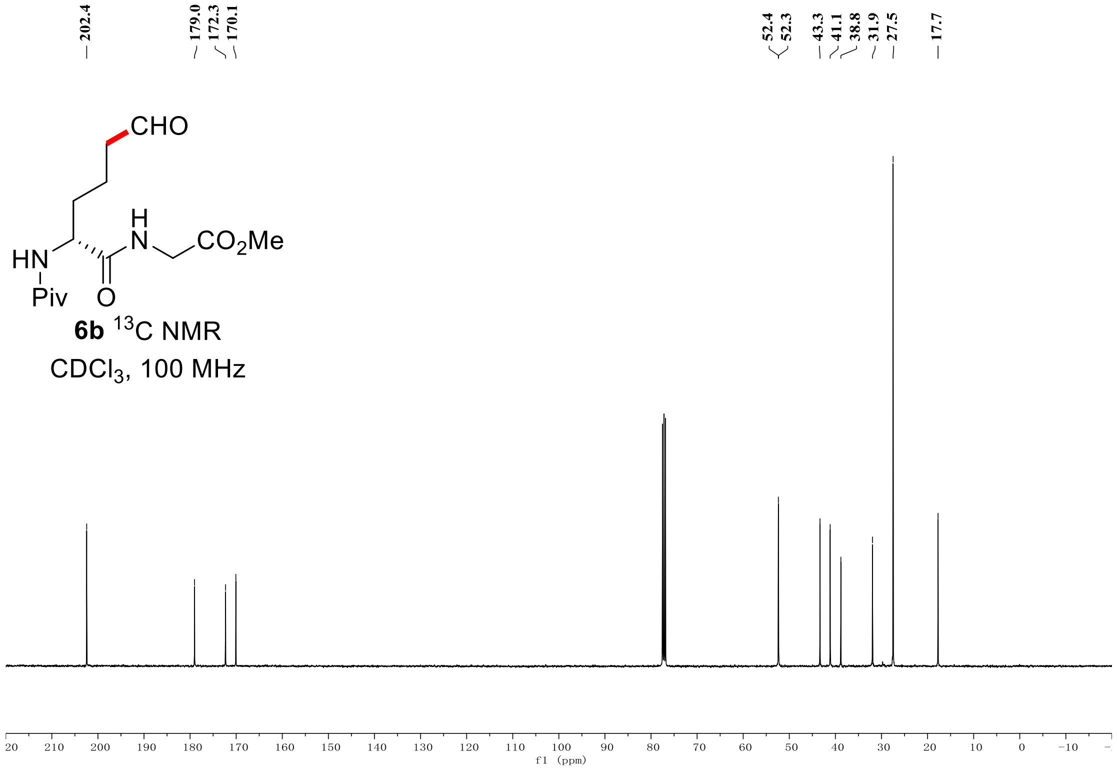
202.4
179.0
172.3
170.1
52.4
52.3
43.3
41.1
38.8
31.9
27.5
17.7
CHO
H
N
CO2Me
HN
Piv
O
6b 13C NMR
CDCl3, 100 MHz
f1 (ppm)

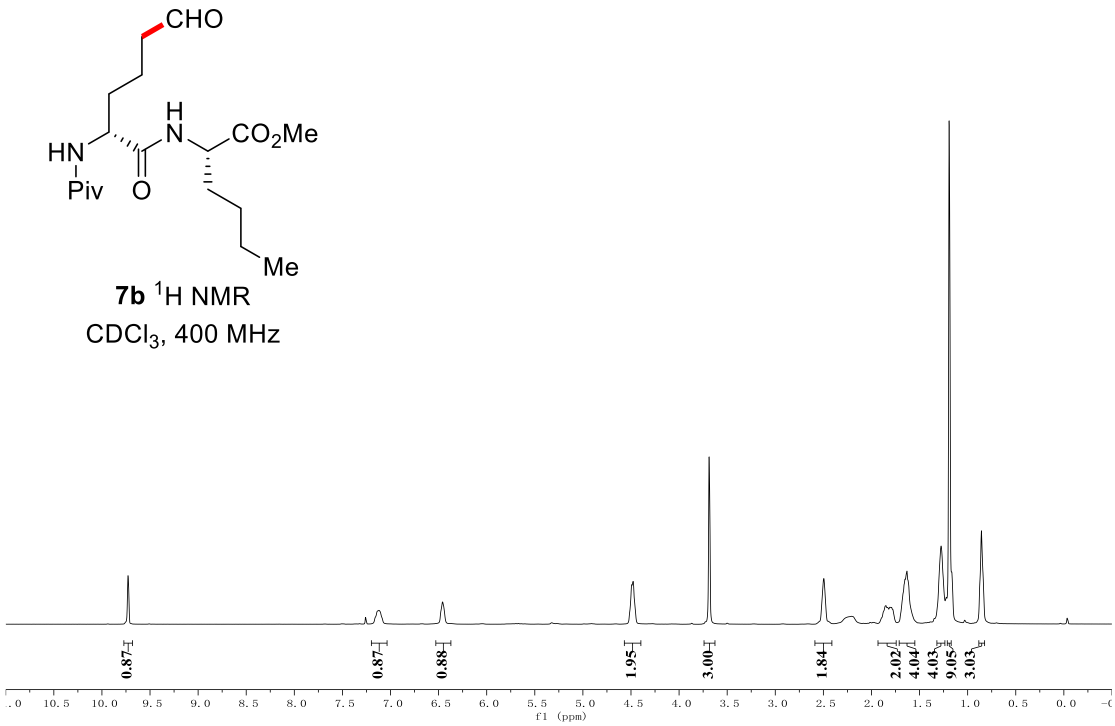
CHO
HN
Piv
O
H
N
CO_2Me
Me
7b ^{1}H NMR
$CDCl_3$, 400 MHz
0.87
0.87
0.88
1.95
3.00
1.84
2.02
4.04
4.03
9.05
3.03
f1 (ppm)

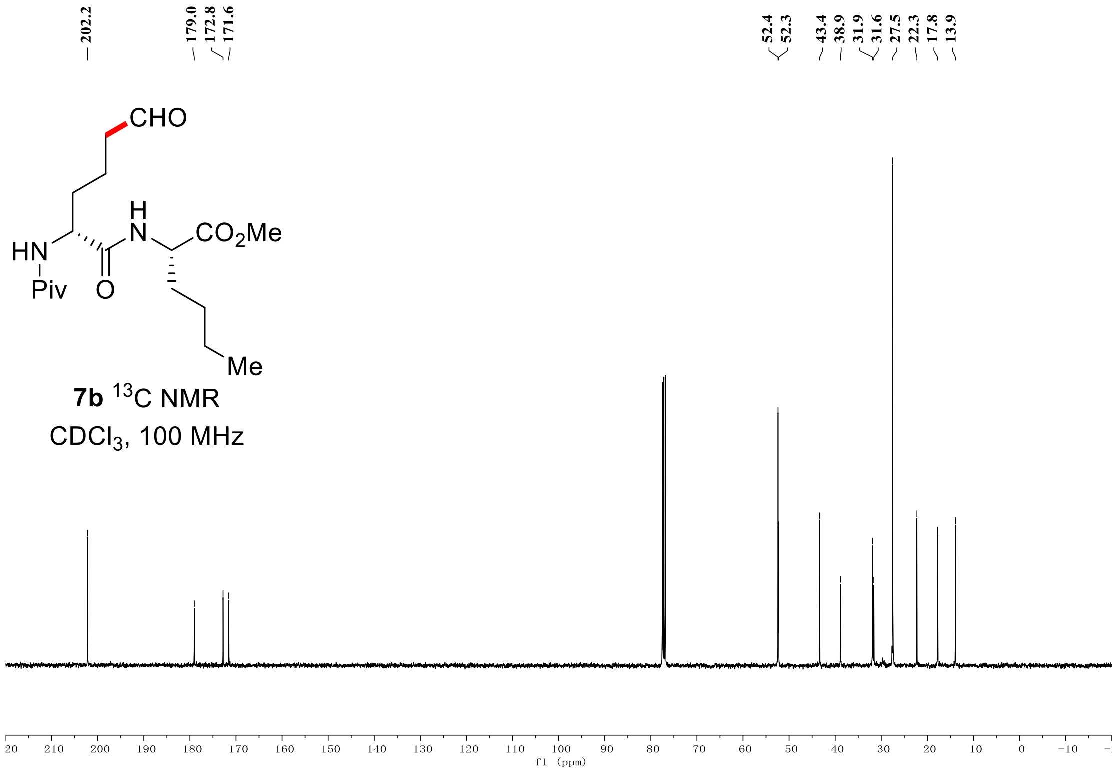
202.2
179.0
172.8
171.6
52.4
52.3
43.4
38.9
31.9
31.6
27.5
22.3
17.8
13.9
CHO
HN
Piv
O
H
N
CO_2Me
Me
7b ^{13}C NMR
$CDCl_3$, 100 MHz
f1 (ppm)

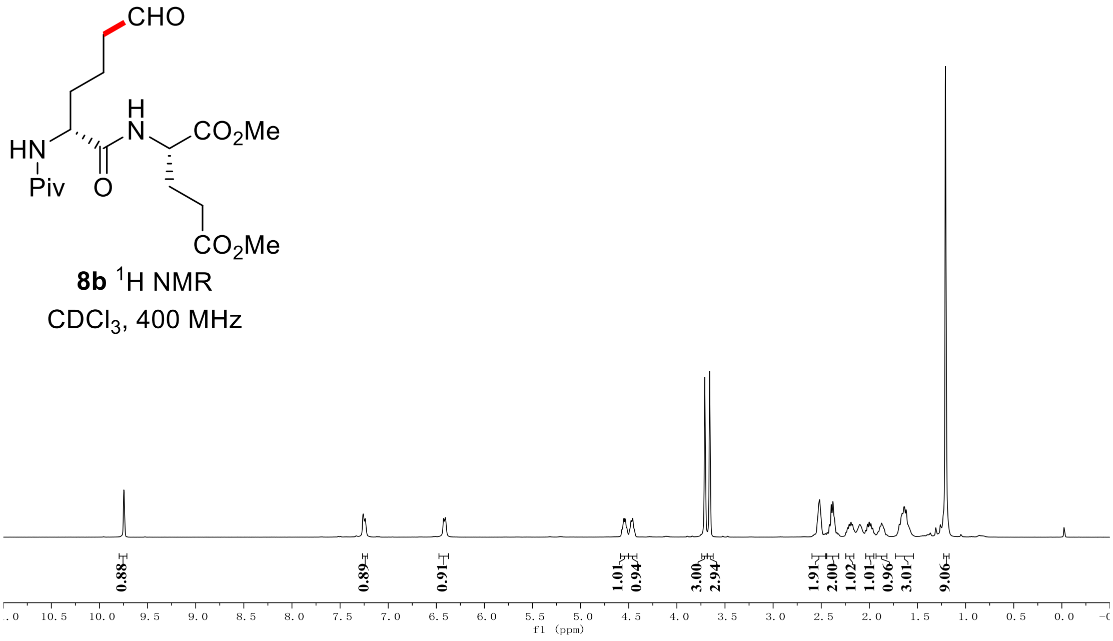
CHO
CO_2Me
CO_2Me
HN
Piv
O
8b ^{1}H NMR
$CDCl_3$, 400 MHz
0.88
0.89
0.91
1.01
0.94
3.00
2.94
1.91
2.00
1.02
1.01
0.96
3.01
9.06
f1 (ppm)

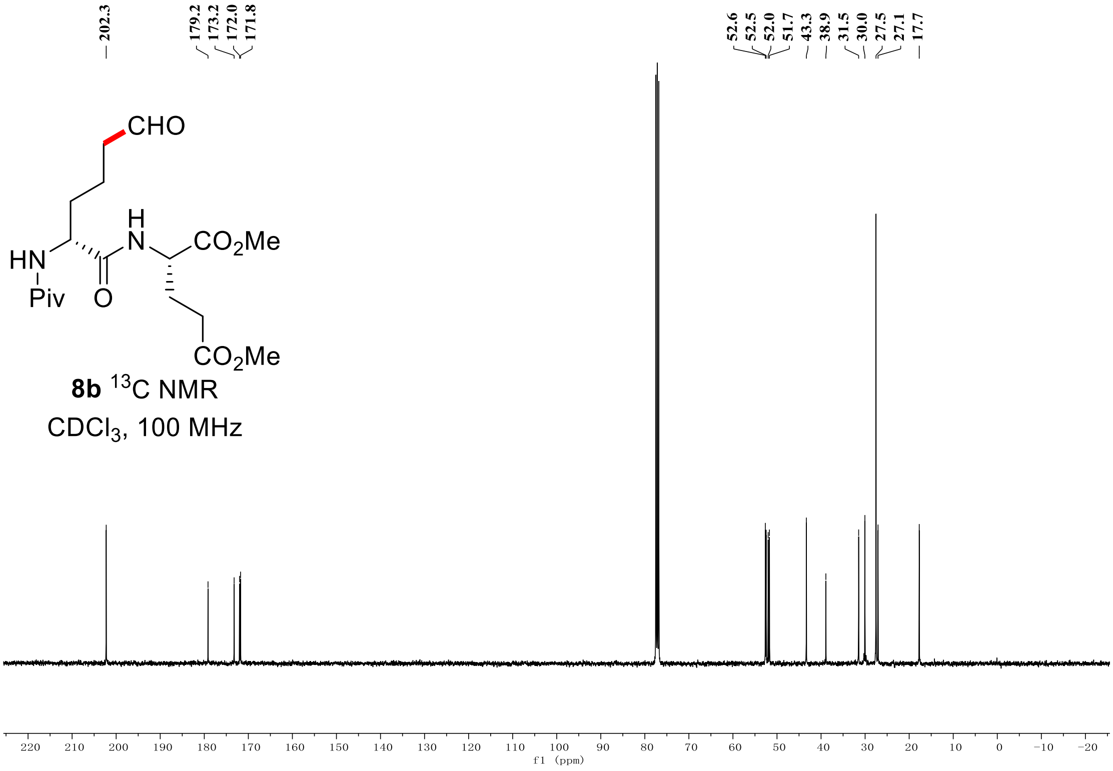
202.3
179.2
173.2
172.0
171.8
52.6
52.5
52.0
51.7
43.3
38.9
31.5
30.0
27.5
27.1
17.7
CHO
CO_2Me
CO_2Me
HN
Piv
O
8b ^{13}C NMR
$CDCl_3$, 100 MHz
f1 (ppm)

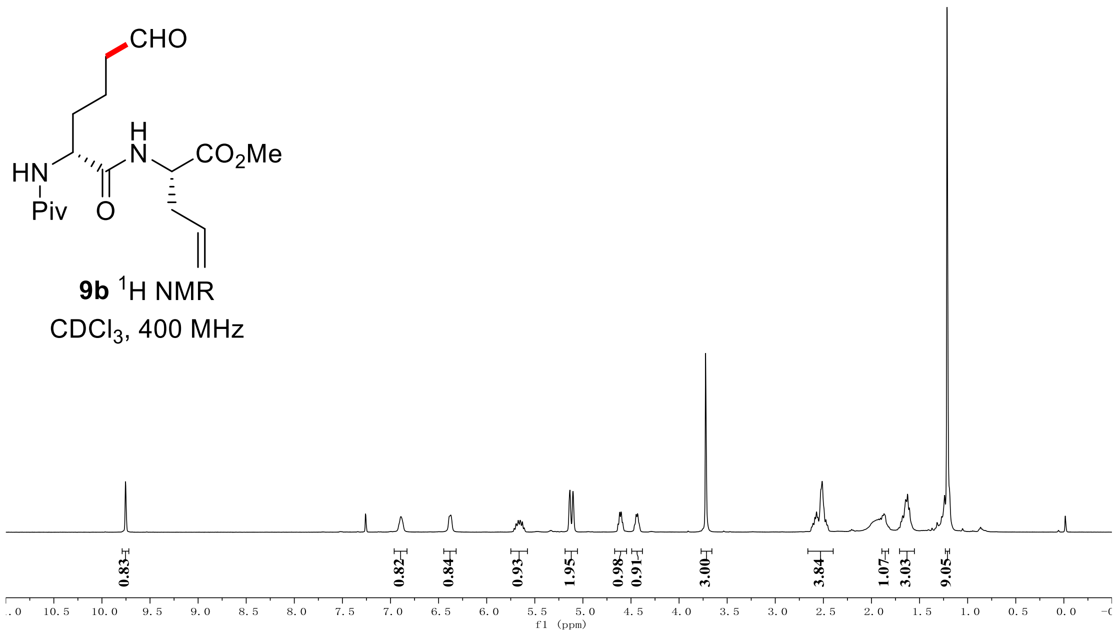
CHO
HN
Piv
O
H
N
CO_2Me
9b ^1H NMR
$CDCl_3$, 400 MHz
0.83
0.82
0.84
0.93
1.95
0.98
0.91
3.00
3.84
1.07
3.03
9.05
f1 (ppm)

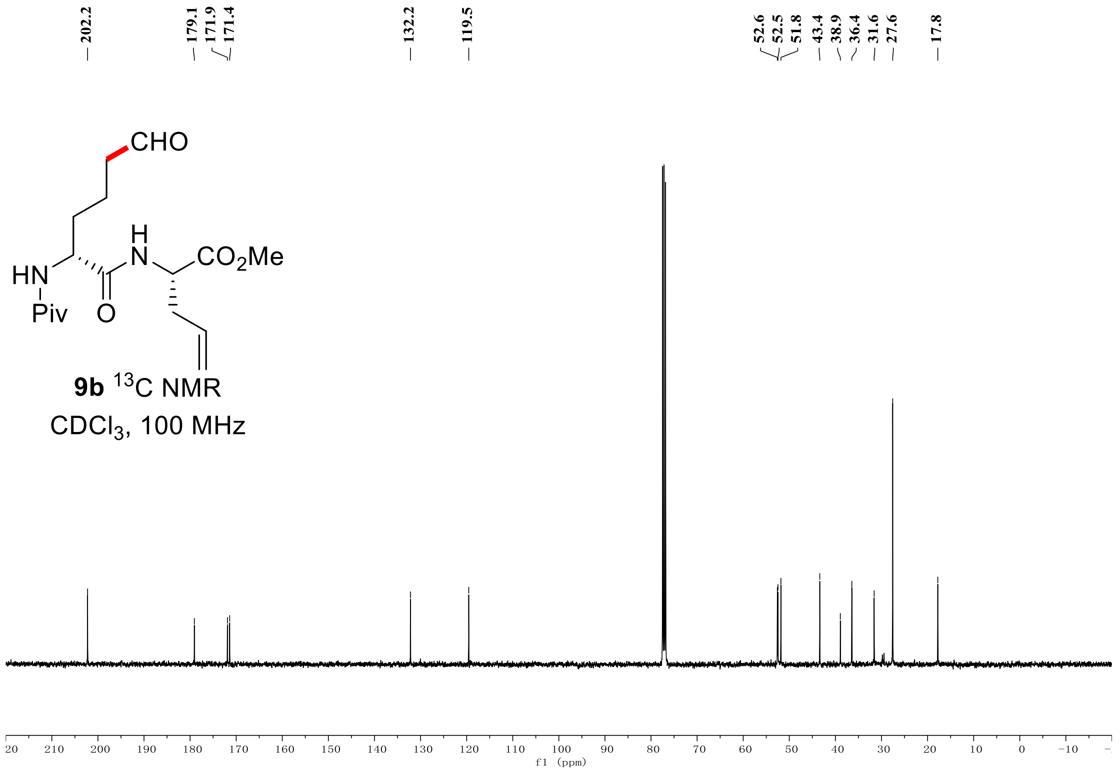
202.2
179.1
171.9
171.4
132.2
119.5
52.6
52.5
51.8
43.4
38.9
36.4
31.6
27.6
17.8
CHO
HN
Piv
O
H
N
CO_2Me
9b ^{13}C NMR
$CDCl_3$, 100 MHz
f1 (ppm)

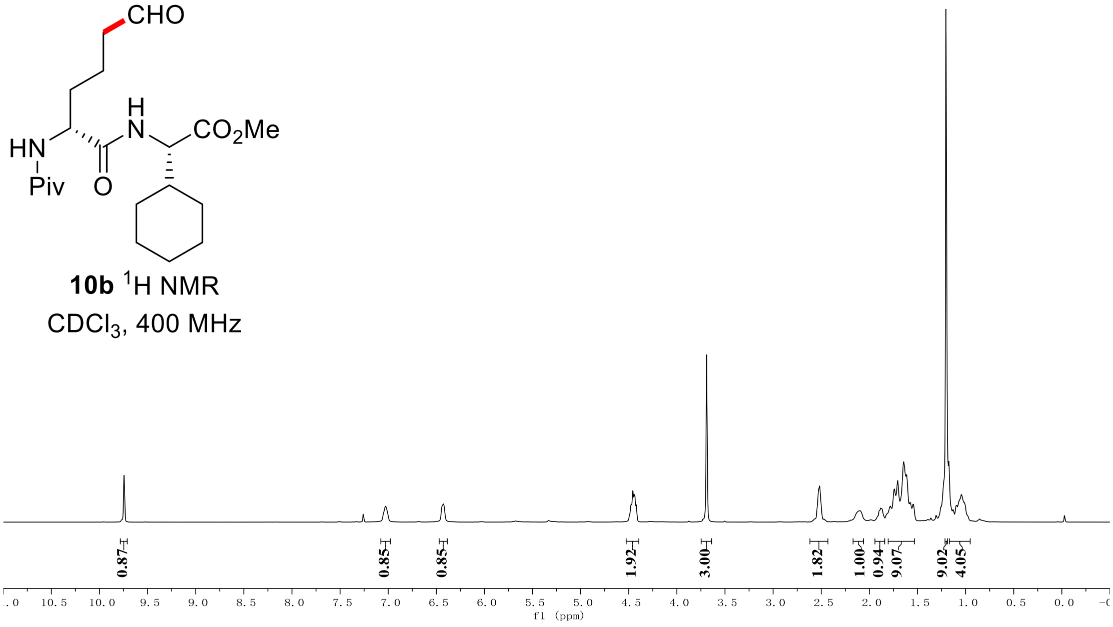
CHO
H
N
CO2Me
HN
Piv
O
10b 1H NMR
CDCl3, 400 MHz
0.87
0.85
0.85
1.92
3.00
1.82
1.00
0.94
9.07
9.02
4.05
f1 (ppm)

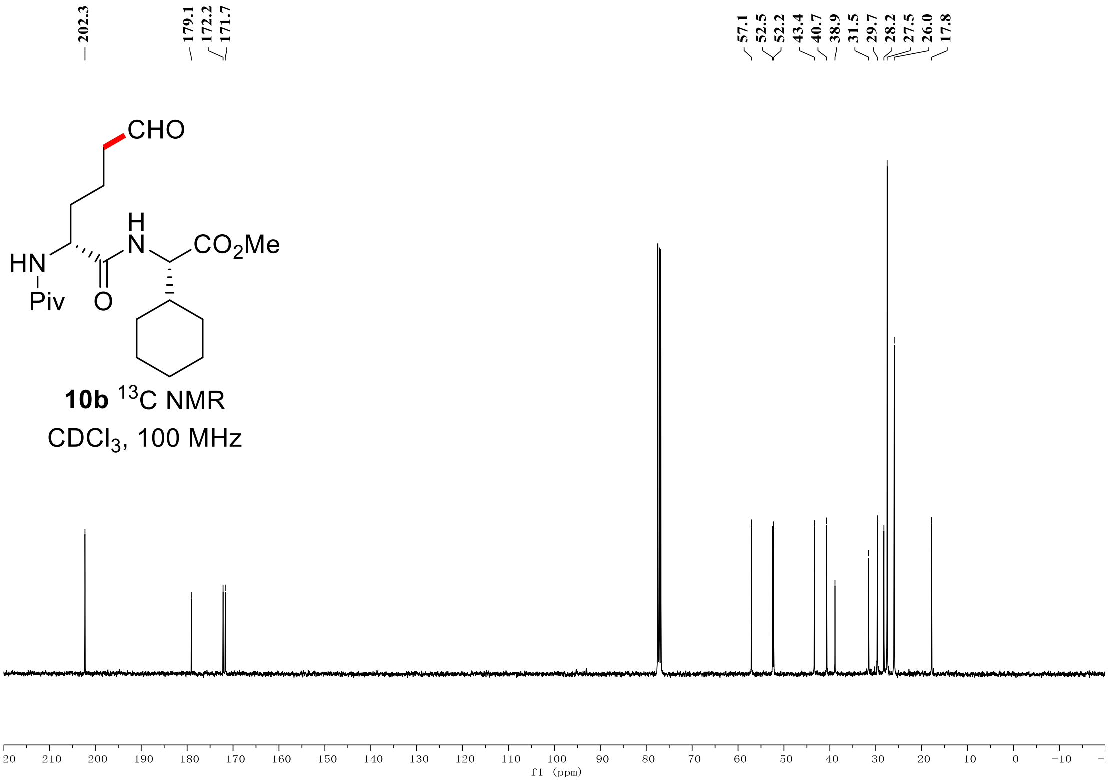
202.3
179.1
172.2
171.7
57.1
52.5
52.2
43.4
40.7
38.9
31.5
29.7
28.2
27.5
26.0
17.8
CHO
H
N
CO2Me
HN
Piv
O
10b 13C NMR
CDCl3, 100 MHz
f1 (ppm)

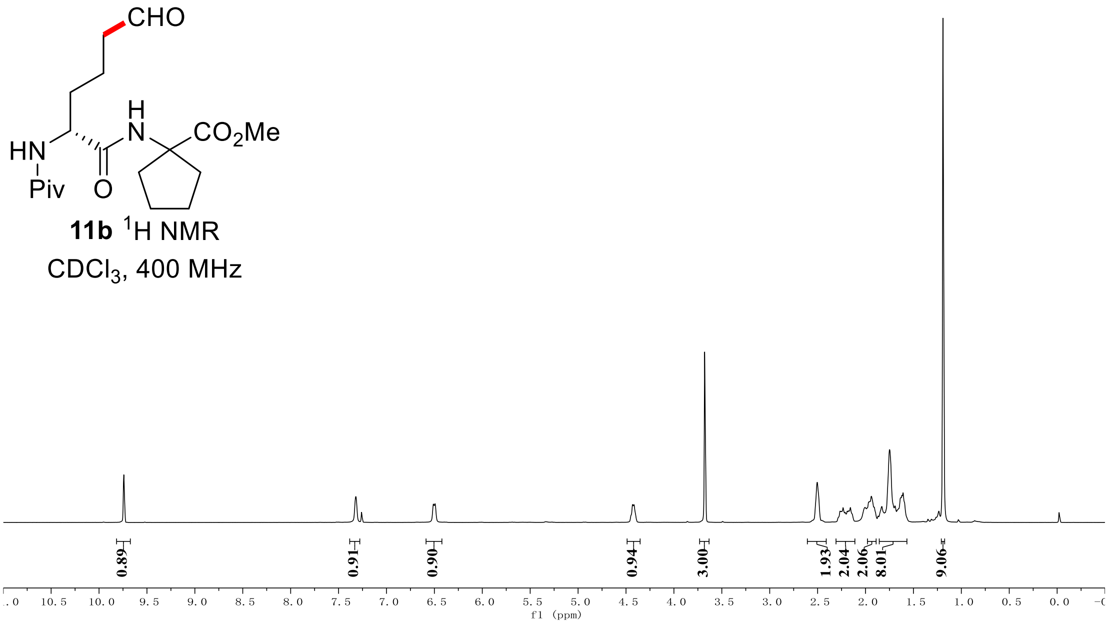
CHO
HN
Piv
O
H
N
CO_2Me
11b ¹H NMR
$CDCl_3$, 400 MHz
0.89
0.91
0.90
0.94
3.00
1.93
2.04
2.06
8.01
9.06
f1 (ppm)

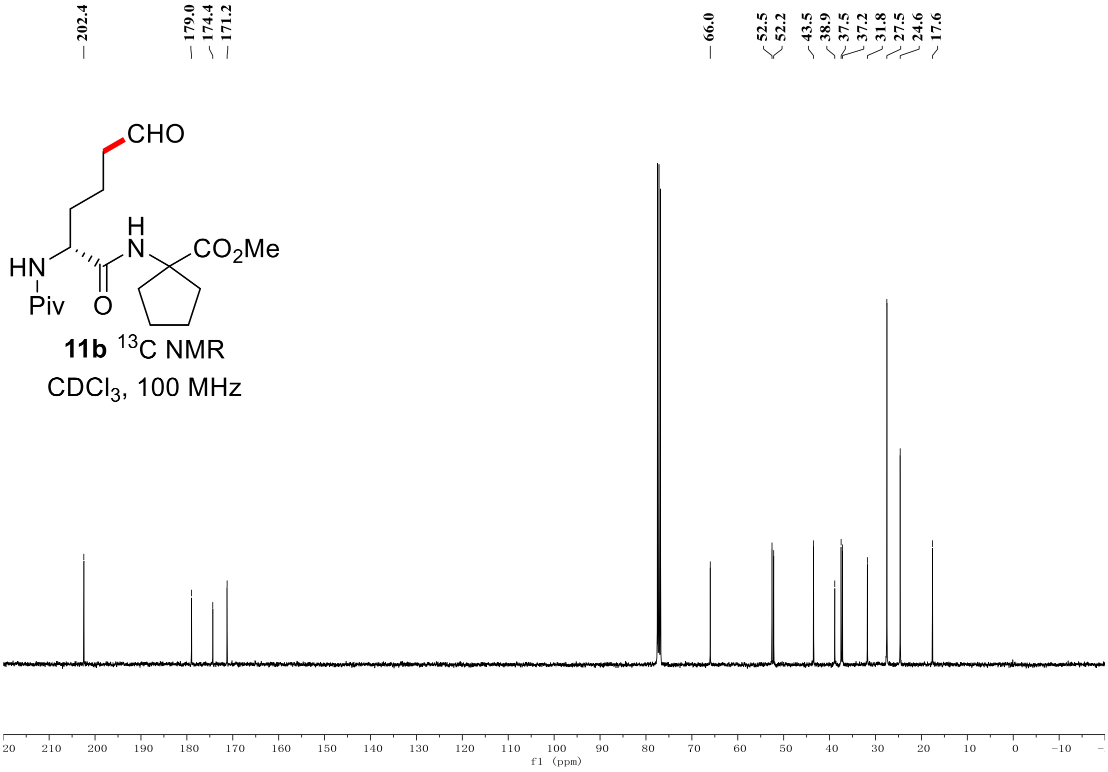
202.4
179.0
174.4
171.2
66.0
52.5
52.2
43.5
38.9
37.5
37.2
31.8
27.5
24.6
17.6
CHO
HN
Piv
O
H
N
CO_2Me
11b ¹³C NMR
$CDCl_3$, 100 MHz
f1 (ppm)

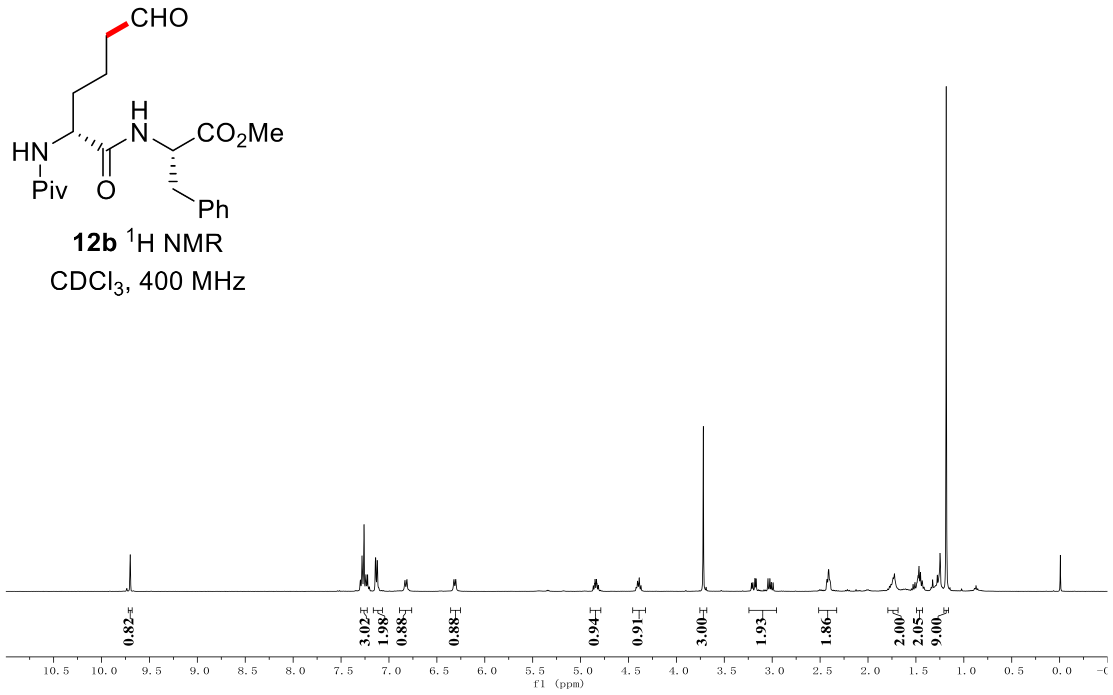
CHO
HN
H
N
CO2Me
Piv
O
Ph
12b 1H NMR
CDCl3, 400 MHz
0.82
3.02
1.98
0.88
0.88
0.94
0.91
3.00
1.93
1.86
2.00
2.05
9.00
f1 (ppm)

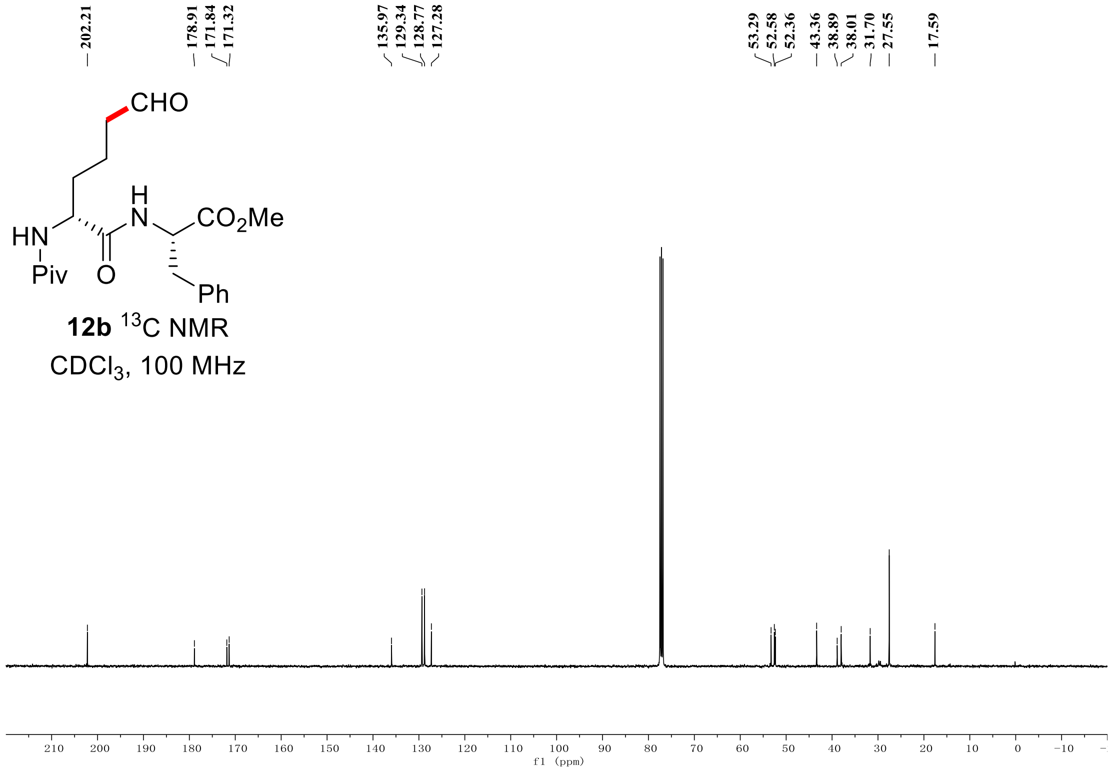
202.21
178.91
171.84
171.32
135.97
129.34
128.77
127.28
53.29
52.58
52.36
43.36
38.89
38.01
31.70
27.55
17.59
CHO
HN
H
N
CO2Me
Piv
O
Ph
12b 13C NMR
CDCl3, 100 MHz
f1 (ppm)

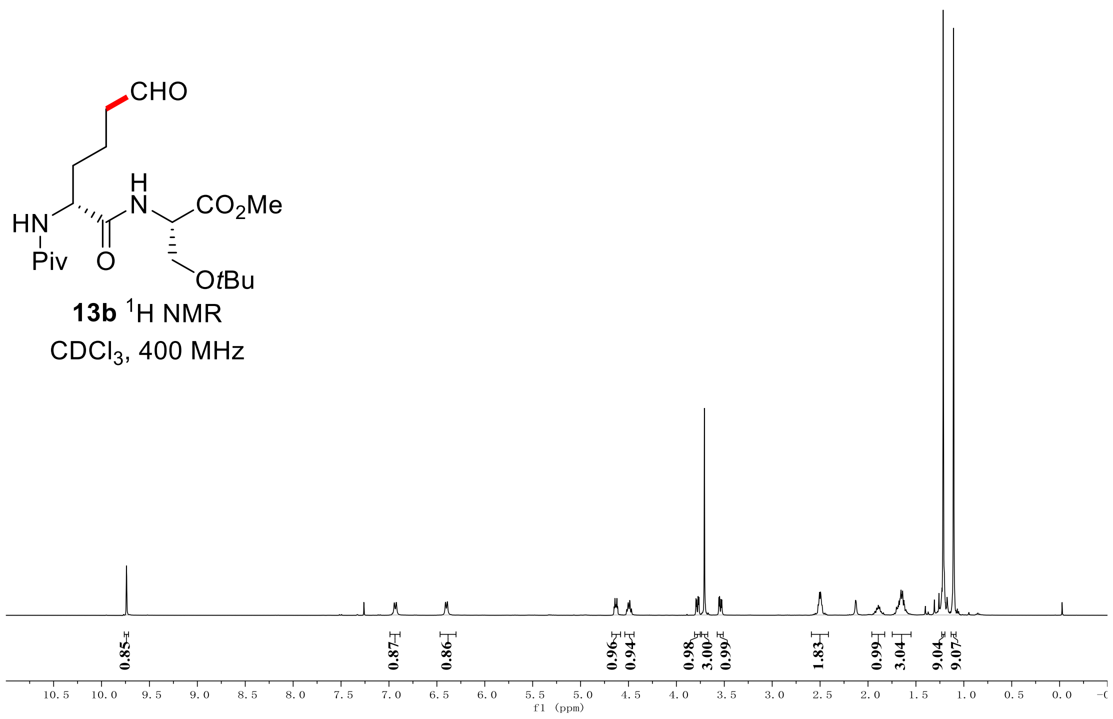
CHO
HN
Piv
O
H
N
CO_2Me
OtBu
13b ¹H NMR
$CDCl_3$, 400 MHz
0.85
0.87
0.86
0.96
0.94
0.98
3.00
0.99
1.83
0.99
3.04
9.04
9.07
f1 (ppm)

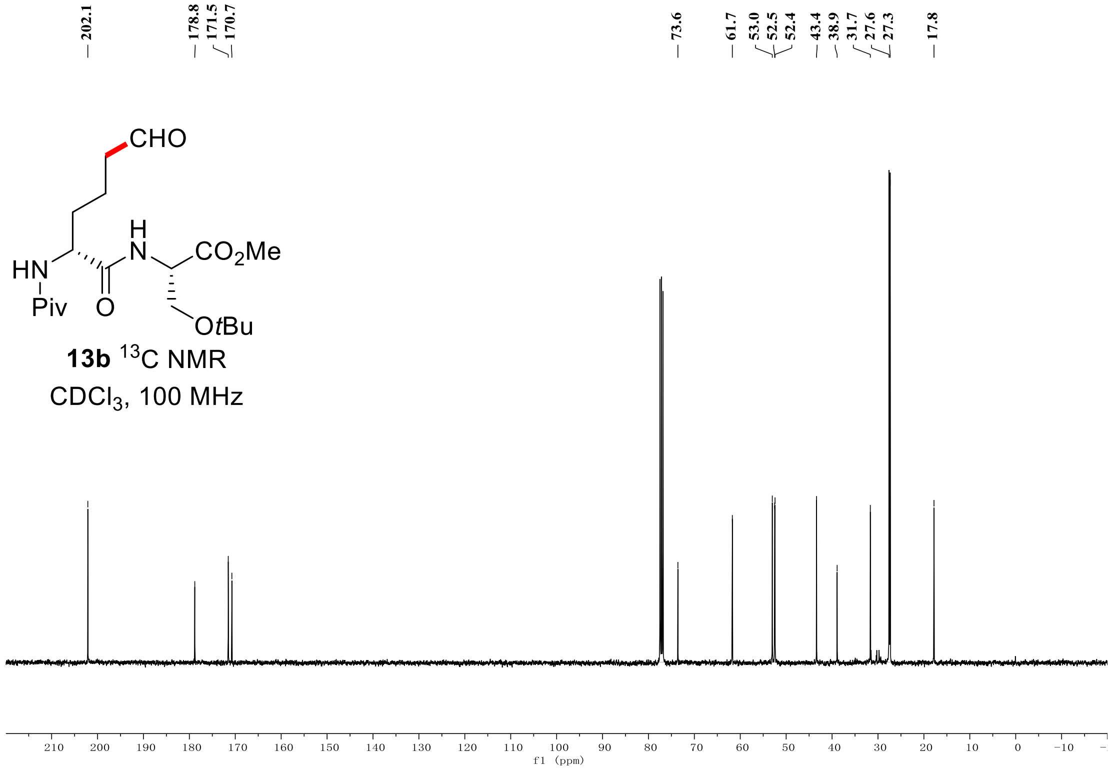
202.1
178.8
171.5
170.7
73.6
61.7
53.0
52.5
52.4
43.4
38.9
31.7
27.6
27.3
17.8
CHO
HN
Piv
O
H
N
CO_2Me
OtBu
13b ¹³C NMR
$CDCl_3$, 100 MHz
f1 (ppm)

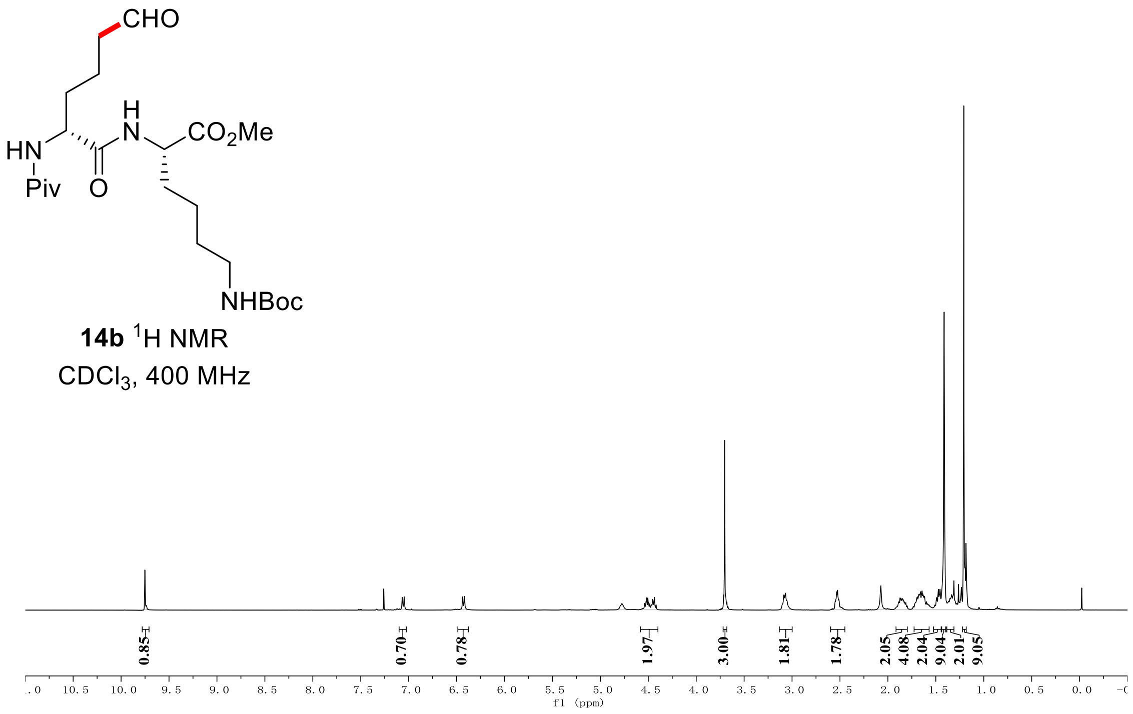

14b ^{1}H NMR
$CDCl_3$, 400 MHz

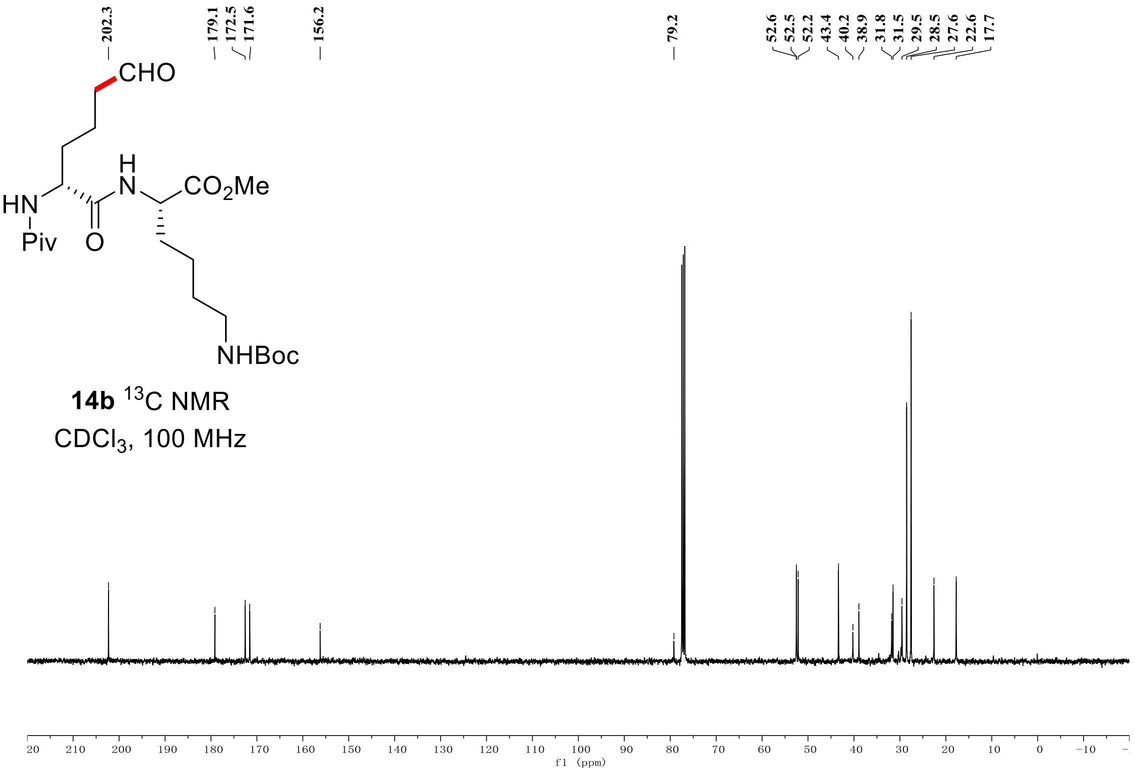

14b ^{13}C NMR
$CDCl_3$, 100 MHz

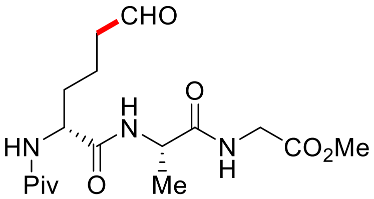

15b ^{1}H NMR

$CDCl_3$, 400 MHz

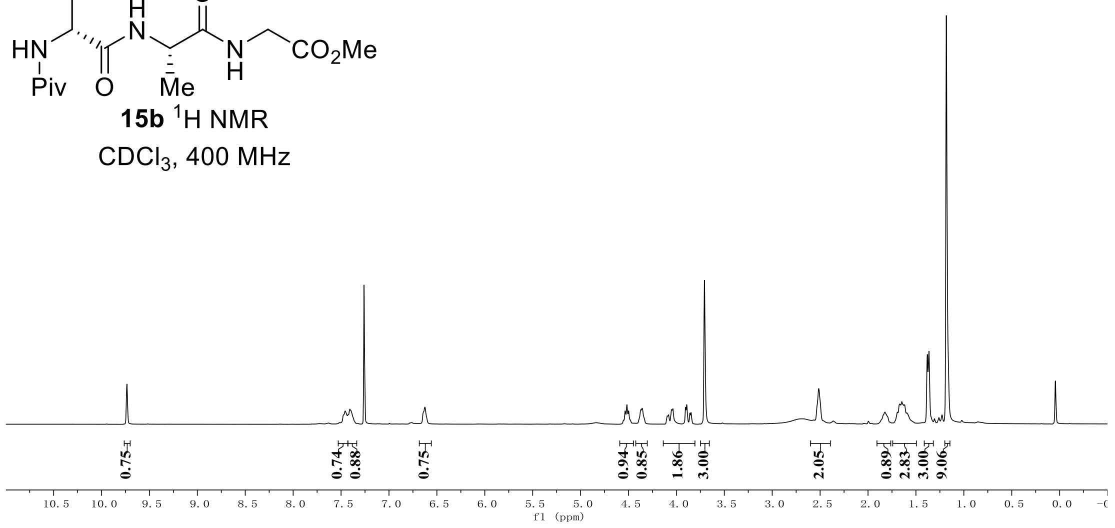

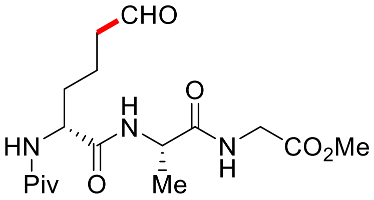

15b ^{13}C NMR

$CDCl_3$, 100 MHz

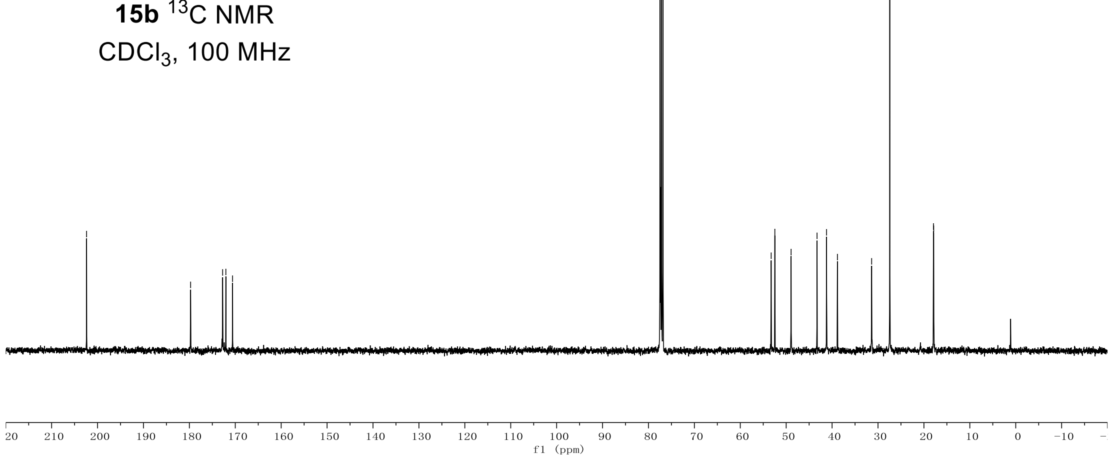

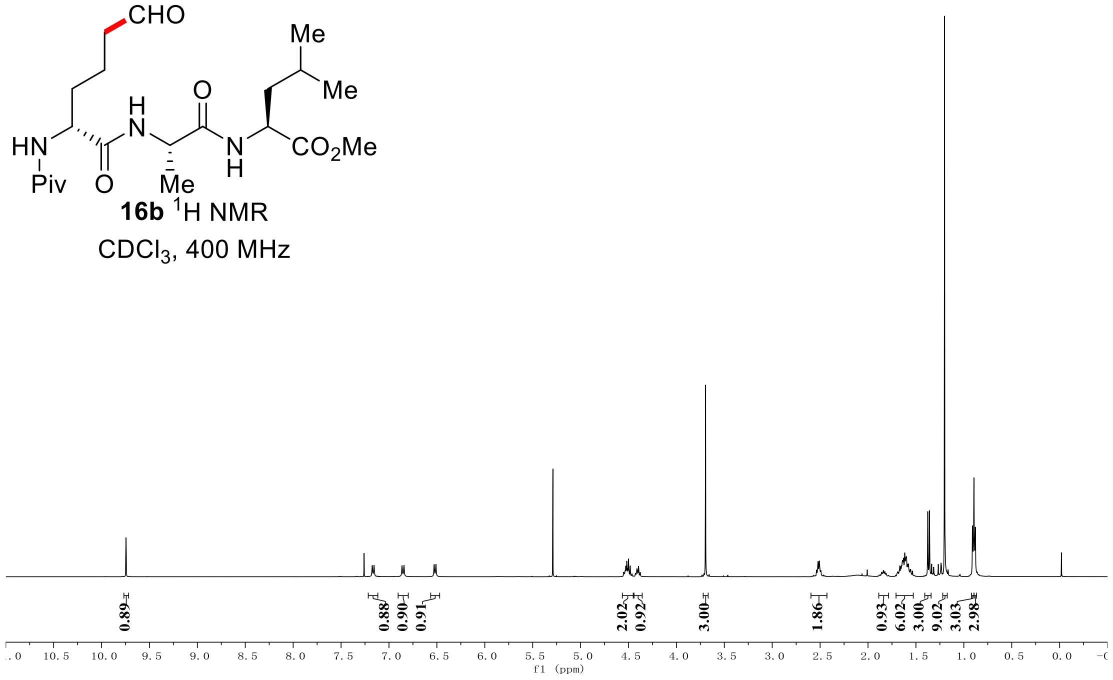

16b ^{1}H NMR

$CDCl_3$, 400 MHz

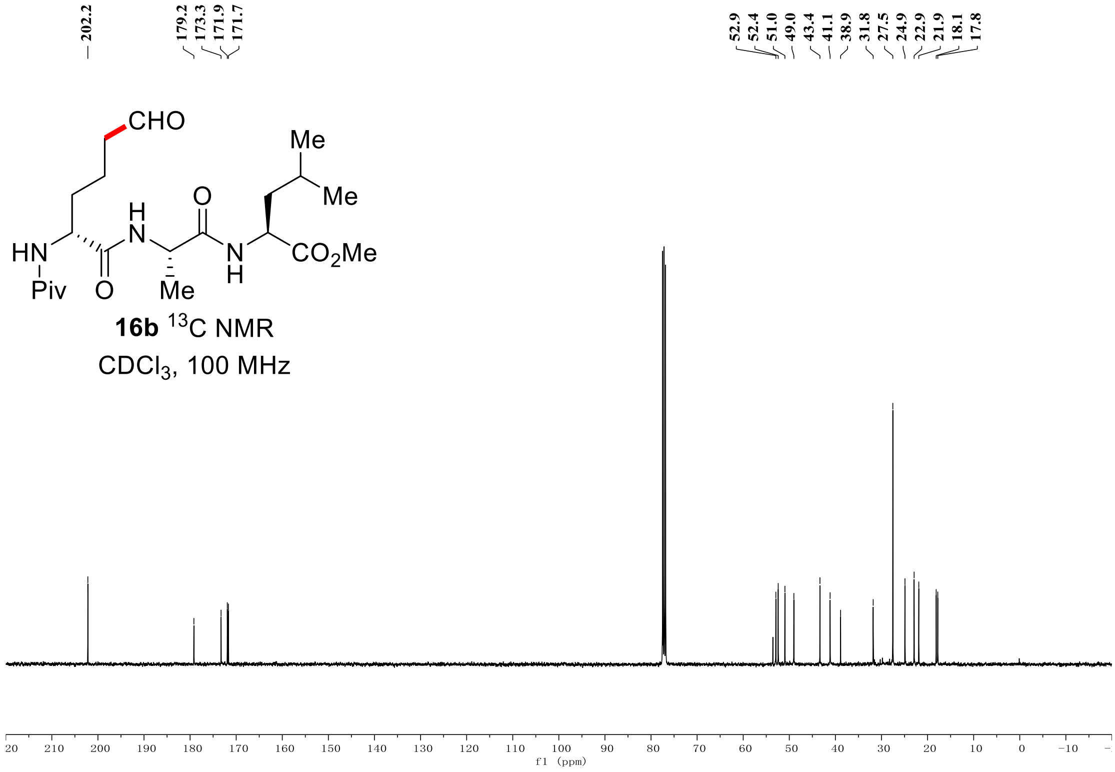

16b ^{13}C NMR

$CDCl_3$, 100 MHz

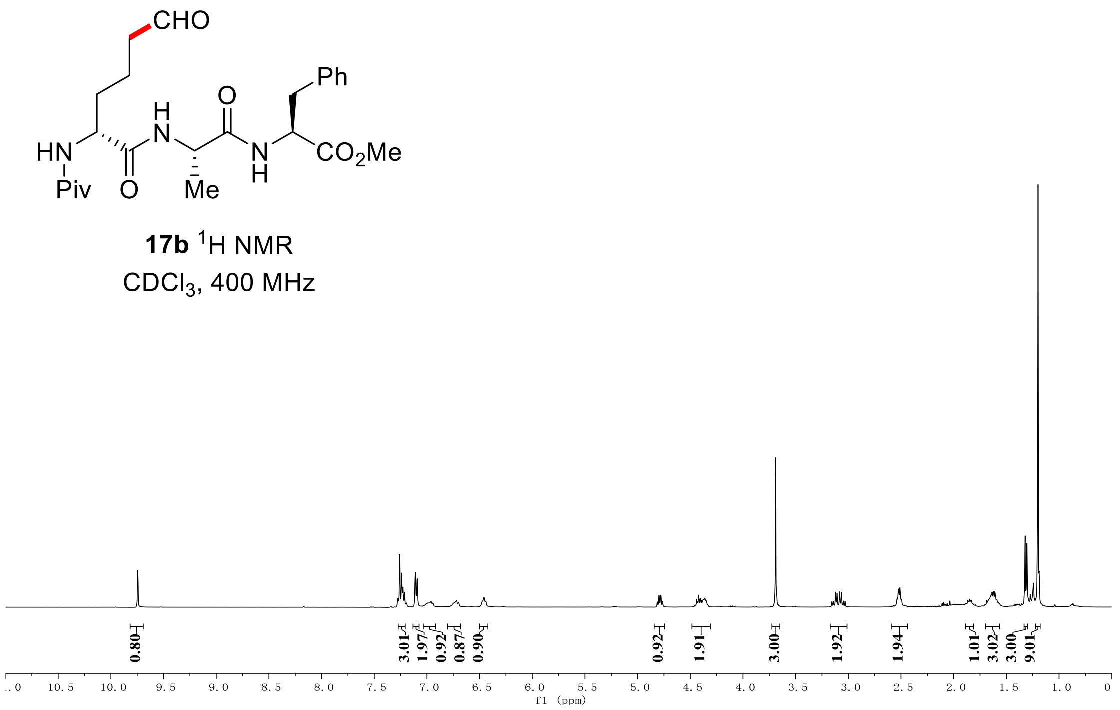
CHO
Ph
HN
Piv
O
H
N
Me
O
N
H
CO2Me
17b 1H NMR
CDCl3, 400 MHz
0.80
3.01
1.97
0.92
0.87
0.90
0.92
1.91
3.00
1.92
1.94
1.01
3.02
3.00
9.01
10.5
10.0
9.5
9.0
8.5
8.0
7.5
7.0
6.5
6.0
5.5
5.0
4.5
4.0
3.5
3.0
2.5
2.0
1.5
1.0
0
f1 (ppm)

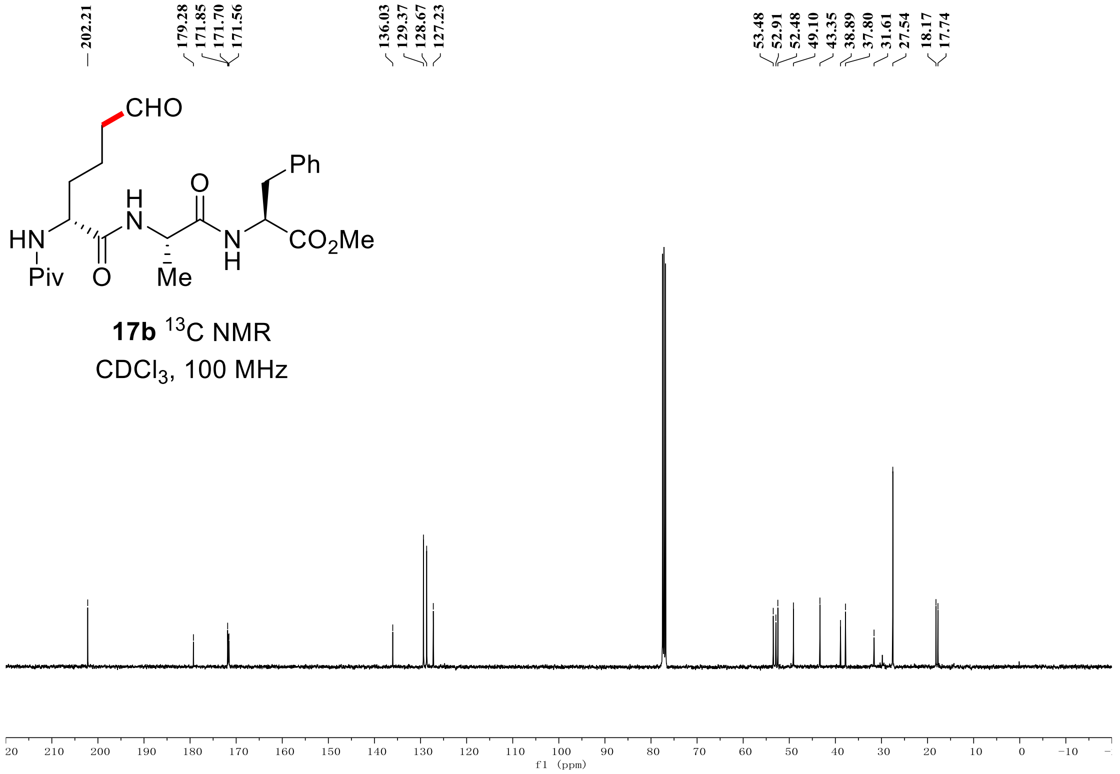
202.21
179.28
171.85
171.70
171.56
136.03
129.37
128.67
127.23
53.48
52.91
52.48
49.10
43.35
38.89
37.80
31.61
27.54
18.17
17.74
CHO
Ph
HN
Piv
O
H
N
Me
O
N
H
CO2Me
17b 13C NMR
CDCl3, 100 MHz
210
200
190
180
170
160
150
140
130
120
110
100
90
80
70
60
50
40
30
20
10
0
-10
f1 (ppm)

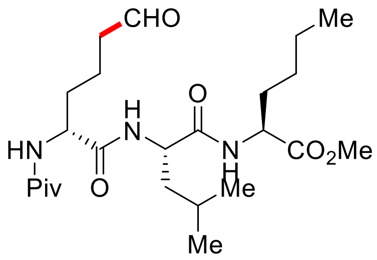

18b ^{1}H NMR

$CDCl_3$, 400 MHz

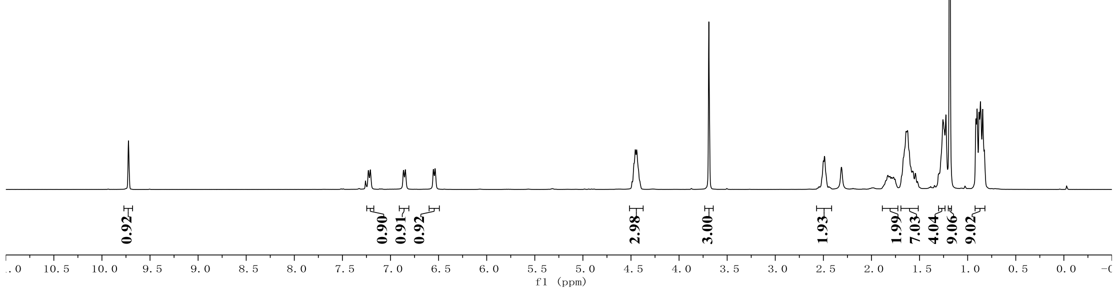

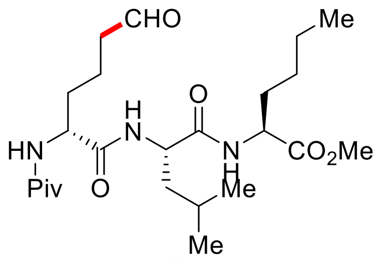

18b ^{13}C NMR

$CDCl_3$, 100 MHz

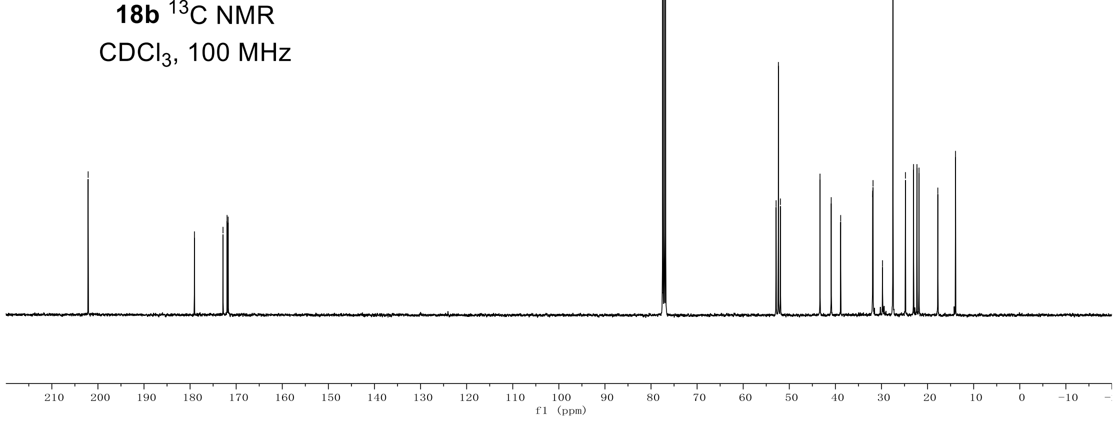

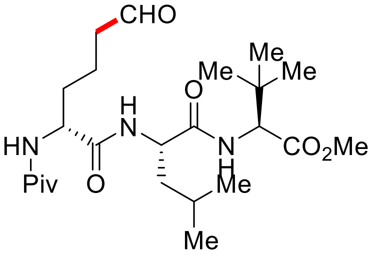

19b ^{1}H NMR
$CDCl_3$, 400 MHz

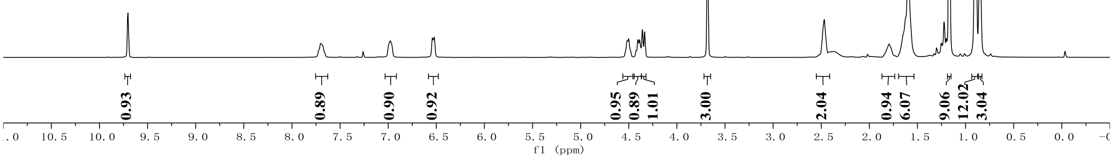

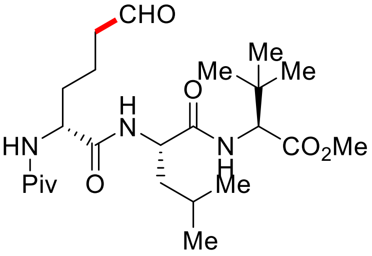

19b ^{13}C NMR
$CDCl_3$, 100 MHz

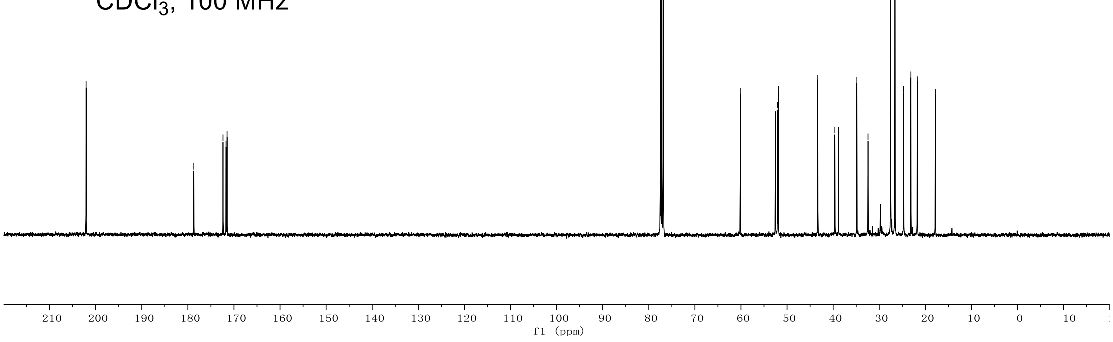

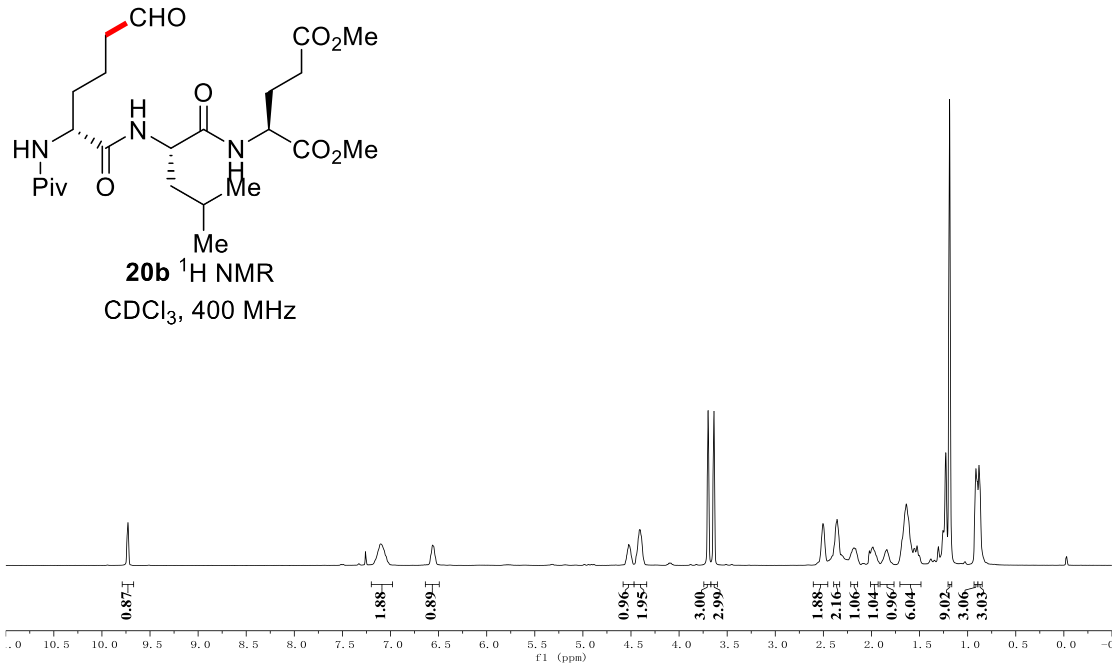

CHO
CO2Me
HN
Piv
O
H
N
O
N
H
CO2Me
Me
Me
20b 1H NMR
CDCl3, 400 MHz
0.87
1.88
0.89
0.96
1.95
3.00
2.99
1.88
2.16
1.06
1.04
0.96
6.04
9.02
3.06
3.03
f1 (ppm)

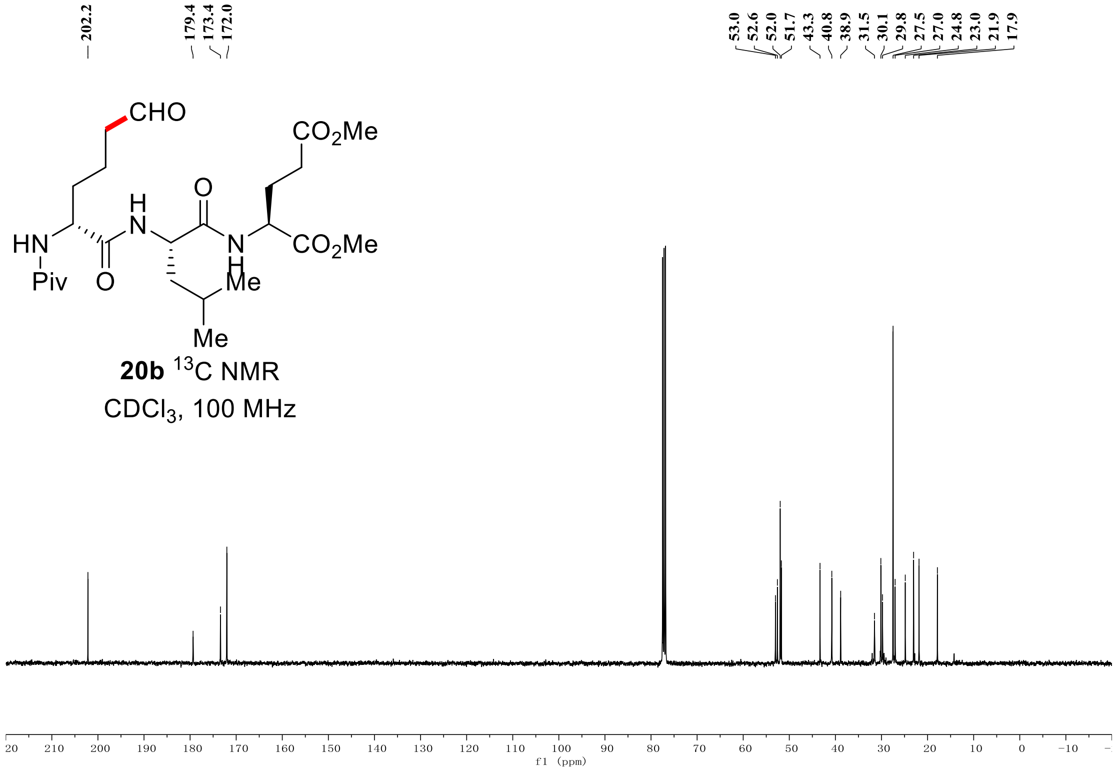

202.2
179.4
173.4
172.0
53.0
52.6
52.0
51.7
43.3
40.8
38.9
31.5
30.1
29.8
27.5
27.0
24.8
23.0
21.9
17.9
CHO
CO2Me
HN
Piv
O
H
N
O
N
H
CO2Me
Me
Me
20b 13C NMR
CDCl3, 100 MHz
f1 (ppm)

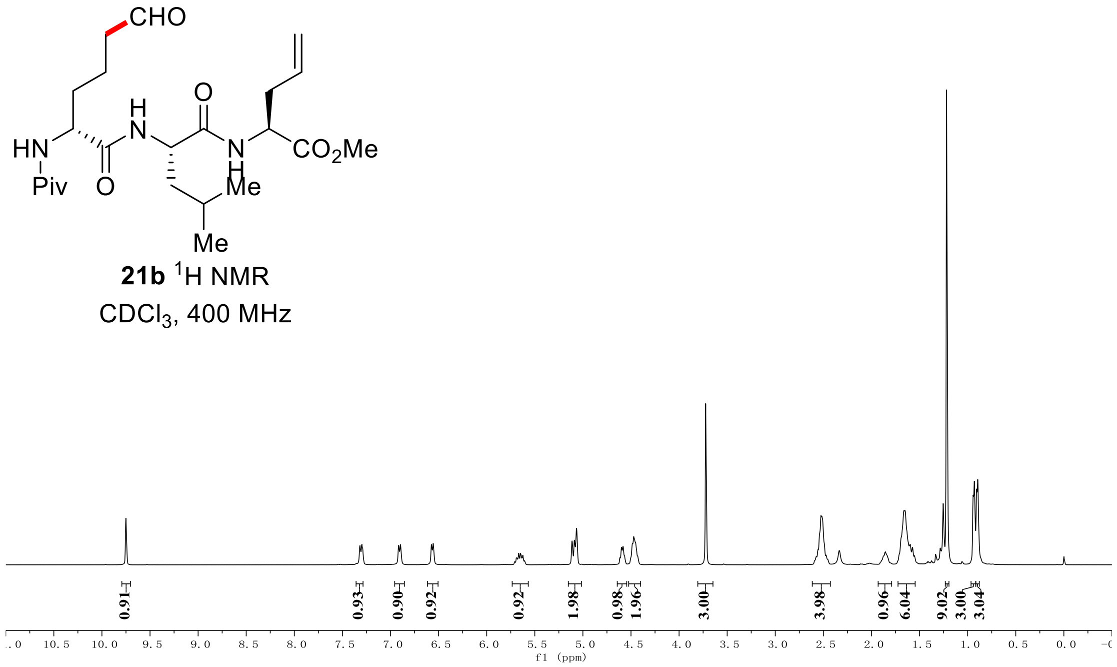

21b ^{1}H NMR

$CDCl_3$, 400 MHz

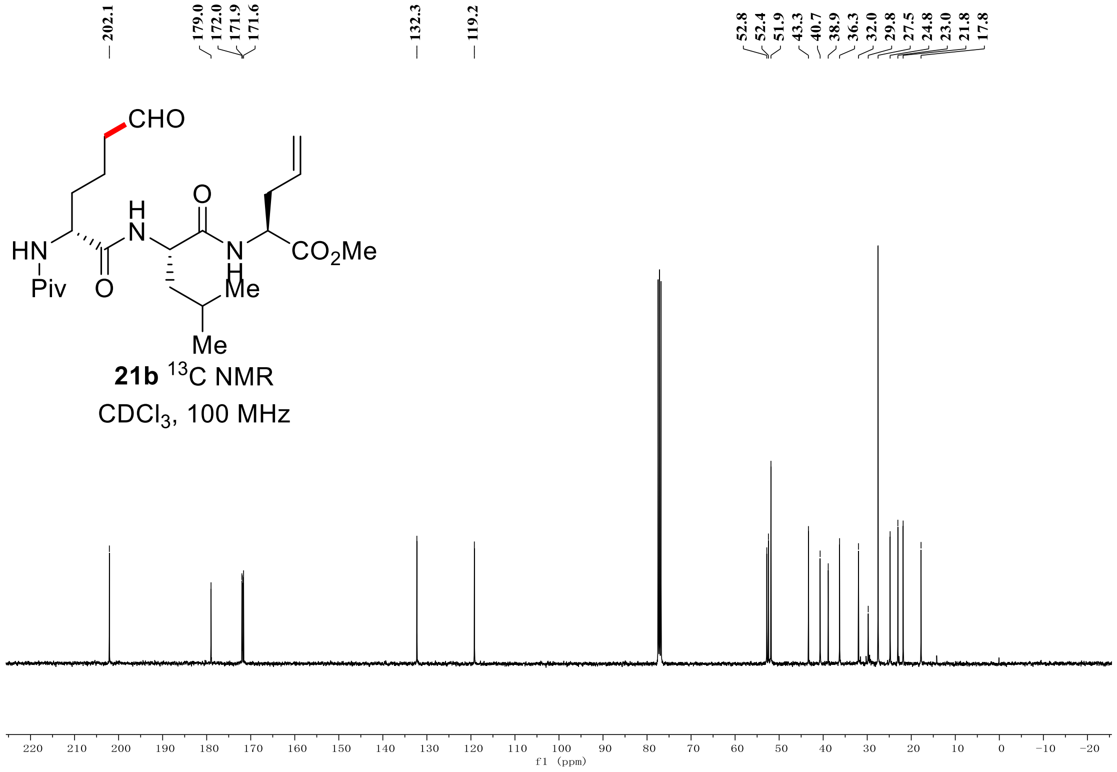

21b ^{13}C NMR

$CDCl_3$, 100 MHz

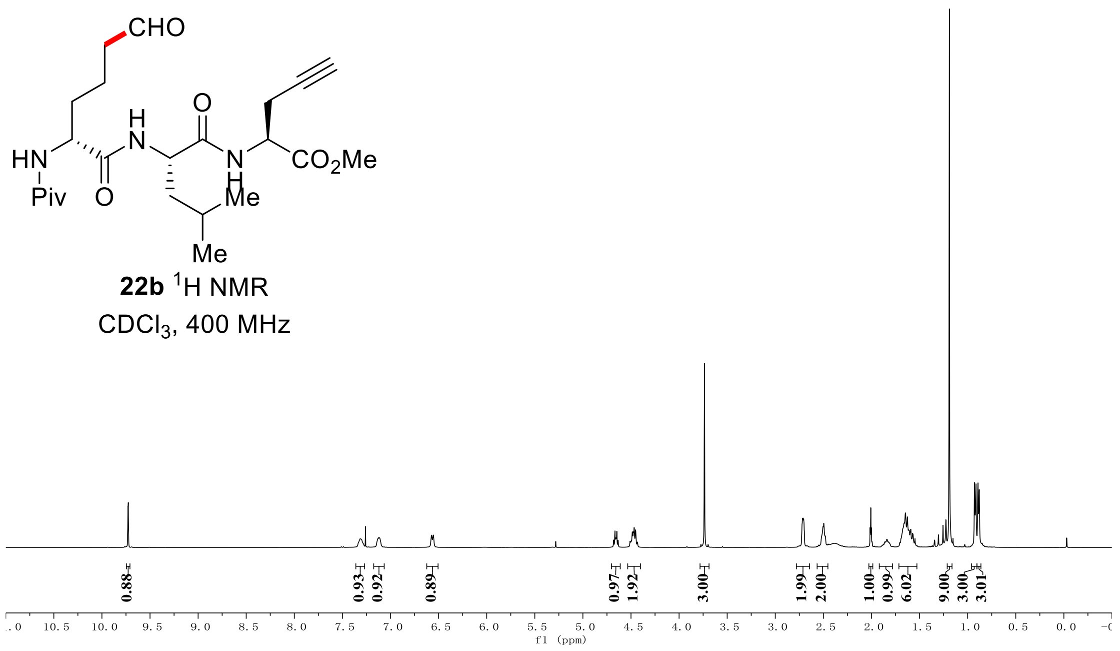
CHO
HN
Piv
O
H
N
O
N
H
CO2Me
Me
Me
22b 1H NMR
CDCl3, 400 MHz
0.88
0.93
0.92
0.89
0.97
1.92
3.00
1.99
2.00
1.00
0.99
6.02
9.00
3.00
3.01
10.5 10.0 9.5 9.0 8.5 8.0 7.5 7.0 6.5 6.0 5.5 5.0 4.5 4.0 3.5 3.0 2.5 2.0 1.5 1.0 0.5 0.0
f1 (ppm)

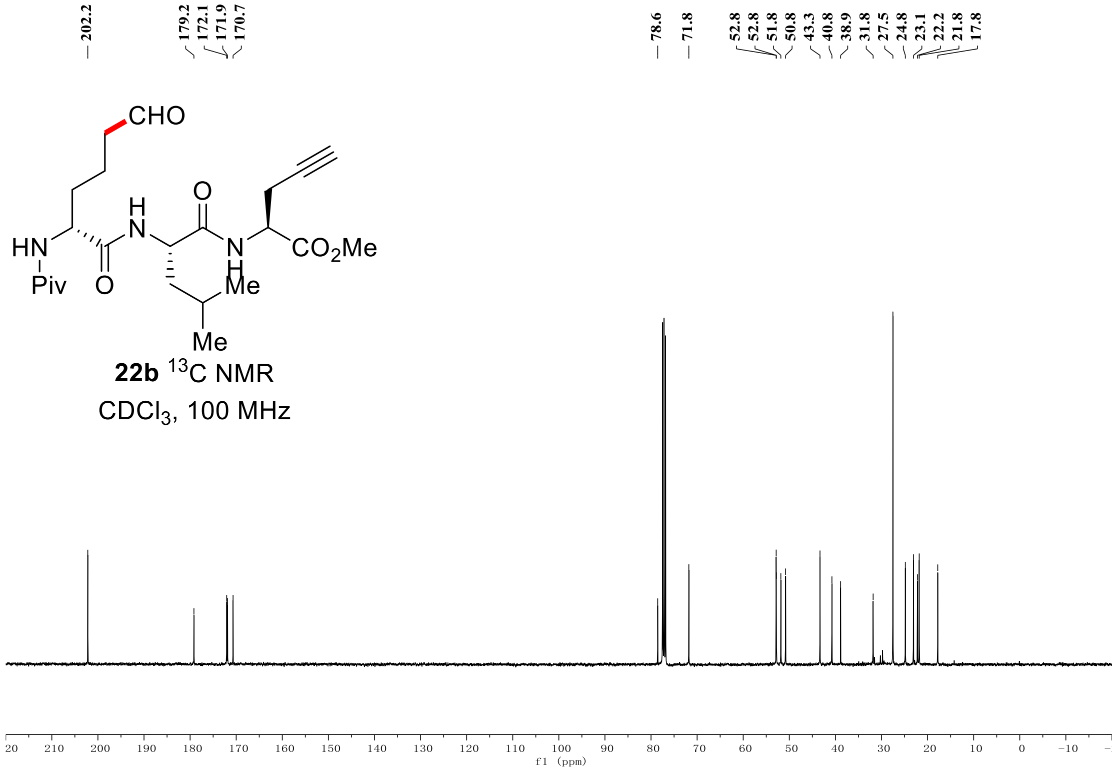
202.2
179.2
172.1
171.9
170.7
78.6
71.8
52.8
52.8
51.8
50.8
43.3
40.8
38.9
31.8
27.5
24.8
23.1
22.2
21.8
17.8
CHO
HN
Piv
O
H
N
O
N
H
CO2Me
Me
Me
22b 13C NMR
CDCl3, 100 MHz
210 200 190 180 170 160 150 140 130 120 110 100 90 80 70 60 50 40 30 20 10 0 -10
f1 (ppm)

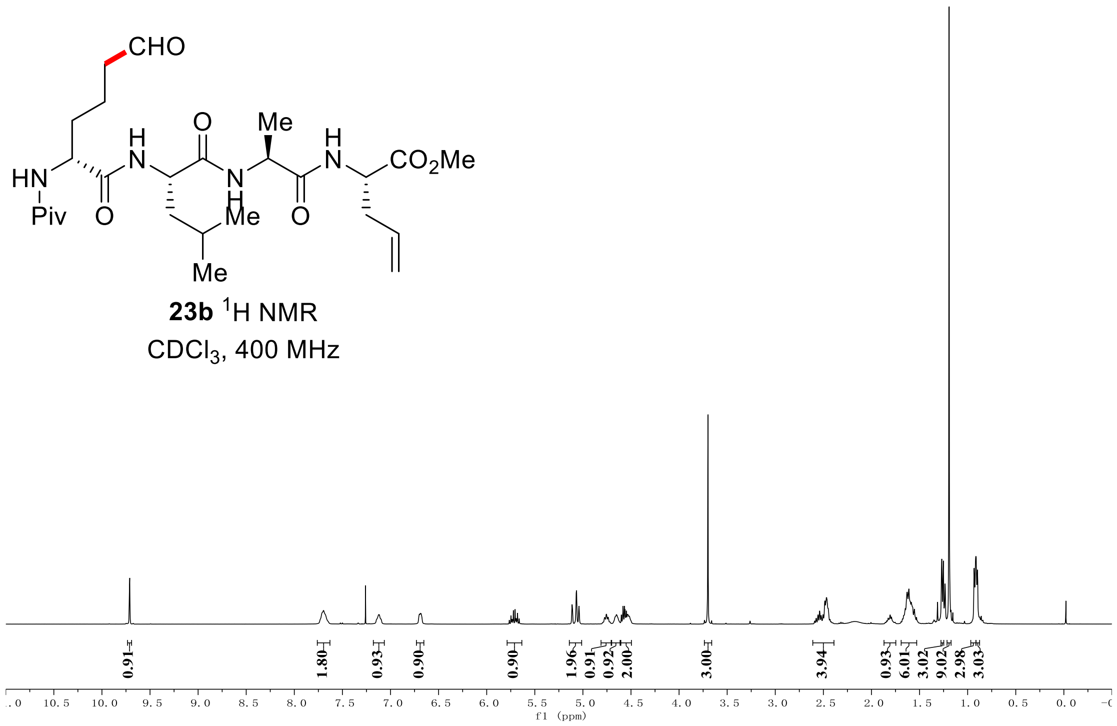
CHO
HN
Piv
Me
CO2Me
23b 1H NMR
CDCl3, 400 MHz
0.91
1.80
0.93
0.90
0.90
1.96
0.91
0.92
2.00
3.00
3.94
0.93
6.01
3.02
9.02
2.98
3.03
f1 (ppm)

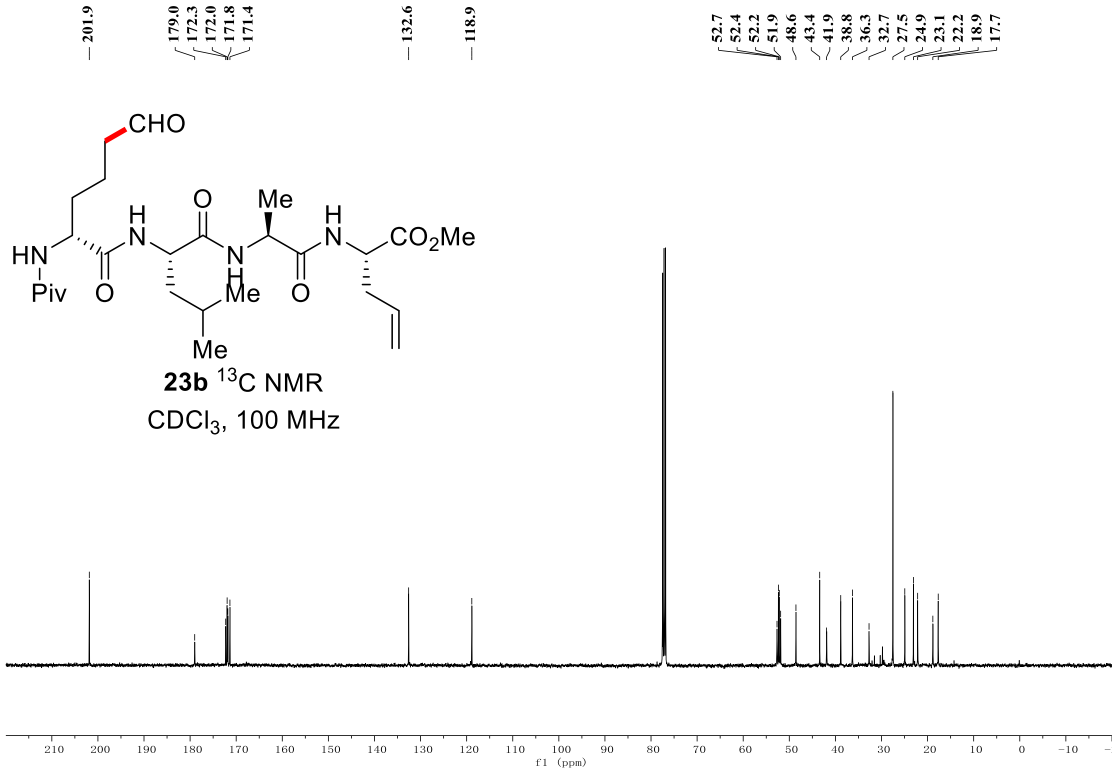
201.9
179.0
172.3
172.0
171.8
171.4
132.6
118.9
52.7
52.4
52.2
51.9
48.6
43.4
41.9
38.8
36.3
32.7
27.5
24.9
23.1
22.2
18.9
17.7
CHO
HN
Piv
Me
CO2Me
23b 13C NMR
CDCl3, 100 MHz
f1 (ppm)

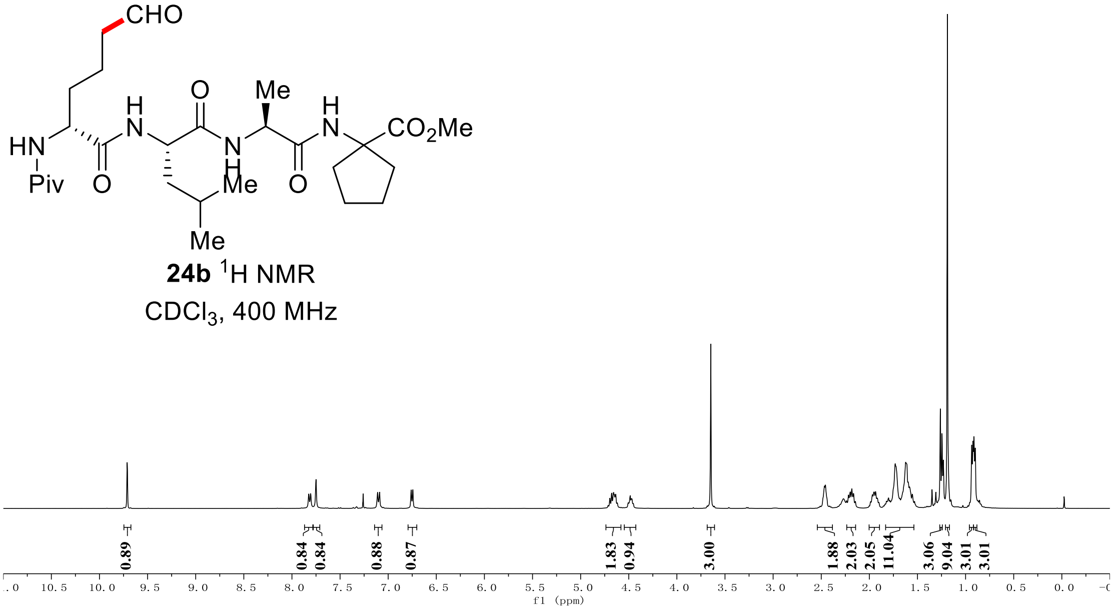
CHO
HN
Piv
O
H
N
O
Me
N
H
O
H
N
CO2Me
Me
Me
24b 1H NMR
CDCl3, 400 MHz
0.89
0.84
0.84
0.88
0.87
1.83
0.94
3.00
1.88
2.03
2.05
11.04
3.06
9.04
3.01
3.01
f1 (ppm)

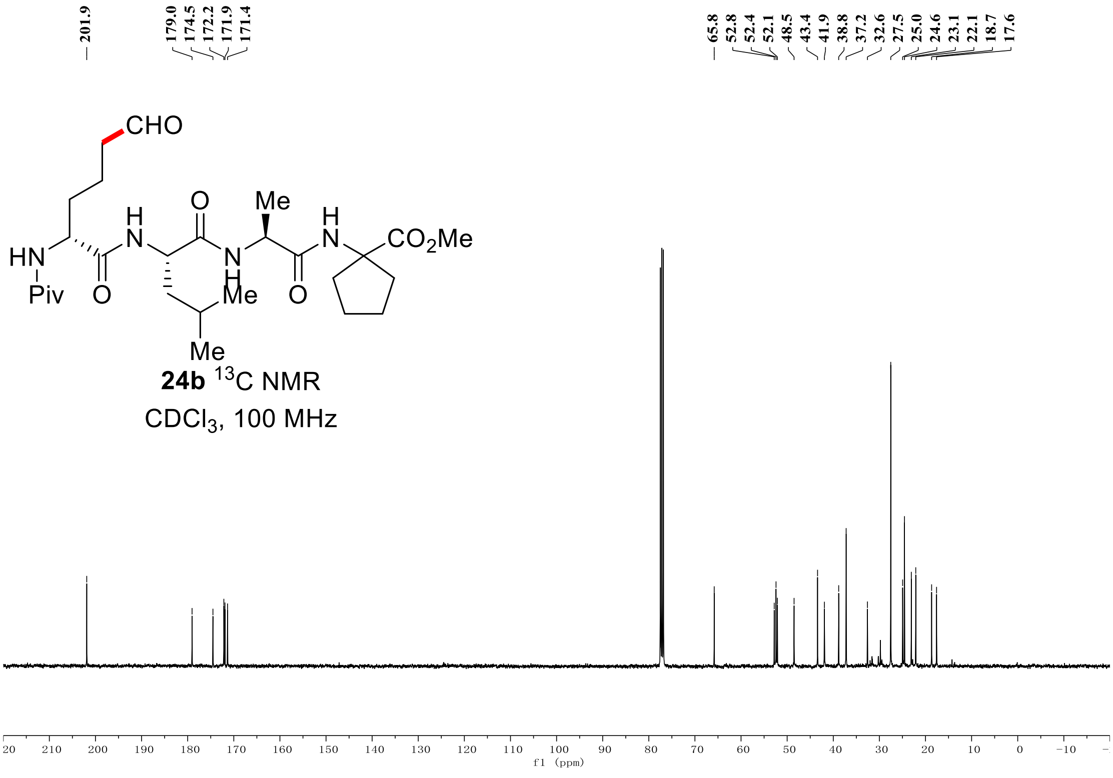
201.9
179.0
174.5
172.2
171.9
171.4
65.8
52.8
52.4
52.1
48.5
43.4
41.9
38.8
37.2
32.6
27.5
25.0
24.6
23.1
22.1
18.7
17.6
CHO
HN
Piv
O
H
N
O
Me
N
H
O
H
N
CO2Me
Me
Me
24b 13C NMR
CDCl3, 100 MHz
f1 (ppm)

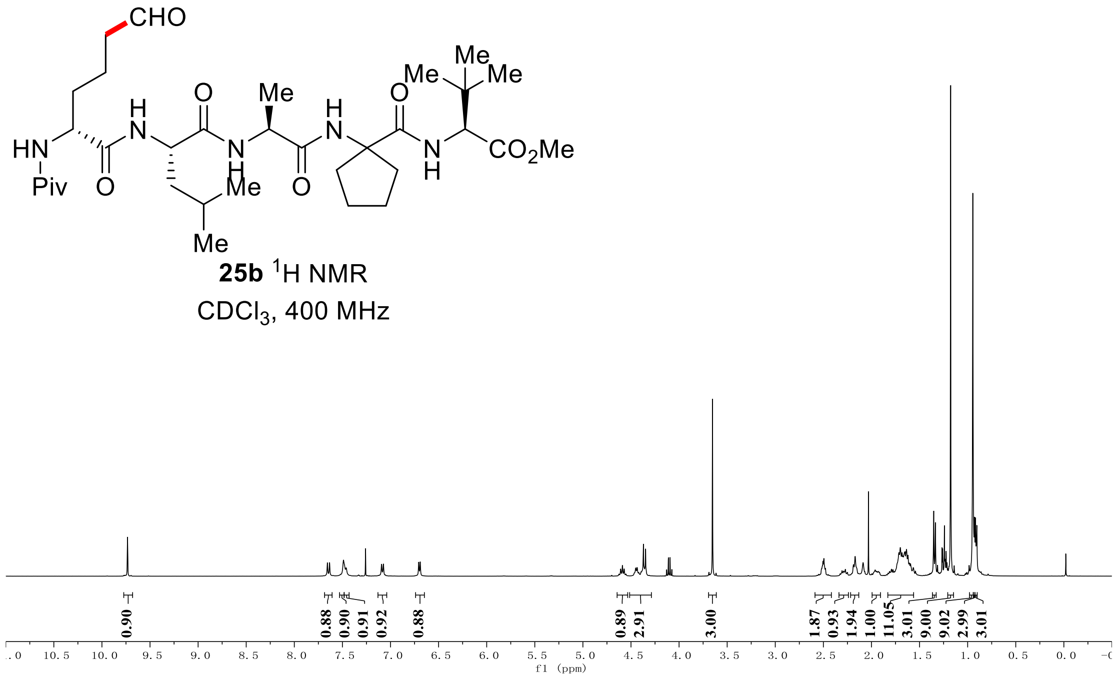
25b ^{1}H NMR
CDCl$_3$, 400 MHz
0.90 0.88 0.90 0.91 0.92 0.88 0.89 2.91 3.00 1.87 0.93 1.94 1.00 11.05 3.01 9.00 9.02 2.99 3.01
f1 (ppm)

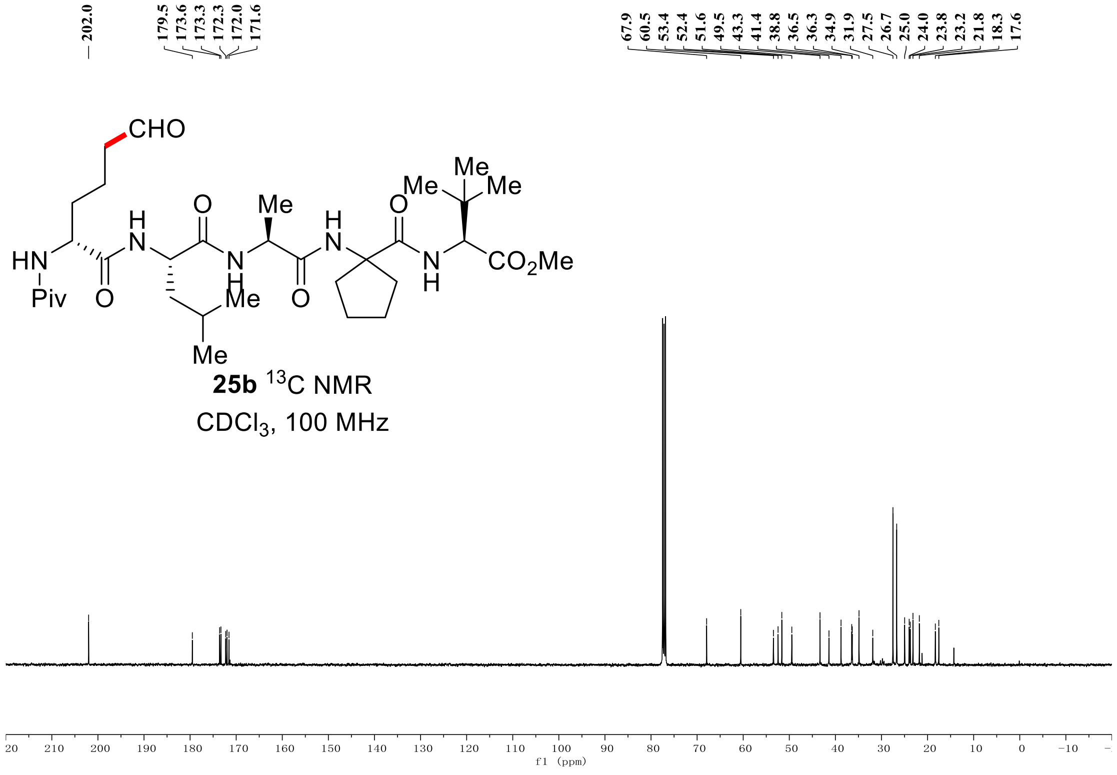
202.0
179.5 173.6 173.3 172.3 172.0 171.6
67.9 60.5 53.4 52.4 51.6 49.5 43.3 41.4 38.8 36.5 36.3 34.9 31.9 27.5 26.7 25.0 24.0 23.8 23.2 21.8 18.3 17.6
25b ^{13}C NMR
CDCl$_3$, 100 MHz
f1 (ppm)

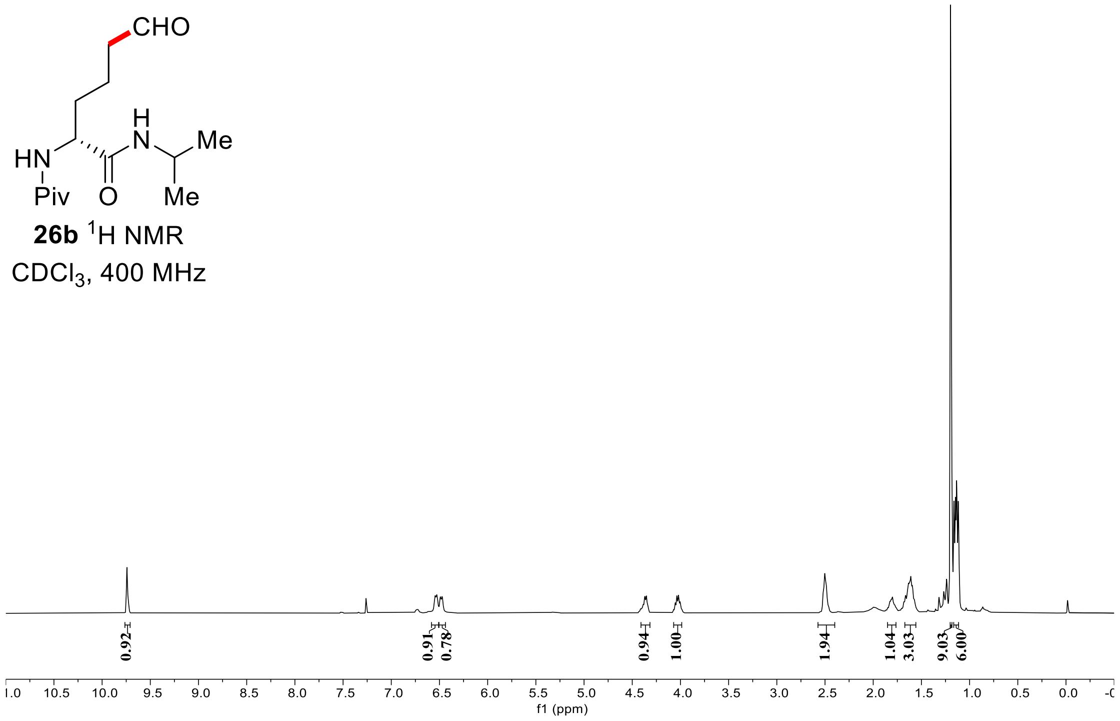
CHO
H
N
Me
HN
Piv
O
Me
26b ^{1}H NMR
$CDCl_3$, 400 MHz
0.92
0.91
0.78
0.94
1.00
1.94
1.04
3.03
9.03
6.00
f1 (ppm)

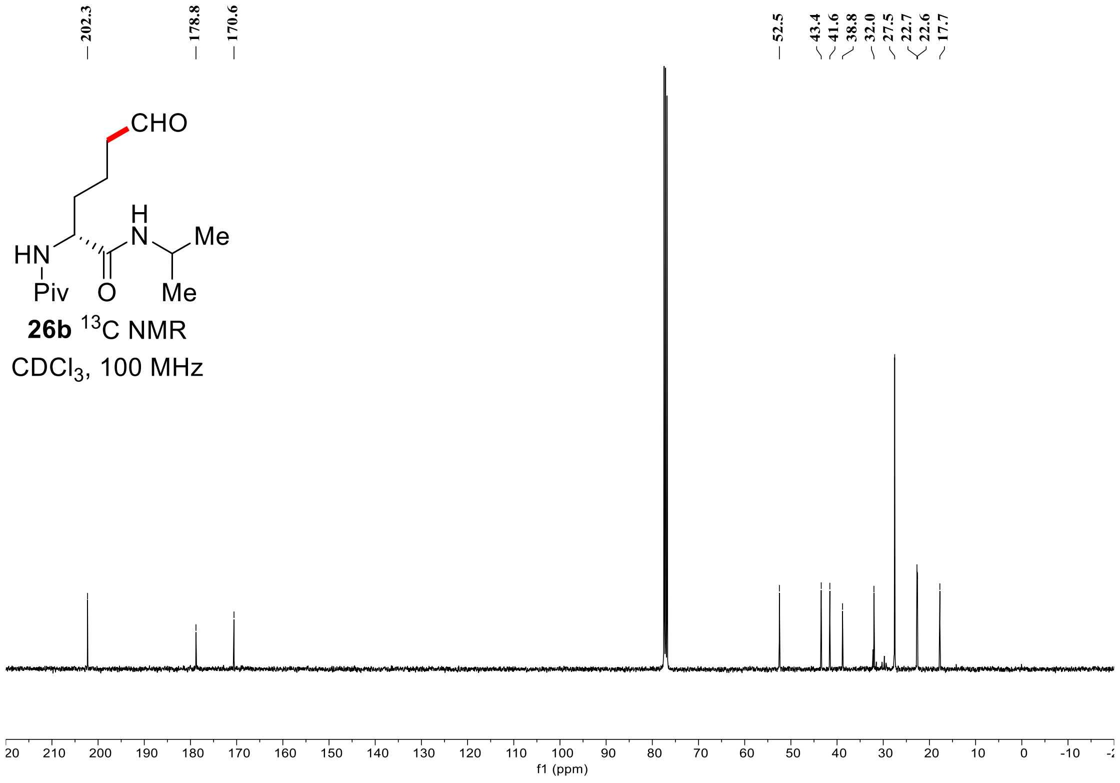
202.3
178.8
170.6
52.5
43.4
41.6
38.8
32.0
27.5
22.7
22.6
17.7
CHO
H
N
Me
HN
Piv
O
Me
26b ^{13}C NMR
$CDCl_3$, 100 MHz
f1 (ppm)

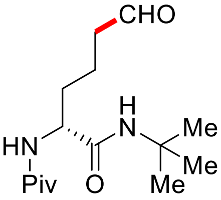

27b ^{1}H NMR
CDCl$_3$, 400 MHz

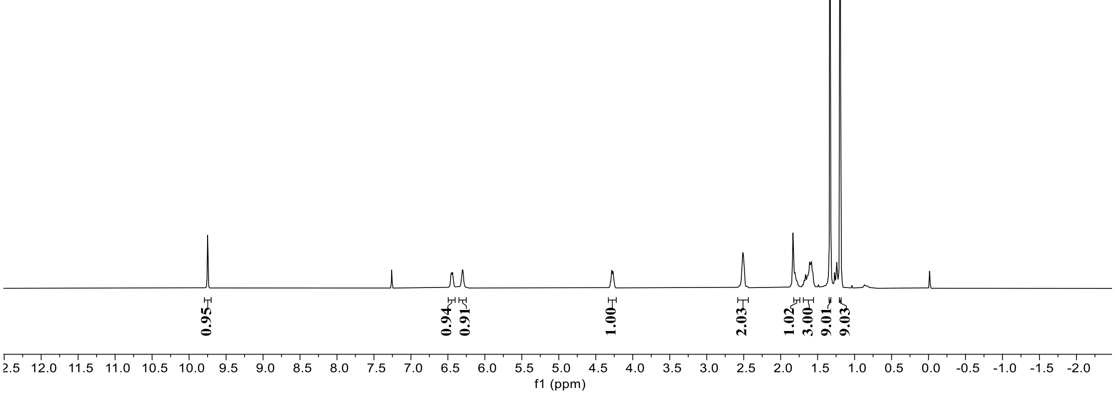

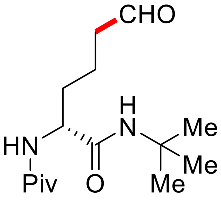

27b ^{13}C NMR
CDCl$_3$, 100 MHz

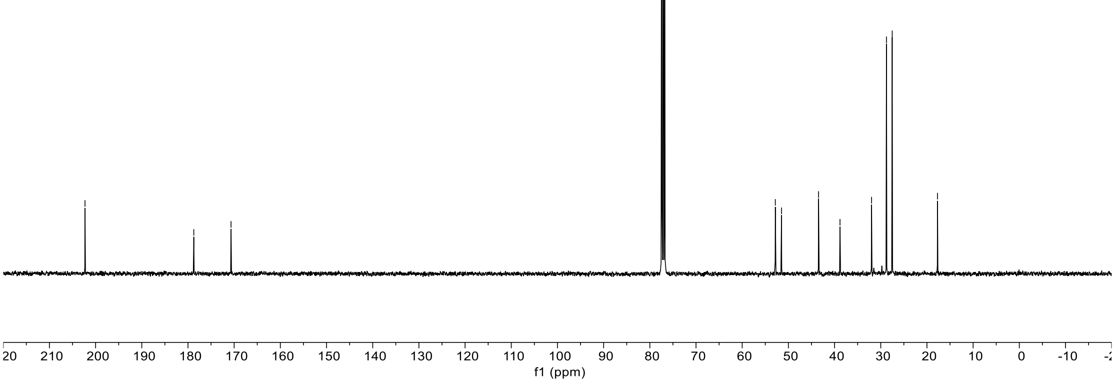

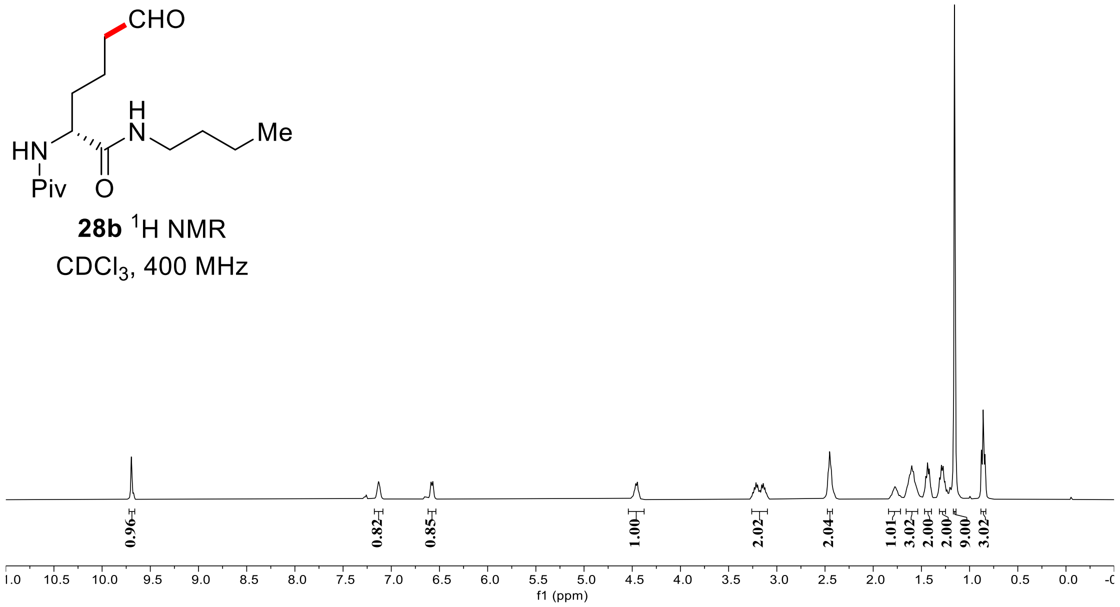
CHO
HN
Piv
H
N
O
Me
28b ^{1}H NMR
$CDCl_3$, 400 MHz
0.96
0.82
0.85
1.00
2.02
2.04
1.01
3.02
2.00
2.00
9.00
3.02
f1 (ppm)

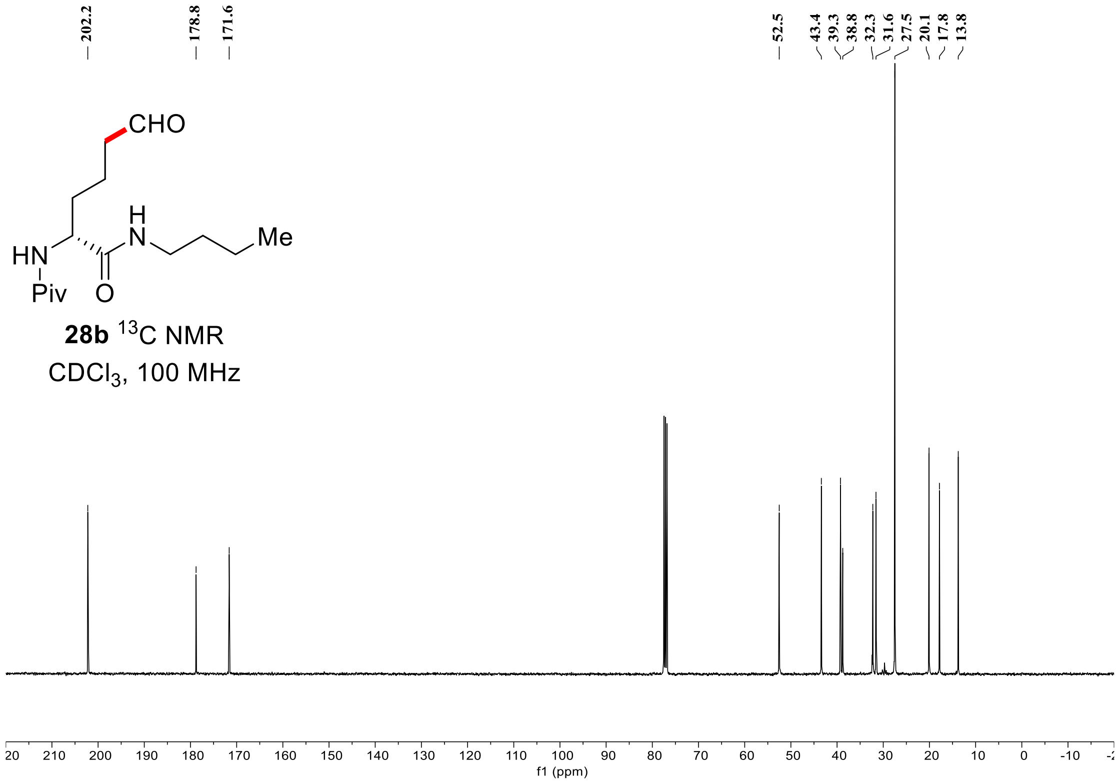
202.2
178.8
171.6
52.5
43.4
39.3
38.8
32.3
31.6
27.5
20.1
17.8
13.8
CHO
HN
Piv
H
N
O
Me
28b ^{13}C NMR
$CDCl_3$, 100 MHz
f1 (ppm)

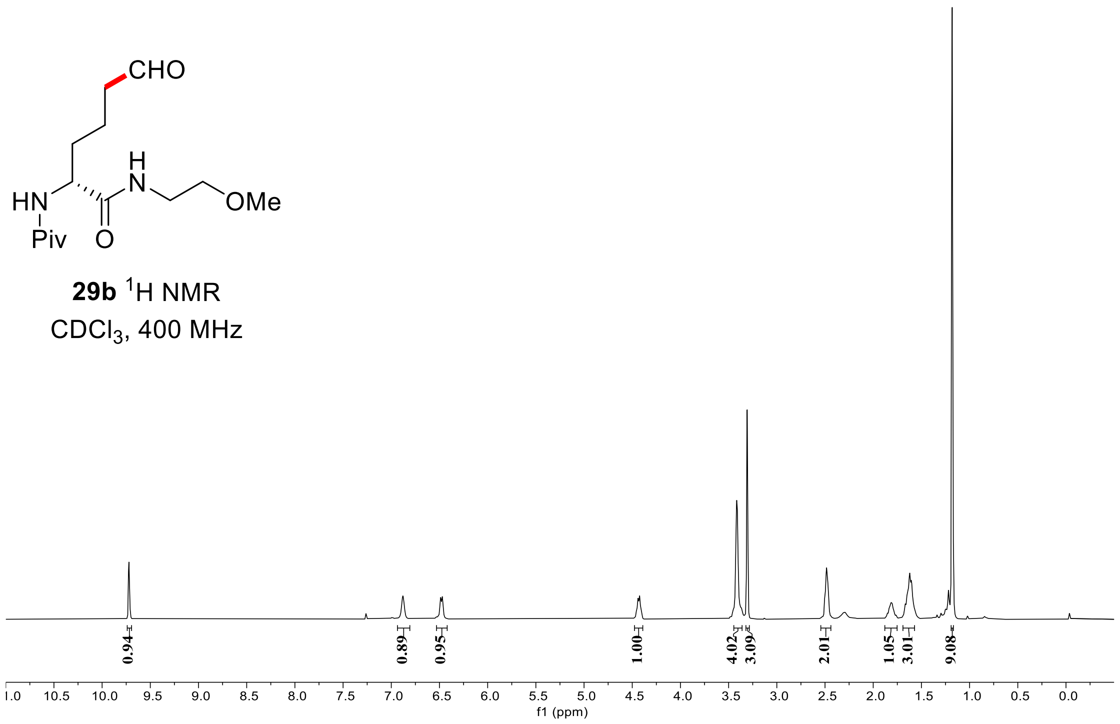
CHO
HN
Piv
H
N
O
OMe
29b ¹H NMR
CDCl3, 400 MHz
0.94
0.89
0.95
1.00
4.02
3.09
2.01
1.05
3.01
9.08
f1 (ppm)

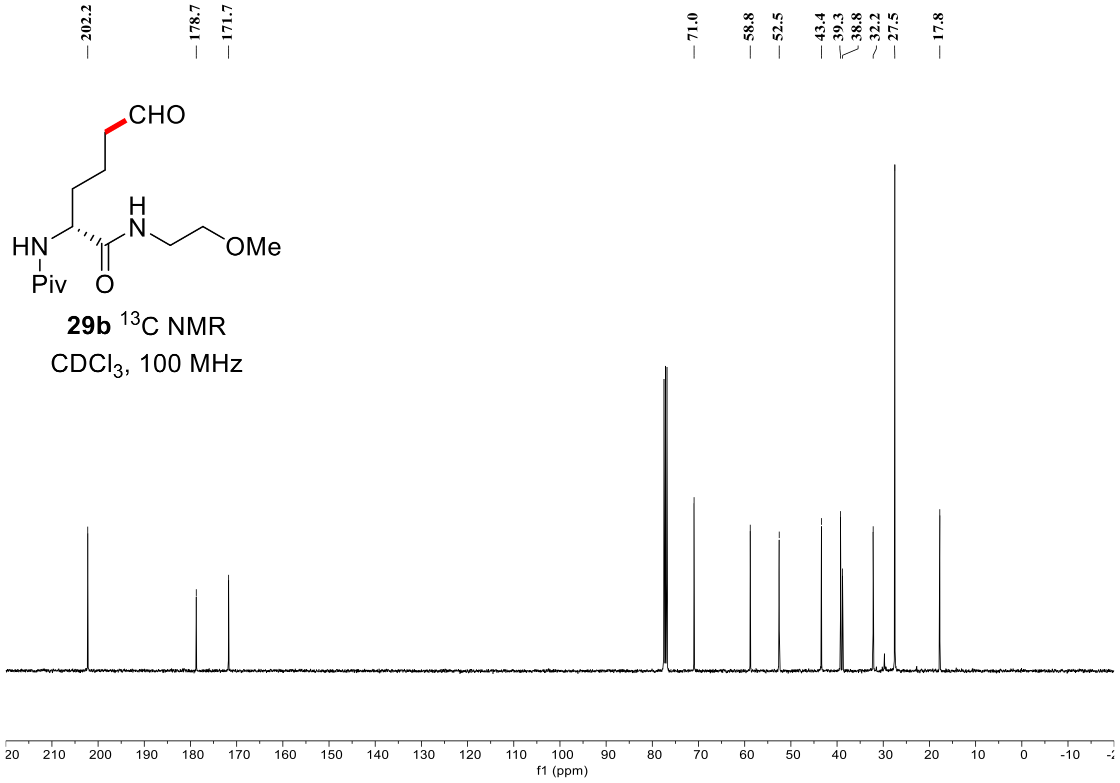
202.2
178.7
171.7
71.0
58.8
52.5
43.4
39.3
38.8
32.2
27.5
17.8
CHO
HN
Piv
H
N
O
OMe
29b ¹³C NMR
CDCl3, 100 MHz
f1 (ppm)

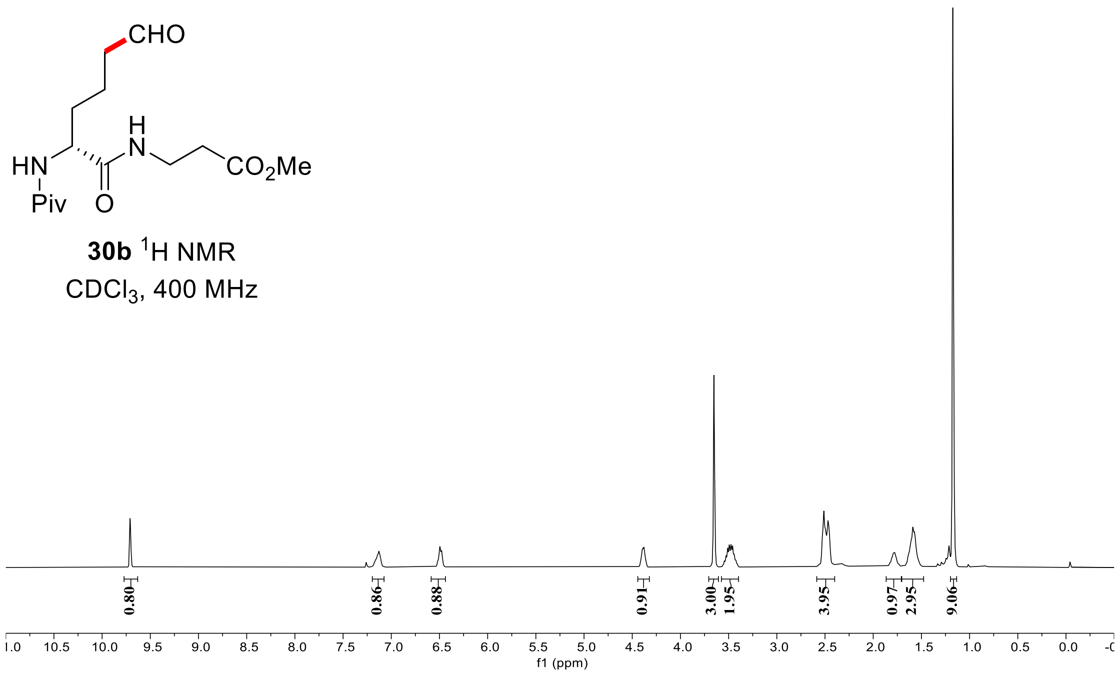
CHO
H
N
HN
CO2Me
Piv
O
30b 1H NMR
CDCl3, 400 MHz
0.80
0.86
0.88
0.91
3.00
1.95
3.95
0.97
2.95
9.06
f1 (ppm)

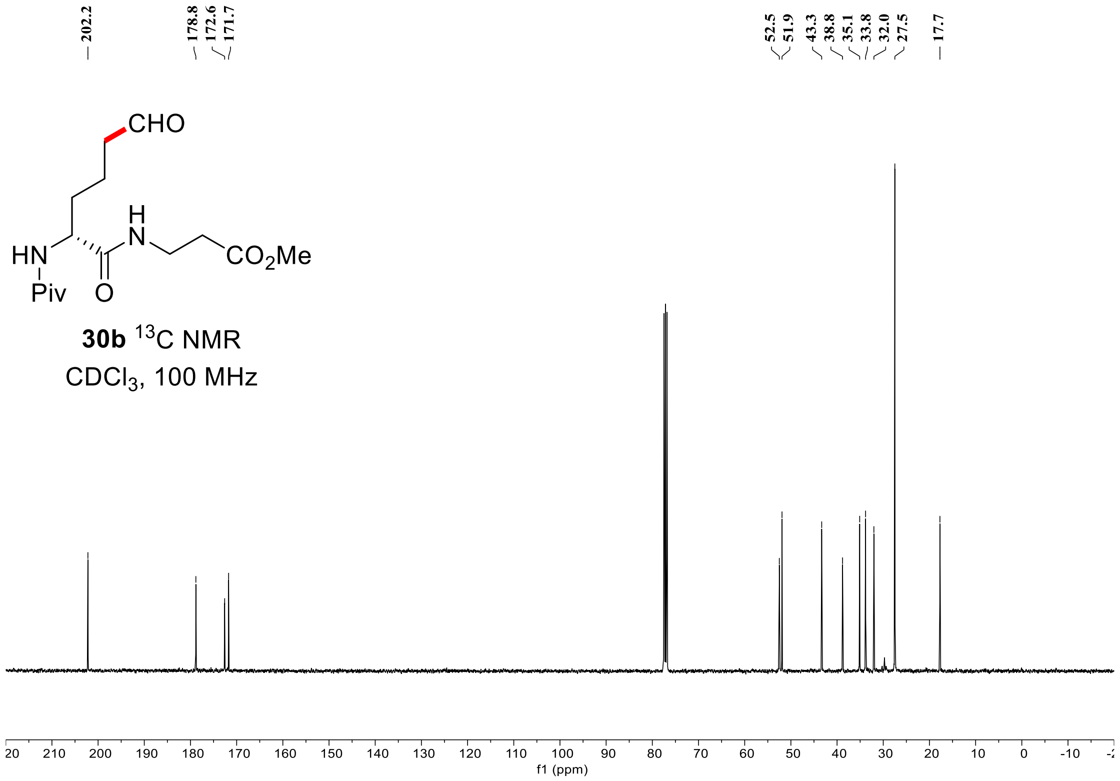
202.2
178.8
172.6
171.7
52.5
51.9
43.3
38.8
35.1
33.8
32.0
27.5
17.7
CHO
H
N
HN
CO2Me
Piv
O
30b 13C NMR
CDCl3, 100 MHz
f1 (ppm)

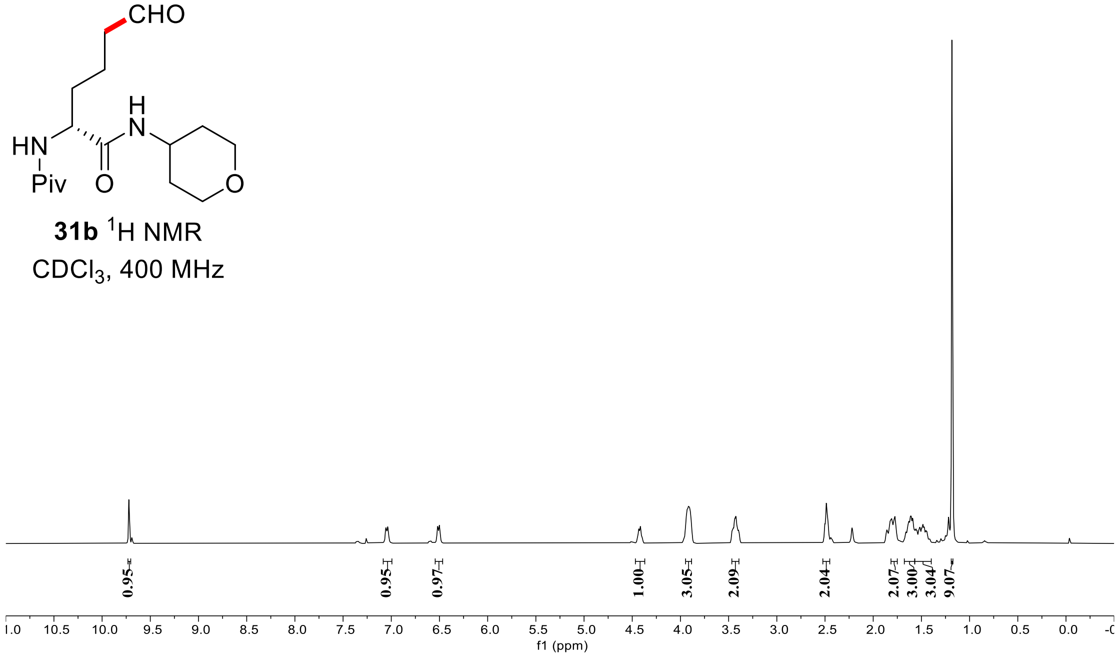
CHO
H
N
HN
Piv
O
O
31b ¹H NMR
CDCl3, 400 MHz
0.95
0.95
0.97
1.00
3.05
2.09
2.04
2.07
3.00
3.04
9.07
f1 (ppm)

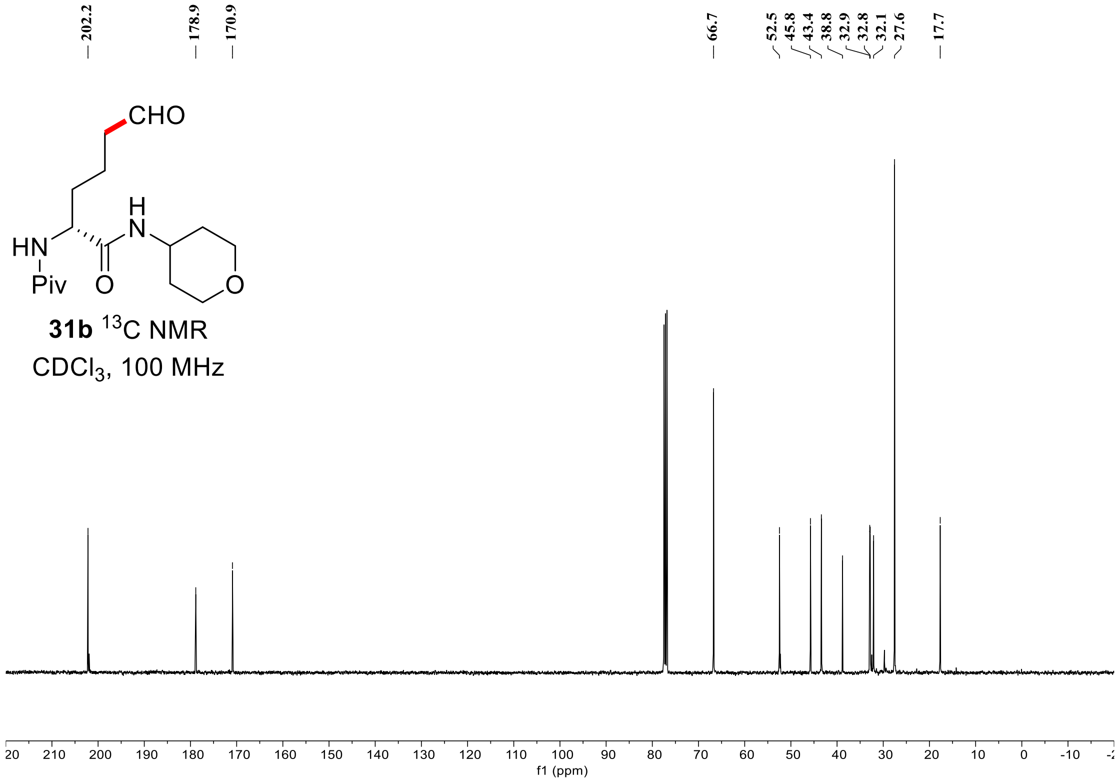
202.2
178.9
170.9
66.7
52.5
45.8
43.4
38.8
32.9
32.8
32.1
27.6
17.7
CHO
H
N
HN
Piv
O
O
31b ¹³C NMR
CDCl3, 100 MHz
f1 (ppm)

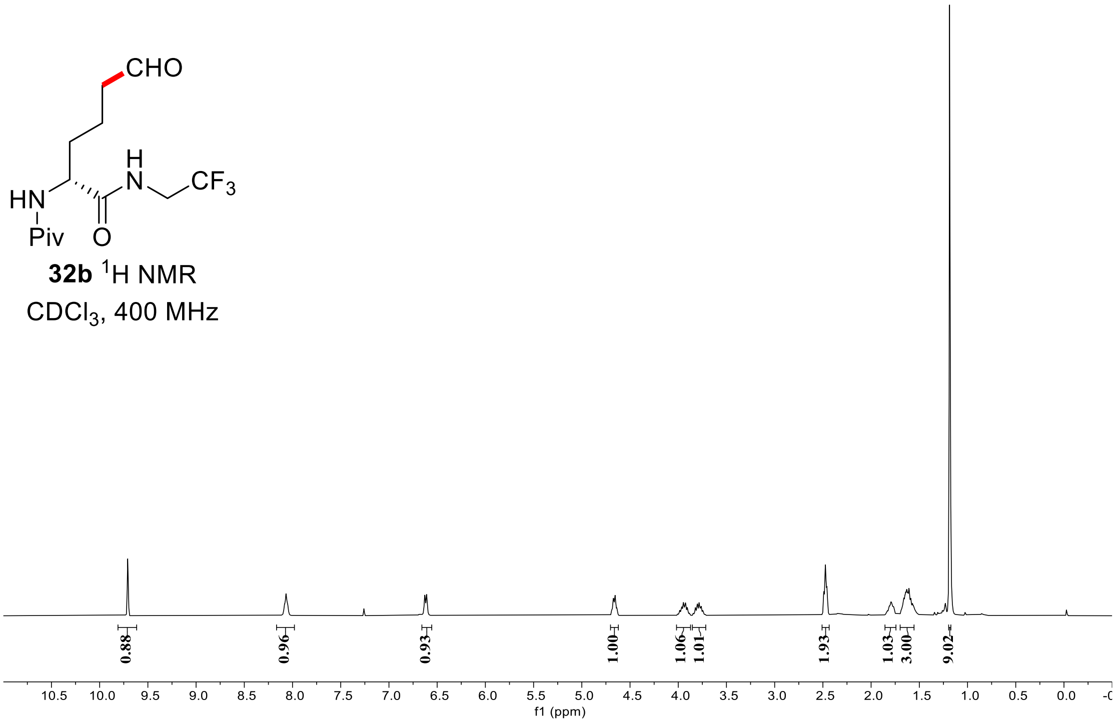
CHO
H
N
CF3
HN
Piv
O
32b 1H NMR
CDCl3, 400 MHz
0.88
0.96
0.93
1.00
1.06
1.01
1.93
1.03
3.00
9.02
10.5 10.0 9.5 9.0 8.5 8.0 7.5 7.0 6.5 6.0 5.5 5.0 4.5 4.0 3.5 3.0 2.5 2.0 1.5 1.0 0.5 0.0
f1 (ppm)

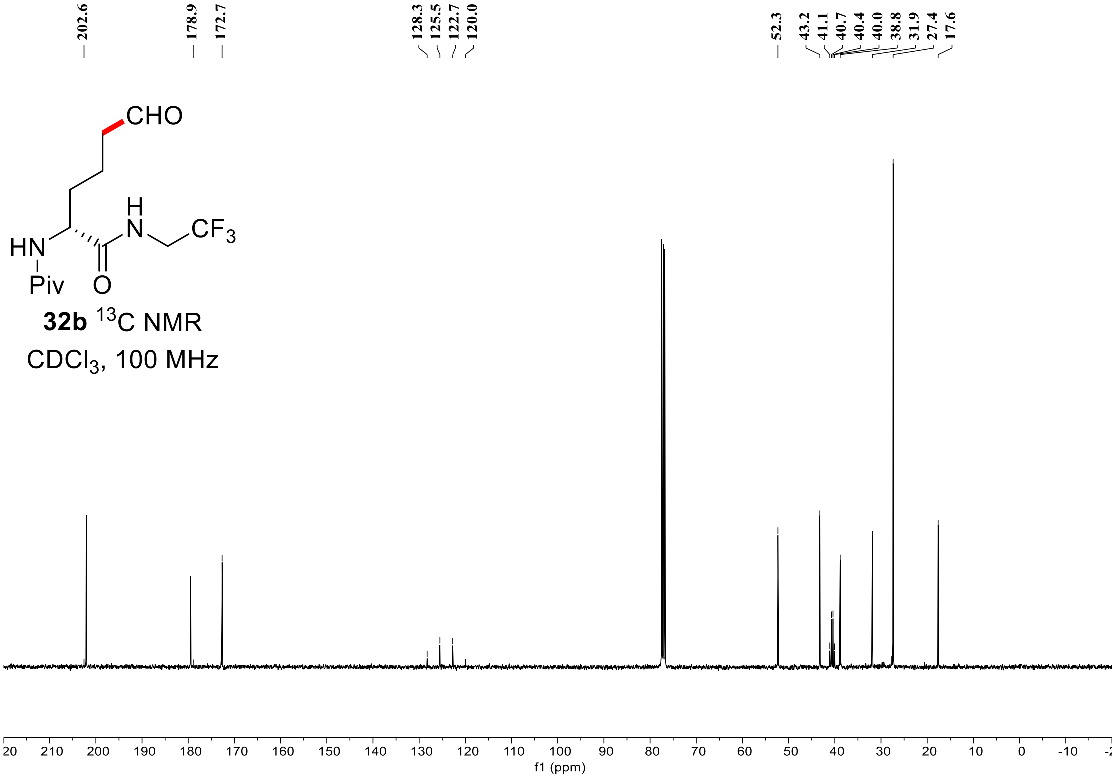
202.6
178.9
172.7
128.3
125.5
122.7
120.0
52.3
43.2
41.1
40.7
40.4
40.0
38.8
31.9
27.4
17.6
CHO
H
N
CF3
HN
Piv
O
32b 13C NMR
CDCl3, 100 MHz
210 200 190 180 170 160 150 140 130 120 110 100 90 80 70 60 50 40 30 20 10 0 -10
f1 (ppm)

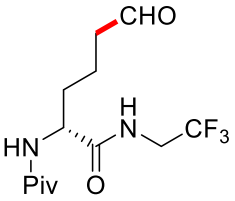

32b ^{19}F NMR

$CDCl_3$, 376 MHz

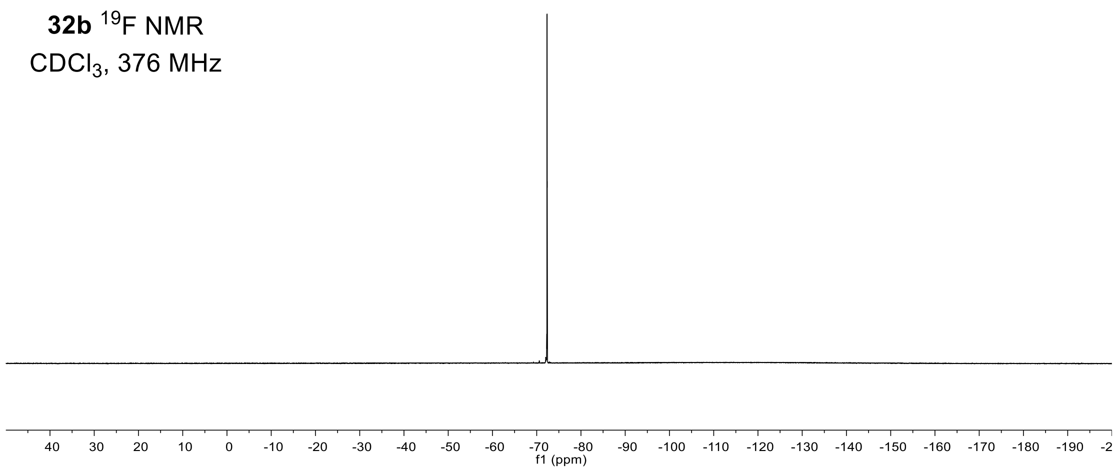

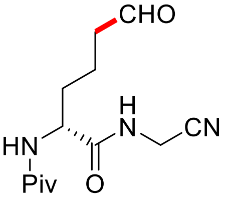

33b ^{1}H NMR

$CDCl_3$, 400 MHz

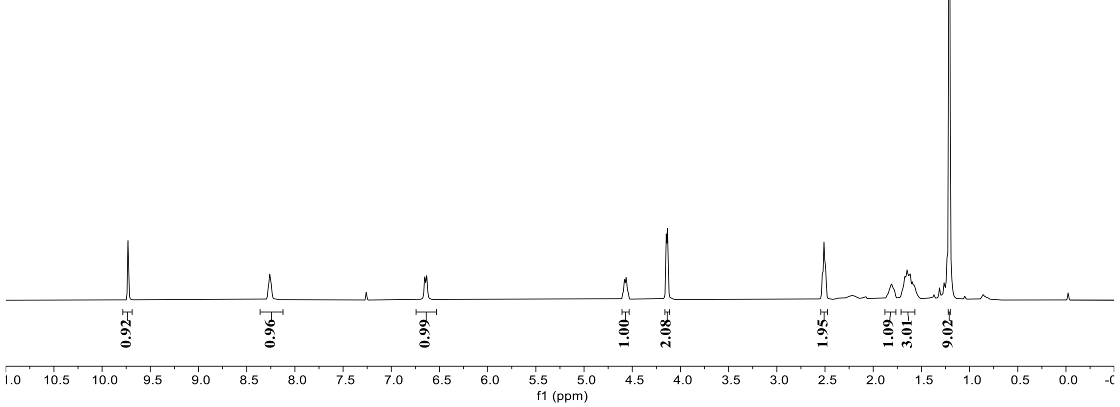

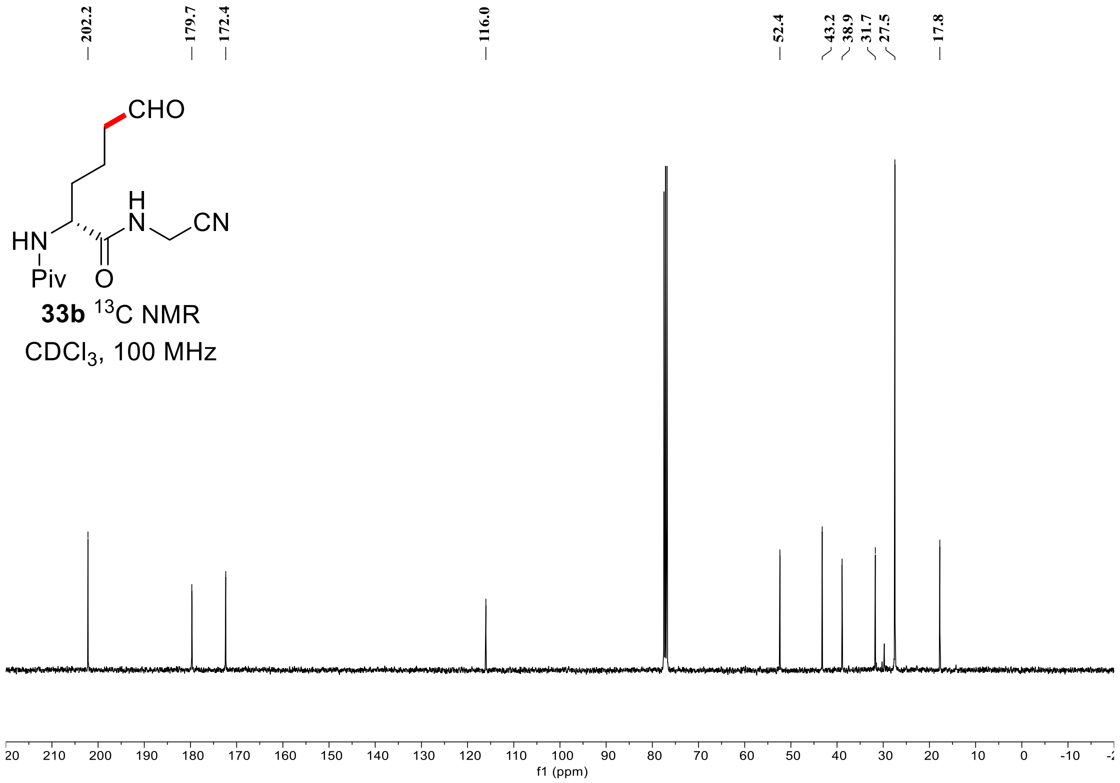
202.2
179.7
172.4
116.0
52.4
43.2
38.9
31.7
27.5
17.8
CHO
H
N
CN
HN
Piv
O
33b ^{13}C NMR
$CDCl_3$, 100 MHz
f1 (ppm)

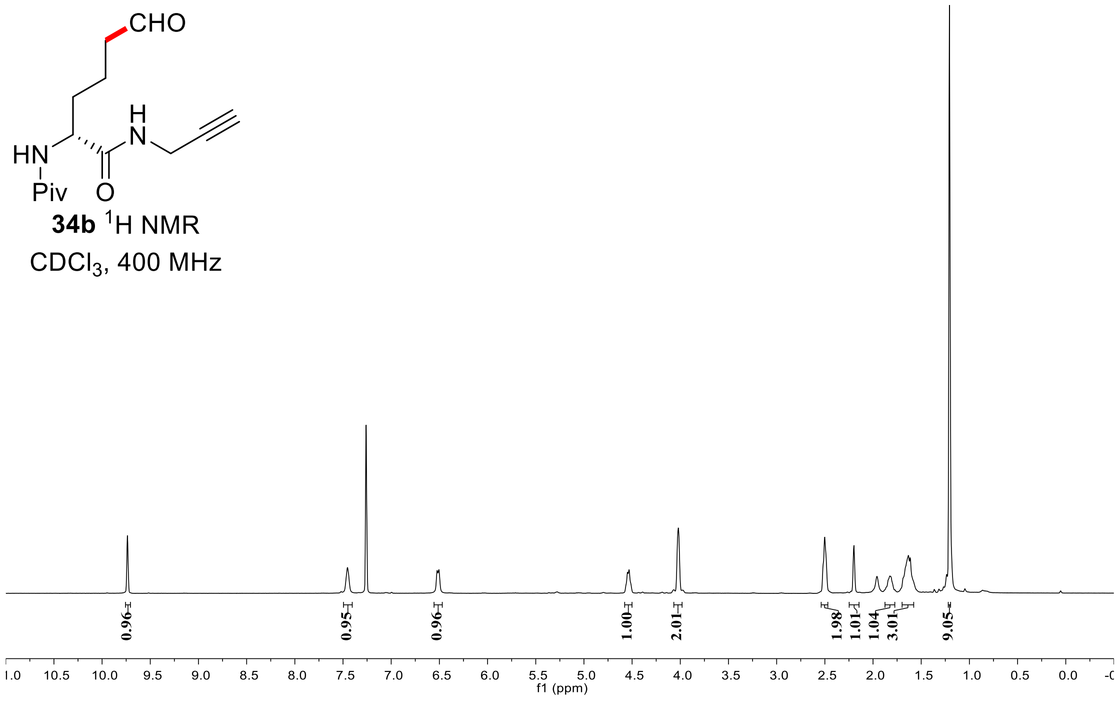
CHO
H
N
HN
Piv
O
34b ^{1}H NMR
$CDCl_3$, 400 MHz
0.96
0.95
0.96
1.00
2.01
1.98
1.01
1.04
3.01
9.05
f1 (ppm)

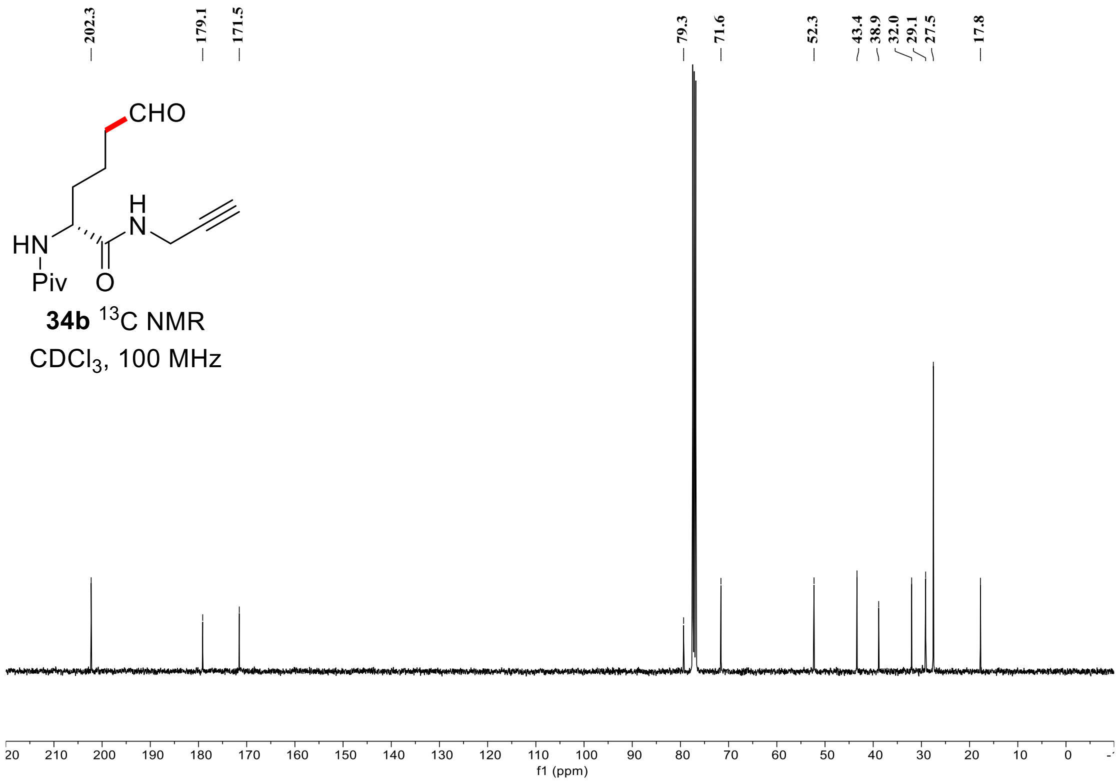

202.3
179.1
171.5
79.3
71.6
52.3
43.4
38.9
32.0
29.1
27.5
17.8
CHO
HN
Piv
O
H
N
34b ^{13}C NMR
$CDCl_3$, 100 MHz
f1 (ppm)

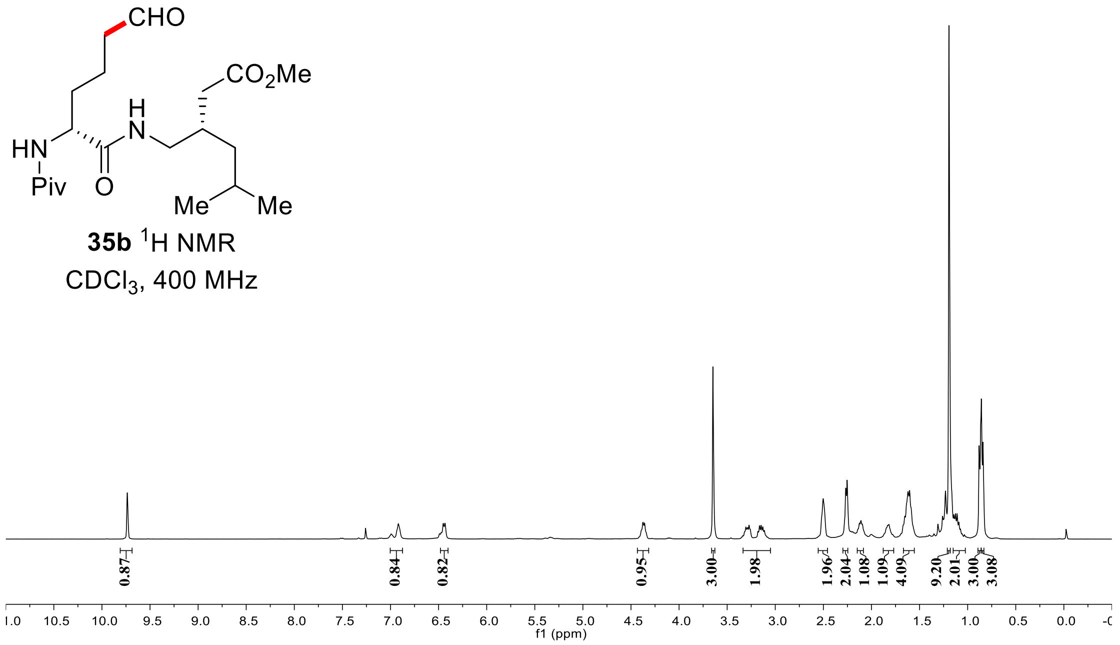

CHO
CO_2Me
HN
Piv
O
H
N
Me
Me
35b ^{1}H NMR
$CDCl_3$, 400 MHz
0.87
0.84
0.82
0.95
3.00
1.98
1.96
2.04
1.08
1.09
4.09
9.20
2.01
3.00
3.08
f1 (ppm)

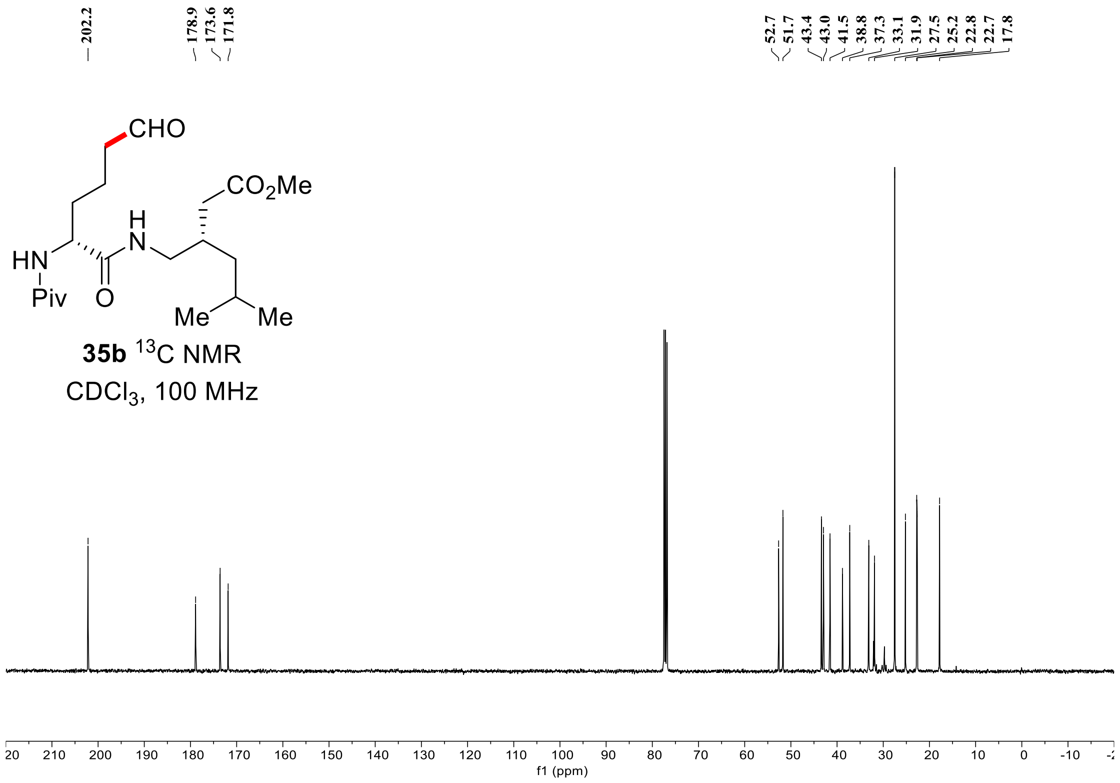
202.2
178.9
173.6
171.8
52.7
51.7
43.4
43.0
41.5
38.8
37.3
33.1
31.9
27.5
25.2
22.8
22.7
17.8
CHO
CO2Me
H
N
HN
Piv
O
Me
Me
35b 13C NMR
CDCl3, 100 MHz
f1 (ppm)

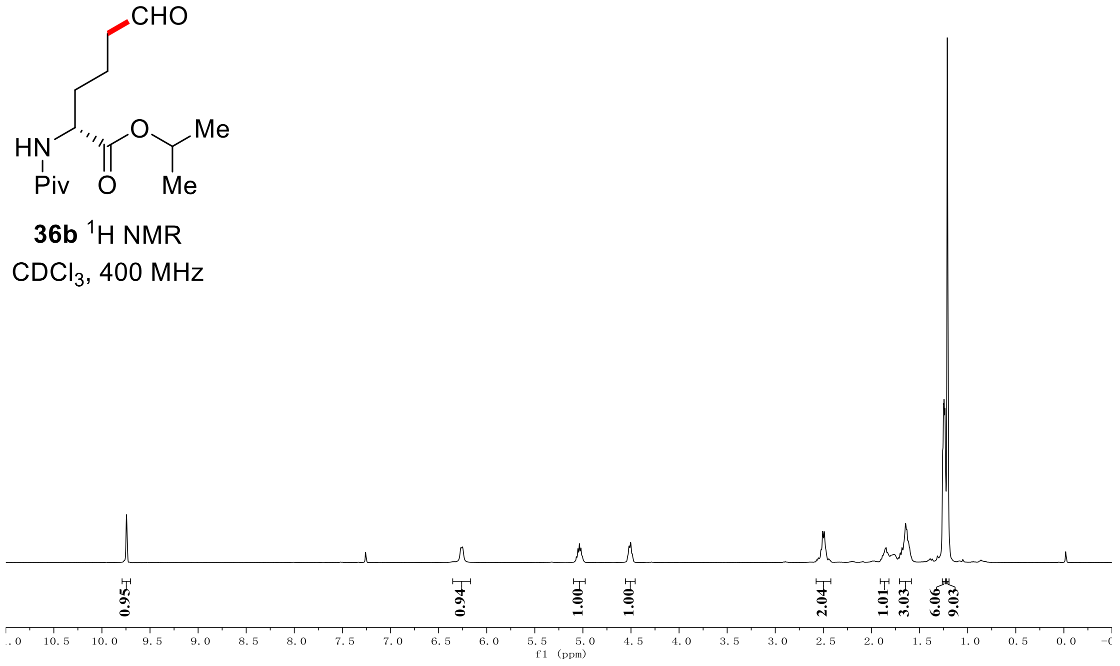
CHO
HN
Piv
O
O
Me
Me
36b 1H NMR
CDCl3, 400 MHz
0.95
0.94
1.00
1.00
2.04
1.01
3.03
6.06
9.03
f1 (ppm)

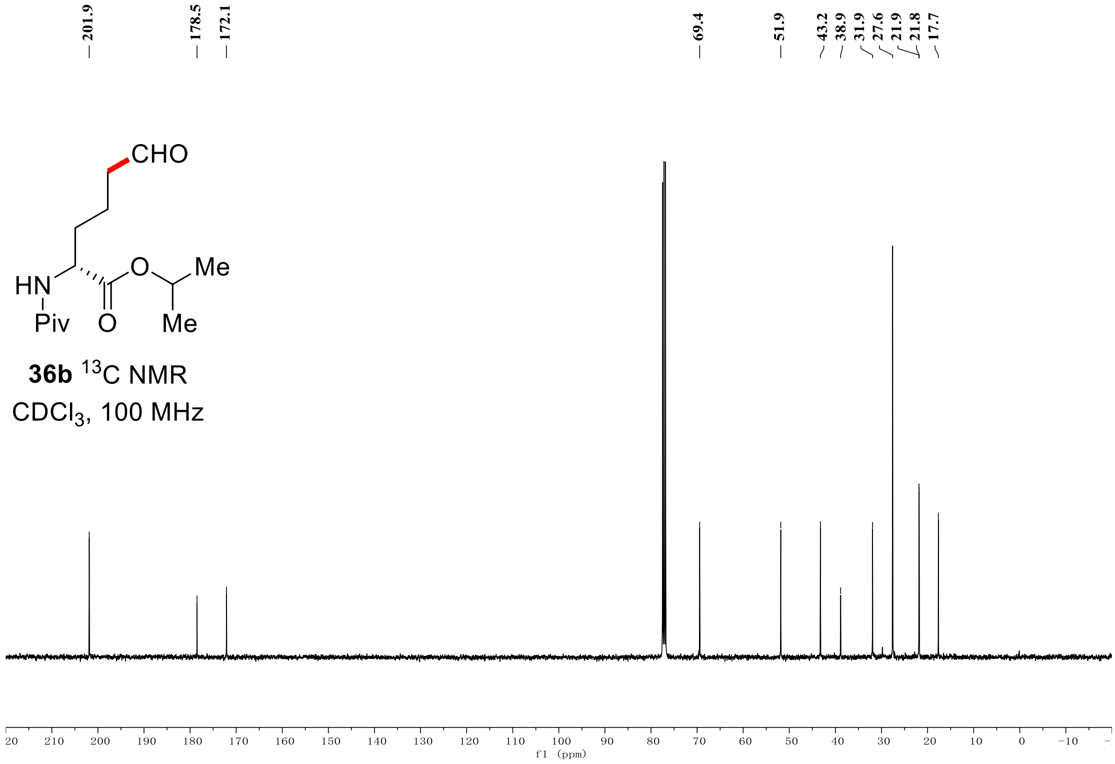
201.9
178.5
172.1
69.4
51.9
43.2
38.9
31.9
27.6
21.9
21.8
17.7
CHO
HN
Piv
O
Me
Me
36b 13C NMR
CDCl3, 100 MHz
f1 (ppm)

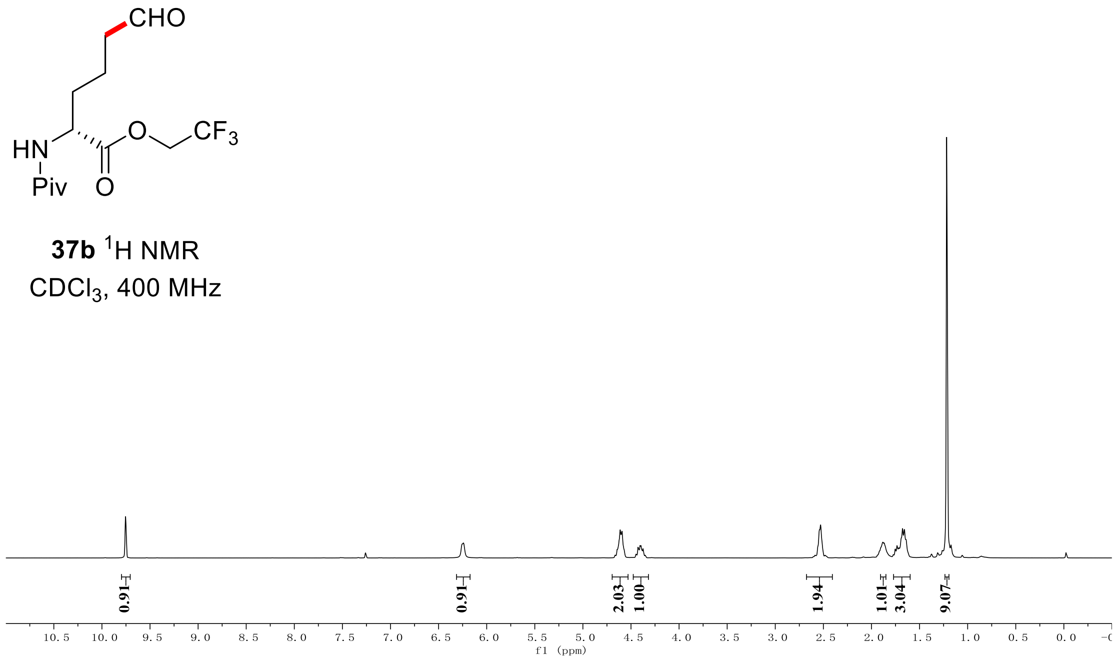
CHO
HN
Piv
O
CF3
37b 1H NMR
CDCl3, 400 MHz
0.91
0.91
2.03
1.00
1.94
1.01
3.04
9.07
f1 (ppm)

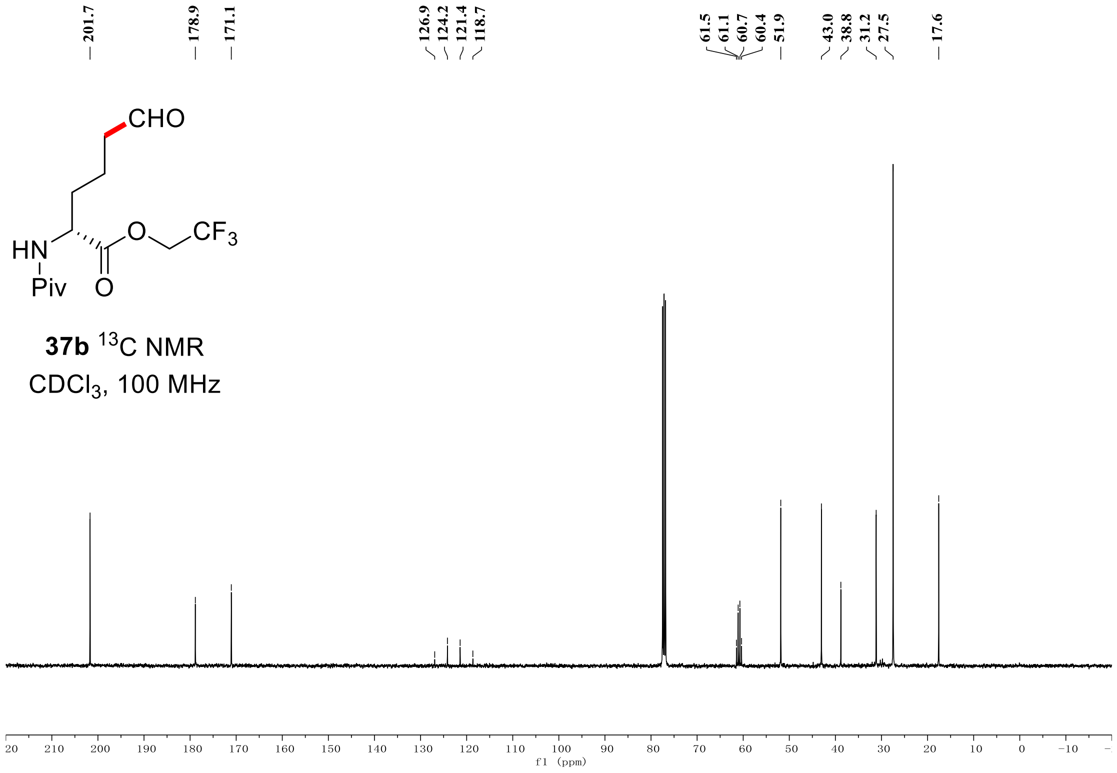
201.7
178.9
171.1
126.9
124.2
121.4
118.7
61.5
61.1
60.7
60.4
51.9
43.0
38.8
31.2
27.5
17.6
CHO
HN
Piv
O
CF3
37b 13C NMR
CDCl3, 100 MHz
f1 (ppm)

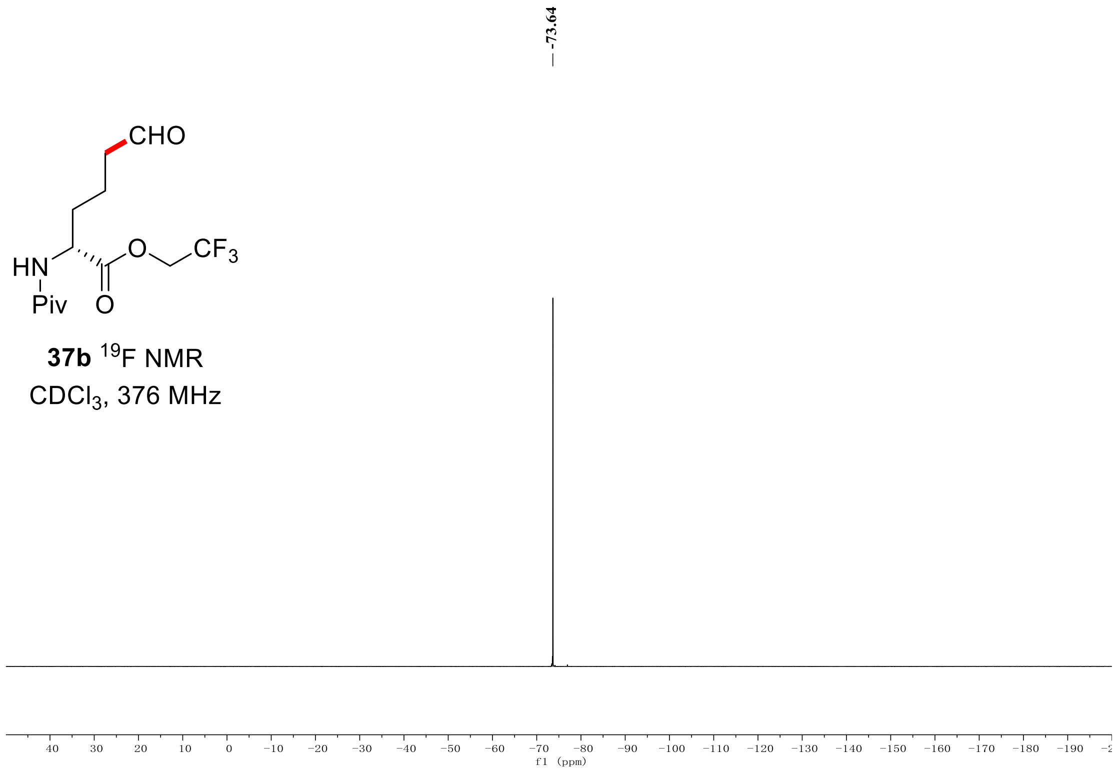
-73.64
CHO
HN
Piv
O
CF3
37b 19F NMR
CDCl3, 376 MHz
f1 (ppm)

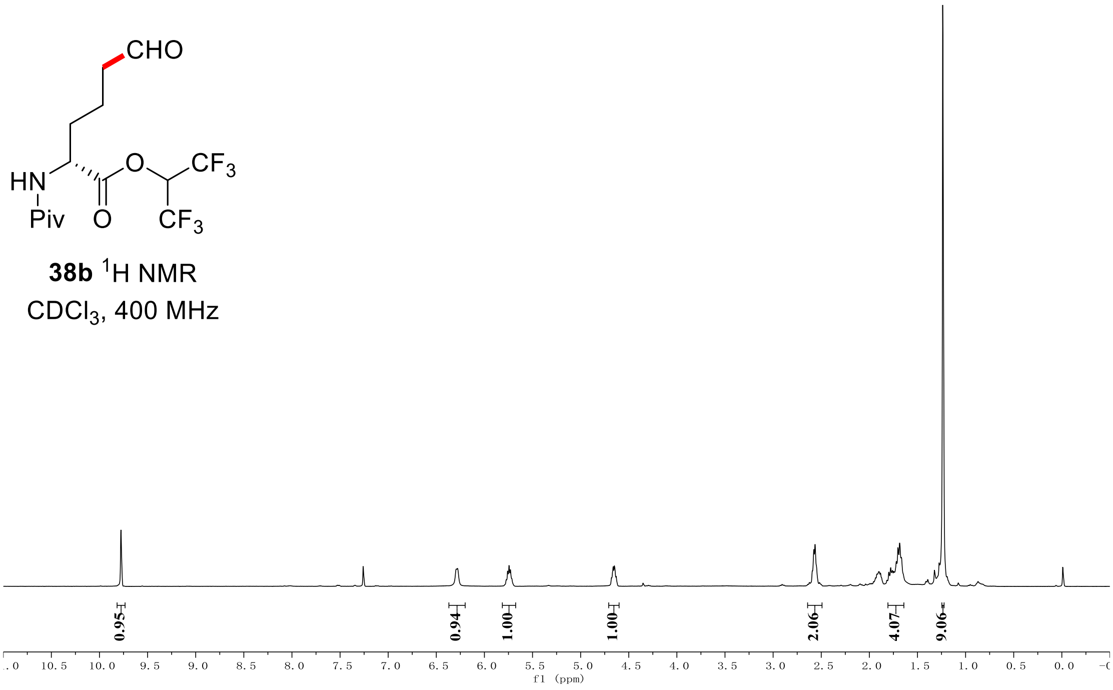

38b ^{1}H NMR

$CDCl_3$, 400 MHz

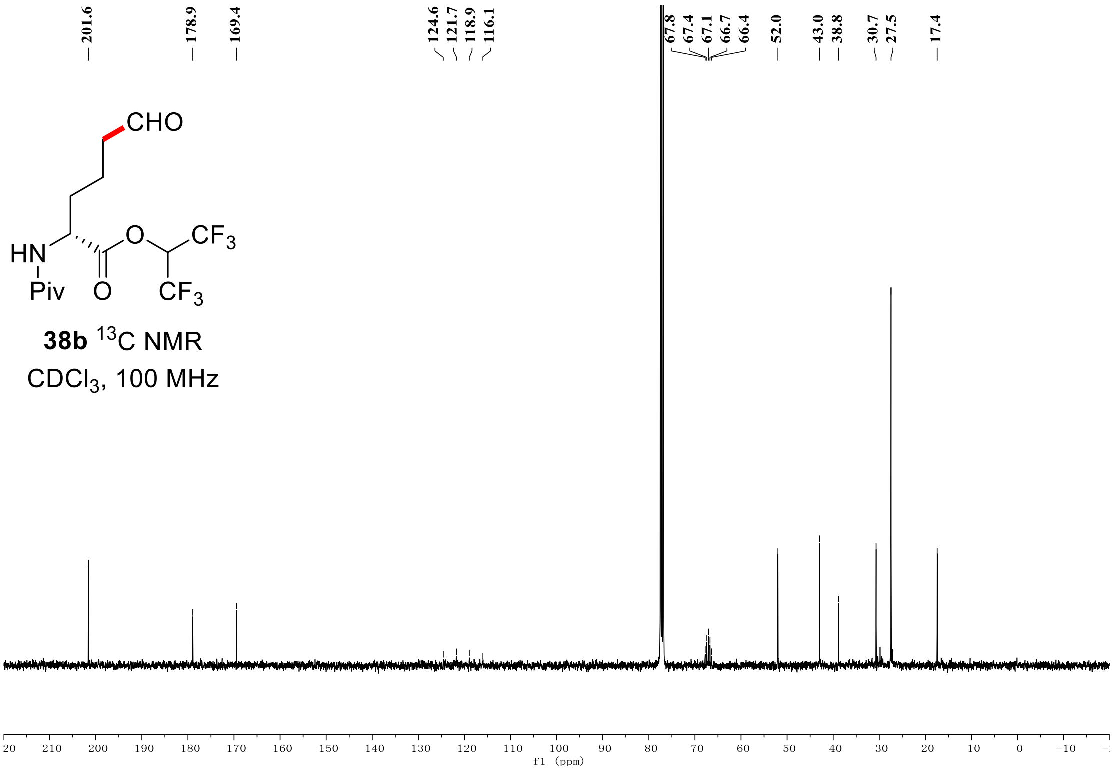

38b ^{13}C NMR

$CDCl_3$, 100 MHz

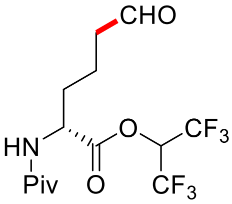

38b ^{19}F NMR
$CDCl_3$, 376 MHz

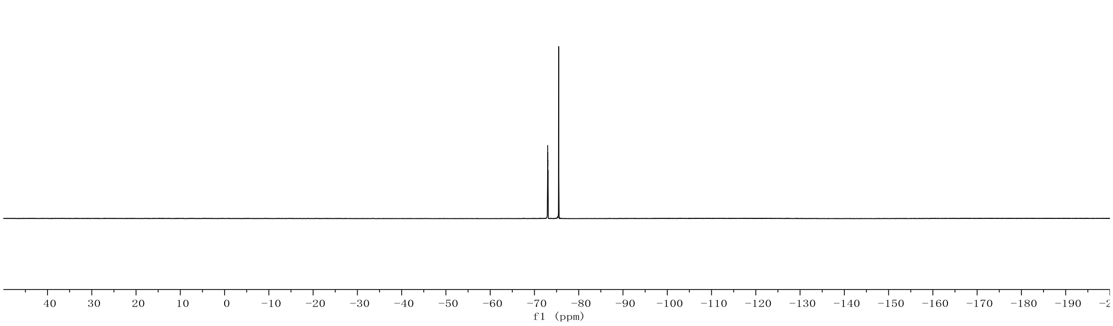

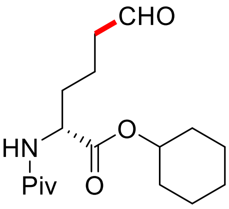

39b ^{1}H NMR
$CDCl_3$, 400 MHz

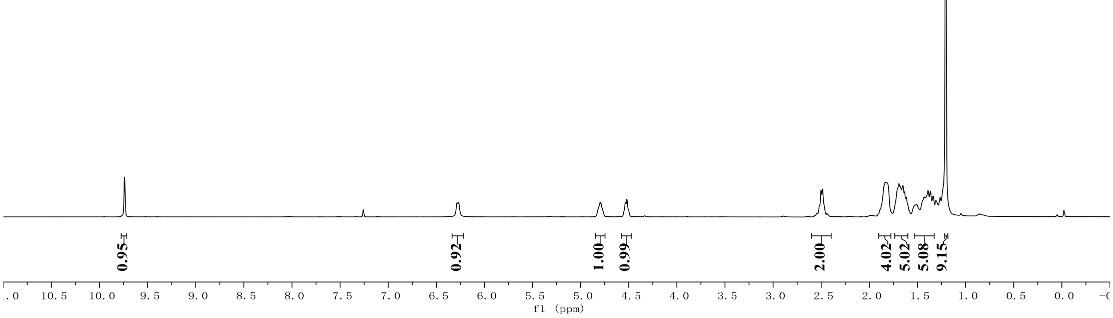

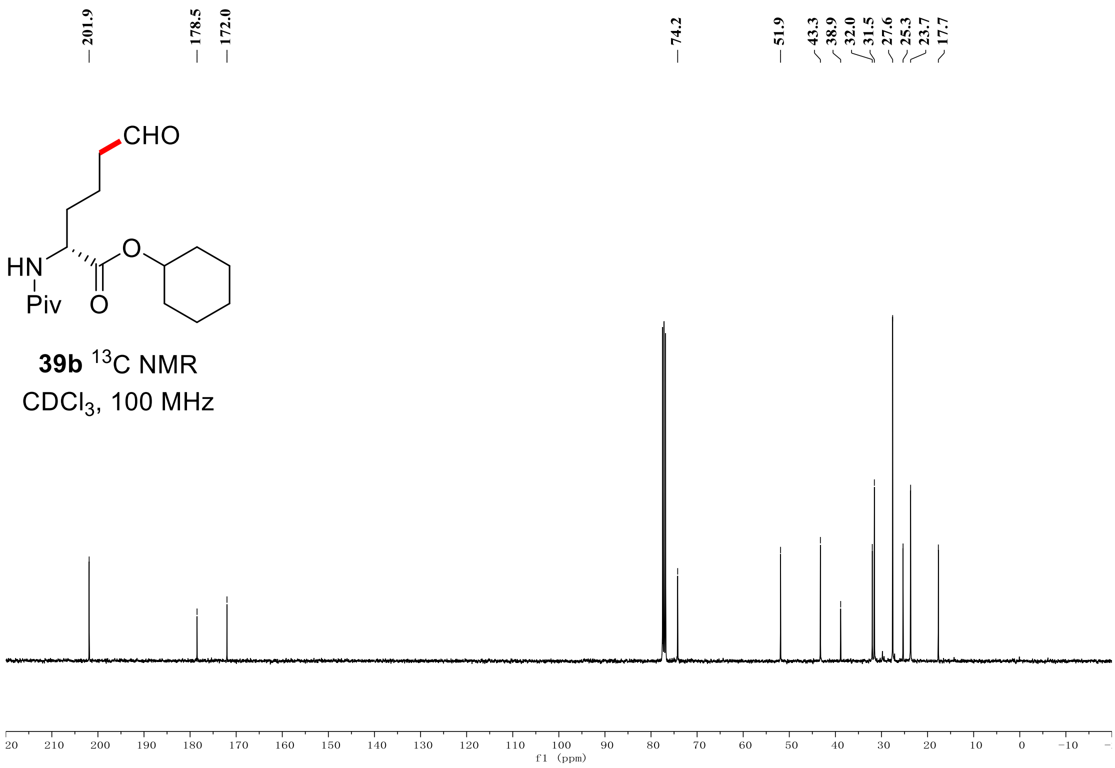
201.9
178.5
172.0
74.2
51.9
43.3
38.9
32.0
31.5
27.6
25.3
23.7
17.7
CHO
HN
Piv
O
O
39b 13C NMR
CDCl3, 100 MHz
f1 (ppm)

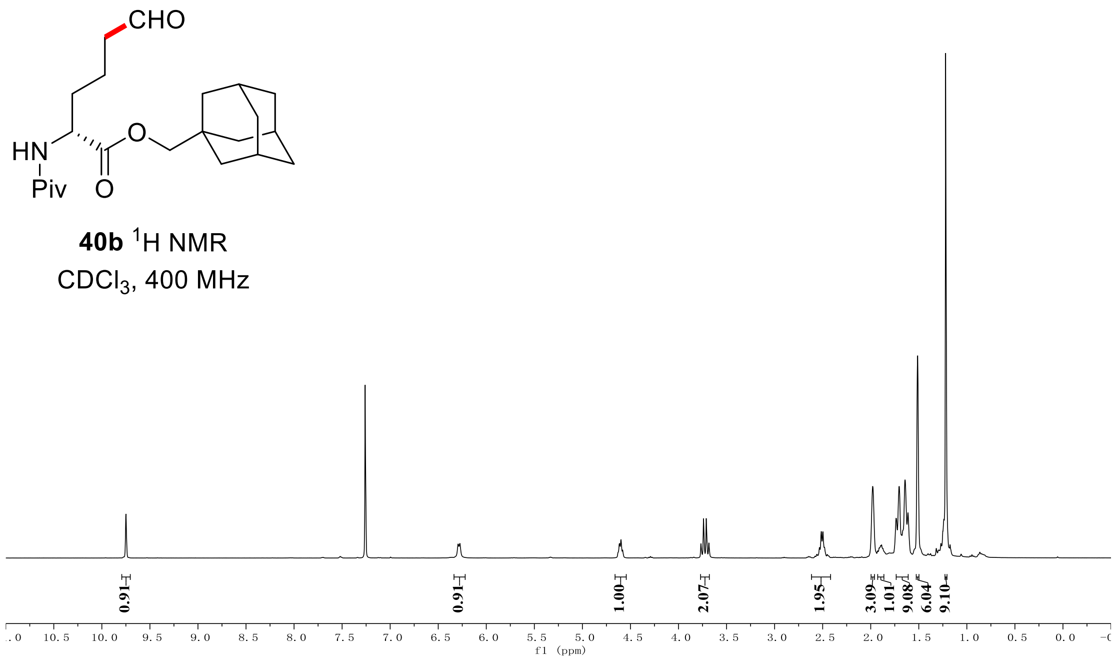
CHO
HN
Piv
O
O
40b 1H NMR
CDCl3, 400 MHz
0.91
0.91
1.00
2.07
1.95
3.09
1.01
9.08
6.04
9.10
f1 (ppm)

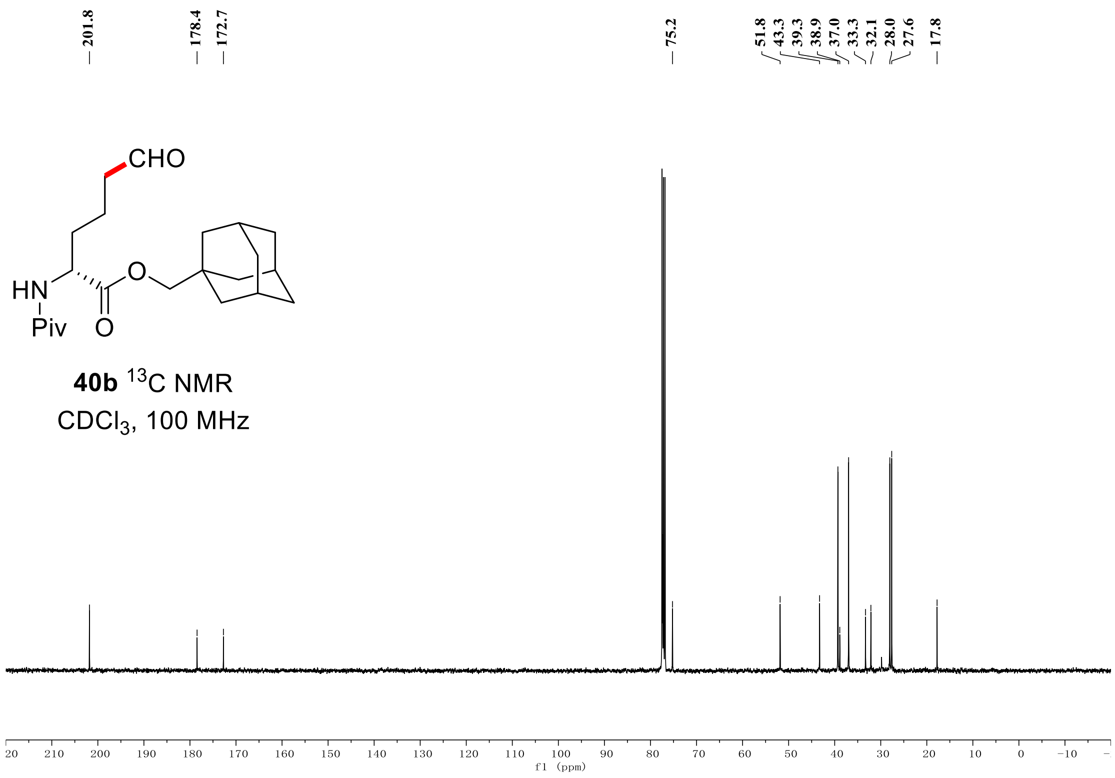

201.8
178.4
172.7
75.2
51.8
43.3
39.3
38.9
37.0
33.3
32.1
28.0
27.6
17.8
CHO
HN
Piv
O
O
40b 13C NMR
CDCl3, 100 MHz
f1 (ppm)

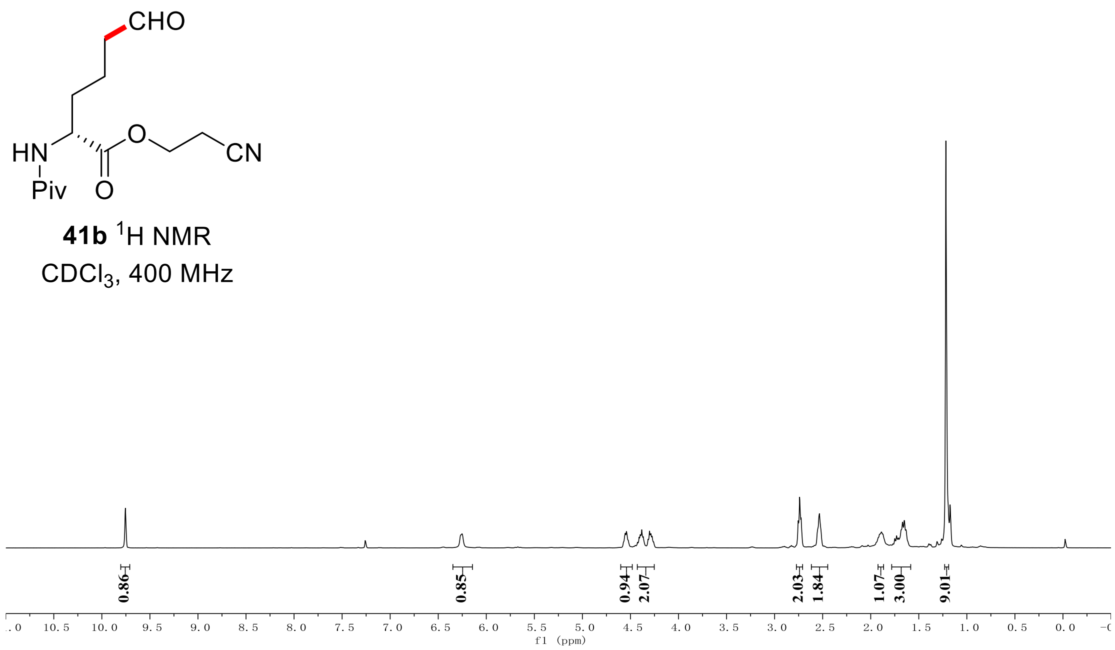

CHO
HN
Piv
O
O
CN
41b 1H NMR
CDCl3, 400 MHz
0.86
0.85
0.94
2.07
2.03
1.84
1.07
3.00
9.01
f1 (ppm)

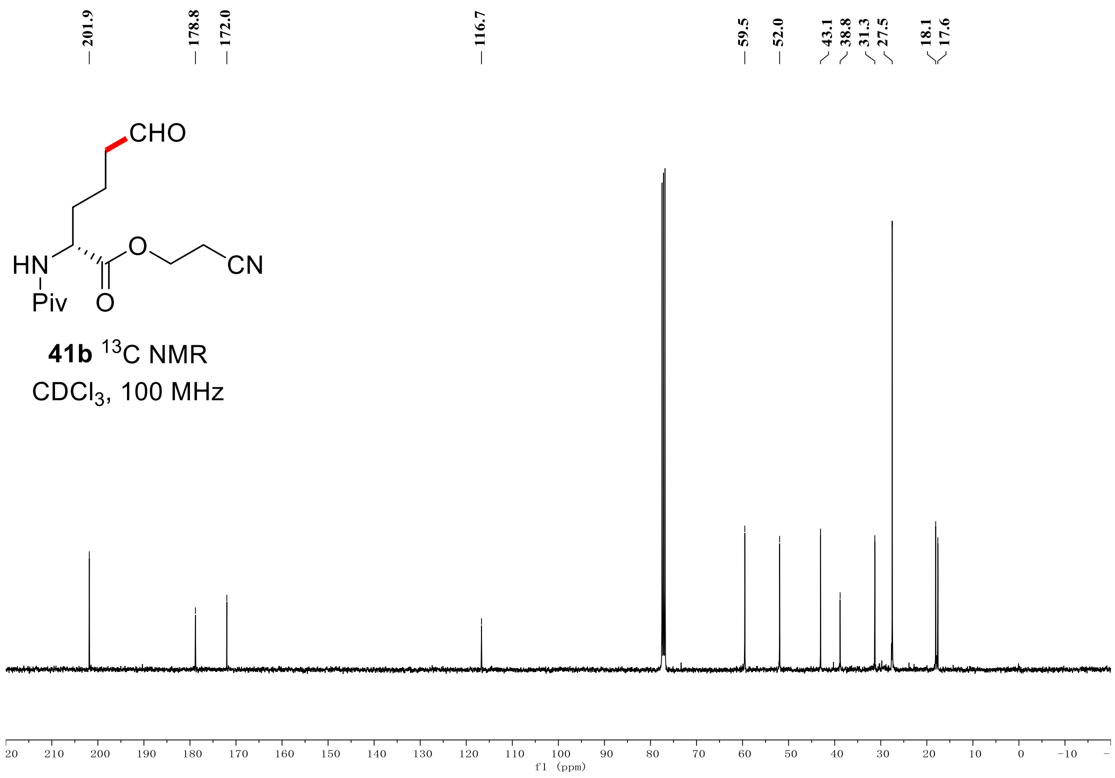
201.9
178.8
172.0
116.7
59.5
52.0
43.1
38.8
31.3
27.5
18.1
17.6
CHO
HN
Piv
O
O
CN
41b 13C NMR
CDCl3, 100 MHz
f1 (ppm)

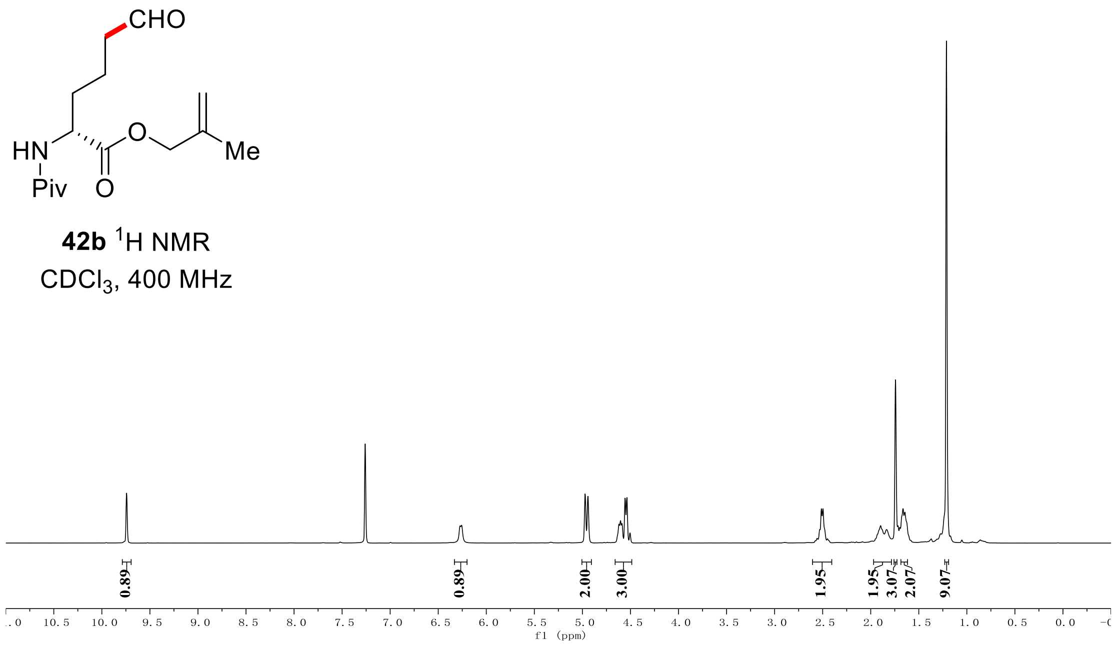
CHO
HN
Piv
O
O
Me
42b 1H NMR
CDCl3, 400 MHz
0.89
0.89
2.00
3.00
1.95
1.95
3.07
2.07
9.07
f1 (ppm)

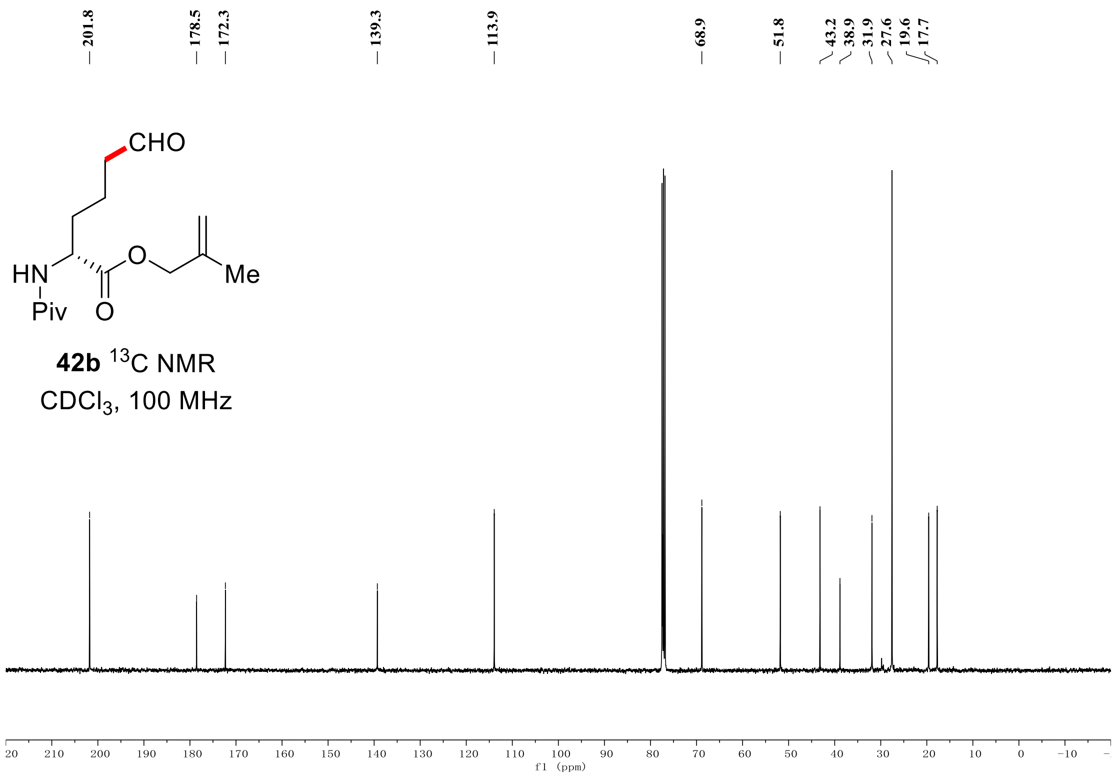

42b ^{13}C NMR
$CDCl_3$, 100 MHz

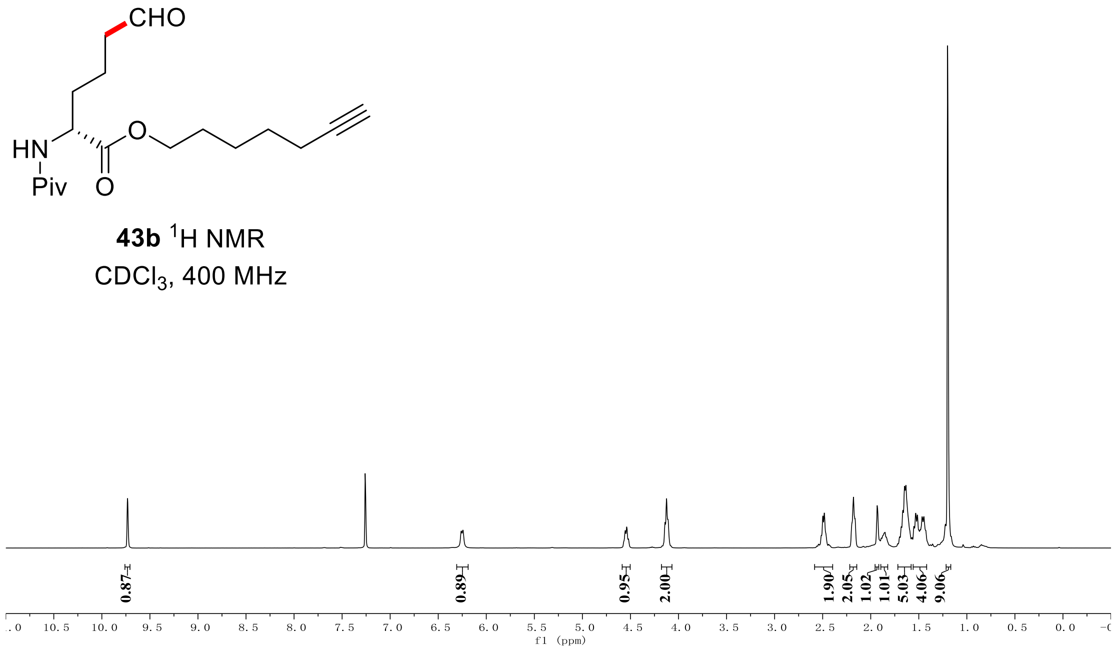

43b ^{1}H NMR
$CDCl_3$, 400 MHz

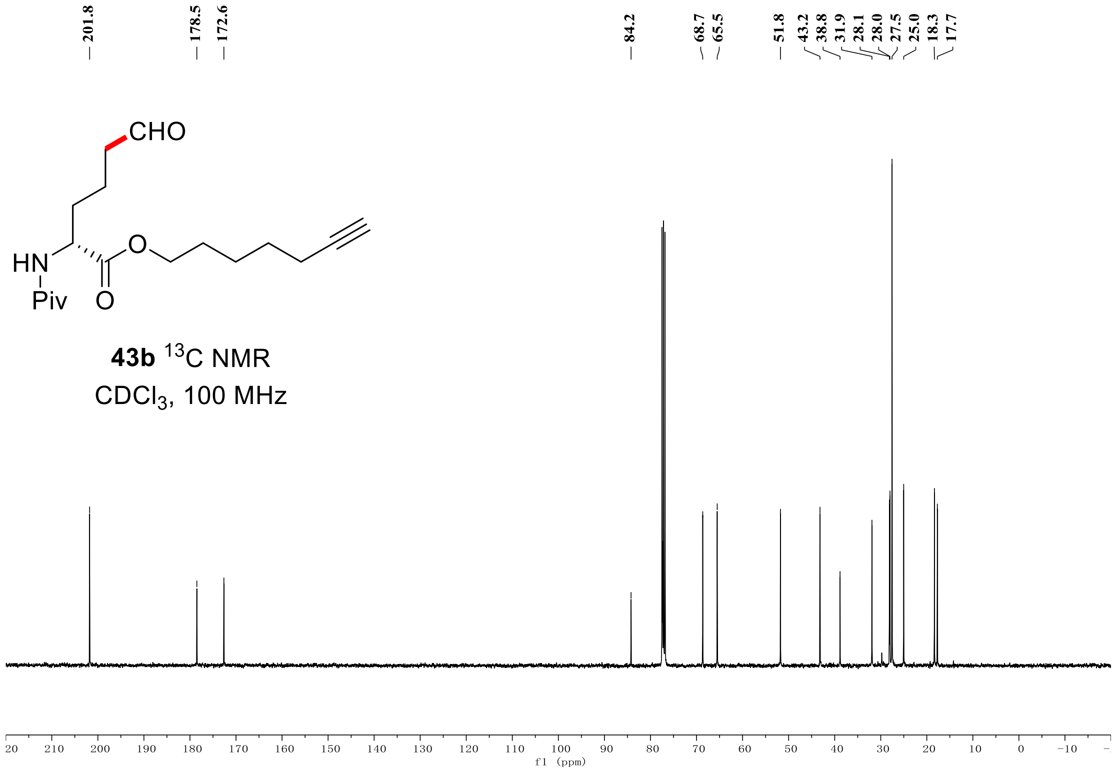
201.8
178.5
172.6
84.2
68.7
65.5
51.8
43.2
38.8
31.9
28.1
28.0
27.5
25.0
18.3
17.7
CHO
HN
Piv
O
O
43b ¹³C NMR
CDCl₃, 100 MHz
f1 (ppm)

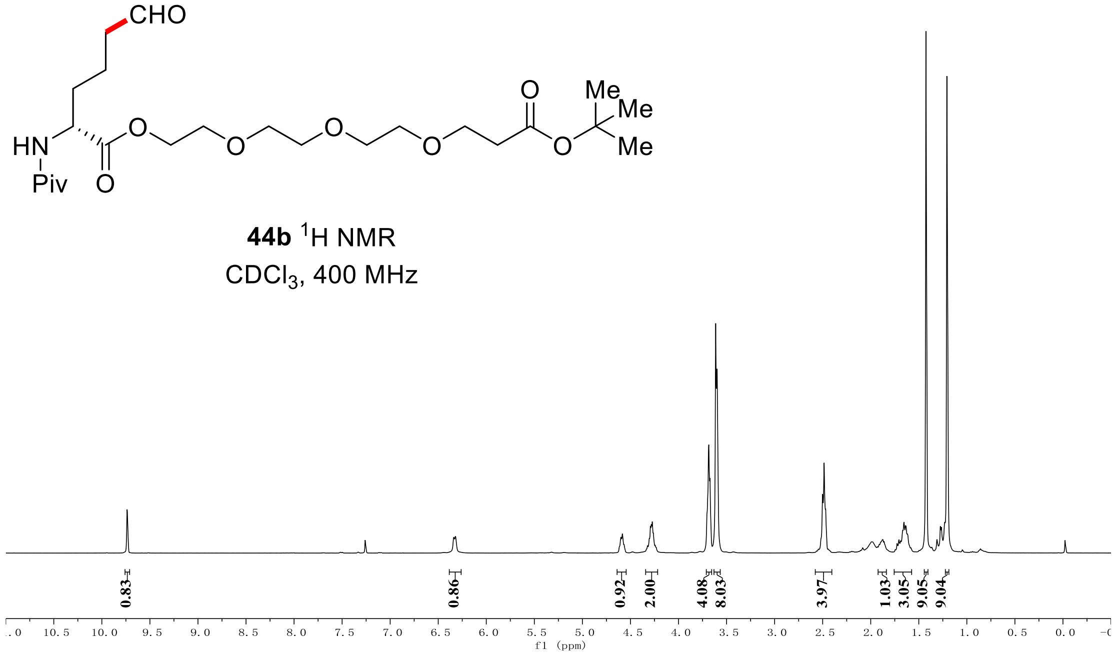
CHO
HN
Piv
O
O
O
O
O
O
O
Me
Me
Me
44b ¹H NMR
CDCl₃, 400 MHz
0.83
0.86
0.92
2.00
4.08
8.03
3.97
1.03
3.05
9.05
9.04
f1 (ppm)

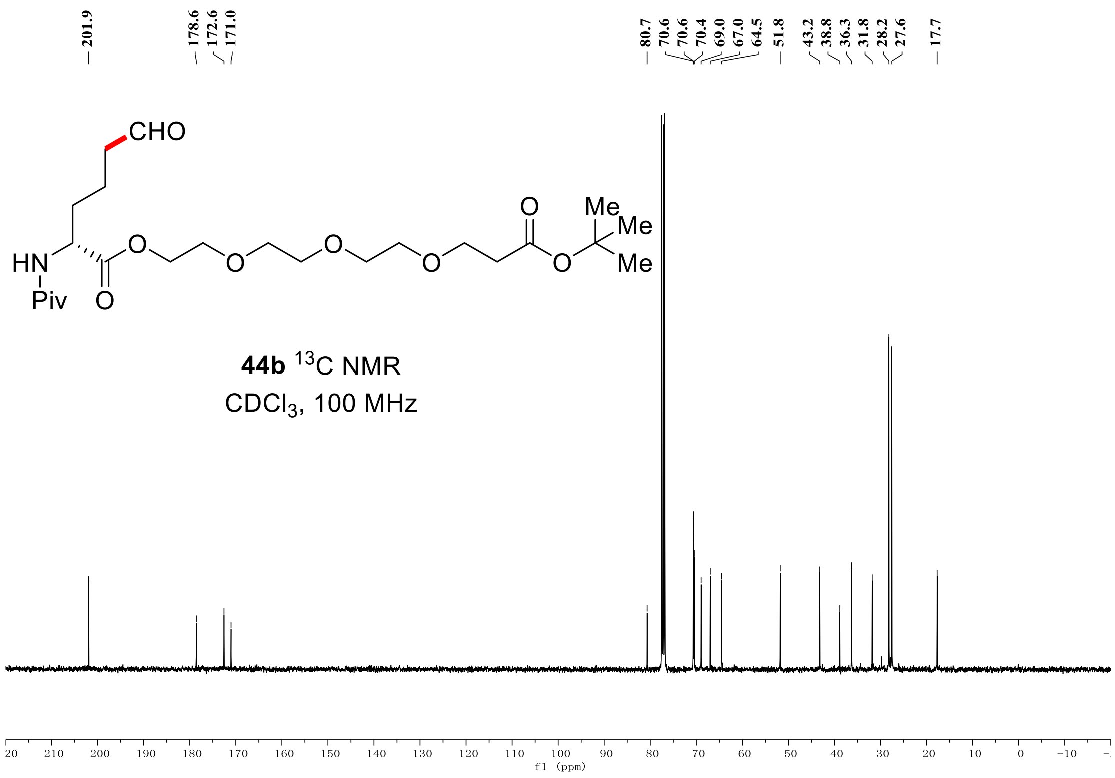

201.9
178.6
172.6
171.0
80.7
70.6
70.6
70.4
69.0
67.0
64.5
51.8
43.2
38.8
36.3
31.8
28.2
27.6
17.7
CHO
HN
Piv
O
Me
Me
Me
44b ¹³C NMR
CDCl₃, 100 MHz
f1 (ppm)

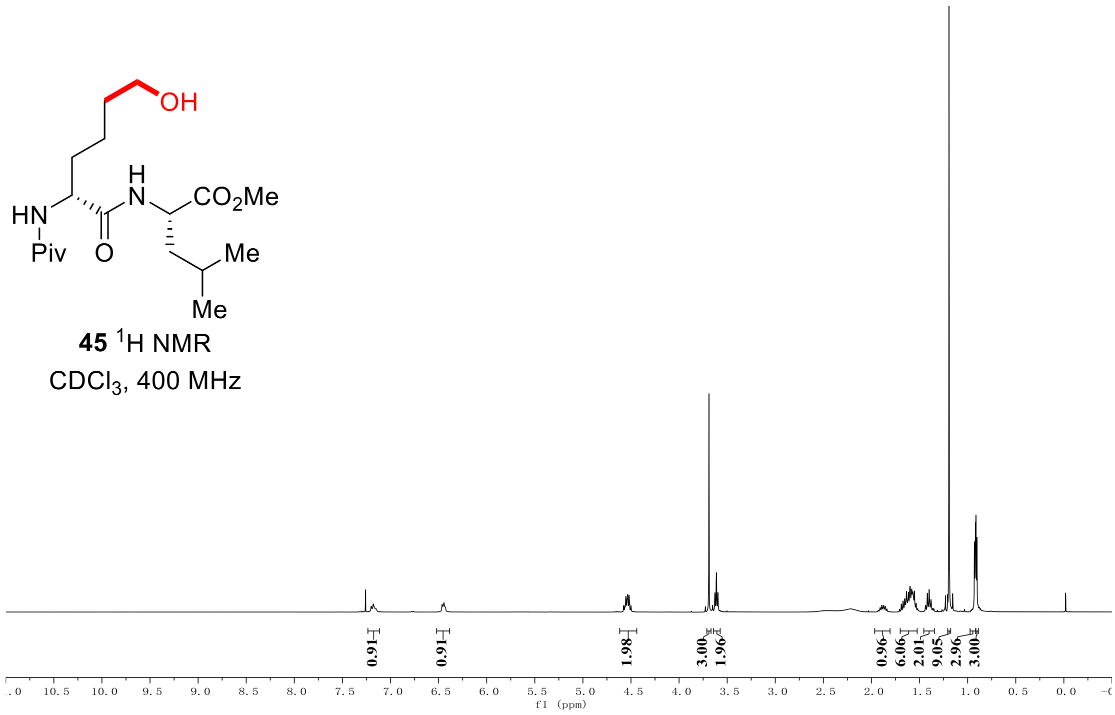

OH
H
N
CO₂Me
HN
Piv
O
Me
Me
45 ¹H NMR
CDCl₃, 400 MHz
0.91
0.91
1.98
3.00
1.96
0.96
6.06
2.01
9.05
2.96
3.00
f1 (ppm)

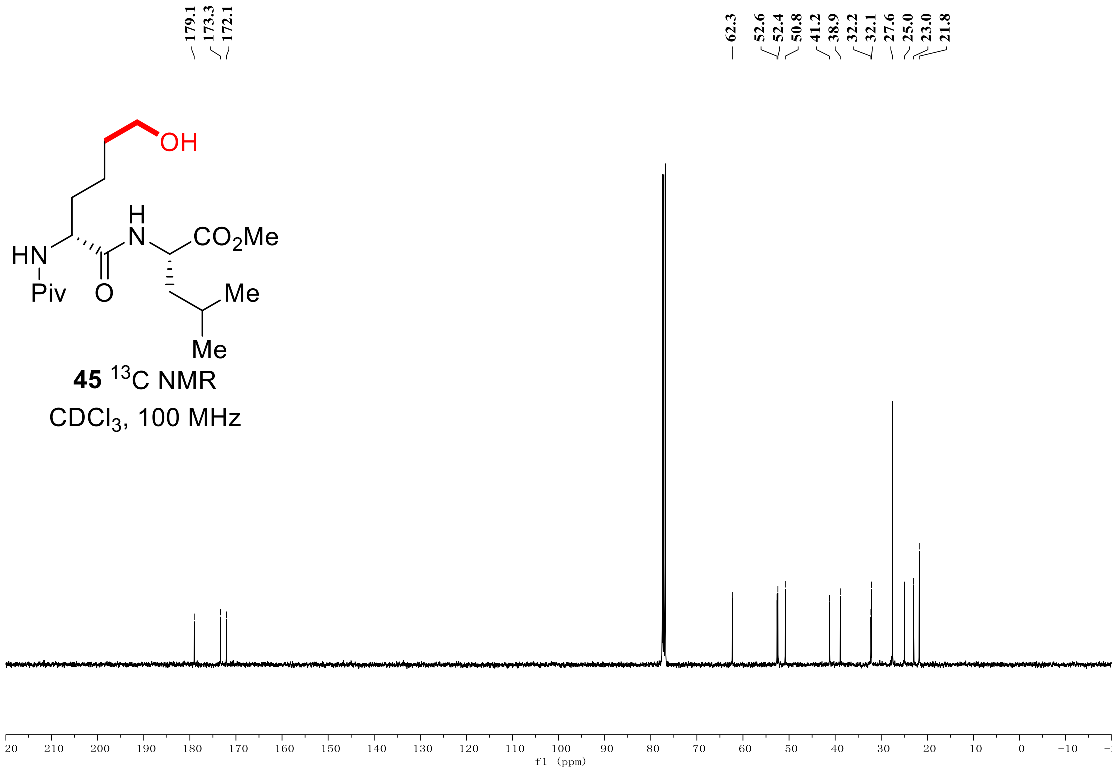
179.1
173.3
172.1
62.3
52.6
52.4
50.8
41.2
38.9
32.2
32.1
27.6
25.0
23.0
21.8
OH
HN
Piv
O
H
N
CO_2Me
Me
Me
45 ^{13}C NMR
$CDCl_3$, 100 MHz
f1 (ppm)

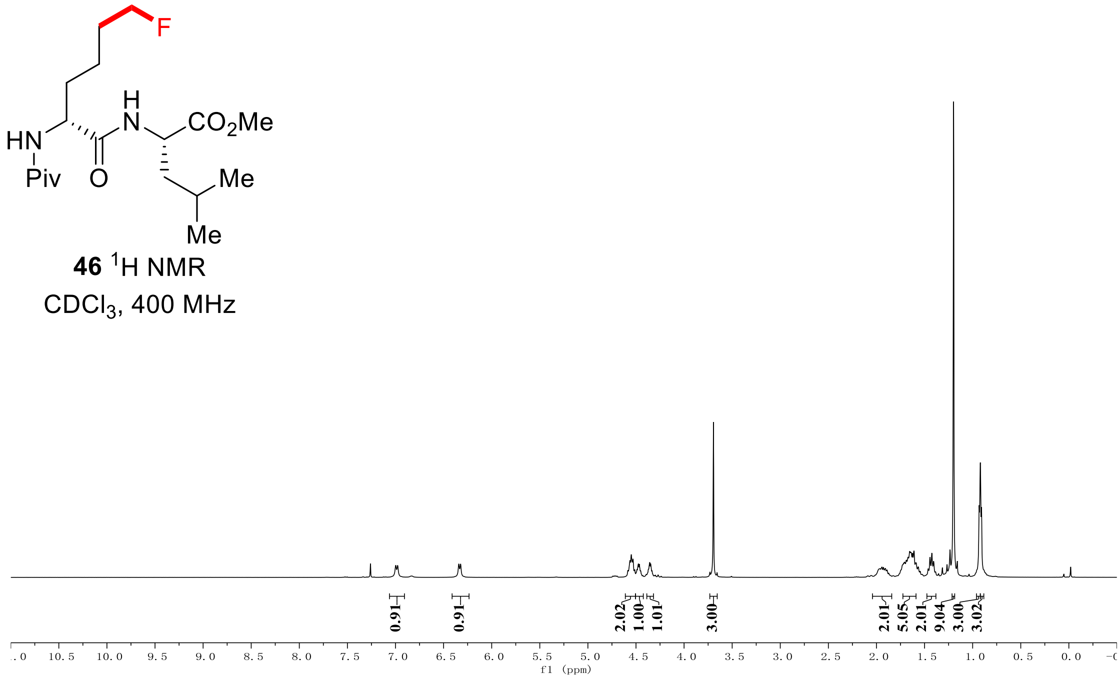
F
HN
Piv
O
H
N
CO_2Me
Me
Me
46 ^{1}H NMR
$CDCl_3$, 400 MHz
0.91
0.91
2.02
1.00
1.01
3.00
2.01
5.05
2.01
9.04
3.00
3.02
f1 (ppm)

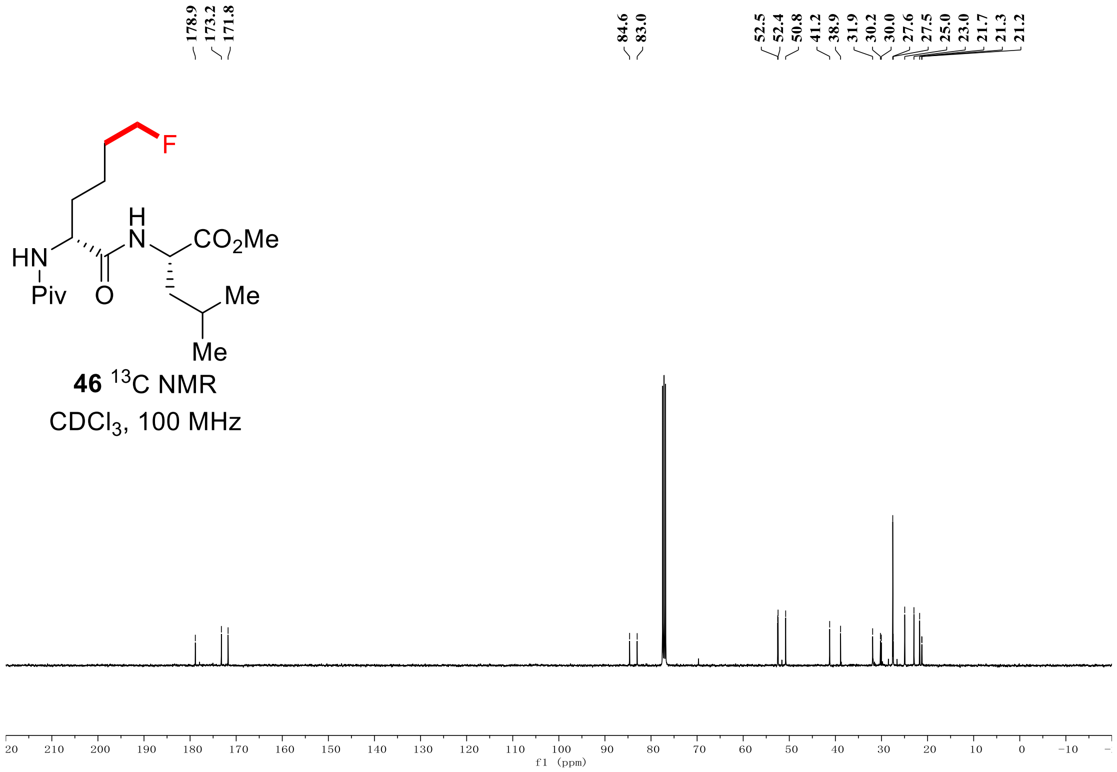
178.9
173.2
171.8
84.6
83.0
52.5
52.4
50.8
41.2
38.9
31.9
30.2
30.0
27.6
27.5
25.0
23.0
21.7
21.3
21.2
F
H
N
CO_2Me
HN
Piv
O
Me
Me
46 ^{13}C NMR
$CDCl_3$, 100 MHz
f1 (ppm)

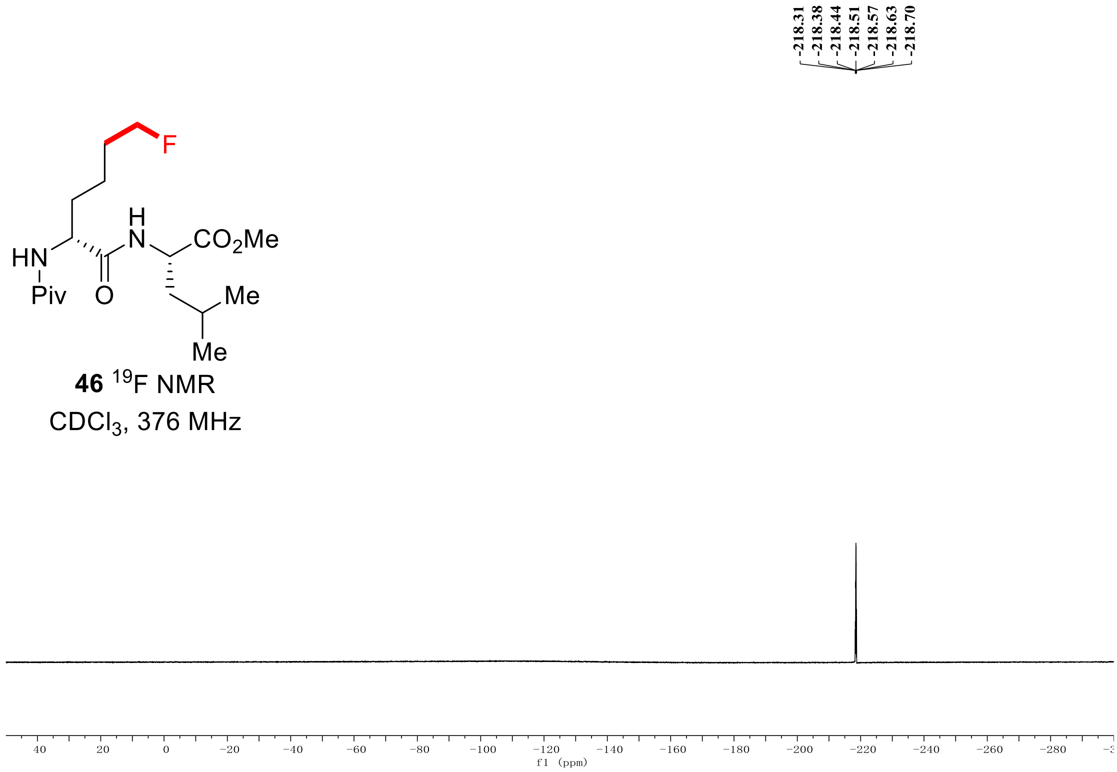
-218.31
-218.38
-218.44
-218.51
-218.57
-218.63
-218.70
F
H
N
CO_2Me
HN
Piv
O
Me
Me
46 ^{19}F NMR
$CDCl_3$, 376 MHz
f1 (ppm)

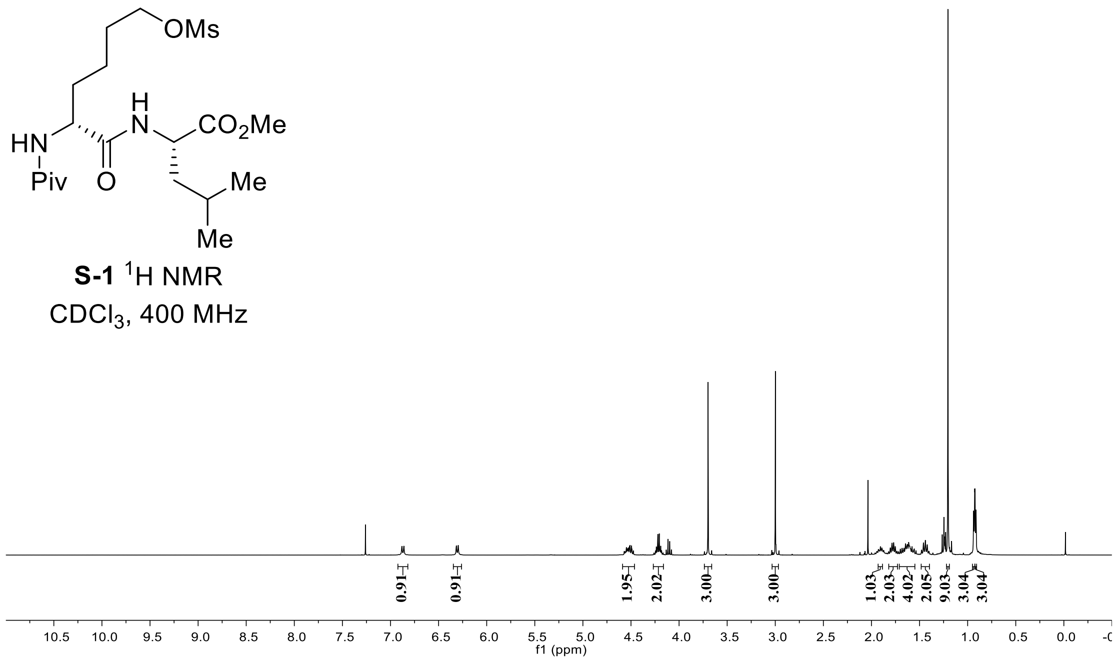
OMs
HN
Piv
H
N
O
CO2Me
Me
Me
S-1 1H NMR
CDCl3, 400 MHz
0.91
0.91
1.95
2.02
3.00
3.00
1.03
2.03
4.02
2.05
9.03
3.04
3.04
f1 (ppm)

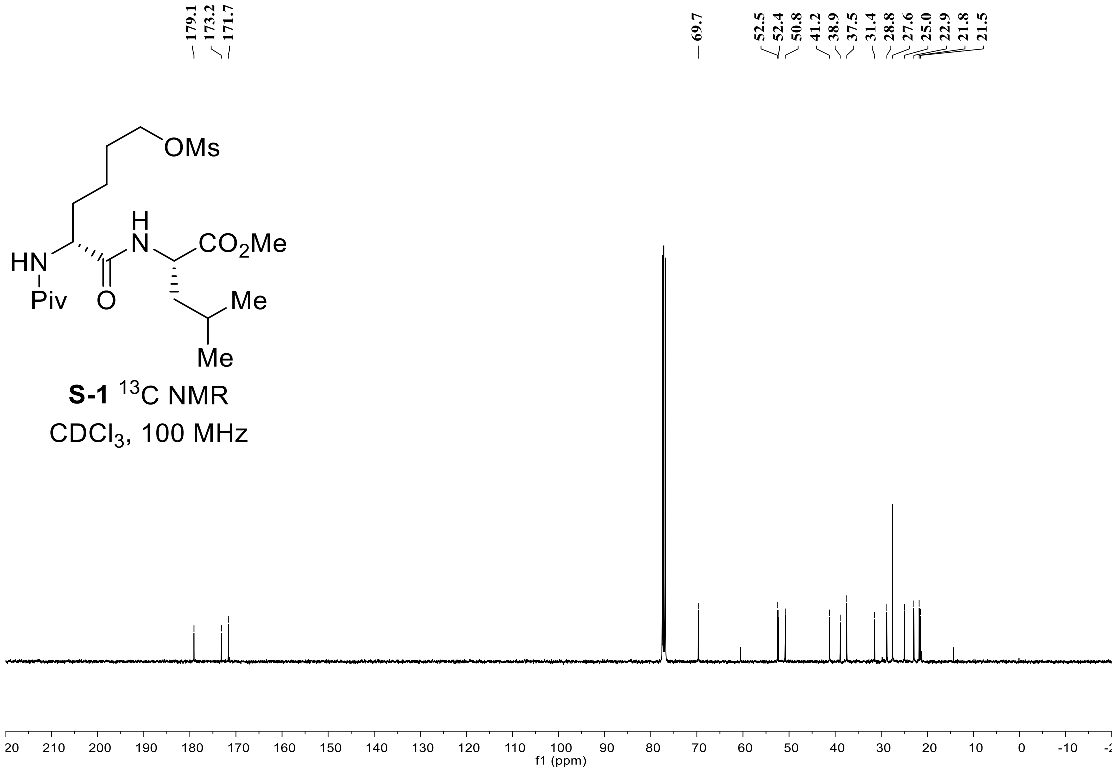
179.1
173.2
171.7
69.7
52.5
52.4
50.8
41.2
38.9
37.5
31.4
28.8
27.6
25.0
22.9
21.8
21.5
OMs
HN
Piv
H
N
O
CO2Me
Me
Me
S-1 13C NMR
CDCl3, 100 MHz
f1 (ppm)

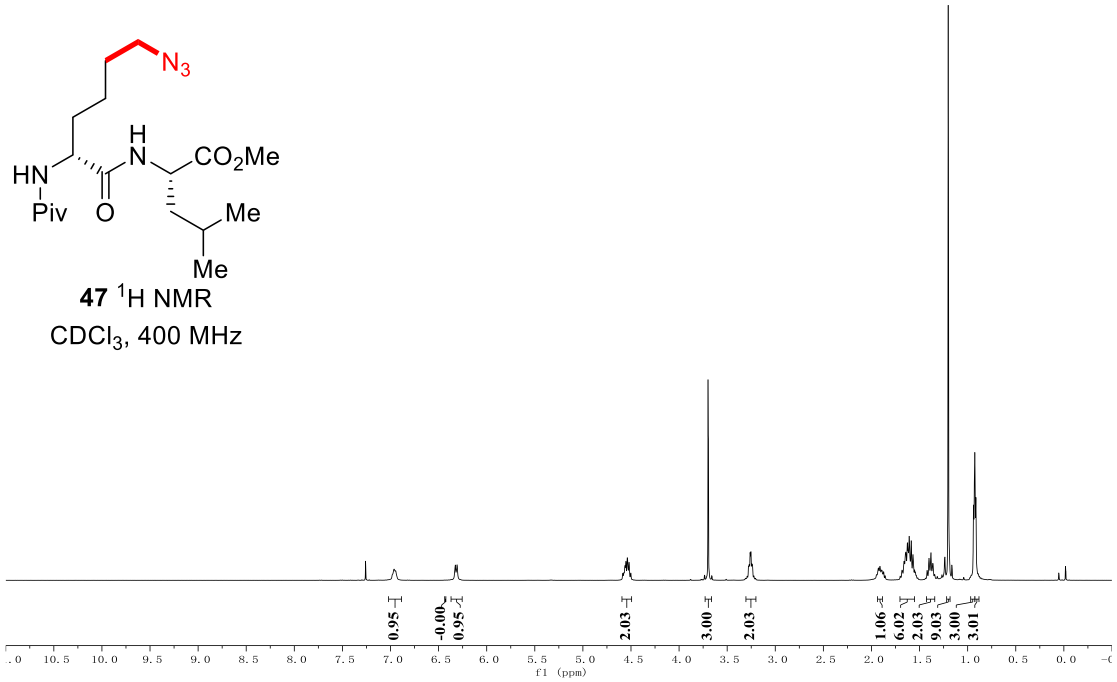
N3
H
N
CO2Me
HN
Piv
O
Me
Me
47 1H NMR
CDCl3, 400 MHz
0.95
-0.00
0.95
2.03
3.00
2.03
1.06
6.02
2.03
9.03
3.00
3.01
f1 (ppm)

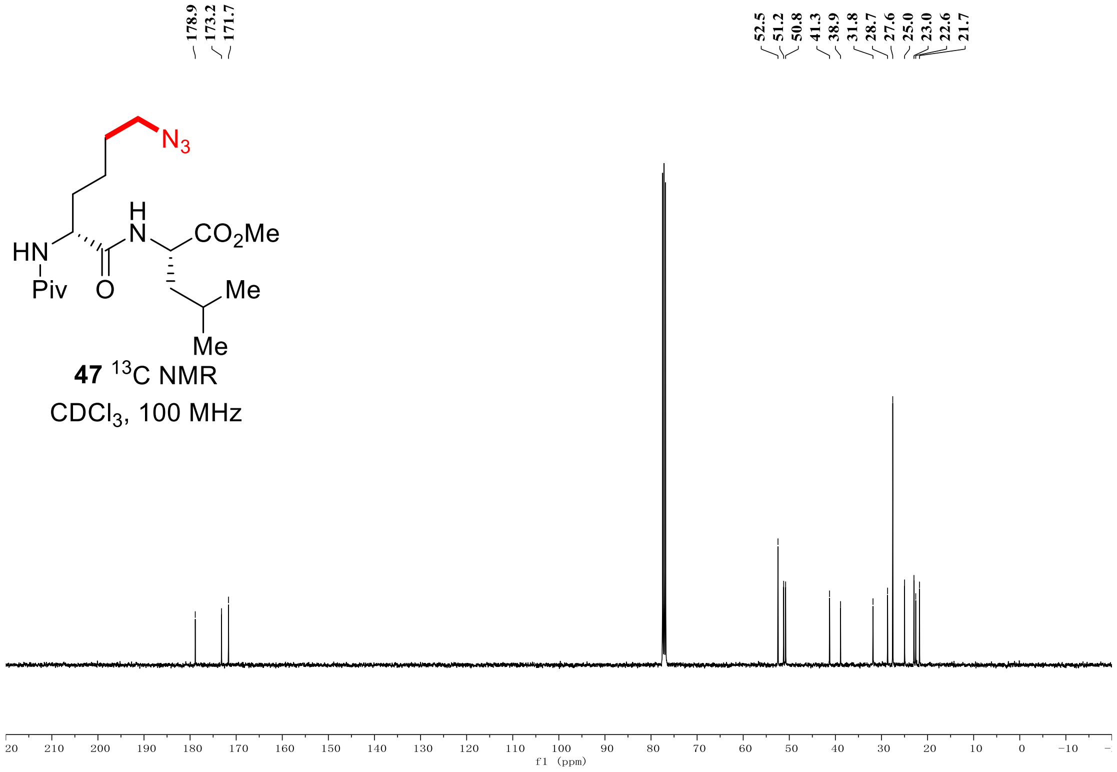
178.9
173.2
171.7
52.5
51.2
50.8
41.3
38.9
31.8
28.7
27.6
25.0
23.0
22.6
21.7
N3
H
N
CO2Me
HN
Piv
O
Me
Me
47 13C NMR
CDCl3, 100 MHz
f1 (ppm)

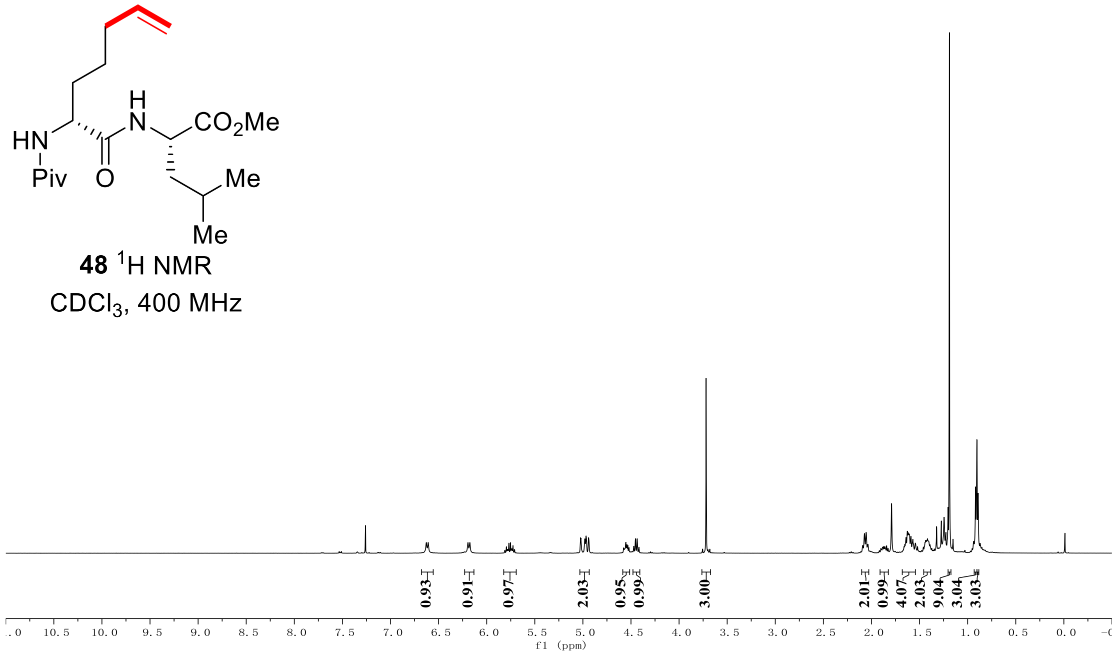
HN
Piv
O
H
N
CO2Me
Me
Me
48 1H NMR
CDCl3, 400 MHz
0.93
0.91
0.97
2.03
0.95
0.99
3.00
2.01
0.99
4.07
2.03
9.04
3.04
3.03
f1 (ppm)

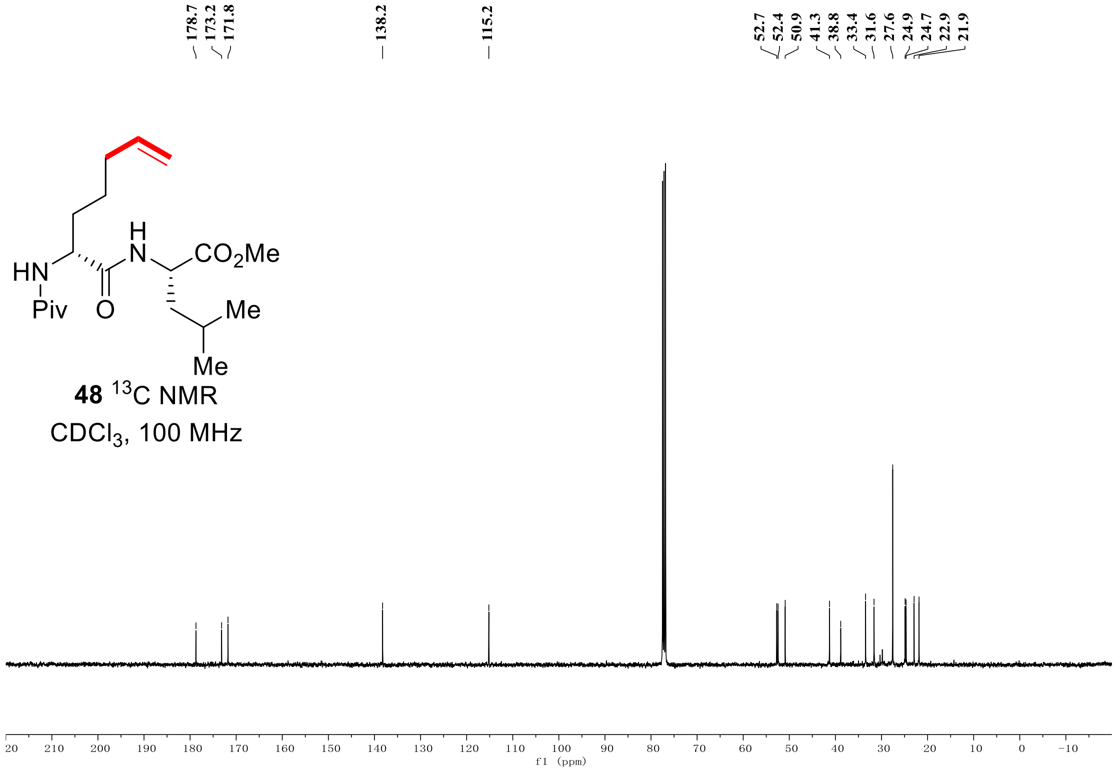
178.7
173.2
171.8
138.2
115.2
52.7
52.4
50.9
41.3
38.8
33.4
31.6
27.6
24.9
24.7
22.9
21.9
HN
Piv
O
H
N
CO2Me
Me
Me
48 13C NMR
CDCl3, 100 MHz
f1 (ppm)

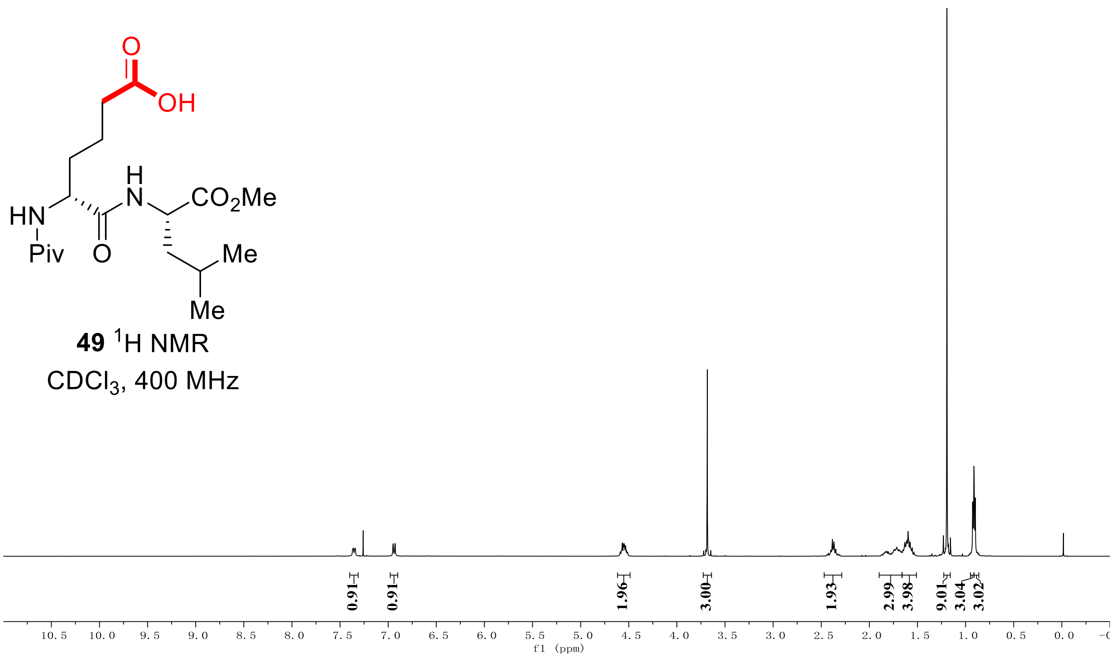

49 ^{1}H NMR

$CDCl_3$, 400 MHz

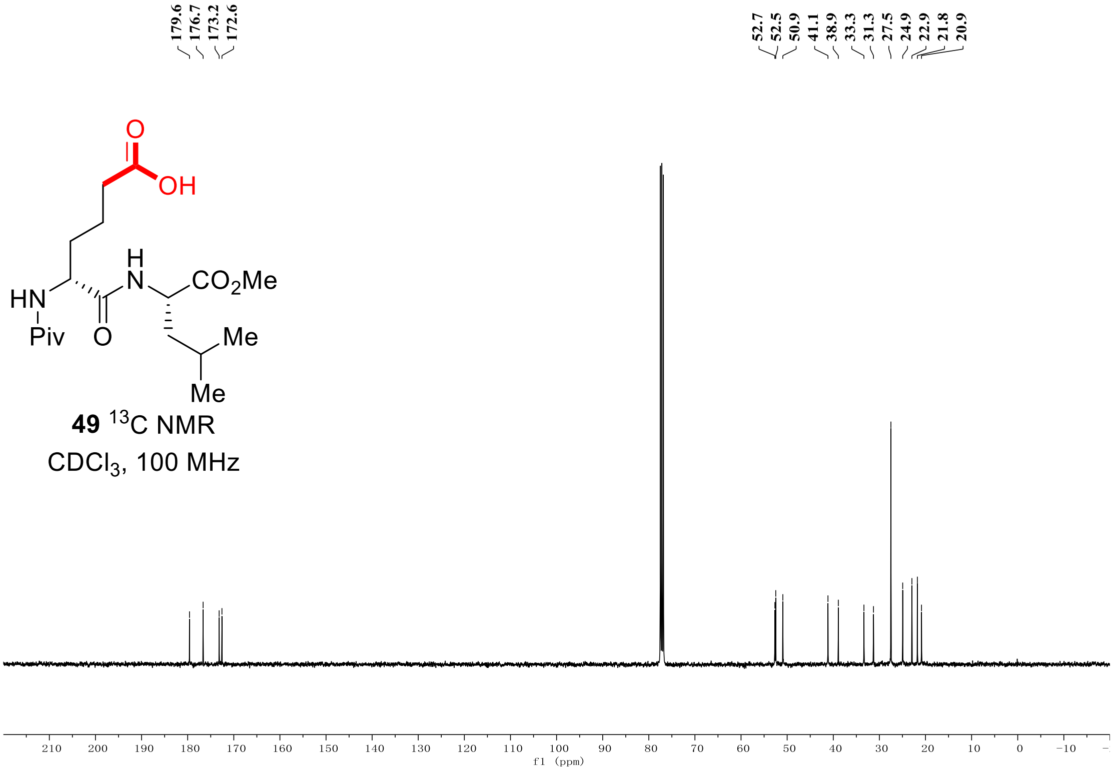

49 ^{13}C NMR

$CDCl_3$, 100 MHz

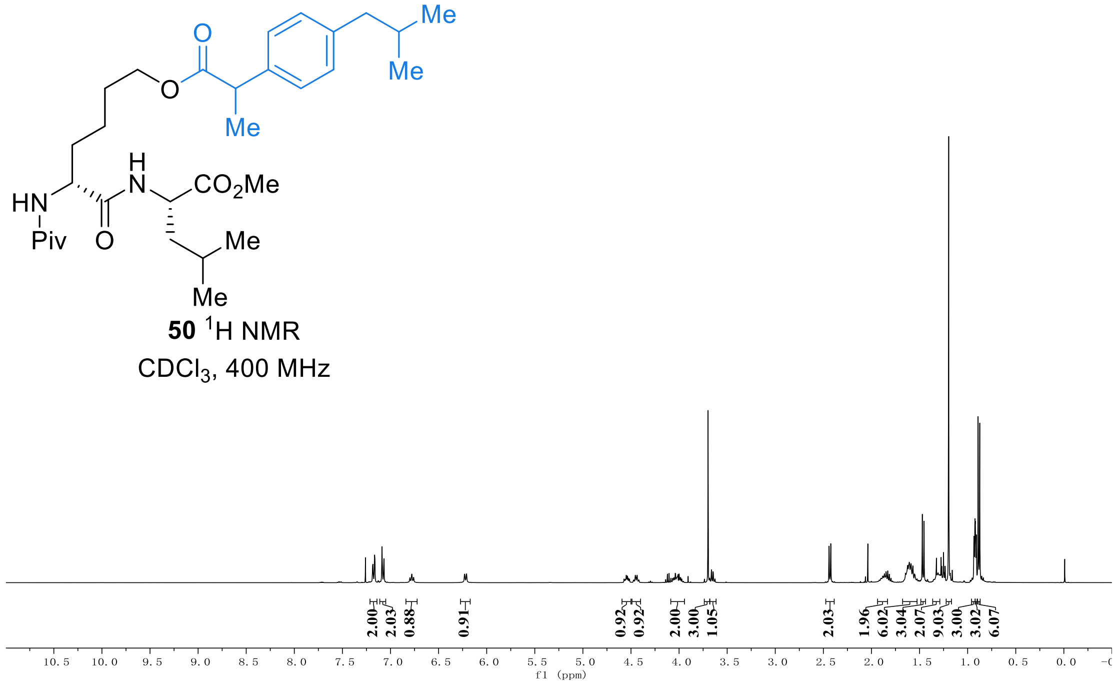

50 ^{1}H NMR

$CDCl_3$, 400 MHz

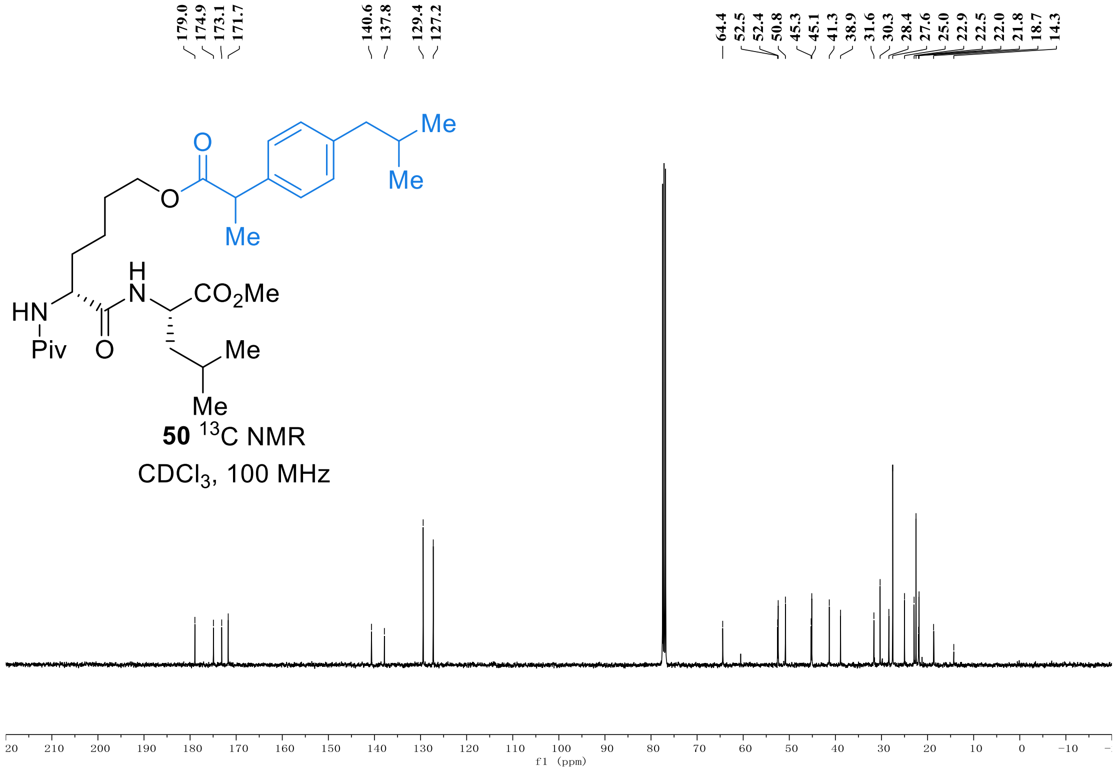

50 ^{13}C NMR

$CDCl_3$, 100 MHz

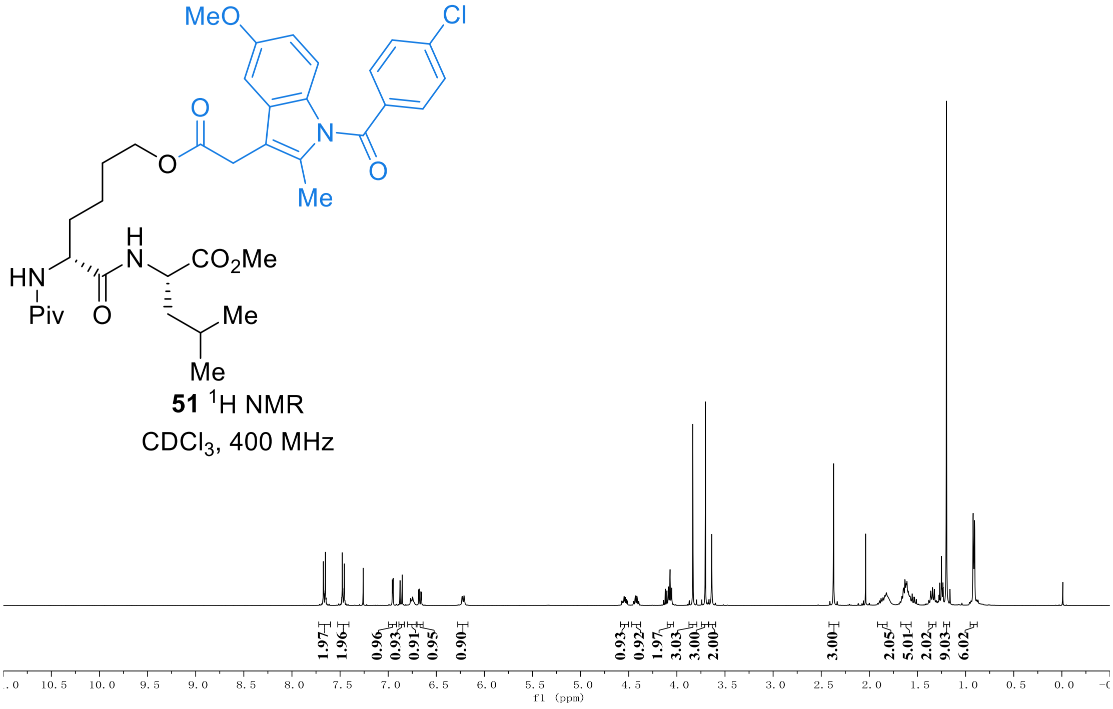
MeO
Cl
O
N
O
O
Me
H
N
CO$_2$Me
HN
Piv
O
Me
Me
51 ^{1}H NMR
CDCl$_3$, 400 MHz
1.97
1.96
0.96
0.93
0.91
0.95
0.90
0.93
0.92
1.97
3.03
3.00
2.00
3.00
2.05
5.01
2.02
9.03
6.02
f1 (ppm)

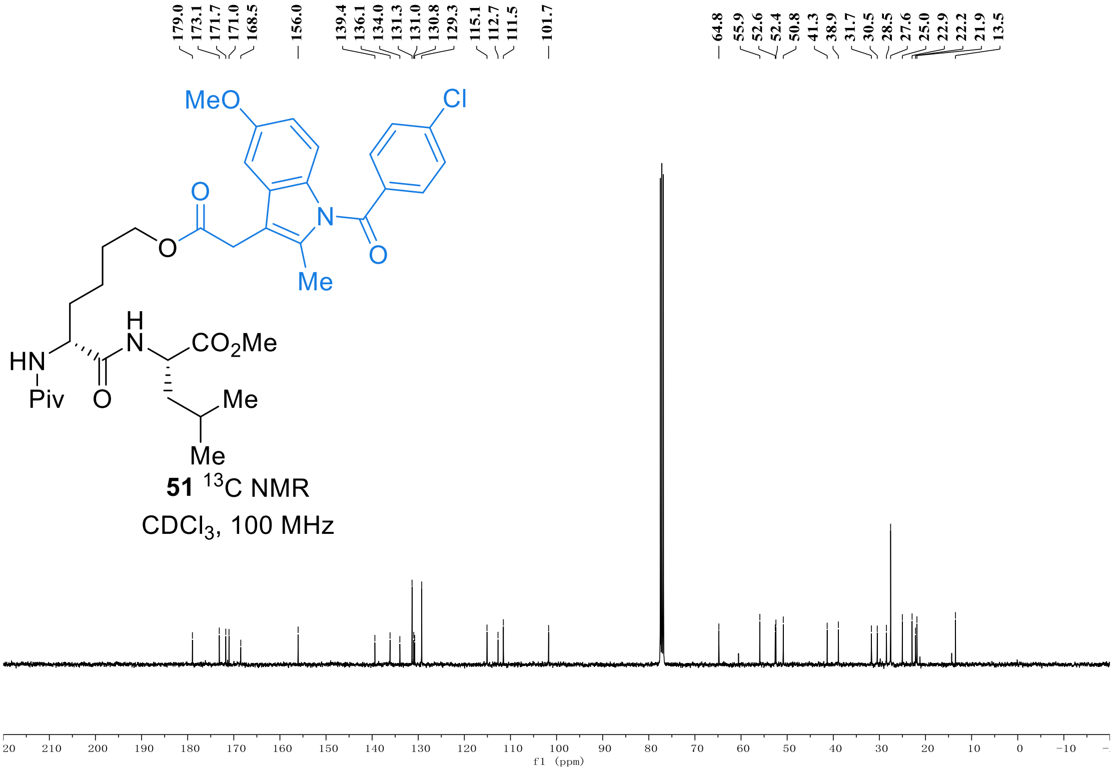
179.0
173.1
171.7
171.0
168.5
156.0
139.4
136.1
134.0
131.3
131.0
130.8
129.3
115.1
112.7
111.5
101.7
64.8
55.9
52.6
52.4
50.8
41.3
38.9
31.7
30.5
28.5
27.6
25.0
22.9
22.2
21.9
13.5
MeO
Cl
O
N
O
O
Me
H
N
CO$_2$Me
HN
Piv
O
Me
Me
51 ^{13}C NMR
CDCl$_3$, 100 MHz
f1 (ppm)

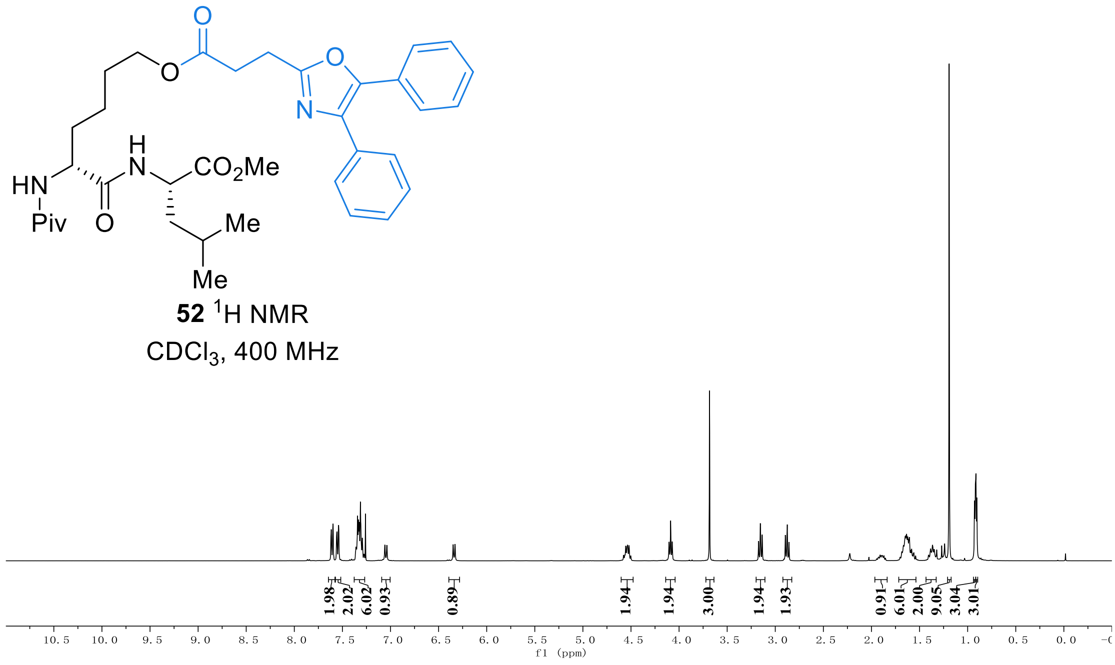
O
O
O
N
H
N
CO2Me
HN
Piv
O
Me
Me
52 1H NMR
CDCl3, 400 MHz
1.98
2.02
6.02
0.93
0.89
1.94
1.94
3.00
1.94
1.93
0.91
6.01
2.00
9.05
3.04
3.01
10.5
10.0
9.5
9.0
8.5
8.0
7.5
7.0
6.5
6.0
5.5
5.0
4.5
4.0
3.5
3.0
2.5
2.0
1.5
1.0
0.5
0.0
f1 (ppm)

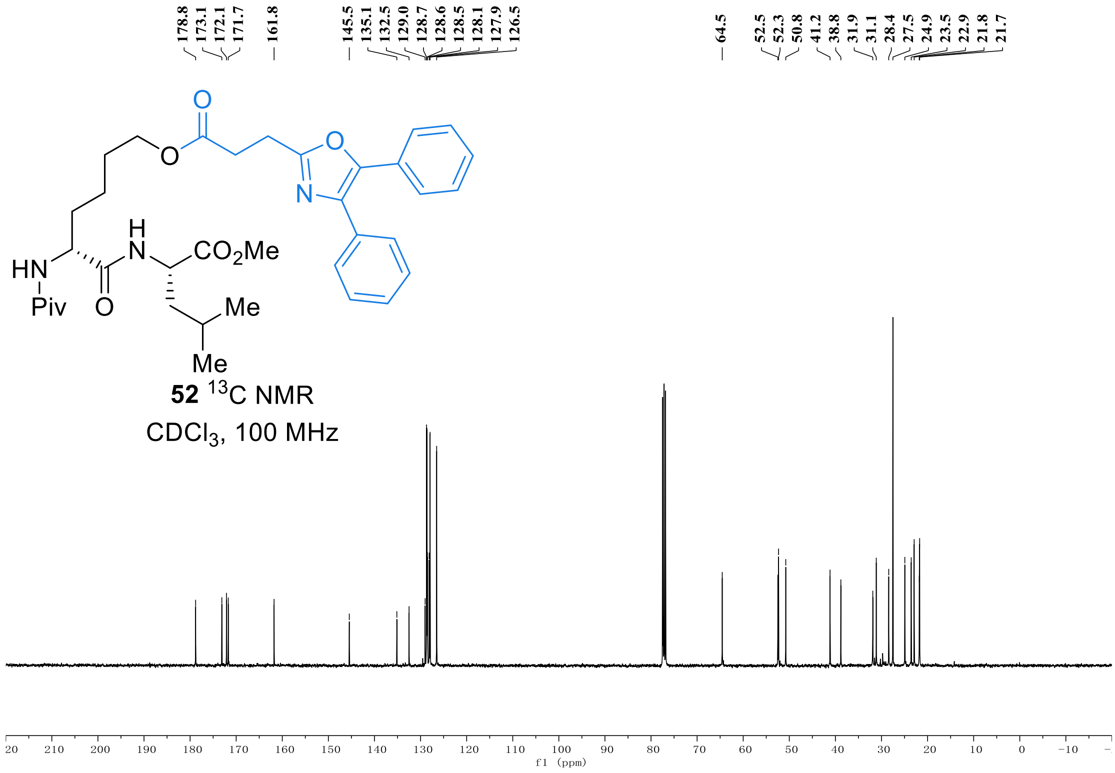
178.8
173.1
172.1
171.7
161.8
145.5
135.1
132.5
129.0
128.7
128.6
128.5
128.1
127.9
126.5
64.5
52.5
52.3
50.8
41.2
38.8
31.9
31.1
28.4
27.5
24.9
23.5
22.9
21.8
21.7
52 13C NMR
CDCl3, 100 MHz
210
200
190
180
170
160
150
140
130
120
110
100
90
80
70
60
50
40
30
20
10
0
-10
f1 (ppm)

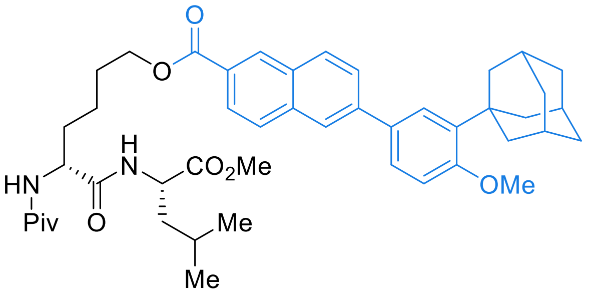

53 ^{1}H NMR

$CDCl_3$, 400 MHz

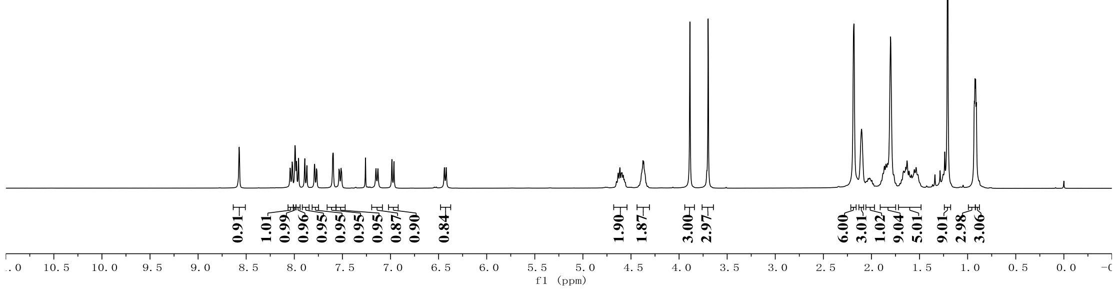

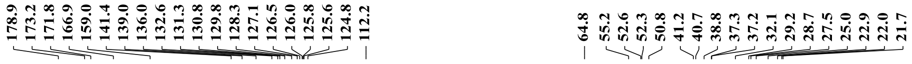

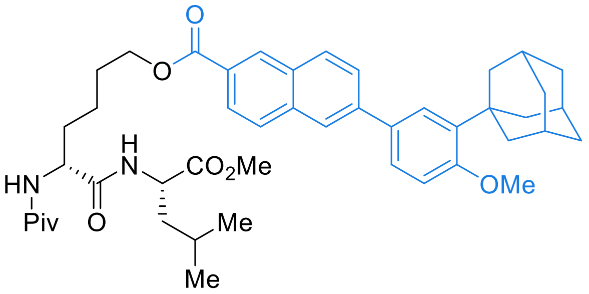

53 ^{13}C NMR

$CDCl_3$, 100 MHz

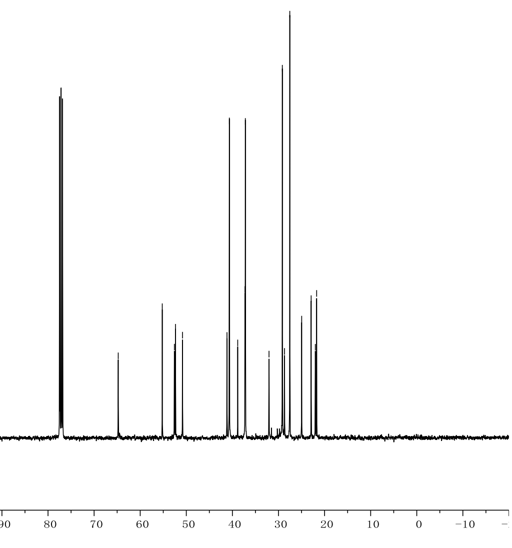

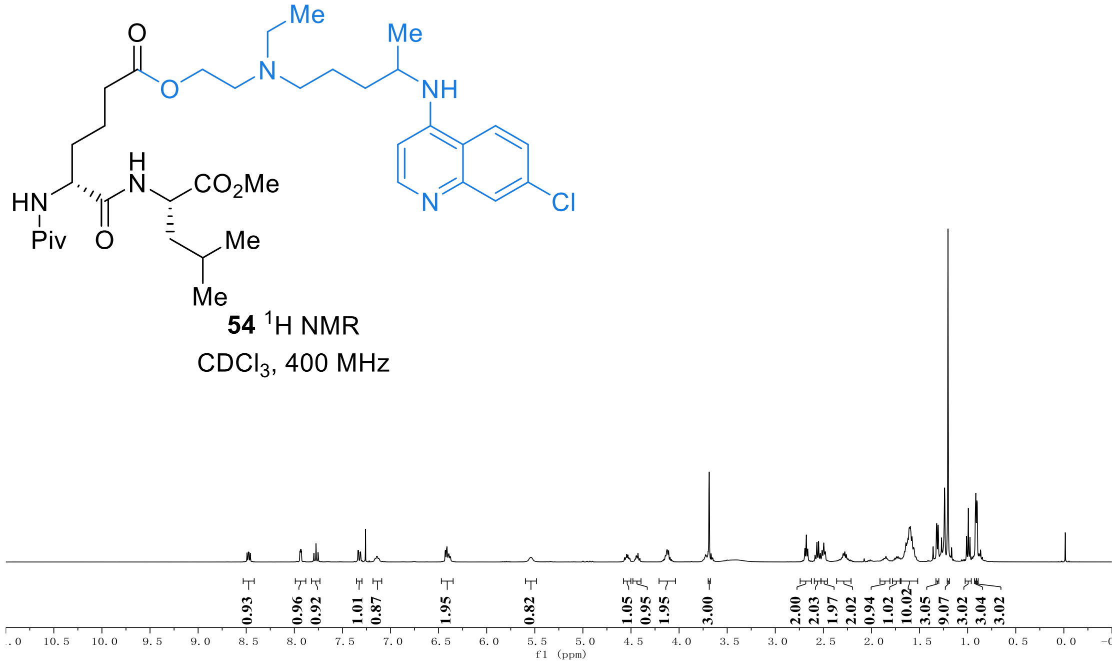

54 ^{1}H NMR
$CDCl_3$, 400 MHz

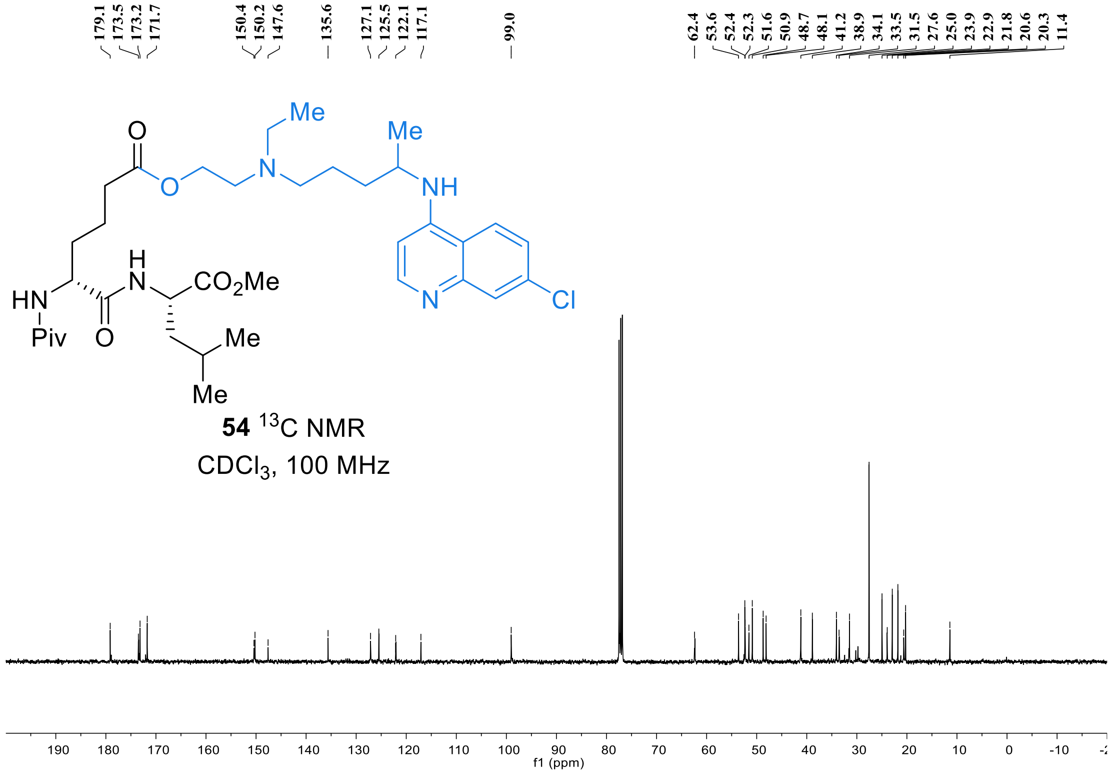

54 ^{13}C NMR
$CDCl_3$, 100 MHz

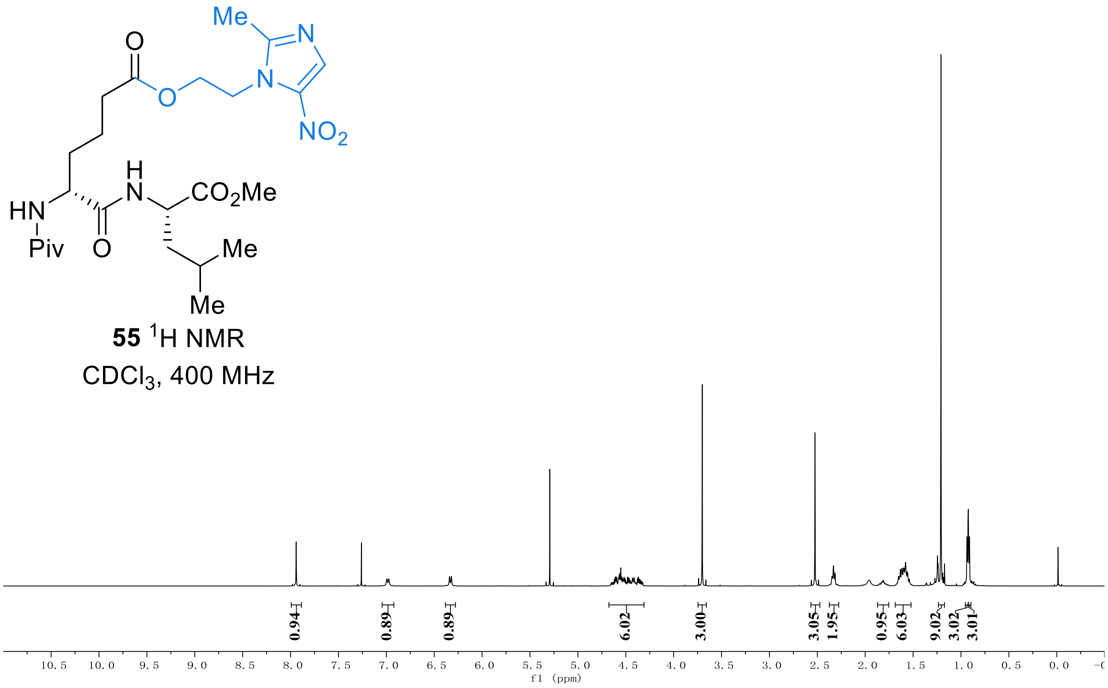

Me
N
O
O
N
NO2
H
N
CO2Me
HN
Piv
O
Me
Me
55 1H NMR
CDCl3, 400 MHz
0.94
0.89
0.89
6.02
3.00
3.05
1.95
0.95
6.03
9.02
3.02
3.01
f1 (ppm)

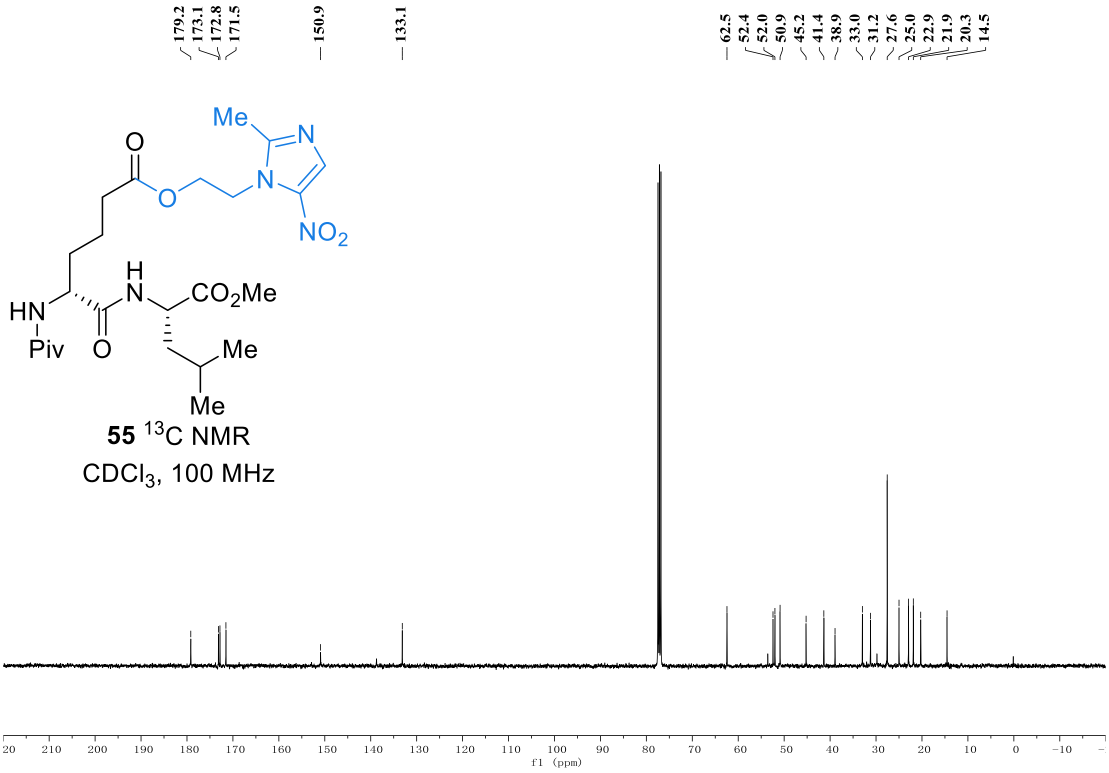

179.2
173.1
172.8
171.5
150.9
133.1
62.5
52.4
52.0
50.9
45.2
41.4
38.9
33.0
31.2
27.6
25.0
22.9
21.9
20.3
14.5
Me
N
O
O
N
NO2
H
N
CO2Me
HN
Piv
O
Me
Me
55 13C NMR
CDCl3, 100 MHz
f1 (ppm)

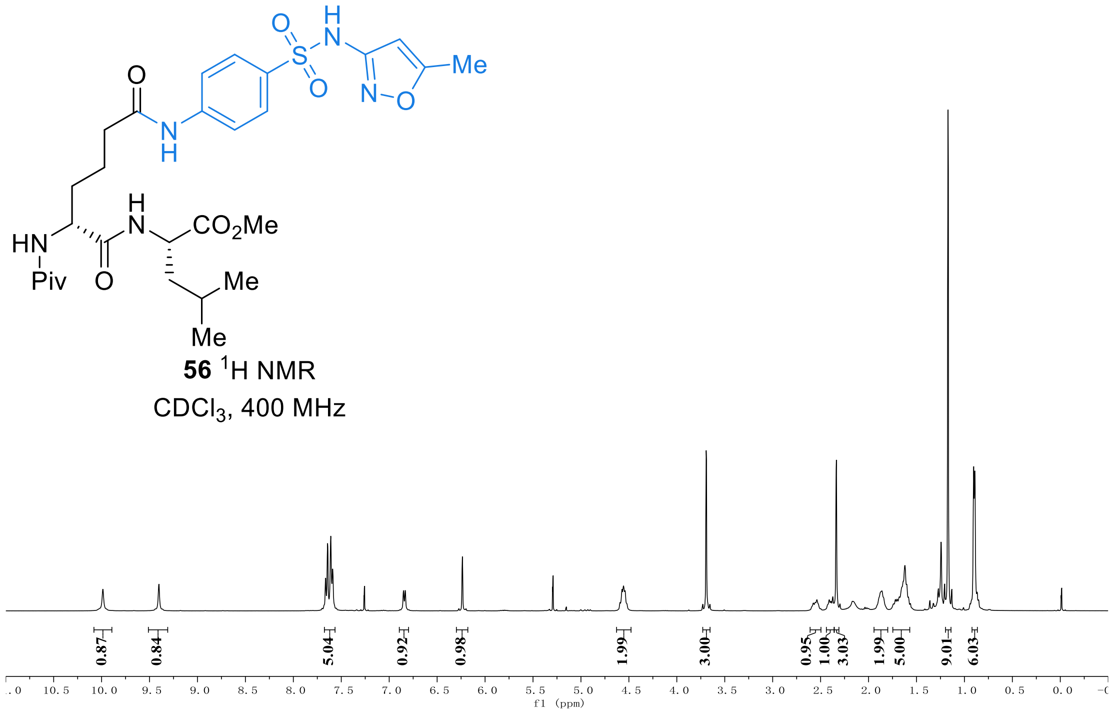

56 ^{1}H NMR

$CDCl_3$, 400 MHz

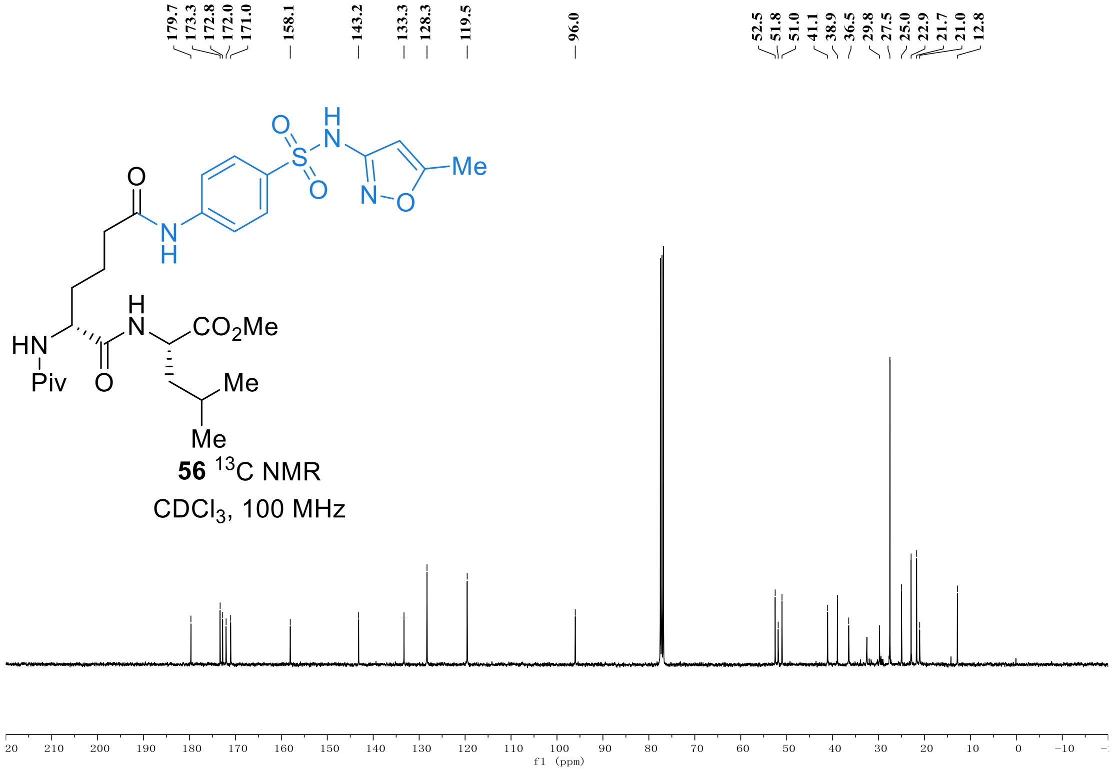

56 ^{13}C NMR

$CDCl_3$, 100 MHz

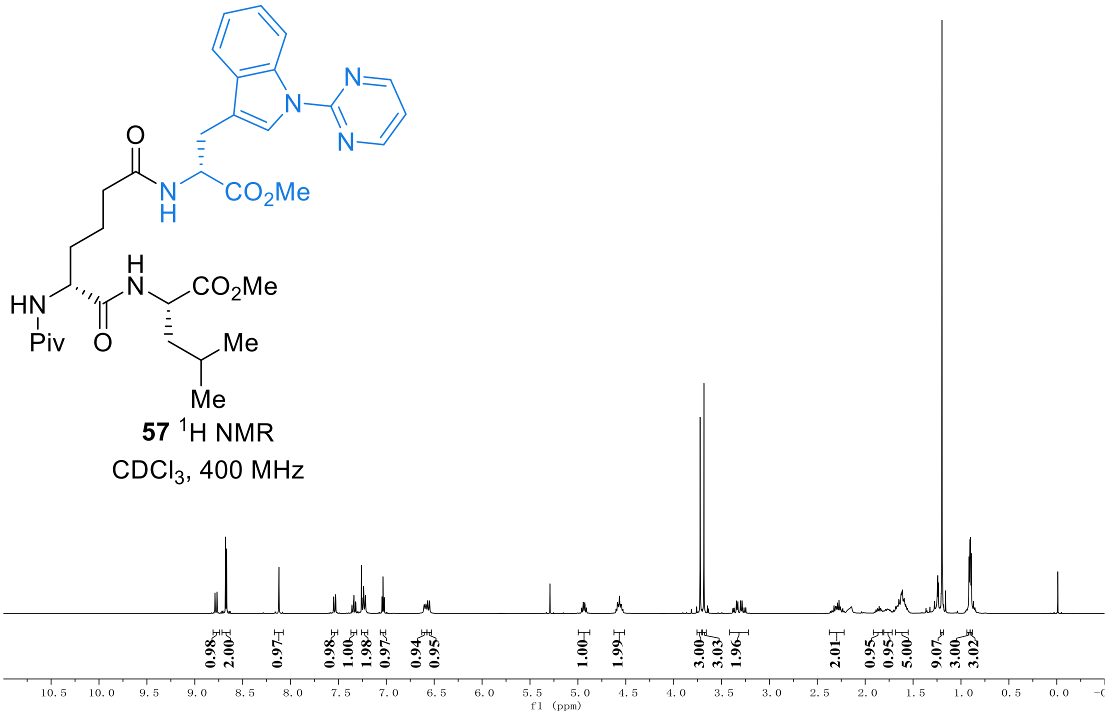

57 ^{1}H NMR

$CDCl_3$, 400 MHz

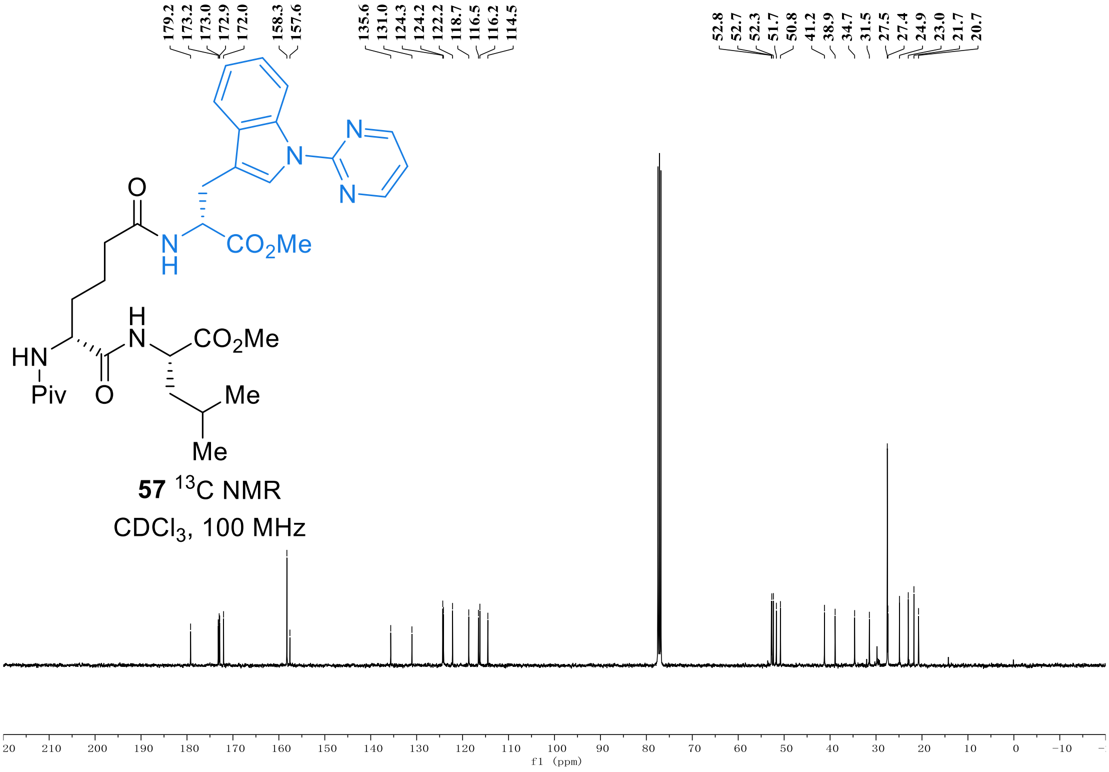

57 ^{13}C NMR

$CDCl_3$, 100 MHz

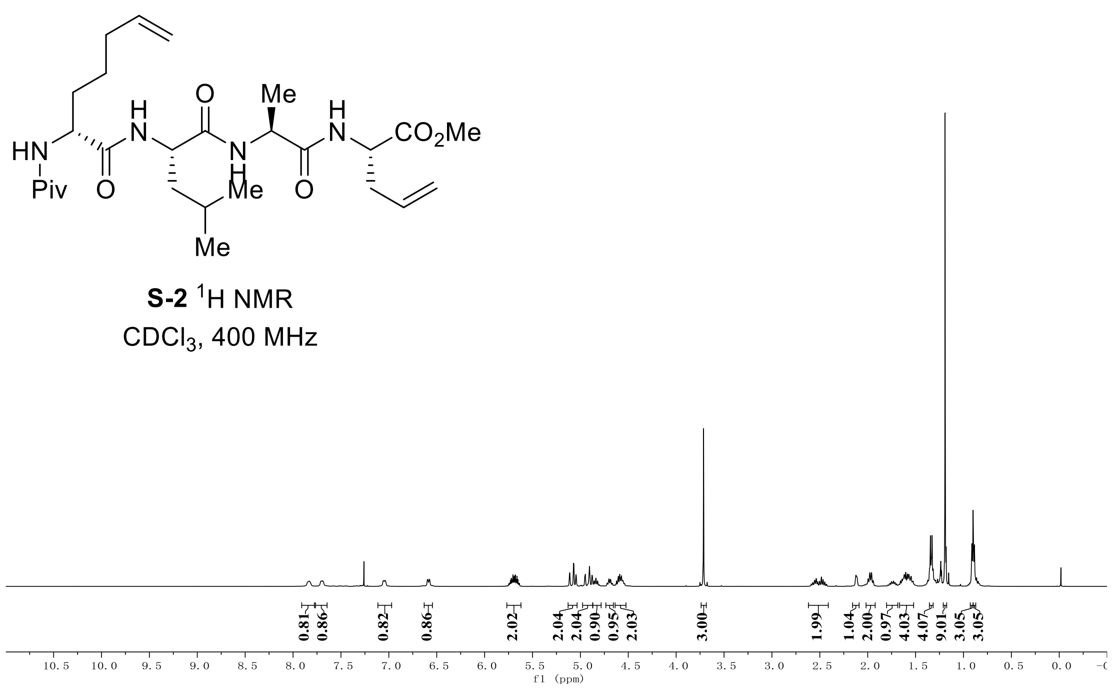
HN
Piv
O
H
N
Me
O
N
H
Me
O
H
N
CO2Me
Me
S-2 1H NMR
CDCl3, 400 MHz
0.81
0.86
0.82
0.86
2.02
2.04
2.04
0.90
0.95
2.03
3.00
1.99
1.04
2.00
0.97
4.03
4.07
9.01
3.05
3.05
f1 (ppm)

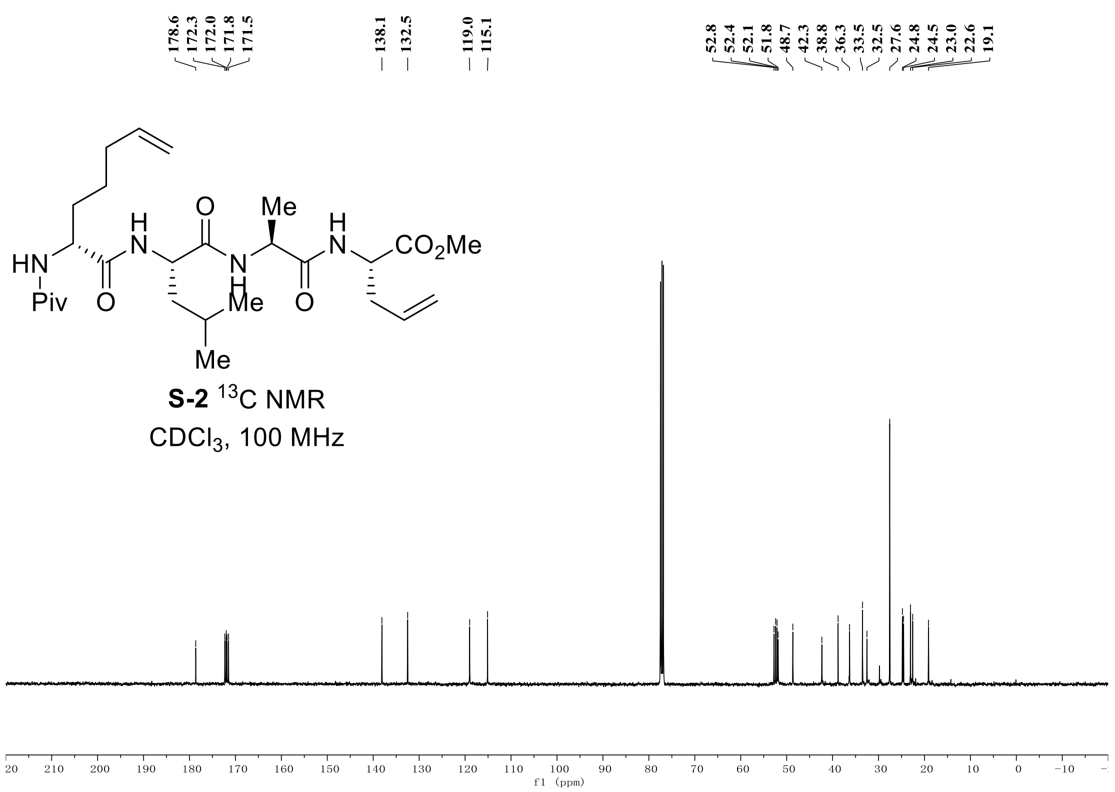
178.6
172.3
172.0
171.8
171.5
138.1
132.5
119.0
115.1
52.8
52.4
52.1
51.8
48.7
42.3
38.8
36.3
33.5
32.5
27.6
24.8
24.5
23.0
22.6
19.1
S-2 13C NMR
CDCl3, 100 MHz
f1 (ppm)

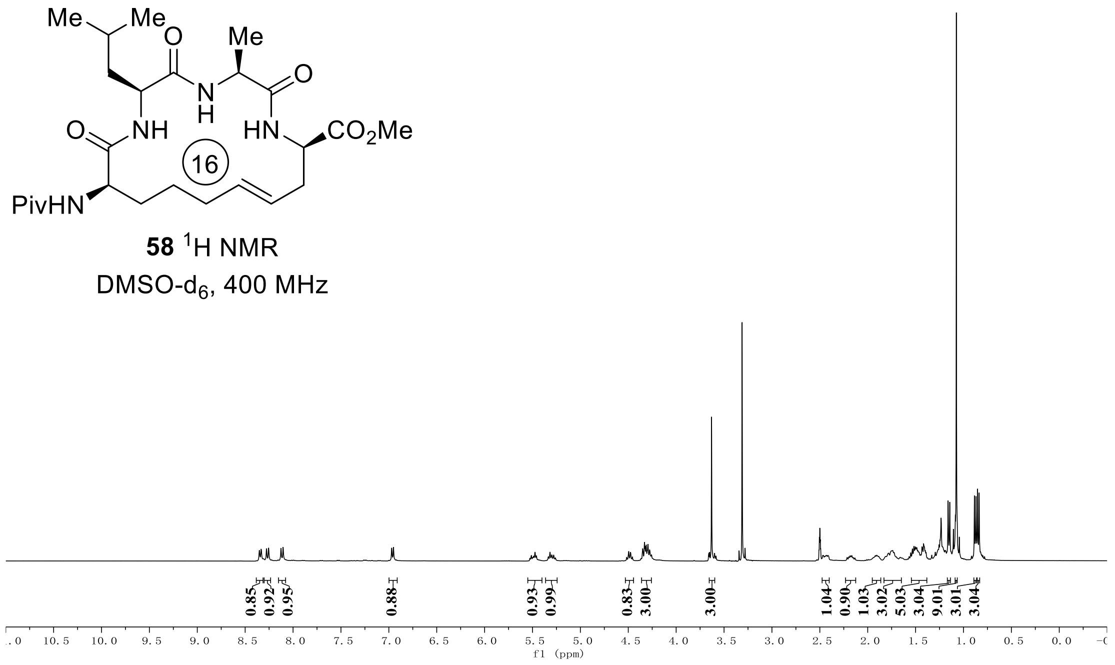

58 ^{1}H NMR
DMSO-d$_6$, 400 MHz
0.85 0.92 0.95 0.88 0.93 0.99 0.83 3.00 3.00 1.04 0.90 1.03 3.02 5.03 3.04 9.01 3.01 3.04
f1 (ppm)

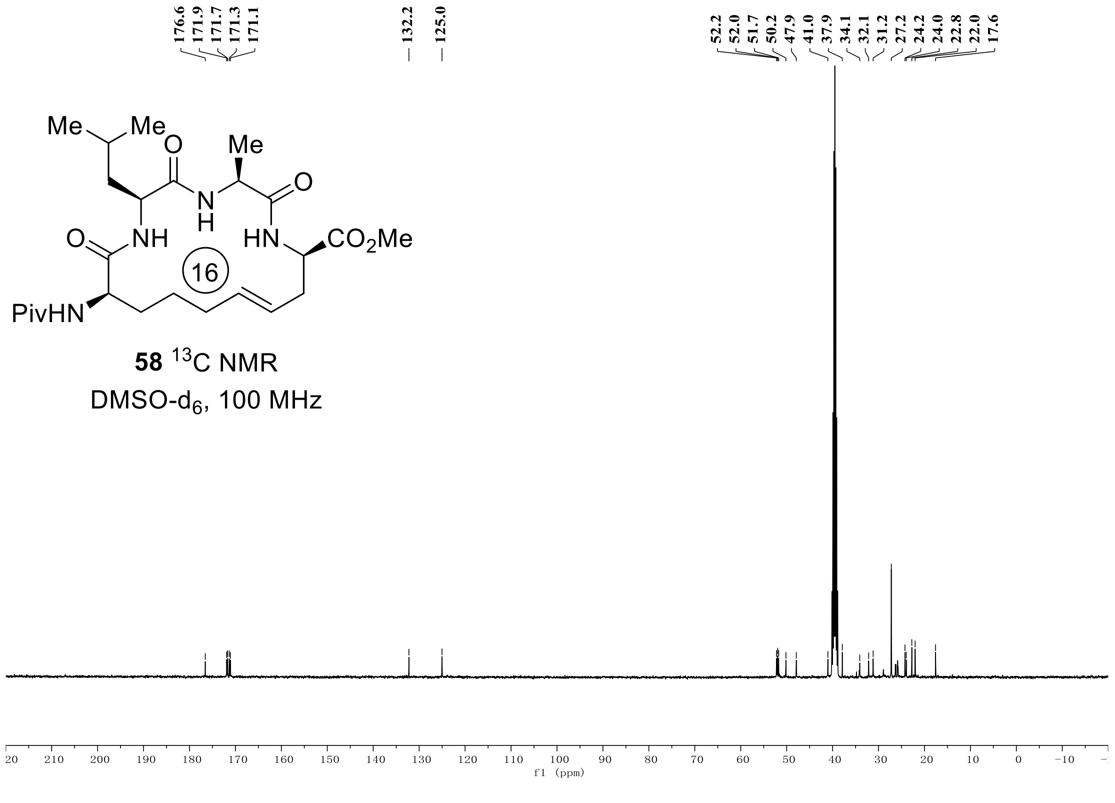

176.6 171.9 171.7 171.3 171.1
132.2 125.0
52.2 52.0 51.7 50.2 47.9 41.0 37.9 34.1 32.1 31.2 27.2 24.2 24.0 22.8 22.0 17.6
58 ^{13}C NMR
DMSO-d$_6$, 100 MHz
f1 (ppm)

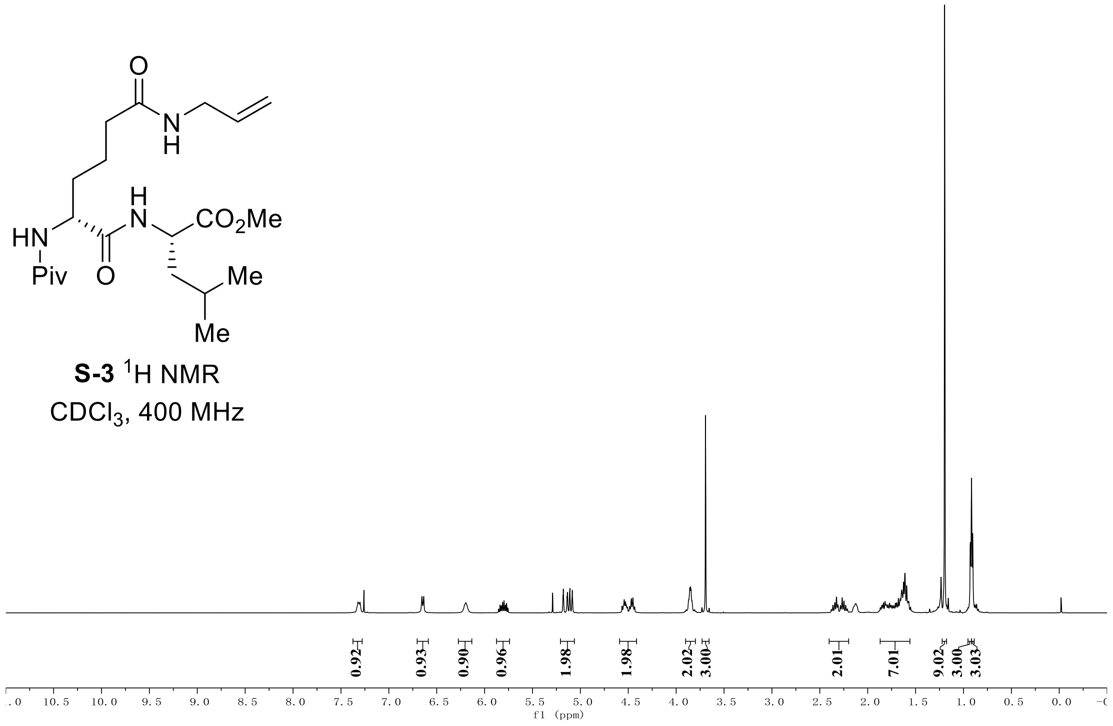

S-3 ^{1}H NMR
$CDCl_3$, 400 MHz

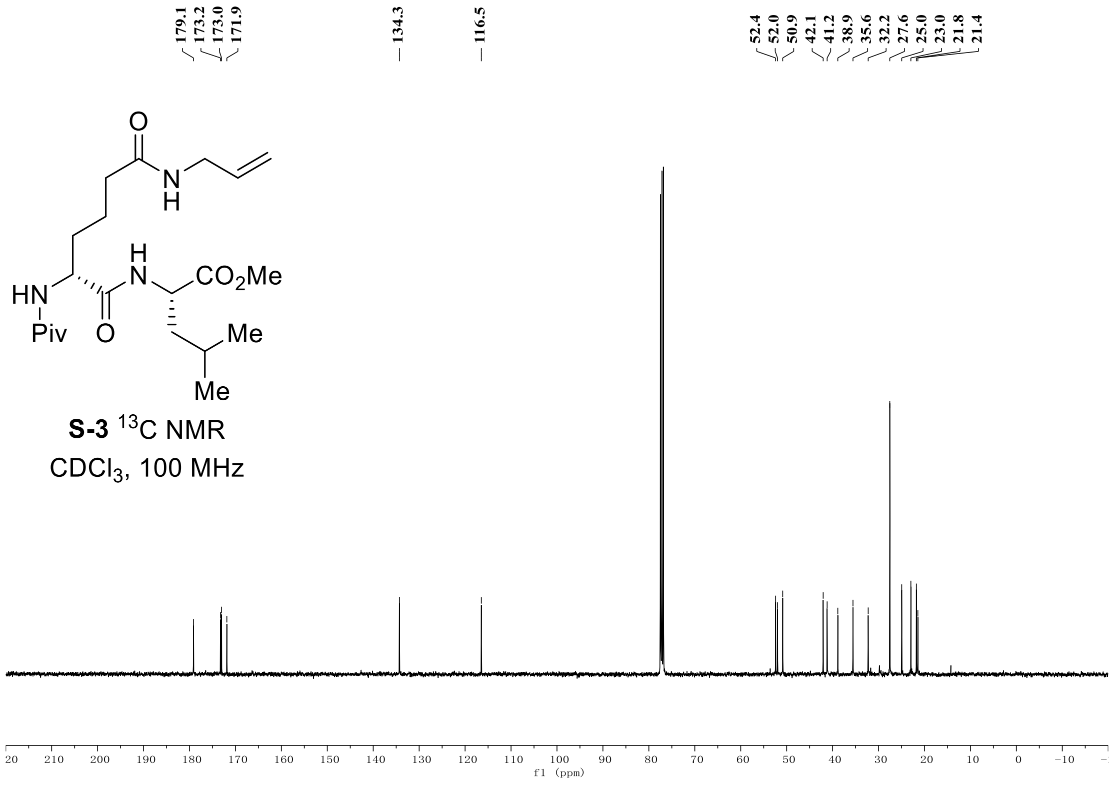

S-3 ^{13}C NMR
$CDCl_3$, 100 MHz

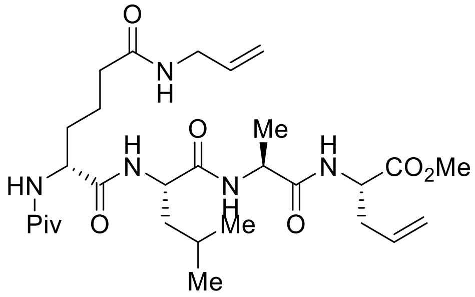

S-4 ^{1}H NMR
$CDCl_3$, 400 MHz

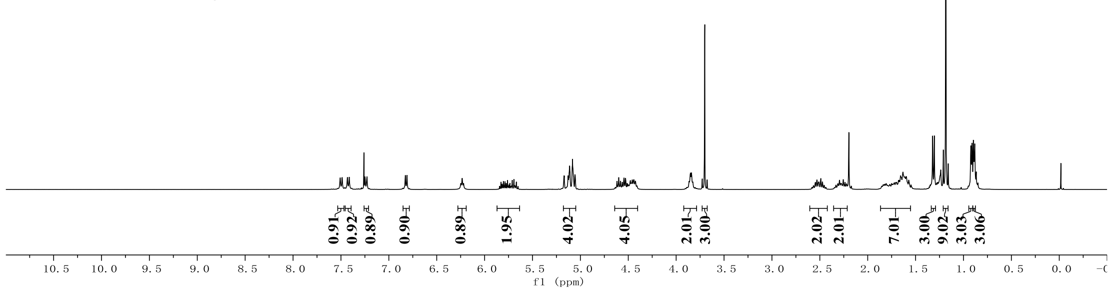

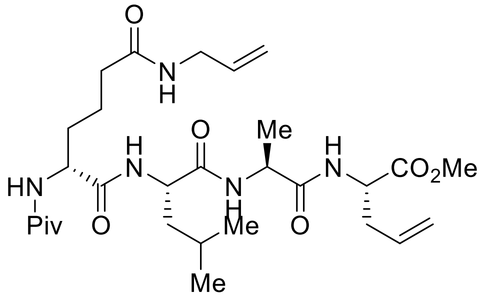

S-4 ^{13}C NMR
$CDCl_3$, 100 MHz

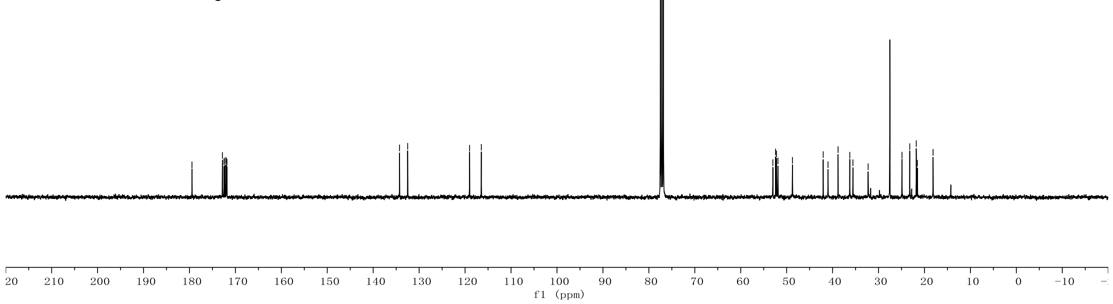

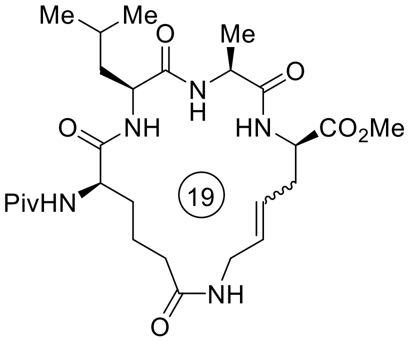

59 ^{1}H NMR
DMSO-d_6, 400 MHz

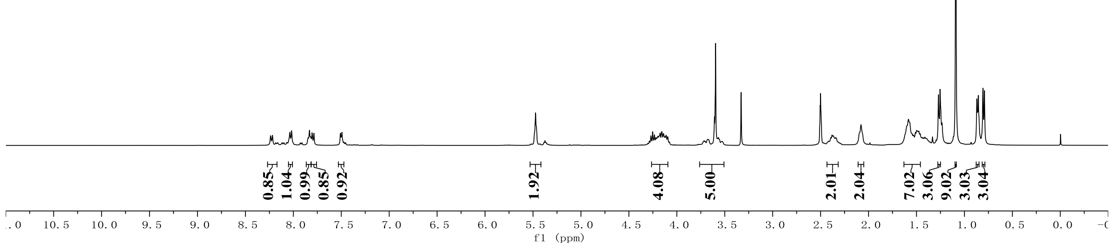

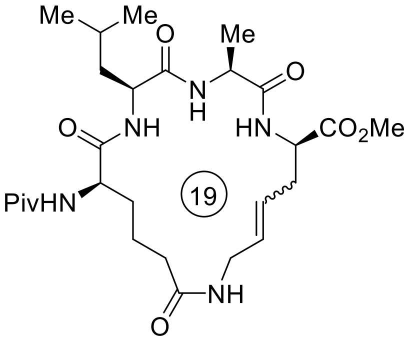

59 ^{13}C NMR
DMSO-d_6, 100 MHz

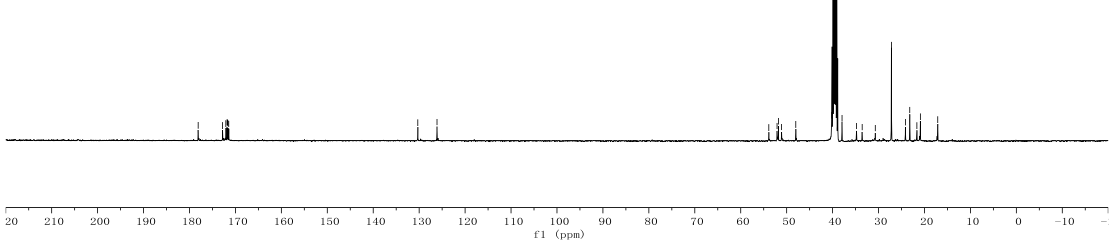

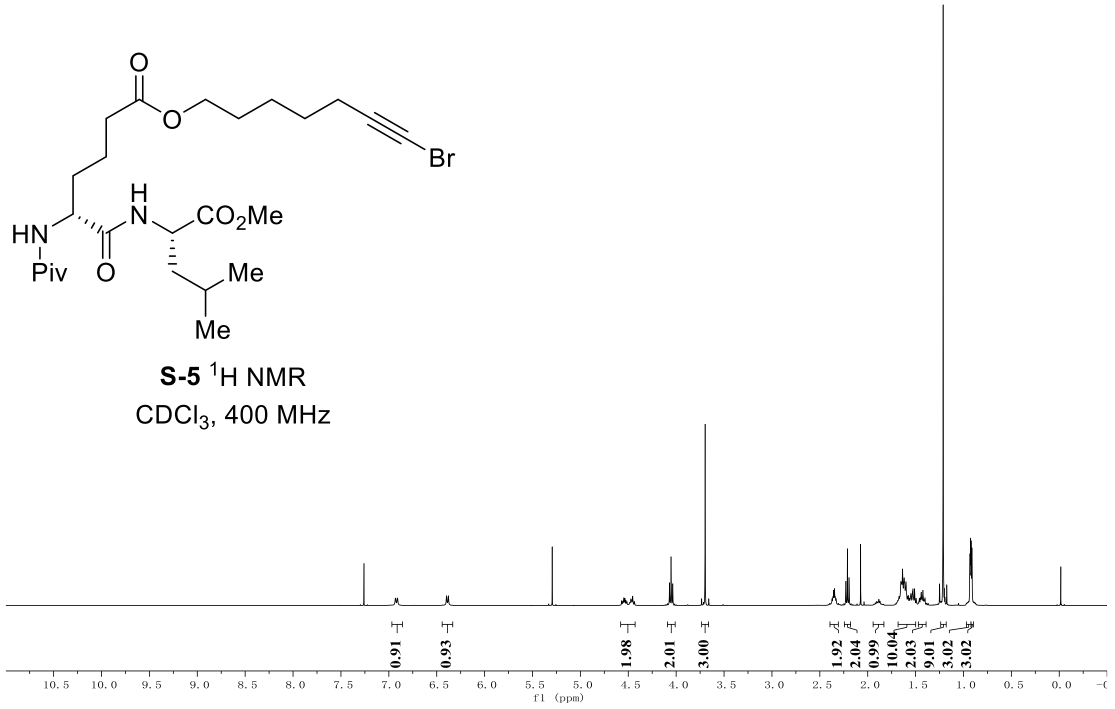
O
O
Br
H
N
HN
CO2Me
Piv
O
Me
Me
S-5 1H NMR
CDCl3, 400 MHz
0.91
0.93
1.98
2.01
3.00
1.92
2.04
0.99
10.04
2.03
9.01
3.02
3.02
f1 (ppm)

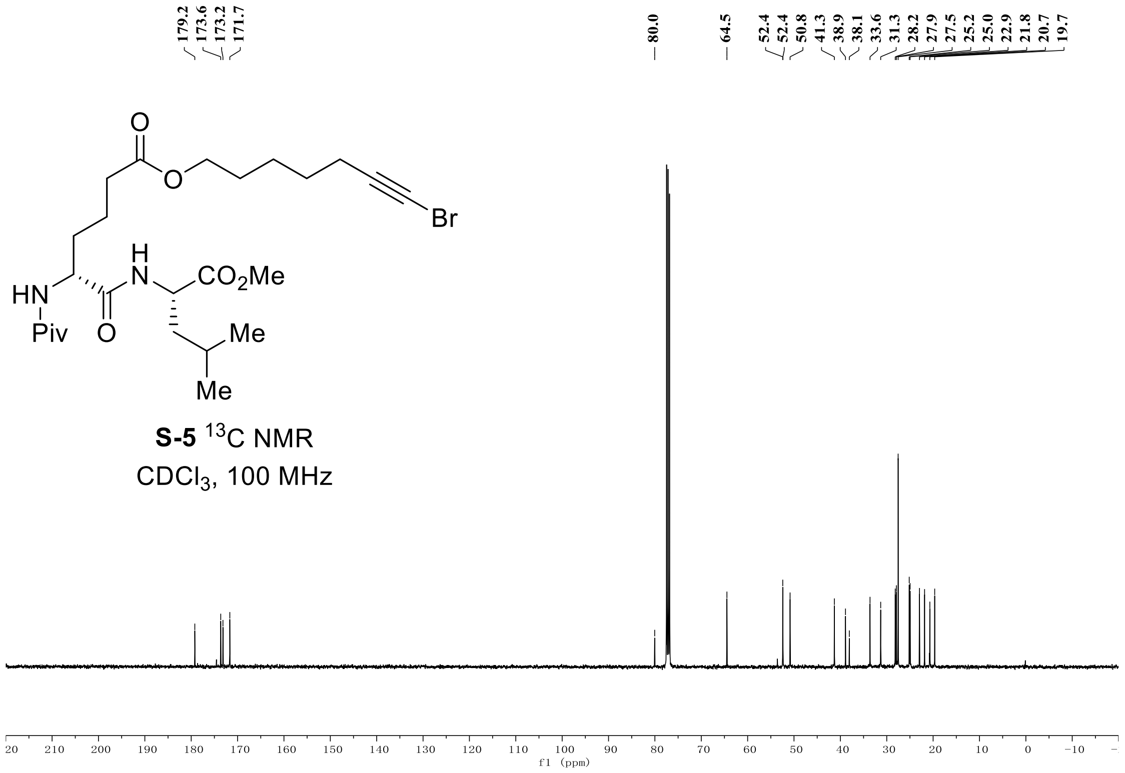
179.2
173.6
173.2
171.7
80.0
64.5
52.4
52.4
50.8
41.3
38.9
38.1
33.6
31.3
28.2
27.9
27.5
25.2
25.0
22.9
21.8
20.7
19.7
O
O
Br
H
N
HN
CO2Me
Piv
O
Me
Me
S-5 13C NMR
CDCl3, 100 MHz
f1 (ppm)

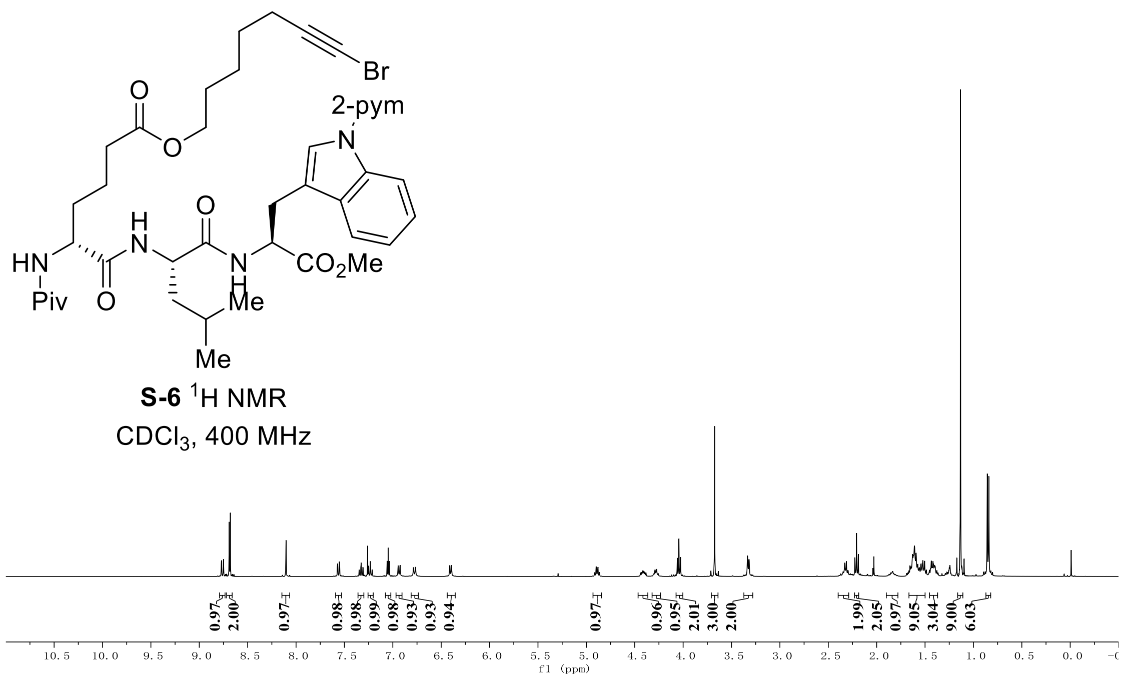
Br
2-pym
N
O
O
H
N
O
HN
N
CO2Me
H
Piv
O
Me
Me
S-6 1H NMR
CDCl3, 400 MHz
0.97
2.00
0.97
0.98
0.98
0.99
0.98
0.93
0.93
0.94
0.97
0.96
0.95
2.01
3.00
2.00
1.99
2.05
0.97
9.05
3.04
9.00
6.03
10.5 10.0 9.5 9.0 8.5 8.0 7.5 7.0 6.5 6.0 5.5 5.0 4.5 4.0 3.5 3.0 2.5 2.0 1.5 1.0 0.5 0.0
f1 (ppm)

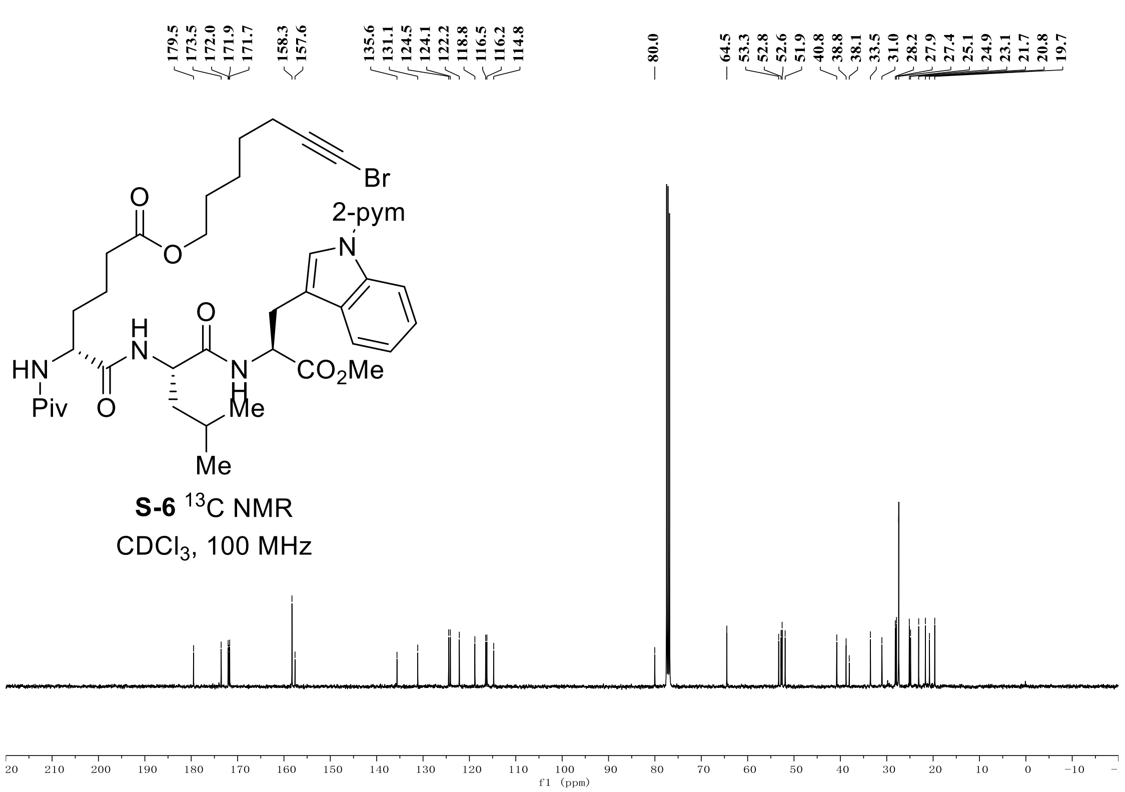
179.5
173.5
172.0
171.9
171.7
158.3
157.6
135.6
131.1
124.5
124.1
122.2
118.8
116.5
116.2
114.8
80.0
64.5
53.3
52.8
52.6
51.9
40.8
38.8
38.1
33.5
31.0
28.2
27.9
27.4
25.1
24.9
23.1
21.7
20.8
19.7
Br
2-pym
N
O
O
H
N
O
HN
N
CO2Me
H
Piv
O
Me
Me
S-6 13C NMR
CDCl3, 100 MHz
210 200 190 180 170 160 150 140 130 120 110 100 90 80 70 60 50 40 30 20 10 0 −10
f1 (ppm)

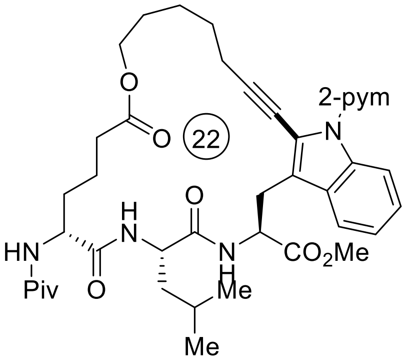

60 ^{1}H NMR

$CDCl_3$, 400 MHz

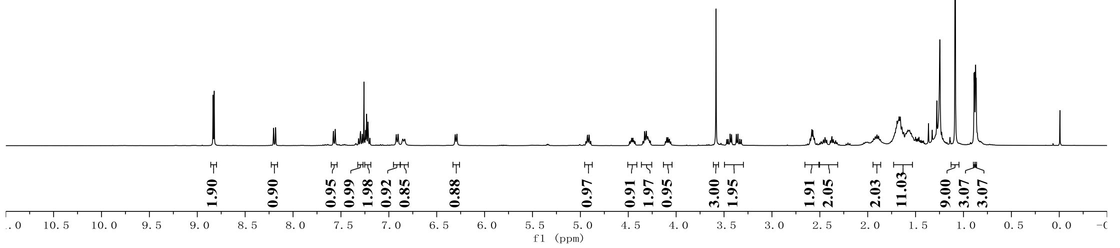

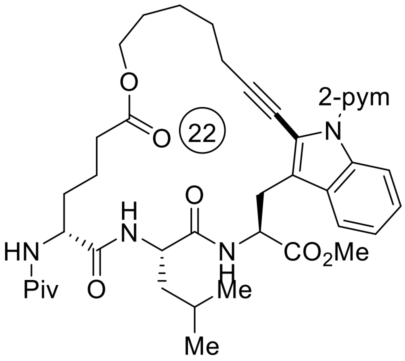

60 ^{13}C NMR

$CDCl_3$, 100 MHz

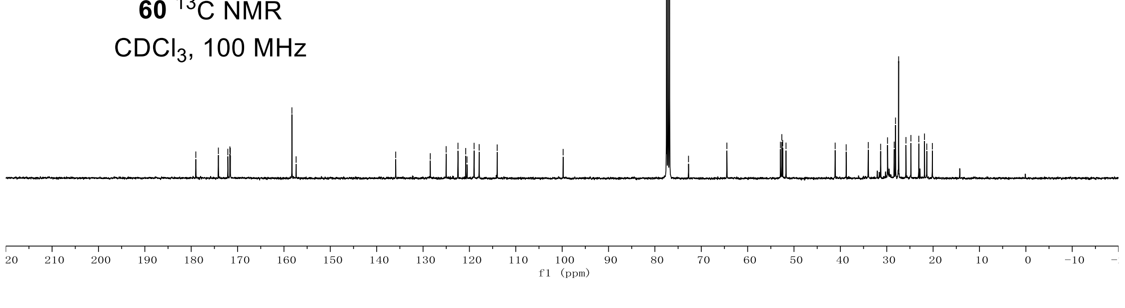

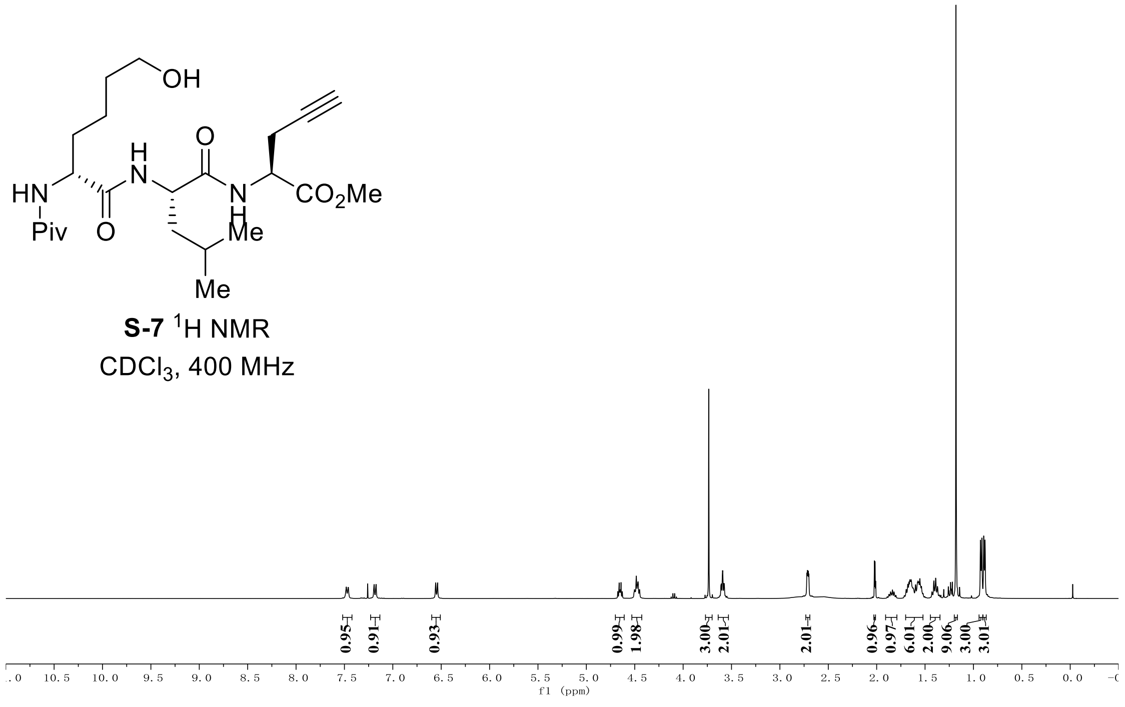

S-7 ^{1}H NMR
$CDCl_3$, 400 MHz

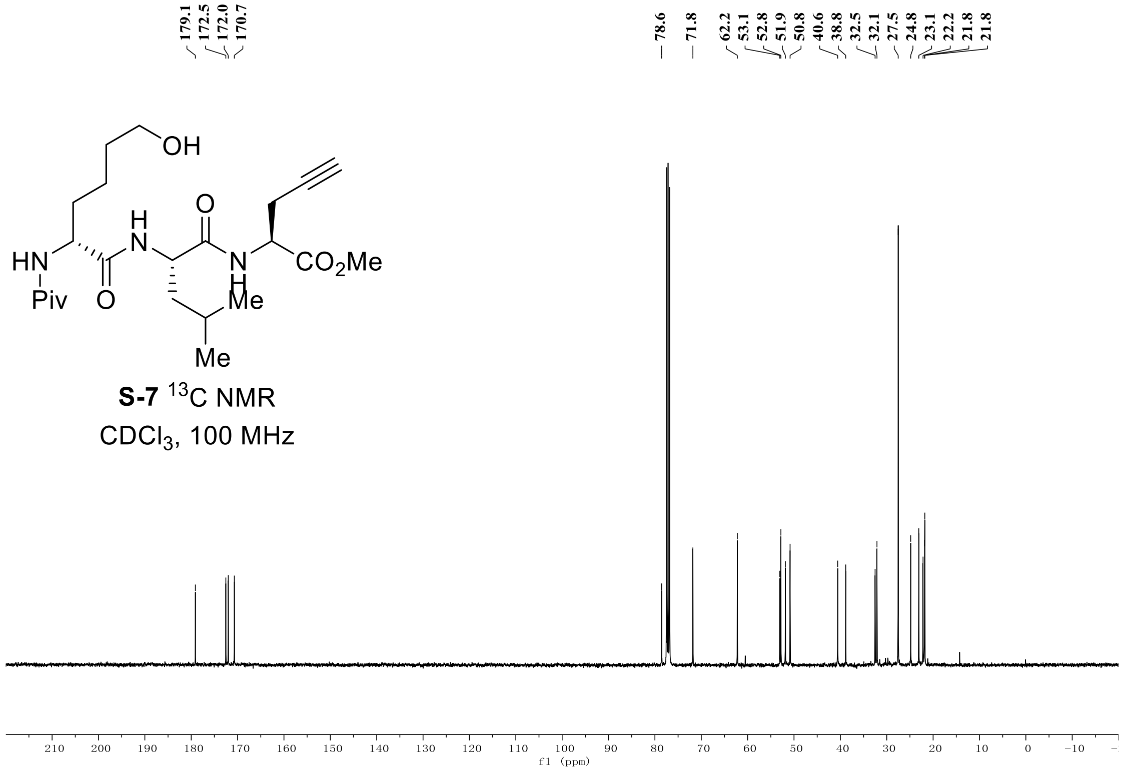

S-7 ^{13}C NMR
$CDCl_3$, 100 MHz

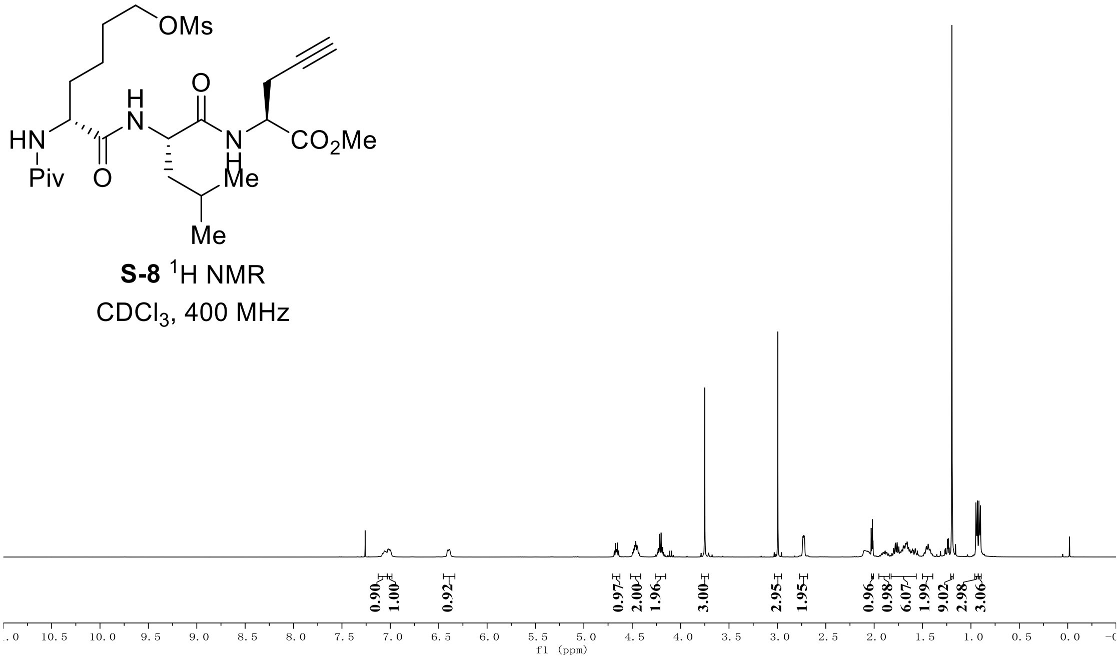
OMs
HN
Piv
CO2Me
Me
Me
S-8 1H NMR
CDCl3, 400 MHz
0.90
1.00
0.92
0.97
2.00
1.96
3.00
2.95
1.95
0.96
0.98
6.07
1.99
9.02
2.98
3.06
f1 (ppm)

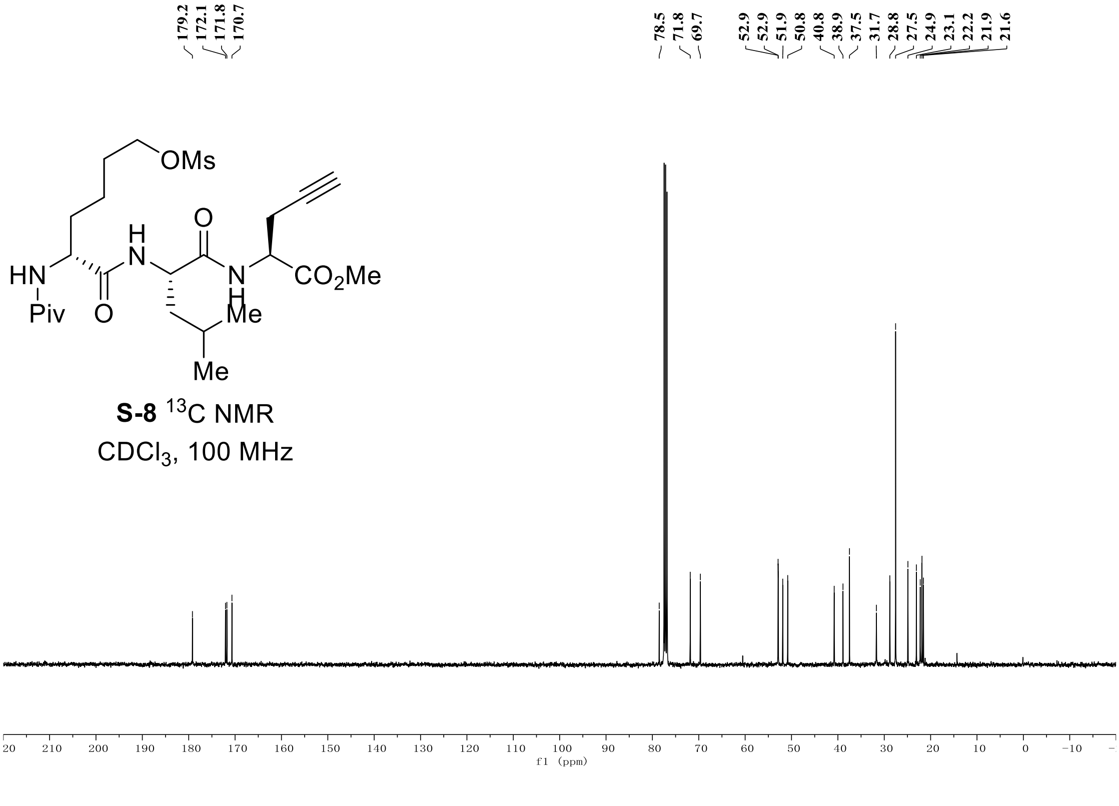
179.2
172.1
171.8
170.7
78.5
71.8
69.7
52.9
52.9
51.9
50.8
40.8
38.9
37.5
31.7
28.8
27.5
24.9
23.1
22.2
21.9
21.6
OMs
HN
Piv
CO2Me
Me
Me
S-8 13C NMR
CDCl3, 100 MHz
f1 (ppm)

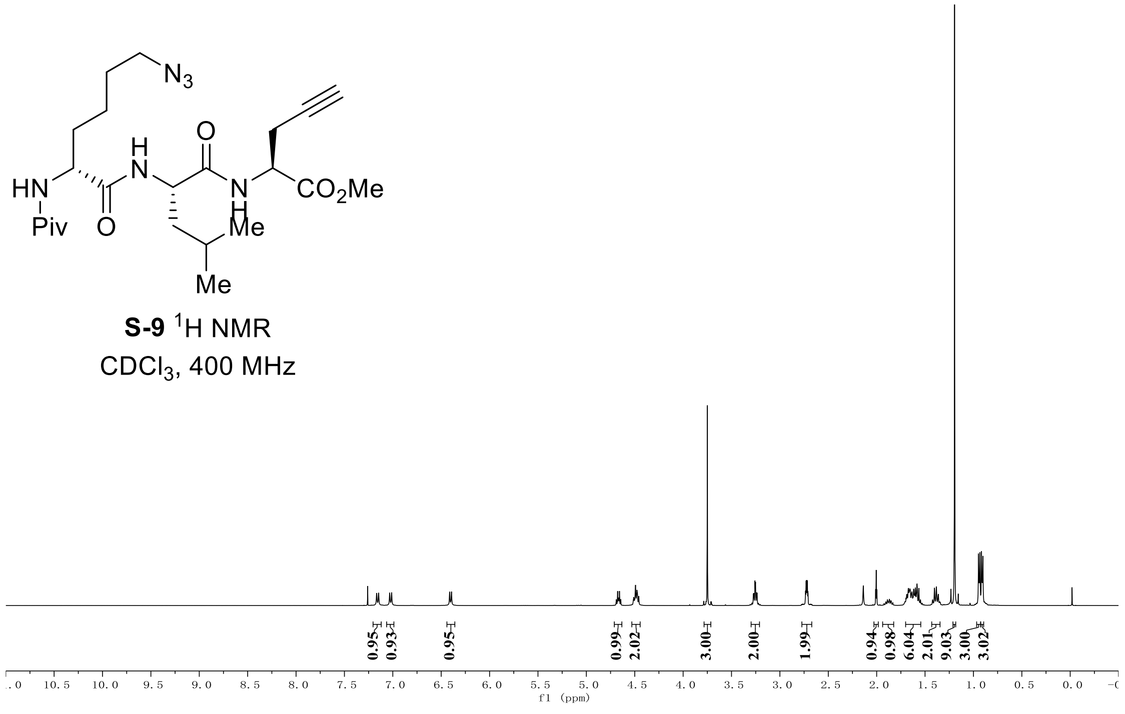

S-9 ^{1}H NMR
$CDCl_3$, 400 MHz

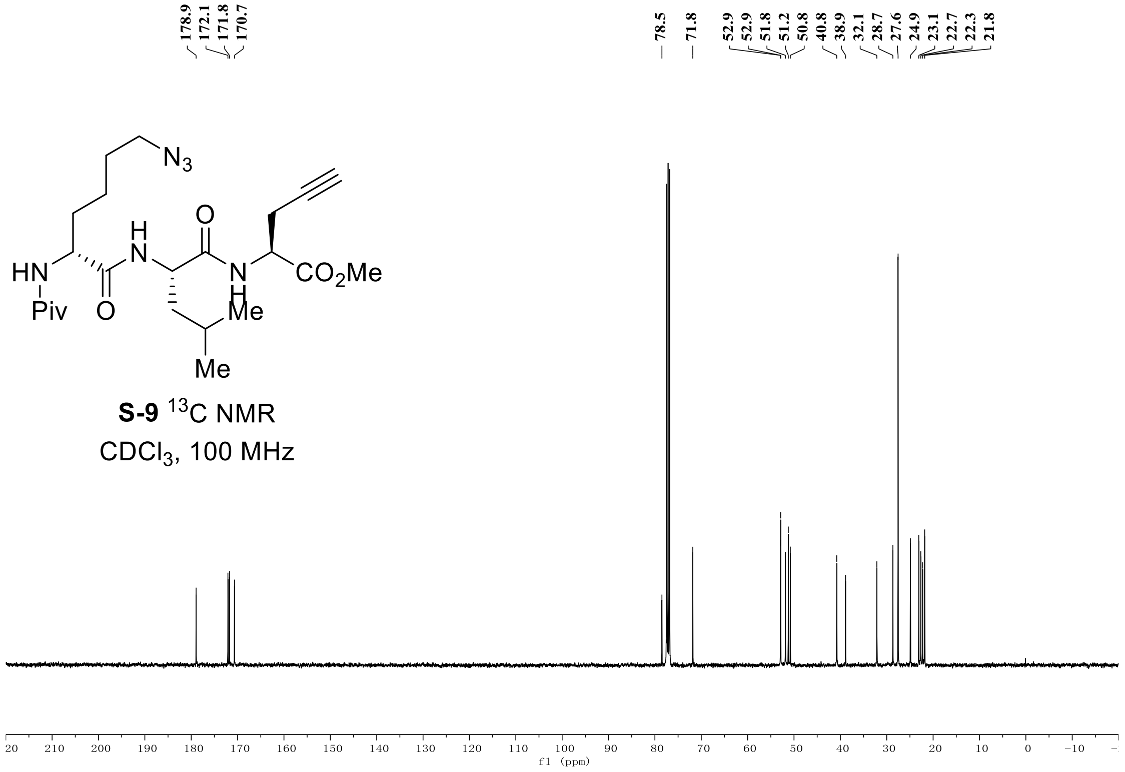

S-9 ^{13}C NMR
$CDCl_3$, 100 MHz

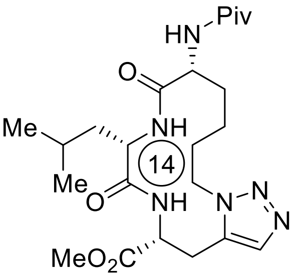

61 ^{1}H NMR
$CDCl_3$, 400 MHz

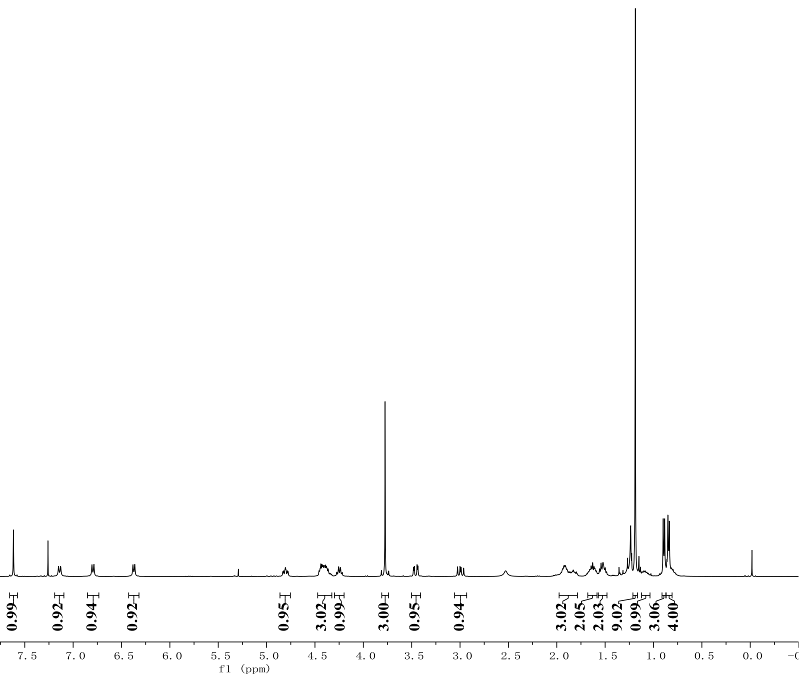

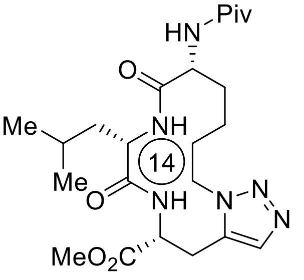

61 ^{13}C NMR
$CDCl_3$, 100 MHz

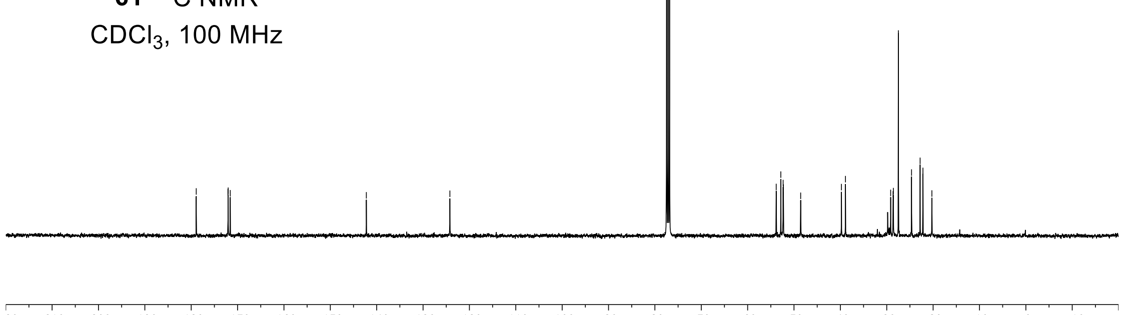

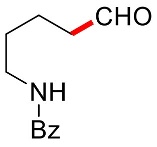

62b ^{1}H NMR

DMSO-d_6, 400 MHz

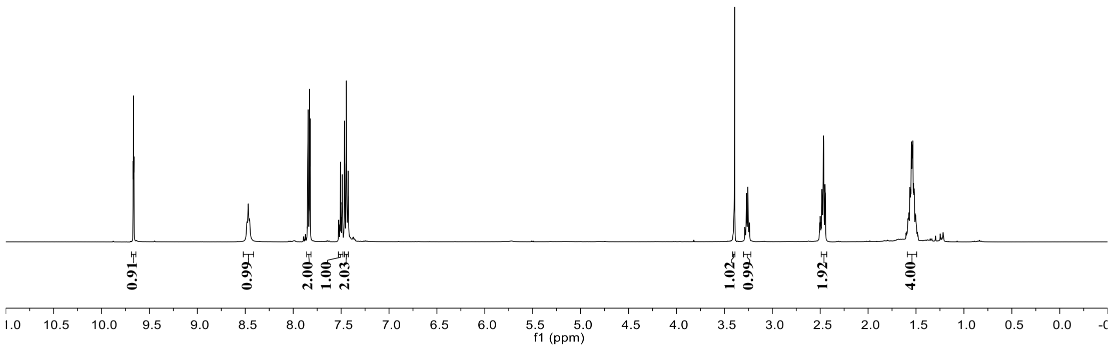

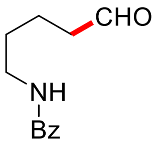

62b ^{13}C NMR

DMSO-d_6, 100 MHz

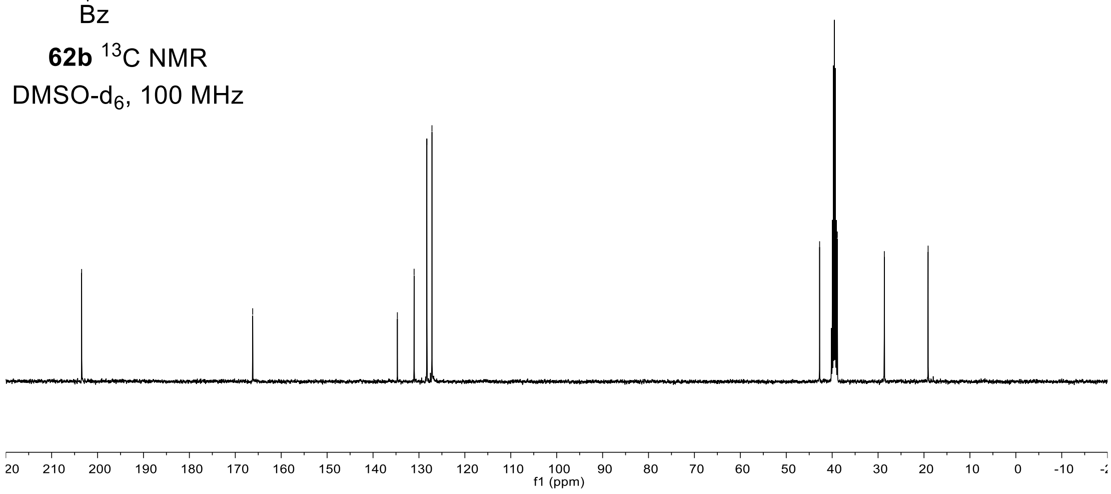

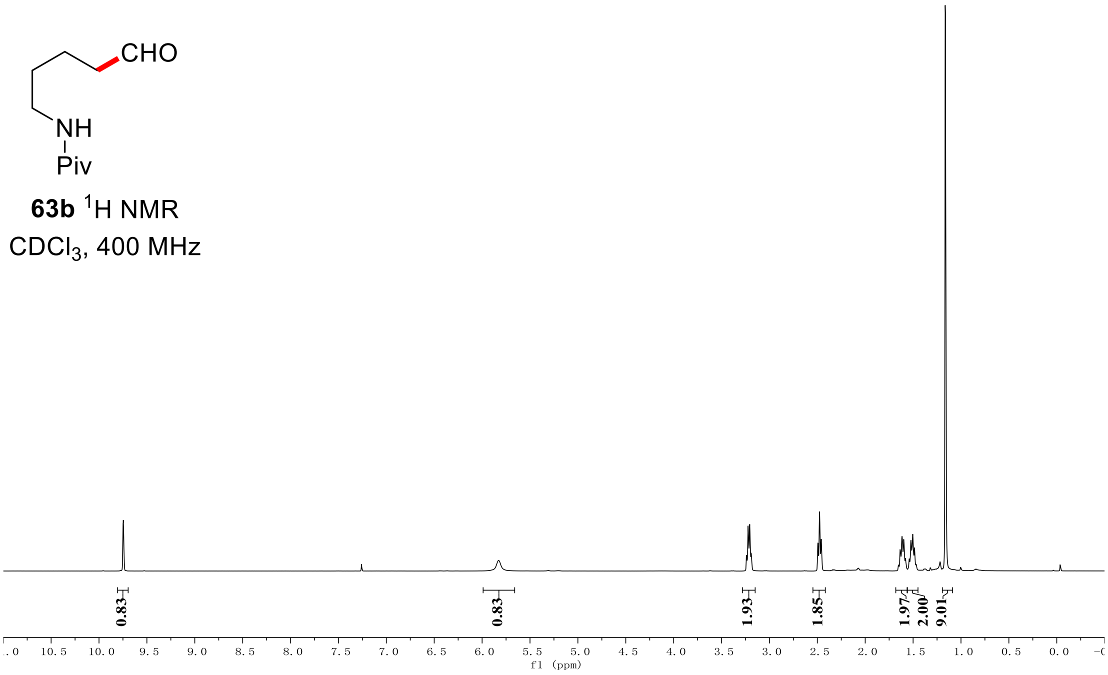
CHO
NH
Piv
63b 1H NMR
CDCl3, 400 MHz
0.83
0.83
1.93
1.85
1.97
2.00
9.01
f1 (ppm)

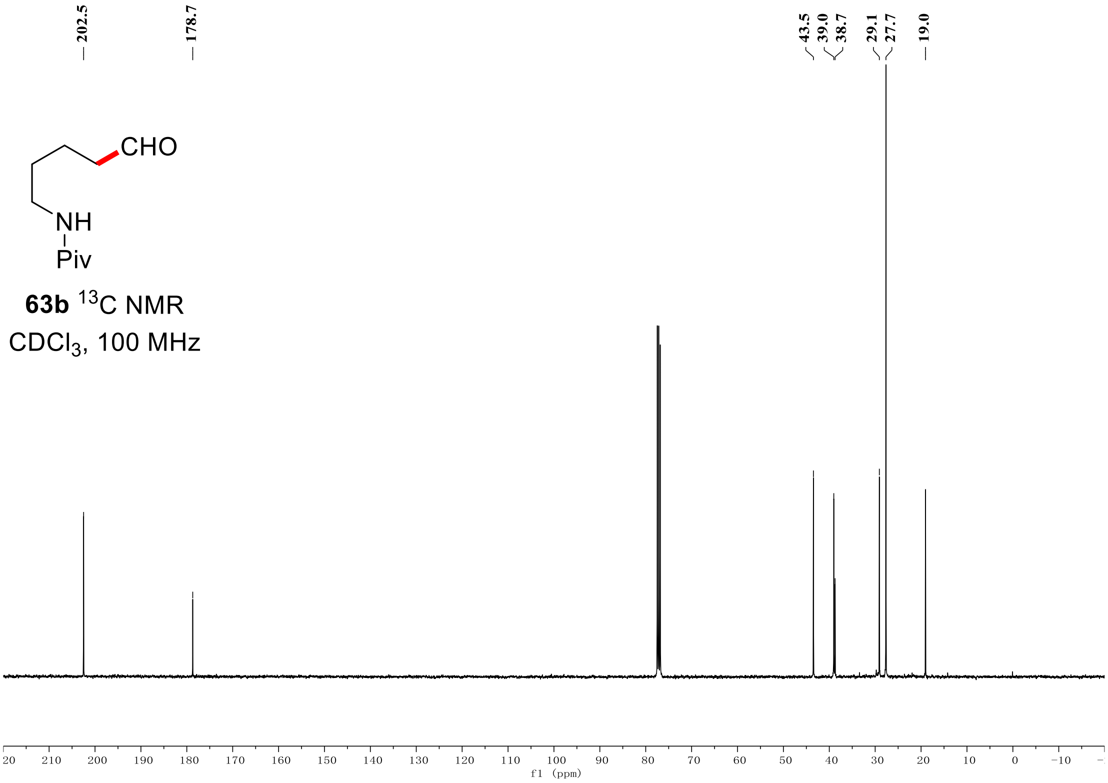
202.5
178.7
43.5
39.0
38.7
29.1
27.7
19.0
CHO
NH
Piv
63b 13C NMR
CDCl3, 100 MHz
f1 (ppm)

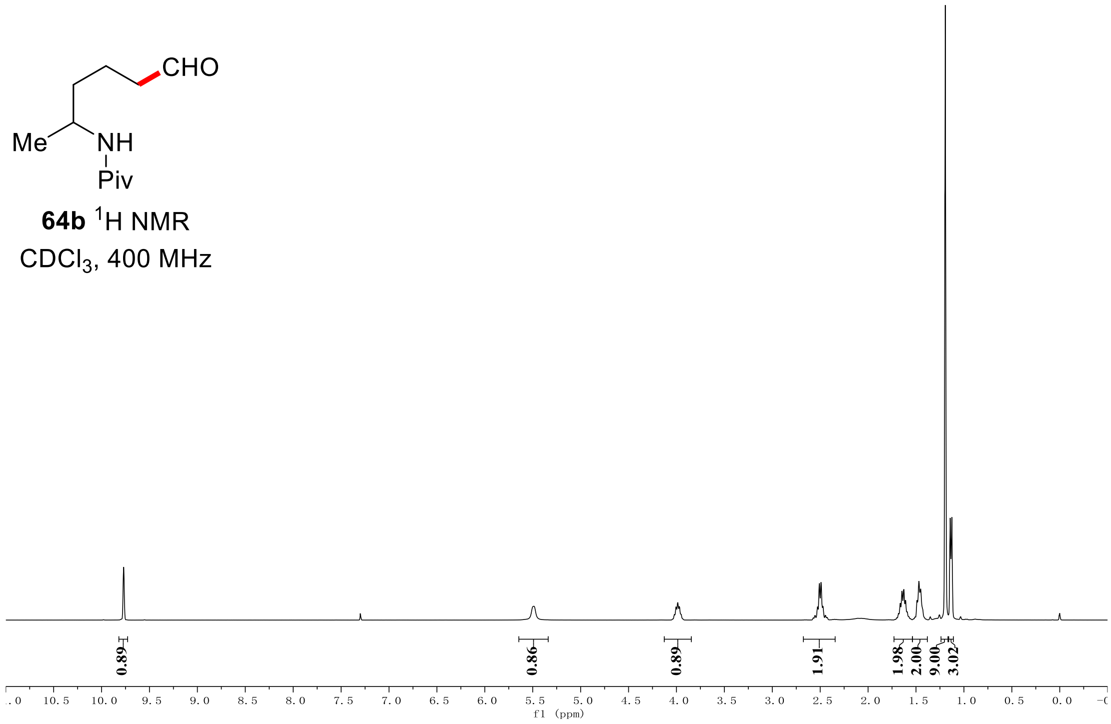
CHO
Me
NH
Piv
64b 1H NMR
CDCl3, 400 MHz
0.89
0.86
0.89
1.91
1.98
2.00
9.00
3.02
f1 (ppm)

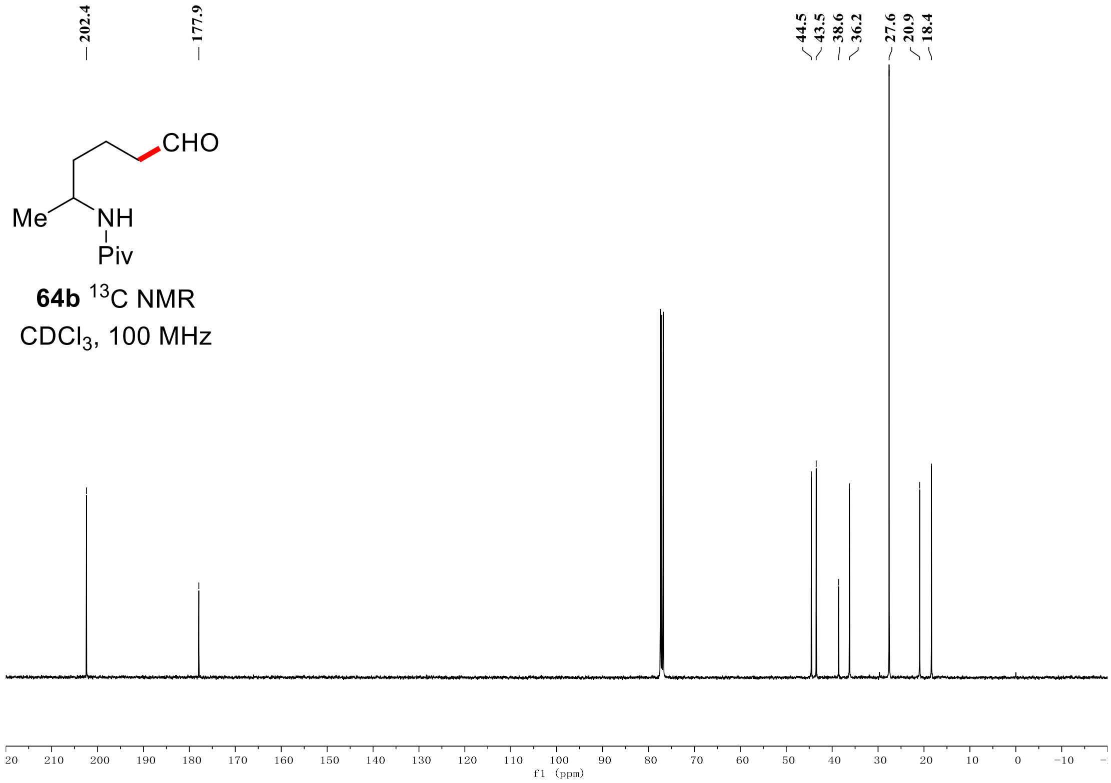
202.4
177.9
44.5
43.5
38.6
36.2
27.6
20.9
18.4
CHO
Me
NH
Piv
64b 13C NMR
CDCl3, 100 MHz
f1 (ppm)

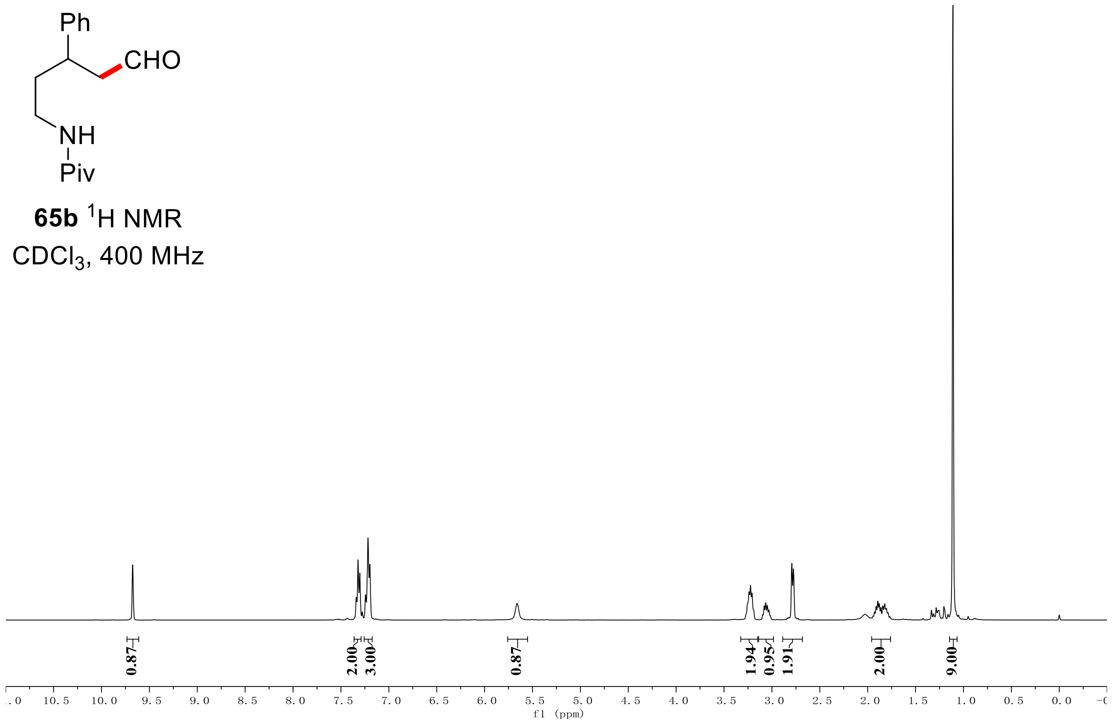

65b ^{1}H NMR
$CDCl_3$, 400 MHz

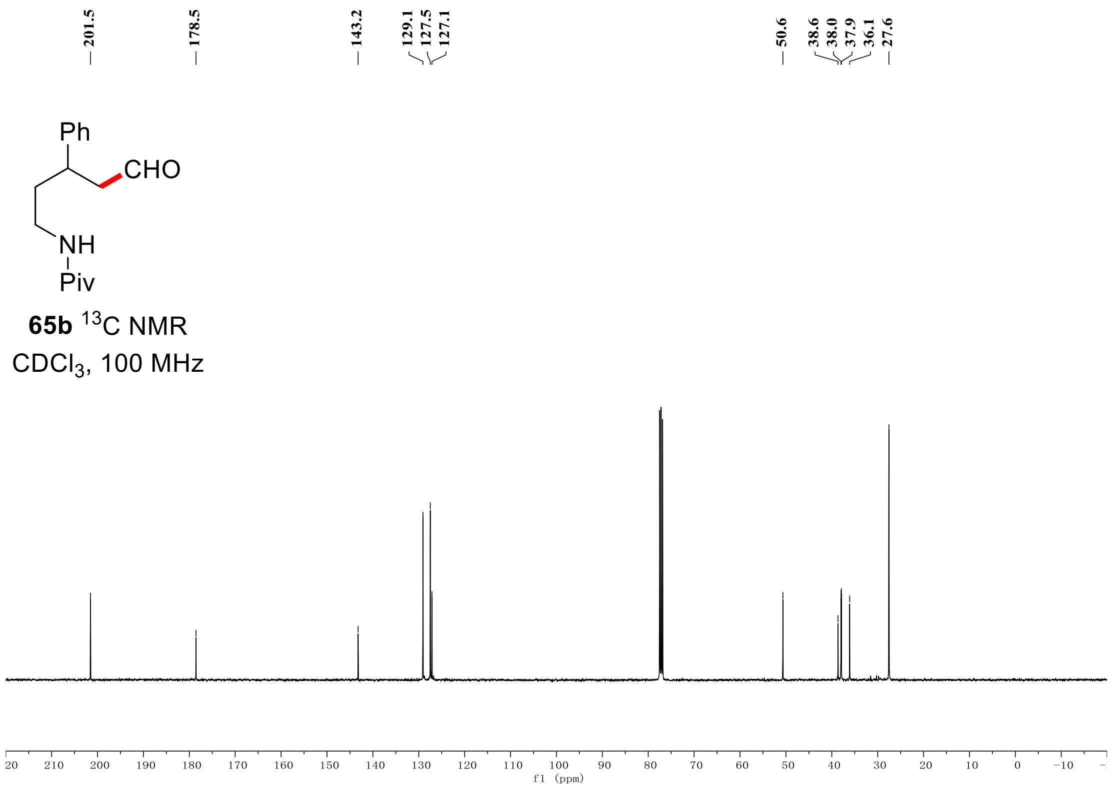

65b ^{13}C NMR
$CDCl_3$, 100 MHz

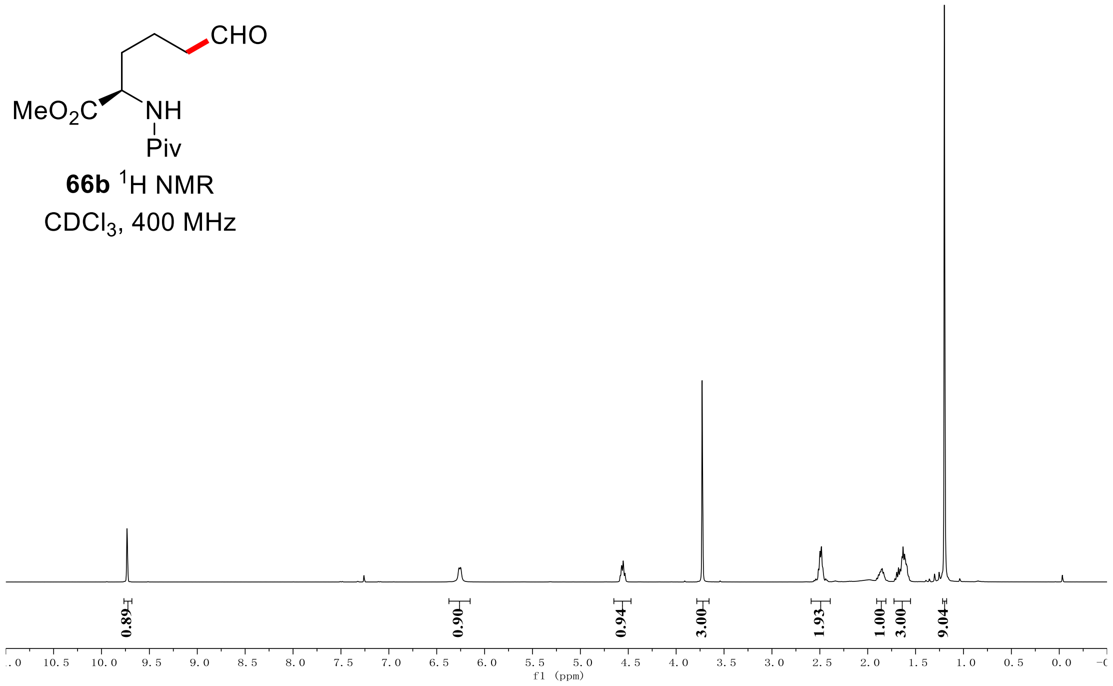
CHO
MeO2C
NH
Piv
66b 1H NMR
CDCl3, 400 MHz
0.89
0.90
0.94
3.00
1.93
1.00
3.00
9.04
f1 (ppm)

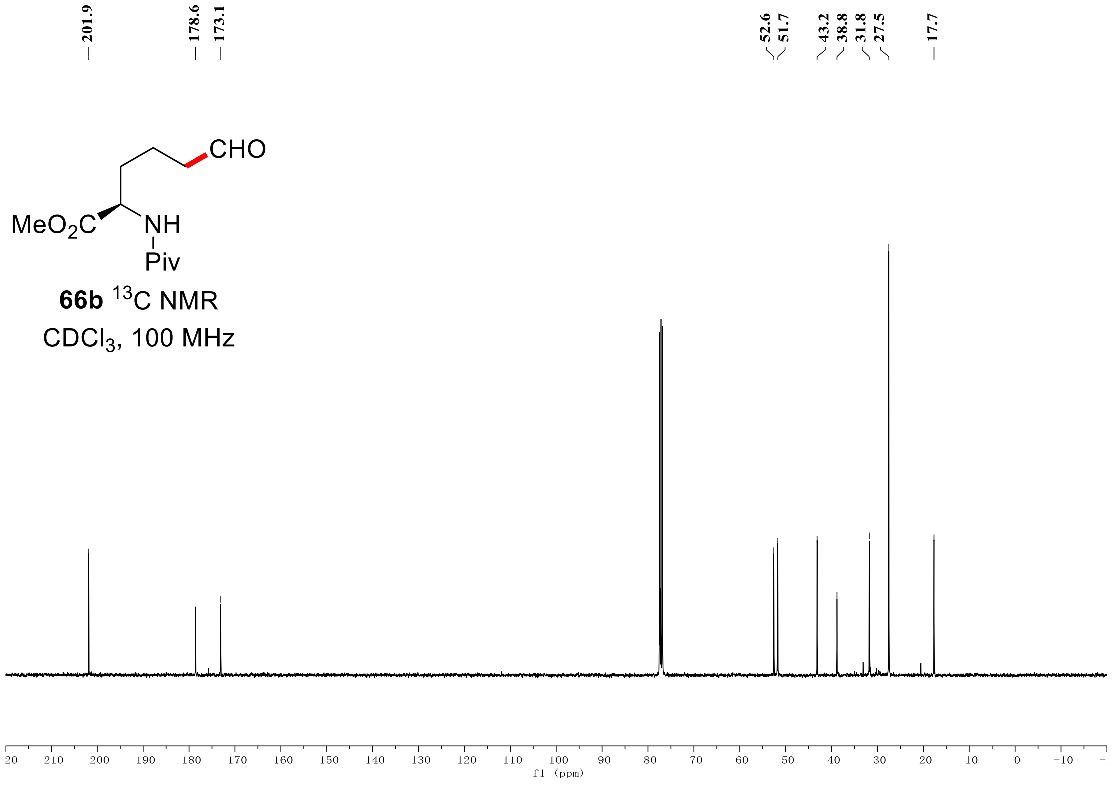
201.9
178.6
173.1
52.6
51.7
43.2
38.8
31.8
27.5
17.7
CHO
MeO2C
NH
Piv
66b 13C NMR
CDCl3, 100 MHz
f1 (ppm)

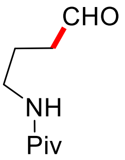

67b ^{1}H NMR

$CDCl_3$, 400 MHz

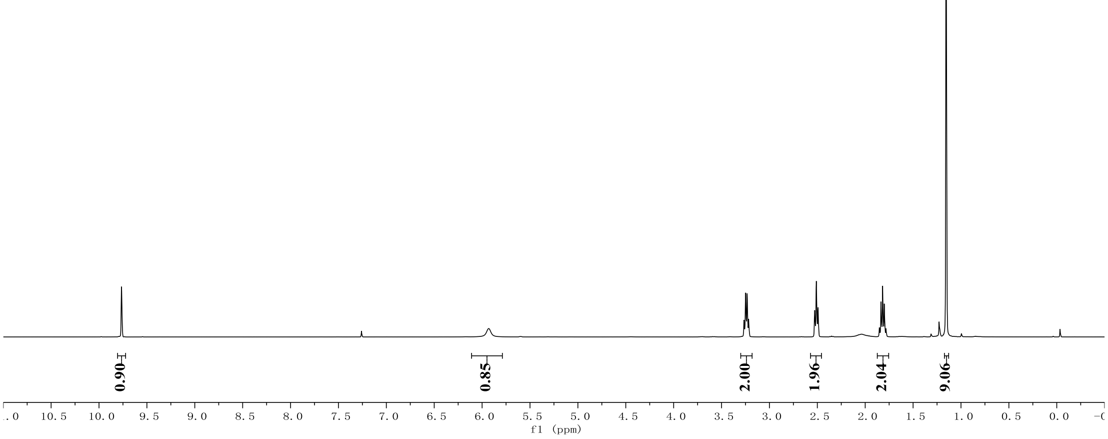

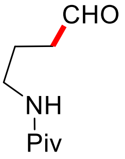

67b ^{13}C NMR

$CDCl_3$, 100 MHz

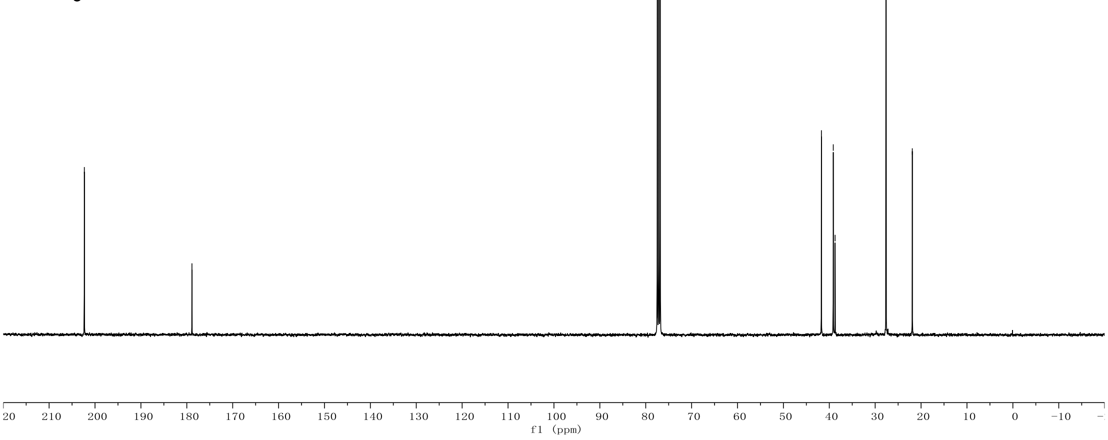

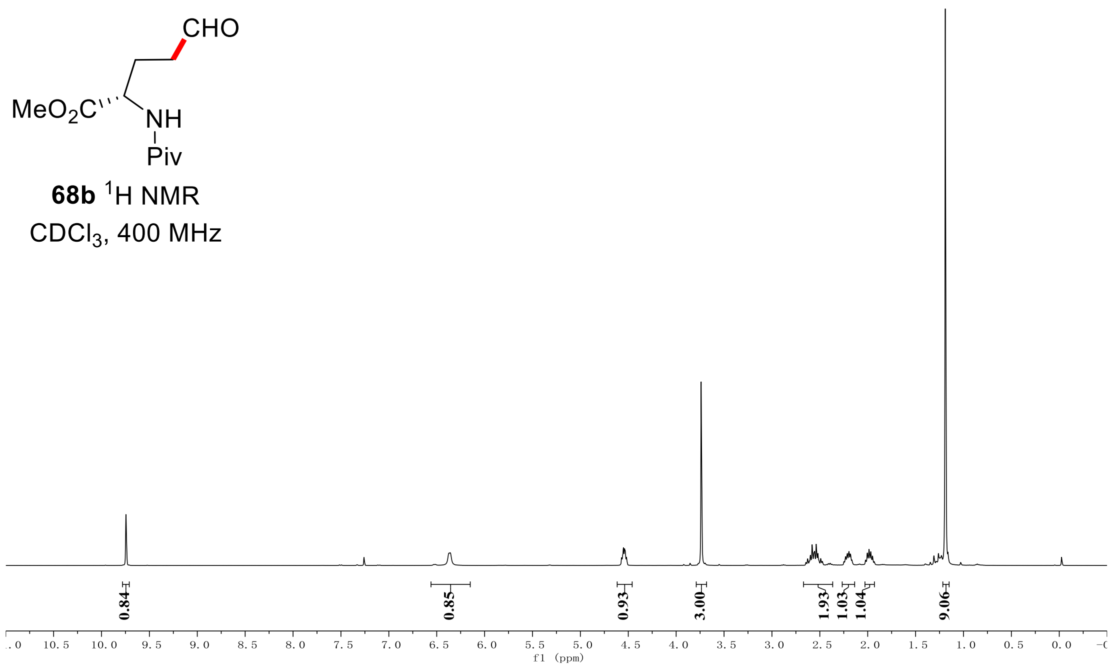
CHO
MeO2C
NH
Piv
68b 1H NMR
CDCl3, 400 MHz
0.84
0.85
0.93
3.00
1.93
1.03
1.04
9.06
f1 (ppm)

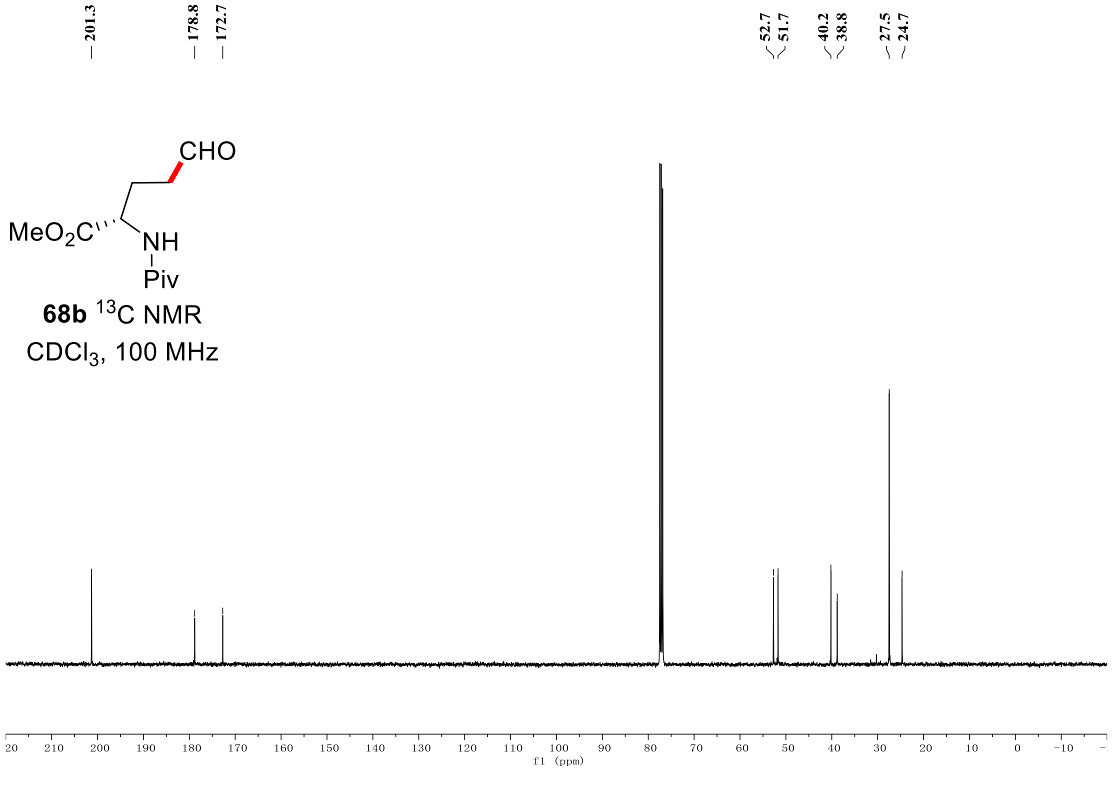
201.3
178.8
172.7
52.7
51.7
40.2
38.8
27.5
24.7
CHO
MeO2C
NH
Piv
68b 13C NMR
CDCl3, 100 MHz
f1 (ppm)

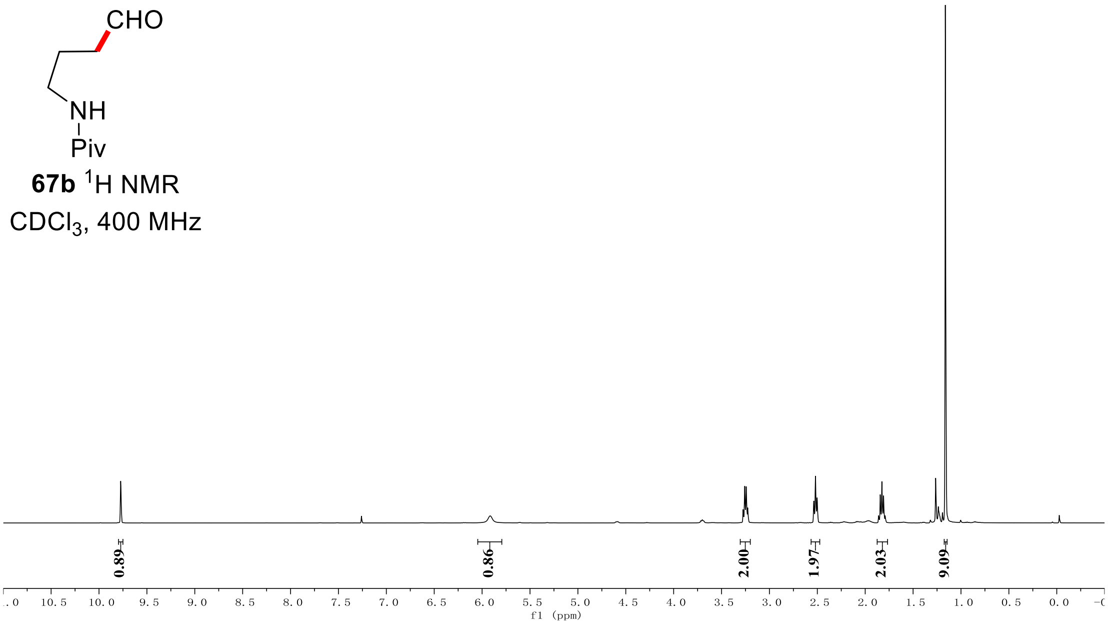
CHO
NH
Piv
67b 1H NMR
CDCl3, 400 MHz
0.89
0.86
2.00
1.97
2.03
9.09
f1 (ppm)

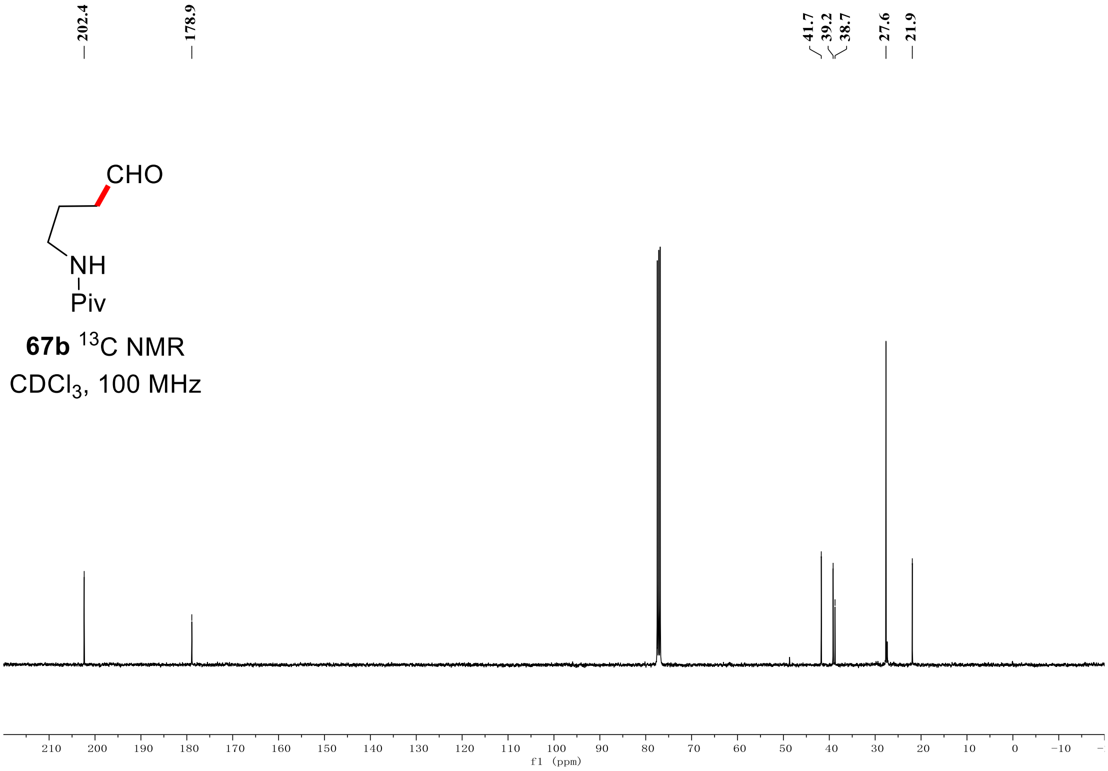
202.4
178.9
41.7
39.2
38.7
27.6
21.9
CHO
NH
Piv
67b 13C NMR
CDCl3, 100 MHz
f1 (ppm)

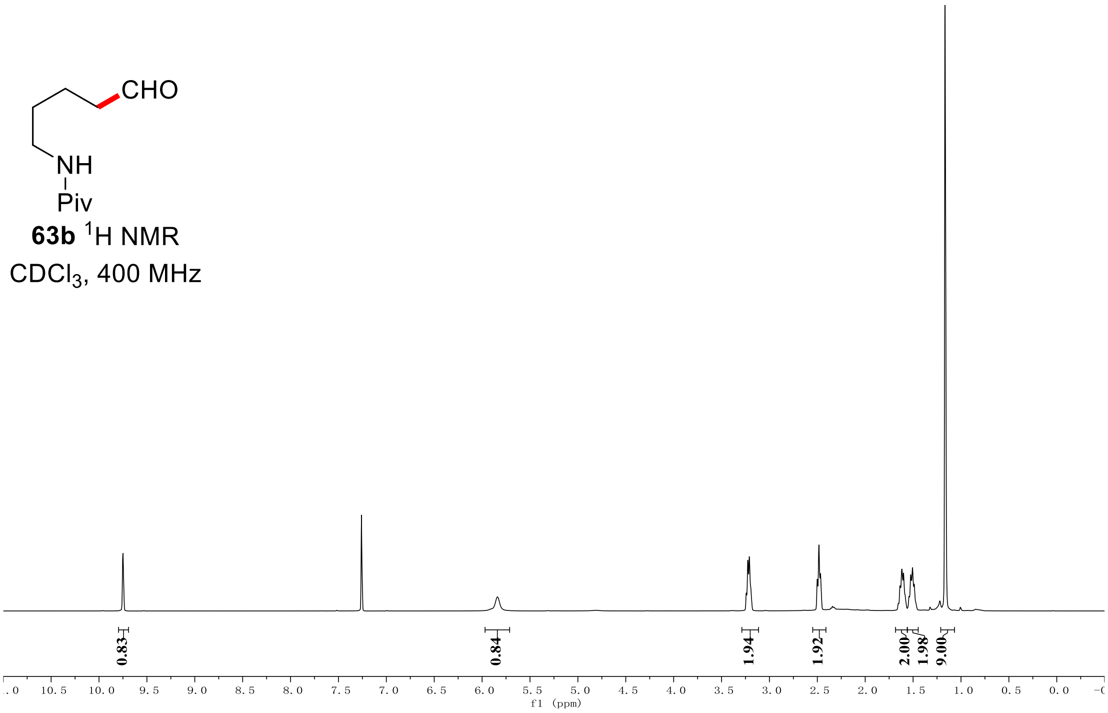
CHO
NH
Piv
63b ¹H NMR
CDCl$_3$, 400 MHz
0.83
0.84
1.94
1.92
2.00
1.98
9.00
f1 (ppm)

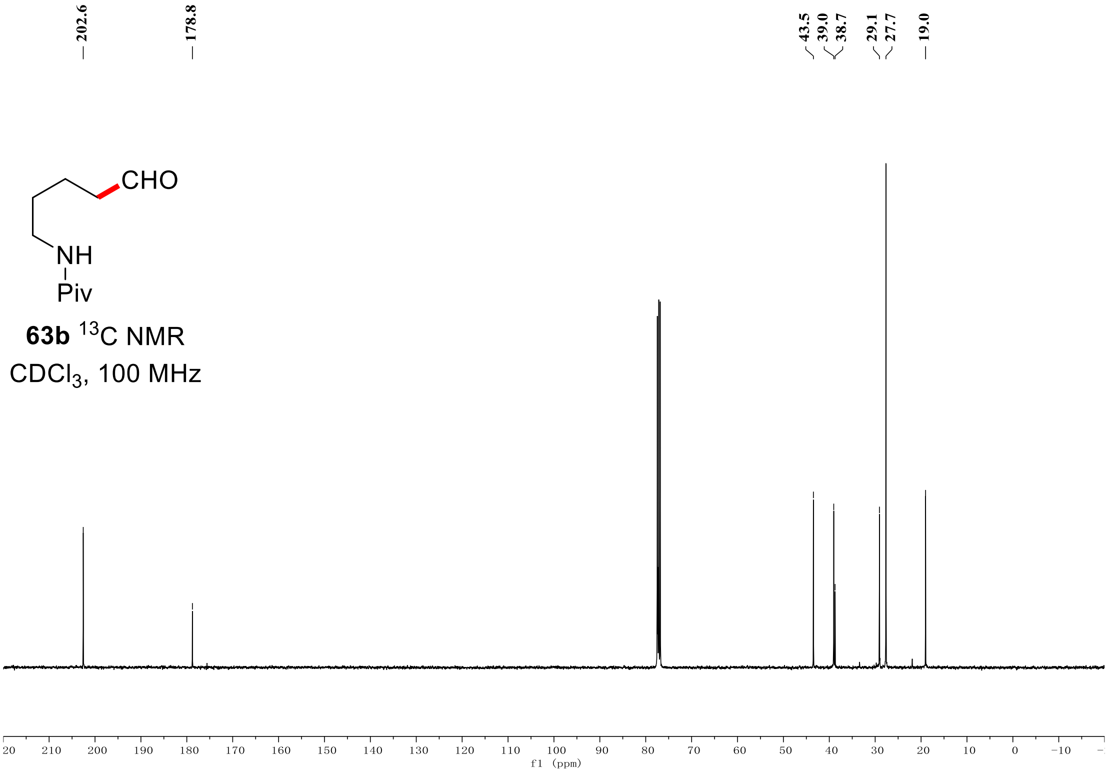
202.6
178.8
43.5
39.0
38.7
29.1
27.7
19.0
CHO
NH
Piv
63b ¹³C NMR
CDCl$_3$, 100 MHz
f1 (ppm)

71b ^{1}H NMR

$CDCl_3$, 400 MHz

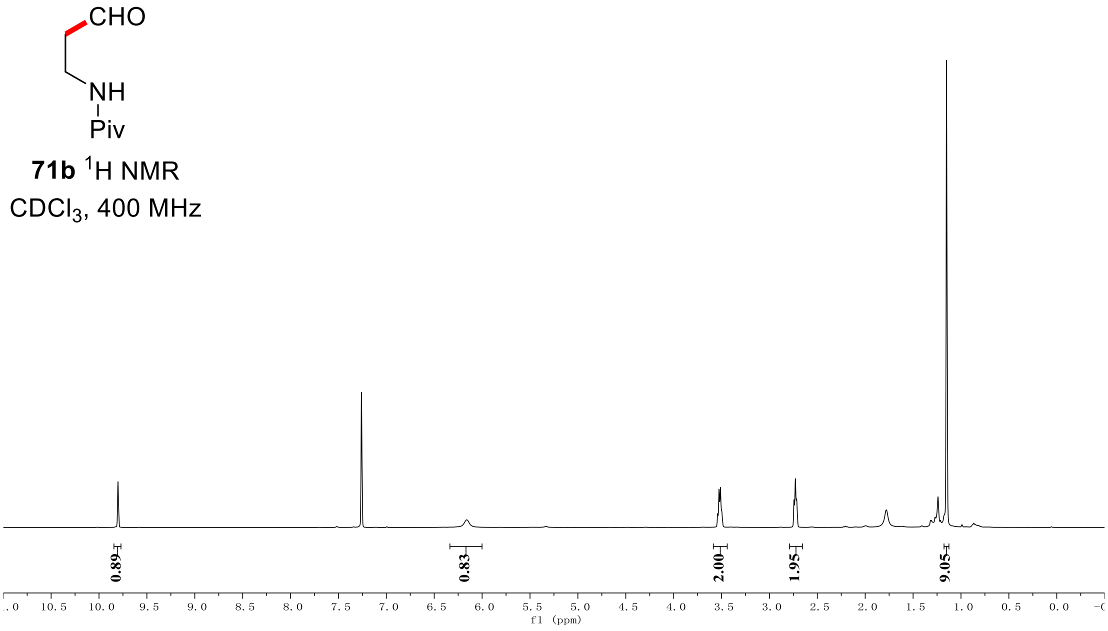

71b ^{13}C NMR

$CDCl_3$, 100 MHz

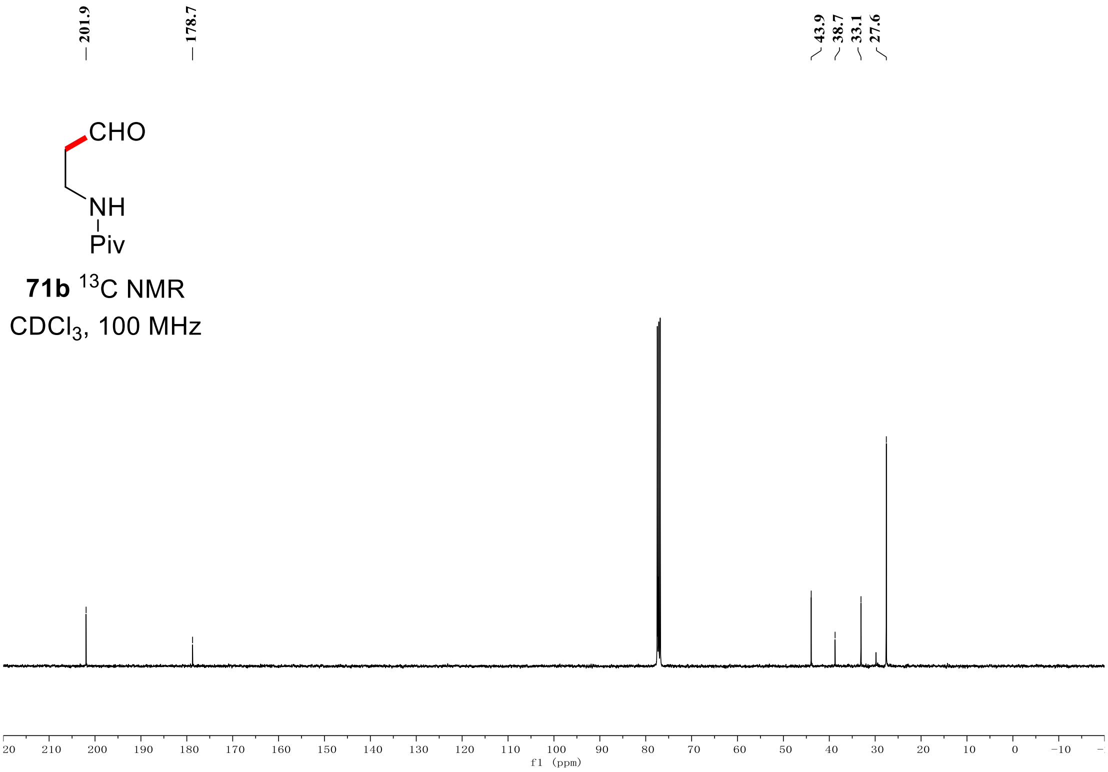

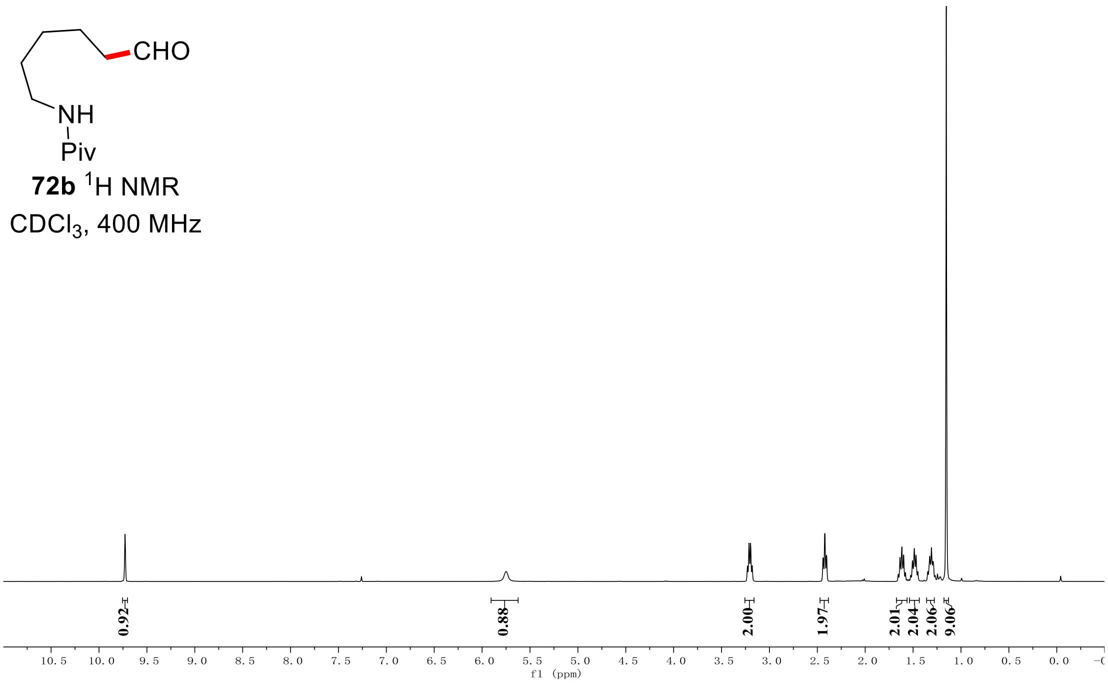
CHO
NH
Piv
72b 1H NMR
CDCl3, 400 MHz
0.92
0.88
2.00
1.97
2.01
2.04
2.06
9.06
10.5 10.0 9.5 9.0 8.5 8.0 7.5 7.0 6.5 6.0 5.5 5.0 4.5 4.0 3.5 3.0 2.5 2.0 1.5 1.0 0.5 0.0
f1 (ppm)

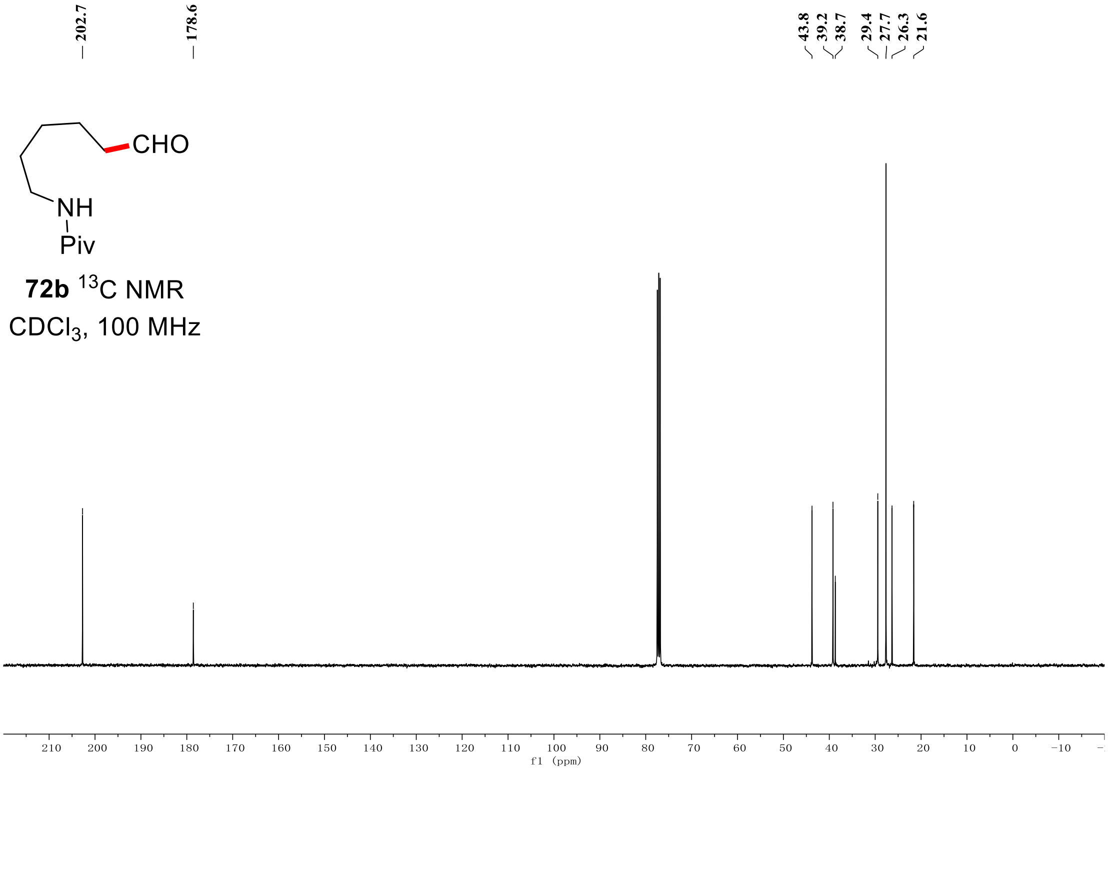
202.7
178.6
43.8
39.2
38.7
29.4
27.7
26.3
21.6
CHO
NH
Piv
72b 13C NMR
CDCl3, 100 MHz
210 200 190 180 170 160 150 140 130 120 110 100 90 80 70 60 50 40 30 20 10 0 -10
f1 (ppm)

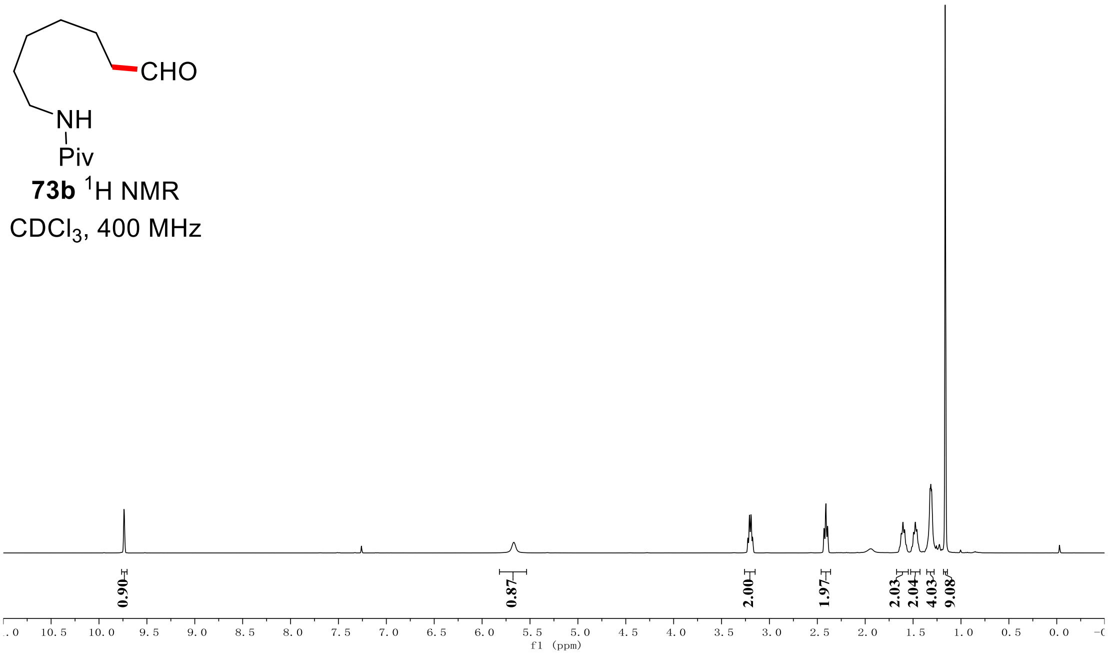

73b ^{1}H NMR
$CDCl_3$, 400 MHz

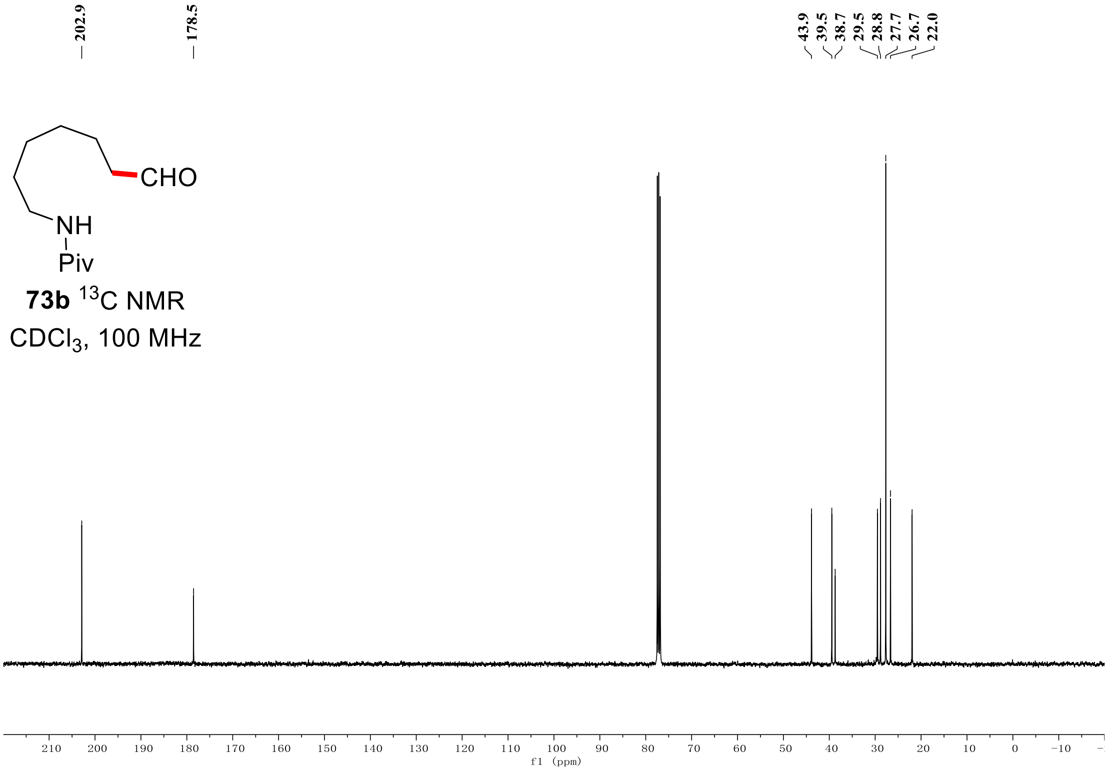

73b ^{13}C NMR
$CDCl_3$, 100 MHz

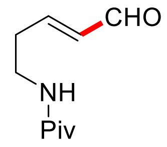

74b ^{1}H NMR
$CDCl_3$, 400 MHz

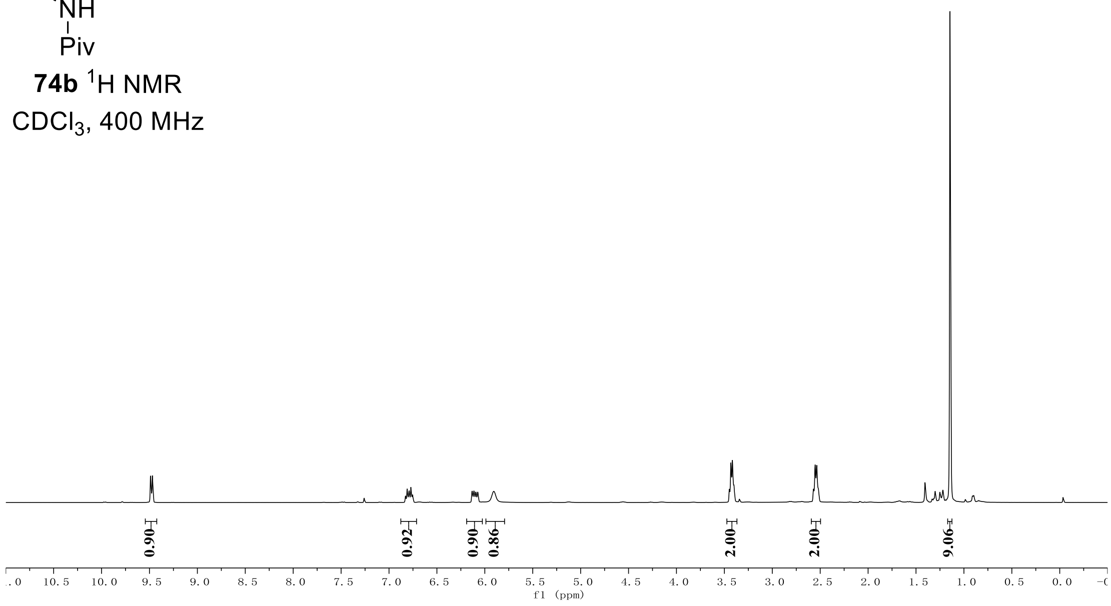

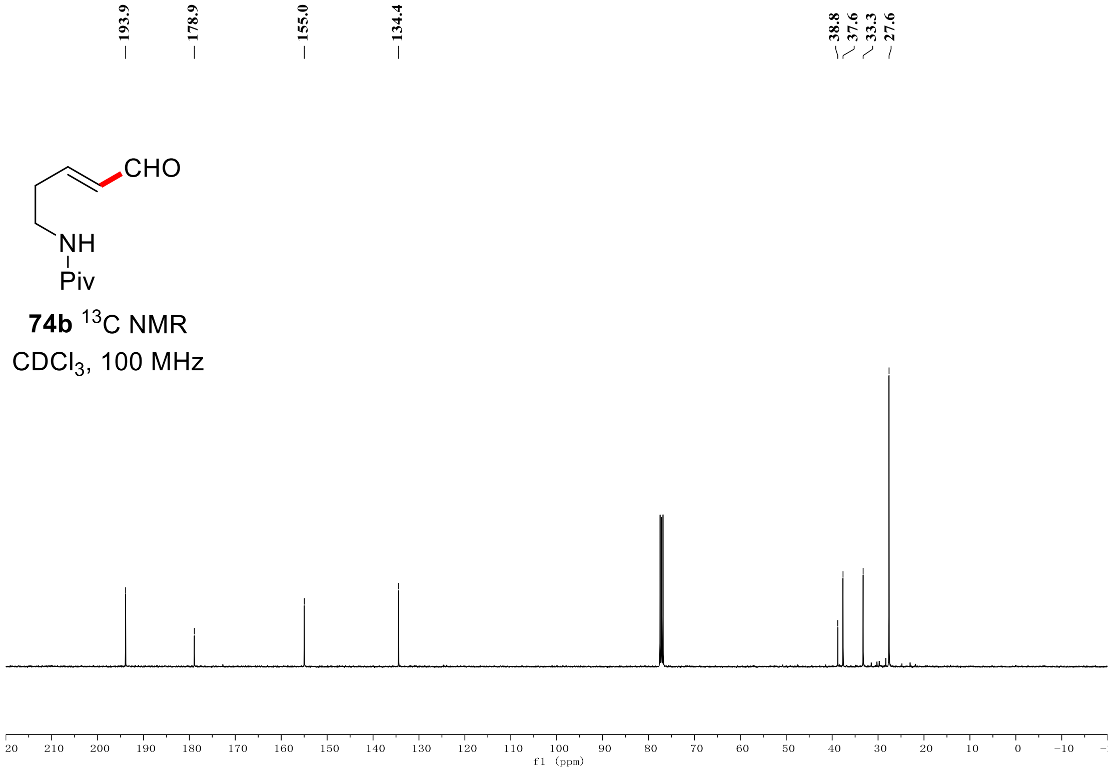

74b ^{13}C NMR
$CDCl_3$, 100 MHz

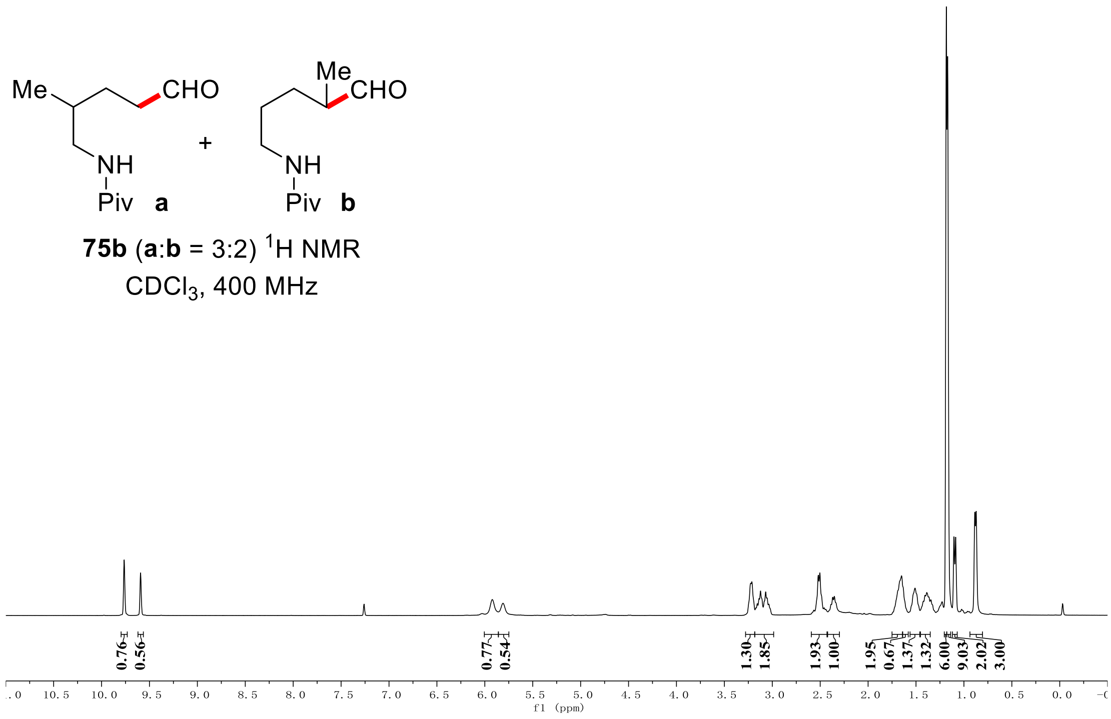

Me
CHO
NH
Piv a
+
Me
CHO
NH
Piv b
75b (a:b = 3:2) 1H NMR
CDCl3, 400 MHz
0.76
0.56
0.77
0.54
1.30
1.85
1.93
1.00
1.95
0.67
1.37
1.32
6.00
9.03
2.02
3.00
f1 (ppm)

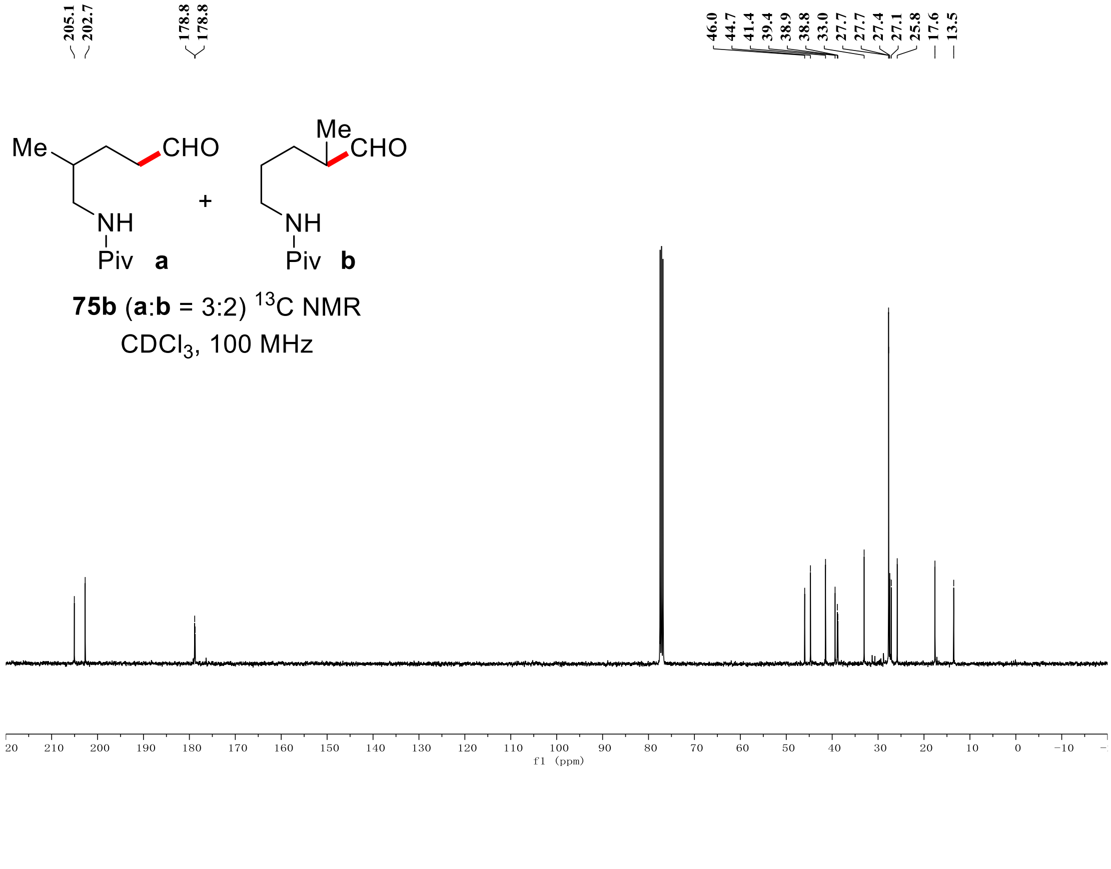

205.1
202.7
178.8
178.8
46.0
44.7
41.4
39.4
38.9
38.8
33.0
27.7
27.7
27.4
27.1
25.8
17.6
13.5
Me
CHO
NH
Piv a
+
Me
CHO
NH
Piv b
75b (a:b = 3:2) 13C NMR
CDCl3, 100 MHz
f1 (ppm)

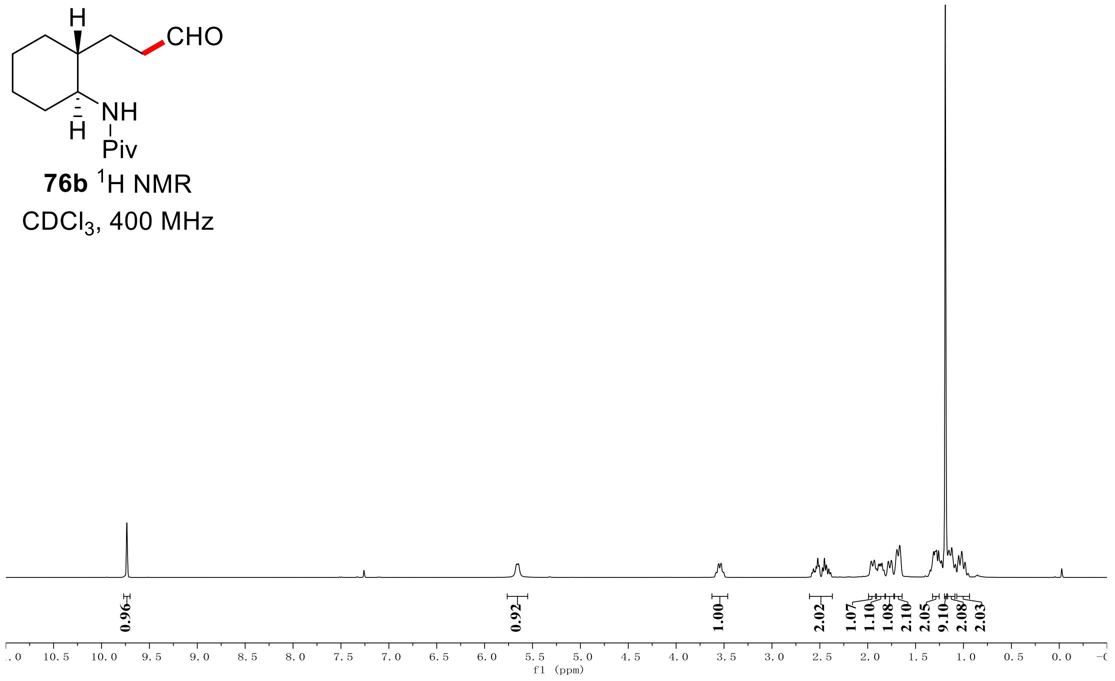
CHO
NH
Piv
76b ^{1}H NMR
$CDCl_3$, 400 MHz
0.96
0.92
1.00
2.02
1.07
1.10
1.08
2.10
2.05
9.10
2.08
2.03
f1 (ppm)

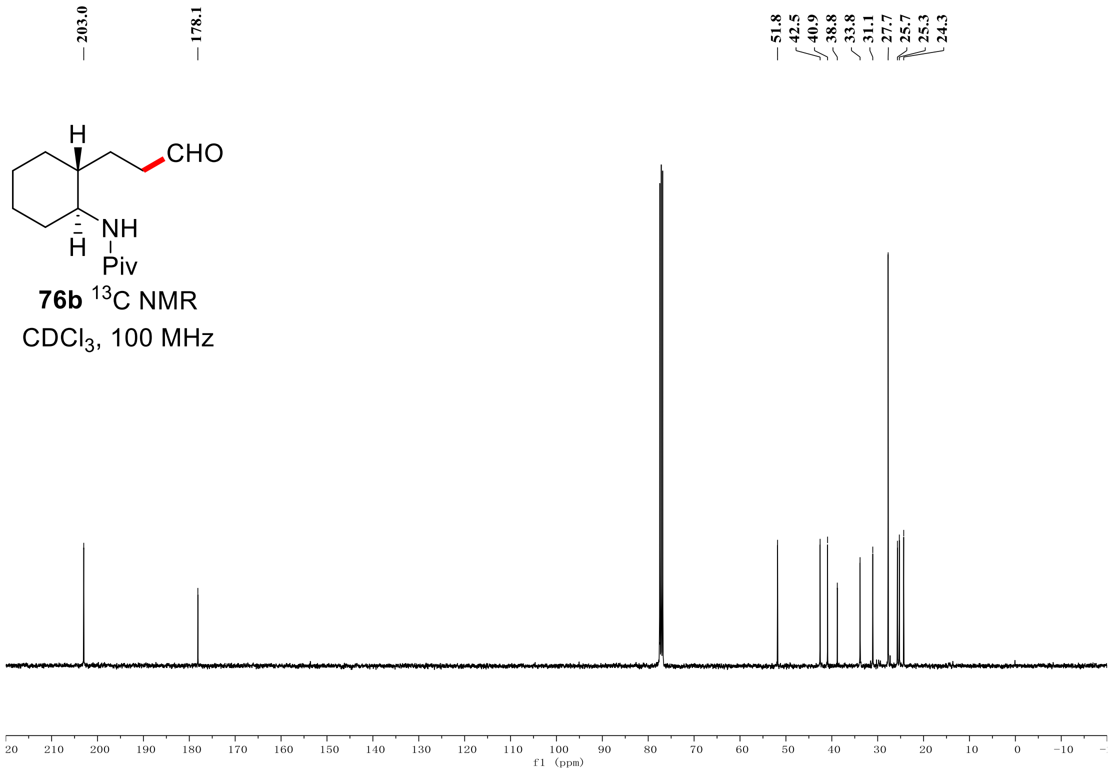
203.0
178.1
51.8
42.5
40.9
38.8
33.8
31.1
27.7
25.7
25.3
24.3
CHO
NH
Piv
76b ^{13}C NMR
$CDCl_3$, 100 MHz
f1 (ppm)

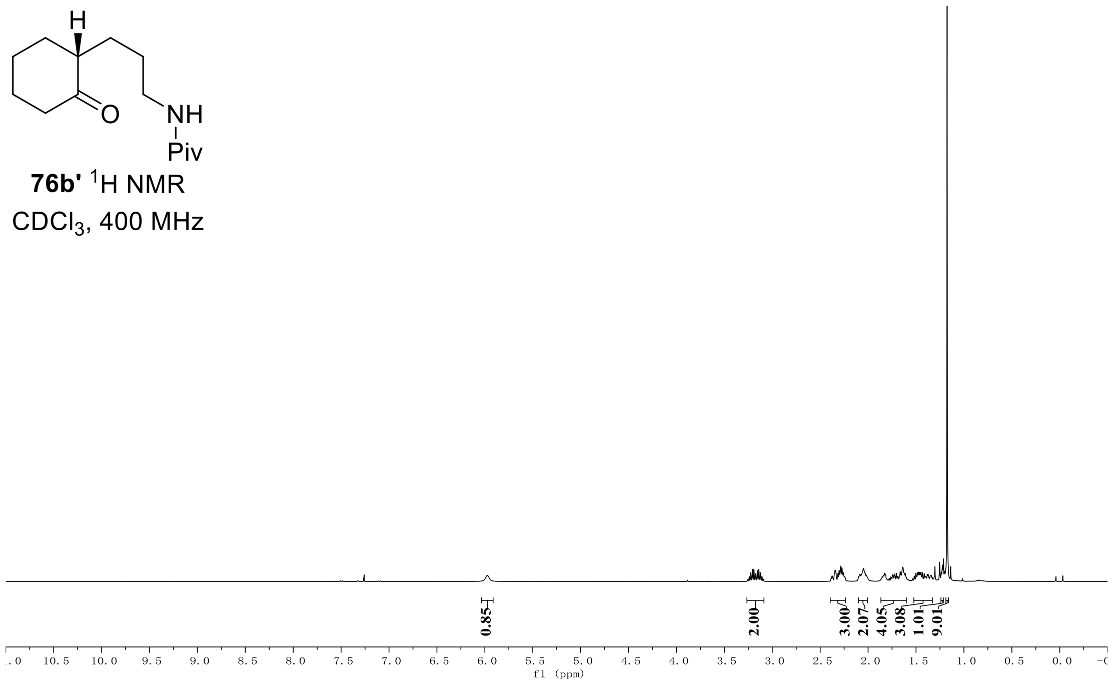
H
O
NH
Piv
76b' 1H NMR
CDCl3, 400 MHz
0.85
2.00
3.00
2.07
4.05
3.08
1.01
9.01
f1 (ppm)

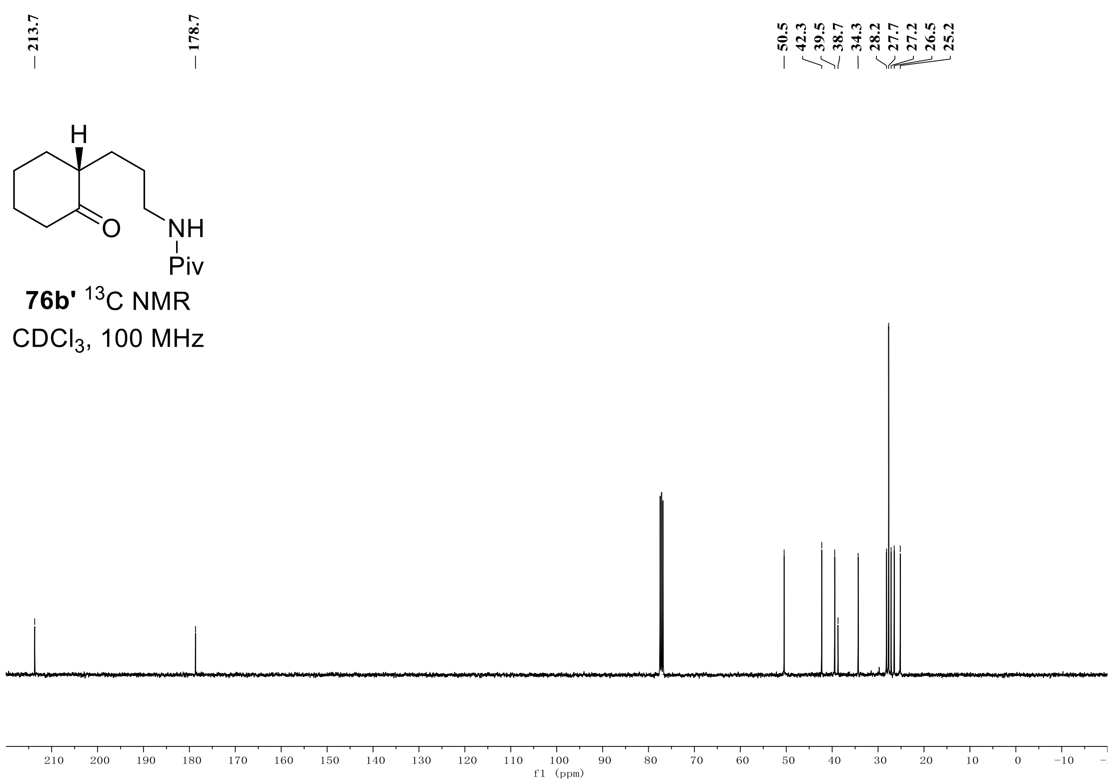
213.7
178.7
50.5
42.3
39.5
38.7
34.3
28.2
27.7
27.2
26.5
25.2
H
O
NH
Piv
76b' 13C NMR
CDCl3, 100 MHz
f1 (ppm)

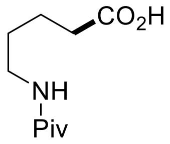

63c ^{1}H NMR
$CDCl_3$, 400 MHz

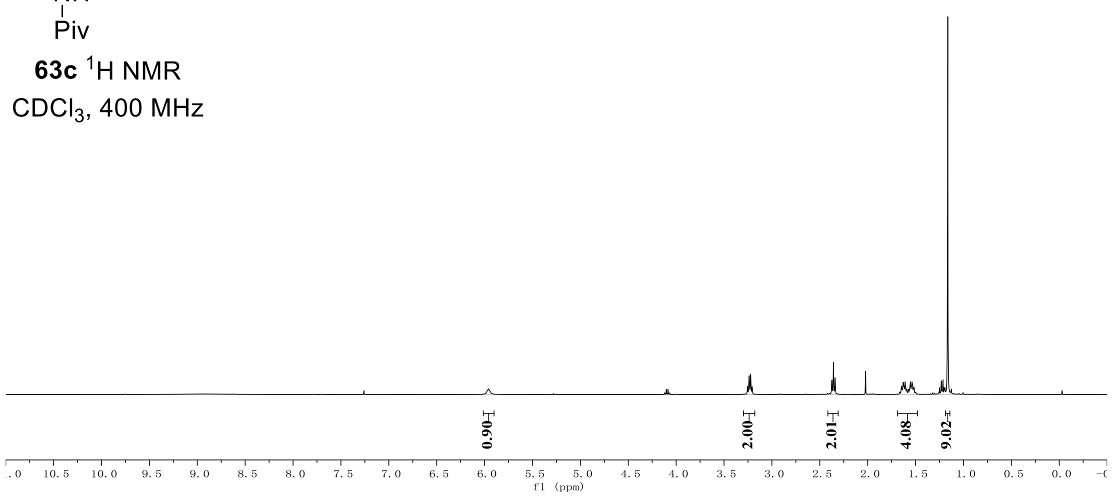

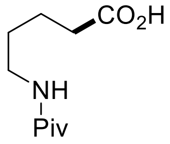

63c ^{13}C NMR
$CDCl_3$, 100 MHz

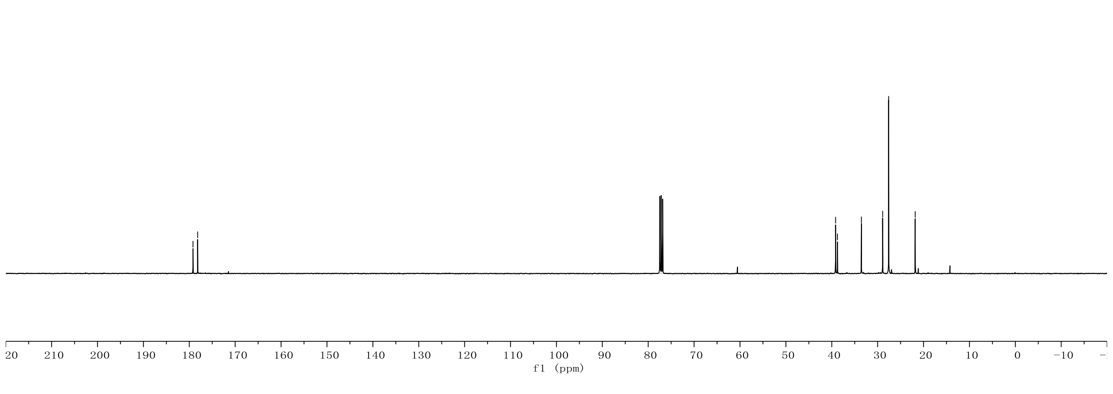

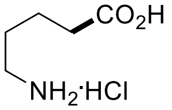

77 ^{1}H NMR
DMSO-d_6, 400 MHz

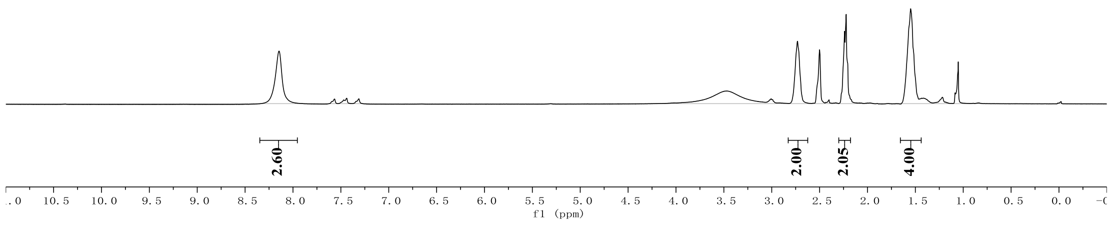

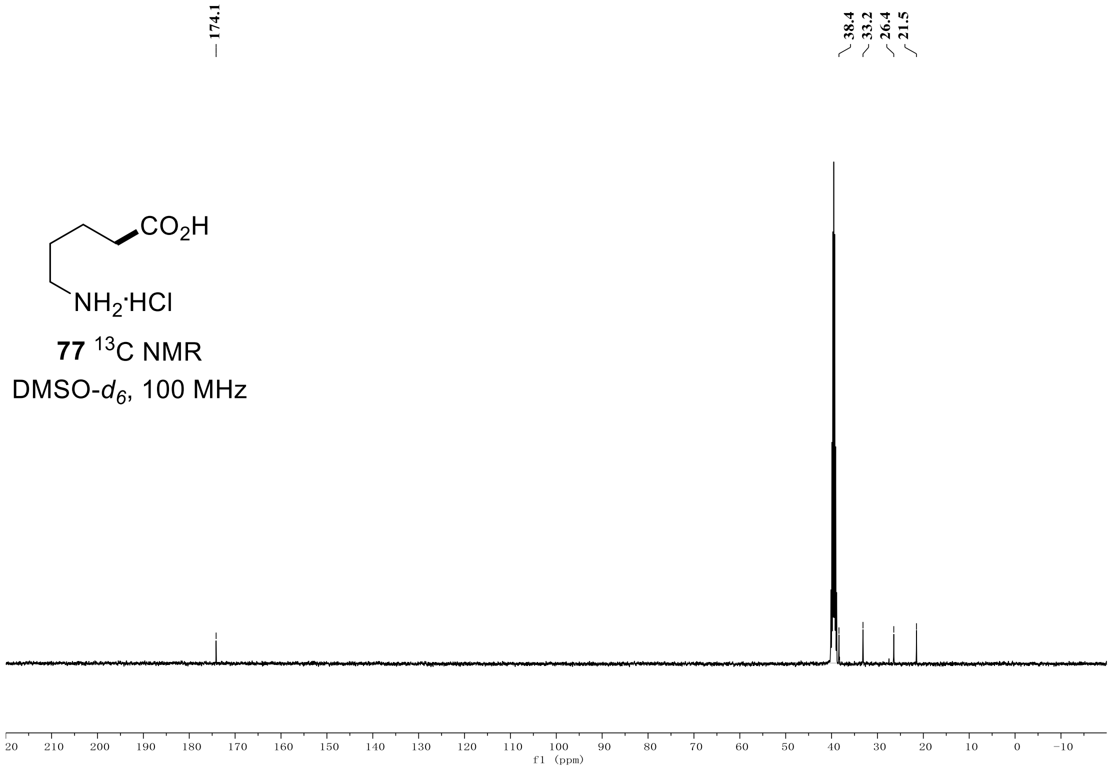

77 ^{13}C NMR
DMSO-d_6, 100 MHz

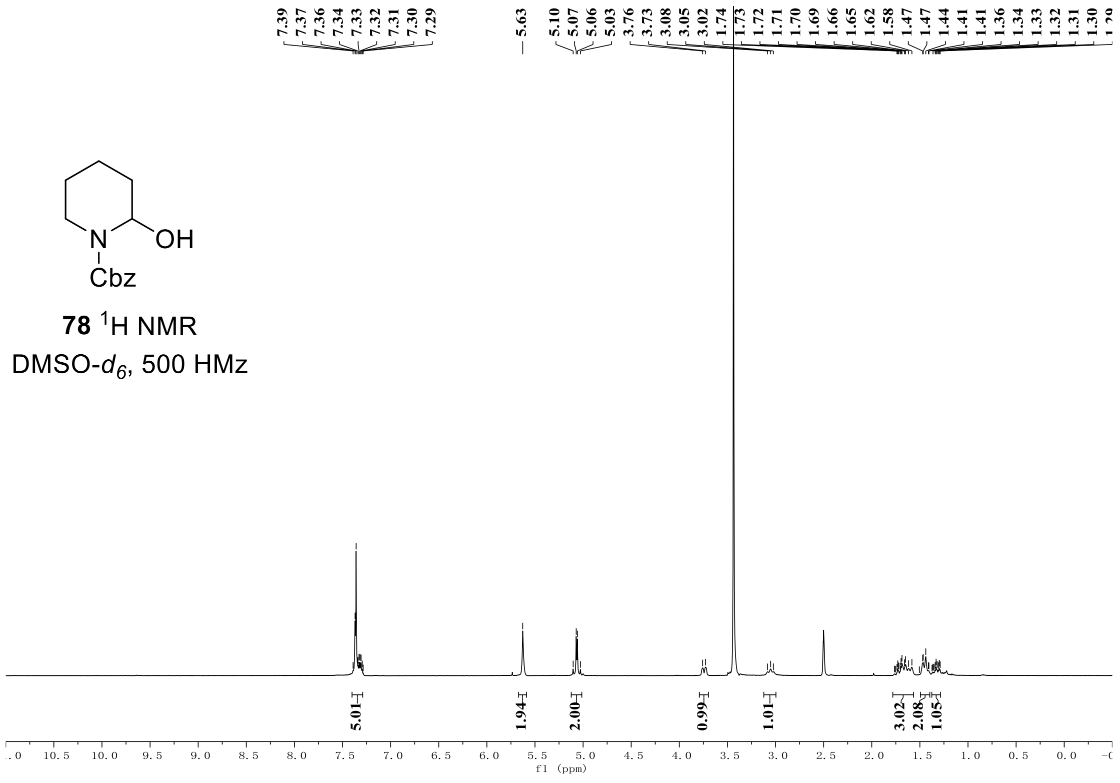
OH
N
Cbz
78 1H NMR
DMSO-d6, 500 HMz
7.39 7.37 7.36 7.34 7.33 7.32 7.31 7.30 7.29
5.63
5.10 5.07 5.06 5.03
3.76 3.73 3.08 3.05 3.02
1.74 1.73 1.72 1.71 1.70 1.69 1.66 1.65 1.62 1.58 1.47 1.47 1.44 1.41 1.41 1.36 1.34 1.33 1.32 1.31 1.30
5.01
1.94
2.00
0.99
1.01
3.02
2.08
1.05
f1 (ppm)

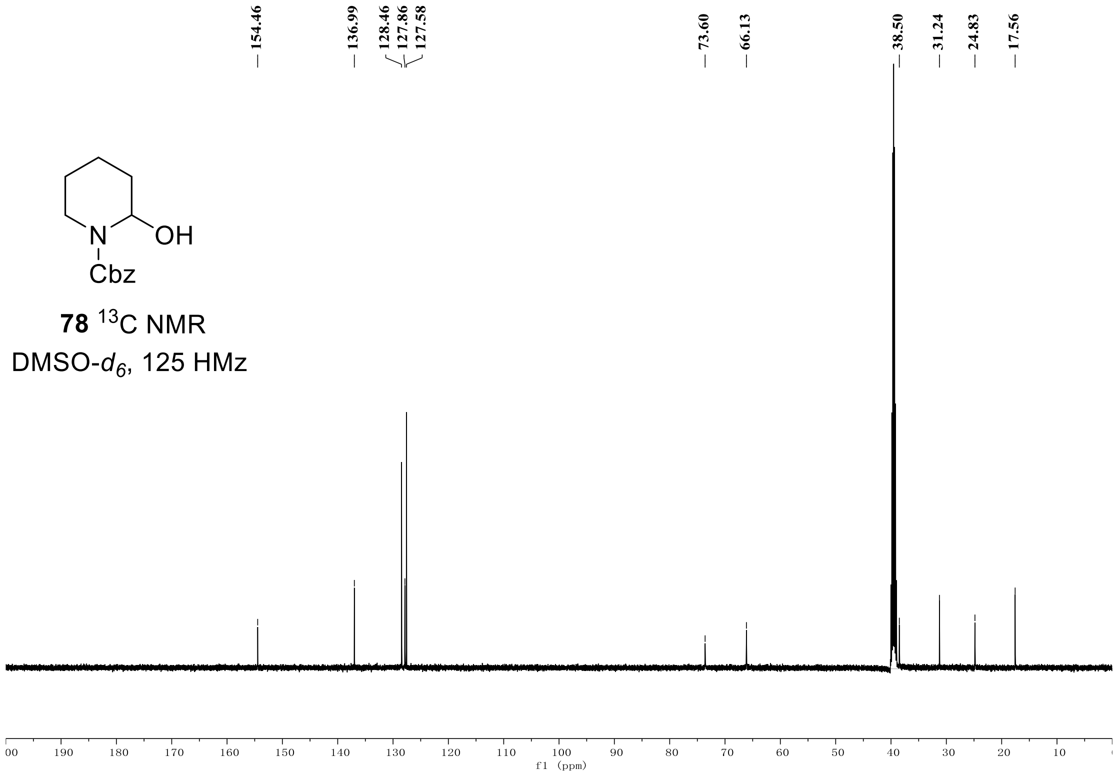
OH
N
Cbz
78 13C NMR
DMSO-d6, 125 HMz
154.46
136.99
128.46 127.86 127.58
73.60
66.13
38.50
31.24
24.83
17.56
f1 (ppm)

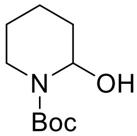

79 ^{1}H NMR
DMSO-d_6, 400 HMz

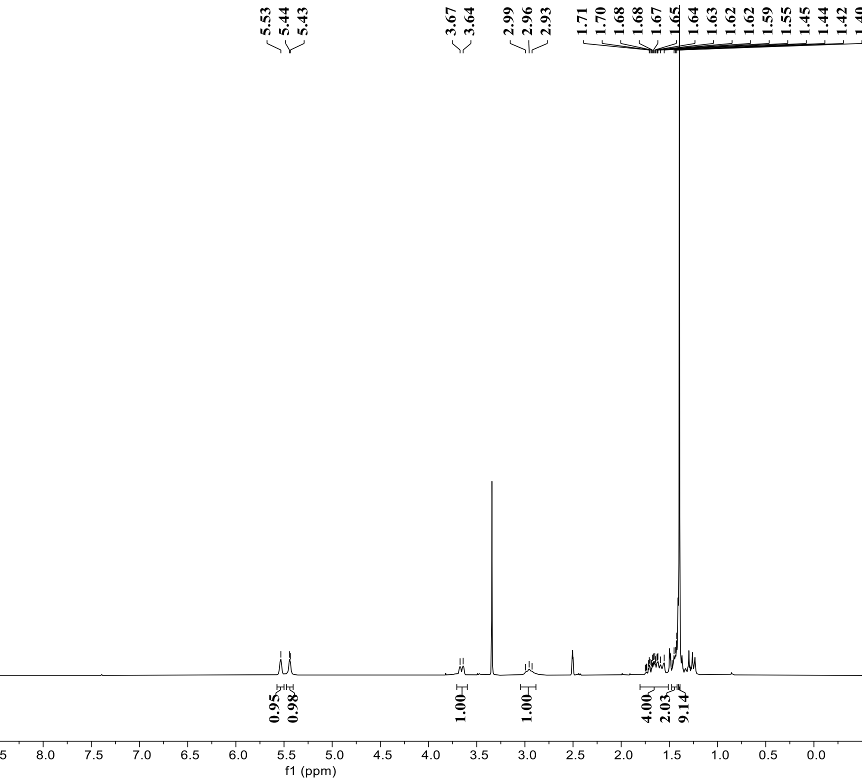

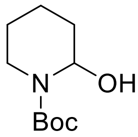

79 ^{13}C NMR
DMSO-d_6, 100 HMz

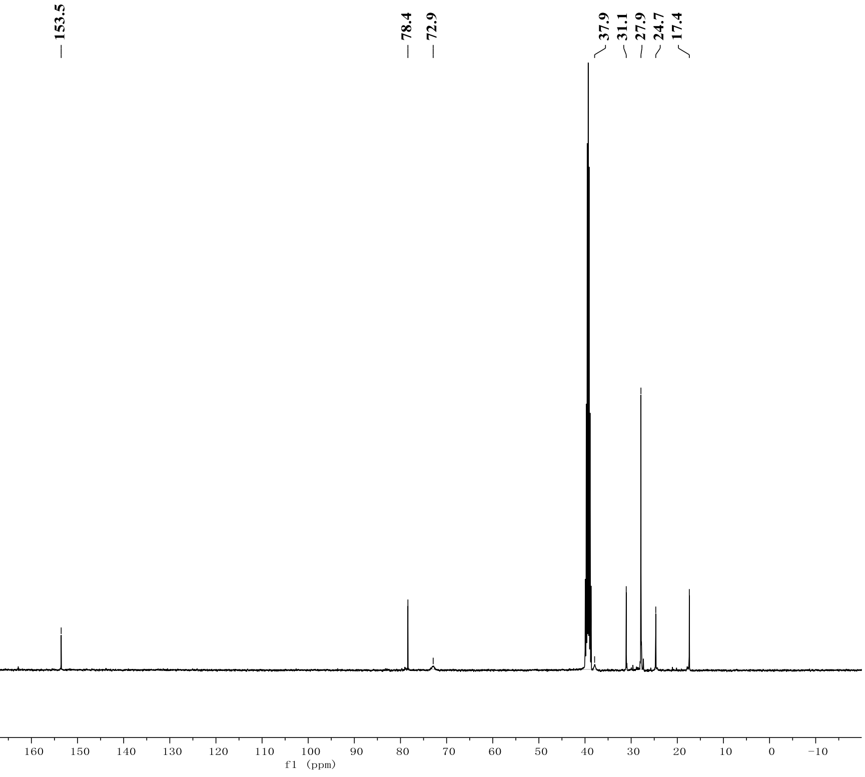

4 Supplementary References

1. Osberger, T. J., Rogness, D. C., Kohrt, J. T., Stepan, A. F. and White, M. C. Oxidative diversification of amino acids and peptides by small-molecule iron catalysis. *Nature.* **537**, 214-219 (2016).

2. Roque, J. B., Kuroda, Y., Göttemann, L. T. and Sarpong, R. Deconstructive fluorination of cyclic amines by carbon-carbon cleavage. *Science.* **361**, 171-174 (2018).

3. Roque, J. B., Kuroda, Y., Göttemann, L. T. and Sarpong, R. Deconstructive diversification of cyclic amines. *Nature.* **564**, 244-248 (2018).

4. Zeng, S., *et al.* Selenium-Electrocatalytic Cyclization of 2-Vinylanilides towards Indoles of Peptide Labeling. *Chem Asian J.* **17**, e202200762 (2022).

5. San Segundo, M. and Correa, A. Site-Selective Cu-Catalyzed Alkylation of α-Amino Acids and Peptides toward the Assembly of Quaternary Centers. *ChemSusChem.* **11**, 3893-3898 (2018).

6. Roy, S., Kumar, G. and Chatterjee, I. Photoinduced diverse reactivity of diazo compounds with nitrosoarenes. *Org. Lett.* **23**, 6709-6713 (2021).

7. Ou, C.-H., Pan, Y.-M. and Tang, H.-T. Electrochemically promoted N-heterocyclic carbene polymer-catalyzed cycloaddition of aldehyde with isocyanide acetate. *Sci. China Chem.* **65**, 1873-1878 (2022).

8. Lü, S., Wang, Z., Gao, X., Chen, K. and Zhu, S. 1, 2-Difunctionalization of Acetylene Enabled by Light. *Angew. Chem. Int. Ed.* **135**, e202300268 (2023)

9. Soro, D. M., *et al.* Photo- and Metal-Mediated Deconstructive Approaches to Cyclic Aliphatic Amine Diversification. *J. Am. Chem. Soc.* **145**, 11245-11257 (2023).

10. Tran, K., *et al.* Constraining the side chain of C-terminal amino acids in apelin-13 greatly increases affinity, modulates signaling, and improves the pharmacokinetic profile. *J. Med. Chem.* **64**, 5345-5364 (2021).

11. Greßies, S., Klauck, F. J., Kim, J. H., Daniliuc, C. G. and Glorius, F. Ligand-enabled enantioselective C–H activation of tetrahydroquinolines and saturated Aza-heterocycles by RhI. *Angew. Chem. Int. Ed.* **57**, 9950-9954 (2018).

12. Liu, R.-H., He, Y.-H., Yu, W., Zhou, B. and Han, B. Silver-Catalyzed Site-Selective Ring-Opening and C–C Bond Functionalization of Cyclic Amines: Access to Distal Aminoalkyl-Substituted Quinones. *Org. Lett.* **21**, 4590-4594 (2019).

13. Cao, J., *et al.* Novel leucine ureido derivatives as aminopeptidase N inhibitors using click chemistry. *Bioorg Med. Chem.* **26**, 3145-3157 (2018).

14. Liu, C.-F., Wang, H., Martin, R. T., Zhao, H., Gutierrez, O. and Koh, M. J. Olefin functionalization/isomerization enables stereoselective alkene synthesis. *Nat Catal.* **4**, 674-683 (2021).

15. Dai, P.-F., Qu, J.-P. and Kang, Y.-B. Organocatalyzed Aerobic Oxidation of Aldehydes to Acids. *Org. Lett.* **21**, 1393-1396 (2019).

16. Suzuki, T., Fujimura, M., Fujita, K. and Kobayashi, S. Total synthesis of (+)-methynolide using a Ti-mediated aldol reaction of a lactyl-bearing oxazolidin-2-one, and a vinylogous Mukaiyama aldol reaction. *Tetrahedron.* **73**, 3652-3659 (2017).

17. Kato, M. and Saito, A. Domino Synthesis of 2,3-Dialkylidenetetrahydrofurans via Tandem Prins Cyclization–Skeletal Reorganization. *Org. Lett.* **20**, 4709-4712 (2018).

18. Wang, X., Xun, X., Song, H., Liu, Y. and Wang, Q. Palladium Metallaphotoredox-Catalyzed 2-Arylation of Indole Derivatives. *Org. Lett.* **24**, 4580-4585 (2022).

19. Ruan, Z., Sauermann, N., Manoni, E. and Ackermann, L. Manganese-Catalyzed C−H Alkynylation: Expedient Peptide Synthesis and Modification. *Angew. Chem. Int. Ed.* **56**, 3172-3176 (2017).

20. Neese, F. The ORCA program system. *WIRES Comput. Mol. Sci.* **2**, 73-78 (2012).

21. Neese, F. Software update: the ORCA program system, version 4.0. *WIRES Comput. Mol. Sci.* **8**, e1327 (2018).

22. Goerigk, L. and Grimme, S. Efficient and Accurate Double-Hybrid-Meta-GGA Density Functionals—Evaluation with the Extended GMTKN30 Database for General Main Group Thermochemistry, Kinetics, and Noncovalent Interactions. *J. Chem. Theory Comput.* **7**, 291-309 (2011).

23. Caldeweyher, E., Bannwarth, C. and Grimme, S. Extension of the D3 dispersion coefficient model. *J. Chem. Phys.* **147**, 034112 (2017).

24. Caldeweyher, E., *et al.* A generally applicable atomic-charge dependent London dispersion correction. *J. Chem. Phys.* **150**, 154122 (2019).

25. Weigend, F. and Ahlrichs, R. Balanced basis sets of split valence, triple zeta valence and quadruple zeta valence quality for H to Rn: Design and assessment of accuracy. *Phys. Chem. Chem. Phys.* **7**, 3297-3305 (2005).

26. Marenich, A. V., Cramer, C. J. and Truhlar, D. G. Universal Solvation Model Based on Solute Electron Density and on a Continuum Model of the Solvent Defined by the

Bulk Dielectric Constant and Atomic Surface Tensions. *J. Phys. Chem.* **113**, 6378-6396 (2009).

27. Stoychev, G. L., Auer, A. A. and Neese, F. Automatic Generation of Auxiliary Basis Sets. *J. Chem. Theory Comput.* **13**, 554-562 (2017).

28. Zhao, Y. and Truhlar, D. G. The M06 suite of density functionals for main group thermochemistry, thermochemical kinetics, noncovalent interactions, excited states, and transition elements: two new functionals and systematic testing of four M06-class functionals and 12 other functionals. *Theor. Chem. Acc.* **120**, 215-241 (2008).

29. Grimme, S., Antony, J., Ehrlich, S. and Krieg, H. A consistent and accurate ab initio parametrization of density functional dispersion correction (DFT-D) for the 94 elements H-Pu. *J. Chem. Phys.* **132**, 154104 (2010).

30. Tomasi, J., Mennucci, B. and Cancès, E. The IEF version of the PCM solvation method: an overview of a new method addressed to study molecular solutes at the QM ab initio level. *J. Mol. Struct(Theochem).* **464**, 211-226 (1999).

31. Lu, T. and Chen, Q. Shermo: A general code for calculating molecular thermochemistry properties. *Comput. Theor. Chem.* **1200**, 113249 (2021).

32. Grimme, S. Supramolecular Binding Thermodynamics by Dispersion-Corrected Density Functional Theory. *Chem. Eur. J.* **18**, 9955-9964 (2012).

33. Feng, T., *et al.* Electrochemical dual α,β-C(sp3)–H functionalization of cyclic N-aryl amines. *Green Chem.* **25**, 2681-2689 (2023).

34. Ho, J., Klamt, A. and Coote, M. L. Comment on the Correct Use of Continuum Solvent Models. *J. Phys. Chem.* **114**, 13442-13444 (2010).

35. Roth, H. G., Romero, N. A. and Nicewicz, D. A. Experimental and calculated electrochemical potentials of common organic molecules for applications to single-electron redox chemistry. *Synlett.* **27**, 714-723 (2016).

36. Isse, A. A. and Gennaro, A. Absolute Potential of the Standard Hydrogen Electrode and the Problem of Interconversion of Potentials in Different Solvents. *J. Phys. Chem.* **114**, 7894-7899 (2010).

37. Mayer, I. Charge, bond order and valence in the AB initio SCF theory. *Chem. Phys. Lett.* **97**, 270-274 (1983).

38. Mayer, I. Bond order and valence: Relations to Mulliken's population analysis. *Int. J. Quantum Chem.* **26**, 151-154 (1984).

39. CYLview20; Legault C. Y. *Université de Sherbrooke*. http://www.cylview.org, (2020**)**.

40. Endo, T., Fukunaga, T., Yoshimura, T. and Esumi, K. Scavenging DPPH radicals catalyzed by binary noble metal–dendrimer nanocomposites. *J Colloid Interface Sci.* **302**, 516-521 (2006).
